# The functional role of glial cells in the pathologic brain as reviewed by Alois Alzheimer in 1910

**DOI:** 10.1186/s13024-026-00933-5

**Published:** 2026-02-13

**Authors:** Helmut Kettenmann, Hans Lassmann, Bilge Ugursu, Xianyuan Xiang

**Affiliations:** 1 Bio-X International Institute, Faculty of Life and Health Sciences, Shenzhen University of Advanced Technology, Shenzhen, China; 2 Max-Delbrück Center for Molecular Medicine in the Helmholtz Association, Berlin, Germany; 3 Center for Brain Research, Medical University of Vienna, Vienna, Austria

**Keywords:** Alois Alzheimer, Pathologic neuroglia, Ameboid glia, Glial granule cells, Microglia, Astrocytes, Neuroinflammation

## Abstract

Alois Alzheimer is known for the clinical diagnosis and neuropathological analysis of the neurodegenerative disease named after him. Yet, a less celebrated but equally fundamental contribution lies in his monograph, “Contributions to the knowledge of the pathologic neuroglia and their relationship to degenerative processes in the nervous tissue.” This work, now translated, meticulously details the dynamic role of glial cells in brain pathology, a paradigm-shifting concept for its time. Alzheimer applied then-novel staining techniques -including adaptations of Scharlach red and Mallory hematoxylin- to visualize lipids and protoplasmic degradation in post-mortem human brains. These approaches revealed striking changes in the glial network across disorders ranging from stroke and trauma to multiple sclerosis, syphilis-related dementia, various forms of neurodegeneration, and schizophrenia. He documented the appearance of two novel glial forms, which he termed “ameboid glia” and “glial granule cells”, involved in the phagocytosis of “fatty matter” and “lipoid substances” from degenerating nervous tissue. He also documented “neuronophagia,” where glial cells appeared to directly interact with and “dissolve” damaged neurons. This work laid the conceptual groundwork for modern neuroscience. We now recognize Alzheimer’s “ameboid glia” as the earliest documented observations of activated microglia and astrocytes, and his descriptions of glia’s engagement with cellular damage provided the first evidence for what we now call neuroinflammation. His methodical approach and detailed observations proved that glial cells are not passive bystanders but central, active players that sense, respond to, and shape the course of brain diseases. The English translation and the original German text can be found in the attachment.

**Supplementary information:**

The online version contains supplementary material available at 10.1186/s13024-026-00933-5.

## Glial cells change their phenotype under pathologic conditions

Beginning in the late 19^th^ century, anatomical and pathological studies of the brain in health and disease started to unravel the nature of damage, that explained different diseases or disease states. It started with the description of changes, seen in focal lesions such as those present in stroke, brain trauma or multiple sclerosis (for examples see [1–5]), but the identification of the pathological substrate of neurological and psychiatric diseases with diffuse affection of the central nervous system proved to be more challenging. An important contribution to this topic was provided by Alois Alzheimer on the pathologic neuroglia in neurodegeneration, published in 1910 in German. We now provide an English translation and the original German text in the attachment to this publication. For the convenience of the reader, we have inserted figures corresponding to the text at the appropriate position in the translation. In this English translation, we also refer to terms which are no longer used today and explain them in modern language. This list of 36 comments can be found at the end of the English translated text and in the detailed description of the various aspects of granular degeneration, summarized in the original table on pages 530 to 535.

In this study Alzheimer outlined a common reaction pattern of the nervous system in response to tissue damage in a variety of different inflammatory, metabolic or neurodegenerative conditions. He outlines the differences between focal destructive lesions (e.g. stroke) and conditions of diffuse brain damage (most likely severe inflammatory conditions such as for instance neurotuberculosis) with the following description:

***If the nervous tissues together with the support tissue is completely destroyed, may it be to a trauma, a bleeding or a softening, we observe that in the first place the mesodermal elements fulfill the task to remove the dead nervous substance. From the rim of the focus, newly formed vessels invade into the dead tissue; granule cells formed from the cells of the vessel wall migrate into the environment, take up part of the dead material and convert it to fat. In the meantime, the glia proliferates around the focus but only contributes to the cleaning process at areas where degradation products are generated within the still remaining (living) nervous tissue. It eagerly forms new fibrous glial tissue which encapsulates the focus. Finally, the fatty products in the granule cells are dissolved and degenerate with the granule cells. In the focus remain the newly formed vessels, the fibrous connective tissue and the massively formed glial surface layer****(page 532)*

***“It is different, when the damage selectively affects the nervous tissue resulting in a larger area of degradation and degeneration, while the support tissue is not affected. Now the glial cells fulfill the cleaning process from the beginning.”****(p. 524)*. These patterns of lesion formation have been described in a number of studies before and one particularly impressive description of focal versus diffuse brain damage is found by Otto [3] in the description of the neuropathology of acute multiple sclerosis [6].

However, in situations of milder or protracted tissue injury, a different pattern of glia reaction occurs, which is described in extensive detail in the manuscript by Alois Alzheimer [7], which is discussed here. The reaction to tissue damage in the brain and spinal cord parenchyma is basically handled by resident glial cells, and the glia reaction uncovers sites of damage, even when acute injury or loss of neurons is not apparent. However, the simple notion that this always leads to the formation of a glial scar is incorrect: ***“A pathologic increase in glial fibers indicates only to a limited extent a deterioration of nervous elements. Where it is present, a degeneration of nervous elements may have happened, but a lack of such an increase is not evidence that nervous elements were not damaged.” (p. 403)***

**Fig. 1:**
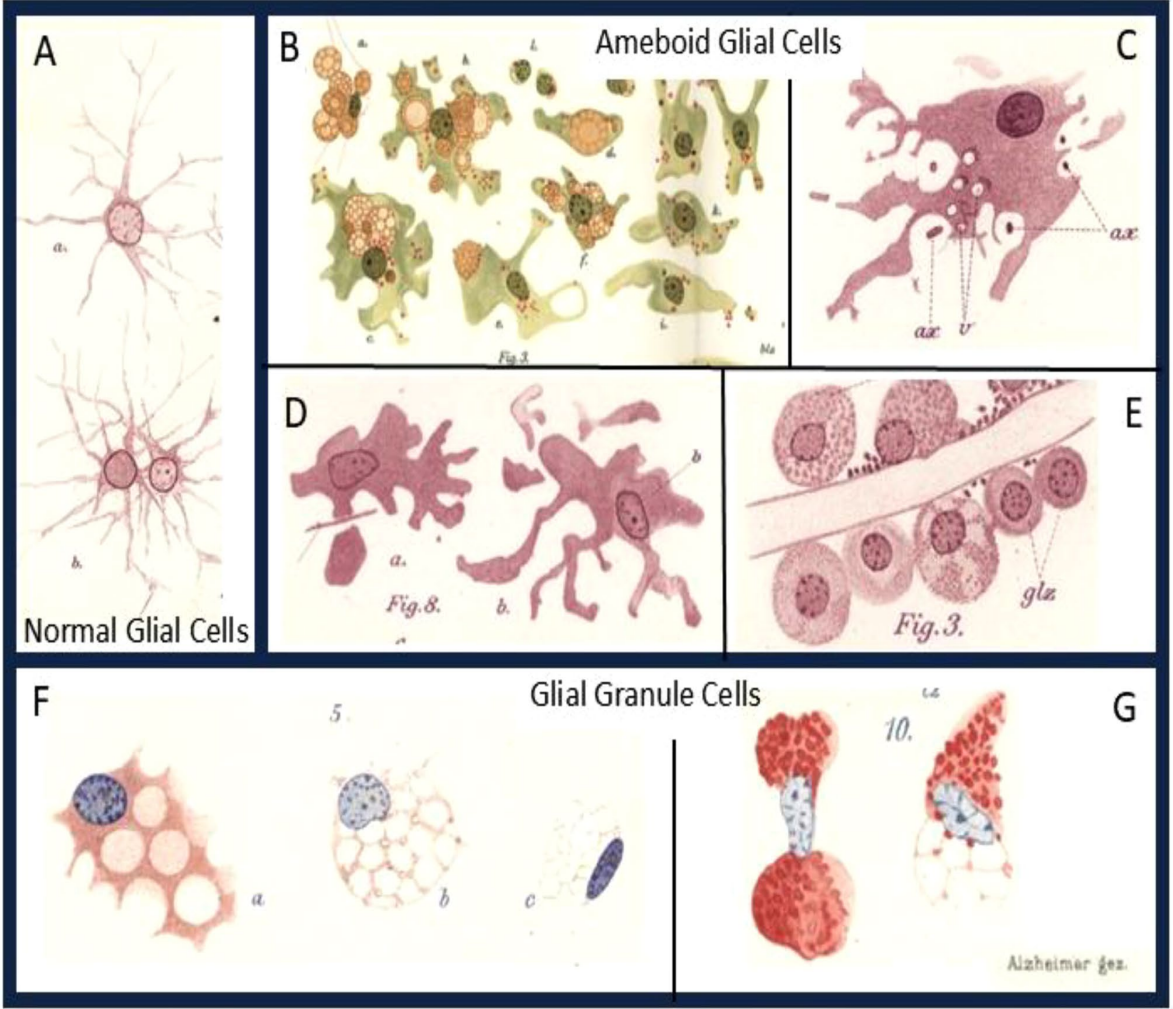
Normal and pathologic glial cells. **A**, Normal glial cells with protoplasmic branches from a human accident victim. **B-E**, Ameboid glial cells from human cases with different neurologic diseases. **F, G** Glial grid or granule cells from human cases with different neurologic diseases. Alzheimer signed his tables with ´*Alzheimer gez’* (drawn by Alzheimer). This signature is inserted to all the figures. (**ax.**, axon; **glz.**, glial cell; **v.**, probably vesicle, not in the abbreviation list). **A**, Table XXVIII, Fig. 1a, b; **B**, Table XXX, Fig. 3; **C**, Table XXVIII, Fig. 6; **D**, Table XXVIII, Fig. 9; **E**, Table XXVIII, Fig. 3; **F**, Table XXXIII, Fig. 5; **G**, Table XXXII, Fig. 10

Alzheimer then described that a key function of glia is to degrade damaged tissue and to transport the debris into the perivascular or subarachnoid space, where it is partly taken up by mesenchymal cells and removed from the central nervous system (CNS) compartment by some form of lymphatic drainage. According to Alzheimer this process is initiated by a transformation of the process bearing glia of the normal brain into two forms, into ameboid glia cells and gliogenic granule cells (Fig. 1).

## The ameboid glial cells

***Already in my publication “Histologische Untersuchungen zur Differentialdiagnose der progressiven Paralyse” I have described and illustrated glial cells, which show an enlarged protoplasmic cell body and disintegrate without producing glial fibers. I have later termed these cells “ameboid glial cells”, since they deviate from the so far known forms of glial morphologies and since they look like an ameba. Studies on different autopsy materials have revealed that these are abundant in acute disease states of the nervous system. With the appearance of the ameboid glial cells, there is a wide-spread disappearance of the classic glial elements.*** *(page 422).*

The transformation into an ameboid phenotype has later been described in the process of microglia activation by Pio del Rio-Hortega in 1919 and the term ameboid microglia is still widely used until now [8]. However, in Alzheimer’s account, this term is used to describe a general pattern of glia activation. The key features are beading and loss of cell processes, accompanied by a profound increase in the perinuclear cytoplasm, which is a cellular reaction, occurring not only in microglia, but also in astrocytes (Fig. 1D) and sometimes in oligodendrocytes, for instance in conditions of severe chronic inflammation. As an example, luetic encephalitis was one of the most common neuropathologoical conditions seen at the time this article was published. However, when such dynamic changes are associated with the intracellular appearance of granules and vacuoles they are typical in the early stages of microglia activation (Fig. 1B). Microglia have been recognized as the immune-related cells of the central nervous system [9] and the role of pyroptosis has been recognized as a pro-inflammatory mechanism of cell death downstream of the inflammasome and its relation to neurodegeneration [10]. Today the focus of neurodegeneration is biased towards Alzheimer´s disease. In the manuscript, discussed here, Alzheimer described neurodegeneration as a broad concept. It should be noted that he published his histopathological description of the disease which was later named after him [11] one year after he published this article on the pathologic glia. Based on his figures, it seems also likely that some of the cells which he described could be astrocytes and it is now known that glial activation can lead to astrogliosis and the formation of glial scars [12, 13].

Alzheimer continues: ***“We will start with the description of ameboid cell changes in the white matter since they are more common there, have more massive forms and are easier to study in the white matter. One can observe a clearly defined, small cell body around the nucleus. The nucleus is small and rich in chromatin. With increasing size of the cell body, the nucleus does not increase significantly. Thus, the small size of the nucleus is a significant feature of the ameboid glial cells.****(pp. 423, 424)****. While small cysts accumulate in the cell body of large ameboid cells, the cytoplasm becomes sparce and less well stained. Finally, one gets the impression that the cysts melt away and the whole cell disintegrates, while also the nucleus undergoes regressive alterations.”****(pp. 426, 427).* Alzheimer’s description of the disintegration anticipates the changes defined as microglia dystrophy or microglia senescence in the recent literature [14], which include the loss of cell processes, the global cell shrinkage, the occasional accumulation of intracytoplasmic lipoid material, the condensation of nuclear chromation and fragmentation of the nuclei.

He then describes that ameboid cells appear very early in the formation of the lesions, they arise in part from existing glia cells (Fig. 1B–E) and in part from cells undergoing proliferation. They are seen in close contact with intact or degenerating neurons and axons and are also clearly attached to the perivascular or subpial space. From this he concludes: ***The condition of their appearance may indicate the function of ameboid glial cells. One does not find ameboid glial cells in the adult, normal brain. Cells with similar shape can be found in the fetal nervous system when the system is built up.”****(page 458).*

It is established today that microglial cells invade the brain early in development. These embryonic cells have an ameboid shape as Alzheimer noted. Subsequently, microglial cells obtain a ramified phenotype at later developmental stages, and it appears as if these cells disappear. Hortega in his seminal series of 4 papers in 1919 concluded that the ameboid cells in pathology originate from the ramified form. Alzheimer did not yet recognize this, and he continues:

***Based on the comparison of clinical and anatomic data, it implies that the appearance of ameboid glial cells is related to the degradation of nervous tissue. (page 461). When ameboid cells are found in larger amounts in the white matter, in most cases, one can find degradation processes in the nervous tissue proper, in the axons and in the myelin. Screening a larger set of material creates the impression that the formation of ameboid glial cells can start before the degradation processes can be verified, yet that those are rarely missing where many and partially regressive ameboid cells are found in the tissue.****(page 462)*

However, Alzheimer clearly recognized that ameboid cells might arise in response to degeneration or pathological products is remarkably prescient. This can be interpreted in light of current understanding of metabolic disturbances or proteinopathy long before degeneration and obvious morphological changes become detectable in the microscope.

***Substances arising from the degradation of the nervous tissue may trigger the formation of the ameboid cells. This may not explain the rare cases in which one finds ameboid cells without obvious nervous system degradation. Maybe in these cases, nervous structures degrade which we cannot yet illustrate, or perhaps pathological products are generated which cannot yet be detected with the microscope—not by a degradation of nervous elements, but due to a disturbance of their consumption—and they aid in their removal.****(page 469)*

**Fig. 2:**
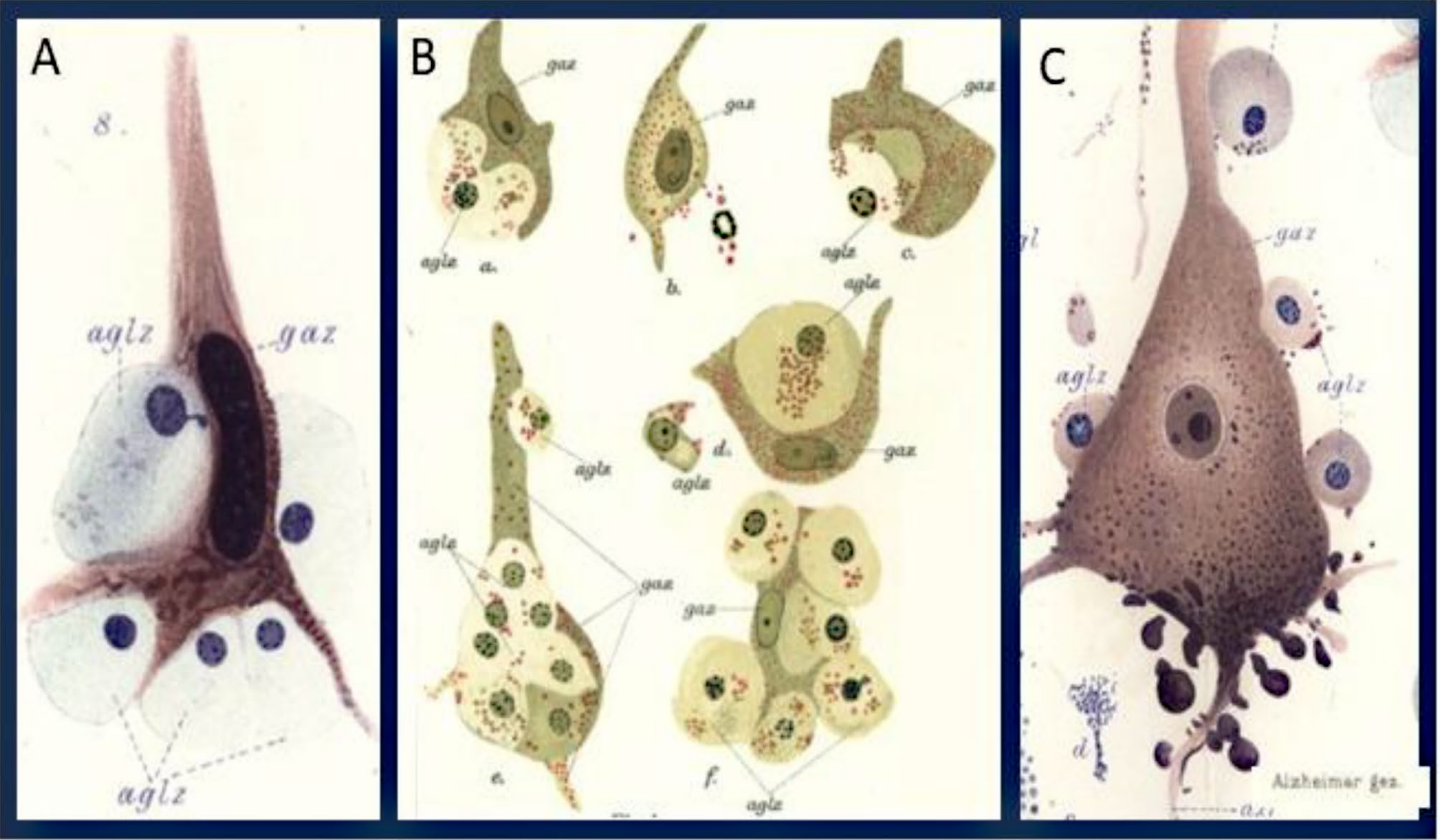
Ameboid glial cells interact with ganglion cells (neurons) and phagocytic degradation. **A**, This image illustrates a ganglion cell surrounded by five ameboid glial cells, taken from a case with a severe neurologic disease. One of the glial cells is shown deforming the ganglion cell’s nucleus by encroaching upon it. The glial cell nucleus itself shows signs of early degeneration. **B**, A series of images from the cortex of a patient with delirium following acute progressive paralysis. This figure demonstrates the deformation of ganglion cells and the eventual replacement of their cell bodies by ameboid glial cells. It also shows the degeneration of the ameboid glial cell nuclei. **C**, A Beetz pyramidal neuron from a patient with delirium after acute paralysis shows profound nuclear pathology consistent with Nissl’s description of severe cellular illness. Basophilic granules are evident along the apical dendritic process and adjacent protoplasmic extensions, whereas drop-like accumulations of basophilic material are localized to the basal soma and axonal hillock. Surrounding ameboid glial cells are in close apposition to the neuronal soma, underscoring their role in engulfing and processing degenerating neuronal structures. (**gaz.**, ganglion cell; **aglz.**, ameboid glial cells). **A**, Table XXXIII, Fig. 8; **B**, Table XXX, Fig. 4; **C**, Table XXXIII, Fig. 7

When he studied the dynamic changes in ameboid glia cells in relation to tissue injury in the lesions he concluded that their main function is the phagocytosis of cell or tissue fragments (Fig. 2). Thus, in diffuse damage of the brain tissue neuro-phagocytosis nearly exclusively occurs in “*neuro-phagocytes of glial origin*”. By analyzing many different conditions, Alzheimer claims that ameboid microglia can take up a broad range of endogenous and exogenous materials, including blood pigments, ink particles, carmine or vermillion after their injection into the brain tissue. He concludes that **“this indicates that the cells only remove substances which are already damaged due to the disease process and are destined for decay”.***(page 468)*.

Alzheimer is convinced that these ameboid cells can migrate in the brain: ***“To clearly understand the processes which occur around the vessels, it is essential to know whether glial cells have the capacity to migrate. Many authors are convinced that they can migrate. For some types of glial cells this may not be possible due to their complex morphology. The roundish ameboid forms could translocate based on their morphology.”****(page 472).* Having taken up debris into their cytoplasm the ameboid cells start to degrade the material, for instance by transforming myelin material into granules stained by fuchsin and methyl blue or into lipid droplets. Then this material will be taken up by mesenchymal cells in the perivascular space or the meninges. Alzheimer, however, acknowledges that these processes are well understood in the white matter, while the structural features and functions of ameboid glia are more complicated in the grey matter.

## Neuronophagia

Comparing ameboid microglia in different human diseases or lesion stages, Alzheimer noted that in all conditions these cells were involved in clearance of damaged material. However, it remained unclear whether these cells are actively involved in the induction of tissue injury or cell degeneration. An example, where this might be the case was suggested by him to be neuronophagia. The ameboid microglia are seen in close contact with nerve cell bodies in the initial stages of injury, they then take up neuronal material in cytoplasmic granules and form a cluster or nodule of cells at the site, where the neuron had previously existed (Fig. 2; *pages 476–478*).

These observations contributed to the hypothesis that glial cells are not only passive responders to injury but could also participate in active mechanisms of neurodegeneration. This concept has been substantiated by modern studies, particularly in microglia, the resident immune cells of the CNS. Under physiological conditions, microglia regulate neural development through phagocytosis of synaptic elements and apoptotic cells, functions that are essential for circuit maturation [15, 16]. Under pathological conditions, however, they may adopt a reactive phenotype characterized by pro-inflammatory signaling and promotion of neuronal dysfunction. For instance, microglia mediate complement-dependent engulfment of synaptic elements, contributing to cognitive decline in Alzheimer’s disease models [17]. Their reactivity can result in sustained release of neurotoxic mediators, including reactive oxygen species (ROS), nitric oxide (NO), and pro-inflammatory cytokines such as TNF-α and IL-6, which disrupt mitochondrial function and promote apoptosis in neurons [18]. These effects may represent active drivers of disease progression. Alzheimer described similar phenomena, stating that ***“ameboid glial cells deform the cell body, the processes and the nucleus,”*** and proposed that glial reactivity may not simply follow neuronal death but may ***“destroy ganglion cells in an active fashion”****(page 477).*

However, glial phagocytosis is not intrinsically detrimental. Microglial engulfment of apoptotic cells can also induce anti-inflammatory pathways and promote tissue repair. The functional consequences of phagocytosis depend on both the “bait” and the surrounding inflammatory milieu. Alzheimer acknowledged this complexity, noting that ***“we do not observe that the glial cells take up debris of decaying nervous tissue components,”****(page 477),* suggesting that glial cell response is highly context dependent. He also stated that ***“defined nervous system diseases are correlated to defined alterations in glia”****(page 403),* which again implies the context and pathology-dependent glial response that we know today.

## The gliogenic granule cells

The second form of glial cells which Alzheimer finds in pathologic tissue he terms gliogenic granule cells (Gliogene Körnchenzellen), glial grid cells (Gliale Gitterzellen) or glial granule cells or often simply granule cells (Fig. 1 F, G). He clearly states that these cells originate from glial cells by conversion into this new phenotype. Their name is due to the fact that they contain granules which result from the uptake of degraded white matter material, predominantly myelin. ***“In some brains in which ameboid glial cells are found in white matter, peculiar cells can be found there and in the grey matter which can be depicted with the Weigert glia method. Table XXXI provides a picture. They are characterized by distinct granules which are stained blue with this method and which I will term fibrinoid. The name should not determine anything with respect to the chemical nature of these granules”****(pages 432,433)*. Alzheimer distinguishes these granule cells from the ameboid glial cells: ***“The more or less uniform image of these cells in the white matter corresponds to different forms in the grey matter. There we observe elements similar to those in white matter, but also others which deviate with respect to their form so much that they cannot be termed ameboid cells. All these cells have in common that during their regression different granules appear which we now have recognized as follows: the fuchsinophil, the light-green granules, the lipoid granules and cysts, the methylene blue granules, and the fibrinoid granules.”****(pages 434,435).* Alzheimer’s histological studies of glial cells, although limited by the technical constraints of his time, already revealed early indications of glial heterogeneity. His morphological classification of glial cells into “*ameboid*” and “*granular*” forms challenged the prevailing concept of glia as passive structural components. This functional perspective anticipated the extent of glial heterogeneity, which is now better understood through recent advances in single-cell transcriptomics. Single-cell RNA sequencing (scRNA-seq) has identified, for instance, multiple transcriptional states in microglia, including homeostatic, interferon-responsive, inflammatory, and disease-associated states [19]. Disease-associated microglia (DAM), initially characterized in Alzheimer’s disease models [20] have since been reported also in other neurodegenerative conditions, indicating conserved transcriptional programs in disease contexts [21]. Integration of additional omics technologies, including ATAC-seq, proteomics, and metabolomics, has further expanded the definition of different glial states. Yet single-cell and spatial omics remain correlative, and these correlative findings must be validated using biochemical, genetic, cell biological and neuropathological approaches to determine functionality and their relevance in the pathophysiology of human disease. As Alzheimer himself wrote in those days **“our current technology is insufficient. We do not yet know any method which will display the details of the nervous structure in a sufficient and reliable manner useful for pathological analysis”***(page 404).* Even today, modern tools only incompletely reveal the details of the molecular characterization of glial heterogeneity and its association with different pathologies.

## Glial cells convert myelin and axonal breakdown products into intracellular fat

**Fig. 3:**
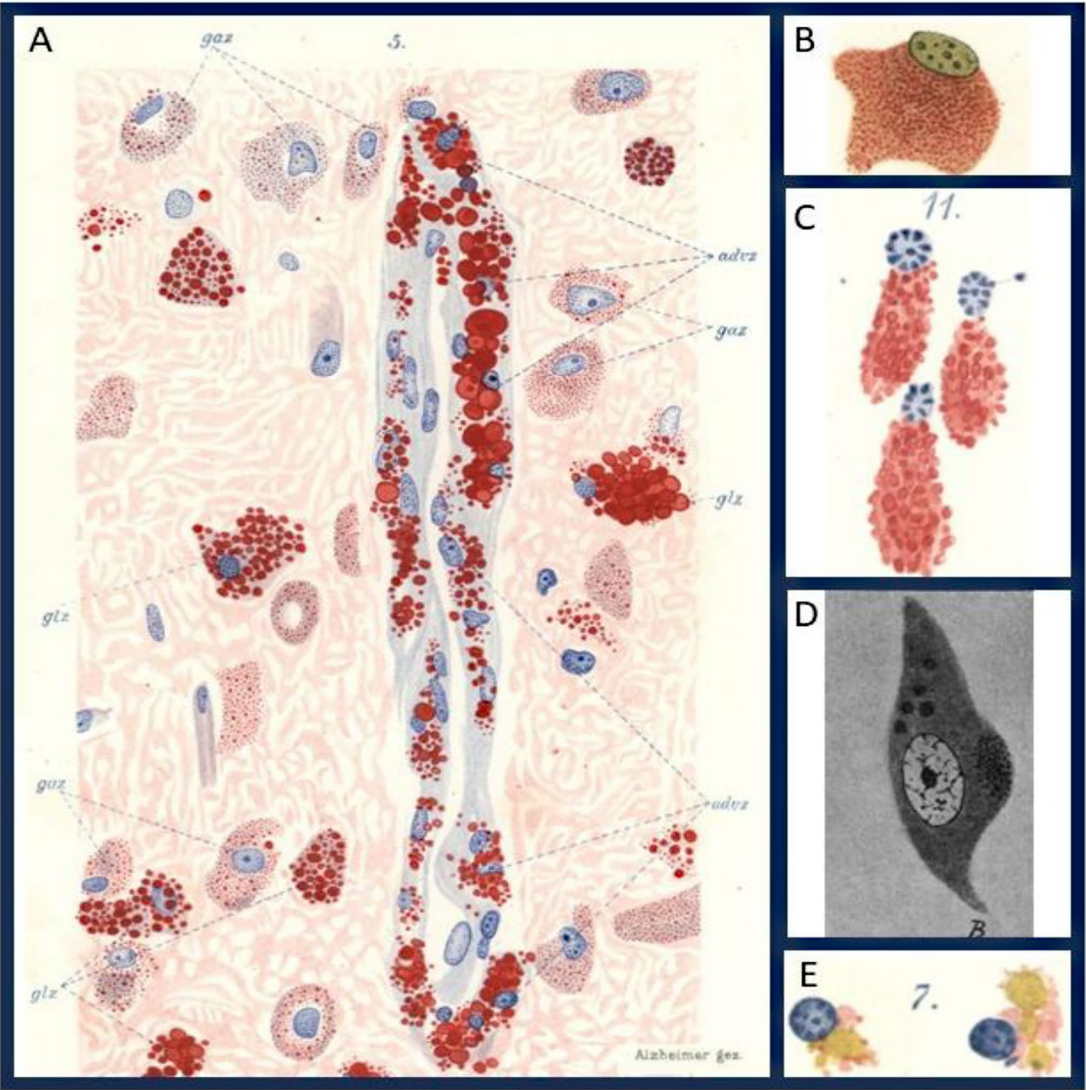
Lipid accumulation in neurons and glial cells across distinct neurodegenerative disorders. **A**, Glial cells and adventitial cells from the cortex of a case of Warren–Tay–Sachs amaurotic idiocy showing abundant Scharlach-positive lipid. Ganglion cells contain fine dust-like fat particles, whereas glial cells display large fatty inclusions, with prominent lipid accumulation in the adventitia. **B**, Glial cell with complete replacement of the cytoplasm by delicate fuchsinophil granules. **C**, Three glial cells from the white matter of the midbrain of a patient with progressive chorea after brief alcohol fixation and toluidine blue staining. It shows how abundantly these substances are deposited in glial cells. **D**, Coarse granular fatty degeneration in ganglion cells of the hippocampus from a patient with senile dementia. In addition to yellow-green stained neutral fat, five large basophilic lipid granules are visible, underscoring the heterogeneity of lipid products accumulating within neuronal soma. **E**, Two glial cells from the white matter of a case of senile dementia containing basophilic, metachromatic, and greenish lipoid substances. (**gaz.** ganglion cell; **advz.** adventitial cells; **glz.**, glial cells). **A**, Table XXXIV, Fig. 5; **B**, Table XXX, Fig. 6; C, Table XXXII, Fig. 11; **D**. Text Fig. 4; **E**. Table XXXII, Fig. 7

He recognized that glial cells can convert breakdown products into intracellular fat, with lipoid inclusions forming inside glia (often through transitions from fuchsinophil granules to lipoid inclusions), not as extracellular fat (Fig. 3).

***They seem to assimilate the substances arising from the dissolution and convert them within their body into fatty substances. Nowhere do we find free fat in such tissue. Fat is generated within the glial cell. The similar size of the fat granules within a given cell hints at their intracellular generation.” (page 468).*** He also described the process by which ***“The glial cells convert into fatty granule cells by circumventing the white matter clumps which are generated from the decaying myelin sheath. They dissolve those and convert it within their cell bodies into fat.****(page 523).*

Alzheimer interpreted these glial granule cells as the glial phagocytic and processing arm of degeneration, which convert breakdown products to intracellular fat and ultimately handing material off toward perivascular or adventitial compartments: *“The degeneration continues by a transfer of the fatty substances from the nervous system into the adventitial lymphatic spaces.” (page 468*, Fig. 3).

He also obviously describes transition states in which only part of the cell takes up material while the other part still has the shape of a branched glial cell. “***Occasionally it occurs that such glial cells completely convert into small fatty granule cells. Sometimes only one part of the cell serves for the fat accumulation, while the remaining part is available for other functions.”****(Page 526)*

Alzheimer’s observation of “glial granule cells” foreshadow today’s ‘lipid-droplet-accumulating microglia’ (LDAM)—microglia that accumulate neutral-lipid droplets with aging and neurodegeneration, showing impaired phagocytosis, elevated ROS, and pro-inflammatory signaling [22]. He also emphasized the importance of these degenerative processes occurring in white matter. Myelin is a product of oligodendrocytes and oligodendrocyte degeneration occurs predominantly in white matter. Today it is evident that white matter aging and neurodegeneration further drive microglial diversity [23, 24]. Human studies have confirmed their relevance: in Alzheimer’s disease brain tissue and in human–mouse chimeric models, plaque-associated microglia accumulate lipid droplets, with this phenotype modulated by TREM2 risk variants [25]. Genetic factors further strengthen the link: APOE4 homozygotes show increased microglial lipid droplet formation, and conditioned media from lipid-rich microglia enhance neurotoxicity [26]. These phenotypes may be molecular correlates of Alzheimer’s ***“ameboid, lipid loaded glial cells”****(page 421).* He wrote ***“glial elements convert the different substances from which the myelin sheath is built up … into fat”****(page 467)* and emphasized that ***“the fat which we find within the ameboid cells is not simply taken up … they are not taken up by cells, but rather generated within the cells”****(pages 471, 472)*. Conceptually, Alzheimer’s early insights align strikingly with LDAM biology. He noted that (i) glia convert myelin and axonal breakdown products into intracellular fat, (ii) extracellular free fat is rarely observed, and (iii) accumulated material is routed toward adventitial or lymphatic compartments. This continuity underscores that LDAM, like Alzheimer’s glial granule cells, represent central players in neurodegeneration, linking lipid metabolism to impaired clearance, inflammation, and disease progression.

Alzheimer also speculated that the inclusions in the gliogenic granule cells are of a different molecular constituent than the inclusions in the ganglion cells, since they show different reactions to the different staining techniques. ***“There are a number of color reactions corresponding to the lipoid inclusions of the ganglion cells, which do not label the fatty substances of the gliogenic granule cells in the white matter … With respect to these staining reactions, the lipoid substances in the gliogenic granule cells respond differently and are stained either not at all or only to a low extent. These are just a few examples, and several others could be added. This indicates that we are dealing with a peculiar lipoid substance.”****page 486*, Fig. 3).

While direct comparative lipidomic data for neuronal lipid droplets are still limited, neurons generally avoid long-term lipid storage, instead exporting excess or oxidized fatty acids to astrocytes for sequestration and metabolism, glial cells serve as the primary sites of lipid accumulation and processing [27, 28].

## Perivascular and subpial lymphatic spaces

**Fig. 4:**
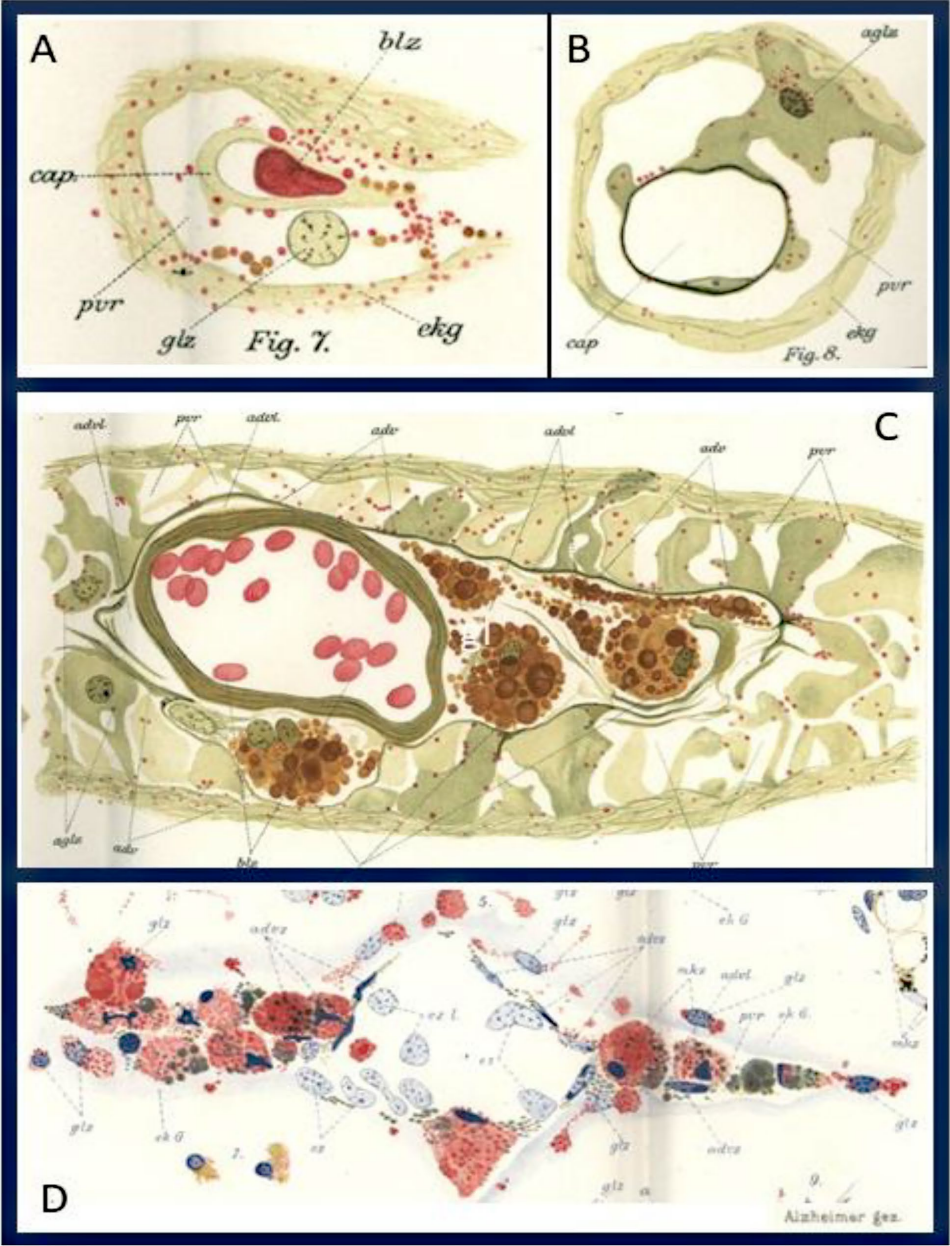
Early depictions of perivascular immune–glial interactions. These drawings anticipate the modern concept of immune competent glial cells engaged in phagocytosis, lipid metabolism, and vascular surveillance. **A, B**, Perivascular changes in capillaries from the cortex of a case of epileptic delirium. In the perivascular space resides a glial nucleus, from which rows of granules emerge formed by lipoid and fuchsinophil granules. They continue to the ectodermal tissue on one side and to the vessel wall on the other. Large ameboid glial cells with fuchsinophil granules, which resides with part of their cell body in the nervous tissue and with the othert they bridge the perivascular space and enwraps a capillary. **C**, Vein from the white matter of a case of a deadly proceeded psychosis of the degenerated age. The adventitial lymphatic space contains large granule cells loaded with lipoid material, while the perivascular space itself lacks such deposits, suggesting compartmentalized immune activity. **D**, Small vein from the white matter of an epileptic patient. The adventitial lymphatic space harbors granule cells almost completely filled with basophil metachromatic substances, including partially greenish-stained components. Similar deposits are also observed in glial cells of the surrounding ectodermal tissue, consistent with immune-glial interactions in pathology. (**cap.**, capillary; **blz.**, red blood cell; **pvr.**, perivascular space, **ekg.**, ectodermal tissue; **glz.**, glial cell; **aglz.**, ameboid glial cells; **adv.**, adventitia; **advl**, adventitial lymphatic space, **ez.**, endothelial cell; **l.**,vessel lumen; **mkz.**,mesodermal granule cell). **A**, Table XXX, Fig. 7; **B**, Table XXX, Fig. 8; **C**, Table XXX, Fig. 10; **D**, Table XXXII, Fig. 5

The accumulation of tissue debris in ameboid glia raises the question of whether the material is locally degraded or transported out of the CNS parenchyma. As mentioned above, Alzheimer suggests that the ameboid microglia can migrate in the CNS parenchyma due to the change of its cell shape and the loss of processes. Indeed, he describes further, that ***“glial cells are positioned half in the perivascular space, half in the nervous tissue,”*** and that ***“clumps and granules extend from the nervous tissue into these spaces”****(page 438*, Fig. 4). This provides an early morphological description of this bidirectional exchange. Thus, he noted that glial cells can migrate out of the CNS parenchyma into the perivascular space and the leptomeninges, as already described before in multiple sclerosis lesions [3]. These changes are seen in areas, where the glia limitans is damaged in the pathological process: ***“One can observe that the ectodermal tissue is still attached at the adventitia, but the glial cells which directly surround the vessel are largely dissolved into methyl blue granules. This generates gaps in the tissue which could generate a perivascular space due to the dissolvement of the limitans. Through such gaps ameboid glial cells could enter the perivascular space without much resistance. Similarly, degradation products of nervous and glial structures could enter partially in a solid, partially in a semi-fluid or fully fluid condition; they will precipitate in the tissue fluid which fills the space due to the fixation.”****(pages 437, 438).* He reported that ***“diverse, peculiar substances accumulate in the perivascular spaces wherever ameboid glial cells appear in the tissue in large number,”*** and noted that **“under pathological conditions perivascular spaces can be found which cannot be interpreted as artificial shrinkage spaces.”***(p. 441)*. These early observations anticipated current evidence indicating that the CNS is functionally connected to peripheral immune systems through specialized drainage routes. This concept has been substantiated by the discovery of functional lymphatic vessels within the dura, which may facilitate the clearance of cerebrospinal fluid, macromolecules, and immune cells to deep cervical lymph nodes [29, 30]. These structures provide anatomical support for Alzheimer’s early hypothesis that pathological products can exit the CNS through perivascular routes. Alzheimer further suggested that ***“in these perivascular spaces a substantial part of the degradation processes of the nervous system occurs”****(page 421)*, an assertion now supported by experimental studies demonstrating extracellular accumulation of neurotoxic substances at CNS interfaces. Glial cells, particularly astrocytes and microglia, play key roles in mediating this CNS-peripheral immune communication. Microglia respond to pathological stimuli by secreting cytokines, chemokines, and extracellular vesicles, which influence peripheral immune activity through diffusion into the meningeal lymphatic and choroid plexus compartments [31]. In turn, peripheral immune cells such as T lymphocytes can access the CNS via these entry points and modulate glial activation via direct signaling [32].

Similar changes, he also describes for the border between the glia limitans superficialis and the pia mater of the meninges *(page 443)*.

In summary, Alzheimer’s descriptions of perivascular glial degradation, extracellular deposits, and pia-associated changes represent early evidence of CNS-immune interface activity. Today, we have classified border associated macrophages (BAMs) as specific cells at that interface [33, 34]. Modern findings confirm that glial cells are not restricted to local homeostatic roles but actively regulate immune communication between the CNS and periphery. This reciprocal interaction may play a central role in neuroinflammatory conditions, including multiple sclerosis and Alzheimer’s disease [35, 36].

## Disease specific modifications of the basic pattern of glia reaction to CNS damage

Alois Alzheimer’s study is based on the analysis of a very broad spectrum of diseases, which includes many different infectious inflammatory diseases, various neurodegenerative diseases, vascular diseases, chronic epilepsy as well as various psychiatric diseases. In many cases, the nature of the diseases was at this time poorly defined and their etiology and pathogenesis were unknown. Others with very severe pathological substrates, like for instance chronic tuberculous or luetic meningoencephalitis, are hardly seen anymore today. Thus, the spectrum of pathological changes in these cases was very broad and heterogeneous. It is a major achievement that he was able to define a general pattern of tissue reactions, as described above, suggesting a common reaction pattern of the CNS to tissue injury irrespective of the underlying cause of the disease. However, he also noticed that there are many neurological diseases with severe clinical manifestations, where no ameboid glia reaction was observed and the brains did not differ from those of control patients. There were, for instance, patients dying with seizures in a state of delirium or with dementia precox (later called schizophrenia). These were apparently cases with functional brain damage in the absence of major structural brain damage. Another example, which was discordant with his description of the ameboid glia reaction was seen in patients with massive lipid loading of neurons and glia, including Tay Sachs disease or a disease with accumulation of metachromatic lipid material. In such diseases, which today are identified as lysosomal storage diseases, the ameboid glia reaction was masked by the massive overload of the cells with the respective lipids.

## Vision on neurochemistry and metabolomics

At Alzheimer´s time, almost nothing was known on the molecular composition of the brain. Proteins as a class of molecules were unknown and it was not until 1954 when Linus Pauling (1901–1994) from Los Angeles received the Nobel prize for characterizing the first protein, hemoglobin. It was only in 1965 that the International Society of Neurochemistry was founded having its first meeting in 1967 [37] and it took many more years until the first proteins were cloned. Even small molecules like neurotransmitters were unknown and it was only in 1921 that Otto Loewi from Vienna (1873–1961) discovered acetylcholine as a signalling substance in the heart, receiving the Nobel Prize in 1936. It took decades until the concept was established that neurotransmitters such as glutamate or GABA are the communication molecules in the brain. While myelin was known to Alzheimer as a vision first described by Rudof Virchow (1821–1901) already in 1854, its structure was not resolved until the 1950^th^ with the advent of electron microscopy into neuroscience [38]. At Alzheimer´s time, it was viewed as a liquid crystal [39]. The cell membrane as a structure of a lipid bilayer became only evident with the discovery of the natural synthesis of cholesterol and fatty acids by Konrad Bloch from Cambridge, USA (1912–2000) and Feodor Lynnen from Munich (1911–1979) which earned them the Noble Prize in 1964. Thus, it was visionary from Alzheimer when he stated: ***“I have serious reservations about identifying substances stained in microscopic preparations as substances identified chemically for several reasons: first, my own previous mistake; second, our insufficient knowledge of the chemistry of the brain. Moreover, the large number of different granules and substances which we find in the nervous system far exceeds the number of substances which chemistry has so far found in the central nervous system, and we are dealing with pathologic products, while chemistry has scarcely studied substances generated under pathologic conditions in the nervous system.”****(page 494, 495).*

Also at that time, there was nothing known on the molecular machinery of the metabolic processes in the brain, not even of other tissues. Otto Warburg (1883–1970) from Berlin described the process of anerobic glycolysis in 1923 (Nobel Prize 1931) and it was only in 1929 that Karl Lohmann from Berlin (1898–1978) discovered ATP as an important small molecule for energy storage and developed methods to determine the ATP content of tissues. Warburg´s student Hans Krebs (1900–1981, Nobel Prize 1953) from Cambridge, England discovered the citric cycle, ´Krebs cycle´ or TCA cycle in 1937 as the metabolic pathway to generate energy under aerobic conditions. We know now from studies over the last decades that astrocytes and oligodendrocytes have intense interactions with neurons and their axons [40, 41] leading to the current concept that brain energy rescue is an emerging therapeutic concept for neurodegenerative disorders [42]. Thus, it was another visionary concept formulated by Alzheimer that glial cells are involved in metabolic processes and that understanding these pathways may lead to novel concepts to treat brain diseases: ***“We also have made single observations that the glia does not only participate in decay of degenerating nervous tissue, but also in disturbances of metabolic processes. This needs further studies. This will yield further visions to obtain a better understanding of pathological processes based on (glial) alterations.***

***With the finding of many different substances in the pathologic nervous tissue, one needs to perform an attempt to collaborate with the chemistry of the pathologic brain as already tried by Wlassek and Reich which has a perspective of success. Since that such pathologic substances are found in large amounts in some disease states, it should be possible to extract and isolate them and determine their composition. This would considerably advance our understanding of these diseases.”*** **(540, 541).**

## Summary and conclusions

On the basis of the detailed observations of the glia reaction to CNS injury Alois Alzeimer reached the following conclusion:

***Of large importance is the progress in our general pathologic insight. It becomes now quite evident that the neuroglia has other functions than simply serving as a support structure of the central nervous system. A variety of glial elements are involved in such a task. They clean up, remove degradation material and help to deliver it to the lymphatic system. In certain conditions, the ameboid glia, by serving such functions dominates over the fiber-forming support system. We recognize a magnitude of different glial cell forms and learn to understand their relevance. This allows us to get a better understanding of the pathologic events of the cortex and the features of diseases processes.***

The presence of ameboid microglia and of reactive astrocytes with similar cytoplasmic alterations at sites of tissue damage in the central nervous system has been confirmed in numerous studies in brain development, in brain pathology and in aging (for review see [43–45]). Intense efforts have been invested during the last years to elucidate their potential function by single cell and spatial omics technologies in specific diseases [19, 46–50]. However, recent efforts revealed that the phenotypes are largely shared between different neurodegenerative diseases, aging, and multiple sclerosis [51, 52]. The respective functional annotations revealed a loss of the homeostatic phenotype, phagocytosis, lipid uptake and metabolism, iron sequestration, stress response, cellular activation, antigen presentation, induction of pro-and anti-inflammatory cytokines and complement activation [52, 53]. All of these pathways are in line with a reaction to tissue injury and damage, while the search for pathways responsible for the direct induction of cell and tissue degeneration was disappointing. This is already anticipated by Alzheimer in this manuscript published in 1910, pointing out that the vast majority of ameboid cells in the lesions are reactive cells mainly involved in the clearance of debris and its transport out of the CNS parenchyma. Genuine cytotoxic actions may be restricted to very small subpopulations, in conditions of slow progression of disease and lesion.

The existence of traffic from the CNS parenchyma and from the lesion into the perivascular space has been proven for solutes [54, 55], particulate material and for debris containing phagocytes in subsequent experimental studies [56, 57]. Whether the spaces in the perivascular and meningeal tissue, which contain the debris are genuine lymphatic vessels and whether and how they are connected with the peripheral lymphatic system is controversial up to now [57, 58].

Considering the time when this manuscript was published, the limited knowledge about the nature of the disease processes under investigation and the very limited specificity of the staining techniques it is remarkable what groundbreaking conclusions were reached by Alois Alzheimer through his descriptive, but meticulous analysis of pathological alterations seen in autopsy tissue of the diseased human brain. Even today, more than 100 years later and with the accumulation of fascinating new genetic, biochemical and neurobiological data, we still have only partially revealed the role of glial cells in normal brain function and in the different disease states of the brain. One of the most important deficits currently is to closely relate the different metabolic alterations to the cell changes and active tissue injury in complex human disease states of the central nervous system. The meticulous work by Alois Alzheimer, published in 1910, provides a road map how to integrate new molecular research into the context of disease-specific neuropathology.

## Short biography Alois Alzheimer

**Fig. 5:**
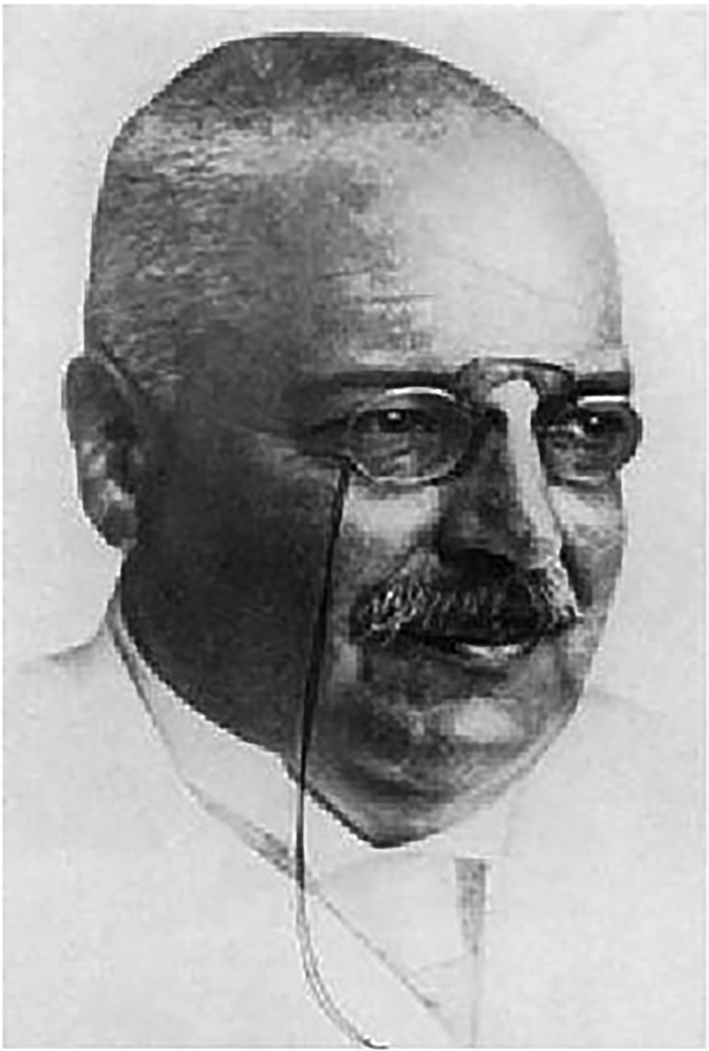
Portrait Alois Alzheimer. From Wikipedia, the picture is in public domain

Alois Alzheimer (Fig. 5) was born in Marktbreit, Germany on June14, 1864. His birthhouse is today a museum and a conference center and at the website of the sponsor, the pharmaceutical company Lilly, you will find information about Alzheimer´s personal and professional life (https://de.lilly.com/alzheimer-haus/en).

He studied medicine in Berlin, Tübingen and Würzburg. From 1888 on, he worked at the Asylum for Lunatics and Epileptics in Frankfurt, Germany, as a neurologist, psychiatrist and researcher together with Franz Nissl. Nissl and Alzheimer edited several volumes of the Histologische und Histopathologische Arbeiten (Histologic and Histopathologic Research) in which his account on the pathologic glia was published. In 1903 he followed Emil Kraepelin, one of the best-known German psychiatrists of the time, to Munich where he received his second degree and was appointed professor in 1908. Kraepelin coined the term Alzheimer’s disease. In 1912, Alzheimer was appointed professor of psychiatry in Breslau, Silesia (present-day Wrocław, Poland) and died there of heart failure on December 19, 1915 at age 51.

## Electronic supplementary material

Below is the link to the electronic supplementary material.

Supplementary Material 1

Supplementary Material 2

## Data Availability

No datasets were generated or analysed during the current study.
