# Supplementary material for: The functional role of glial cells in the pathologic brain as reviewed by Alois Alzheimer in 1910
Source: Mol Neurodegener. 2026 Feb 13;21:19. doi: 10.1186/s13024-026-00933-5 (PMC13005332; doi:10.1186/s13024-026-00933-5)
Supplement: Supplementary file 1 — Supplementary Material 1 [file 13024_2026_933_MOESM1_ESM.docx]

**401**

**Contributions to the knowledge of the pathologic neuroglia and their relationship to degenerative processes in the nervous tissue**

**By Alois Alzheimer**
(With Tables XXVIII–XXXV and 11 Figures in the text)

1. **Aim of the investigation**

When we apply the Nissl stain on ganglion cells and the Weigert glia and myelin stain, methods which we usually use to study the central nervous system, it was not difficult to distinguish between a paralysis, a senile dementia, arteriosclerosis, and some forms of neurolues (1). When studying brain sections from individuals who had so-called simple psychosis, it is impossible to distinguish different, clinically distinct disturbances. It is not that we do not have enough pathological material, but despite all efforts, we cannot find specific features.
Using the Nissl stain, we can find distinct changes in ganglion cells. They are, however, as Nissl himself and others have noted, similar in different diseases and different in some cases of the same disease. Thus, there are many transition forms in multiple combinations reflecting the diseased ganglion cell. One can find typical forms of the pathological cells, but one cannot relate

**402**

those to defined clinical cases. For most pathological changes of the ganglion cells, we do not understand what they mean. It may be a severe change which will not recover or a transient change — in short, we do not know its pathological impact. This insight led to today’s attitude that one no longer pays attention to changes in ganglion cells revealed by the Nissl stain.
Even if a significant portion of ganglion cells degenerates, one can hardly notice it, since they occupy the entire cortical layers and are very abundant and in complex arrangements. We also cannot do better by focusing on the nerve fibers. We know little about the pathological change of fibers, which also easily generate artifacts. A degeneration of fibers is hard to note if it is not massive, since they form a complex mesh and the remaining fibers can move closer together.
Also, what we know about glia using the current methods is not sufficient to obtain diagnostic features related to defined diseases. Weigert stated that a degeneration of nervous elements is replaced by a proliferation of glia and a novel generation of glial fibers. If this were true, one would expect to observe in glial preparations alterations in the arrangement of nervous degenerations in different diseases, like in a negative of a photographic picture. But these expectations were not fulfilled. It is now clearly established that a significant degeneration of nervous elements can occur without a generation of novel glial fibers. In preparations which show, without doubt, a complete representation of the fibrous glia, there are not more or fewer glial fibers compared to normal, even when many nervous elements are damaged or have deteriorated. We will even observe that normal or pathological glial fibers can deteriorate together with nervous structures in some diffuse pathological processes.

**403**

A pathological increase in glial fibers indicates only to a limited extent a deterioration of nervous elements. Where it is present, a degeneration of nervous elements may have happened, but a lack of such an increase is not evidence that nervous elements were not damaged.
Nissl was the first who noted that defined nervous system diseases are correlated to defined alterations in glia. His investigations are not restricted to formation of glial fibers but include progressive changes of plasma and nucleus of glial cells. Unfortunately, his results were never reported in detail; it can be assumed that he may have seen much of what I will show in the following. Yet the images provided by his method — he mainly used the stain related to his name — are so subtle that it was only possible for Nissl’s detailed observation to recognize morphological correlations. The importance of single changes could not be revealed in his preparations.
Moreover, disease-related changes in the mesodermal tissue components of the cortex are not distinct enough to be related to different forms of psychosis. For paralysis, syphilis-related dementia (Hirnlues), and senile dementia, these are important characteristics for differential diagnosis. Moreover, we find in all processes where we assume a degeneration of nervous tissue similar alterations, namely an accumulation of fatty products in cells of the adventitia and the adventitial lymphatic space partially accompanied by a slight excrescence of the cells of the vessel walls.
The methods by which diseases related to paralysis can be distinguished by pathological histology will not help to advance the diagnosis of different forms of psychosis. There is little chance.
With our methods based on pathological histology, we have reached the limits to distinguish different diseases and provide clinical differential diagnosis.

**404**

Exceptions to this may be achieved for less well-known paralysis-related diseases or different forms of cretinism. We regularly have patients who die based on severe cortical disease symptoms or transit into an incurable idiocy, without obtaining any results based on anatomical investigations which may explain such a clinical course. This indicates that we are at the very beginning of the pathologic anatomy of psychosis. We always find pathological changes in those brains, but we find those also in brains of any patient with insanity who is affected only by slight psychic disturbances. What we are therefore substantially missing is the recognition of the pathological value of the different changes.
Is it possible to fill the gaps with other means? One has to assume that the differences in the appearance of distinct brain diseases are based on distinct damages of nervous elements of the cortical layers. This may be based on the features of the pathological process, its arrangements, or progression. Our current technology is insufficient. We do not yet know any method which will display the details of the nervous structure in a sufficient and reliable manner useful for pathological analysis. One has to advance in a looping way.
If it is true — as new investigations indicate — that glia, besides forming glial fibers, also form other detailed structures enwrapping nervous elements in a delicate manner, one can expect changes in these closely adapted glial associations when there are changes or deteriorations in the nervous elements. Nissl’s studies mentioned above indicate that this assumption may be true. One therefore has to focus on displaying the detailed glial structures in a much better way.

**405**

It is furthermore obvious that in all psychotic cases which lead to a dumbing down, one can find a significant increase of fatty matter in the cortex. In some cases, one can find an incredible amount of lipoid substances in the cells of the adventitia and pia. Many cells are loaded with fatty matter or even convert into fatty granular bodies. It is unlikely that the fat in the cells is a degradation of its intrinsic cellular substance. Its mass is too much. It leads to the assumption that it originates from nervous elements and is stored there. It is thus the next task to find the pathways by which these lipoid substances enter the cells. One has therefore also to study the intermediate degradation products which transit between the normal tissue components of the cortex and these final products in the cells of the lymphatic sheath and the pia.
This indicates novel approaches to identify delicate degenerative processes in the cortical layers. This is a similar approach as the Marchi method which analyzes the degeneration of entire fiber bundles based on the degradation products of the myelin. For our purpose, it is however much too crude.
On the search for pathological substances, one can find those abundantly and distinctly. One can find even other notable phenomena. It is noticeable that the two mentioned pathways are often combined since a large portion of these pathological products are embedded in glial cells, partially in a particular form.
It has been often discussed that they do not only play a role as supportive elements but also provide nutritious tasks for the nervous tissue. As much as it is likely, the evidence is still weak and this type of task is still in the darkness. Yet with this option of such a biological function of the glia, it raises the question of whether all these substances we find are degradation products of nervous elements or at least partially retained or altered nutrients for the nervous elements

**406**

or in short, products of a disturbed metabolism of the nervous substance.
The attempt to explain these many findings raises various questions, which are still unresolved.
The current study claims to be not more than an attempt to intrude deeper into the pathological processes of the central nervous system, in particular in the cortex.

1. **Method of Investigation**

It became soon obvious that the usual methods applied for the investigation of the central nervous system are not sufficient to reach the goal as described above. To describe all the approaches in all their details which have been tried in recent years is of little help. Many methods were abandoned since better and simpler approaches were found. Thus, some of the researchers who have worked in the lab of this clinic will now find other staining methods as I have taught them previously. They will be more satisfied as they try those. Often it was desirable to stain the same preparation with different staining methods based on distinct principles or combinations of those. This was partially done to obtain information on the nature of the stained substance based on the staining reactions, partially to determine whether substances which are similarly stained by one method could be differentiated by using other color mixtures and thus are of different types. But also, the latest applied methods are still imperfect. With further experiments they could have been improved. It was, however, sufficient for me if they enabled me to obtain novel insights.
As one will see, these methods are not completely novel but are adaptations based on older ones. I will summarize them and number them and later refer to these numbers. Since the primary goal was to depict the substances which are formed by the degradation of the nervous tissue, ganglion cells, axons, or myelin sheath. In particular, one had to focus on lipoid bodies. The classical fixation for the investigation of the nervous system, namely alcohol or otherwise fixed material with a subsequent treatment with alcohol, ether, chloroform, and xylol for embedding, will extract these substances and make the presentation of these substances impossible. It was therefore obvious not to use substances which extract fat.

**407**

Thus, it was first tried with fresh material. The difficulties which occurred were even more severe than with other fresh tissue. Already due to squeezing there arise so many artifacts that the interpretation of the results was very difficult. The addition of reagents and colors augments swellings or generates unnatural shrinking. The squeezed material changes rapidly during observation; thus, there remains only a short time to study the preparation. Its transition also makes comparisons impossible, which are mandatory for such complex relationships. Thus, I could only convince myself that there are pathological cell inclusions which we could not display with subsequent fixation and staining. Only at the pia could one obtain informative preparations; in particular, the labeling with neutral red can be recommended. It was thus necessary to use fixed material, and first we worked with frozen formaldehyde sections. We still use it today, in particular for the Scharlach staining according to Herxheimer (2), which we apply in the following way:

I. With a mixture of
  absolute alcohol   70
  sodium hydroxide solution  20
  distilled water   10
one fills a test tube to ¾ and adds Scharlach red in excess, seals it, and lets the mixture sit for a few hours, while one frequently shakes it. Then the stain is ready and retains its staining power for 36 hours. For staining, one filters the solution and adds some into a watch glass, which is immediately covered by a second glass to avoid evaporation. Now one adds the slices to the color, covers it, and warms it up carefully two or three times until the upper glass is fogged. Then one lets it sit for ¼ hour, transfers the slices into distilled water, then 10 minutes into Ehrlich hematoxylin (3), into distilled water, into tap water for 1–2 hours. Then one mounts the slices on a glass slide and embeds them in glycerin. With some exercise, one can obtain preparations that are free of Scharlach red deposits. After several weeks, many start to deteriorate by releasing Scharlach red in granules or crystals. Some preparations remain unchanged for months or even years.

II. Using formaldehyde frozen sections, one can display certain substances, which I will term basophil metachromatic, related to substances termed by Reich as protagon (4) or even identical. One should not use old formaldehyde preparations

**408**

since these substances undergo changes with time. One stains the slices in a 1% solution of toluidine blue or cresyl violet, either one hour in the cold or by slight heating and cooling. Then one transfers the slices into distilled water, from there into alcohol, xylol, and embeds them in balsam (5). With some exercise, one can find a time point at which the myelin sheath is sufficiently unstained and the respective substances are sufficiently labeled in fine detail. The preparations deteriorate rapidly. Some of these substances can also be observed in alcohol slices which are treated with conventional toluidine blue stain. One has to take into account, using the latter method, that artificial products are co-stained, such as components of the myelin sheath extracted by the alcohol and released into the tissue.

III. Certain degenerative products associated with certain diseases can be well displayed selectively in formaldehyde frozen sections using a solution of May-Grünwald stain (6) in methanol. One studies the preparations in glycerin, where they, however, deteriorate rapidly. By rapid transfer into water-free acetone and xylol, by a short pretreatment with osmium acid — two drops of the 2% solution in a watch glass with water — the staining will last longer. This staining can also be applied to Flemming (7) slices. Also, those substances change after some time when stored in formaldehyde and lose their stain.
In the following studies, I found that the stain of the glial cell cytoplasm was negatively influenced after long storage in formaldehyde. Therefore, I used material treated with Orth solution. Method II can also be applied here and is best performed according to Reich’s procedure. Yet still, it was not optimal. After many experiments with other fixation solutions, pieces fixed in Weigert’s glial stain were quite useful for the presentation of some protoplasmic structures. They also did not shrink noticeably. We now generally use Weigert’s formula with fluorochrome. Material immediately embedded in Weigert’s stain results in much better images than those embedded first in formaldehyde. Also here we mainly used frozen sections. To avoid damaging the knife with acid, it is necessary to wash small pieces in running water for 2 hours. Longer washing up to 12 hours does not affect the stain. Mainly two methods were used.

IV. One places 10 μm thick frozen sections with glial (Weigert) staining 1. briefly in destilled water, 2. two minutes in water with some drops of pure acedic acid (about one drop for 10 ml), 3. Directly into a strongly diluted solution of Mallory hematoxilin

**409**

10 ml 10% phosphor molybdenum acid
1.75 g hematoxylin
5 g carbolic acid
200 g water.
The stain is only after 6 to 8 weeks sufficiently aged, in a furnace in half of the time. With age, the stain intensity increases. One then needs only 4–5 drops on a watch glass with distilled water. The solution should contain so much stain that it is nontransparent on the glass. A staining solution that is too old results in an unattractive blue color. The slices remain in the hematoxylin for 2 minutes, then are transferred to distilled water (some minutes), increasing concentrations of alcohol, xylol.
The color of the slices should be reddish blue. Blue slices are less favored. The reddish blue staining is secured by pretreatment with acidified water. It gets lost by remaining too long in distilled water and alcohol. — This method results in a brilliant presentation of the cell body of the ameboid cells including the granules of these cells. The glial fibers are partially well visible. In normal cortical preparations, one can observe the delicate protoplasmic arborizations of the glial cells. Furthermore, well illustrated is the content of the perivascular space. In addition, ganglion cells, axons, and vessels are also stained. These preparations are particularly valuable as overview slides. One can rapidly get an overview of the condition of the glia. The method was developed based on the one by Eisath. Eisath’s preparations are sometimes nicer and more selective, but often less so. Moreover, the Eisath method is more cumbersome. The protoplasm of the ameboid cells is much better labeled with this method. The granules and the content of the perivascular space are much better displayed compared to the original method. Severe shrinkage, as sometimes occurs with the Eisath method, is not observed. Table XXVIII shows preparations obtained with this method. They remain well preserved.

V. One transfers the same slices

1. for 2–12 hours into a saturated water solution of phosphor molybdenum acid;
2. briefly washes twice with distilled water;
3. transfers the slices to Mann solution:
    35 ml 1% methylene blue solution
    35 ml 1% eosin solution
    100 ml distilled water;
4. rinses briefly with distilled water until the slices no longer release colored stain;
5. transfers the slices to 96% alcohol (1–2 minutes) until a light blue color occurs;
6. transfers to absolute alcohol and xylol.

**410**

The protoplasm of the ameboid cells is stained lighter or darker blue, certain granules of it (the protoplasm) dark blue, vacuoles reddish, the axons are blue or reddish blue, degenerated ones shining red or black blue, glial fibers light blue, ganglion cells dark blue, connective tissue fibers dark blue, blood cells shining red. The white matter is stained red and becomes almost colorless after a longer time.
This method yields more delicate and more detailed images as compared to method IV. An overstain as with method IV does not happen. The fine, protoplasmic ramifications of the normal glia of the cortex are not visible. Table XXIX shows preparations obtained by this method.
The preparations can be maintained for several months. However, after some time, they fade, in particular the red color.
Furthermore, it was desirable to work with thinner slices as can be obtained with the freezer microtome. This was necessary to display the relationship of the cells to the vessels, which often fall out from the frozen sections and which cannot be resolved in detail in the thick slices.

VI. This method turned out to be the best:

1. Fixation of the material for 24 hours in 10% formaldehyde solution;
2. from there directly for 8 days in Flemming solution;
3. 12–24 hours washing in running water;
4. embedding in paraffin of a 58° Celsius melting point;
5. slicing to 2–3 μm thickness;
6. attachment of the slices with water on the glass slide;
7. deparaffinate and transfer into 96% alcohol;
8. 1 hour in the furnace at 58° Celsius in saturated water solution of acidic fuchsin;
9. washing twice with water until no color appears;
10. insert the glass slide with movement into a solution of saturated alcoholic pikrin acid; solution 30, distilled water 50, 10–20 seconds;
11. carefully washing twice with water;
12. embedding into a saturated water light green solution for 20–50 minutes;
13. rapid rinsing with water, rapid transfer to 96% alcohol, absolute alcohol in xylol.

This method is based on the one by Altmann and was similarly used by Galeotti and Levi and results in beautiful preparations. I prefer the light green over the methyl green since it results in sharper color contrasts. The light green displaces the S-fuchsin from certain tissue parts.

**411**

In the beginning, I did not apply the pikrin acid differentiation. But this has the advantage of yielding more homogeneous staining.
The optimal time for the light green staining must be tested for each material. I mainly stain for 30 minutes. Often, a longer staining yields better preparations. Good preparations show neither a red nor a green staining, but are violet.
The cell bodies of the ameboid glial cells are stained in green, certain granules of the cell body shining red, the lipoid inclusions delicate brown. In the tissue, one finds numerous other red granules, while the protoplasmic parts are green; the glial fibers and the red blood cells are red, the myelin sheath unstained. The elements of the vessel walls also stain very nicely, in particular the connective tissue fibers of the adventitia and certain substances in the perivascular space.
Pieces of the central nervous system which have remained longer in the formaldehyde yield less clear pictures due to the darkening of slabs and granules from osmium. If one wants to continue with that material, one replaces the Flemming solution with a chrome-acetic acid mix. One obtains reasonable preparations if one more carefully applies the pikrin acid and stains only half of the time with light green.
Finally, one can apply other color combinations instead of the S-fuchsin–light green staining with good success. The reported method provided me with images with the best contrast. Table XXX shows preparations stained with that method.

VII. For the depiction of the fuchsinophil granules and the neurosomes (8), one can apply the following method.
One uses method VI until point 7, but then transfers the slices to distilled water.
8. 1 hour in the furnace at 35° Celsius in a water solution saturated with acetic copper;
9. twice wash with distilled water;
10. ½ hour in 10% alcoholic hematoxylin solution (10 ml), distilled water (87 ml), saturated lithium carbonatesolution (3 ml);
11. short wash with water;
Transfer through alcohol to xylol.
From the light basis, different granules are dark blue stained and are very well visible. The protoplasm is slightly yellow-gray or blue-stained, so that the cell borders and the axons are sufficiently visible.
For the visualization of some of the degradation products, a Weigert myelin staining is helpful.

**412**

VIII. One places not-too-old formaldehyde material for 6 days in Weigert’s myelin stain in the furnace, washes it briefly, embeds it in photoxylin (9), cuts the slices, stains with lithium hematoxylin, and differentiates it in borax-ferricyanide potassium solution.
Certain lipoid substances can be displayed best with the following method, which was developed in our lab by Dr. Mooers and Dr. Minkowski and, according to Minkowski, is best performed as follows:

IX. Alcohol slices or those also embedded in photoxylin will be transferred into water,
2. heated up in carbol fuchsin solution until bubbles appear;
3. from the warm solution transferred into distilled water until no stain clouds are visible;
4. in Nissl soap methylene blue for 1 minute stained at room temperature;
5. in aniline oil alcohol differentiated as for Nissl preparations.
The slices show the Nissl image in an acceptable manner and, besides, lipoid material in a shining red staining.

X. For displaying fibrinoid granules, one applies the Weigert stain with a slight modification, which shows the fibers in an acceptable fashion.
The material from the Weigert stain which was not previously embedded in formaldehyde will be rapidly embedded for 3–4 days in photoxylin, then cut, and the slices are then further handled. From the potassium permanganate, the slices are transferred for 10 minutes into chromogen-formic acid-sodium sulfide solution, but I do not add 10 ml sodium sulfide, only 2 ml. Then the slices are rinsed in water, mounted on the glass slide, and stained according to the protocol. One regularly obtains good preparations even without strictly following Weigert’s protocol.
For the display of glycogen, one should use material embedded in absolute alcohol. The staining works best with the protocol according to Best.

1. **On the normal structure of neuroglia**

Following the work by Weigert, which clarified and promoted knowledge on the support system of the central nervous system, new investigations on glia followed which described other glial formations besides the glial fibers. Most contributions were provided by Held. To display the glial structures as clearly and selectively as Weigert displayed the fibers has not yet been achieved.

**413**

Thus, the fine structure of the glia still remains in the dark today.
If we want to discuss the features of glia under pathological conditions, we need a vision of their structure under normal conditions. Since Weigert provided us a basis for the concept of the support system of the central nervous system with his publication “Contributions to the Knowledge of the Human Neuroglia,” we will start from there.
His description of the structure of the nervous support system raised opposition with respect to his assumption that the glial fibers were a true intercellular substance. Indeed, one can easily be convinced that some glial fibers pass through the cell body of the glial cells and that many free fibers are indeed associated with a seam or a hull of a protoplasmic substance, and that most of them are only a reinforcement of a complex protoplasmic cell body. This can be proven by any staining method which labels besides the fibers also the protoplasm. Weigert’s method displays the fibers too selectively, and the relationship to the structures of the protoplasm cannot be recognized. Thus, Weigert’s concept that the glial fibers are an intercellular substance is not correct.
Held had focused on the relationship of glial fibers and glial cell protoplasm and could demonstrate that the glia, besides its fibers, also generates other structures which lend it a feature of a syncytial tissue. Weigert’s description of the glia receives a substantial completion. The delicate protoplasmic processes of the glial cells branch, and the branches of different cells fuse into a net-like formation. These glial nets penetrate the entire central nervous system. The filled nets of Bethe, who even considered them as coagulation products, and the Golgi nets according to Bethe in close association with nervous structures are, according to Held, part of the glial reticulum. It has been emphasized several times that this network is not produced

**414**

from a single cell protoplasm, but from another, different, special substance. As particular syncytial formations of the glia, Held describes the glial border membranes, the membrana neurogliae superficialis and perivascularis. One delimits the surface of the central organ against the pia; the other covers the vessels which penetrate through this organ. They are supposed to be formed by mosaic-like composition of parts of the glial reticulum and the glial fibers, which insert with their ends in the form of glial endfeet into the border membrane.
Nowhere in the normal nervous system can the glia reticulum be presented in such detail, as Held already emphasized. In the deeper cortical layers, it is difficult to recognize. It can best be illustrated in the superficial layers of the cortex. One can easily display a net-type arranged substance using several methods. Through its mesh, the axons penetrate, and in their bars, the glial fibers are embedded. Processes of protoplasmic formations which are arranged around the nuclei are in relation with these nets and are lost in them, and cell borders cannot be recognized. Towards the first ganglion cell layer, the mesh becomes rapidly denser and more delicate. In deeper layers, I could not display it with any method. It is obvious that the most delicate nervous structures are stained together with it. In the white matter, it is better visible. At different regions of the cortex, the structure is different. It can be recognized better at the frontal sulcus as compared to other regions since the mesh is coarser. I can agree with Held that the reticulum of glia is not an ordinary cell protoplasm. Methods which stain the protoplasm intensely result only in a faint, delicate label. Depending on the methods of fixation, it appears more granular or as a more delicate, skin-like extension. The assumption of Held that the filled nets of Bethe and Golgi correspond to the glial reticulum is substantiated by several observations. One can see that the Golgi nets transit into the wider mesh of the filled net,

**415**

embedding even axons and forming nodes of Ranvier, as Held has reported in detail.
Thus, the Bethe method is most efficient to selectively display the glial reticulum. I should remark, however, that it failed on pathological material. Either the reticulum was labeled as in the normal condition, or it could not be displayed at all. Due to the variability of that method, one cannot make any conclusion. Thus, it was not possible to obtain results on the properties of the syncytial glia under pathological conditions.
It is much easier to visualize the glial reticulum with some other methods, such as in strongly atrophic cortical regions at areas which are degenerated by neighboring lesions. Table XXXV, Fig. 6 shows such a glial reticulum at the striatum in a case of progressive chorea (10). Oppenheim recently stained this reticulum with a simple, not selective method and described the changes in lesions of multiple sclerosis. I will not further elude on the net-type glial structures since they have little relevance for the alterations which I will mainly describe here. They seem to be repaired after the course of the acute process in a form coarser and deviating from the normal form. Its presentation can be valuable for recognizing small lesions. It may be due to the fact that the reticular glial formation serves as an embedding and support structure and therefore does not contribute to the degradation processes.
While there are few doubts on the existence of the reticular formation of glia, one has to oppose the opinion that all or the majority of the glial nuclei which we find in the central nervous system, particularly in cortical regions, are protoplasm accumulations

**416**

of the general reticulum and that all glial elements have lost their individuality in favor of the net.
Already the Golgi technique has provided us glial cells with numerous and long processes. In slices of spinal cord where glial cells are well placed, we can see glial cells, closely together, which project a large number of very thin and long processes. Together they form a dense felt of crossing fibers. On many cells, we observe processes projecting to blood vessels. It is most likely that each cell has connections to the vessel system or the perivascular border membrane. One may fail to recognize all these relations due to the limits of the slice and the extension of the area occupied by one cell. We know from studies of neurons that the Golgi technique does not label all cells. One can object that a complete reticulum formed by the processes of the glial cells cannot be recognized due to the varying conditions of impregnation. But the images do not look like that. We never observe that fibers which cross, fuse. Why are those many crossing fibers present, if the reticulum is the essential?
Even if we consider this, the Golgi images provide evidence that there are highly complex glial cells embedded in the reticulum.
This is supported by other stains such as the Eisath method and the modification described by me. Since these are simple stains, this disregards the concern raised against the Golgi method. Even with these stains, it is possible to label complex glial cells, particularly in cortical layers (Table XXVIII, Fig. 1a, b).

**417**

One is never certain to have identified the end of a branching. But a transition of one process of one cell into one of another or into a net can also not be found. On particularly good preparations, one can follow the processes as far as in Golgi preparations; with this method, one cannot find such high branching and generally not as long processes, since one has to use much thinner slices. These images do not definitively exclude that the processes of the glial cells, at the point where they become invisible, enter a network of an altered glia substance. They show, however, that highly branched cells are within the reticulum. Also, at these pure protoplasmic formations, we can observe the multiple interactions with the vessels as described above.


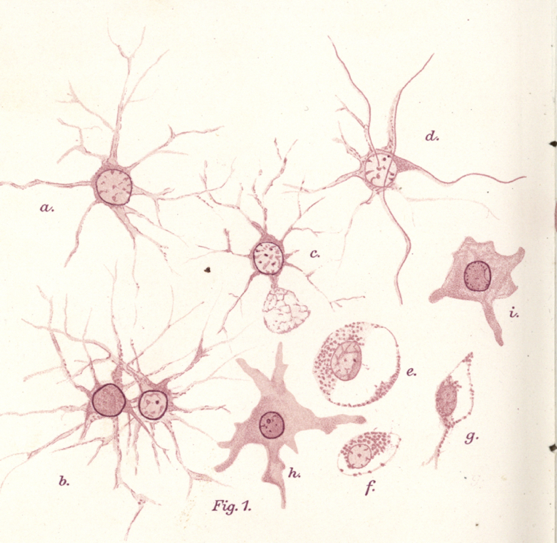
*Fig. 1 a, b. Normal glial cells with protoplasmic branches from the cortex of a 34-year-old man who died due to an accident. c glial cell from the cortex of a 68-year-old man diseased with arteriosclerosis. d glial cell from the deeper cortical layers of a 63 years old woman diseased with depression. e, f, g glial cells from the white matter of a 34-year-old man died due to an accident. h, I glial cells from the cortex of juvenile epileptic demented due to multiple seizures.*

In the same images, we found glial elements which are quite distinct (Table XXVIII, Fig. 1 e, f, g). Eisath has already described and illustrated them. One can recognize them also in alcohol slices after Nissl staining. Around a glial nucleus, we find an accumulation of granular substance, often only on one side of the nucleus. In a larger bow, separated by an empty space, there is a round or oval line formed out of granules circumventing the nucleus, sometimes made out of only one row of granules. Attached to this are often irregular, small, granular tatters which are sometimes connected with a bridge to the granular protoplasm around the nucleus. Sometimes one has the impression that there are thin threads projecting outward and getting lost in the surroundings. They are so subtle that it may also be an illusion.
One can hardly imagine that these elements have such large gaps between the nucleus and peripheral cytoplasm under vital conditions. These images resemble images of ganglion cells in the upper layers of the cortex obtained after alcohol fixation and Nissl stain. Nissl assumed that due to the shrinkage of the tissue, the cell´s protoplasm was ruptured.

**418**

On those glial cells, one does not observe such a widely branched cell body as in those described first. Maybe these delicate processes, which one sometimes believes to see from the edge of the cells to the periphery, are connections to the unlabeled reticulum, and the nuclei are the basic nuclei of the syncytium. It may be that the multiple connections with the surrounding tissue cause the ruptures. With the Bethe method, we see the glia nuclei positioned freely in the reticulum.
So far, these are only assumptions which cannot be verified. It is certain that in the normal cerebral cortex, two types of glial cells can be distinguished: one with a widely branched protoplasmic cell body which can form fibers, and those which can only be inadequately displayed with our staining and fixation methods. On those, we can recognize only a small, roundish cell body of granular composition, often torn apart from the nucleus leaving a gap. The relationship between these two forms and between the glial reticulum and their biological relevance is not yet resolved.
May now all glial cells belong to the reticulum or some have remained cellular individuals — with degeneration of the nervous system, many glial elements are quite autonomous, acquire peculiar forms, assimilate degradation products, and even disassemble without generating glial fibers. If they had originally belonged to a syncytial network, they have now detached from it.
Of elementary importance for the understanding of the degenerative processes and for the function assigned to glial cells is the issue of the glial border membranes, which are closely connected to the lymphatic space in the central nervous system. Held has already viewed this border membrane as a filter membrane for the fluid movements towards and from the nervous parenchyma. We have already, at the beginning of our observations, tried to find out by which pathway

**419**

the massive accumulation of lipoid substances occurs in the pathologic nervous tissue. We find these substances in the cells of the adventitia and assume that they originate from the nervous tissue and raise the question by which pathway they end up at the deposit site and how they pass the filter membrane.
The issue of the glial border membrane is not yet resolved. With respect to the membrana superficialis, I fully support Held’s report, based on my own preparations. Also, at the larger vessels of the central nervous system, one can often clearly observe a border membrane, so that there is no doubt of its existence. If it exists clearly at some areas of the vessel tube, it is most likely that it is present in general.
More difficult is the issue of deciding whether there is a lymphatic space between membrana gliae and the adventitia. Nissl denied it and considered the perivascular space as a space due to shrinkage. He considers the adventitia as the border between mesodermal and ectodermal tissue. Held considered the glial membrane as the border. Earlier, he had considered together with His that there is a perivascular lymphatic space between the border membrane and adventitia. Also, Schröder describes an outer and inner lymphatic space and notes that both are connected at the smaller vessels by a wide gap. Recently, Held considers a tight connection between the glial border membrane and adventitia, if I understand him correctly.
Indeed, we observe in many preparations a close relationship between glia and adventitia, and it can be hardly explained as an artificial adhesion. Using the acid-fuchsin–light-green method, one can label glial fibers in red, the connective tissue in green. In preparations of paralytic cortices, one can occasionally observe that glial fibers had grown into the connective tissue fibers and thus it is unlikely that there is a lymphatic space between them. Moreover, one often observes, in quite distinct pathological processes, that young, protoplasm-rich glial cells closely enwrap capillaries and seem soldered with the adventitia. A border membrane cannot be recognized.
One can argue against such observations that they represent defined pathological cases.

**420**

At least they indicate that one has to clarify the question of whether a perivascular lymphatic space normally exists. Even if one considers a membrana limitans perivascularis as being established, there is the possibility that it is directly attached to the adventitia and that there is no space in between. Then the adventitia and the membrana perivascularis would represent the border membrane.
I agree with the view of Nissl that mesodermal infiltration cells can cross the adventitia only under special conditions. In paralysis, where the adventitial lymphatic space is filled with blood elements, they are hardly found outside. This speaks against the view that a perivascular lymphatic space is in connection with the Virchow-Robin space via large gaps, as Schröder assumes.
It is generally assumed that perivascular tissue gaps between the vessels and the nervous system and remnants of membrana gliae represent shrinking artifacts. One can often observe that a glial fiber projecting towards a vessel passes through this empty space. It attaches at the adventitia or to remnants of the membrana perivascularis or is lost in endfoot-type enlargements at a membrane that seems to be in this tissue gap. This membrane could be considered as membrana limitans, which had enwrapped a vessel and has been ripped apart from the nervous tissue due to subsequent tissue shrinkage while the vessel contracted in the opposite direction.
With respect to the normal relationship of the lymphatic spaces and the perivascular glial border membrane, it is still unresolved whether perivascular lymphatic spaces exist. We will, in the following, provide evidence that under pathological conditions perivascular spaces can be found which are not due to shrinking processes and that the membrana perivascularis can disintegrate and that in these perivascular spaces a substantial part of

**421**

the degradation processes of the nervous system occurs. Like the vessel border membrane, the surface membrane can also disintegrate. These perivascular spaces belong to the nervous tissue since we find glial cells there and no mesodermal elements. This speaks against the assumption that they represent lymphatic spaces.

1. **The pathologic changes of neuroglia during severe acute disease conditions of the central nervous system.**

***A. The ameboid glial cell and its different forms.***

Already in my publication “Histologische Untersuchungen zur Differentialdiagnose der progressive Paralyse” I have described and illustrated glial cells (Table VIII, Fig. 8, 14, 16, 17, 18) which show an enlarged protoplasmic cell body and disintegrate without producing glial fibers. I have later termed these cells “ameboid glial cells,” since they deviate from the so far known forms of glial morphologies and since they look similar to an ameba. Eisath found these types of cells in large numbers in an acute state of dementia praecox (11). Studies on different material have revealed that these are a common widespread event in acute disease states of the nervous system. I would like to mention different descriptions of neurophagic processes. The specific state of their appearance, their distinct morphologic appearance, their obviously peculiar biological importance, which is distinct from other glial forms, in particular the ones forming fibers, has not been sufficiently described. This also includes their important role which they have for the acute disease state of the nervous system, in particular acute psychiatric disorders. They help us to better understand the anatomical basics. In part, this is due to the issue that

**422**

the current methods for the study of glial cells do not yield a clear picture; not the Weigert glia method, since it displays only the fibers; not the Nissl method, which illustrates the coarse protoplasmic glial structures but stains surprisingly little of the cell bodies of the ameboid glial cells.
Besides the general typical forms which we find predominantly in white matter, there are, predominantly in grey matter, multiple forms of distinct appearance. They often do not show any similarity to an ameba. These multiple intermediate stages which can be found and the occurrence of defined granules, which are common in these deviating forms with the ameboid cells, hint at a common status and show us that they are defined less by their morphology and more by their biological features.
All observations indicate that the ameboid glial cell is only a transient form. Only on rare occasions do they develop into more deviating, permanent forms. Apart from that and based on their rapid life cycle, one can find all developmental stages in the same section.
In concert with the appearance of the ameboid glial cells, there is a widespread disappearance of the classic glial elements. Before we focus on the ameboid cells, we should give this fact some attention.
Table XXXIII, Fig. 1, 2, and 3 show those regressively altered glial cells in the same slice with a simultaneous production of very young ameboid cells. The cells in Fig. 1 are from the white matter of the spinal cord of a case of meningitis due to tuberculosis; Fig. 2 and 3 from the cortex and white matter of septic delirium. In Fig. 1, we can observe the multiple changes in the nucleus, which should be interpreted as regressive, such as homogenization of the nucleus and the cytoplasm (a, b, c), clumpy condensation of the nuclear chromatin (e, g, i, k), disintegration of the nucleus into single spheres or clumps (f, h, n, o, r), apparent faint staining of the nucleus (p, q).


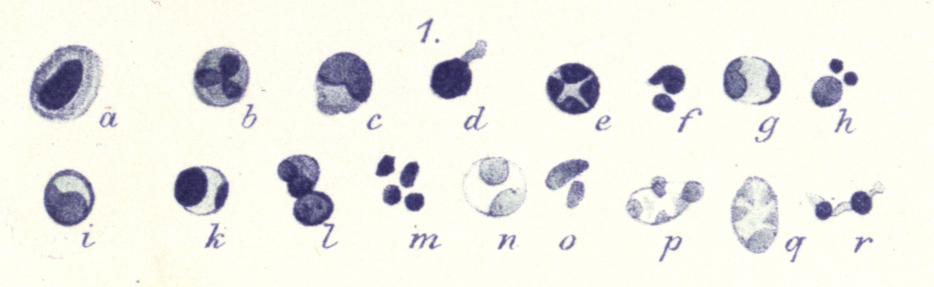


*Table XXXIII, Fig. 1. Different forms of degenerative altered glial nuclei from the white matter of the spinal cord of a case of an acute meningomyelitis due to tuberculosis.*


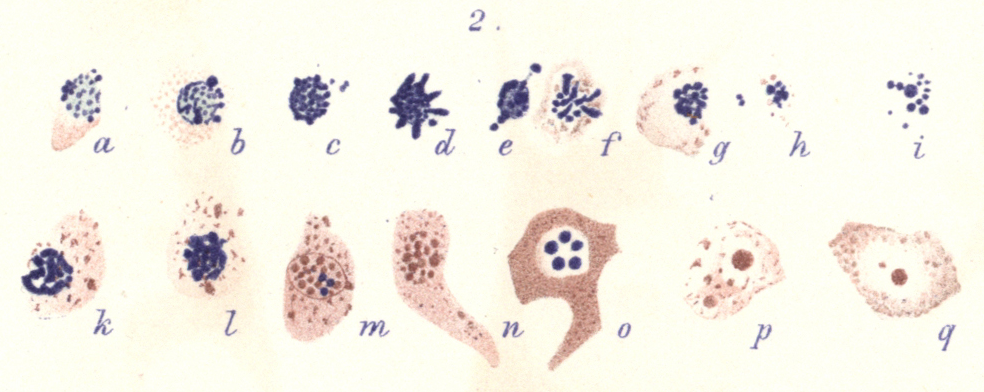


*Table XXXIII, Fig. 2. Different forms of degenerative altered glial cells from the cortex and the white matter from different cases of delirium after sepsis. In the cytoplasm of the cells are in many cases basophil metachromatic granules and substances*.


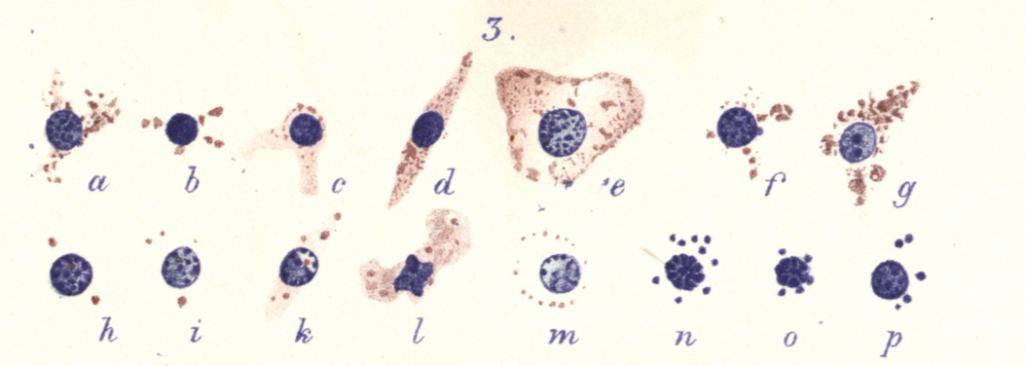


*Table XXXIII, Fig. 3. Different forms of degenerative altered glial cells from the cortex and the white matter of different cases of delirium after sepsis. In the cytoplasm of the cells are many basophil metachromatic granules and substances*.

**423**

In those preparations, one observes nuclei or nuclear products which were very weakly stained.
In Fig. 2, we observe different nuclear alterations. Part of the nuclear chromatin protrudes from the nucleus, gets diluted after a complete loss of the nuclear membrane, and loses its stain. In other forms, we see that the nuclear content condenses and gets distinct from the well-recognized nuclear membrane, similar to what was observed by Nissl at the nuclei during severe ganglion cell disease (m). Finally, single spheres originating from the chromatin label metachromatic and finally lose their label (m, n, p, q). This alteration of the nucleus reminds us of the diseased ganglion cell nuclei in the hippocampus of rabies in rabbits, which Achúcarro (12) describes in the first chapter of the third volume of this series.
Fig. 3 illustrates (in a–g) peculiar slabby and granular, metachromatically stained substances, which we often observe in the cytoplasm of these degenerating cells. n and o of this figure show the mulberry shape of these perishing glial nuclei. We will come back to that when discussing the nuclear changes of the ameboid cells. One also finds it in these disintegrating glial cells, which had never been ameboid cells. The granules around the nucleus can be considered as blast parts of the nuclear chromatin.
Besides the appearance of pyknosis (13), karyohexis, and karyolysis, which can be widespread or observed only occasionally, we find, besides the young ameboid cells, more or less karyokinesis in the glial cells, which is rare or not at all found at the peak of ameboid glia formation. In the brain of cases that died of status epilepticus, one can sometimes find disintegrating nuclei and a lot of karyokinesis and only few, if any, ameboid glial cells; in other instances, many glial nuclear divisions and many young ameboid cells; and sometimes many large ameboid glial cells and not a single nuclear division.
The ameboid glial cells of white and grey matter are distinct, as mentioned before, in significant aspects. We will start with the description of ameboid cells

**424**

in the white matter since they have more common, massive forms and are easier to study due to the clearer conditions. One can observe a clearly defined, small cell body around the nucleus. It hardly stains with basic aniline colors, but intensely with method IV, V, VI. The nucleus is small and and rich in chromatin. With increasing size of the cell body, the nucleus does not increase significantly. Thus, the small size of the nucleus is a significant feature of the ameboid glial cells. In younger, smaller cells there are often convex border lines (Table XXX, Fig. 3 g–k) (14) ; older ones show more or less many concave sections (Table XXX, Fig. 3 b, c).


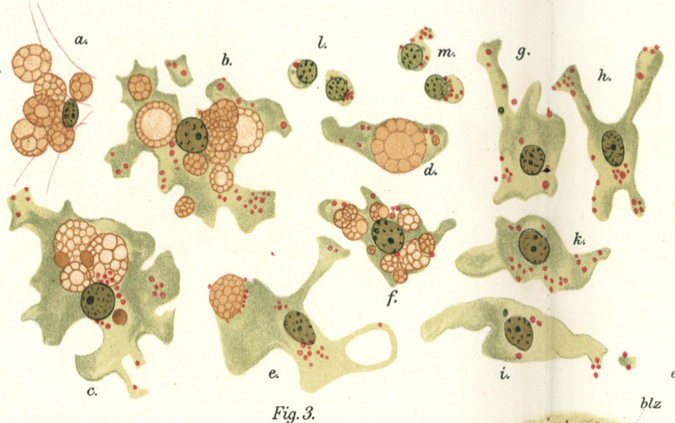
*Table XXX, Fig. 3. Ameboid glial cells from the white matter of different cases of status epilepticus and peculiar psychosis of the degenerated age (15). l, m smallest forms of ameboid glial cells from the cortex of a case of delirium after infection. g, h, I, k younger forms; in g and i each with a light green granule, otherwise only fuchsinophil granules. b, c, d, e, f older forms with large and smaller lipoid cysts. a glial fiber forming cell without clear cytoplasm filled with lipoid cysts.*

In those are the white matter sheaths fitted in (Table XXVIII, Fig. 6, 7). The cells flow around the neighboring white matter sheaths, so that they are completely enwrapped in cross sections and embedded like with a coat in longitudinal sections. As they increase in size, they become more complexly formed structures. They have relationships not only with one, but with several neighboring white matter sheaths.


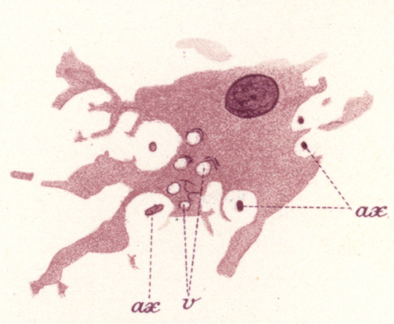


*Table XXVIII, Fig. 6. Larger ameboid glial cell with vacuoles (v), surrounding myelin sheath. Status epilepticus.*

*
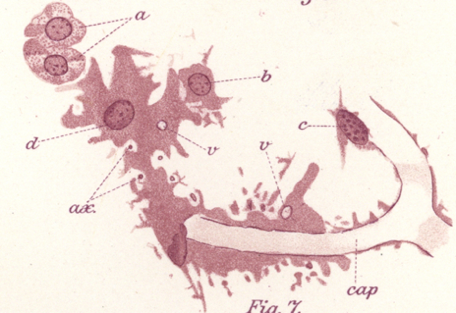
Table XXVIII, Fig. 7. a. small glial cells potentially transition forms from round glial cells with cytoplasmic granules to ameboid cells. b and c small ameboid cells potentially transition forms of glial cells with cytoplasmic processes to ameboid cells. d large ameboid cell with two vacuoles (v) forming a wristband around a capillary and surrounding many myelinated fibers. ´fear psychosis´.*

In thin, consecutive serial slices, one can observe single cells with varying border lines and differently formed processes. In thicker slices, slight changes in the focal plane yield different forms while they extend lobe-type processes in all directions, winding in different ways through nervous structures. It is almost impossible to document the complex structures of these large ameboid glial cells in drawings or photographs. In most cases, they are abundant around vessels. They often are next to each other in multiple layers, young between old, and form coats around vessels. One often observes that a cell projects a broad process toward a capillary and enwraps it like a collar (Table XXX, Fig. 7). Usually, the processes of the ameboid cells broaden significantly at the vessel wall.


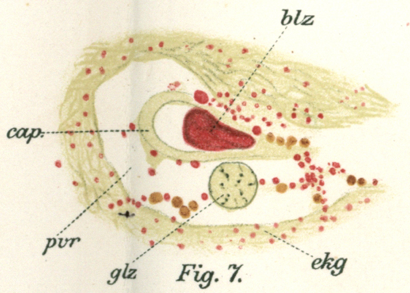


*Table XXX, Fig. 7. Capillary from the cortex of a case of epileptic delirium. In the perivascular space resides a glial nucleus, from which rows of granules emerge formed by lipoid and fuchsinophil granules. They continue to the ectodermal tissue on one side and to the vessel wall on the other.*

In young elements, the cell body appears homogeneous, without granules, quite uniformly and intensely stained.

**425**

In the following course of maturation, several changes within it can be noted. To study these, one has to use different methods. In preparations stained with Mallory hematoxylin, one can observe roundish vacuoles in the large cells (Table XXVIII, Fig. 6 and 7).


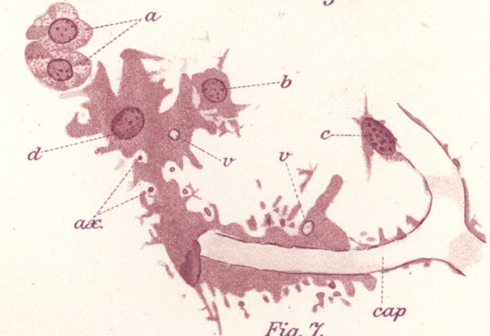

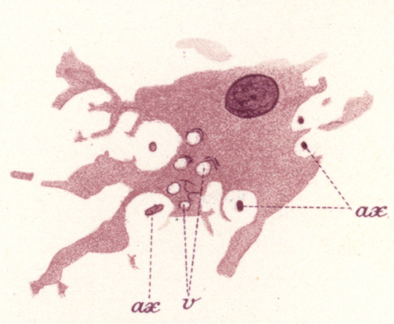


*Table XXVIII, Fig. 6. Larger ameboid glial cell with vacuoles (v), surrounding myelin sheath. Status epilepticus.*

*Table XXVIII, Fig. 7. a, small glial cells potentially transition forms from round glial cells with cytoplasmic granules to ameboid cells. b and c, small ameboid cells potentially transition forms of glial cells with cytoplasmic processes to ameboid cells. d, large ameboid cell with two vacuoles (v) forming a wristband around a capillary and surrounding many myelinated fibers. ´fear psychosis´.*

They are first small, but can acquire a size of multiple nuclear diameters. With close inspection, one can observe that their content is slightly yellowish. In Mann’s solution, the vacuoles are often delicately reddish stained against the blue-labeled protoplasm. With increasing size of the cells, the number of vacuoles increases until the entire cell body is filled with them, one next to the other, leaving only small cytoplasmic bridges. These vacuoles can be followed even better in preparations stained with acidic fuchsin light green (16) after fixation in Flemming’s solution. One can observe, within the evenly light-green stained cell body, red and dark-green labeled granules (fuchsinophil and light-green granules, Table XXX, Fig. 3 g, h, i, k). The light-green granules appear sparser and can be completely absent in some cells. Only rarely does one observe them in larger numbers. Also, the fuchsinophil granules are in the beginning singular but become more numerous and are scattered throughout the entire cell body, often accumulating at some locations. They are always round and typically of the same size. Their considerable size distinguishes them from other fuchsinophil granules. The granules of the ganglion cells (neurons) are smaller, condensed, of smaller size, and partially not round. Only in pathologic ganglion cells do we observe such large fuchsinophil granules.
Obviously, only at later stages do substances appear which can be labeled brown with osmium. These fatty substances are rarely found in the form of round granules. They rather form large clumps of solid nature and generally irregular form or as extraordinary delicate formations which can reach double or triple the size of the nucleus (Table XXX, Fig. 3 a–e). It cannot be doubted that the latter formations correspond to the vacuoles which we observe in cells labeled with Mallory hematoxylin or Mann’s stain.

**426**

These are not vacuoles, but lipoid cysts. After this treatment, they appear circular or oval and composed of many small cysts which are also round. Using high magnification, one can observe that their walls are formed by extraordinarily small, tanned granules. Sometimes one observes a large central cyst surrounded by numerous small ones which together form a large sphere. The content appears lightly yellow-brown. These cysts can also be found in glial cells which have formed fibers, and they seem almost the only thing left from the cell body of the ameboid cell (Table XXX, Fig. 3a).
One can hardly assume that these lipoid cysts in our acidic-fuchsin-light-green preparations are normally present in the form seen here. Against this is the observation that in formaldehyde slices after Herxheimer staining, one can find solid red-labeled spheres around the glia nuclei belonging to ameboid cells. The recognition of the ameboid glial cells in these preparations is not easy. We have to assume that the form of these cysts is determined by our fixation and embedding methods. It has to be emphasized that the accumulated lipoid substances, as often observed in ganglion cells, the cells of the vessel wall, and the glial-type and mesodermal granule cells, are not found in this form. Also, the fatty products embedded in the large fiber-forming glial cells are usually present in the form of solid granules or drops. Only in the coarse, fatty degeneration of ganglion cells, as discussed later, do we find the lipoid substances in a similar form as small cysts. This indicates that we are facing a particular lipoid substance. This is supported by the observation that the content of these lipid drops is labeled distinctly red with Mann’s stain, while the lipid granules of the ganglion cells and the cells of the vessel wall are muddy green.
While more and more of those cysts accumulate in the cell body of the large ameboid cells of the white matter,

**427**

the cytoplasm becomes sparse and less well stained. Finally, one gets the impression that the cysts melt away and the whole cell disintegrates, while also the nucleus undergoes regressive alterations.
Besides this form of regressive alteration of the ameboid glial cells, one frequently observes a second kind which even more rapidly results in their disintegration. The cell body of all young ameboid cells is homogeneous and intensely stained as previously mentioned. In slices stained with Mallory hematoxylin or Mann’s stain, there often occurs a granulation of the ameboid glial cells. The commonly dimly labeled cytoplasm appears as if pruinose, with dark violet or dark blue stained granules (Table XXVIII, Fig. 8c, 9 and 10; Table XXIX, Fig. 17).


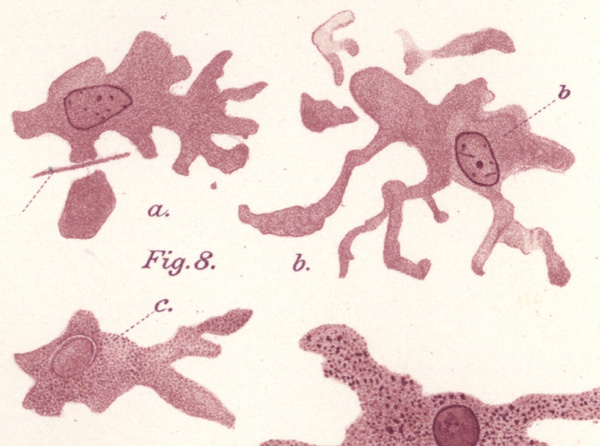


*Table XXVIII, Fig. 8 a, b, c. Large ameboid glial cells from the cortex of a very rapidly progressing paralysis. In c detachment of the nuclear membrane from the nuclear content. Dust-like, fine methylene blue granules in the cytoplasm. Progressive paralysis.*


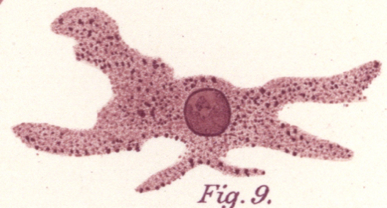


*Table XXVIII, Fig. 9. Large ameboid glial cell with methyl blue granules. ´fear psychosis´.*


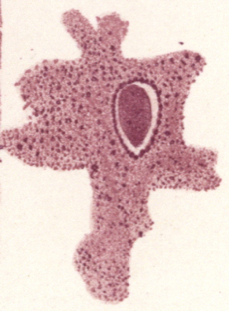


*Table XXVIII, Fig. 10 Large ameboid glial cell with nuclear degradation and methylene blue granules. Status epilepticus.*


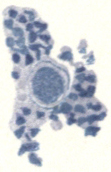
For simplicity, I will term them methylene blue granules, despite them being more apparent when applying Mallory hematoxylin. These methylene blue granules are sometimes very delicate; in other cells, they are significantly larger, yet within individual cells, they are of similar size. The formation of the granules coincides with a loosening of the cytoplasm structure. Using acidic-fuchsin-light-green stain, in which the methylene blue granules are not labeled, the cell body is hardly labeled light green and has obtained a foamy or granular appearance. At the same time, severe changes of the nucleus indicate that the cells decay. The nuclei stain homogeneously or faintly, and one can observe that the contents of the nucleus condense and retract from the nuclear membrane (Table XXVIII, Fig. 8c and 10).

*Table XXIX, Fig. 17. Different forms and decay stages of ameboid glial cells at a progressive chorea.*

Often, glial cells in the white matter change which do not have the characteristics of the ameboid glial cells but have long, slender processes and hardly a cell body. It appears as if the processes disintegrate into granules, which stain like the methylene blue granules of true ameboid cells with methylene blue and even more intensely with Mallory hematoxylin (Table XXVIII, Fig. 11, 12, 13).


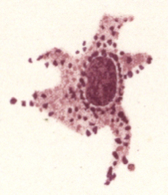

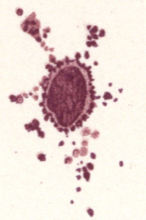

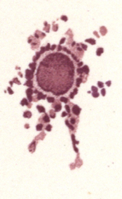


*Table XXVIII, Fig. 11. Glial cell with methyl blue granules in the arrangement of protoplasmic branching. Final delirium at phthisis.*

*Table XXVIII, Fig. 12 and 13. The same cells from the white mater of a suddenly deceased due to catatonia*.

Also here, we observe that the largest number of cells which obtain such methylene blue granules are around the vessels. Yet, in the entire white matter, these disintegrating cells are dispersed.

**428**

Part of them seems to have lost their label. In any case, a large number of ameboid cells decay with the formation of methylene blue granules.
More heterogeneous, complex, and thus much more difficult to understand are the glial alterations in the grey matter which coincide with the appearance of ameboid glial cells in the white matter. Only in rare cases do we observe ameboid cells in the grey matter that correspond to those in the white matter. Among diffuse diseases of the cortex, I have observed this only in paralysis patients who died after very strong and multiple seizures and in one case of progressive Chorea Huntington. Table XXVIII, Fig. 8a, b shows ameboid glial cells from the cortex of a fulminant (17) paralysis.


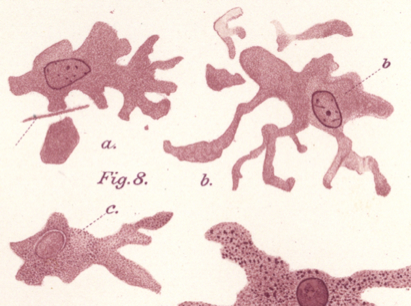
*Table XXVIII, Fig. 8 a, b, c. large ameboid glial cells from the cortex of a very rapidly progressing paralysis. In c detachment of the nuclear membrane from the nuclear content. Dust-like fine methylene blue granules in the cytoplasm. Progressive paralysis.*

These cells often have longer, smaller, and delicate processes, while their other features are not different compared to those in white matter. While the protoplasmic processes of normal glial cells become thinner with increasing distance from the cell body, in these, the processes often terminate in club-shaped thickenings. Due to the wide distribution and large number of long processes, one often observes a cut-off process in the slice preparation that belongs to the same cell.
A further deviation in that direction is represented by cells shown in Table XXIX, Fig. 3, 15, and 16 from a case of progressive Chorea Huntington.


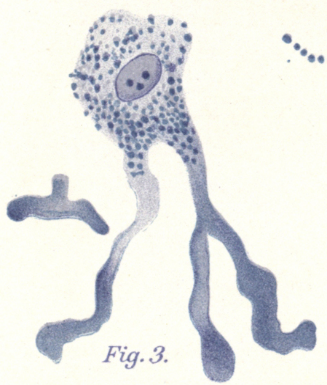

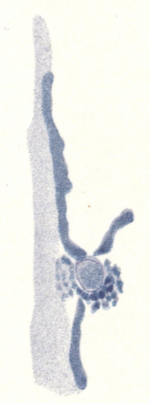

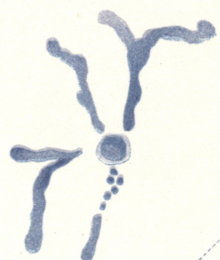


*Table XXIX, Fig. 3. Glial cells in the cortex of the frontal sulci from a case of progressive chorea. The cell body is dissolved in multiple, coarse methyl blue granules, the band-like processes show a uniform dark stained cytoplasm.*

*Table XXIX, Fig. 15, 16. Different forms and decay stages of ameboid glial cells at a progressive chorea.*

The processes, which show the features of the processes of ameboid glial cells, are longer and thinner, while the cell body is hardly formed. They often follow the processes of ganglion cells, as shown in Table XXIX, Fig. 16, where the glial cell projects a process up and down which aligns with the main dendrite of the ganglion cell.

In these cells, we observe very peculiar regressive alterations, which I will refer to later. They are illustrated in Table XXIX, Fig. 4, 5, 8, and 15.


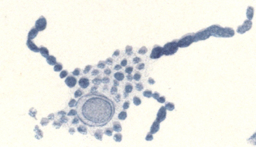

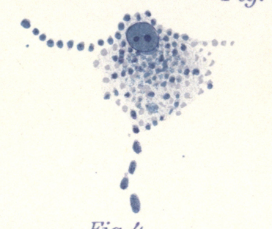

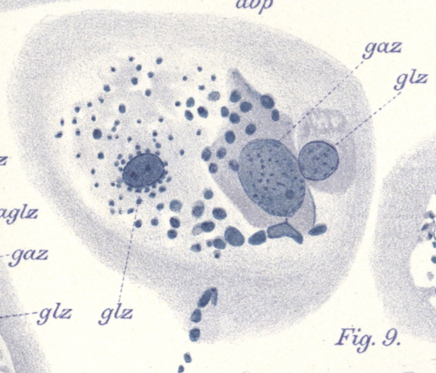

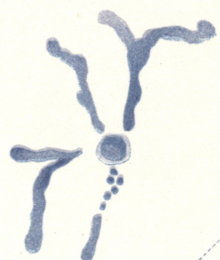


*Table XXIX, Fig. 4, 5, 15. Similar cells of the same case (progressive chorea), frontal, central sulcus, with rosary-like processes, partly disintegrated in single pieces. Degeneration at the nucleus.*

*Table XXIX, Fig. 8. Ganglion cells surrounded by a large perivascular space in which degradation products of an ameboid glial cells are positioned. The same case (progressive chorea).*

The long process often has a rosary-like (bead-like) form and later disintegrates

**429**

into single segments, while the entire cell body disintegrates into single slabs and the nucleus shows the same regressive changes as observed in ameboid cells of the white matter. We find ganglion cells which themselves show degenerative features and are surrounded by lighter and darker stained slabs which originate from the processes of such ameboid glial cells (Table XXIX, Fig. 8). Moreover, one finds an accumulation of those degradation products, in particular around vessels.

A more abundant type of the ameboid glial cells as the one described is a form which even further deviates from the forms in the white matter. It is yet similar in its progression. We see such forms depicted on Table XXVIII, Fig. 2 and 3 where they enwrap ganglion cells or are attached to vessels.


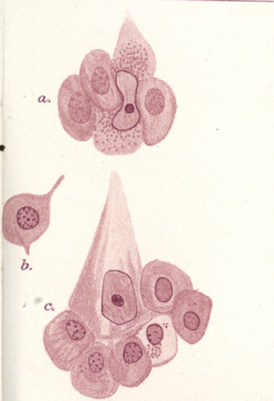

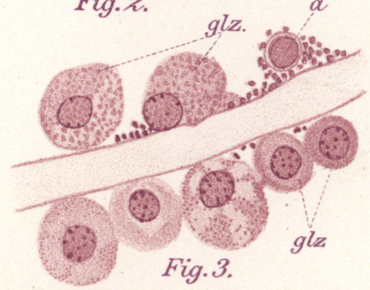


*Table XXVIII, Fig. 2. Ameboid glial cells from the cortex of a case of severe delirium after infection. a, the glial cells deform the cell body and nucleus of the ganglion cell. b, an ameboid glial cell with two stump-like processes (maybe the rest of originally longer processes). c, a ganglion cell surrounded by six ameboid cells.*

*Table XXVIII, Fig. 3. A capillary from the cortex surrounded by younger and older ameboid cells; in the older cell beginning degradation of the cytoplasm, at a cell with methylene blue granules.*

The cell body is round or oval, on the smaller younger elements homogenous and intensely stained like the cell body of younger ameboid glial cells of white matter. The nucleus in the younger forms is often in the center and in older ones out of the central position.

Since this cell form is most abundant in the cortex, we will study them in more depth. One can display them reasonably well in slices fixed in alcohol and stained with toluidine blue if the differentiation has not progressed too far, also with methyl green pyronin staining and even better with a Giemsa solution if one stains for 12 hours and moderately de-stains with Anilin oil-alcohol. In particular with the last staining the cytoplasm of the cells acquires a pale blue color which sets itself well apart from the tissue. With these staining one can document regressive changes in the cytoplasm and the nucleus. In the cytoplasm there appear irregular granules and crumbs of dark staining, often with a yellowish mixed tone. (Table XXXIII, Fig. 6, 8, 9). In the nucleus one can observe light patches (Fig. 6, below). It acquires a blackberry-form, since the nuclear membrane disappears and coarse chromatin slabs extrude over the border of the nucleus (a cell in Fig. 11, the lowest cell in Fig. 9). Furthermore, single chromatin parts move out of the nucleus (Fig. 8 and 9).

**430**

And finally, the entire nucleus dissolves into single slabs which are dispersed in the cytoplasm (Fig. 9, middle cell).

Particularly notable are the relations of the ameboid glial cells to the ganglion cells and to the vessels. One can observe that they accumulate around the ganglion cells (Table XXVIII, Fig. 2a, c), whereas they often press themselves into the ganglion cells, so that they are positioned in an indentation of the ganglion cell body and press the nucleus together so that it acquires a form of a biscuit or kidney (Table XXVIII, Fig. 2a, Table XXXIII, Fig. 8, 9, 10).


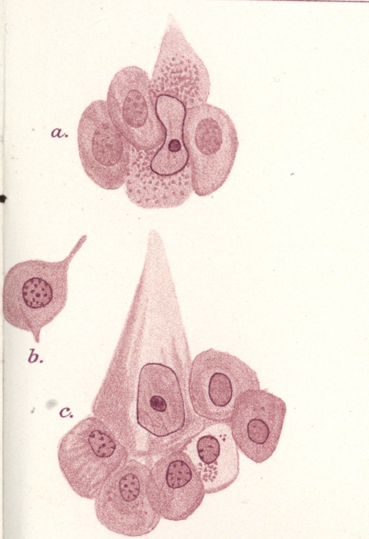

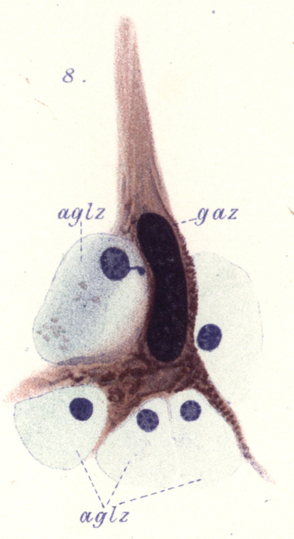

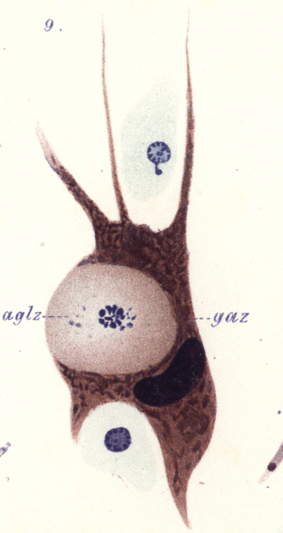

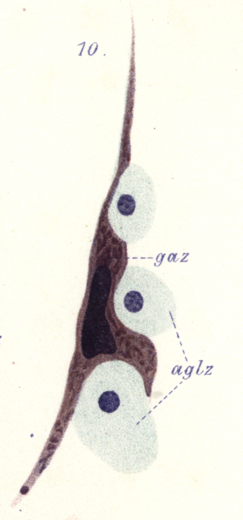


*Table XXVIII, Fig. 2. Ameboid glial cells from the cortex of a case of severe delirium after infection. a, the glial cells deform the cell body and nucleus of the ganglion cell. b, an ameboid glial cell with two stump-like processes (maybe the rest of originally longer processes). c, a ganglion cell surrounded by six ameboid cells.*

*Table XXXIII, Fig. 8. Ganglion cell surrounded by five ameboid cells. The nucleus is deformed due to the encroachment of an ameboid cell. In this glial cell beginning degeneration of the nucleus. Of a strange severe disease in the climacterium.*

*Table XXXIII, Fig. 9. A ganglion cell with an ameboid cell intruded into the cell body. The nucleus of the ganglion cell is deformed and relocated. Caryohexis of the glial nucleus. At the nuclei of the other ameboid cells also regressive alterations. From the same case as in Fig. 8.*

*Table XXXIII, Fig. 10. Deformation of a ganglion cell and its nucleus by approaching ameboid cells.*

Not only the cell body, but also the protoplasmic processes are altered. What peculiar reorganization of cells can occur is shown best in Fig. 8, 9, 10 of Table XXXIII and the Figs 6, 10, 11, 12 and 13 of Table XXIX.


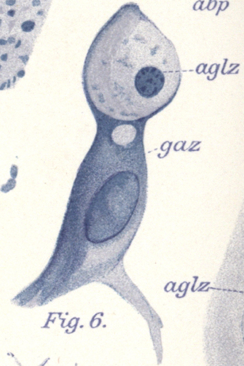

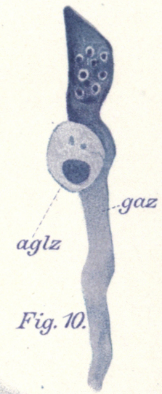

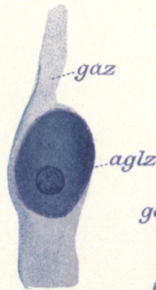

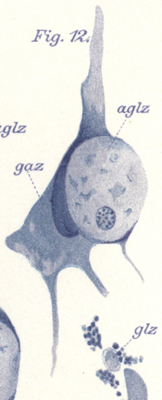

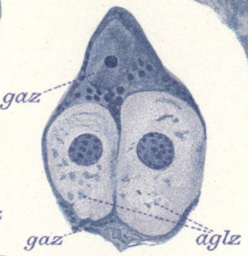


*Table XXIX, Fig. 6. Ganglion cell with the frontal process deformed by an ameboid glial cell. The frontal process is goblet-like pressed apart. Progressive chorea. Frontal, central sulcus.*

*Table XXIX, Fig. 10, 11, 12 and 13. Ameboid glial cells which lay in ganglion cells or their processes. Central sulcus. Progressive chorea.*

On those pictures the border of the cell body of glia and ganglion cell is often difficult to determine. It is not so rare that a small ameboid glial cell is positioned in a niche or cavity of a ganglion cell.

The acid-fuchsin-light green staining reveals best the relationship between glial cells and ganglion cells. Table XXX, Fig. 4 shows a series of such images from the cortex while Table XXX, Fig. 2 illustrates two individual glial cells from the grey matter of the spinal cord.


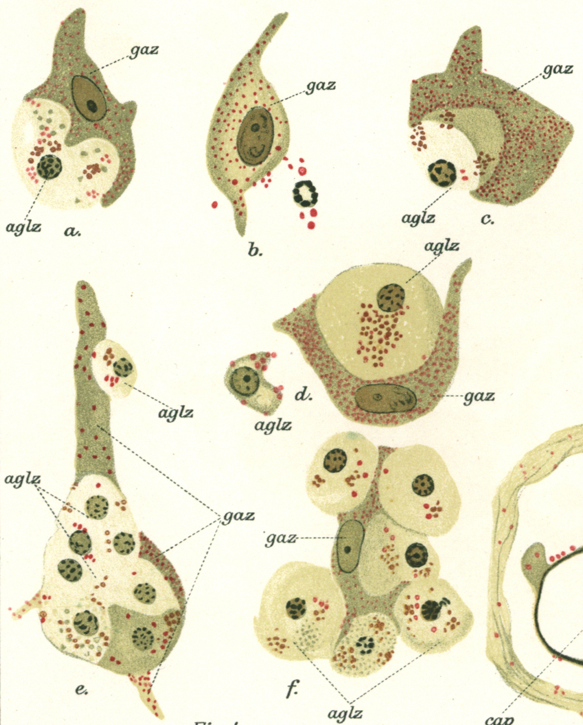


*Table XXX, Fig. 4. Ganglion cells with ameboid glial cells from the cortex of a case of delirium after acute progressive paralysis. Deformation of the cells. Substitution of the cell body by ameboid glial cells. Nuclear degeneration of the ameboid cells.*


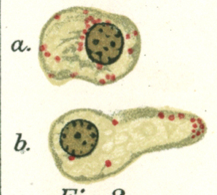


*Table XXX, Fig. 2 Ameboid glial cells from the ventral horn of the spinal cord of a paralysis with deliria with an acute course*.

We also see here more or less masses of fuchsinophil granules and besides that large number of osmium tanned substances stained with light green which are regularly found in older cells. How such an entire ganglion cell can be gradually replaced by such ameboid glial element is nicely shown in e and f of Fig. 4. The nuclei are also altered as described above. We often observe images as shown in Table XXIX, Fig. 7 and 9.


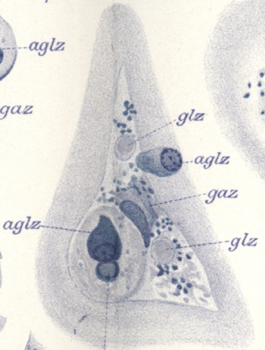

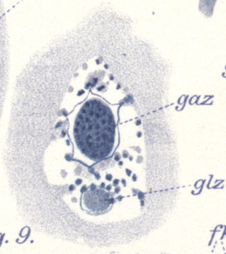
 *Table XXIX, Fig. 7. In a tissue gap which shows the form of a ganglion cell are remnants of a ganglion cell, two young ameboid glial cells and three degenerated nuclei which obviously belong to decaying ameboid ganglion cells. The same case (progressive chorea).*

*Table XXIX, Fig. 9. Status of a severe cellular alteration according to Nissl. Delirium-acutum-like condition at paralysis. In the tissue gap, which was filled by a ganglion cell, lays a degenerated nucleus of the ganglion cell with dissolved membrane and a degenerated glial cell nucleus, in between piles of granules.*

In gaps of the tissue, which occurred due to the disintegration of the ganglion cells, one finds rests of a nucleus, which allows to identify them as stemming from the ganglion cells. This is surrounded by a potpourri of granules which reside in regressive altered ameboid glial cells and in between

**431**

young, ameboid glial cells which obviously just started their development. When these glial cell forms are found at the ganglion cells and elsewhere in the tissue, they are also accumulated at the vessels (Table XXVIII, Fig. 3; Table XXXIII, Fig. 14). We will later deal with those.


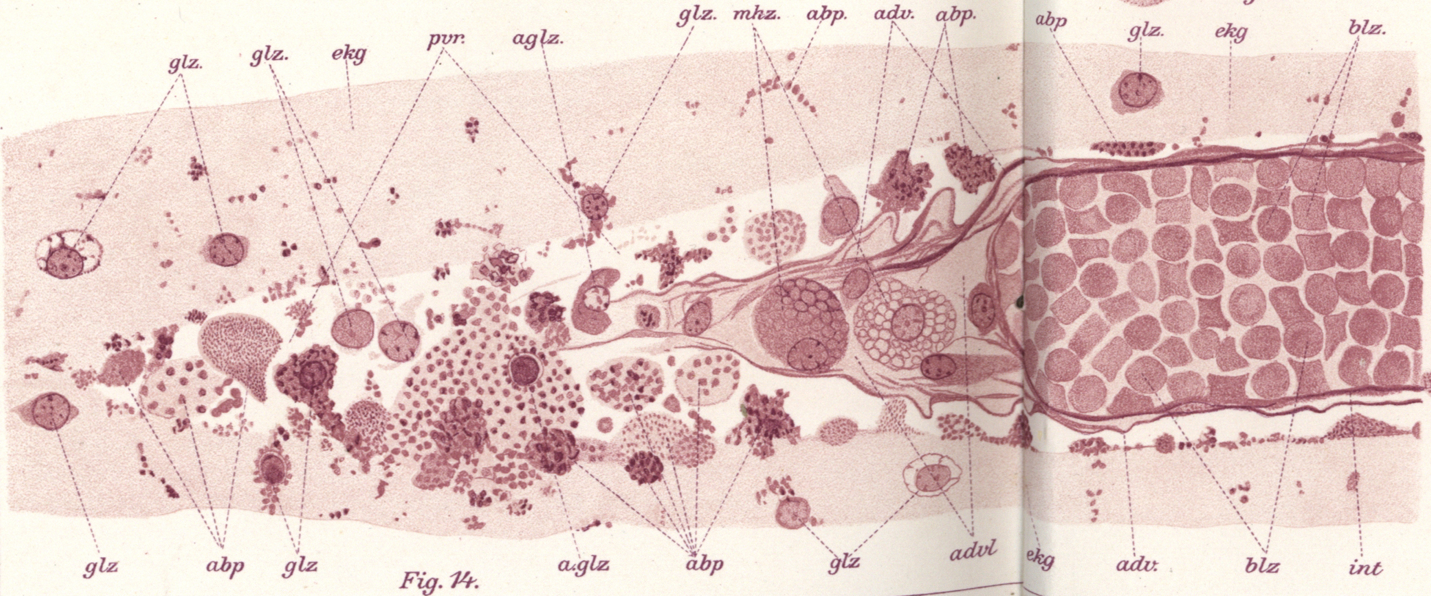


*Table XXXIII, Fig. 14. Small vein of the white matter of a case of ´fear psychosis´. One can clearly observe the perivascular space and the adventitial lymphatic space. In the latter are, besides other cells, two typical grid cells (granule cells). In the perivascular space are besides a number of young glial cells a young and a large ameboid cell; the later contains large methylene blue granules and a regressive altered nucleus and is alike the piles lacking nuclei in the perivascular space. In the nervous tissue young glial cells of different types, also some with methylene blue granules.*

In general, the above-described cell forms show many similarities to the ameboid cells of the white matter, despite deviating in their morphologic appearance. This becomes apparent if we study other pathologic glial forms which are also present in grey matter, where true ameboid glial cells are in the white matter. We observe in infectious deliria and in experimental intoxication glial elements in the cortex which form only a small, round cell body around the nucleus, not even half the diameter of the nucleus, often toward one side, but sharply delineated. In this small cell body there are many red, green, or brownish granules after fuchsin-light-green staining. One can assume these are miniature forms of ameboid cells (Table XXX, Fig. 3 l, m). But even the last remnant of an ameboid form is lost in pathologic glial forms, as shown in Table XXX, Fig. 1 a–c from an epileptic case which died in status epilepticus.


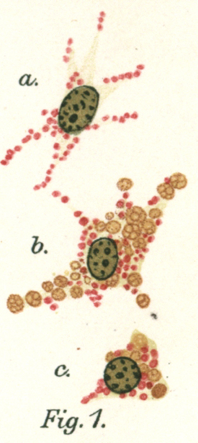
 *Table XXX, Fig. 1 a-c. Glial cells from the cortex of a case of epileptic deliria. a contains only fuchsinophil granules, b and c fuchsinophil granules and lipoid cysts.*

Among the many fuchsinophil granules found in the cortex and which belong to other structures such as nerve cells, neurosome accumulations, or axons, one can observe slightly smaller granules which should belong to glial cells, since they are often in long rows oriented toward the glial nucleus. Normal glial cells have very few if any fuchsinophil granules. In a normal brain, one does not find the arrangement of long rows of granules toward a glial nucleus. Based on this formation of rows, one can conclude that they are attributed to glial cells with ramified processes as shown in Table XXVIII, Fig. 1. Glial cells from the same cortex stained with Mallory hematoxylin support this assumption (Table XXVIII, Fig. 1 h, i).


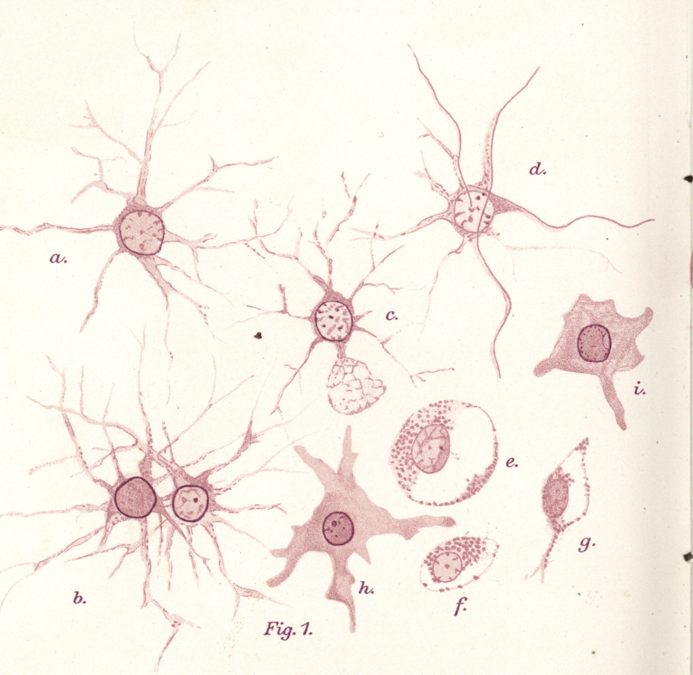


*Table XXVIII, Fig. 1 a, b. Normal glial cells with protoplasmic branches from the cortex of a 34-year-old man who died due to an accident. c glial cell from the cortex of a 68-year-old man diseased with arteriosclerosis. d glial cell from the deeper cortical layers of a 63 years old woman diseased with depression. e, f, g glial cells from the white matter of a 34-year-old man died due to an accident. h, I glial cells from the cortex of juvenile epileptic demented due to multiple seizures.*

They resemble the form of the normal branched glial elements, but their branching is more heavy-handed and massive. They are closer to the form of cells which I described in progressive Chorea Huntington.

**432**

The delicate cell body of these formations has retained little light green of the acid-fuchsin-light-green stain. Often, we observe no granules stained brown with osmium in these cells. Sometimes cysts occur, which we have observed in the ameboid cells of the white matter. They are significantly smaller. Sometimes one finds that the fuchsinophil granules are in the processes, and the brown cysts are around the protoplasmic cell body. These same elements we also observe in the neighborhood of vessels; often rows of red granules project to the walls of the vessels.
One finds all kinds of transition forms in the different brain diseases between these forms of cells which we often observed in epilepsy and the earlier described ameboid glial cells with abundant cytoplasm around the nucleus and without fine protoplasmic processes. We find glial cells with short processes or with only one longer one, miniature forms with roundish cell bodies with only one or some longer and shorter pseudopodal ramifications of the cell body with fuchsinophil granules and lipoid inclusions. In acute cases of dementia praecox (11) one finds such intermediate forms, which show the intrinsic connection of these different elements.
In other acute and subacute diseases, e.g., alcohol delirium, we can find a substantial accumulation of fuchsinophil granules around glial nuclei in acid-fuchsin-light-green preparations. A row of such granules also projects toward the ganglion cells or a vessel. It is difficult to correlate these abundantly dispersed fuchsinophil granules with individual glial cells if their cell body is not clearly labeled. With this, we have reached the limits of our methods which we cannot overcome despite all efforts.
In some brains in which ameboid glial cells are found in white matter, peculiar cells can be found there and in the grey matter which can be depicted with the Weigert glia method. Table XXXI provides a picture. They are characterized

**433**

by distinct granules which are stained blue with this method and which I will term fibrinoid. The name should not determine anything with respect to the chemical nature of these granules; the Weigert method labels, besides fibrin, also many other substances. It should be a preliminary name for common understanding.
Some of these cells are quite similar to cells shown in Table XXVIII, Fig. 11–14, raising the assumption that the methylene blue granules are identical to the fibrinoid granules.


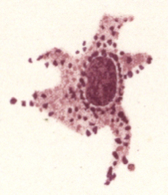

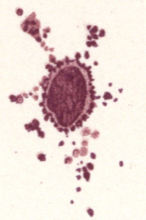

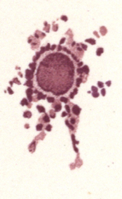


*Table XXVIII, Fig. 11. Glial cell with methyl blue granules in the arrangement of protoplasmic branching. Final delirium at phthisis.*

*Table XXVIII, Fig. 12 and 13. The same cells from the white mater of a suddenly deceased due to catatonia.*


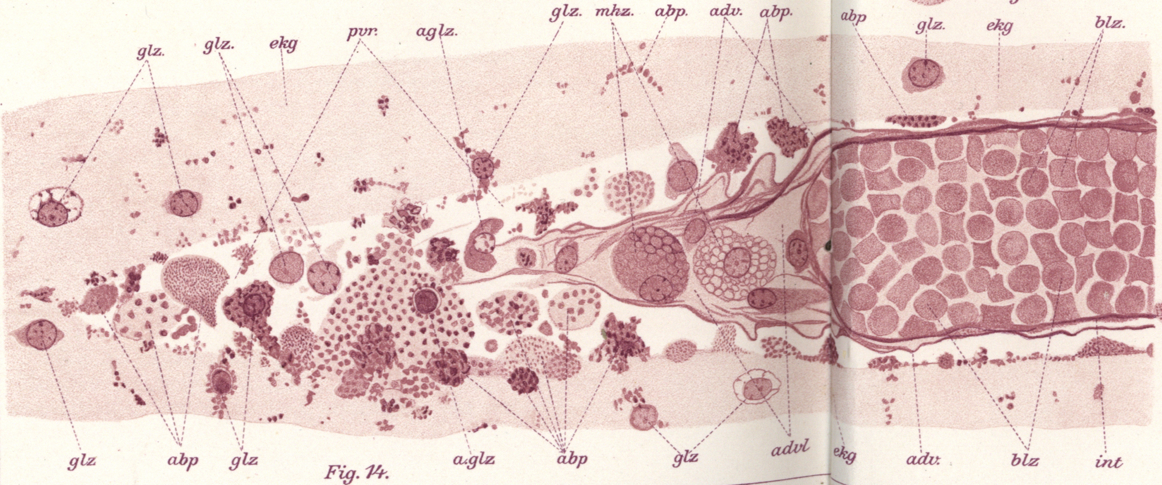


*Table XXVIII, Fig. 14. Small vein of the white matter of a case of ´fear psychosis´. One can clearly observe the perivascular space and the adventitial lymphatic space. In the latter are, besides other cells, two typical grid cells (granule cells). In the perivascular space are, besides a number of young glial cells, a young and a large ameboid cell; the later contains large methylene blue granules and a regressive altered nucleus and is alike the piles lacking nuclei in the perivascular space. In the nervous tissue young glial cells of different types, also some with methylene blue granules.*

If one compares preparations from the same brain with the two methods, it appears as if some cells contain granules which stain with both, the Weigert glia method and the Mallory hematoxylin or Mann’s stain. Yet the images obtained by that way do not coincide. In particular in the grey matter, I have never observed with other methods the masses of granules as labeled with the Weigert stain. It may be similar as with substances labeled by osmium or Scharlach, where some are reddened and blackened, others only reddened, and others only blackened. Most of the cells depicted on Table XXXI are from dementia praecox, ‘fear psychosis’, or severe infectious deliria. As we have seen with fuchsinophil granules of other glial cells, rows of blue granules aspire from a far distance to the glial nucleus, which is surrounded by a dense accumulation of these granules. Nothing can better illustrate how widespread the processes of a glial cell are than the images of Fig. 1, 2, 3, 5, and 14, which are all from the grey matter. If we imagine that there are protoplasmic bridges between the single granules, then we would obtain images as shown in Table XXVIII, Fig. 1 (see above).

Since the cells characterized by such granules are dispersed in the tissue, one finds granule rows or accumulations not associated with a nucleus, but this may be due to the fact that it is in the adjacent section. One does not get the impression that these granules are free, without relationship to a cell, despite the fact that one can often not document a cell plasm in which they reside.

**434**

It is remarkable that the sizes of the granules in the individual cells are similar, but can be different in different cells from the smallest dust-like granules (Fig. 5) to those quite coarse (Fig. 6). Quite often one can observe in the grey matter that a row of granules extends to a vessel (Fig. 4). Occasionally such cells are closely attached to a ganglion cell, and the row of granules passes over it or around it (Fig. 14).
The corresponding cells of the white matter have a slightly different form (Fig. 8, 9, 11, 12). The granules accumulate more at the nucleus, the processes are shorter, and frequently one can observe a faintly stained cytoplasm in which they are embedded. That makes it likely that also the fibrinoid granules of the delicate cells in the grey matter are within the cytoplasm and that their existence is not, as one could imagine, due to a disintegration of the cytoplasm. Often one can observe that the granules surrounding the nucleus are faintly or not even stained and can be recognized by a slightly yellowish tone. Sometimes the granules are moved away from the nucleus, resulting in a granule-free aura around it (Fig. 7). At any rate, these fibrinoid granules have nothing in common with the fuchsinophil granules.
Let us summarize these observations as follows:
In acute and subacute disease states of the central nervous system, one can find pathologic glial elements which usually rapidly arise and decay without generating glial fibers. In their prominent form, which we predominantly observe in white matter, their form reminds one of an ameba, thus designated as ameboid cells. The more or less uniform image of these cells in the white matter corresponds to different forms in the grey matter. There we observe elements similar to those in white matter, but also others which deviate with respect to their form so much that they cannot be termed ameboid cells. All these cells have in common that during their regression

**435**

different granules appear which we now have recognized as follows: the fuchsinophil, the light-green granules, the lipoid granules and cysts, the methylene blue granules, and the fibrinoid granules.

***B. About the changes at the vessels corresponding to the appearance of ameboid glial cells***

With the appearance of ameboid glial cells in the nervous system, we also observe changes at the vessels which hints to a relationship between the two. One finds various products deposited in the perivascular space at a time when degenerating ameboid glial cells are abundantly found in the nervous system, as determined on frozen slices treated with Mallory hematoxylin or Mann’s stain. These include roundish clusters or larger and smaller, quite irregular piles of granules and crumbs which can have all tones, from the faintest to an intense staining. In the clusters, one can distinguish a basic substance which is homogeneously and lighter stained and the dust parts, granules of darker color. The granules of the same cluster have similar size; in different clusters their size greatly varies. Sometimes all the formations within the same perivascular space are similar in color and size of the cluster and its granules, sometimes the clusters vary.

The defined staining of the adventitia with this method shows without doubt that these deposits are perivascular. In preparations labeled with Weigert’s glia stain which show the fibrinoid granules very well, we find inclusions in the perivascular spaces which show similarities to the ones described. Also in this case, these are clusters and accumulations of round and irregular forms, usually with a faint basic label and fine or coarse granules, while the granules of the same cluster are of similar size.

**436**

In contrast to the just described preparations fixed with the Weigert method and stained with Mann’s solution, the Mallory hematoxylin or the Weigert glia staining, preparations which are from the Flemming material and stained with acid fuchsin-light green stain have a different appearance. Table XXX, Fig. 7, 8, 9, and 10 show those images.


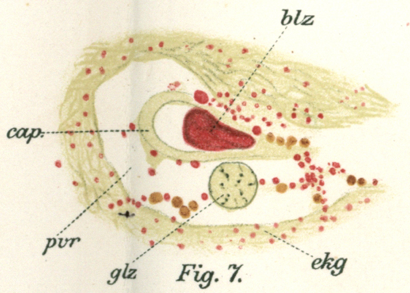

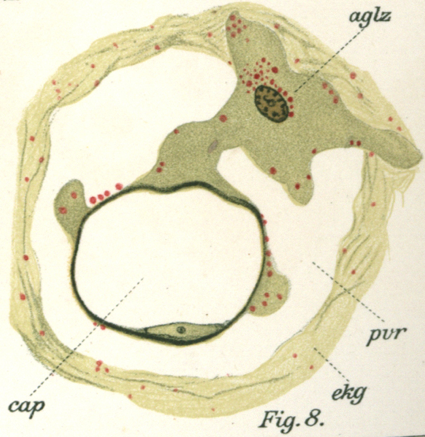


*Table XXX, Fig. 7. Capillary from the cortex of a case of epileptic delirium. In the perivascular space resides a glial nucleus, from which rows of granules emerge formed by lipoid and fuchsinophil granules. They continue to the ectodermal tissue on one side and to the vessel wall on the other.*

*Table XXX, Fig. 8. Large ameboid glial cells with fuchsinophil granules, which resides with part of their cell body in the nervous tissue and with the other it bridges a perivascular space and enwraps a capillary. Status epilepticus.*


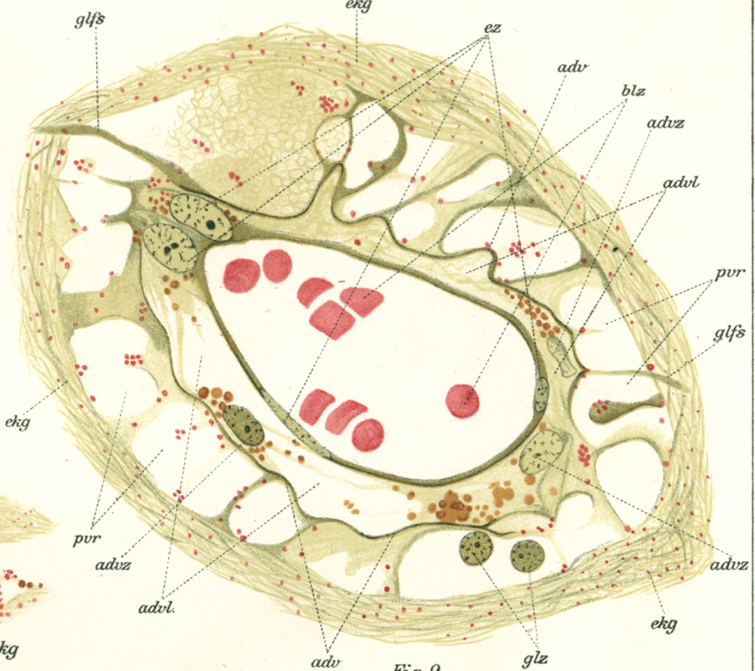


*Table XXX, Fig. 9. Small vein of the cortex of a case of epileptic delirium. The perivascular space is pervaded by band-type bridges in which fuchsinophil granules reside. Some of them which continue into the ectodermal tissue maybe viewed as processes of glial cells, the others may be considered as clotting products. In the perivascular space are no lipoid substances, in contrast to the adventitial lymphatic space and in the cells of the adventitia*.


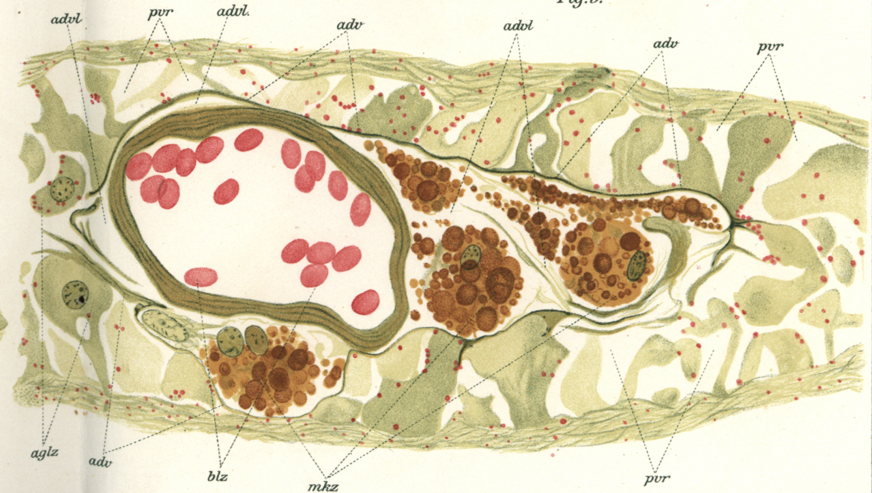


*Table XXX, Fig. 10. Two glial cells from the medulla oblongata loaded with basophil metachromatic substances from a case of an arteriosclerotic. The cell on the right shows at is lower part a honeycomb-like structure of the cytoplasm.*

Due to the distinct staining of the adventitia, the boundaries of the Virchow-Robin lymphatic space and also the perivascular space can be clearly recognized, at least at the larger vessels. In the latter, there are not the granular clusters as found in the preparations described above. In contrast, it is pervaded by numerous band-like bridges or small threads which are attached to the adventitia and sitting at the rim of the nervous tissue as a border line. Other, more slab-like formations are entirely free in the perivascular space. Sometimes these bridges are homogeneous structures, then also loosened as a coagulum. They commonly contain fuchsinophil granules.

Also in alcohol-fixed slices stained with toluidine blue or Giemsa solution or methyl-green-pyronin, one can observe such peculiar structures in the perivascular spaces (Table XXXIII, Fig. 14). They have been previously described by Cerletti. Tube- and half-moon-formed or roundish, one side stronger illuminated, delicate or dark-stained formations extend from the vessel wall through the perivascular wall to the nervous tissue. In their staining they remind one of the cell body of ameboid glial cells and one may consider that they are remnants of such fallen-apart cells. Some of those show such an intense color which is never found in deteriorating cytoplasm. Occasionally, the entire perivascular space is filled with this mass formed by these formations without a nucleus being observed — as I have seen in severe septic deliria.

**437**

What are these substances and how do they get into the perivascular space?
Based on the fact that they acquire a quite different appearance using different fixation methods, it implies that they are partially generated as artifacts by the applied reagents obtaining these forms as seen in the preparations. In particular, where the entire perivascular space is filled by this homogeneous mass, there is no doubt that these are coagulation products.
In the normal brain, we do not observe such formations. Here and there, we observe with the same methods sparse granules or threads, which one can consider as coagulation products of the lymphatic fluid, but never do we observe such intensively stained clusters and masses. These can only be pathologic products. But if they are as recent as after the hardening process converted into a solid form, one can consider the possibility that they are pushed from the neighboring tissue into the artificial perivascular shrinking space and then precipitate during the artificial shrinking of the tissue due to the fixation.
This explanation does hardly apply to another content of these perivascular spaces. Considering Table XXX, Fig. 8, one might consider that during the shrinking of the tissue, the contracting vessel could have pulled the attached glial cell out of the nervous tissue.


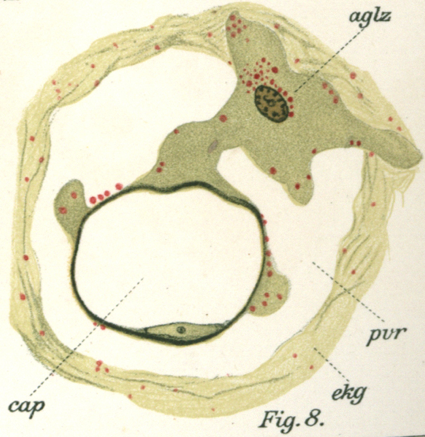
*Table XXX, Fig. 8. Large ameboid glial cells with fuchsinophil granules, which resides with part of their cell body in the nervous tissue and with the other it bridges to the perivascular space and enwraps a capillary. Status epilepticus.*

But it is hard to believe that large and small ameboid glial cells have been translocated in the same way into these spaces where they are located completely free as shown in Table XXVIII, Fig. 14, Table XXIX, Fig. 2, Table XXX, Fig. 10. In some of the clumps described above, one can observe remnants of nuclei, making it likely that they originate from degenerated glial cells as seen in clumps in Table XXVIII, Fig. 14.


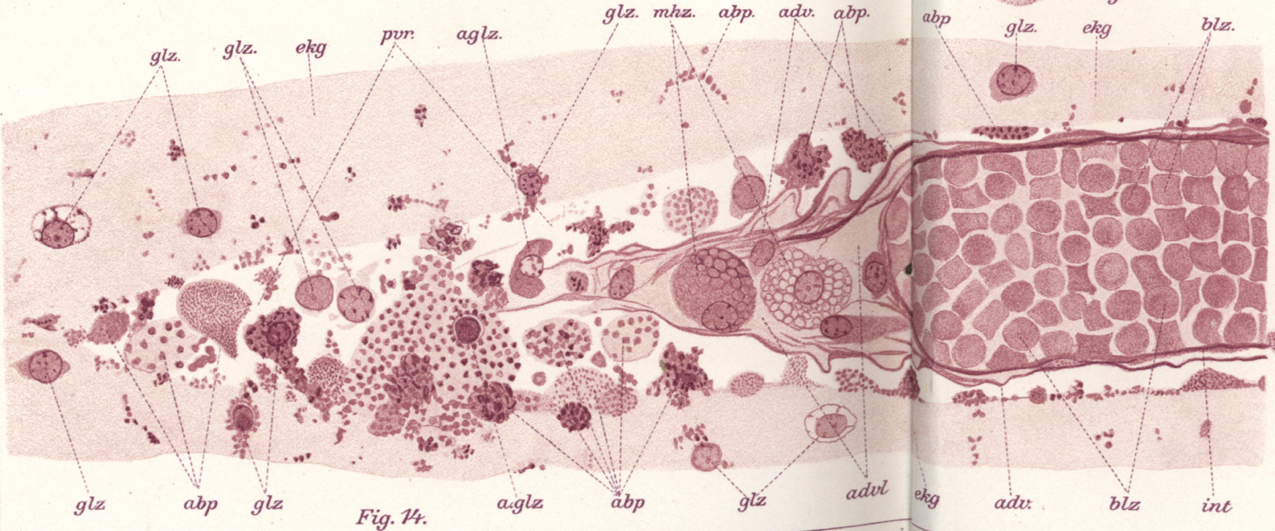


*Table XXVIII, Fig. 14. Small vein of the white matter of a case of ´fear psychosis´. One can clearly observe the perivascular space and the adventitial lymphatic space. In the latter are, besides other cells, two typical grid cells (granule cells). In the perivascular space are besides a number of young glial cells a young and a large ameboid cell; the later contains large methylene blue granules and a regressive altered nucleus and is alike the piles lacking nuclei in the perivascular space. In the nervous tissue young glial cells of different types, also some with methylene blue granules.*


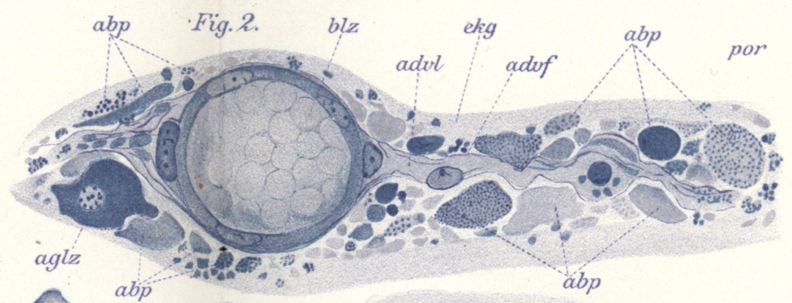


*Table XXIX, Fig. 2, Fig. 2. Small vein from the white matter of a cases of ´fear psychosis´ with band-like development of the adventitia. One can clearly observe the perivascular space in which there are large ameboid cells besides multiple piles of degenerative products.*


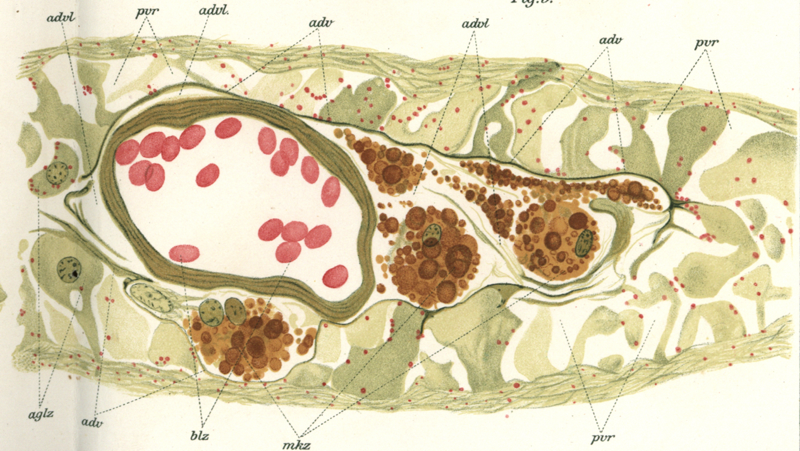


*Table XXX, Fig. 10. Vein from the white matter of a case of a deadly proceeded psychosis of the degenerated age. The perivascular space is almost everywhere bridged by broad bands in which fuchsinophil granules reside. Most of them terminate at nervous tissue and show no connection to nuclei. In between are two ameboid glial cells. In the perivascular space are no lipoid substances, large accumulations of those in the large granule cells of the adventitial lymphatic space.*

That all these products of the perivascular space originate from glial cells is not plausible since the clumps are much bigger than the largest ameboid cells, much too frequent, and definable in their form through the fixation. Thus, the frequent presence of complex ameboid cells in the perivascular spaces provides evidence that perivascular spaces exist under pathological conditions. That they also normally exist cannot be concluded.

**438**

Furthermore, these observations lead to the assumption that in these cases a membrana perivascularis gliae does not exist. That it normally does not exist, cannot be concluded. The methods which we have used display the glial border membrane only very inadequate. By direct observation, we recognize very little. I believe that I have sometimes observed that numerous ameboid glial cells (between them were filled bodies as later discussed) push a delicate membrane against the perivascular space. I also observed that ameboid glial cells extend into the perivascular space like trough a brake pass and where I could recognize the border membrane in its extend. There a limitans could not be recognized. Yet, those observations are only singular and could potentially be explained by the stretch and rupture of frozen slices and are not clear evidence. Often, one can observe that the glial cells are positioned half in the perivascular space, half in the nervous tissue and furthermore that the above-described clumps and granules extend from the nervous tissue into these spaces. This indicates that a membrana limitans which may normally exist, has now disappeared.

Images from cortical areas which show numerous ameboid cells in full development and few in degeneration indicate that at least part of the perivascular spaces and its enlargement come about due to the disintegration of ameboid glial cells accumulated around vessels. One can observe that the ectodermal tissue is still attached at the adventitia, but the glial cells which directly surround the vessel are largely dissolved into methyl blue granules. This generates gaps in the tissue which could generate a perivascular space due to the dissolvement of the limitans. Through such gaps ameboid glial cells could enter into the perivascular space without much resistance. Similarly, degradation products of nervous and glial structures could enter partially as solid partially in semi-fluid or fully fluid condition; they will precipitate from the tissue fluid which fills the space due to the fixation.

**439**

More difficult and less obvious as compared to the large vessels in white matter is the relationship at the small ones in particular in grey matter. After staining with Mallory hemoxylin we find in very severe cases similar products as in white matter. Using acidic fuchsin-green staining, one observes also here green labelled threads or bridges spanning through the perivascular space, yet they are more delicate. Where ameboid glial cells have acquired roundish forms, such round cells, partially young and fresh, mostly regressively altered, are freely positioned in the perivascular space (Table XXVIII, Fig. 3).


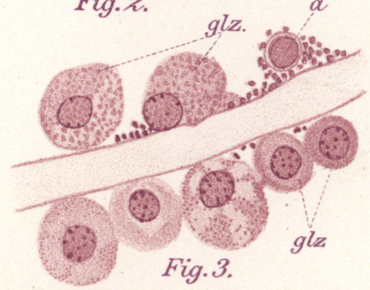
*Table XXVIII, Fig. 3. A capillary from the cortex surrounded by younger and older ameboid cells; in the older cell beginning degradation of the cytoplasm, at a, acell with methylene blue granules.*

Where one finds cells with long rows of fuchsinophil granules, one observes that that the rows of granules pass through a perivascular space and attach to a vessel. Occasionally, also the nucleus is positioned free in the perivascular space and its granules, partially brownish, partially reddish labelled are oriented on one side towards the vessel and are on the other side lost in the nervous tissue. Often vessels which are apparently not surrounded by a perivascular space, but are surrounded by fuchsinophil granules which are partially within glial processes and which partially cannot be associated with them. Other vessels are within a large empty space and are covered by fuchsinophil granules. The interpretation of these observations is impeded, since it is impossible to decide whether we face an artificial space due to shrinking or a true perivascular space or that an existing perivascular space has been significantly expanded due to shrinking and that the natural conditions are deformed.

At places where we find the perivascular products, there are usually alterations in the adventitia and the adventitial lymphatic spaces. We find more or less numerous inclusions in the adventitial cells. They are partially similarly shining reddish labelled such as fuchsinophil granules, yet usually much larger, partially tanned or blackened by osmium. One can also often find granules which have a blackened shell and a red core. If the mass of the perivascular products is high, the cells of the adventitia enlarge. Usually, one observes only nuclei with little cytoplasm and connective tissue fibers.

**440**

Now one can observe around some nuclei an obvious cytoplasmic cell body which is elongated either at one pole of the nucleus or at both into a long, dark labelled process. One can also observe cells with a round cell body. Using a cytoplasm stain, one can display a meshwork while other methods which display the degradation products within the meshwork show mainly tanned and blackened, rarely reddish granules.

When the mass of the perivascular products is heavy, one can find lymphocytic elements in the adventitial lymphatic space, in particular at the larger vessels of the white matter, where the perivascular products are abundant. There one finds large round cells with a larger nucleus. The cell body is homogeneously stained in the early stage and I believe that they are the mother cells of many granule, respective grid cells, which we find there at later stages. Table XXVIII, Fig. 14 shows two typical ones of those cells in the adventitial lymphatic space.


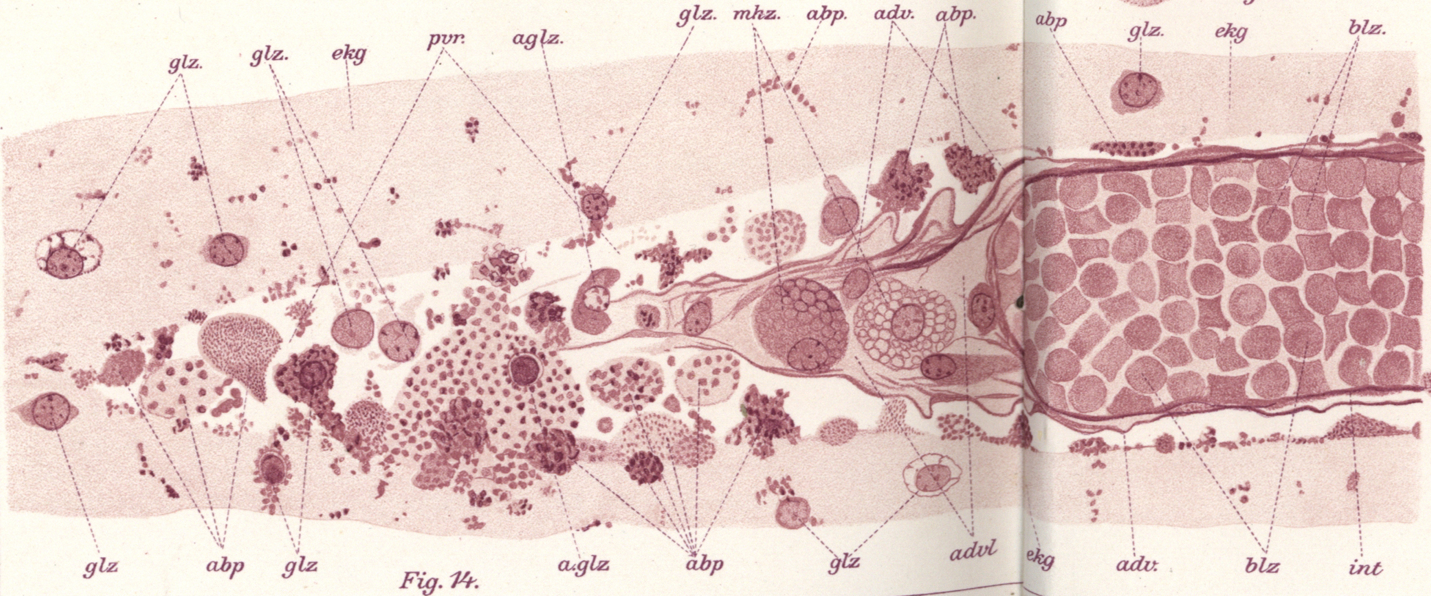


*Table XXVIII, Fig. 14. Small vein of the white matter of a case of ´fear psychosis´. One can clearly observe the perivascular space and the adventitial lymphatic space. In the latter are, besides other cells, two typical grid cells (granule cells). In the perivascular space are, besides a number of young glial cells, a young and a large ameboid cell; the later contains large methylene blue granules and a regressive altered nucleus and is alike the piles lacking nuclei in the perivascular space. In the nervous tissue young glial cells of different types, also some with methylene blue granules.*

While there are only few blackened or reddish granules in the perivascular space as stained by application of osmium or Scharlach, the number of labelled substances in the adventitia and the adventitial lymphatic space is enormous. One should observe the images in Table XXXIV, Fig. 4, 5 and 7 – images which can be obtained from all cortical diseases leading to the degeneration of the nervous tissue. Also, very nicely can the same relation be seen in images from Flemming material (Table XXX, Fig. 9 and 10) where we do not observe any tanned substances in the perivascular space, while they can be observed in Fig. 9 in the cells of the adventitia and piled up in the in large amounts in the granule cells.


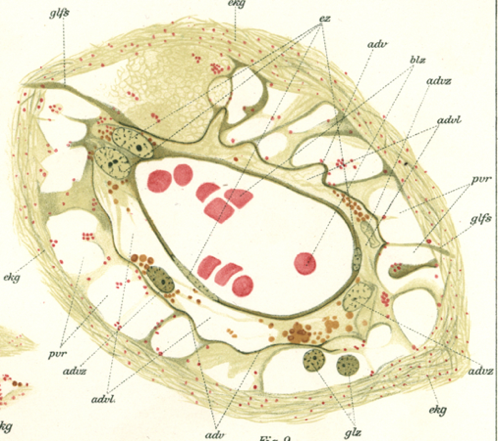


*Table XXX, Fig. 9. Small vein of the cortex of a case of epileptic delirium. The perivascular space is pervaded by band-type bridges in which fuchsinophil granules reside. Some of them which continue into the ectodermal tissue maybe viewed as processes of glial cells, the others may be considered as clotting products. In the perivascular space are no lipoid substances, in contrast to the adventitial lymphatic space and in the cells of the adventitia.*


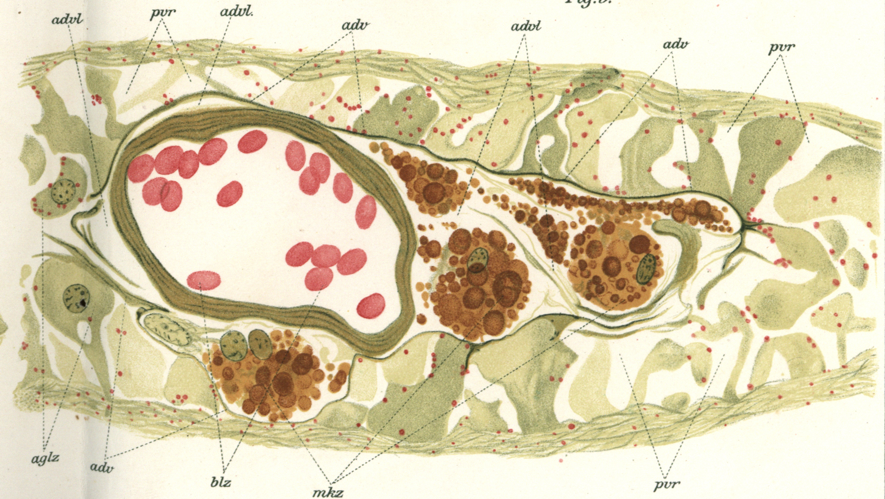


*Table XXX, Fig. 10. Vein from the white matter of a case of a deadly proceeded psychosis of the degenerated age. The perivascular space is almost everywhere bridged by broad bands in which fuchsinophil granules reside. Most of them terminate at nervous tissue and show no connection to nuclei. In between are two ameboid glial cells. In the perivascular space are no lipoid substances, large accumulations of those in the large granule cells of the adventitial lymphatic space.*

The application of different staining methods shows us that the incorporated substances are not homogeneous; we will later come back to that.

Furthermore, these fat-storing cells undergo regressive changes. The nucleus will be finally pressed to the periphery, flattened, stained diffusely and dark and acquires an irregular, angled form. Gradually, the lipoid substances are again washed out;

**441**

Finally, one finds often cells with only remnants of a reticular plasm, but no incorporations can be detected.
We thus observe that diverse, peculiar substances accumulate in the perivascular spaces wherever ameboid glial cells appear in the tissue in large number. Their morphological diversity revealed by the application of different fixation methods makes it likely that they are, at least in part, coagulation products from the pathologic tissue fluid. Besides that, one finds ameboid glial cells, fresh ones and those which show signs of deterioration in the same spaces. It indicates that the glial border membrane is dissolved and that under pathologic conditions perivascular spaces can be found which cannot be interpreted as artificial shrinkage artifacts. Wherever these changes occur around the vessels, many cells of the adventitia and certain cells of the adventitial lymphatic space convert into fat granule cells.

***C. The pia and the degeneration process in the nervous tissue.***
The described events which occur in the cells of the adventitia and in the adventitial lymphatic space are also accompanied by changes in the large pial lymphatic space. Previously, it was generally considered that rearrangement of the pia is only influenced by those events in the central nervous system which are linked to a shrinkage of the cortex, resulting in a thickening of the pia except for meningitis or paralysis. It should fulfill the purpose to fill the empty space. The filling of an empty space cannot be the only trigger of the pial alterations. This is implied by the observation that rearrangements already occur when the nervous system is not yet atrophic and when a swelling of the ganglion cells, an increase of the glial cytoplasm, and the occurrence of ameboid cells result rather in a volume increase of the brain.

**442**

We also observe the appearance of extracellular products at acute disease processes, which are similar to the ones in the perivascular spaces, but can be partially different. In very severe diseases of the cortex, they can be observed to occur in large masses. Their investigation is impeded by the fact that the pial fluid is often mixed with blood serum due to the rupture or cutting of pial vessels as this occurs often during sectioning. On the other hand, pathologic substances of the pia are easily taken up by adjacent fluid compartments due to the fixation. Yet it is easier to obtain fresh preparations as compared to the nervous system. One can detach the pia and transfer it directly into different staining solutions. Very useful is neutral red (de Montet).

Already in Nissl preparations, we often observe a peculiar cloudiness of the pia as a result of an incorporation of single granules, granular clumps, small particles, and slabs, commonly unstained substances which become apparent when lowering the illumination. Some have taken up the basic Anilin stains to a different extent, others appear greenish, similar to the pigmented lipoid substance of the nervous tissue. With the Weigert’s glia stain we often observe clusters or clumps of blue-stained granules of different size. Using Mann’s stain we observe similar large blue-stained clumps. Very rapidly occur also alterations in the cellular elements of the pia. The fibroblasts and the cells of the adventitia show enlargements of their cell body resulting in a roundish form. Subsequently, they develop one or two large, slab-like processes. Staining that labels the cytoplasm shows an enlarged cell body with a net-type structure, staining that illustrates the content of the net as incorporation of lipoid substances. Very soon the mass of the deposited, fatty substances can become quite significant. This can be well illustrated using the carbol-fuchsin-methylene blue staining. Besides fibroblasts, we always find single lymphocytic elements and peculiar blood elements with a large, round, and well-stained cell body which we have already encountered in the adventitial

**443**

lymphatic sheath. They later transform into grid cells. In between are several fibroblasts which have increased beyond their normal size and have formed several nuclei. They are loaded with fatty products.
One finds in general that these changes are less pronounced at the peak of the gyri as compared with the sulci and in particular at the depth of the sulci. It may be due to the fact that more lymphatic fluid accumulates or that degradation products come together from two sides.
Concomitant with the changes in the nervous tissue which are characterized by the appearance of ameboid glial cells, occur changes in the pia. They are characterized by a deposition of different extracellular substances, by proliferation of the cellular elements, and conversion of those into grid cells loaded with lipoid products.

***D. The filled bodies and their relationship to glia.***
In slices of the nervous system where multiple ameboid cells are present, one often finds peculiar extracellular formations, and one must assume that they are related to disease states. In particular, one form seems to be closely associated with the presence of ameboid glial cells and due to its numerous numbers of significant pathological importance. It will therefore be discussed in relation to ameboid glial cells. Other extracellular products which are not associated with ameboid glial cells will be later discussed.
I would like to term these extracellular formations ‘filled bodies’. It is not easy to comprehend them. At best, one can obtain an understanding of their origin in the spinal cord where the circumstances are simplest and straightforward. In the drawings 19 and 20 of Table XXIX, which both show sections from the white matter of the spinal cord — Fig. 19 from a young goat which corresponds well to the white matter

**444**

of the human spinal cord, Fig. 20 from a deceased human of a progressive Chorea Huntington are carefully all details depicted, except for the red labelled white matter sheaths. In Fig. 19 one can observe single glial nuclei with clear protoplasmic cell body and numerous glial fibers, from which a part is shown in longitudinal course, others in short inclined sections and most in dot-like cross sections. A very delicate staining of the substance between the fibers shows that it is embedded in a peculiar modified plasm of the glial reticulum.


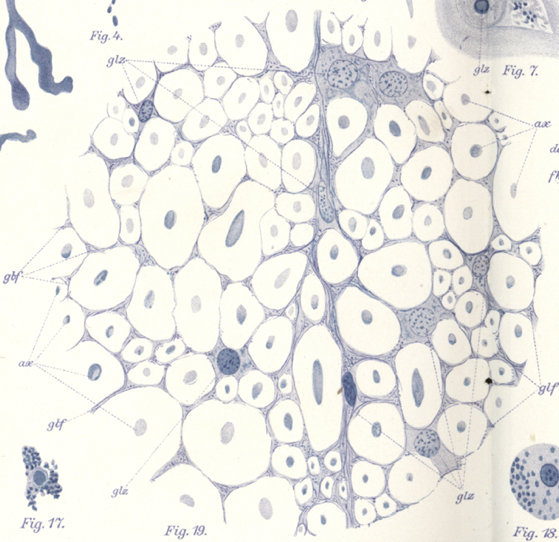


*Table XXIX, Fig. 19. Slice from the white matter of spinal cord from a half-grown goat. glz (glial cell), glf (glial fiber), ax (axon).*


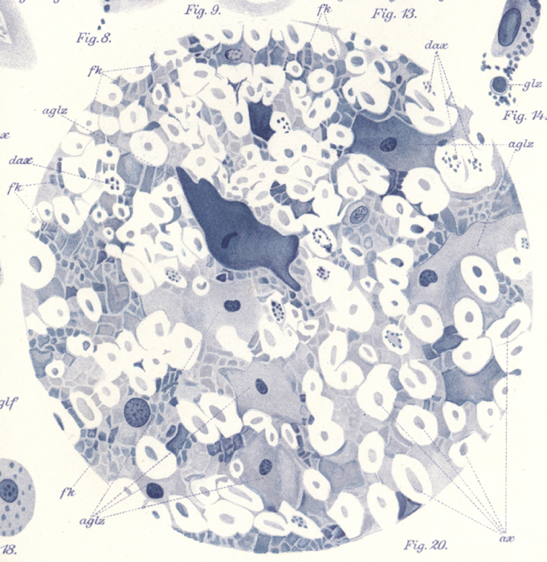
Fig. 20 shows other relations. One cannot recognize any hint with respect to glial fibers. A large number of large faintly and some strongly stained ameboid glial cells dominate the image. Besides those we can observe mosaic-like composed bodies filling the space between the down-sized nerve fibers. They are similarly stained than the protoplasm of the glial cells. If we imagine that each of the small fibers as shown in Fig. 19 is tremendously swollen which cannot be recognized in Fig. 20, it would explain such a picture. Originally, I have interpreted it that way. But obviously it is not so simple. It is obvious that one rarely observes in longitudinal slices a fiber which is broadened to a band. If one inspects longitudinal instead of cross sectioned slices in the same spinal cord to clarify the relationship, one finds only mosaic-type areas and only rarely longer band-type stripes. In particular around the vessels, the filled bodies form multi-layered, thick covers. This must indicate that these are not cross-sections of protoplasmic cellular processes, but rather freely arranged bodies which are not in connection with cells, but are rather arranged next and on top of each other like copper stones.

*Table XXIX, Fig. 20. Slice from the white matter of the spinal cord from a case of progressive chorea. aglz (ameboid glial cell), ax (axon), dax (degenerated axon), fk (filled body).*

Where are these bodies from? If one studies some locations of the figure, in particular in the neighborhood of the ameboid cells on the top right, one may consider that they are released from ameboid cells. We find such bodies also in the grey matter in the neighborhood of ameboid cells as shown in Fig. 3 of Table XXXV where they are located in large number on the bottom right next to a group of ameboid cells.

**445**


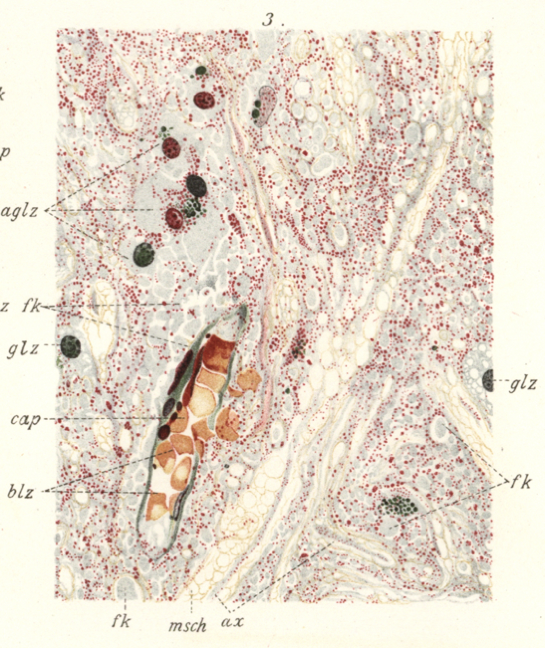
*Table XXXV, Fig. 3. From a slice through the spinal cord of a hare which had died due to a bacterial infection with ascending paralysis. Border between dorsal horn and dorsal strand. In the neighbourhood of a capillary, partially in the perivascular space, partially in the nervous tissue are multiple larger filled bodies and among them larger ameboid glial cells with fuchsinophil and lipoid granules. Filled bodies are dispersed throughout the entire tissue. aglz (ameboid glial cell), ax (axon), blz (red blood cell), cap (capillary), (dax (degenerated axon), fk (filled body), glz (glial cell), msch (myelin sheath)*

Yet such an issue has little analogies and it requires further evidence. Occasionally one finds large filled bodies which are similar to the cell body of an ameboid cells, but do not contain a nucleus. In others, one can observe remnants of degenerated nuclei, indicating that they originated from degenerated cells. But this does not apply to all, considering the large number in which these formations appear (Table XXIX, Fig. 1, , Table XXXV, Fig. 2, 3, 4).


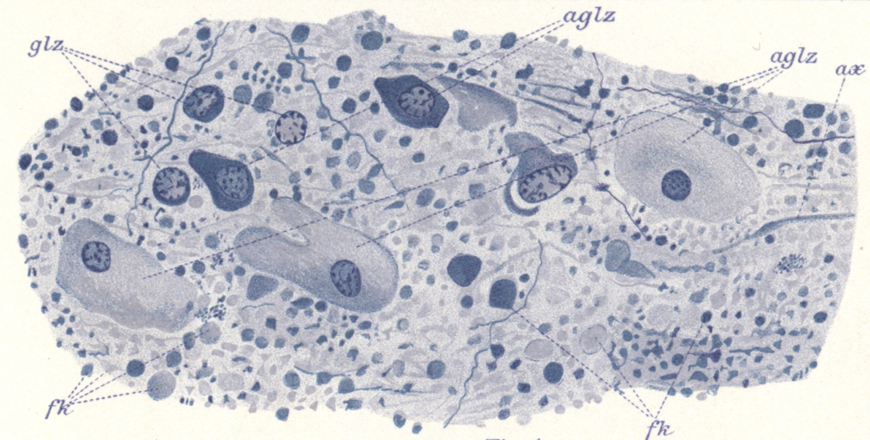
*Table XXIX, Fig. 1. Section of the corpus striatum close to the ventricular surface from a case of progressive chorea. The tissue is seeded with filled bodies, besides are some ameboid glial cells, some glial nuclei with little cytoplasm, single glial fibers and axons visible. aglz (ameboid glial cell), ax (axon), fk (filled body), glz (glial cell)*


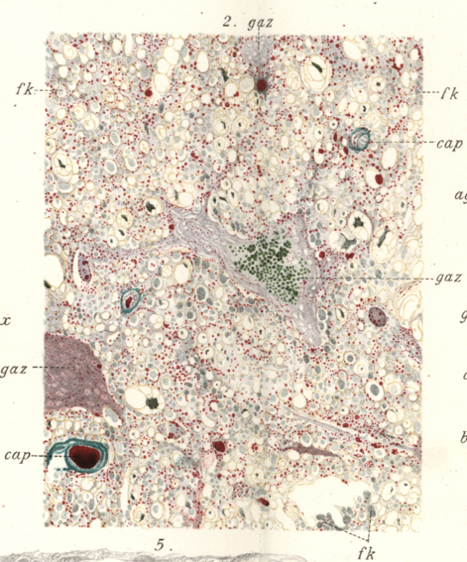


*Table XXXV, Fig. 2. Degenerated section from the ventral horn of a case of progressive chorea. In the middle a ganglion cell with the nucleus not sliced with multiple lipoid inclusions. Piles of neurosomes are only occasionally observed. The axon and the protoplasmic processes of the ganglion cells are hardly visible. The entire tissue is dispersed with greenish, roundish bodies (filled bodies). aglz (ameboid glial cell), ax (axon), cap (capillary), (dax (degenerated axon), fk (filled body), glz (glial cell)*


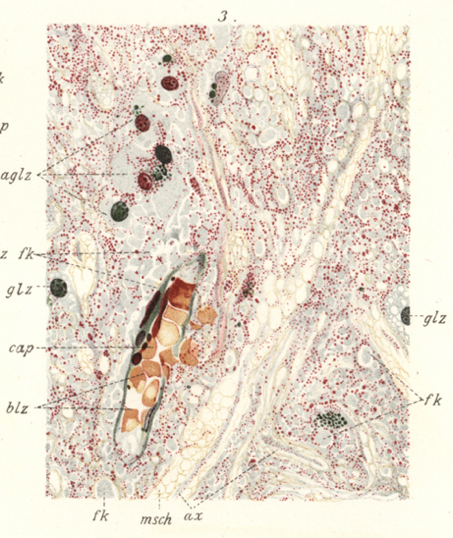


*Table XXXV, Fig. 3 From a slice through the spinal cord of a hare which had died due to a bacterial infection with ascending paralysis. Border between dorsal horn and dorsal strand.in the neighbourhood of a capillary, partially in the perivascular space, partially in the nervous tissue are multiple larger filled bodies and among them larger ameboid glial cells with fuchsinophil and lipoid granules. Filled bodies are dispersed throughout the entire tissue. aglz (ameboid glial cell), ax (axon), blz (red blood cell), cap (capillary), (dax (degenerated axon), fk (filled body), glz (glial cell), msch (myelin sheath*)


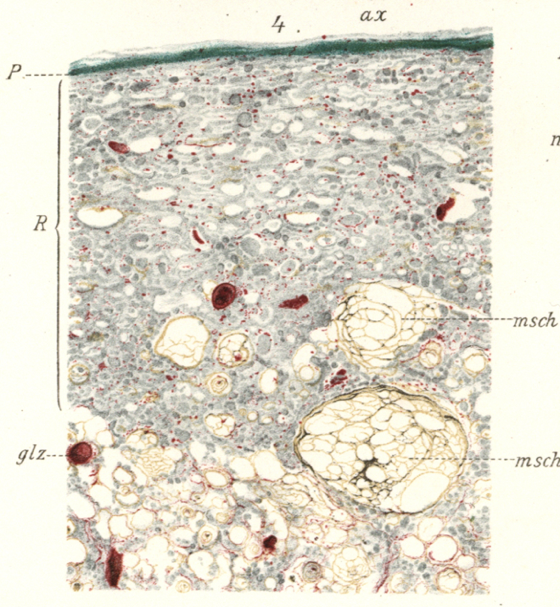
*Table XXXV, Fig. 4 Cut through the superficial border layer of the spinal cord from a case of acute progressive paralysis which died due to sepsis. Instead of a surface layer formed by glial cells, we observe masses of filled bodies, which are also present as small piles between the myelinated fibers., glz (glial cell), msch (myelin sheath)*

It is a remarkable observation that glial fibers are devoid or strongly reduced wherever filled bodies are present in large numbers. One may consider that their existence is related to the post mortal transformation of glial fibers. Since the studies of Weigert it is known that they disintegrate after death.

Due to a lucky coincidence, it was possible to resolve the issue without doubt. I had purchased a number of macaques for experimental studies. They, however fell ill to an epidemic which caused a persistent diarrhea. They massively lost weight, and a disection of a deceased indicated that they had ulcer in the intestine and purulence regions in the brain. Since also the others seemed to be lost, I sacrificed them and placed the nervous system right after dissection into different fixation solutions.

In the grey and white matter of the spinal cord there were ameboid glial cells besides alteration in ganglion cells indicating a severe Nissl disease. The degree of these changes varied between the different animals and it was possible to obtain images as shown in Table XXIX, Fig. 19 and 20. All stages of the same process could be compared.


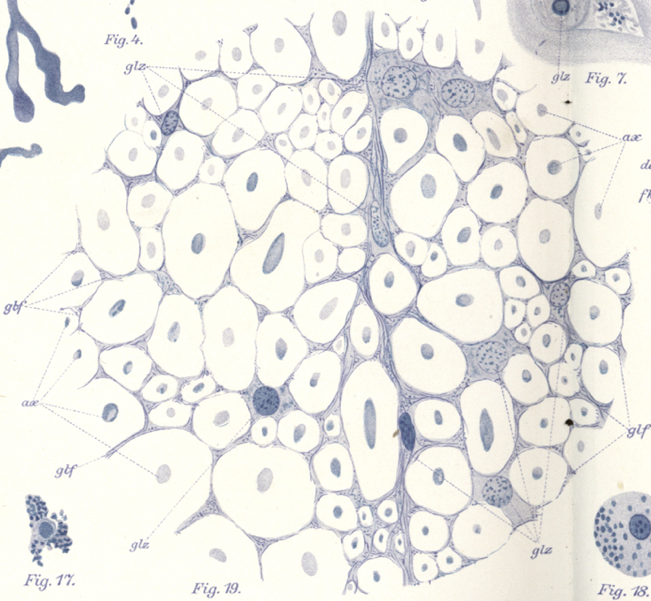

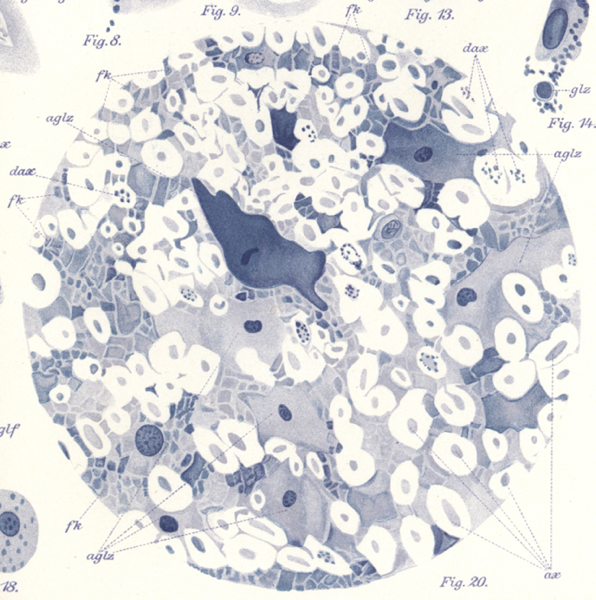


*Table XXIX, Fig. 19 Slice from the white matter of spinal cord from a half-grown goat.*

*Table XXIX, Fig. 20 Slice from the white matter of the spinal cord from a case of progressive chorea.*

In normal regions, the glial border membrane was formed by very delicate, red labelled glial fibers after staining with acidic fuchsin light green. A delicate greenish label between the fibers indicated that they were embedded in a protoplasmic substance. At other regions with obvious alterations, one did not observe red fibers, but rather

**446**

greenish stained, slightly broader bands, in the same arrangement as the glial fibers. They were broader than those and one could observe that some had a relation to nuclei which were below the border layer or even within. In between appeared single cells with the features of young ameboid glial cells and multiple roundish or angled formations, were our filled bodies. In regions of advanced disease, the images were dominated by ameboid cells and filled bodies. Fibers or an arrangement of a normal surface layer could no longer be observed. Below the filled bodies were some coagulated, granular formations of irregular shape, potentially coagulation products of altered lymphatic fluid which had been between the tissue elements, potentially degrading filled bodies.
Based on the observations, one can conclude that glial fibers dissolve under pathologic conditions. In our preparations, it happened that the fibers swelled together with the attached protoplasm, and that these swollen structures disintegrated and resulted in the generation of the filled bodies.
The images of Table XXIX, Figs. 4, 5, 8, and 16 which we have discussed earlier represent similar processes. Long protoplasmic processes of glial cells acquire a form like a rosary and disassemble into single formations corresponding to the filled bodies.


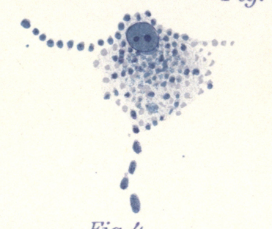

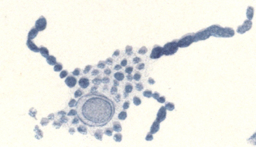

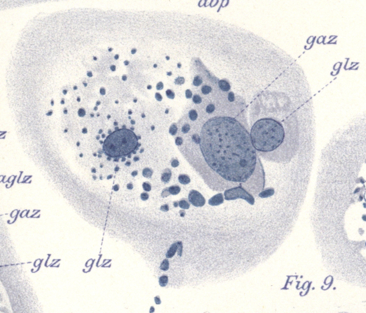

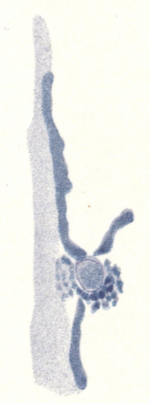


*Table XXIX, Fig. 4, 5. Similar cells of the same case (progressive chorea), frontal, central sulcus, with rosary-like processes, partly disintegrated in single pieces. Degeneration at the nucleus.*

*Table XXIX, Fig. 8 Ganglion cells surrounded by a large perivascular space in which degradation products of an ameboid glial cells are positioned. The same case (progressive chorea).*

*Table XXIX, Fig. 16 Different forms and decay stages of ameboid glial cells at a progressive chorea.*

In the human central nervous system, we easily find single stages of the same process. Based on the material from that experiment, one can identify all the stages of that process. A composition of single images from human disease states indicates that the filled bodies are generated in the same way. In a case of acute meningitis due to tuberculosis of the spinal cord, one could find that the fibers of the border layer were replaced in certain regions by protoplasmic fibers of considerable width. Several could be traced up to the slightly swollen cell bodies of glial cells which were within or below the border layer. In between were some ameboid glial cells. In some regions, the membrana limitans was extended and below was a cluster of large ameboid cells. In other regions, one no longer observed a membrana limitans,

**447**

and ameboid cells had pushed beyond the border of the spinal cord into the pial tissue. In the stage of reparation, glial fibers could grow at these locations beyond the original surface layer as one can observe in older paralysis cases or in some chronic forms of meningomyelitis.
Another advanced stage is shown in Fig. 4 of Table XXXV. It was obtained from a case of rapidly progressing paralysis in a patient who had died of sepsis. Potentially the latter was not without influence on the changes.


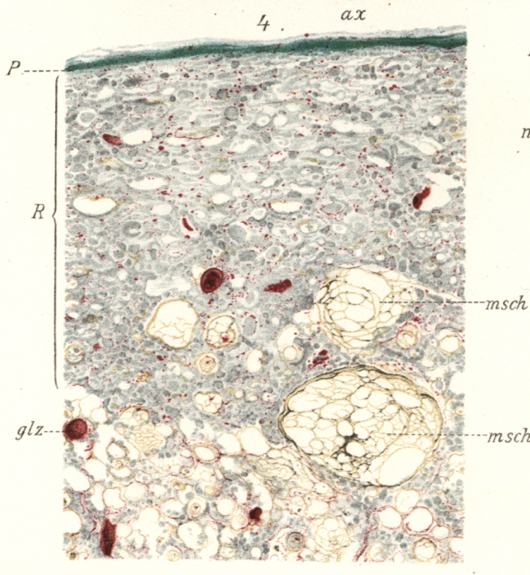
*Table XXXV, Fig. 4 Cut through the superficial border layer of the spinal cord from a case of acute progressive paralysis which died due to sepsis. Instead of a surface layer formed by glial cells, we observe masses of filled bodies, which are also present as small piles between the myelinated fibers*.

On top, we can see the dark green-stained connective tissue fibers of the pia. It is only partially illustrated. Without an obvious border membrane the glial surface layer of the spinal cord starts which occupies two-thirds of the image; only in the lower third, we find cross sections of larger and smaller axons stained yellowish or brownish. At the border membrane, which is normally formed by glial fibers being red stained in cross or longitudinal sections, we find only single red dots relating to glial fibers. The majority of the tissue consists of filled bodies which also occur between the axons as small clusters.


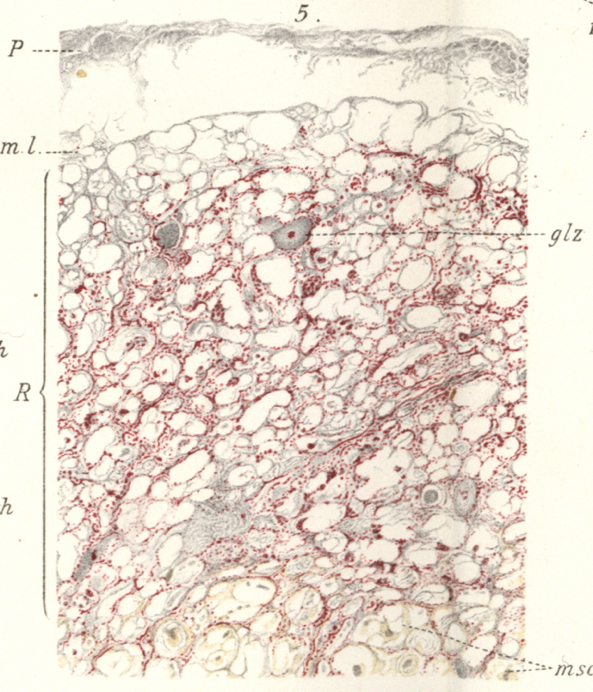


The degree of the changes becomes apparent when studying Fig. 5 of the same Table which is from a case of chronic paralysis. We also observe on top some connective tissue fibers of the pia, an obvious membrana limitans, and four-fifths of the image fill the border layer which consists almost exclusively of red-labelled cross sections of glial fibers. Only a little net-type, delicately labelled protoplasm is in between. Filled bodies are not present.

*Table XXXV, Fig. 5 Cut through the border layer of the spinal cord of an old case of paralysis. The strongly thickened surface layer is formed almost exclusively by perpendicular cut glial fibers.*

How massively these formations can occur in the grey matter under pathologic conditions is best seen when comparing Figs. 1 and 2 of Table XXXV. Fig. 1 shows a normal cell from a dog.


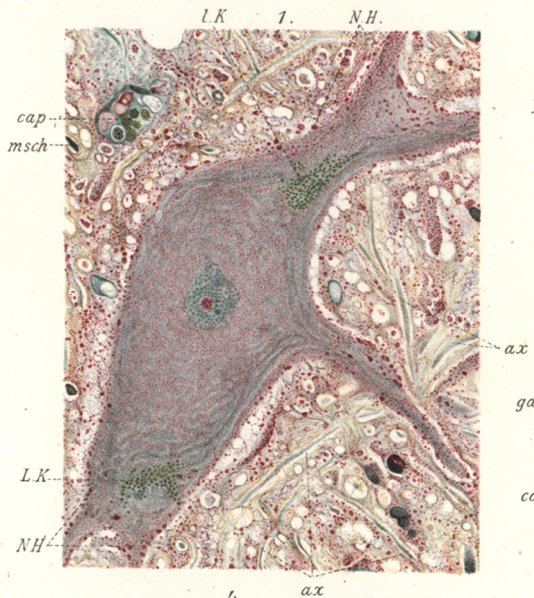


*Table XXXV, Fig. 1 Ganglion cell from the ventral horn of a normal, older dog. In the cells are green Nissl slabs and fuchsinophil granules associated to the cytoplasm. At two locations are lipoid granules. At the rim of the cell and at processes are red labelled piles of neurosomes visible. At the capillary are lipoid substances. In the tissue, one observes multiple myelin sheaths and axons. The rest is largely filled with protoplasmic processes of neighbouring ganglion cells, which are embedded by piles of neurosomes.*


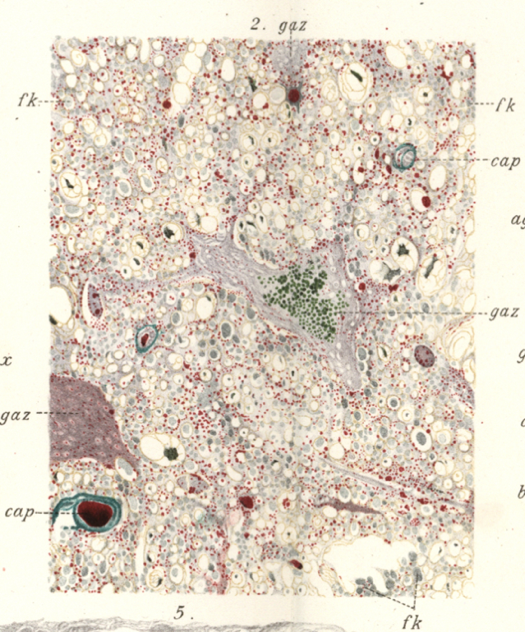
Fig. 2 shows a dorsal horn cell of a case of progressive chorea (Huntington). The majority of the green spots are filled bodies, since the axons are hardly stained and much finer.

*Table XXXV, Fig. 2 Degenerated section from the ventral horn of a case of progressive chorea. In the middle a ganglion cell with the nucleus not sliced with multiple lipoid inclusions. Piles of neurosomes are only occasionally observed. The axon and the protoplasmic processes of the ganglion cells are hardly visible. The entire tissue is dispersed with greenish, roundish bodies (filled bodies).*

Also in Fig. 3, the entire tissue is filled by such bodies.


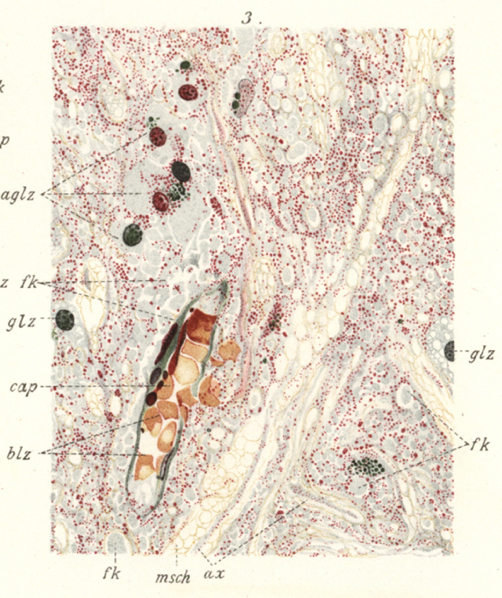
*Table XXXV, Fig. 3 From a slice through the spinal cord of a hare which had died due to a bacterial infection with ascending paralysis. Border between dorsal horn and dorsal strand. In the neighbourhood of a capillary, partially in the perivascular space, partially in the nervous tissue are multiple larger filled bodies and among them larger ameboid glial cells with fuchsinophil and lipoid granules. Filled bodies are dispersed throughout the entire tissue.*

**448**


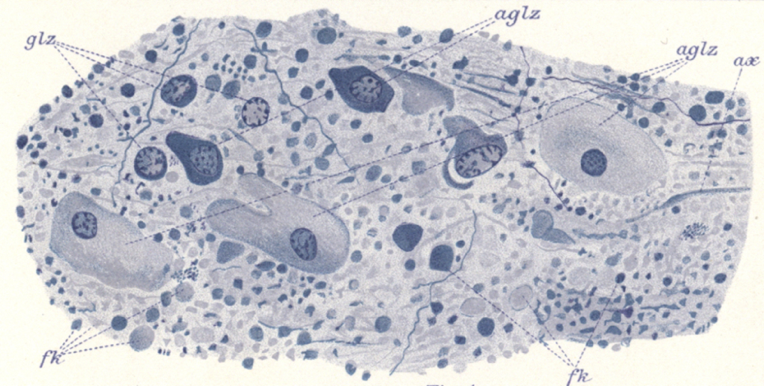
Also, Table XXIX, Fig. 1 shows the massive amount based on another staining.

*Table XXIX, Fig. 1 Section of the corpus striatum close to the ventricular surface from a case of progressive chorea. The tissue is seeded with filled bodies, besides are some ameboid glial cells, some glial nuclei with little cytoplasm, single glial fibers and axons are visible.*

It is not likely that the filled bodies are only generated where glial fibers degenerate, since we observe them also in the cortex in large amounts. In some cases of status epilepticus and in one case of delirium after infection, I have observed that they occupy the entire cortex. It is much harder to determine their generation. It is most likely that they are generated from a degradation of the reticular glia.

One should not mix up other formations with the filled bodies which can be found in the vicinity of tumors or focal diseases. They have the same homogeneous properties like the filled bodies, but occasionally reach an enormous size. They are generally found at the vicinity of vessels filing a perivascular space, sometimes also in tissue gaps which are not related to vessels. Based on their occurrence I assume that they are generated from the coagulation of pathologic tissue fluids due to the fixation process. This explains that ameboid glial cells are embedded within these formations. One could potentially assume that the filled body are generated in the same manner. I yet believe that this can be excluded for most of the filled bodies, in particular due to the detailed study of preparations as shown on Table XXIX, Fig. 20 and due to a comparative analysis of the changes in the border layer of the spinal cord as described above. Also, their entire way of deposition argues against that. One would not observe such small, copper stone-like, apposed and clearly delineated bodies, but rather large coagulations completely filling tissue gaps similar as observed in tumors. We also observed that they have similar shapes, while coagulation products show very different formations depending on the method of fixation. For few coagulation-like formations between the bodies one must leave the question open

**449**

whether they stem from the coagulation of fluid of half-fluid substances, or from a pathologic lymphatic fluid or from partially degenerated filled bodies.

It appears to me that the filled bodies have only a short live span. They lose their ability to be stained while strongly swelling and finally completely dissolve. At disease processes which have come to a halt, one does not find them. At new disease states one observes, besides the solid and clearly delineated bodies, also those which stain weakly and have lost their homogeneous feature.

*The filled bodies are important for understanding disease processes which are accompanied by the formation of ameboid glial cells. These are smaller and larger, roundish or angled, some dispersed in the nervous system, sometimes as clumps or mosaic-type composed formations. In the beginning they are intensely stained with light green, methyl blue and the Mallory hematoxylin. Later they lose their label and seem to dissolve. These observations indicate that they are largely formed by the degradation of pathologic glial structures.*

***E. The relation of the ameboid glial cells to the normal glial structures, to the pathologic fibrous glia and the pathologic glial reticulum. Permanent forms of ameboid glial cells.***

To understand the relevance of ameboid glial cells it is important to understand how they are related to normal glial structures, whether they are mainly formed from newly generated glial cells while the already existing glial structures remain, or whether they are generated by a transformation of normal glial cells either from those with delicate protoplasmic processes or from the round ones which have granules in its protoplasm. In addition, it is important how they are related to fiber-forming cells. Furthermore, it should be studied how the support system is re-established after an acute process in which the ameboid glial cells show their activity

**450**

and subsequently perish.

To resolve all these questions is impossible today since one cannot completely depict the glial reticulum. These can only be answered by complete experimental serial analysis which will clarify issues, but I have not found the time to perform this. Thus, only single observations can be reported which may address some questions, but will not show all relations as a clear picture.

It is evident that in some rapidly progressing disease events, quite a number of normal glial cells perish before the formation of ameboid cells starts. The extent of this decay in different cases needs further studies. To obtain appropriate human material is very difficult. The decay of such glial elements makes it likely that also a part of the glial reticulum is degraded and that other glial structures replace it. It is possible that with this decay, filled bodies are generated, but a proof is today not possible.

One can observe images which makes it likely that ameboid glial cells are generated both from newly formed ameboid glial cells generated by indirect cell division, from old cells with protoplasmic processes and from round cells with protoplasmic granules. The first is substantiated by the fact that multiple mitosis are observed at the first developmental stages of ameboid glial cells while at a later stage one finds only ameboid cells. In other cases, one finds hardly a significant expansion of cells prior to the formation of ameboid cells. Images like on Table XXVIII, Fig. 2b indicate that cells with protoplasmic processes transform into ameboid cells, images like on Table XXVIII, Fig. 7a indicate that that small round glial cells with protoplasmic granules do the same.


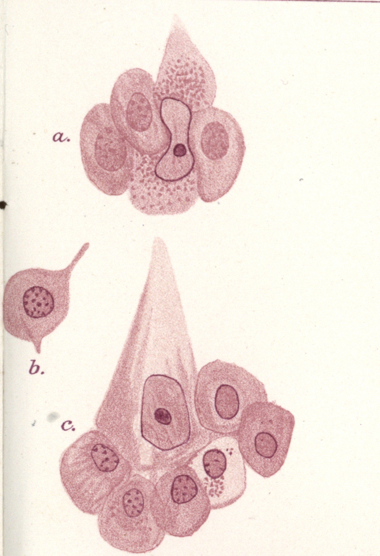

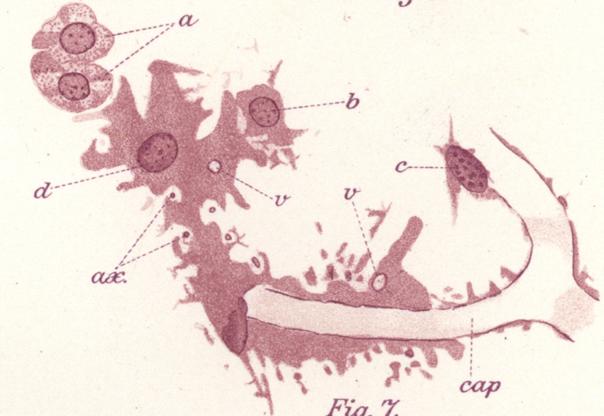


*Table XXVIII, Fig. 2b Ameboid glial cells from the cortex of a case of severe delirium after infection. b, an ameboid glial cell with two stump-like processes (maybe the rest of originally longer processes).*

*Table XXVIII, Fig. 7 a, small glial cells potentially transition forms from round glial cells with cytoplasmic granules to ameboid cells. ´fear psychosis´.*

It is difficult to determine to which extend these different cell forms contribute to the generation of ameboid cells. Sometimes, one has the impression that even glial cells with fibers in white matter swell beyond the fibers that the glial fibers degenerate while the protoplasm is

**451**

stronger stained and acquires the form of an ameboid glial cell. Thus, cells which have formed fibers might transform into ameboid cells. Eisath indicated to me that in preparations stained with his method, nicely labelling protoplasmic glial structures (as shown in Table XXVIII, Fig. 1a and b), one never finds glial elements with protoplasmic, multiple processes together with ameboid glial cells in white and grey matter. In the normal brain, they can be stained easily.


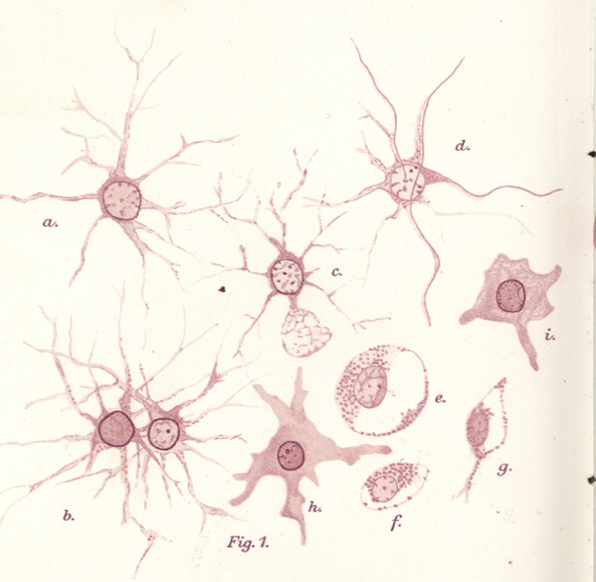
*Table XXVIII, Fig. 1 a, b Normal glial cells with protoplasmic branches from the cortex of a 34-year-old man who died due to an accident*.

Also, my observations confirm this in many cases. This indicates that with the appearance of ameboid glial cells the normal glial structures undergo substantial changes. Instead of multi-branched glial cells one observes, with the same staining technique, those with little increased protoplasm without processes, or those with an enlarged cell body and granular substance. This observation is important. Yet, I observed with the Golgi method multiple branched cells in the same preparations in which I did not find ramified cells with the Mallory hematoxylin stain. Thus, I want to be cautious and will not claim that the processes degenerate or are retracted by the cells. Maybe they do not show the same affinity to the Mallory hematoxylin stain.

It is remarkable that with the expansion of the ameboid cells, the fibrous glia can degenerate to a large extend. If one can no longer display glial fibers in some cortical or spinal cord regions, it may not due to a failure of the Weigert method but rather to the fact that the fibrous glia has degenerated to a larger or lesser extent. I have for a long time opposed this view and would recommend that one should not draw the conclusion that fibers have degenerated, if one observes a reduction in fibers. It seems a common feature in certain disease states and can be clearly observed.

**452**

With the discussion of the filled bodies, I have provided evidence that these are not postmortem alterations. The normal support system of the nervous system undergoes fundamental changes in acute disease states which leave little of its original structure.
Depending on the type of disease, we find quite a different relationship between ameboid and newly formed fibrous glia. In paralysis, for instance, both forms develop in parallel. Besides glial cells with a massive cytoplasm from which bundles of thick glial fibers extend, there are giant ameboid glial cells in all stages of development and disintegration. With more rapid progression of the disease process, the ameboid cells predominate over the fiber-forming. We observe similar issues at acute lesions and at acute syphilis processes, in particular at the terminal arteries (18) and also at progressive senile dementia. At other acute disease processes, including acute dementia praecox, we observe besides the ameboid cells no fiber formation. In chronic cases it is rarely significant or even absent. In genuine epilepsy, it is restricted to the surface layer or the white matter. In alcoholism and progressive chorea, it remains negligible. The same applies to many forms of cretinism. These obvious differences may be due to fundamental variations in the disease processes which we do not yet understand. In general, it can be stated that the degree of enlargement of the glial fiber border layer in different diseases correlates with the deficits in the cortex. In some individual cases, particularly in some cases of cretinism, this may not apply. The formation of glial fibers in the cortex may not depend on the amount of degenerating nervous tissue, but may depend on other processes such as vessel expansion or infiltration of lymphatic tissue and so on.
How are the conditions of the delicate glial structures at diseases which have progressed from an acute state

**453**

with formations of ameboid cells into a chronic or stationary state with no or only little formation of glial fibers? Sometimes the original state is reconstituted. One can observe images of glial cells close to the normal tissue, with some anomalies. Yet this needs to be studied more closely; now it is still very difficult to analyze these different findings. I will focus on some single observations which seem to be clarified now and which have a general pathologic relevance.
We have already observed that the fatty cysts which are formed in ameboid cells also occur in fiber-
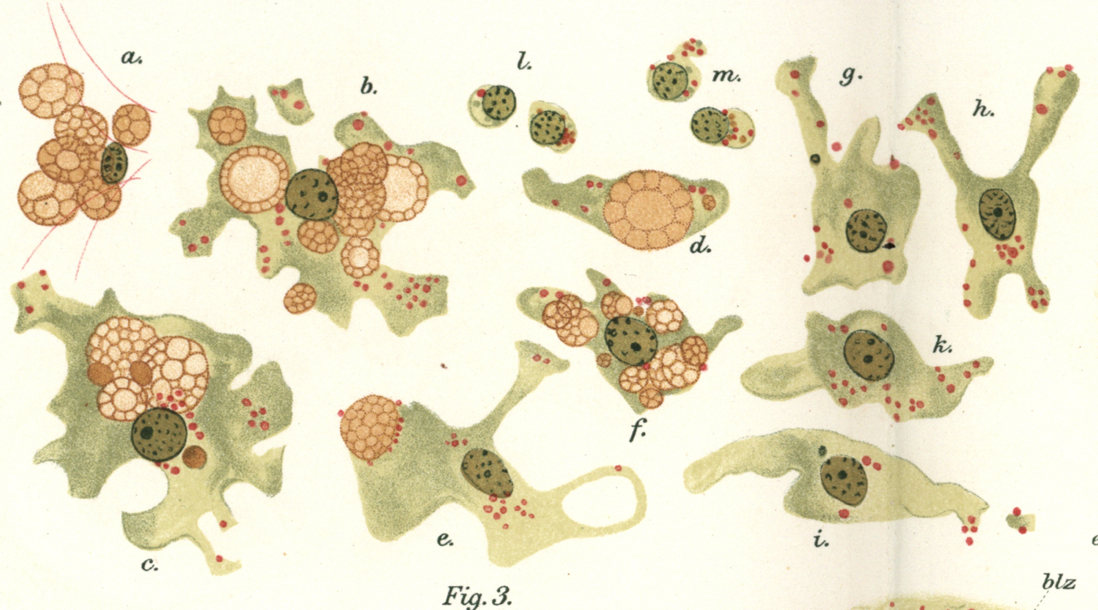
forming cells.

*Table XXX, Fig. 3a Ameboid glial cells from the white matter of different cases of status epilepticus and peculiar psychosis of the degenerated age (15). Glial fiber forming cell without clear cytoplasm filled with lipoid cysts.*

In cases in which the entire cell body is not converted into cysts, we observe fuchsinophil granules in the cells. Such cells would serve dual tasks, forming fibers and serving as ameboid cells. The large fiber-forming glial cells lack lipoid cysts, but we observe solid fatty granules, even coarse fatty spheres. The fuchsinophil granules which we find within these cells are distinct from those of the ameboid cells.


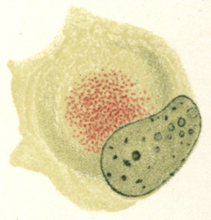

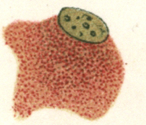
*Table XXX, Fig. 5 Large cells belonging to the type of fiber forming glia with a pile of dust-like fuchsinophil granules in the center of the cytoplasm. Progressive paralysis.*

*Table XXX, Fig. 6 Similarly, a cell with complete replacement of the cytoplasm by delicate fuchsinophil granules.*

Table XXX, Fig. 5 and 6 show such cells from a case of paralysis after staining with acidic fuchsin light green. In the center of the cell body, which is less intensely stained in Nissl preparations and often shows a greasy gloss, there is a cloud of densely packed, dust-like, delicate, red granules which appear minute compared to the fuchsinophil granules of the ameboid glial cells. With increasing age of the cells, they expand until the entire cell body is filled and one can hardly observe any cytoplasm. The tendency of these granules to convert into lipoid substances is obviously low. Only now and then, one detects some blackened granules in between. I have also never observed granules stained with methylene blue in these large fiber-forming cells. Based on these variations in the structural features, one may assume important differences in the functions of these different elements.

**454**

As we have observed, the lifetime of these ameboid cells is rather limited. Individual ones can, under certain conditions, convert into permanent forms. Such permanent forms can again be further differentiated. On Table XXXIII, Fig. 13 is such a form depicted which is not rare.

.
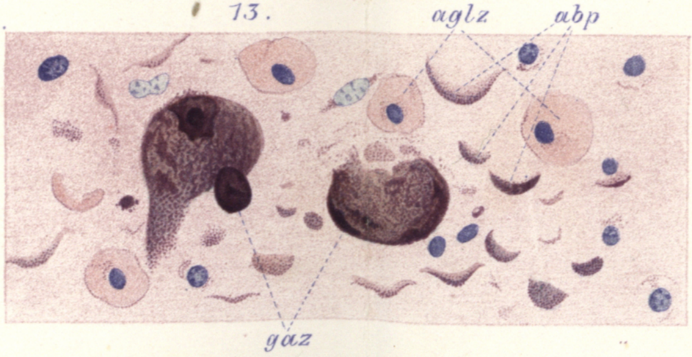


*Table XXXIII, Fig. 13 Section from the nucleus of the regio subthalmacica of a case of progressive chorea. Two ganglion cells in which basophil products dissolve, in between permanent forms of ameboid glial cells and basophil degeneration products which show a peculiar ship-like form.*


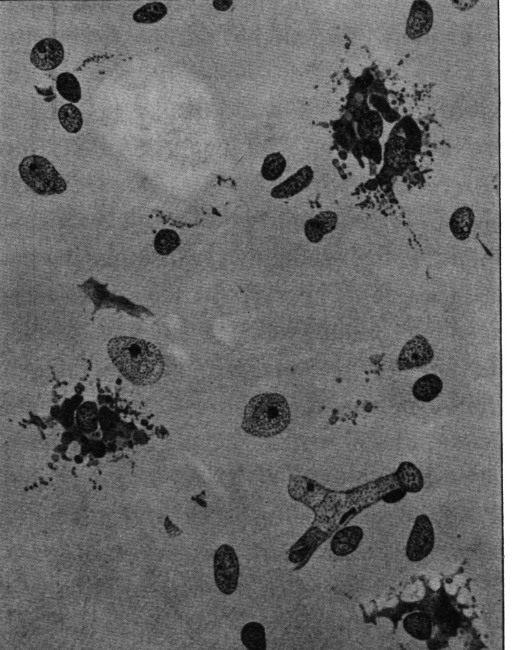
It was obtained from a section stained with toluidine blue after alcohol fixation. The nucleus is dark and homogeneously labelled, the cytoplasm is weakly stained and it has a gloss which cannot be reproduced in the drawing. It indicates that it represents a modified cytoplasm. Obvious is the sharply delineated rim of the cell body in this form.
In other forms, the relation to ameboid cells is not as obvious but is likely based on their shape. The figure 1 in the text shows such cells. They are from the cortex of a case of cretinism with epilepsy stained with the Weigert method.

*Text Fig. 1 Peculiar permanent forms of ameboid glial cells from the cortex of a case of cretinism with epilepsy. Weigert´s glia method.*

The nucleus seems to be positioned freely, and around it are plates, ramified slabs, and single pieces of a substance intensely stained with methyl violet. Occasionally, one finds single particles far off from the blue accumulated mass in the tissue. The entire picture reminds one of an ameboid cell, yet the substance of the cell body is altered and seems to be dissolved from the nucleus.

**455**

It seems to be decomposed into larger pieces around the nucleus and into smaller ones at the processes and seems not to be interconnected. Since the nucleus does not exhibit regressive alterations, it may not be considered that it has dissolved from the cytoplasm. It is likely that it is connected via unstained cytoplasm with the methyl violet stained slabs. Since we have observed other glial cell forms in which the cytoplasm shows the tendency to decompose into pieces (filled bodies), it is therefore not unusual. Remarkable are the strange staining reactions of the pieces and slabs. Their staining properties may indicate that they are related to the Weigert´s glial fibers. Then we would have a case in which glial cells at the edge of their cell body shed off slab-like forms of the same substance instead of glial fibers. Yet the staining reaction is not proof for this assumption. One can stain those slabs also very well if one heats frozen sections from the glia stain in aqueous hematoxylin and subsequently de-stains with Borax-iron-cyan potassium solution. Then no glial fibers are labelled. This also does not indicate that they are of another chemical substance than the glial fibers, since they are both stained in an undifferentiated preparation and the lack of de-staining of the slabs may be due to their larger mass. Also, the fact that they are strongly light-scattering in the unstained condition does not argue against it.
Additionally, the new formation of the glial reticulum may, under certain circumstances, occur with the participation of cells related to ameboid glial cells. Table XXXV, Fig. 6 shows a section of a strongly atrophic striatum from a case of progressive chorea, in which all ganglion cells are strongly degenerated.

**456**


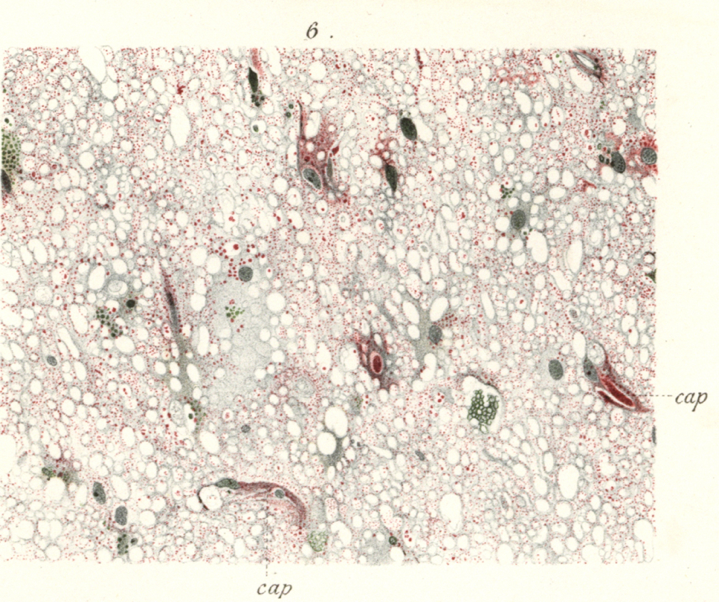
*Table XXXV, Fig. 6 Cut through a degenerated corpus striatum of a case of progressive chorea. A delicate glial reticulum formed by a type of ameboid cells penetrates through the entire tissue.*

There is no trace of fibrous glia. The support system is formed by peculiar, protoplasmic glial cells which show a similarity to ameboid glial cells, in particular due to the fact that they contain accumulations of fuchsinophil granules and small cysts. Around the individual glial nuclei is a delicately labelled cell body obviously of modified cytoplasm, which contains multiple holes at a distance from the nucleus and which diversifies into many processes that seem to invade into a reticulum of the same substance related to other nuclei. It penetrates the entire tissue.

One cannot prove from such preparations that all cell borders are waved. At least it shows that complex and obviously permanent glial structures form as a scar formation without fiber formation under participation of cells reminiscent of ameboid glial cells.
Very similar images I have obtained from a white matter region of a young case of dementia praecox. Besides ameboid cells which seem to divide at their borders by insertion of multiple gaps and dissolve into a reticulum, there were net-type cytoplasmic connections in large parts of the white matter tissue which seemed to be generated by the same type of cells.
I have obtained interesting images in the neighborhood of bloody lesion areas which were several weeks old using our methods, which may shed light on the reconstitution of net-type glial structures. In regions of white matter which were not directly damaged but which showed deteriorated axons due to secondary degeneration, by use of Mann’s staining, there were large glial cells in large numbers, some forming a lot of fibers and others indistinguishable from true ameboid glial cells at all developmental stages. Other glial cells with a large and slabby protoplasm with a larger nucleus were characterized by a cell body which diverted at its edge into a distinctly stained delineated reticulum containing in its mesh normal and degenerated axons. Text figure 2 shows such a cell. At some regions, the cell body

**457**

seemed to have retracted from the reticulum, giving the impression that the cell lay in its own formed nest.


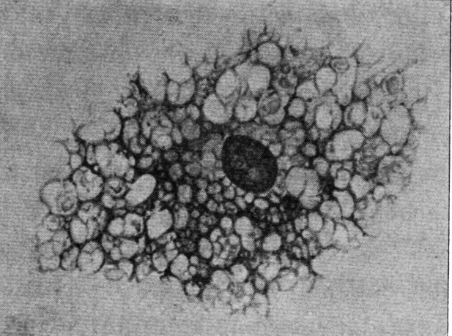
*Text Fig. 2. Glial cell in the neighborhood of a 14 day old bloody lesion in the white matter; axons pass through the reticulum, Method V.*

In this disease focus one finds all forms of glia next to each other: the fiber-generating, degenerating, and elements building the reticulum. In areas where several net-forming cells are adjacent, the nets seemed to fuse. Such nets were visible only around nuclei, while in large areas in between, one could not observe a reticulum. I assume that I have seen similar processes in other diffuse disease states. In the vicinity of the disease focus, the size of the elements defines them. In these diffuse disease foci, the structures are very delicate, hardly stained, and undetermined, and one does not feel to be on safe ground. Therefore, the methods should first be improved before one can make solid conclusions. But already these fragmentary observations indicate that the glial structures are very complex and distinct in different diseases, illustrating also the high variability of neuronal damage where glial cells are the complementary negative forms.
Finally, I would like to make some remarks about regenerative processes which can be observed in perivascular spaces. These processes are very difficult to judge, and I will restrict myself to some observations which will not allow reconstruction of the entire process. In cases of psychosis which were in a chronic state, e.g., dementia praecox, I have often observed that strange, meshwork-like formations of a type of ameboid cells surround vessels, which are partially in the nervous tissue and partially free in the perivascular space.

**458**

One often observes that protoplasmic glial processes bridge the perivascular space and attach to the adventitia, replacing earlier bands. One gets the impression that closer connections between glia and adventitia are being formed.
In other regions, one finds a mass strongly stained with S-fuchsin filling more or less the perivascular space. Since the adventitia is strongly labelled green and can be clearly recognized and distinguished, it is thus not a connective tissue mass. It seems to be a glial product, maybe a thickened membrana perivascularis.
Moreover, one observes in preparations from chronic cases a loosening of the tissue in the vicinity of vessels. These may have been caused by defects in circulation or lymphatic congestion that may have occurred earlier at this location. The same process may result in regions around vessels with only a few nuclei, which is often found in chronic cases.
I have already discussed that the membrana superficialis gliae is dissolved in response to common or local degeneration of glial structures and that this results in the progression of fibrous glia beyond the original surface layers.

***F. The biological importance of ameboid glial cells, the conditions of their appearance and their relation to the degeneration of the nervous tissue.***

The condition of their appearance may indicate the function of ameboid glial cells. One does not find ameboid glial cells in the adult, normal brain. Similar cellular forms can be found in the fetal nervous system when the system is built up. We also do not observe them in past or halted disease processes except for the permanent form which I have discussed above.

We do not find them in old encephalitic scars, in the vicinity of encapsulated bleeding and degenerative foci, in most cases of cretinism, in old cases of dementia praecox (11) without a recent boost;

**459**

in epilepsy with longer pauses of seizures, a halt or very slow progression of the disease, in alcoholism with a longer period of abstinence before death; in stationary paralysis. Also, in some cases of progressive paralysis and senile dementia they are less abundant. In several manic-depressive patients who died during extreme excitement, I have not found them; in some others, they were occasionally found. Yet the case was blurred by additional severe non-CNS comorbidities. The typical ameboid glial cells are often not or only sparsely observed at extensive degeneration of nervous tissue in encephalitic cores, in foci and bleeding, in strand degeneration of the spinal cord, in meningitis infiltration, in the cores of rapid degeneration of myelin as often found in epileptiform seizures of paralysis, thus in cases where a massive formation of ectodermal or mesodermal granule cells occurs.

We find them in severe deliria related to infections, in alcohol delirium, in many cases of status epilepticus and in some chronic epileptic conditions, in paralysis, in senile dementia, in lues cerebri, in the three latter depending on rapid or slow progression of the disease, in progressive chorea, in many acute or subacute experimental intoxications, in the neighborhood of tumors, occasionally next to granule cells at the edge of acute foci and abscess. Truly gigantic forms were found in a case of sclerosis due to tuberculosis. They were occasionally found in severe cases of uremia, in diabetic coma, in sepsis, and in several cases where a several-days-long complete unconscious state occurred before death without any mental or psychiatric symptoms. Yet in all these cases they are related to severe infectious or toxic final mental disturbances. They are not dealt with in the textbooks of psychiatry, since they have no significant psychiatric relevance.

**460**

It is hardly possible to list all disease states in which they occur, since some do not yet have a name. Yet this listing shows that they are not characteristic for a defined disease state but accompany many different acute mental disturbances and acute states of chronic psychosis which lead to mental symptoms. From an anatomical perspective, they are related to nervous tissue degeneration. Single observations that seem to be exceptions need further analysis.
The finding that ameboid glial cells are occasionally lacking or sparse in some disease states with a very rapid course may be due to the fact that it takes some time for their complete formation and that first, other glial elements have to degenerate or be newly formed by indirect division. Death can occur before they are fully developed. In some cases of status epilepticus, heavy intoxications or infections, this may be the case. How rapidly ameboid glial cells appear and disappear can be judged in status epilepticus of genuine epilepsy. In the brain of a genuine epileptic patient who has no subsequent seizures or states of deterioration, we find no ameboid cells or only very sparse ones. After a 12-hour status epilepticus, we find them well developed, some even next to multiple cell divisions (karyokinesis). In a status that has led to death after 6 hours, they are completely absent, but karyokineses are abundant. In a case in which the status continued for 27 hours after a 6-hour interruption, many degenerated ameboid cells with methylene blue granules were observed, which were rare in the previously described cases. We cannot exclude that their formation started before the first seizure of the status epilepticus, as in some cases where regressive altered ameboid glial cells are found. Yet it is highly likely that they are formed within a few hours after onset. In contrast, in experimental intoxications

**461**

based on Nissl’s subacute maximal intoxication, it will take a few weeks until the ameboid cells appear. Early glial alterations are found; however, severe damage to the nervous tissue is required before the development of ameboid glia occurs.
Based on the comparison of clinical and anatomical data, it appears that the appearance of ameboid glial cells is related to the degradation of nervous tissue, yet it still remains to be determined how these events are related. In particular, the degradation of the nervous tissue requires histologic proof. This is yet a task which cannot yet be sufficiently resolved based on our present methodologies. Despite this, I believe that we can recognize some issues related to the degradation of nervous tissue based on the methods currently used.
It is noteworthy that in many disease states one finds the majority and the largest of the ameboid cells in the white matter, particularly in the medullary crest of the cerebral gyri. So far, one has assumed that disease conditions resulting in psychosis occur predominantly in the cortex and that the white matter is only affected due to a loss of axons in response to the degeneration of cortical elements. The appearance of many ameboid glial cells in the white matter is difficult to explain. If we analyze even more severe degeneration processes in which the glia generates typical fatty granular cells as often found in paralysis after apoplectic seizures, we even find more of these fatty granular cells in the white matter as compared to the cortex. It may be due to the fact that the degenerating white matter generates more fatty products since it is built up by lipoid substances. A contribution to the accumulation of degeneration products in white matter may be due to the fact that the meshwork of capillaries is much denser in the cortex compared to white matter, which promotes their removal in the former. Moreover, the degradation process in the cortex seems to be different from the one in

**462**


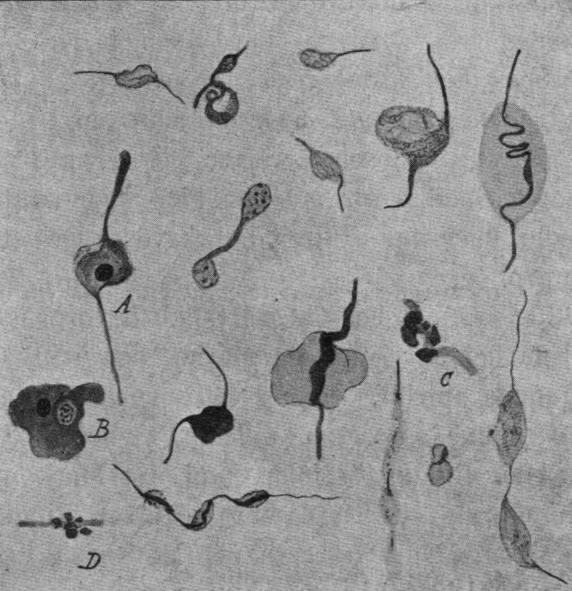
white matter in so far that there could be dissolvement processes being dominant at the protoplasmic structures. When ameboid cells are found in larger amounts in the white matter, in most cases, one can find degradation processes in the nervous tissue proper, in the axons, and in the myelin. Screening a larger set of material creates the impression that the formation of ameboid glial cells can start before degradation processes can be verified, yet they are rarely missing where many and partially regressive ameboid cells are found in the tissue. Yet sometimes ameboid glial cells in all stages of development can be found without observing a degeneration of axons. For the detection of degenerating axons, method V can be used.

*Text Fig. 3 Delicate degenerating axon from the cortex of a case of dementia praecox (11) and status epilepticus illustrated with method V. A, an ameboid glial cell lays in the swollen Axon. B, a remnant of an axon enclosed in an ameboid cell. C and D white matter fragments.*

If one follows the instructions, the normal axons are stained blue. It may occur that a smaller or larger number of axons are stained in a reddish-blue mixed color. These reddish-blue axons, like the blue ones, show no morphological alterations. Yet certain axons, usually the thinner ones, are obvious due to their shining light-red color. These show in their form and structure obvious alterations

**463**

compared to normal (text figure 3). The normal axons are bordered by straight lines and of homogeneous structure and staining. The red ones show a variable thickness, large swellings, a snake-like course, a granular structure, even a granular disintegration. With respect to their structural features, they remind one of images which one obtains from degrading axons with the hematoxylin staining according to Donaggio. As shown in comparative preparations, they correspond to the images of Donaggio. Since it corresponds only to few and very thin axons, the method of Donaggio is not suited to label them selectively. If the larger, normal fibers are de-stained with this method, these degenerated thin fibers are also de-stained. Significantly, yet less brilliantly, one can observe such degenerated fibers also in Flemming’s slices, which are stained in a 1% acidic fuchsin solution after embedding in photoxylin for 5 min. The normal axons are dull light red, while the degenerated ones are shining dark red. The morphological alterations are similar as in the Mann’s preparations.
Besides the red-labelled axons using method V, one can find others which are pathologically altered. The staining is dark blue or violet, much darker than the normal axons. The axons seem to be dissolved as slabs or granules of dark blue color (Table XXIX, Fig. 20). In general, such axons are rare.


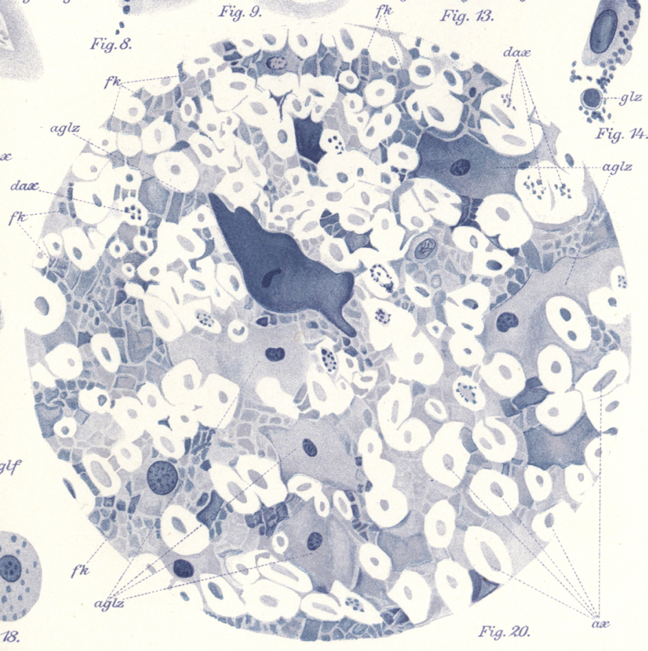
*Table XXIX, Fig. 20 Slice from the white matter of the spinal cord from a case of progressive chorea.*

In severe cases, they can outnumber the red ones or even be the only ones present. It seems, as if these red and dark blue axons do not reflect different stages of degeneration, but rather two distinct forms of degeneration, since one finds sometimes the one or the other form. Occasionally, one observes, with the same method, axons which are heavily swollen and stained light blue. In particular in the neighborhood of lesions and in meningitis infiltrations in the spinal cord, they can be observed. Treating cortical tissue, in which many red axons could be labelled with the Mann’s method,

**464**

with the Bielschowsky silver method, one can find, besides smooth and homogeneous axons, also those with an irregular course, partially swollen, consisting of granular mass or seeming to be in granular disintegration. Using different methods, one finds that the formation of multiple ameboid glial cells is associated with the degeneration of axons.
Much more difficult is the identification of degenerative processes in the white matter of the central nervous system. The generation of artefacts is favored by the fact that the white matter has the tendency to undergo post-mortem changes due to the difficulty of fixation and due to the easy extraction of white matter components. One has to select and treat the material with great care and critically evaluate the results. Otherwise, one comes to incorrect interpretations due to artefacts.
Using method IV, parts of the white matter of the central nervous system are red-labelled. In between, one observes clumps of clearly delineated, large, strongly red-labelled slabs in the white matter; they are of irregular form and are often arranged in rings around an axon, which does not exhibit a morphological alteration (text figure 3 C, D).
Also, with the Marchi method one can recognize pathologic changes in the white matter, if one acts with care. For such delicate investigations, one should not use material which has been embedded in formalin; the longer the brain sections are embedded in formalin, the more appear brownish or black products in the tissue, if later treated with osmium after a previous chromium exposure. They are distinct in form and position from the pathologic white matter products, yet make it difficult to recognize the latter or make it even impossible with larger accumulation. Also, in non-fresh material or material damaged by finger pressure or rinsed with water, artefacts are generated with the Marchi treatment in the form of brownish or black slabs. In cortical sections, freshly taken from the dead body, directly transferred to Müller solution

**465**

and treated according to Marchi, one finds occasionally disintegration of the white matter at areas where multiple ameboid glial cells are observed. It reminds one of Wallerian degeneration in the form of black-brown clumps arranged in rows, often with a more or less abundant accumulation of brownish or blackened round formations. They resemble the bodies of the peripheral nerves described by Elsholz. At other white matter boundaries, there are single or accumulated dark-labelled slabs appearing against the unlabeled background. These are images that have often been found at a low degree of discontinuous degradation of peripheral nerve fibers (in toxic and infectious neuritis).
Another occurrence that requires attention, yet is not sufficiently explained, is the appearance of peculiar, green-labelled, often loop- or honeycomb-like formations in the white matter boundary using acidic fuchsin light green staining. These are frequently found at locations where white matter disintegrates, e.g., in the neighborhood of foci and scattered everywhere where ameboid cells occur, and very rarely or actually never in the normal brain. They should have a pathological relevance.
In these investigations one should note that one hardly finds a human brain without any single black bodies or brown slabs in the white matter. It is likely that they are related to degenerative processes which are within the range of normality. One also has to be careful not to mix up fatty substances enclosed in glial cells with actual degradation products of white matter. The already mentioned cyst-like formations and accumulated arrangements of lipoid substances indicate that they are embedded in glial cells. To avoid a mix-up, it is necessary to make thin slices and add a staining with saffranin and acidic fuchsin light green to the Marchi treatment. Only then can one identify axons, cell nuclei, and protoplasm and thus determine the location of the black or brown substances. Indications that need to be followed up suggest that even other alterations occur in the white matter;

**466**

yet, those already described, sufficiently show that the appearance of ameboid glial cells coincides with degenerative processes in the nervous system.
How does the appearance of the ameboid glial cells relate to the disintegration of the nervous tissue?
A space-filling role is certainly not the issue since the ameboid glial cells appear before the nervous system has deteriorated. Their short lifetime and their tendency to rapid decay speak against such a biological function. They must have another relevance.
As one observes how the ameboid glial cells surround the white matter region until they completely enclose it, one could assume that they participate in the destruction of the nervous elements, thus fulfilling a neurophagocytic function. The last years have provided significant literature on neurophagocytosis, and there are different views on the type and origin of the cells which develop neurophagocytic activity and how the neurophagocytic activity operates. The neurophagocytes could either dissolve the nervous cells or incorporate parts. They could attack living cells or only destroy those which have already died.
In these transformations which we will now discuss, only neurophagocytes of glial origin will be relevant. The neurophagocytic activity does not correspond to ganglion cells but to myelin and axons. That glial cells incorporate foreign material is well known. In the neighborhood of previous regions of blood infiltration they are loaded with blood pigments; they collect ink, carmine, vermillion after injection into nervous tissue within their cell body, eventually becoming round formations fully filled with foreign material.
The observation that glial cells surround myelin sheaths with their protoplasmic cell body does not necessarily correspond to a neurophagocytic activity. If glial cells positioned between myelinated axons enlarge, they can only move into the narrow spaces between the myelin sheaths, and a simple enlargement of their cell body could generate such images.

**467**

Severe degeneration processes as those so far described acquire fatty granular cells and clearly demonstrate that the fat which accumulates in the glial-type granule cells originates from the white matter and products of white matter. Thus, glial cells degrade white matter. This process can be analyzed very clearly in acute degenerative foci, for instance, in the spinal cord in meningomyelitis due to tuberculosis. Fig. 6 of Table XXXIV shows this case treated with the Marchi and the Herxheimer method.


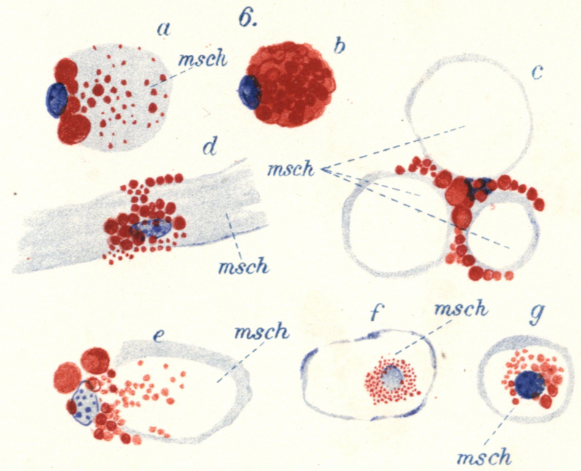


*Table XXXIV, Fig. 6 Different developmental stages of glial granule cells in the spinal cord. Meningitis with tuberculosis. f, g small, fat-loaded glial cells within the stil existing white matter tract. a, c, d, e beginning accumulation of cells with fatty granules. b developed fatty granule cell.*

The Marchi method shows many brown and black white matter slabs. The Herxheimer method illustrates that quite a number of small glial cells infiltrate into the myelin sheath and store finer and coarser fatty granules in their cytoplasm (f, g). They then degrade rapidly after their nucleus has acquired a homogeneous staining and finally become unstained. One could assume that these elements could be blood elements based on their small size and roundish cell body. Yet control preparations demonstrate that at these locations there was no emigration of blood elements. These seemed to be only small early combats. The actual degeneration process starts with an accumulation of multiple fat droplets in the larger glial cells which are in between the myelin sheaths (a, d, e, c) which convert into true fatty granular spheres (b). Using Marchi preparations, one can find that the glial cells which had accumulated such fatty granules surround slabs and pieces of white matter which are not stained red by the Herxheimer method. A comparison of many such images shows without doubt that with an increase of the fatty granules in the cell body of the glial cells, the enclosed white matter pieces become smaller and paler until they disappear. In any case, the glial elements convert the different substances from which the myelin sheath is built up and which with the disintegration of the myelin sheath accumulate as pieces (white matter slabs) into fat. The white matter slabs are stained brown with a chrome reaction with osmium and are not labeled by Scharlach in formaldehyde slices. Due to this feature, it shows that

**468**

they are converted substances but not yet transformed into fat. They appear in the cells in another form, as granules which are stained black with osmium and red with Scharlach. There is no indication that the glial cells initiate their degradation activity on normal myelin sheaths and damage them. This indicates that the cells only remove substances that are already damaged due to the disease process and are destined for decay. The glial cells certainly do not enlarge because there is additional space due to the decaying nerve fibers. In contrast, the degenerating nerve fibers occupy more space than the healthy ones. They enlarge in response to the stimulus generated by the pathological products of the myelin sheath. Occasionally, we observe that an individual white matter piece is entirely enwrapped by a glial cell, thus embedded within it. We do not observe that they take up small particles into their cytoplasm. They seem to act in a degrading fashion on these products. They seem to assimilate the substances arising from the dissolution and convert them within their body into fatty substances. Nowhere do we find free fat in such tissue. Fat is generated within the glial cell. The similar size of the fat granules within a given cell hints at their intracellular generation. The degeneration continues by a transfer of the fatty substances from the nervous system into the adventitial lymphatic spaces. In this particular form of nervous system degeneration, the activity of the glial cells can be well studied and sheds light on disease states of the nervous system in which ameboid glial cells play a role.

In regions where ameboid glial cells, and not fatty granular cells, dominate, one can occasionally find shiny, red-labeled remnants of an axon in a space within a blue-labeled ameboid glial cell (text figure 3B). Yet, images showing that the ameboid glial cells completely take up parts of disintegrated nervous tissue are rare. More often, one can observe that ameboid glial cells attach to pathologically altered myelin and axons (text figure 3A). Often, one does not find an ameboid glial cell at a disintegrating axon, or

**469**

at locations where ameboid cells are present in large numbers, no remnants of a degenerated axon are found. This may not indicate that the ameboid glial cells attack living and healthy axons. It may be that substances they release contribute to the dissolution of the degenerating nervous material. They may assimilate products released from the dissolved nerve material that have been incorporated into the extracellular fluid and subsequently degrade it. Substances arising from the degradation of the nervous tissue may trigger the formation of the ameboid cells.

This may not explain the rare cases in which one finds ameboid cells without obvious nervous system degradation. Maybe in these cases, nervous structures degrade which we cannot yet illustrate, or perhaps pathological products are generated which cannot yet be detected with the microscope—not by a degradation of nervous elements, but due to a disturbance of their consumption—and they aid in their removal. I have spent a lot of time clarifying this question and have not yet reached a final conclusion.

Nevertheless, the granules that we observe to appear during their life cycle are not taken up by the cells but generated by them. The first form of granules which we can easily detect are the fuchsinophil granules. In particularly large ameboid cells, for instance in hypertrophic sclerosis, one can identify besides the fuchsinophil also granules stained with light green. My experience with these is yet insufficient to provide many details. The light green granules should not be mixed up with certain lipoid substances which are lightly stained in brown with osmium but also contain some light green. The true light green granules are similar in form and size to the fuchsinophil granules and one observes only a few within a given cell. They do not seem to be lipoid substances.

**470**

The fuchsinophil granules belong to the ameboid glial cell and are regularly found inside it. Thus, they are related to their regular function. They are present in early stages and appear earlier than the brown or black substances.

In the white matter, there are sparse and differently formed fuchsinophil bodies. These refer to the more rod-type fuchsinophil bodies of the axons and very small granules in the cytoplasm of the glial cells, maybe also in the glial reticulum. Large, round granules as found in the ameboid cells are not found in the normal white matter. They are also not found outside the cells in pathology. Thus, one can conclude that they are generated within the cells.

Their staining properties do not give any hint of their chemical composition. In preparations where they are found, many other structures show the same red color, such as red blood cells, glial fibers, and other granules which are normally present and belong to the ganglion cells. Also in the hematoxylin staining mentioned above, the granules of the ameboid cells label similarly to those which are normally present. This applies also for the Held method. We know since the studies of Altmann and his students that fuchsinophil granules occur in many cells, and Heidenhain’s recent studies show that their relevance is still speculative.

Several observations in our preparations indicate that the fuchsinophil granules of the ameboid glial cells represent precursor products of lipoid substances. Fuchsinophil granules accumulate mainly at locations where osmium-stained black or brown substances appear. Not rarely, we observe individual granules that have acquired a brown label but still retain their red label. Sometimes the red, sometimes the brown color is dominant. The large lipoid cysts seem to evolve from small brown bodies which at first are of the size of the fuchsinophil granules. Thus, we may follow the transition of the fuchsinophil cellular granules into lipoid substances.

**471**

It is thus certain that the fat which we find within the ameboid cells is not simply taken up. It is a remarkable fact that we almost never find lipoid substances stained with Scharlach outside of cells in the nervous system, except where it is completely destroyed. They are all within cells. At locations where they seem to be outside, a closer inspection indicates that they are within cellular processes which had been cut off from the cell body during the slicing process. Whether this applies also to the substances that are brown or black stained by osmium—particularly the slabs which occur in the degeneration of the myelin sheath and do not stain with Scharlach—is less certain. In different cases, it is hard to decide whether such a slab still belongs to the white matter structure or is already isolated. In preparations of status epilepticus, I have often observed ameboid cells with fatty cysts at locations where no brown or black slabs were present. We thus conclude that the lipoid substances of the ameboid cells are generated by the activity of the cells themselves through a conversion of fuchsinophil granules. These substances accumulate significantly within the cell and almost entirely replace the cytoplasm, indicating that there is a tremendous buildup of lipoid substances. They can remain longer there or also gradually be released.

As we have seen, the formation of fuchsinophil granules and fatty cysts seems to be a milder form of degeneration. Dramatic degenerative processes are indicated by the formation of granules strongly stained with methyl blue and phosphor molybdenum hematoxylin. With the formation of fuchsinophil granules and fatty cysts, the cellular protoplasm is partially maintained and the nucleus undergoes no regressive changes, or only at later stages. With the appearance of methyl blue granules, the cell body and nucleus dissolve.

**472**

What kind of substances form these massively generated methyl blue granules, we do not know. They do not occur in ganglion cells or in mesodermal cells of the cortex and are not related to fuchsinophil granules, nor to known lipoid substances or stainable fatty acids. They are not taken up by cells but rather generated within the cells, as is evident from the observation that they are of similar size within a given cell and of variable size among different cells. In cells in which methylene blue granules are present, we also observe a few small brown or black granules and sometimes a few tenuous fuchsinophil granules. They are neither precursors nor transition products. The methyl blue granules are stained fainter while they increase in size and finally become unstained. They seem to swell and liquify along with the rest of the cell. The fibrinoid granules behave similarly to the methyl blue ones, and we also do not know anything about their chemical properties.

At any region where methyl blue granules appear in the tissue or around vessels, there are glial cells below, in the perivascular space. I mentioned earlier that these perivascular spaces are not shrinkage spaces. They are rather formed due to a decay of ameboid cells attached to the membrana limitans after their protoplasm has been used up by the formation of these granules.

To clearly understand the processes which occur around the vessels, it is essential to know whether glial cells have the capacity to migrate. Many authors are convinced that they can migrate. For some types of glial cells, this may not be possible due to their complex morphology. The roundish ameboid forms could translocate based on their morphology. But it is difficult to decide and cannot definitely be proven. By following the translocation of substances injected into the nervous system, it cannot be excluded that particles in distant glial cells were transported by the lymphatic flow. Glial cells can also rapidly proliferate at a location and their increased number does not necessarily have to result from migration. As mentioned above, cells which can be assumed to be glia are found within degenerating myelin sheaths. They must have invaded there.

**473**

Also, single ameboid cells from the nervous tissue could transit into the perivascular space. Through gaps of the membrana perivascularis, other substances such as the filled bodies could transit into the perivascular space. Some of the products which we find there could have passed the intact border membrane in a dissolved form and aggregated beyond it by our fixation procedure. We have seen earlier that the mass of all these products which deposit in the perivascular lymphatic spaces can be enormous. Where they are quite abundant, the perivascular space is widened, also when using fixation materials which do not cause much shrinkage. It is difficult to decide whether the tissue in the neighborhood is edematous and shrinks despite careful fixation or whether lymphatic fluid builds up in the perivascular spaces filled with pathological products. It cannot be determined with certainty using histological methods whether lymphatic fluid can build up in the nervous tissue. We always have to consider artificially shrunken spaces when we observe gaps in the tissue. That lymphatic fluid can build up is beyond doubt. We occasionally observe that the adventitial lymphatic space is tremendously enlarged and pervaded by meshed connective tissue with incorporated clearing cells. Occasionally we observe such cells beyond the adventitial border in the perivascular space. In nervous tissue surrounding vessels, one finds openings and gaps, in particular in thick, diffusely labeled slices. These are very unlikely to be artificially shrunken spaces based on their arrangement in relation to the course of the vessels.

Obviously, all products of the perivascular spaces are dissolved. They may even be transferred already in a dissolved or semi-dissolved form. Clumps which are half stuck in the nervous tissue and half in the perivascular space are usually stained dark and obviously more solid. With their dissolution, the lymphatic fluid will obtain a pathological composition.

**474**

This explains the finding that the perivascular space is filled with substances which are distinct from the coagulation products of normal lymphatic fluid and that, depending on the applied fixation method and on the type of pathological tissue changes, differently stained substances precipitate.

In the perivascular space, one never finds products stained with Scharlach. More abundantly, one finds it at locations where the above-described substances accumulate in the perivascular space, in the cells of the adventitia, and in the adventitial lymphatic space as well as in the cells of the pia. Based on the enormous accumulation of fatty substances in the mesodermal tissue, it could indicate to us that the mesodermal cells rapidly take up substances from the pathologically contaminated tissue fluid and store them as fatty products. Only gradually would they turn them over. Thus, the entire process would work in the following way:

With the degradation of the myelin and the axons in white matter, the ameboid cells assimilate the degradation products which are mainly dissolved in the tissue. They transform them into fuchsinophil granules and lipoid substances. With a rapid and dramatic decay of the nervous elements, the glial cells disintegrate, forming different granules, in particular methyl blue granules. These degradation products are dissolved in the tissue and mainly in the perivascular space, taken up by mesodermal cells and converted into fatty substances. It remains open whether the substances are taken up from the perivascular spaces or may also transfer to the adventitial lymphatic spaces and be taken up from there. Maybe both possibilities occur. It is also open how these substances are transferred to the cells of the pia, either via the lymphatic space or whether the perivascular spaces open to the pia.

The biological importance of the ameboid glial cells may reside in their task to rapidly remove the degradation products of the nervous system or at least convert them into other substances. After a second dissolvement, they will be taken up by the mesodermal

**475**

cells where they are deposited. Since we cannot follow this process from the beginning to the end under the microscope, and since some stages might be in between in which the degradation products are dissolved and only partially visible, this assumed process can only be partially verified because there are unconfirmed gaps. We will see that some of these gaps can be filled by observations in the grey matter and from the analogy of other degradation processes which will be later described.

The degradation processes in the grey matter are of such high diversity that it would require a book of its own to fully describe them. This is not the task which will be solved here. The main purpose here is to relate certain glial forms to the degradation processes in the nervous system. Therefore, some examples may be sufficient.

One of the most obvious degenerative transformations of ganglion cells was described by Nissl as a severe cellular disease. As with other ganglion cell diseases, it occurs in different variations and concomitant with other alterations of the cell. The pure form is characterized by two features: 1. A severe transformation of the nucleus, which becomes rounder and smaller and stains darker in a metachromatic label; the nuclear membrane detaches totally or partially from the condensed nuclear content; 2. A peculiar transformation of the protoplasm beginning at the zone around the nucleus which is less labeled by basic color; it then dissolves into strange, small granules or ring-shaped formations. With the cell body, also the dendrites and the axon dissolve after labeling with basic Aniline stain in a peculiar dirty color. The axon initial segment remains longest.

One finds this typical cellular illness in some psychiatric disturbances with particularly severe symptoms. I have studied it in progressive paralysis, acute pellagra (19), untreatable forms of malaria, severe septic deliria, and peculiar, deadly progressing psychoses of progressing years.

**476**

In Nissl preparations, one usually observes particularly small, darkly labeled glial nuclei next to it, often with alterations as described above as regressive nuclear alterations of ameboid cells. Such nuclear forms are shown in Table XXXIII, Fig. 8–11 and 14.


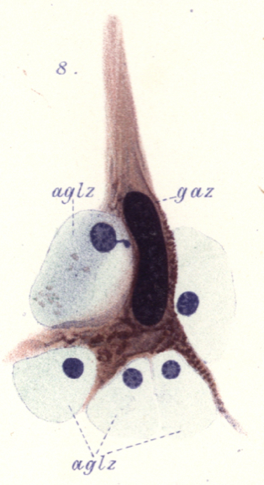

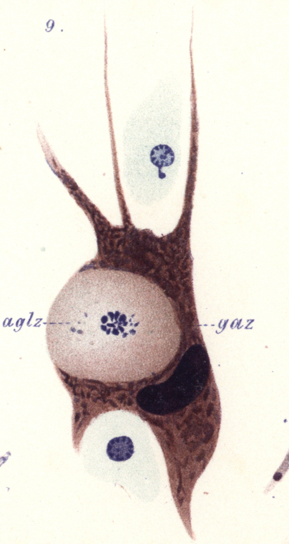

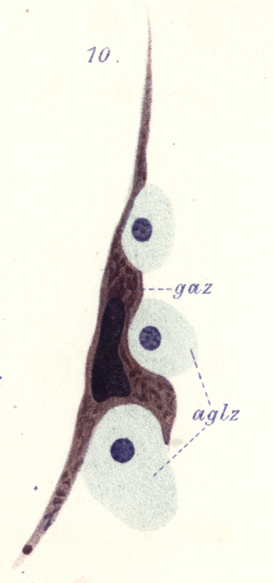

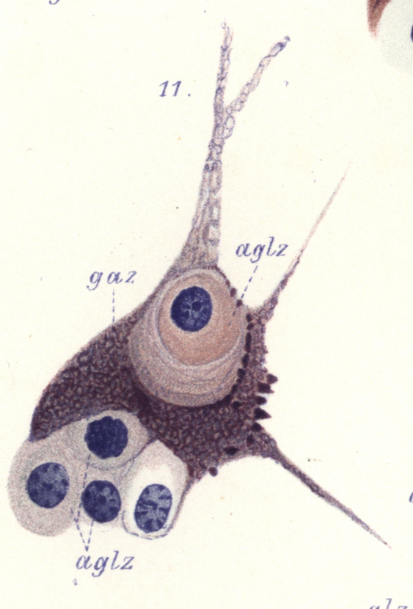


*Table XXXIII, Fig. 8. Ganglion cell surrounded by five ameboid cells. The nucleus is deformed due to the encroachment of an ameboid cells. In this glial cell beginning degeneration of the nucleus. Of a strange severe disease in the climacterium.*

*Table XXXIII, Fig. 9. A ganglion cell with an ameboid cell intruded into the cell body. The nucleus of the ganglion cell is deformed and relocated. Caryohexis of the glial nucleus. At the nuclei of the other ameboid cells also regressive alterations. From the same case as 8.*

*Table XXXIII, Fig. 10. Deformation of a ganglion cell and its nucleus by approaching ameboid cells.*

*Table XXXIII, Fig. 11. Ganglion cell with satellite cells from a case of acute catatonia. The ameboid glial cell is within a large niche of the cell body of the ganglion cell which it does not quite fill; at the edges of the niche simple basophil products, The glial nucleus shows regressive alterations (mulberry-shaped).*


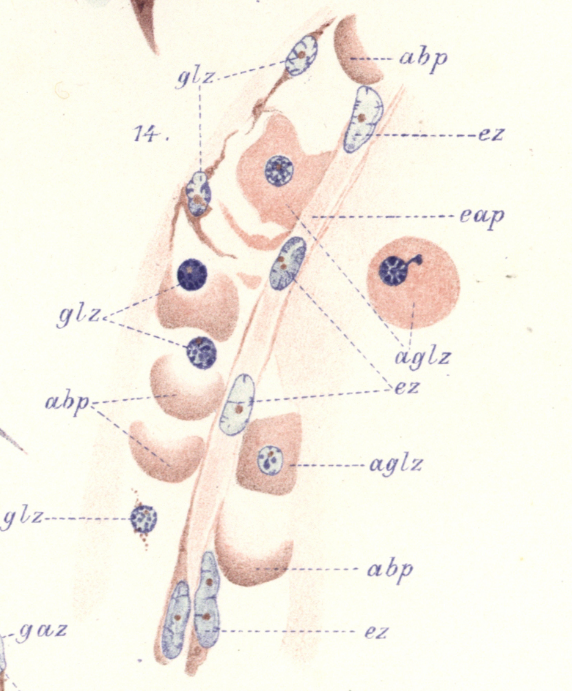


*Table XXXIII, Fig. 14 Capillary with perivascular space from the cortex of a case of fear psychosis. In the perivascular space reside ameboid glial cells and peculiar, red-labelled masses which represent pathologic coagulation products.*

If we are staining the alcohol slices with Giemsa solution as described above, we observe that the small glial nuclei are associated with relatively large, roundish protoplasmic cell bodies (Table XXIX, Fig. 7, 10, Table XXX, Fig. 4, Table XXXIII, Fig. 7).


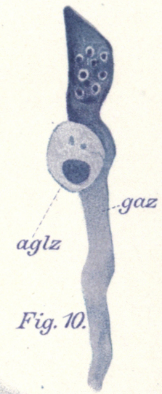

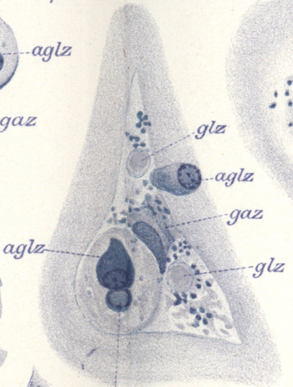


*Table XXIX, Fig. 7 In a tissue gap which shows the form of a ganglion cells are remnants of a ganglion cell, two young ameboid glial cells and three degenerated nuclei which obviously belong to decaying ameboid ganglion cells. The same case (progressive chorea).*

*Table XXIX, Fig. 10 Ameboid glial cells which lay in ganglion cells or their processes. Central sulcus. Progressive chorea.*


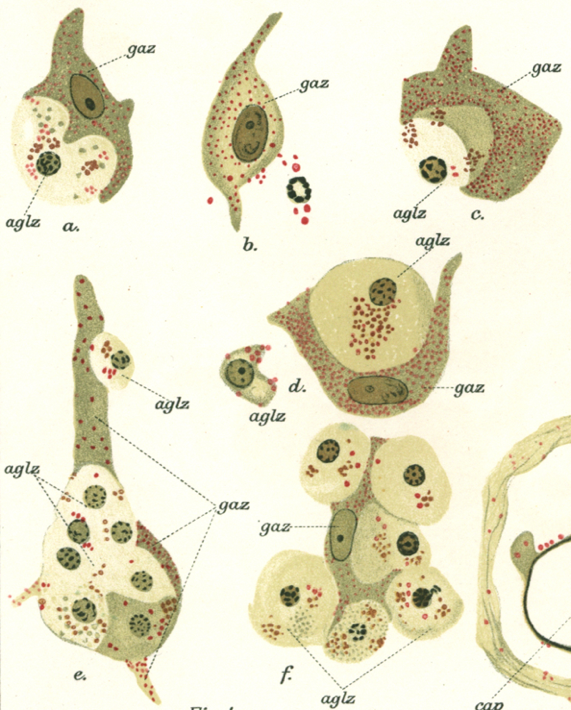


*Table XXX, Fig. 4 Ganglion cells with ameboid glial cells from the cortex of a delirium acute case of progressive paralysis. Deformation of the cells. Substitution of the cell body by ameboid glial cells. Nuclear degeneration of the ameboid cells.*


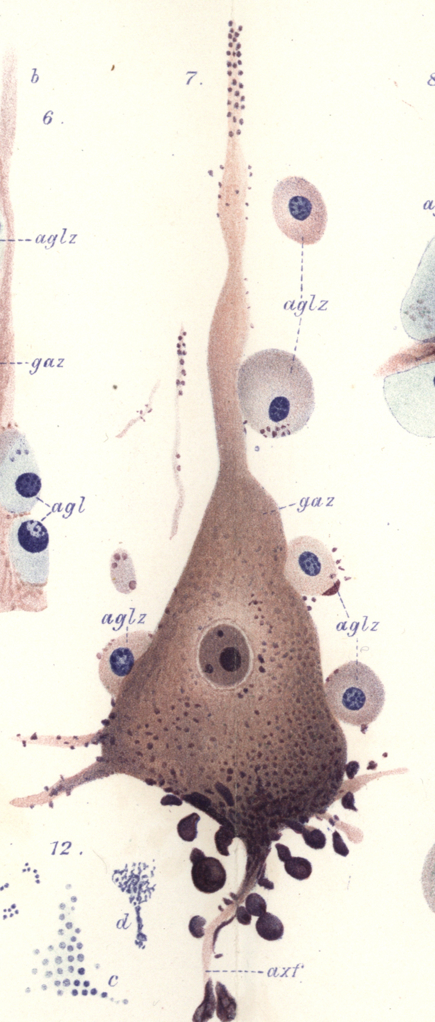


*Table XXXIII, Fig. 7 Beetz pyramidal cell of a case of delirium after acute paralysis. Nuclear alterations of a severe cellular illness of Nissl. At the top process and at an adjacent protoplasmic process are basophil granules. At the base of the cell and at the axonal process are drop-like accumulations of simple basophil products. Also, at the cell body of the ameboid glial cells which are around the ganglion cell.*

Besides Nissl’s severe ganglion cell transformations, there are ameboid glial cells quite common in the cortex. Using Method V, one often finds decayed axons in the white matter and in the cortex.

When studying the decay more precisely, it becomes clear that the ring-shaped bodies into which the cytoplasm decays are very variable with respect to staining. Some are clearly stained by basic Aniline colors, some dark initially but mostly faint, some even unstained. Using the Weigert glia method or the fibrin staining, some are light blue, most unlabeled. A larger number will be red after Flemming fixation and acidic fuchsin-light green staining, and among them are sparse granules stained brown or black with osmium.

Based on their staining reaction, we can make no conclusion on the chemical nature of these ring-formed granules: we must assume that they undergo a rapid transformation and are completely dissolved before they, at least the majority, are converted into lipoid substances.

This peculiar cellular degeneration seems to stimulate the formation of ameboid glial cells in a stormy fashion. Mainly glial cells with a roundish cell body are generated. These yield striking images of so-called neurophagy. This is not a space-filling task of glia. At the time of the disease, the nerve cells are rather swollen than shrunken and not yet decayed in large numbers, and the ameboid glial cells surround them in large numbers.

**477**

They insert into the cytoplasm of the ganglion cells, push the nucleus forward, and distort it. Similar events also occur at the protoplasmic processes, far from the cell body. We do not observe that the glial cells take up debris of decaying nervous tissue components. Our observations rather indicate that they exert a dissolving effect on the cytoplasm of the sick and decaying ganglion cell. One often observes, as shown in Table XXXIII, Fig. 11, that an ameboid glial cell resides in a larger, lighter indentation of a ganglion cell or that the part of the ganglion cell facing one or multiple ameboid glial cells is lighter and seems to be in a more advanced stage of disintegration compared to the opposite side.

*Table XXXIII, Fig. 11 Ganglion cell with satellite cells from a case of acute catatonia. The ameboid glial cell is within a large niche of the cell body of the ganglion cell which it does not quite fill; at the edges of the niche simple basophil products, The glial nucleus shows regressive alterations (mulberry-shaped).*

When we study the subsequent development of the ameboid glial cells with the acidic fuchsin-light green method, we find that within the small, dark, and homogeneously stained cell body, fuchsinophil granules develop and that with increasing size, substances appear which are labeled brown with osmium. They occur in the form of solid granules, while lipoid cysts are rarely found. With the appearance of these inclusions, the cytoplasm stains less intensely, becomes looser in its composition, coagulates, and dissolves completely. One often observes decaying ganglion cells surrounded by a group of ameboid cells in different stages of their development. Sometimes we find them at the remnant of a ganglion cell, characterized only by a decaying nucleus. Finally, the previous position of the ganglion cell is completely occupied by a cluster of ameboid glial cells. At the same time, pathological products and multitudinous ameboid glial cells accumulate in the perivascular spaces, and fatty substances in mesodermal vessel cells show a significant augmentation.

The observation that ameboid glial cells deform the cell body, the processes, and the nucleus could indicate that they destroy ganglion cells in an active fashion. Yet it is more likely that they are attracted by the pathological products generated by the ganglion cells as a stimulus. They may accelerate the dissolution.

**478**

It is thus likely to assume that the ganglion cell has been crossed over in a converted form into the protoplasm and the enclosed granules of the glial cells. They occupy in large number, filled with different degradation products, the space previously occupied by the ganglion cell. The major part of the pathologic substances which we find in the perivascular space seems to stem from the decay of these glial cells. These products are later transformed in mesodermal cells into lipoid substances. This observation closes gaps which could not be obtained in white matter. There, we could only rarely observe glial cells in a closer relationship to decaying nervous elements. Here we observe in multiple conditions how glial cells attach to sick nerve cells and how they obviously contribute to their dissolvement.

Another common disease type of the ganglion cells is the fatty degeneration. Also, here we find many variabilities, on the one hand with respect to number, size, and arrangement of the lipoid granules incorporated in the cell, on the other hand with pathological changes of the cell. In its typical forms, we find it in senile dementia, in paralysis, in arteriosclerosis, in lues (syphilis-related dementia), chronic alcoholism, and morphinism, at psychosis due to intoxications and infections, and finally in all degenerative processes in the nervous tissue combined with other alterations of the ganglion cell. It can be studied in the spinal cord in senile dementia. It occurs also in the cortex at senile dementia often accompanied by other alterations such as sclerosis.

The fatty degeneration of the ganglion cells has previously been described as pigment degeneracy, and the granules were described as yellow pigments since unstained and freshly isolated, they show a light-yellow color. Since we observe that many fatty substances acquire a yellowish color and found that the granules show reactions of lipoid substances, we should describe these incorporations as lipoid granules and the disease as lipoid degeneration. Moreover, the lipoid granules which are formed in younger years

**479**

and which newly appeared in acute degenerative processes appear often unstained, and the pigmentation is not a characteristic feature.

There is a large literature on the fatty degeneration of ganglion cells. In the papers by Obersteiner and Marinesco, we find these described including their own studies. With respect to the literature and the general features of the lipoid degeneration, I can refer to these authors. I will not pay attention to the brown and black melanotic pigment of the locus coeruleus, the substantia nigra and the vagal nucleus which has obviously another importance, and to the sympathetic and spinal ganglion cells including also the different storage of the fatty granules in the different cells. We will focus on the importance of the fatty degeneration for the ganglion cell and the nervous tissue in general, the kind of the fatty products, and the relation of the fatty degeneration to the ameboid glial cells.

The assessment of the pathological relevance of the fatty degeneration of the ganglion cells is hampered by the observation that lipoid granules are found in humans and larger animals without being related to any damage of the nervous system or without any disturbance in any functional aspects. We observe that the fatty granules appear already in early youth before the nervous system is fully developed. At that time, one cannot assume any wear or even involution of the nervous system. The opinion came up (Olmer) that the fat in the ganglion cells is a kind of reserve material for the nutrition of the cell. As Obersteiner, Marinesco and others have emphasized, the strong accumulation of fat in the ganglion cells at old age, as well as the increase in many conditions affecting the cell, speaks against such an assumption. We observe that important cellular components — cytoplasm, Nissl slabs and fibrils — are damaged, which are important for function, and that the cell dissolves with excessive accumulation of fatty granules. Therefore, the assumption

**480**

that fat is a beneficial component of the cell can hardly make sense. Obersteiner promoted the view that the fatty granules which occur in the ganglion cells in an undamaged nervous system correspond to waste products of metabolism which cannot be cleared to the disadvantage of the cell and that potentially those cells which more frequently accumulate fatty granules (lipophilic cells) are more involved in activity than those which show low or no accumulation of fat (lipophobic cells of Obersteiner). The first view has received general agreement. We observe in other cells of the body that degenerative fatty granules occur in early age, which seem not to affect the function of the cell, until their accumulation with age reaches an excessive stage, resulting in an alteration of the cytoplasm and resulting in a dysfunction of the organ. We will regard also those fatty granules as degenerative products. It thus makes sense to distinguish between a physiological fat degeneration, which remains at a normal level, and a pathological one which oversteps the level. For the analysis of the pathological fatty degeneration, the understanding of the physiological is a prerequisite. It is very difficult to distinguish between these two conditions since there is considerable variation between different regions and cell types, changes with age, and even individual variations. Thus, it is very difficult to define rules for defining the physiological and the pathological state. Nissl emphasized that a cell can be defined as degenerated if the entire cell body is filled with fatty granules with atrophic dendrites incorporating fatty granules. This may refer only to the most severe and most obvious cases. We will see, and come back to this, that the fatty granules are increased due to several pathological processes, and this should be viewed as pathological. In humans, the pathological fat accumulation can often only be verified by

**481**

identifying the precursor stages which exist only for a short time; in animals which lack fatty bodies under normal conditions, such as in rabbits, the appearance of even a few fatty granules can be viewed as pathological. Also, some peculiar arrangement of the fatty granules in ganglion cells can indicate a pathological origin; Obersteiner has depicted some examples.

The view expressed by Obersteiner that the fatty substances accumulate in ganglion cells since their removal is not possible raises the question about the mechanism by which lipoid granules generated in nerve cells are exported and removed. Wherever we find abundant fatty granules in ganglion cells, they are also present in satellite cells (20) and in cells of the vessel wall. One has to address the question whether the fatty granules in these cells are formed due to the same damage as in the ganglion cells by conversion of the cytoplasm or whether they originate from ganglion cells. The first option may certainly occur. The glial cells of the first layer of the cortex generally contain fat which originates due to the degenerative alteration of the cytoplasm of the ganglion cells. In older glial scars, we observe a decaying cell body of fibrous glial cells filled with fatty granules which may have been generated in a similar fashion. Occasionally, we observe images indicating that the ganglion cell may be able to expel lipoid granules. In the pyramidal cells of the cortex, one observes fatty granules which are extended to sausage- or barbell-like formations and with half of their extent beyond the ganglion cell body. These are mainly sclerotic cells, and one has to consider that due to the shrinkiage of the cell body, these formations could be pressed out. Also, in other cells such images could occur due to the artificial shrinkage of the cell body. Thus, these observations cannot be taken as proof that the ganglion cells can get rid of their ballast in such an easy way.

**482**

In sclerotic cells, we often find the lipoid granules cup-like, as if being leached out. These are necrotic cells, and it is evident that these cells, due to fatty degeneration, rapidly fall apart into piles of fatty granules. With the contribution of glial cells, they become completely dissolved.

That the living cell can release the fat generated under pathological conditions is likely based on animal experiments. The interpretation of the findings is less difficult, since with adequate selection of the experimental animal, physiological fatty degeneration is not relevant. Young rabbits normally do not have any trace of fat in the central nervous system. After two months of intoxication with lead carbonate, there can be a considerable mass detected in the ganglion cells, scattered in glial cells, and in the cells of the vessel wall. In control animals, the intoxication was stopped on the day when the first cohort was sacrificed; after an additional 6 weeks, there was little fat in the ganglion cells, more in the glial cells, and even more in the cells of the vessel wall. This result suggests a transport of the fatty substances from the ganglion cells via the glial cells to the cells of the vessel wall. Also for this result, some concerns remain. Under certain pathological conditions, one finds fat in glial cells which does not originate from ganglion cells, since it is found before it appears in those. I have observed this for quite some time for alcohol and phosphor intoxication, Achúcarro for rabbies, Bonfiglio for lead intoxication.

It is remarkable that everywhere where fat is found in ganglion cells under physiological conditions, it is also present in glial cells and cells of the vessel wall. In animals which normally lack any lipoid granules in the nerve cells, they also lack them in glial cells and cells of the vessel wall. In humans and in several animals, the presence of fatty substances in the glia and in the cells of the vessel wall, like in the ganglion cells,

**483**

is within the range of normality. If one would consider the presence of fatty granules in glial cells and cells of the vessel wall as generally pathological, one would in vain be searching for a normal brain of an adult human. That fatty products are found or not found at the same time in ganglion cells, glial cells, and vessel wall cells indicates a close relationship of these three cell types with respect to the presence of fatty substances.

Under pathological conditions, there are exceptions from these rules. I have frequently observed cases of senile dementia in which the ganglion cells in the cortex were excessively fatty degenerated, while the glial cells did not contain any fat. With closer inspection, these glial cells seemed considerably degenerated, and it cannot be excluded that they have lost their capability to take up or generate fatty substances.

It is unlikely that the appearance of fatty granules is due to normal or high functional activity and that they are taken over by glial cells. The metabolic function of the cell is related to substances which we cannot yet identify, but which are not related to the production of fatty products. Only under certain conditions may a granular conversion of the cytoplasm to fat occur. We also have no evidence that a transition or uptake of fatty granules from one cell to another occurs. Analogies to other types of degenerative processes rather indicate that fatty substances are dissolved before they enter other cells.

The animal experiment indicates that single fatty granules scattered over the cell disappear more easily from the cell than large accumulated fat masses which occupy a large part of the cytoplasm, establish at preferred areas of fatty degeneration, and lead there to a change in the cellular structure. However, it needs to be clarified whether cells generated in other parts of the cell may translocate and be deposited at these preferred localizations (21).

**484**

It is undisputed that the incorporations in the ganglion cells are of lipoid substances. They stain brown with osmium after previous chrome treatment and are stained bright red with Scharlach. After longer incubation of the slices in alcohol and ether, they are largely extracted, in particular with simultaneous heating. They differ in several important ways from fatty substances found under other circumstances in the nervous system.
A slice of the spinal cord white matter containing multiple gliogen granule cells with incorporations labeled black with osmium after chrome treatment or a freeze-fractured formaldehyde slice labeled red with Scharlach is hardly labeled with the Nissl stain (Table XXXIII, Fig. 4 and 5).

*Table XXXIII, Fig. 4 Glial grid cells (granule cells) from the white matter of the posterior and lateral strand of a case of progressive paralysis. The fat which resided in the sphere-shaped cavity is completely extracted (compare Table XXXIV, fig. 2 and 6).*

*Table XXXIII, Fig. 5 Glial grid cell close to a focus in the white matter of a case of syphilis (?) related encephalitis with completely empty cavities; control preparations show that they were filled with fat droplets.*

This fat does not show the tendency to acquire a yellowish color. In such a slice of the spinal cord after being embedded in 96% alcohol at room temperature, one finds that most of the incorporations in the granule cells are extracted and only the grid structure of the cell membrane can be nicely illustrated; the small lipoid granules in the grey substance remain undamaged. They are thus more difficult to dissolve in alcohol compared to the fatty substances of the gliogen granule cells. There are a number of color reactions corresponding to the lipoid inclusions of the ganglion cells, which do not label the fatty substances of the gliogen granule cells in the white matter. The lipoid substances of the ganglion cells stain blue or black with different variants of the Weigert stain, while in the other cells only the grid structure is labeled. After fixation with chrome acetic acid without osmium and acidic fuchsin-light green staining they are red, after fixation in alcohol and carbol fuchsin methylene blue staining they retain the color of the carbol fuchsin. As I have known for a long time and as Casamajor recently described, they stain blue with the Weigert fibrin method. With respect to these staining reactions, the lipoid substances in the gliogen granule cells respond differently and are stained either not at all or only to a low extent.
These are just a few examples, and several others could be added. This indicates that we are dealing with a peculiar lipoid substance.

**485**

It is not only specific for the central nervous system but can be found in other cells of the body. In the nervous system, we find it not only in the ganglion cells but also in the glial cells, the cells in the vessel wall, and the pia. I will not provide a name for this lipoid substance referring to its specific chemical composition, also for reasons I will discuss when describing the basophilic metachromatic substances.
We also learn that this substance does not show a completely uniform staining reaction. In some instances, it appears in the unstained preparation, as previously mentioned, not with the usual yellowish color, but colorless. In others, it is obviously dark, brownish, or even greenish. In particular, in acute stages one observes in alcohol preparations many granules that acquire the blue basic Aniline colors with a greenish tone. Not even rarely does one observe that the shell and the core of the individual granules stain differently. There are granules that are black with osmium and after chrome treatment, others obtain yellowish or dark brown labels. I will not further discuss the variability of the granules with respect to form and size and their arrangement and composition within the cell.
We have found that the fatty cysts, the lipoid accumulations in the ameboid glial cells, originate from fuchsinophil granules. Therefore, it is interesting whether precursor lipoid substances are found in ganglion cells. One has the impression that in preparations stained with the silver method of Ramón y Cajal or even better with the silver method of Levaditi for identifying spirochetes, there are more granules which stain brown with osmium or red with Scharlach. This impression is obvious in cells which show an acute fatty state or in severe deliria due to infections. It is difficult to reach a final conclusion since, by using different methods, one does not observe the same cells or even the same region in the cortex.

**486**

One regularly observes that between brownish granules, red ones are found with acidic fuchsin-light green staining. These are much larger than the normal fuchsinophil granules of the ganglion cells and are rather of the size of lipoid granules. Since one observes pathologically large fuchsinophil granules in the ganglion cells, these findings are not unequivocal. Recently, I obtained preparations which clearly indicate that the lipoid granules have fuchsinophil precursor states. In a case of severe tetanus after resection of a struma, I found in the dorsal horn cells of the spinal cord, in particular at swelling of the neck, many cells with clumps of lipoid inclusions surrounded by a tight ring of granules stained intensely with fuchsin. In the border zone between them, there were always those which were brown with a mix of red and red ones with a mix of brown. These precursor products of the lipoid granules were distinct from the fuchsinophil granules of the ganglion cells due to their larger size and their looser composition. We thus must assume that granules are generated from the cytoplasm which have the tendency to be stained with acidic fuchsin and that the fuchsinophil granules transform into fatty products.
The chronic fatty degeneration of the ganglion cells usually starts at the same position and then progresses gradually from there. Sometimes it is a zone close to the nucleus (dorsal horn cells, pyramidal cells of the cortex), sometimes at the nucleus at the onset of the main dendrite (Purkinje cells), sometimes at the main dendrite proper (cells of the hippocampus). In acute fatty degeneration, we often observe a uniform distribution of fatty granules within the entire cell body. From our images, we get the impression that the fat forms from the basic substance of the cytoplasm and that the Nissl substance is part of that transformation, while the fibrils are fairly resistant. Wherever fatty bodies occur in mass, the ganglion cell undergoes changes like any other cell in which fatty bodies are formed. The cytoplasm obtains, at the positions where fatty bodies are present, a comb-like structure, like in the grid cells, and in the combs reside the granules. The Nissl substance deteriorates. The fibrils survive longer in the combs.

**487**

What one can stain with the Bielschowsky method (22) is not a network of fibrils, but rather the mesh of the plasma comb network. The formation of abundant lipoid granules results in swelling of the cell. Thereby, it is often deformed. In particular, at simultaneous sclerosis, the fatty positions protrude (fatty sacs), the nucleus is displaced by giving way to the fatty accumulations, the fibrils are partially displaced, the processes swell whenever fat is forming there, or they shrink if there is an accumulation of fat at their origin (Nissl). Finally, the entire cell can convert into fatty granules, resulting in its decomposition.
One can distinguish a variation from the usual form of fatty degeneration of the ganglion cells. In senile dementia, arteriosclerosis, and within or around the focus of an encephalitis, one occasionally finds ganglion cells that contain particularly large fatty granules. This variation could refer to the fourth form of fatty degeneration described by Marinesco ("gros corpuscules"). One observes that particularly large fatty granules occupy part or even the entire cell body, and often each fatty granule seems to reside within a vacuole. These large fatty granules show certain specialties. A part of them, obviously the newer developed, acquires a remarkable dark blue color with method V and also by staining with Ehrlich’s hematoxylin and do not stain red with Scharlach. Another part is stained with Scharlach. None of the granules stain with the method of May-Grünwald. With lithium carmine, a part of the granules stains remarkably bright red; in the Bielschowsky preparation they take up the silver. Using the acidic fuchsin-light green staining after Flemming fixation, some are stained bright red, others strongly brownish like the cysts of the ameboid glial cells. Thus, we see here precursor stages of the large fatty granules, which we did not find in the fine-granular fatty degeneration.
Repeatedly, I have found in very old people who had developed a rapidly progressing degeneration resulting in complete immobility, multiple ameboid cells in the spinal cord and in the grey and white matter.

**488**

They were not distinct from the ameboid cells which we have observed in the severe cellular alteration described by Nissl or in the monkey spinal cord. The Nissl preparation shows strongly fatty degenerated cells mainly with peach-like or round shapes without processes; some had protoplasmic processes strongly swollen due to the fatty incorporations. In other, obviously chronic cases, similar alterations were found in ganglion cells without any trace of ameboid cells. How can the appearance and relevance of the ameboid cells be explained? I think that the acidic fuchsin-light green preparations give some conclusions. Let us observe a normal control preparation. One best selects slices from the cervical or lumbar swelling where many large ganglion cells are found next to each other.

*Text Fig. 4. Coarse granule fatty degeneration of ganglion cells. A from the hindbrain of an arteriosclerotic. B from the hippocampus of dementia senilis (shows besides the yellow-greenish stained ordinary fat 5 blue labelled large granules). Method V.*

Except for the ganglion cells, one observes that the entire tissue is filled with abundant, highly branched protoplasmic processes which have been separated from the ganglion cell due to slicing. All are densely covered by countless neurosome clumps (23) and above, a large number of delicate axons run by. They often run parallel to the protoplasmic processes and circumvent cell and dendrite. We observe how their delicate branches descend to the level of the neurosomes (8) , passing through, and here or there one has the impression that they interact.

**489**

The preparations do not permit recognizing more. Besides that, larger axons and myelinated fibers cross through the field of view. The rest is filled by delicately structured glia and single glial fibers. Yet the support tissue is reduced compared to the masses of neuronal elements.
In the senile spinal cord with the strongly fatty ganglion cells, the image is quite different. The number of protoplasmic processes is sparse. Quite a lot have already degenerated. Related to that, the normally highly branched ganglion cells have often acquired a round form. The neurosome accumulations are missing at the fatty regions, and also in other regions they have become sparse. Together with the protoplasmic processes, they have disappeared. The number of axons which connect to the cell is low compared to the numbers in normal preparations.

*Text Fig. 5. Normal ganglion cell from the ventral horn of a rabbit. It shows the neurosome clumps from a ganglion cell, the granules in the cell body and the granules belonging to glia. Method VII.*

The number of glial fibers has increased. The number of ameboid cells has increased, which are either attached to the ganglion cells or dispersed in the tissue. Besides that, one finds filled bodies in more or less amounts. If one considers how close the nervous structures are compacted in the grey matter, it becomes clear that with the presence of those multiple glial formations, quite a lot of nervous tissue has already degenerated. Since I cannot present all of

**490**

these relations in individual drawings, I will refer to Table XXXV, Fig. 1 and 2, which illustrate similar conditions.

*Table XXXV, Fig. 1 Ganglion cell from the ventral horn of a normal, older dog. In the cells are green Nissl slabs and fuchsinophil granules associated to the cytoplasm. At two locations are lipoid granules. At the rim of the cell and at processes are red labelled piles of neurosomes (8) visible. At the capillary are lipoid substances. In the tissue, one observes multiple myelin sheaths and axons. The rest is largely filled with protoplasmic processes of neighbouring ganglion cells, which are embedded by piles of neurosomes.*

*Table XXXV, Fig. 2 Degenerated section from the ventral horn of a case of progressive chorea. In the middle a ganglion cell with the nucleus not sliced with multiple lipoid inclusions. Piles of neurosomes are only occasionally observed. The axon and the protoplasmic processes of the ganglion cells are hardly visible. The entire tissue is dispersed with greenish, roundish bodies (filled bodies).*

We can see clearly that at the fatty degeneration of ganglion cells, a massive degeneration of delicate nervous tissue components occurs while the coarse form remains. This is because the widely branched dendrites of the cell and the approaching axons, the pericellular arrangement, have deteriorated. Such examples are quite instructive, since they demonstrate how much of the nervous tissue became deserted and that with our old methods, we do not detect it. Unfortunately, the relations in the cortex are even more complex than in the spinal cord, since all elements are more delicate and thus more difficult to demonstrate. Even with the new methods, we cannot yet obtain completely clear images. Thus, we have to restrict our observations to what we can obtain from the coarser grey matter structures. Besides the ameboid cells, we find other glial elements which have formed fibers or those, like small granule cells, which are fully loaded with fatty granules and are devoid of fibers. All observations indicate the rapid fatty degeneration of ganglion cells, and the appearance of ameboid glial cells correlates to a rapid decay of dendrites and the delicate surrounding axons, representing the pericellular apparatus. This makes it likely that in regions where we observe fat-loaded glial-associated cells, even without observing fat in the ganglion cells, it is correlated to a decay of periganglionic structures.

I believe that these examples are sufficient to illuminate the biological tasks of the ameboid glial cells. Their task is not to provide a support system like normal glia. They do not generate fibers and networks, but rather originate where a degeneration of the nervous tissue components occurs, because the stimulus of the degradation products induces them. Since we do not observe that they take up degrading nervous structures in a physical way, we have to assume that they help to dissolve those. Images which we observed on ganglion cells

**491**

do not leave room for any other interpretation. Finally, they replace the ganglion cells, which completely disappear or are dispersed in the tissue, while they increase in size. This indicates that they assimilate substances which are generated due to the dissolution of the ganglion cells and other nervous structures. After arriving at the peak of their development, they undergo regressive changes with the formation of different granules and decay rapidly. Their degradation products end up in the lymph and in the perivascular spaces, partially in a dissolved form, are taken up by mesodermal cells and transformed into lipoid substances. Thus, they clean up the nervous tissue of degradation products, transform them into a form less harmful to the ectodermal tissue, and pass them on to the mesodermal tissue which stores them for the time being.
While we cannot display all the different degenerative processes in the nervous tissue, one should mention the observation that besides simple forms of ameboid glial cells in the white matter, there are extraordinary different variations in the cortex. One could explain this by the fact that the disease processes in the cortex are much more variable based on different disease states of the ganglion cells, due to varying contributions of the delicate nervous structures, the delicate protoplasmic processes, and the tender axon branches, whereas in white matter there are more quantitative differences. For some of these degenerative processes, it can already be directly substantiated. It seems that the closer the cell form corresponds to the basic ameboid type, the stronger the damage. Thus, the degree of development of the ameboid glia is a measure of the severity of the nervous transformation.

**492**

Of high pathologic relevance and closely related to the appearance of ameboid glial cells is the appearance of multiple large fuchsinophil granules in the ganglion cells. We observe this under different conditions: first during chronic changes of the ganglion cells, namely in some forms which correspond to sclerosis. In the Nissl image, we find a reticular structure of the cytoplasm of the ganglion cells, and we must assume that in the meshes of the reticulum are large fuchsinophil granules embedded besides a fibrous and inactive glia.

*Text Fig. 6. A Degenerative fuchsinophil granules of the nerve cells. Third cell layer of paralysis. Frontal cortex. B Corresponding image from a Nissl preparation, same location, shows gaps instead of granules.*

Then in severe cellular sickness, e.g., in paralysis or dementia praecox, alongside multiple ameboid cells. At some other disease states, one finds ganglion cells that are crammed with fuchsinophil granules without deviating in form and size from the normal. In the processes, the granules are arranged in rows (they are besides glial cells with rows of fuchsinophil granules in long processes or not altered glial cells). In the first case, it corresponds to a chronic degenerative condition — a type of necrotic cellular degeneration; in the second case, to a degenerative process; and in the third, only to a functional change. These fuchsinophil granules are in no case precursor products of lipoid substances. Attempts to differentiate the different fuchsinophil granules by staining were so far not successful.

**493**

Quite unusual and so far less understood preparations I have obtained from a case of psychosis in the menopause (24) which was, with respect to the clinical features and course, unusual. Everywhere one found ameboid cells. Many ganglion cells of the deeper cortical layers showed severe cellular alterations according to Nissl.
Attached to the cell were club-like, striated formations stained red with the acidic-fuchsin light green method which obviously did not belong to the cell and most resembled pathologically altered axons attached to the cell (Text figure 7). Strangely, they stain blue with the Weigert method. I have never found that again.

*Text Fig. 7. Ganglion cell with severe cellular Nissl alteration. It contains lipoid granules and dispersed large fuchsinophil granules. The cell is covered with thread-like, club-like enlarged formations which could be considered as axon endings. Acidic-fuchsin light green.*

These methods show us alterations which could be generally understood, but in addition some others which are yet unclear. Future more detailed studies will yield further insight into the detailed pathologic processes of nerve cells.

**5. On special basophil metachromatic degradation products.**

(p-granules of Reich)

In several studies, Reich described a peculiar form of granulation in Schwann cells of peripheral nerves. He described their morphological features and staining properties in detail. Based on their solubility in 45 °C heated alcohol and heated ether and based on common staining features, he considered them identical or at least closely related to protagon (25) first described by Liebreich in 1865 in brain substance based on chemical procedure.

**494**

Reich expressed the opinion at several places that my studies on the degeneration of the nervous system originated from his. He came to this assumption due to an error on my side, which I need to correct. As I stated at the beginning of this paper, I defined it as my task to analyze how lipoid substances from degeneration of the cortex end up in cells of the adventitia and pia. Thereby I searched for precursors of these substances. It was evident to analyze whether the same substances appear during the generation of the nervous tissue as compared to during development. Thus, I considered Wlassek’s studies on the origin of myelin. Wlassek showed that the protagon is stained with Weigert’s myelin stain. These and other observations, which are unnecessary to detail here, inspired me to assume that the incorporations in the ganglion cells at amaurotic (26) idiocy were related to the protagon, and I named those and other granules protagonoid granules. Already the term "protagon-similar substances" should have expressed some concern about the complete similarity to protagon, which was substantiated by further studies. However, finally, Reich’s study from 1907 on "The organization of nerve fibers based on micro-histochemical studies" (27) appeared when a good part of my study was already complete. It taught me that Wlassek and I were most likely wrong. The substances termed by me and my students as protagonoid granules were in fact neither protagon granules nor π-granules of Reich. Since then, I have intensely studied the π-granules of Reich. I had previously observed them in peripheral nerves and was particularly alerted by Reich’s lecture in Dresden. At that time, I believed that they were mainly relevant for the peripheral nerve. There are several reasons why I term substances related to the π-granules of Reich as basophil metachromatic granules and not as protagon or protagonoid granules. It is recommendable to provide a novel name to a newly described substance to avoid discussions of what one talks about. Reich found it likely that his π-granules are related to protagon. I have serious reservations about identifying substances stained in microscopic preparations as substances identified chemically for several reasons: first, my own previous mistake; second, our insufficient knowledge of the chemistry of the brain. Moreover, the large number of different granules and substances which we find in the nervous system far exceeds the number of substances which chemistry

**495**

has so far found in the central nervous system, and we are dealing with pathologic products, while chemistry has scarcely studied substances generated under pathologic conditions in the nervous system.

Today we know already different substances identified by our staining methods which may belong to fat, some to myelin-like, and two to the protagon group. Which ones should we term fat, myelin, or protagon? Since we can distinguish them today only by their staining features, we should classify them based on their uptake of colors. Moreover, several observations indicate that these substances occur in the central nervous system not in a pure form, but rather in multiple mixed conditions. They are in a labile condition and can undergo rapid changes in the tissue. Overall, I would have no concern to name the basophil metachromatic termed substances as π-granules as defined by Reich. I have no doubt that all substances which I describe in the following as metachromatic basophil coincide with the π-granules of Reich.

Reich deserves the merit that he determined the morphological and staining properties of his π-granules very precisely. Table XXXII, Fig. 8 shows four Schwann cells from a root of a luetic meningomyelitis which illustrate the form and staining very well. They are accumulated more abundantly than usual in the obviously enlarged cell body.

*Table XXXII, Fig. 8 Four Schwann cells from the root of a case of syphilis related dementia with menigomyelitis, which shows Reich´s p-granules.*

One can well recognize how the cell, only recognizable by the nucleus and the accumulation of granules, enwraps the entire myelin sheath. The granules are well defined by their form: they are rod-, striated-, comma-shaped fairly large formations, which are grouped closely together at single locations — here at the lower position like onion shells and arranged in larger formations. Secondly, they are also characteristic due to their staining. They show, with treatment of thionin, toluidine blue, or cresyl violet, a particularly brilliant metachromasia (28), brighter than the metachromasia of the cytoplasmic cell bodies or the granules of mast cells. It is highlighted nicely, as Reich already mentioned, by artificial illumination with electric light.

**496**

We must memorize these morphological and staining features if we want to follow up their presence in the central nervous system. One faces larger difficulties illustrating this. While it is easy to obtain a selective staining of the π-granules in peripheral nerves, this is hardly possible in alcohol slices of the central nervous system and even more difficult in slices fixed with formaldehyde or Müller or Orth solution. In alcohol slices, Unna’s neuromucin is stained, and I agree with Reich on his interpretation. In preparations stored in little or unreplaced alcohol, one additionally observes the rosette-shaped precipitates — extraction products of myelin sheath at positions, e.g., in the ventral horn cells of the spinal cord, where they were obviously finally deposited after precipitation from alcohol after it became less concentrated by evaporation or dilution with water from the tissue. With progressive destaining of the myelin sheath substances, the present metachromatic cellular inclusions also destain, as verified in control slices. It is then difficult to determine the amount of these substances or to distinguish them from extracts of myelin sheath precipitated in cells. Less problematic, but still disturbing, is the observation that part of the basophil metachromatic substances, particularly the delicate incorporations, destain before the white matter is sufficiently destained to make them more clearly visible. The finding that they can be stained much more easily and isolated in peripheral nerves and converted to permanent preparation is due to the fact that they are deposited in larger portions and in a more pure and tighter form.

As careful as one must be in the judgment of the alcohol preparation, it is still useful to deal with them. The Nissl stain is still the most common method for the study of the central nervous system to obtain a general overview of the pathologic changes.

**497**

As control preparations demonstrate, we can recognize quite the same from the basophil metachromatic inclusions if we operate with care.
Table XXXII, Fig. 6 shows a longitudinal section through a vessel with its adventitial lymphatic space from the white matter of a man who had rapidly died from an infectious delirium (of unknown type).

*Table XXXII, Fig. 6 Longitudinal section of a vein in the white matter from a case of delirium after infection. In the adventitial lymphatic space are lymphocytes and cells with a large, faint red labelled cytoplasmic cell body (presumably macrophages). In the granule cells originating from the macrophages are greenish, yellowish and metachromatic substances (the fatty substances are partially extracted by the alcohol).*

The slice was prepared after only 18 hours of fixation in 96% alcohol without embedding and stained with toluidine blue. In the adventitial lymphatic space we observe a number of nuclei with little cytoplasm, which we can clearly identify as lymphocytes. Besides are some cells with large, light nuclei and larger, round, homogeneously faintly labeled cell bodies which can be considered, as observations at other locations indicate, as the mother cells of granule cells. They are grid-shaped and partially filled with reddish, yellowish, and greenish masses and fill the lymphatic space. Of the different colored incorporations, we are mainly interested in the reddish labeled. This color refers to the π-granules of Reich. Yet we do not observe any forms characteristic for the granules of the peripheral nerves. The red substance is deposited in the smallest clusters which are roundish, yet mainly irregularly delineated. They are densely placed above or next to each other and seem to fill the entire cell body like a cloud and seem to push the nucleus to the periphery. In most cells, we observe besides the reddish substances, yellow and green clumps. In general, one finds in the younger-looking cells predominantly or exclusively red substances, in the older predominantly or exclusively yellow or green substances, leading to the conclusion that in these cells the red substances convert into yellow and green.

Table XXXII, Fig. 11 shows three glial cells from the white matter of the midbrain from a case of progressive chorea after brief alcohol fixation and toluidine blue staining. It shows how abundantly these substances are deposited in glial cells.

*XXXII, Fig. 11 Three glial cells from the white matter of the midbrain of a case of chorea progressive, loaded with basophil metachromatic products.*

In both cases, one can exclude that these metachromatically stained products were released from the fixation alcohol in which they could have been dissolved by extraction of alcohol-soluble white matter components and deposited in the cells;

**498**

the entire form of the cell, the transition of the nucleus and its degenerative alteration indicates that we face cells which had been transformed by the accumulation of pathologic substances into granule cells. If one still would have concerns, it can be relaxed by observing images like in Fig. 5 and 10 which were obtained from frozen formaldehyde slices. Fig. 5 shows an image of a small vein from the white matter of an epileptic patient who had many seizures and died when he suffocated during a seizure. Fig. 10 shows two glial cells from the medulla oblongata of an arteriosclerotic.

*Table XXXII, Fig. 5 Small vein from the white matter of an epileptic patient. One observes granule cells in the adventitial lymphatic space which in part are almost completely loaded with basophil metachromatic substances which contain partially greenish labelled metachromatic substances. The basophil metachromatic substances are also found in glial cells of the ectodermal tissue.*

*Table XXXII, Fig. 10 Two glial cells from the medulla oblongata from a case of an arteriosclerotic loaded with basophil metachromatic substances. The cell on the right shows at its lower part a honeycomb-like structure of the cytoplasm.*

These pictures confirm that there are granule cells which are filled with metachromatic basophil substances. In Fig. 5, we observe multiple large cells with nuclei in the adventitial lymphatic sheath which are pressed towards the surface and which show the same regressive changes as we observe at the nuclei of the clean-up cells which are filled with fatty granules. Also, here the red substances are incorporated in larger and smaller aggregates which often are composed of smaller, generally roundish, mainly undefined clumps, but not in such striolated and shell-like form as the π-granules of the peripheral nerves. Also here, we observe that the cells have greenish inclusions. One finds clumps which are still reddish, but with a trace of yellow or green, or those which are half red, half greenish stained. It appears without doubt that the reddish labelled substance is converted into a yellow or greenish. The same image gives us a good overview of these basophil metachromatic substances. The largest mass we find in large cells, most probably of mesodermal origin, within the adventitial lymphatic sheath. All the way to the left, we observe a cell, which is positioned half in the nervous system and half in the perivascular space. Due to its position, it may be considered to be a glial cell despite the fact that it does not look different in appearance from the cells which are within the adventitia. Yet one cannot exclude that in a frozen slice such a cell may be displaced by the knife from its original position.

**499**

Fig. 10 demonstrates that very similar glial cells occur in the middle of the nervous tissue. Almost everywhere where we find many of those substances in the lymphatic sheath, we find glial cells in the neighboring nervous tissue which contain the same substance. Those which are closest to the vessels are most strongly loaded; those which are further away contain less of those substances within the cells. Among the cells, we observe those which have only a few granules around the nucleus, others in which they are arranged at both poles of the cell, and those in which they are localized in tight clumps at one side of the nucleus or in long, whip-like extended processes. The last form we observe most frequently. In those cells, there are usually no yellow or green inclusions. As shown in Fig. 7, one can find in some glial cells both forms next to each other.

*Table XXXII, Fig. 7 Two glial cells from the white matter of a case of senile dementia which contain basophil, metachromatic and greenish lipoid substances.*

Some of these metachromatic basophil labelled inclusions correspond due to their rod-shaped form more to the π-granules. One can state that one finds those at locations where there are only few inclusions in the glial cells.

A similar image as Fig. 5 shows Fig. 4 which is obtained from deeper cortical layers in the area of the radii from a patient with severe manic excitement who died of exhaustion (?).

*Table XXXII, Fig. 4 Surrounding of a small vein of deeper cortical layer from a man who died after severe manic excitation. Basophil metachromatic substances in the glial cells, in the perivascular space, and greenish lipoid substances in the cells of the adventitia.*

We observe again a small venous vessel. The cells of the intima and adventitia are clearly outlined and also the perivascular space and the slightly stained nervous tissue. In the perivascular space there is a pile of glial cells in which the nuclei are obvious, in between other glial cells which are loaded with red substances. The largest amount of these substances is placed in a strange arrangement radial to the vessel, most likely within glial processes; within the large red clumps are green granules embedded. Also in the nervous tissue, one can recognize some glial cells with basophil, metachromatic inclusions.

Frequently one observes inclusions in the form of the π-granules of the Schwann cells. Fig. 9 contains images of a mushroom glial cell from the white matter of the spinal cord of a case of meningitis due to tuberculosis.

Table XXXII, Fig. 9 Basophil metachromatic inclusions in a slightly growing glial cell in the white matter of the spinal cord of a meningitis due to tuberculosis in the form of Reich´s p-granules. Features of degeneration at the nucleus of the cell *a*; in cell *d* onionskin arranged p-granules.

The entire cell body is filled with tightly formed, stippled structures.

**500**

Sometimes one observes it only in the processes, far away from the cell body. In Fig. 9d, it is entirely arranged in onion skins, as also often found in peripheral nerves. Also, in some cells of Fig. 1 and 2 one observes clearly stippled structures.

Table XXXII, Fig. 1 Glial cells from the cortex and white matter from different diseases with metachromatic inclusions.

Table XXXII, Fig. 2 Ganglion cells surrounded by satellite cells which contain metachromatic basophil inclusions from deeper cortical layers of a case of severe manic excitation (death by exhaustion?).

As far as I can judge, one does not find these substances freely in the tissue, but rather under conditions which indicate that they are incorporated within cells or their processes, which can be separated from the cells due to cutting. Are all these substances identical to the granules of Reich? Their morphological features are not completely concordant. Also, their solubility seems different. Placing the formaldehyde slices into alcohol results in a decrease of their amount. I was mostly worried by the observation that a control slice corresponding to Fig. 5, when stained with Scharlach, shows the cells located in the adventitial lymphatic sheath glowing red so that they seem to exist as a red-labeled mass, while we can hardly find any fat labeling in the glial cells of the nervous tissue. Experiments with other material delivered the same results and the content of the glial cells was stained with Scharlach. Yet we know that the π-granules are not stained by Scharlach. It could be considered that the red color predominates and that the basophil metachromatic substances positioned in between are completely masked. Also, in several double staining experiments, there was also a predominant fat staining. One can casually explain these observations that under pathologic conditions substances appear in the glial cells which correspond to π-granules in the peripheral nerves. Often, they have common staining features with those but are morphologically slightly distinct. In comparison to the π-granules, they seem loosened and not deposited in striolated and sharply delineated slabs, but rather in smaller or larger clumps. They are found between cells, but most abundantly within glial cells, in the neighborhood of vessels, and in mesodermal cells of the adventitial lymphatic space. In the latter, they convert into compounds which are not stained by alkaline (basic) colors or are yellowish or greenish labeled

**501**

and which are also labeled red with Scharlach. A large portion of the cell content is stained by Scharlach, and toluidine blue labels a significant amount of basophil metachromatic substances, indicating that many cells contain a mix of both substances. The special property of these fatty substances to appear yellowish or, by application of blue basic colors, to stain green indicates that it is a distinct fatty substance. This tendency is so characteristic that wherever one finds in alcohol preparations such strongly yellow or green labeled masses in cells of the adventitial lymphatic spaces, one can find metachromatic basophil substances in tissue of formaldehyde slices.

We will add a short consideration on the relationship of glial cells to these substances. The appearance of the substances relates to other glial types as the appearance of the above-described granules. In ameboid glial cells, one does not observe them or only allusively. They accumulate in the glial cells and their processes, which appear swollen as we know them from the fatty substances labeled with Scharlach. With increasing accumulation of these substances, the cell loses its processes and becomes roundish. The nucleus moves to the periphery like in fatty granule cells and shows regressive changes similar to these. In the cells loaded with these substances, we observe large chromatin slabs in the periphery of the nucleus while the nuclear membrane appears vague or not stained (Table XXXII, Fig. 9a and 11).

Table XXXII, Fig. 9a Basophil metachromatic inclusions in a slightly growing glial cell in the white matter of the spinal cord of a meningitis due to tuberculosis in the form of Reich´s p-granules. Features of degeneration at the nucleus of the cell *a*; in cell *d* onionskin arranged p-granules.

Table XXXII, Fig. 11 Three glial cells from the white matter of the midbrain of a case of chorea progressive, loaded with basophil metachromatic products.

The observation that we find these substances most abundantly in the cells of the vessel wall and then in decreasing amount in the glial cells around vessels and in the support cells further away from the vessels indicates that these substances or their not yet identified precursors are transported towards the vessels. In the mesodermal cells, if not done already in glial cells, it is completely converted to fat. Which pathologic relevance relates to these substances? We have to search for an answer based on their occurrence in the central nervous system and in different disease processes. One finds those basophil metachromatic

**502**

substances less abundant in grey compared to white matter. I cannot recall that I did find in any case significant amounts in the cortex beyond the radii. One finds them in larger amounts in the white matter tracts. Also in the hindbrain, where they are abundant, their appearance is confined to the white matter and sparse in the grey matter. In the spinal cord, one observes them rather in the white matter as compared to the ventral and dorsal horn. Occasionally one finds them in the grey matter. Yet also this tissue contains quite some myelinated fibers. The arrangement of their occurrence indicates that they have a relationship to white matter. Their presence in the Schwann cells makes this likely and it has already been assumed by Reich.

Considering their appearance in different diseases, it is evident that they do not lack in the normal brain, but they are much less abundant than in the normal peripheral nerves. They are found quite abundantly in some brains which may not be considered pathologic in a narrow sense, such as in old people, in the brains of patients with tuberculosis and also in a brain of a severe anemic they were abundantly present. Larger accumulations one finds in significant mental disturbances. Most abundantly, I found it in an already mentioned case of manic excitation, in depressive conditions of a manic-depressive patient, furthermore in deliria due to infections, in epilepsy, in progressive Chorea Huntington, and in particular masses in the midbrain in dementia senilis and arterial sclerosis. Also, in cases of syphilis-related dementia, meningitis due to tuberculosis, in the neighborhood of tumors, and in local degenerations and bleedings one finds them occasionally. Sometimes one finds ameboid glial cells next to it and the appearance of a decay of the nervous tissue. In some cases of severe cortical illness with multiple decay of axons, one does not find any trace of them. Their appearance seems not to be connected to a defined disease form and not necessarily to a decay of myelinated fibers. At regions where myelinated fibers are degraded in larger amount, such as at lateral strand degeneration after bleeding and where very many fatty granule cells

**503**

were found, there was little or nothing of them detected, similarly in a case of paralysis, where lateral and ventral strands were densely filled with granule cells. Unfortunately, more extended, comparative investigations are hampered by the fact that there is a rapid decay of these substances in formaldehyde preparations. I did not succeed, using the same material several months later, to stain the basophil metachromatic incorporations while they could be nicely illustrated earlier.

I have also tried to understand the importance of these substances using animal experiments. It was surprising how little I could find in some animals. Neither in the healthy nor in the experimentally damaged rabbit brain could I ever find traces of these substances, and also not in the Schwann sheath of the peripheral nerves. Also, in many other animals, I could find them only in low amounts or in forms with staining reactions which were not compatible with the features of π-granules in peripheral nerves of humans. My studies are not so extensive that I could claim that they are not at all present in vertebrate animals. Yet it is beyond doubt to me that they play a less important role. It still seems necessary to extend research in this direction, so that we can primarily determine the importance of these substances based on animal experiments.

Until those studies are performed, we have to reach at least a preliminary conclusion. Since it is evident that the metachromatic basophil substances may be absent or present in only low amounts wherever myelin sheath is degraded, they are thus not a degradation product of the myelin sheath, like the lipoid substances labeled with Scharlach. Since they can be found at locations where we have no evidence that the myelin sheath is degraded, it is likely that they appear without degradation of myelin. Since they increase in pathologic cases, one can assume that they are generated at least in part...

**504**

under pathologic nutritious conditions. That they are degradation products in a broader sense is indicated by the fact that they are converted to fat and delivered to the vessels similarly to other degradation products. These substances would therefore have particular relevance and should be of particular interest, since they are an example of a delicate disturbance of the nervous system for which we lack an anatomical understanding. The studies of Reich might provide progress for a better understanding.

I can, however, not concur with the conclusions which Reich made based on his studies. What glial cells in the nervous system provide is provided by the Schwann cells in the peripheral nervous system with modifications. They form a cover and support substance, participate in the formation of the myelin sheath, support its nutrition, and clean up those which degenerate. They are in close biological connection, in a symbiotic relationship with the peripheral nerve fiber. While distinct granules can be found in Schwann cells, this does not relate them to ganglion cells. The types of granules correspond rather to a biological relationship to glial cells. The findings of Reich and our own observations indicate that the Schwann cell can be considered as the glial cell of the peripheral nerve — as it is also based on developmental studies of its origin.

**6. On simple basophil degradation products.**

Besides the basophil metachromatic substances, we find in different acute diseases of the nervous system other basophil substances, which are distinct from those based on their staining and morphological properties. After fixation with alcohol and staining with basic aniline colors, we find products which have acquired the corresponding stain without any tendency to metachromasia and are therefore

**505**

considered as simple basophil. Their tendency to be labeled by basic colors, like the Nissl substance, makes them difficult to study. When they appear in ganglion cells, they are very difficult to distinguish from the Nissl substance. All trials to stain them selectively have failed. One finds the simple basophil products quite frequently in acute diseases and in experimental intoxications. They obviously appear already at subtle damages of the ganglion cells but also accompany very severe degeneration processes of nerve cells, such as the severe Nissl cell disease. Their appearance at light disease states can best be studied under experimental conditions. After subacute, maximal alcohol toxification in rabbits, for instance, one finds in the central nervous system protoplasmic processes hardly stained but characterized by rows of dark blue granules. They extend further through the nervous tissue than one normally can follow the protoplasmic processes in the Nissl preparation. Another fortunate object is dogs which have died of tetanus after removing the thyroid gland and the epithelial bodies. The first assumption on the nature of these granules is a conversion of Nissl substance into granules. Against that is the finding that these granules are observed at locations where no Nissl slabs are found, such as in the protoplasmic processes far from the cell body and in the axon. Some of the granules are not within the cellular processes, but on the cells and around them and at a distance from the ganglion cells in the tissue. Occasionally, one observes quite delicate protoplasmic processes and axons which seem to dissolve into those rows of granules. They had been proposed to me by students in the lab as bacteria. We observe such substances also outside of ganglion cells; we find them in glial cells and often in the perivascular spaces, often composed of larger clumps. Corresponding substances in much larger amounts, we find in different severe psychoses.

*Table XXXIII, Fig. 7 Beetz pyramidal cell (29) of a case of delirium after acute paralysis. Nuclear alterations of a severe cellular illness of Nissl. At the top process and at an adjacent protoplasmic process are basophil granules. At the base of the cell and at the axonal process are drop-like accumulations of simple basophil products. Also, at the cell body of the ameboid glial cells which are around the ganglion cell.*

**506**

On top at the process and at a close by protoplasmic process which probably belongs to the cell are almost round, basophil granules. It is difficult to decide whether they are within the protoplasm or at it. Further below, we observe granules which are certainly outside of the cell or attached to it. At the base of the cell around the axon and its axon initial segment and even above are large slab- or drop-like formations of the same substance. Along the cell body, they become smaller. It is impossible to decide whether these are altered Nissl slabs previously in the cell or pathologic products deposited outside of the cell. One has to find a staining procedure which differentiates these two substances by staining. The same substances we also observe at the ameboid satellite cells which surround the ganglion cells. In other cells, they are certainly also within the cytoplasm of the glial cells.
Fig. 12 of the same Table shows us the same substances from a case of a uremic delirium: at a) we observe bacterial-like rows; at c) a part of a cytoplasm of a ganglion cell which dissolves into those substances; at d) another part of a cell; at b) a round clump from a perivascular space in which the same basophil substances are embedded into an almost unlabeled homogeneous basic mass.

Table XXXIII, Fig. 12 *a,* simple basophil granules originating from the decay of a protoplasmic process of the ganglion cell arranged in coccus-like rows. *b,* pile of basophil granules from the perivascular space. *c,* piece of a cell body of a ganglion cell in which basophil granules dissolve. *d,* rest of a ganglion cell disassembling into basophil granules. From a case of uremic psychosis.

In contrast to the metachromatic basophil substances, which we predominantly find in white matter, we find the simple basophil predominantly in grey matter, preferentially around the ganglion cells and their protoplasmic processes. One has to address the question whether these are products which are deposited from the tissue fluid at these locations or whether they are generated by degradation of existing tissue structures. In many cases, one can clearly observe that at the location of their appearance the protoplasmic processes show severe damage and the ganglion cells show signs of dissolution. Images like Fig. 12c show clearly that they are generated due to a disintegration of the cellular cytoplasm. The possibility seems not excluded

**507**

that they are generated from a decay of pericellular nervous structures at positions where they are attached to intact protoplasmic cellular processes and cells. I did not succeed in displaying them in material fixed in formaldehyde, nor with the acidic fuchsin light green method on slices of Flemming. Thus, it is questionable whether they exist in life as we see them in alcohol slices or whether they are generated in that form due to fixation in alcohol. Moreover, the fact that we find them as drop-like formations, as shown in Fig. 7 on Table XXXIII, might indicate that during life they are in a fluid or semi-fluid state.
It is not easy to determine the relationship of these basophil products with the glial cells. One finds them often surrounding the glial cells, yet it is often difficult to decide whether they are on or within the cytoplasm. Occasionally one finds glial cells which contain these substances in their cytoplasm. Nevertheless, they seem to find their way to the perivascular spaces, where they accumulate as very large masses. Also here, we often find them embedded in larger clumps which are less stained. They do not seem to transfer to mesodermal cells in the form observed in the nervous tissue. Yet at positions where they are quite abundant, the cells of the adventitia are heavily loaded with lipoid substances. Thus, it is possible that they are converted into fatty substances within these cells.
Such simple basophil substances are found not only in acute disease states, but sometimes also in entirely degenerated cortical areas or around ganglion cells which are completely dead. They frequently show angular, sharp forms and it requires further investigation to determine whether they are identical to those found in acute diseases or whether they have shrunk during longer deposition and become tighter, or whether they are of a different kind. In part, these two forms of basophil products, in particular the latter, might refer to what Nissl described as 'visualization of pericellular pants.

**508**

Besides the ones just described, we find several other basophil inclusions in the glial cells of diseased nervous tissue: those which have no tendency to take up a metachromatic stain and those which are slightly metachromatic stained, yet not as intense as the true metachromatic basophil. Occasionally there are single, round granules of different sizes which are located in roundish cells. There is often only the nucleus and the stained cell border visible, while there is a large gap around the nucleus (Table XXXIII, Fig. 3 h-m); in other cases, these are slab-like formations or more foamy structures (Table XXXIII, Fig. 3 a-g).

Table XXXIII, Fig. 3 Different forms of degenerative altered glial cells from the cortex and the white matter of different cases of delirium after sepsis. In the cytoplasm of the cells are many basophil metachromatic granules and substances.

Sometimes they are found in significant numbers, in particular in the cells of the white matter, and since one finds similar incorporation in almost all cells, they might have a special pathologic relevance. At present, one cannot state anything specific.
Finally, to these belong also the strange stippchen (30) highlighted by the Nissl method, which one finds in proliferating glial cells and glial lawns to which Nissl has alerted us. As for the relevance of these cytoplasmic incorporations, I do not know anything except that they are different from the previously mentioned.

**7. On some extracellular pathologic metabolism products.**

Earlier we have recognized a form of extracellular formations in the acute diseased nervous system and its appearance was closely related to the presence of ameboid cells. We have considered it as degradation products of glial structures – the filled bodies. Besides those there are several other extracellular pathologic formations. Some show a wide-spread presence and are obviously not connected to acute disease processes and the presence of ameboid cells.

In the analysis of these extracellular formations, one has to raise the question whether they exist in live as we observe them in our preparations

**509**

or whether the fixation process transformed them from a fluid state into a solid form. To decide this question, it is of great importance to study their relation to glia. If we observe that the glia treats them as foreign material and forms its own structures around them, then we can conclude that they existed in life in such a form as we find them in our investigation. Should the glia lack any reaction to them, then the question remains open. There may exist substances which are so little attractive that they do not respond to them, yet this may be rare.
The best-known of these metabolic products of the central nervous system are the corpora amylacea (31), which we will briefly describe. There is a rich literature on that topic and there are constant efforts to determine their origin from elements of the central nervous system. The corpora amylacea are found in the normal nervous system, in particular below the surface layers of the glia, certainly strongly increased in the chronically degeneratively altered nervous tissue: in the dorsal demyelinated strands, in the border layers of the spinal cord of senile dementia, in the thickened mesh of the cortical surface, in the neighborhood of arteriosclerotic foci, and around the degenerated vessels of the basal ganglia during arteriosclerosis. Here one finds excessive accumulations. They can often form quite rapidly, and one observes them in meningitis due to tuberculosis in the spinal cord of only a few days' duration. They are present in large amounts in areas which had undergone the strongest alterations due to the meningo-myelitic process. In a case of severe delirium due to intoxication which lasted only a few days, they were at many locations in the cortex in several layers, one on top of the other, at the glial border layer. Between two glial endfeet which inserted into the border membrane was such a corpuscle. They also belong to the picture of some acute disease processes of the nervous system. In such acute cases, one finds the corpora amylacea between the remnants of decayed axons, even in the middle of the granular mass in which they dissolve. One often finds them in the protoplasmic body of proliferating ameboid glial cells.

**510**

Are they generated from axons or glial cells? Their accumulation below the membrana superficialis of glia, their massive abundance below the membrana perivascularis of larger vessels (text figure 8), excludes the conclusion that they originate from nervous elements, as Obersteiner already emphasized.

Text Fig. 8 Accumulations of Corpora amylacea around a vessel of the thalamus opticus from a case of arteriosclerosis. Weigert´s iron hematoxylin, von Gieson staining.

Just there, in the glial border zone of the cortex, where they obviously appeared very acutely in large numbers, one did not find a single one within a glial cell. This makes it unlikely that they originated from glial cells. The finding that they are deposited at the border membrane in large numbers, which can be considered as a filter membrane,

**511**

indicates that they originate from tissue fluid as a precipitate. The glia treats them as it handles any other foreign body. If they are found within glial cells, then they have been incorporated. The most obvious glial border membranes which can be found are the delicate hulls in which the glia embeds the amyloid bodies. Using acidic fuchsin light green preparations, one can demonstrate this very nicely, since the corpora amylacea are labeled green and the border membrane in red (text figure 9A). Sometimes also glial fibers contribute to the encapsulation.
Obviously, the amyloid bodies undergo further chemical conversions as soon as they are deposited in the tissue. This illustrates their extraordinarily distinct response to different staining methods. Using basic Anilin staining, they are either not labeled, or weakly, or even very dark. Using hematoxylin, they sometimes hardly label, sometimes deep blue. One often observes that they incorporate different colors in their various layers. Therefore, we should consider the corpora amylacea as products which precipitated from the tissue fluid already in the living state. Their basic relevance remains unclear.
If we look further, we find in the pathologic nervous tissue several deviating but similar things. In Nissl preparations we often observe small, mainly roundish, sometimes oval, more rarely irregularly delineated bodies which are widely dispersed in the entire tissue. They are hardly of the size of a ganglion cell nucleus, sometimes label intensely with basic stains, sometimes are hardly stained. They do not show the features of the corpora amylacea. A few times, I have observed them in large numbers in the dorsal horn of the spinal cord from an alcoholic; they are also in the cortex of fresh and older paralysis. As far as can be seen, the glial cells seem not to pay any attention to them. Maybe they precipitate due to the fixation process.
Most likely, it relates similarly to the glycogen, which I found several times within and below the pia, sometimes in the cortex, partially enclosed in glial cells, often only close by.

**512**

Mainly I found it in the adventitial lymphatic spaces as numerous granules or drop-shaped formations, in particular in paralysis and infectious deliria. Recently Casamajor in the Obersteiner lab could demonstrate its presence in the ganglion cells of the cortex. The relevance of the glycogen and its occurrence in different disease states needs to be clarified in future studies.

**8. Strange degradation products of special disease processes.Problem here**

The pathologic substances which we have discussed so far are wide-spread at different diseases of the central nervous system and can be considered as common indications for the degeneration of the nervous tissue or pathologic metabolic disturbances in the central nervous system. Others maybe restricted to single disease states or a group of related diseases. We have to assume that the degradation process deviates from the common form. So far, we know that only from the amaurotische cretinism and a single paralysis-like disease state which was studied by Dr. Baroncini in our lab belongs into this category

Text Fig. 9. Different extracellular pathologic formations. A amyloid bodies surrounded by a glial envelope. B, C Frequent formations between myelin sheath. D Formations filled with fuchsinophil granules. E Peculiar substances deposited in slabs or clumps in the wall of a capillary. Acidic fuchsin light green. The dark areas in the preparation are stained red, the light in green.

The drusen, well-described by Fischer also belong to the pathologic formations which he considers characteristic for the senile dementia. As shown by the studies of Perusini and of Simchowicz in our lab on senile dementia, the essential component of the drusen is the deposition of a pathologic metabolic product into the previously altered glial reticulum. The alterations in the nerve cells and the axon may be considered as secondary. The glial cells in the neighborhood try to encapsulate these deposited foreign substances and as a result, drusen are formed which are a complex formation which are not deeply understood yet. Since those studies show that these substances show other staining reactions as compared to all other pathologic

**513**

substances which we know so far from the pathologic anatomy of the brain, we have to consider that they are products of a very special type.

For some other substances which are found less consistent and rarer it needs further studies to determine their relevance. One often observes round clumps in acidic fuchsin light green preparations, particularly in the cortex. They are of the size of a middle ganglion cell and consist out of very small, red-labelled granules of irregular form and size which are compacted. Other of those clumps show a greenish base color, but are also spread over with very small red granules. Their form is irregular, sometimes longitudinal, biscuit-shaped, often one finds in the neighborhood of the large clumps smaller ones which are characterized by a large assembly of small granules obtaining a red color. Other formations remind in size and form on large ameboid cells, are yet more angular in their outline. The periphery is stained green, the center is formed by a dense accumulation of red granules and in between a green staining cannot be recognized (text figure 9D). I could never find a nucleus within those; I do not assume that they are decaying ameboid glial cells since they are much larger than all glial cells which I find in this preparation. Relatively abundant are other formations which I do not understand. They are found frequently between myelin sheath and they can reach the diameter of the largest of those, but can also be very small (text figure 9 B, C). Their form is quite irregular, rarely round, mainly more square, often with impressed laterally. They are labelled with the acidic fuchsin light green stain dirty green and contain mostly at the edge, sometimes also in the center red dust grains. A nucleus cannot be recognized. Probably something else represent piles formed from green and red masses of granules which are deposited around vessels, sometimes penetrating the enlarged vessel wall (text figure 9E). If one wants to study the generation and relevance of these last described formations, one would have to apply other staining methods to determine whether they behave similar with respect to the other staining mixtures

**514**

or whether they are of a different kind – what seems most likely to me. Their rare abundance indicates that they have a peripheral interest for the pathologic metabolism of the cortex. It maybe enough to emphasize that a number of other products are formed at the degeneration of the nervous tissue about which we know little today.

**8. Peculiar degradation products of particular disease processes.**

While the pathologic substances which we have discussed so far are wide-spread at different diseases of the nervous system and they should be considered as a regular event of the degradation of the nervous tissue or pathologic metabolic disturbances of the central nervous system, the appearance of others maybe restricted to single diseases or a group of related diseases. We must therefore assume that the degradation process occurs in a form which deviates from the general one. So far we know only the amaurotic idiocy (26) and a single paralysis-like disease case, which has been intensely studied by Dr. Baroncini in our laboratory. Potentially, a case of congenital cerebellar atrophy described by Sträussler might also be considered. Moreover, I have seen from some other cases which are clinically different, single preparations which seem to contain different substances or in a different appearance. Yet I do not have sufficient material to analyze this in depth or they were available at a time when I did not focus on such cases and these preparations have become unusable. It is thus likely that future studies of mental disturbances, in particular the innate idiocy and related, will be characterized by distinct substances related to these diseases. It is also not unlikely that substances which correlate to a single disease

**515**

may be found in related diseases, potentially in varying arrangements.
We know two variants of amaurotic idiocy: one first described by Warren-Tay-Sachs and, with respect to its histological peculiarity, studied in detail by Schaffer; the other first described and anatomically investigated by Spielmeyer and further described by Vogt. Characteristic for the Warren-Tay-Sachs form is the massive appearance of granules of a peculiar substance in ganglion and glial cells, which represents the lipoid substance found in the fatty degeneration of ganglion cells. The cytoplasm of the ganglion cells transforms by massive swelling of the parts that contain this substance. We observe shape changes of the ganglion cells similar to those in usual fatty degeneration, but less pronounced: pear-like swelling of the cell body, sac-like protrusion of the cytoplasm, spindle-shaped swelling of the cytoplasmic processes and axon, translocation of the nucleus, appearance of a cytoplasmic scaffold within the cell at the deposition area of the granules in the Nissl and Bielschowsky images, marginal position of the fibrils, formation of basket-like fibrillary scaffolds at the surface of the cells and swelling of the cytoplasmic processes and axons, and finally the conversion of the entire ganglion cell into sacs filled with these substances and disintegration of the cells. Besides a generation of giant fiber-forming glial cells and delicate fibered glial arrangements throughout the cortex, we also see accumulations of the same substances in the glial cells as in the ganglion cells. Many of these convert into granule cells, while others have these incorporations only in the periphery of their strongly enlarged cell body. Besides that, there are numerous granule cells in the adventitial lymphatic sheath, which is often enlarged and has become complex due to numerous connective tissue septa.

**516**

The Spielmeyer-Vogt variation differs due to the presence of a lower mass of these substances. Several other differences in the histologic changes, which interest us less here, justify defining it as a special form of the Warren-Tay-Sachs case. Many clinical and anatomical similarities support combining these into one group of amaurotic idiocy.
With respect to the alterations of the ganglion cells, one may consider it as a fatty degeneration, which is similar in features to senile fatty degeneration (as mentioned above) and only exceeds in magnitude. A closer inspection indicates that this substance differs in some aspects from the lipoid substances which we usually find in ganglion cells. Already at the first material I received due to the courtesy of Prof. Schaffer six years ago, I noticed that I did not find a usual mass of pathological incorporations labeled red with Scharlach in the ganglion cells using the Herxheimer method. Besides sparse small fatty bodies, which did not correspond to the granular incorporations, there was at most an undefined dull red staining of the remaining pathological cell content. In contrast, substances stained with Scharlach were abundantly incorporated in the glial cells and in the cells of the adventitia. Table XXXIV, Fig. 5 shows such an image.

.

Table XXXIV, Fig. 5 From a slice of the cortex from a case of Warren-Tay-Sachs amaurotic idiocy. In the ganglion cells little dust-like distributed fat, in the glial cells, large fatty piles, in particular large accumulation of fat in the adventitia.

The cells which appear like being dusted with fine fatty granules correspond to granule cells; the cells with the big fatty drops are the glial cells, and moreover, one finds fat accumulations in the cells of the adventitial lymphatic space. Very similar are preparations...

**517**

treated with osmium after previous chrome incubation. In the ganglion cells there were only sparse or even no small fatty drops, more in glial cells and abundant in the cells of the adventitia. The incorporations in the ganglion cells, which do not stain with Scharlach, could illustrated with different other methods. Particularly brilliant were images produced with method III. Text figure 10 shows such an image which I obtained from the Spielmeier-Vogt form of amaurotic idiocy based on material which I received due to the courtesy of Prof. Vogt.

Text Fig. 10. Elective illustration of the granules in a case of Spielmeier-Vogt amaurotic idiocy with method III.

The incorporations in the ganglion cells become apparent in much larger mass, often filling the entire cell body, sometimes also the processes are lightning blue stained. In the glial cells we see the same substance in the form of granular clumps positioned around the nucleus. At the Sachs form they are even much more abundant. They fill the entire ganglion cell and some glial cells, which have acquired granular cell forms. In other, large fiber-forming glial cells they are in the periphery of the protoplasmic cell body. In different areas they seem to be embedded as small granules in a net which might represent the glial reticulum. Furthermore, I achieved with modifications of the Weigert myelin sheath staining to almost selectively label these incorporations. The text figure 11 shows a photography of such an image.

All the dark clumps are cells loaded with these substances. This massive amount in which these substances are found in the nervous tissue is illustrated in this case of the Sachs form. At the material of the Sachs form which I obtained from Prof. Adolf Meyer in Baltimore and which is from a progressed disease state, there were more substances in the ganglion cells stained with Scharlach red. But they also did not match the amount of strongly refractive granules which were found in the cells. Here the substances were often not found in the form of granules, but rather in striatal formations, often onion shell-like composed in large, roundish conglomerates like the p-granules of Reich. This the material was for a longer time embedded in formaldehyde; it cannot be excluded that these are modifications

**518**

which were generated later in the conservation fluid. One has to confirm on fresh material whether these morphological peculiarities are not artefacts; in general, it seems to be a feature of these substances to slowly convert in formaldehyde over time and loose some of their ability to be stained.

Text Fig. 11. Selective presentation of the incorporations of the Warren-Tay-Sachs amaurotic idiocy. Cortex. Weigerts myelin sheath staining. Method VIII.

Due to the rarity of that material to determine the specific features of these substances. Furthermore, their illustration could be achieved with the method described by Benda for labelling glial fibers. Some of the granules are red, some blue

**519**

labelled while in others a red cover and a blue central mass could be recognized.

Spielmeyer has extensively studied his form of amaurotic idiocy. He also highlights similarities with the senile fatty inclusions, mentions also the yellowish label, but emphasizes that the lack of the osmium reaction argues against an identity of both forms of incorporations. Finally, Schaffer introduced a very simple staining method consisting of a brief staining of the formaldehyde slices with Ehrlich’s hematoxylin. All cells containing these substances are stained dark blue. On my old material, one did not observe clear granules after a short staining at many locations, but just a cloudy blue mass. If one stains for several hours, the single granules become more visible. Treating these formaldehyde slices first with Ehrlich hematoxylin and then for half an hour with alcoholic Sudan solution, one observes that the ganglion cells are labeled completely blue with only a few red granules in the blue mass; in contrast, the glial cells show, besides blue granules, many larger red ones, some even entirely stained red. Often one or more entirely red-labeled glial cells are embedded into the cell body of a blue-labeled ganglion cell.
If we apply the same method to other cortical preparations, such as in senile dementia, we find different staining reactions of the lipoid granules. They stain with some modifications of the Weigert myelin stain but not with the May-Grünwald staining or with Ehrlich’s hematoxylin. In contrast, the granules of amaurotic idiocy do not stain with Scharlach or osmium after chrome treatment, while they stain intensely in senile dementia. Using the carbol fuchsin-methylene blue staining, which labels the lipoid granules of the senile ganglion cell intensely red, the incorporations in the ganglion cells of amaurotic idiocy are only lightly labeled red, or sometimes they do not take up any stain. In contrast, the granules of the glial cells are intensely labeled red, and the glial cells converted to granule cells are bright red. Using the fibrin method, I could not label the granules of amaurotic idiocy.

**520**

Despite that not all these reactions could be performed on fresh material, and thus should be confirmed on that, there are substantial differences indicating distinct chemical properties. Based on the similar reaction to staining, it seems likely to me that the two forms of amaurotic idiocy are of the same type. The fact that at the Sachs form the granules are commonly not labelled yellow and are easier stained with Weigert´s hematoxylin seems to me not relevant, since I observed in my material of the Spielmeier variant of the amaurotic idiocy many unpigmented cells with incorporations. I could stain those in blue with the above mentioned Weigert myelin stain.
What are these substances? If we place a slice fixed in formaldehyde for one hour in absolute alcohol heated to 50°C, one finds instead of a pile of blue labelled granules mainly only empty bags. The mass of pathologic incorporations is also significantly reduced after incubation in ether. This would indicate that it is a substance of a lipoid nature or closely related.
Of particular interest is the finding that incorporations which we find in glial cells are significantly more darkened with Scharlach and osmium. This is even more pronounced in incorporations of the adventitial cells. There are obviously similar relations as in the basophil metachromatic substances which are converted in the glial cells and even more in the adventitial cells into pure fatty substances. This is a further indication for a transport of these degeneration products towards the vessels and their further degradation during that passage.
One therefore might consider that the characteristic substances of the amaurotic idiocy are precursors of the lipoid granules of the common fatty degeneration of the ganglion cells which have not fully converted to the common fatty products under these specific pathological conditions. My preparations do not provide a final conclusion. In some cells they are labelled

**521**

by acidic fuchsin light green. Yet my material was not fresh enough that I can make a final conclusion. I would also like to remark that we observe fuchsinophil granules as precursors of lipoid substances at the coarse granular fatty degeneration of the ganglion cells which are labelled by Ehrlich´s hematoxylin. There may be an opportunity to finally decide this question on the basis of fresh material which should be of significant interest. So far, we can claim that the substances of the amaurotic idiocy are distinct from, but related to those found in senile dementia and common fatty degeneration of the ganglion cells. We cannot yet decide whether it is an intermediate stage of the same pathway or a different degradation pathway.
As Spielmeyer has already emphasized, the incorporations in the Sträussler case are distinct from the amaurotic idiocy due to their tendency to label black after chrome treatment with osmium. Sträussler himself considers the incorporated granules identical to the incorporations of the common fatty degeneration of the ganglion cells. The images of the cells and the massive swelling of the processes remind one of cells of the amaurotic idiocy as already emphasized by Sträussler. If this should indeed be simple fatty incorporations, then they would result in a deformation of the cells as never observed before at that degeneration. It would be necessary in a new case to focus on the staining properties of the substances to determine whether these substances are identical with those of the common fatty degeneration or rather have a relation to the amaurotic idiocy, or even are distinct from both.

**522**

of the white matter. Besides a strong and a more or less diffuse atrophy of the white matter, in particular of the white matter tracts and in acute Marchi degeneration, one finds numerous, large glial cells, partially round, partially in the form of large, plasmatic, fiber-forming cells. They were stuffed with substances which yielded an excellent metachromatic staining with toluidine blue. Based on the restriction to the white matter and the deeper cortical region containing many myelinated fibers and based obvious staining reaction it is related to the above-described basophil metachromatic substances which we often find in white matter. In other features, it was different. They are found in accumulations and in a form of giant cloudy masses as not observed elsewhere. In older cells they show with thionin staining a peculiar rust-colored metachromasia. With Ehrlich´s triacid, they are labeled shining green. Also here, one could clearly follow the transitions of these substances into true fatty within the glial cells and in particular in the adventitial cells and a transport towards the vessels.
It seems to me that in particular these diseases with special degradation products, provide us important information of the entire degradation process and the role which glial cells play. It would make sense to make a chemical investigation on these massively appearing pathologic products. This could result in insights on the form of deviation of this degradation process and the nature of these diseases more than based on staining procedures. It will be useful to search for other related disease types. Based on the methods which we have so far used, these pathologic products do not become apparent. It would make sense to try all methods or even use new ones.

**9. The different forms of degradations of the nervous tissue**

After it has become obvious to us that the biological importance of the ameboid glial cells is closely related to the degradation of the nervous

**523**

tissue, it will result in an even better understanding of their role by analyzing their relation to other degradation processes of the nervous tissue.

If the nervous tissues together with the support tissue is completely destroyed, may it be to a trauma, a bleeding or a softening, we observe that in the first place the mesodermal elements fulfill the task to remove the dead nervous substance. From the rim of the focus, newly formed vessels invade into the dead tissue; granule cells formed from the cells of the vessel wall migrate into the environment, take up part of the dead material and convert it to fat. In the meantime, the glia proliferates around the focus, but only contributes to the cleaning process at areas where degradation products are generated within the still remaining (living) nervous tissue. It eagerly forms new fibrous glial tissue which encapsulates the focus. Finally, the fatty products in the granule cells are dissolved and with the granule cells degenerate. From the focus remains the newly formed vessels, the fibrous connective tissue and the massively formed glial surface layer: mesodermal type (Schröder).

It is different, if the damage selectively affects the nervous tissue resulting in a larger degradation and degeneration, while the support tissue is not affected. Now the glial cells fulfill the cleaning process from the beginning. The glial cells convert into fatty granule cells by circumventing the white matter clumps which are generated from the decaying myelin sheath. They dissolve those and convert it within their cell bodies into fat. Only later, also mesodermal cells participate in the cleaning process. The fat which we find first in the glial cells, we will soon observe also in cells which reside within the adventitia and the pia, later exclusively in those. It migrates from the ectodermal to the mesodermal tissue. That this occurs that way is obvious , when comparing images at different stages of a given process in which gliogenic granule cells appear . With the reduction of the fatty granule cells in the nervous substance, the fatty granules increase in the adventitia and in the adventitial lymphatic space. How these proceeds

**524**

in details is not yet fully clarified. One can observe that the glial granule cells extend halfway into the perivascular space. Around the vessel there are several rows of granule cells separated by small connective tissue-like septa. One may consider that the glial granule cells migrate into the perivascular space and are there enwrapped by the adventitia. Yet since one cannot distinguish granule cells of mesodermal or ectodermal origin, this is not conclusive. Other observations indicate that the fat from the ectodermal cells is transferred to the lymph in an altered form and from there taken up by the mesodermal cells. One can observe that wherever gliogen granule cells reside in the nervous system, the cellular elements of the adventitia enlarge and that the enlarged cell body acquires a flabby appearance. This cell separates from the tissue and converts to a grid cell or granule cell. Occasionally, macrophages of the lymphatic space undergo the same conversion. At least here, we observe an interaction between ectodermal and mesodermal cells. As soon as the clearance is in full action, one can observe that other glial elements form glial fibers and how the new formation of glial elements makes progress until finally the entire nervous tissue is replaced by a fibrous glial scar. This ectodermal form of degradation we observe at different processes (Schröder), most typical in the strand degeneration of the spinal cord, at the so called lobal sclerosis at at the paralysis after apolectiform seizures mainly with focal restrictions in the white matter. The second form of degeneration is not fundamentally different from the first. In some forms of encephalitis, for instance, one finds both forms of granule cells next to each other.

Besides those degeneration processes known from older and more recent studies (Farrar, Schröder, Merzbacher) represent the previously described degeneration processes which are associated with ameboid cells, a third distinct group. Also here nervous tissue degrades, but to a much smaller degree and more dispersed as the other modifications. Also here

**525**

it is an important issue that the formation of those ameboid cells and their decay can be quite significant without a parallel or subsequent fiber formation of the glia. We observe at dementia praecox, at epilepsy and some other disease processes during the acute progression of the disease a massive appearance of ameboid cells in the cortex without augmentation of glial fibers. At other processes, in contrast, the appearance of ameboid cells is accompanied by a formation of glial fibers, such as in paralysis. The ameboid glial cells disappear soon and the glial fiber formation remains. Thus, such disease states result in different images as one gets them in an acute state or in a chronic investigation. Also, this third modification of the degenerative process (type of the ameboid cells) is not fundamentally different from the previous ones and we find ameboid glial cells besides gliogen granule cells, for instance at the progressive paralysis, in different forms of meningomyelitis, in the neighborhood of foci and tumors.
But not everywhere where the nervous substance degenerates, one finds ameboid cells. They seem to be present at a rapid decay and maybe not at all instances. Usually, we find at chronic degenerative processes and also quite often besides

**526**

the ameboid cells different types of glial cells which have incorporated more or less masses of lipoid products. They are often positioned around the nucleus of the glial cells, in other cases in a bag which is formed by the transformation of one of the processes (Table XXVIII, Fig. 1c).

Table XXVIII, Fig. 1c, glial cell from the cortex of a 68-year-old man diseased with arteriosclerosis.

One also finds cells which have two or three of such bags while the other processes have formed delicate, branched protoplasmic structures or also glial fibers. Occasionally it occurs that such glial cells completely convert into small fatty granule cells. Sometimes only one part of the cell serves for the fat accumulation, while the remaining part is available for other functions. Regularly, one finds the cells of the adventitia abundantly loaded with fat and frequently one can make the observation that the glial cells in the neighborhood of the vessels show abundant fat incorporation as compared to those more apart.

With a lower intensity of the disease process the difficulties increase to follow the relations such as how the lipoid substances enter the glial cells or the cells of the aventitia. The evidence is in favor for the assumption that at this fourth degeneration process, the resident glial elements are sufficient to preliminarily take up the substances formed by the degradation of the nervous material and subsequently pass them on to the mesodermal elements. Special forms of glial cells are not generated.

At a lower degree we observe this fourth type of degradation type in all brains of adult humans and it is likely that the degradation process of this low grade is within the normal physiology. If we would consider the presence of lipoid substances in ganglion cells, glial cells and adventitial cells as pathologic, then there would not exist a normal human brain.

Besides the different types of degenerative processes, we have observed other events which are more or less independent from those, such as the processes by which simple basophil products are generated. We have to consider this process as a special form of dissolvement of protoplasmic cellular substances. It is distinct

**527**

from other widely spread, difficult to analyze dissolvement processes. It delivers a simple basophil product which can be partially followed up to perivascular spaces where it dissolves. It appears probably in the cells of the vessel walls converted into fatty substances.

Then we have learned about a special product, namely the metachromatic basophil products. It is certain that it has a relationship to the white matter, despite the fact that it only can be verified as an inclusion in the glial cells. We have seen that these substances accumulate in particular in the glial cells around vessels and then are transferred to the adventitial lymphatic sheath. There they are converted into fatty substances and it remains open whether these substances were generated by a degradation of the myelinated fibers or possibly generated due to a disturbance of the nutrition of those.

Finally, we have observed that at particular disease states, substances appear in large amounts which correspond to the lipoid substances of the ganglion cells at normal degeneration processes (amaurotic idiocy) and substances which are rather related to the basophil metachromatic. Also, with those we observe that they are partially in glial cells, partially in cells of the vessel wall converted into simple fatty substances. On the nature of other degradation products, we have little insight due to a lack of appropriate specimen.

After all these observations it is evident that many degenerative products which are generated in the nervous tissue are transferred via the different forms of glial cells to the mesodermal tissue where they finally appear as fatty substances. The original products which appear in the ganglion cells, in the myelinated sheath and in the glial cells show several staining and probably also chemical variations. They become more uniform the closer they get to the vessels. Thereby we can better understand some of the regressive changes in the glial cells. The cells show degenerative features as soon as they are excessively loaded with pathologic substances and after they have fulfilled their task, their short developmental process comes to an end. It now becomes evident why such far reaching relations

**528**

between the changes of the ganglion cells, the changes in glia and vessels are present. They are in close symbiotic relationship and the damage in one result in a conversion of the other. Today,we are able to conclude that if we observe alterations in one of these elements with one analysis method, we can predict that other methods will reveal alterations in the other elements. This results in a better understanding of detailed pathologic processes in the central nervous system. We do not only know that certain alterations in the nerve cell result in certain changes in glial cells, but we know why. In this study I have only dealt with a small part of those relations. It will not be difficult to align others. Just to refer to one example, it is obvious in the process which is generally summarized under the name neurophagy which has been studied in the last years and lately well described by Marinesco.

We observe an obvious accumulation of glial elements around ganglion cells under different conditions, at older people, in the brain of a case of phthisis (32), at some light psychotic conditions while the ganglion cells do not show any changes in the nucleus or their cytoplasm. With our analysis of the glial elements, we do not observe an enlargement of the glial cell body and no included granules. It leads to the conclusion that this is not a case of neurophagy, yet we do not understand the relevance of this glial accumulation at the ganglion cells.

In other cases, we observe that strongly degenerated, and in its substance converted ganglion cells are enwrapped by delicate, skin-like formations from the surrounding glial cells. On can observe that in particular at calcified ganglion cells. Also here, we observe none or only few granules in the cell body of the glial cells. Thus, it seems not a neurophagic process, but rather a kind of

**529**

funeral, like an encapsulation of dead ganglion cell bodies which have become foreign to the nervous tissue (Table XXXIII, Fig. 15).

Table XXXIII, Fig. 15 Three coffin embedded, dead ganglion cells with severe nuclear alterations and almost unstained cytoplasm enwrapped by glial cells which are not of the type of ameboid cells (coffin formation). From the white matter of a case of endarteritic syphilis (33) related dementia with many foci.

Finally, we find glial cells of ameboid character with fuchsinophil granules and lipoid bodies around ganglion cells which show substantial alterations in their form and cytoplasm. The glial cells sometimes penetrate into the ganglion cells, sometimes only a finger-like pseudopodial process of the cell body attaches to the ganglion cell. It seems to dissolve it. This could be a true neurophagy, by which the glial cell contributes to the dissolvement of the ganglion cell and assimilated substances which are generated due to the dissolvement. This true neurophagy can appear in several variants with respect to the form of the participating glial cells and the changes in the ganglion cells. We find that what has been described as neurophagy can have different relevance at closer inspection.

To the above described about the degenerative processes, I will add a schematic overview (page 530). It is evident that this is not at all a final description of the degenerative processes in the nervous tissue, but a preliminary sketch. It will get corrections and require further analysis. Future research will add issues so far not touched or mentioned. This account will provide advantages despite its incompleteness to get oriented and find gaps which need to be filled to better understand and foster confirmation.

**10.The results of this investigation and the limits of this efforts.**

In the presentation above, many changes have been described which can be found in acute disease events of the central nervous system and an explanation has been provided. These observations have been collected from many materials and by many investigations using different methods and it can be considered solid.

**530**

**Sketch of the degenerative processes**

| Form of degradation | Occurrence | Preproducts | Staining specialties of the particular pathologic substance in the nervous elements |
| --- | --- | --- | --- |
| **I. Degradation events at the ganglion cells**  1. Granular degeneration of the cytoplasm |  |  |  |
| a) fatty degeneration of the ganglion cells | Wide spread  Type: senile dementia | Fuchsinophil granules | + yellowisch pigmentation.  Brown to black after chrome osmium. Scharlach. Carbol fuchsin methylene blue. Weigert´s myelin stain. Weigert´s fibrin stain.  - May-Grünwald, Ehrlich´s hematoxylin stain |
| a) coarse-granular- fatty degeneration | Senile dementia, arteriosclerosis, focal diseases, encephalitis | Dark blue with Mann´s staining, brown-black with Bielschowsky.  + lithium carmin,  Ehrlich´s hematoxylin.  - Scharlach, osmium after chrome | Like 1a. Fatty granules very big, in the chrome osmium preparations show the form of lipoid cysts |
| b) granules of the amaurotic idiocy | amaurotic idiocies | Fuchsophine granules? | + May-Grünwald, Weigert´s myelin stain, Ehrlich´s hematoxylin  - osmium after chrome, Scharlach. At the Spielmeyer form slight pigmentation, at the Tay-Sachs form it lacks entirely. |
|  |  |  |  |
|  |  |  |  |

**531**

| Staining features of the | particular pathologic | substance | Remarks |
| --- | --- | --- | --- |
| In the glial cells | In the perivascular space | In the mesodermal cells of the vessel wall, blood elements, pia |  |
| Similar. Besides in acute disease states also fuchsinophil granules. At rapid progressing disease also like 1d | - | Like in glial cells, at acute disease states in addition fuchsinophil bodies | - |
| Like 1a | - | Like 1a | - |
| In fewer events like in the ganglion cells, in most events + Scharlach, osmium after chrome, carbol fuchsin methylene blue | - | + Scharlach, osmium after chrome, carbol fuchsin methylene blue | - |
|  |  |  |  |

**532**

| Form of degradation | Occurrence | Preproducts | Staining specialties of the particular pathologic substance in the nervous elements |
| --- | --- | --- | --- |
| c) fuchsinophil granules | Abundant at acute processes, and partially identical with 1d and at chronic degenerative processes (maybe these are two different issues) | - | + acidic fuchsin light green, hematoxylin staining with method VII  - Scharlach, osmium ather chrome, May-Grünwald |
| d) granules of the severe Nissl disease | Abundant in acute processes | - | Obviously a rapid change with respect to the different stainings  + basic Anilin stains, acidic fuchsin light green, Fibrin staining (but only transient)  - May-Grünwald, Scharlach, osmium after chrome |
| Dissolving processes of the cytoplasm |  |  |  |
| a) simple basophile substances | Abundant at acute and chronic processes | - | - |
| b) Multiple dissolvement processes at which special, so far detectable products are not formed | Abundant at acute and chronic processes | - | - |
|  |  |  |  |

**533**

| Staining features of the | particular pathologic | substance | Remarks |
| --- | --- | --- | --- |
| In the glial cells | In the perivascular space | In the mesodermal cells of the vessel wall, blood elements, pia |  |
| At chronic degenerative processes often without granules  At acut like 1d | At acute like 1d | At acute like 1d | - |
| Ameboid glial cells with fuchsinophil granules and fatty cysts. Methylene blue granules, fibrinoid granules, filled bodies | With acidic fuchsin light green, with Mallory hematoxiline, Mann´s solution (methyl blue), fibrin methods stainable products, degenerated ameboid glial cells | With Scharlach, osmium after chrome stainable substances | Shows a peculiar dissolvement process of the cytoplasm |
| Like 1d and also other glial cell forms with simple basophil inclusions | Like 1d and simple basophil products | Like 1d | - |
| Like 1 a and 1 d | Like 1 a and 1 d | Like 1 a and 1 d | One compares as example what has been stated on the degeneration of the protoplasmic processes at the fatty degeneration |
|  |  |  |  |

**534**

| Form of degradation | Occurrence | Preproducts | Staining specialties of the particular pathologic substance in the nervous elements |
| --- | --- | --- | --- |
| I**I. Degenerations at the myelinated fibers and axons** |  |  |  |
| a) slab decay of the myelin sheath, Elzholz bodies, myelin slabs and balls | Abundant. Type: strand degeneration of the spinal cord | - | + colored brown to black with osmium after chrome  - Scharlach, carbol fuchsin methylene blue, May-Grünwald, basic Anilin dyes |
| b) basophil metachromatic products  (p-granules of Reich) | Abundant, occurring obviously without decay of the myelin sheath | - | First found in glial cells |
| b) particular basophil metachromatic products | At special disease states | - | First found in glial cells |
| c) granular decay of the axons | Abundant, particular at acute processes | - | Stains red with Mann´s stain, with acidic fuchsin shining red; can be illustrated with the Doaggio´s hematoxylin staining |
|  |  |  |  |

**535**

| Staining features of the | particular pathologic | substance | Remarks |
| --- | --- | --- | --- |
| In the glial cells | In the perivascular space | In the mesodermal cells of the vessel wall, blood elements, pia |  |
|  |  |  |  |
| + Scharlach, osmium after chrome  - yellow pigmentation, carbol fuchsin methyl blue | - | Like in glial cells | - |
| Mostly with toluidine blue, thionin, with cresyl violet metachromatic reddish stained, partially rapidly transforming into fatty substances stained green with the same dye and with Scharlach | - | Largely with the mentioned stains labelling greenish, stainable with Scharlach and osmium after chrome | - |
| With toluidine blue and thionin reddish metachromatic stainable, abundant in large numbers, in older cells rust-colored metachromasia, green labelled with Ehrlich´s triazid | - | Only little reddish metachromatic basophil, largely greenish, stainable with Scharlach and osmium after chrome | - |
| Ameboid glial cells like 1d | Like 1 d | Like 1d | - |
|  |  |  |  |

It cannot be concluded with the same certainty that the conclusions which can be drawn are in all details correct. Despite there was quite an abundant material of specimen from which these conclusions were drawn, there are still significant gaps which have to be filled with future studies and one has to bridge with an explanation. There are still observations which do not fit into the conclusions. It may be possible that some may be corrected after more detailed analysis. Even if some details have to be considered, this study has certainly advanced our understanding of pathologic processes in the nervous system.
It is obvious that there are substantial tissue alterations in a large number of diseases for which we could obtain only uncertain and difficult to determine pathological results. This can be best illustrated in an example. If we are studying figure 20 of Table XXIX, then the severe tissue alteration compared to the normal spinal cord is quite obvious.

Table XXIX, Fig. 20 Slice from the white matter of the spinal cord from a case of progressive chorea.

Before I have illustrated such images, I had slices of the same spinal cord stained with the Weigert myelin sheath and glia method and according to Nissl. Inspecting the myelin sheath preparation closer, one observes that the myelinated fibers in the white matter are further apart from each other as compared to the normal spinal cord. The reason for that could not be understood. In the glial fiber preparation, the low number of fibers in the border zone and in the entire white and grey matter was obvious. One might consider that it was an appropriate staining, yet one could not draw much conclusion from this result. In the Nissl stain the ganglion cells were fatty, partially small and peach-like. The glial nuclei were remarkably small, dark and often surrounded with small protoplasmic cell bodies and a pile of greenish pigments. Yet what is the pathologic relevance? Based on this preparation, the changes in the spinal cord could only be categorized as those pathologic findings which are often found in the diseased nervous system, but are so undefined and vague that they did not advance our understanding and are difficult to distinguish from other conditions. Now we observe sharply delineated deviations spread over the entire spinal cord. We can recognize why the myelinated fibers of the white matter are separated from each other: since large ameboid glial cells have moved in between. We also recognize why the glial fibers are so sparse: since ameboid glial cells and filled bodies have formed and with their formation the glial fibers decay in more or less large numbers. We find that the ganglion cells are not only fatty degenerated, but they have also lost their protoplasmic processes and are damaged at their terminal ends. The delicate nervous structures are replaced by tissue useless for the nervous function: ameboid cells and filled bodies. The entire spinal cord is with respect to its nervous mass strongly atrophic, while the useless substitution and filling material is hypertrophic. With this recognition the entire disease process is much better understood. Severe symptoms and disease conditions where only sparse results were available are now better understood based on their anatomical structure: the epileptic state, severe paralytic seizures, severe deliria due to infections and intoxications, catatonic anxiety states. Whoever still had doubts whether dementia praecox (11) or epilepsy are indeed diseases which are accompanied by severe organic alterations in the cortex, must now be convinced. At other acute diseases where we already knew pathologic alterations, it is even more evident. This is even more obvious when analyzing series of experimental subacute maximal intoxications with novel methods and comparing it to previous studies with the Nissl method.
With the opportunity of a more precise analysis of the pathologic alterations it opens the possibility

**536**

It cannot be concluded with the same certainty that the conclusions which can be drawn are in all details correct. Despite there was quite an abundant material of specimen from which these conclusions were drawn, there are still significant gaps which have to be filled with future studies and one has to bridge with an explanation. There are still observations which do not fit into the conclusions. It may be possible that some may be corrected after more detailed analysis. Even if some details have to be considered, this study has certainly advanced our understanding of pathologic processes in the nervous system.
It is obvious that there are substantial tissue alterations in a large number of diseases for which we could obtain only uncertain and difficult to determine pathological results. This can be best illustrated in an example. If we are studying figure 20 of Table XXIX, then the severe tissue alteration compared to the normal spinal cord is quite obvious.

Table XXIX, Fig. 20 Slice from the white matter of the spinal cord from a case of progressive chorea.

Before I have illustrated such images, I had slices of the same spinal cord stained with the Weigert myelin sheath and glia method and according to Nissl. Inspecting the myelin sheath preparation closer, one observes that the myelinated fibers in the white matter are further apart from each other as compared to the normal spinal cord. The reason for that could not be understood. In the glial fiber preparation, the low number of fibers in the border zone and in the entire white and grey matter was obvious. One might consider that it was an appropriate staining, yet one could not draw much conclusion from this result. In the Nissl stain the ganglion cells were fatty, partially small and peach-like. The glial nuclei were remarkably small, dark and often surrounded with small protoplasmic cell bodies and a pile of greenish pigments. Yet what is the pathologic relevance? Based on this preparation, the changes in the

**537**

spinal cord could only be categorized as those pathologic findings which are often found in the diseased nervous system, but are so undefined and vague that they did not advance our understanding and are difficult to distinguish from other conditions. Now we observe sharply delineated deviations spread over the entire spinal cord. We can recognize why the myelinated fibers of the white matter are separated from each other: since large ameboid glial cells have moved in between. We also recognize why the glial fibers are so sparse: since ameboid glial cells and filled bodies have formed and with their formation the glial fibers decay in more or less large numbers. We find that the ganglion cells are not only fatty degenerated, but they have also lost their protoplasmic processes and are damaged at their terminal ends. The delicate nervous structures are replaced by tissue useless for the nervous function: ameboid cells and filled bodies. The entire spinal cord is with respect to its nervous mass strongly atrophic, while the useless substitution and filling material is hypertrophic. With this recognition the entire disease process is much better understood. Severe symptoms and disease conditions where only sparse results were available are now better understood based on their anatomical structure: the epileptic state, severe paralytic seizures, severe deliria due to infections and intoxications, catatonic anxiety states. Whoever still had doubts whether dementia praecox (11) or epilepsy are indeed diseases which are accompanied by severe organic alterations in the cortex, must now be convinced. At other acute diseases where we already knew pathologic alterations, it is even more evident. This is even more obvious when analyzing series of experimental subacute maximal intoxications with novel methods and comparing it to previous studies with the Nissl method.
With the opportunity of a more precise analysis of the pathologic alterations it opens the possibility

**538**

to analyze its distribution within the central nervous system. Some of the results are surprising.
Yet, we also face our limitations. The ameboid cells are short-lived formations and they and most pathologic substances which are formed in the nervous tissue disappear within a relatively short time. If, for instance, a storm of catatonic changes has wiped through the cortex, then there is not much seen of the peculiar glial cells and the pathologic granules, apart from the accumulation of lipoid substances in ganglion and glial cells and in the cells of the vessel walls which are removed much more slowly. The traces which are left in nerve and glial cells are very difficult to determine and to detect even with the new methods of investigation. From the same disease, we obtain different images if we study acute and chronic states. In contrast, we obtain similar results from different diseases if they are at similar states of progression. In slices of a delirium from severe intoxication of an acute character, we observe similar changes as in a catatonia case that had died after a strong excitation. This indicates that a differential diagnosis is difficult. Drawing the conclusion that the disease process of these mentioned cases is identical is certainly not correct. We just illustrate the degeneration processes. Also, in the spinal cord we obtain quite different results whether it is a rapidly progressive, a chronic, or a stationary tabes, if we only label the granule cells. For the granule cells in the dorsal cord we cannot decide whether it is a tabes (34) or an ascending disease of the dorsal cord after spinal cord lesion. The difference between these two disease processes resides in their different progression, in the involvement of different systems of the spinal cord, and in the regions that are destroyed and those that remain untouched. The progression of acute changes in the cortex we can now better study than before, since they are better illustrated.

**539**

The final deficiencies are much more difficult to diagnose in the cortex compared to the spinal cord and sometimes not detectable at all. It is also evident that we observe obvious changes in the acute phase of some disease states while the changes in the chronic state seem to be much less relevant and we do not understand it. Thus, the differential diagnostic analysis does only provide a very limited insight.
Alone the appearance of multiple ameboid cells in some cases will give us important indications for the diagnosis. We find, for instance, that in some cases of patients who died after severe excitation the formation of ameboid cells in any form has not happened, while in other cases they are quite abundant. One has to assume that these are distinct disease processes. It seems likely to me that cases in which many ameboid cells are found without a severe peripheral disease which can stimulate the formation of ameboid cells in the nervous system do not belong to mania. A lack of any ameboid cells in a psychosis with severe excitation speaks against dementia praecox. In the municipal asylums, one observes quite often acute severe states of excitement without obtaining a clear differential diagnosis until death. I will not state that the presence of multiple ameboid glial cells indicates dementia praecox (11) and a lack of them a manic-depressive lunacy. Some cases in which we try to find a differential diagnosis between these two disease states may not be resolved even with future psychiatric insights.
It seems to indicate that the formation of the ameboid glial cells depends rather on the severity of the disease state and less on the type of disease, and that future research and better observations will result in differential diagnostic indications. I have never found those round ameboid cells in the cortex at a status epilepticus, as I have observed it in the case of

**540**

status paralyticus or at deliria after severe infections. In these cases, we do not need a histological investigation for confirming the diagnosis, yet this observation may help for a differential diagnosis in other cases.
Some disease states seem to be characterized by degradation products of a special kind. Its number is so far very small and we are just at the beginning of our effort.
Of interest is another result. Based on the response of glia, we observe that many degenerative processes undergo alterations with respect to the progression of the disease. Some progress slowly, some very rapidly, others are stationary. While studying a large amount of anatomical material I have gained other insights as based on the clinical observations alone.
Of large importance is the progress in our general pathologic insight. It becomes now quite evident that the neuroglia has other functions than simply serving as a support structure of the central nervous system. A variety of glial elements are involved in such a task. They clean up, remove degradation material and help to deliver it to the lymphatic system. In certain conditions, the ameboid glia, by serving such functions, dominates over the fiber-forming support system which more or less decays. We recognize a magnitude of different glial cell forms and learn to understand their relevance. This allows us to get a better understanding of the pathologic events of the cortex and the features of disease processes.
We also have made single observations that the glia does not only participate in decay of degenerating nervous tissue, but also in disturbances of metabolic processes. This needs further studies. This will yield further visions to obtain a better understanding of pathological processes based on (glial) alterations.
With the finding of many different substances in the pathologic nervous tissue, one needs to perform an

**541**

attempt to collaborate with the chemistry of the pathologic brain as already tried by Wlassek and Reich which has a perspective of success. Since that such pathologic substances are found in large amounts in some disease states, it should be possible to extract and isolate them and determine their composition. This would considerably advance our understanding of these diseases.

**11. Reichardts determination of brain weight and critical remarks on the pathologic histology of the central nervous system.**

In several studies over the last years which are focused on the weight of the brain, Reichardt has discussed the limitations of the current histopathological analysis methods of the brain and also in that context my studies. It is important for me to discuss his views since his studies on the weight of the brain and the brain material has relevance for the acute changes of the nervous tissue as described above. It is an opportunity to further discuss the usefulness of the pathologic histology for some special areas.

As proof for the depressed mood which has now spread in the last decade among pathologists of the central nervous system, Reichard quotes an introductory sentence of my lecture ´On the degradation of the nervous tissue´ (Über den Abbau des Nervengewebes) in which studies are mentioned which should partially overcome these limitations those and should enable further progress. I assume that on that projected path the pathologic anatomy should yield more progress as the physical brain studies as outlined by Reichardt in his publications on brain matter. It should be in some respect more detailed and more exact as this could be ever achieved with the scale and without chemistry.

**542**

In the above-mentioned lecture, I have briefly mentioned that we find a large number of proliferating glial cells in certain disease states of the central nervous system and special pathologic products in the tissue. With little loss and the swelling of many nervous structures, it results in a swelling of the brain, as has already been stated by French scientists for the acute state of paralysis. What I just mentioned in passing and what needed further confirmation at that time, I have now further expanded in the present study.
The results of Reichardt’s precise brain weighing are supported by the anatomical studies and provide an addition and a confirmation of the anatomical investigation by a completely different research method. Reichardt obtains a number which reflects a significant change in the brain in very fresh cases. For an explanation of these changes, this physical brain investigation can only provide hypotheses (of which Reichardt warned with respect to the pathologic histology). With the microscope, we observe that the increase in weight is due to many components, of substances of quite diverse and distinct morphologic and staining features and thus also of distinct chemical properties. We can also recognize relations of these pathologic formations to normal structures. In some cases, we observe that besides the production of larger pathologic elements and a deposition of substances which should increase the brain weight, nervous structures more or less decay. Thus, the brain weight is composed of a reduction of normal and an increase of pathologic tissue components. This provides a deeper insight into the disease-related changes of the nervous tissue. I admit that it is of certain interest to obtain a number related to the changes of the brain.

**543**

Yet the Reichardt method does not yield a better performance as compared to pathologic histology.
What is outlined here in more general terms will be detailed with some examples.
For the considerations which resulted in these studies, one of those rare and surprising cases plays a role, namely that of a stuporous (35) catatonic who suddenly fell dead to the floor without any signs of diseased organs. While the inspection of the corpse did not result in any indications, it was obvious to indicate changes in the brain being responsible. At first inspection, nothing obvious could be observed. There was no hyperemia, no edema; in contrast, the brain substance was abnormally dry, without hydrocephalus and without exudation. Also, the inspection with the methods available at that time did not yield any results which could explain the sudden death. There was no inflammation, no gliosis, and no change which is known to pathologic anatomists if organs are enlarged. Some apparent small alterations from the normal remained incomprehensible in their relevance.
The lack of corresponding results could be explained in another way, namely that the cellular plasm of the brain is converted into something else, like Reichardt would name it, into a plant-like form, undetectable by the microscope. But it is more likely to assume that our current methods of investigation are not sufficient to recognize the existing pathologic changes. If one recognizes how many structures of this incredibly complex composition of the brain we are able to illustrate only with great effort, uncertainty, and sometimes only under certain conditions, then one must admit that there could be death of the brain without knowing the anatomical basis. We have to make efforts to improve our methods of investigation. Therefore, I again studied that interesting brain and applied methods

**544**

which were before tested on cross brain alterations. Now it shows that fundamental disturbances can be found in the cortex of a morphological and physiological type. Several figures of the present work are from this case. The pathologic histology solved part of the riddle.
Reichardt objects to the interpretations of these findings as degenerative processes. The results of his brain weight speak against such an explanation. He will be satisfied if they are viewed as another mix of the protoplasm. Indeed, in this brain there were no degenerating axons found. Also, a decay of cells could not be observed. Despite that, I have the opinion that the observed alterations can be considered as a general degenerative process. The decay of the nervous tissue, in particular the acute, does not proceed in the way that part after part of the nervous tissue disappears without trace as the present study shows. Without doubt, dissolution events play a significant role, in particular in acute processes. This is preceded by a more or less swelling of the protoplasmic structures and with changes in substances of the cytoplasm. The glia shows a rapid reactive alteration, resulting in a strong swelling of the glial elements and eventually a decay. By the dissolution of nervous tissue components and degenerating glial cells, the extracellular fluid is changed. In the microscopic image, this can be displayed if during the fixation process pathologic components of the tissue fluid precipitate and are transferred into insoluble products and illustrated by staining. Such products we have known in larger number. The degeneration of the nervous tissue is accompanied by multiple changes in the mix of the different protoplasmic formations and the tissue fluid. The majority of the previous description deals with changes in types and mixture of the protoplasm, or better, the tissue structures as expressed by Reichardt.

**545**

I believe that there are tissue alterations which are based on qualitative changes of the cytoplasm without degeneration, as in the view of Reichardt. I observe those, in particular in tissue images which are found in slight psychosis after infectious diseases (not in severe infectious deliria). One finds a swelling of nervous elements in the form of an acute cellular change according to Nissl or a bleak swelling described by other authors, but no decay of cellular structures. One just has to photograph cells at defined positions in the cortex in cases of psychosis after infections and compare them with areas at comparable locations in the normal cortex, and one is easily convinced to what degree the acute cellular changes increase the volume of the ganglion cells. The swelling of the ganglion cells is accompanied by changes in substances. The most obvious is the loss of Nissl slabs. We do not find ameboid glial cells and none of the substances as described above and which are correlated with a decay of the nervous system. In contrast, in the mentioned case of dementia praecox, ameboid glial cells were abundantly present. In particular, cells with fibrinoid granules were present in large numbers. I believe that this formation is correlated with the decay of the nervous system and with degenerative processes as described above. In this case, we observe only the beginning since death has stopped that development.
In particular, this shows the superiority of the histologic investigation over brain weighting, since with this method we can distinguish different processes which could never be separated by a scale.
What is relevant for dementia praecox (11) is also relevant for seizure-like disturbances of paralysis and epilepsy. So far, it is only partially correct, as Reichardt claims, that one cannot determine with the microscope whether a patient who shows paralytic changes in the brain was truly mentally ill

**546**

and whether he died of mental illness or in a complete psychic remission, whether he died of a seizure respective due to his mental illness or accidentally by another disease. Based on the changes in the cortex at the progressive paralysis, we can recognize something on the clinical condition at the time of death. A paralytic in which we detect masses of ameboid cells, degenerated axons, changes in ganglion cells in the form of the severe cell changes of Nissl, has likely died due to his brain disease, maybe also in severe mental disturbance on pneumonia sepsis or another intercurrent physical disease. A patient in which large ganglion cells and many axons have decayed in the cortex has certainly no complete psychic remission as he had no patellar reflexes, and dorsal strands are completely degenerated. The statement of Reichardt is also not correct that the pathologic anatomy could not provide an insight on the mechanisms of paralytic seizures in the brain. Starlinger provided evidence in 1895 that certain apoplectic seizures at paralysis are accompanied by a widespread myelin degradation in certain cortical areas. The studies by Lissauer and me, which I still continue, provide histologic evidence that the apoplectiform seizures of the paralytics cause a very rapid disease progression in certain regions of the cortex. Also, in cumulative epileptiform paralytic seizures, it can be clearly recognized that the paralytic disease process has undergone a significant acute augmentation, which is reflected in tissue changes as described in this report, with special modifications (vessel infiltration, etc.) attributed to paralysis. I also claim to have regularly found defined changes in status epilepticus, the genuine epilepsy. This has been mentioned so often that I will spare a repetition for the reader.
It seems to me an otiose question whether one can get a vision of what happens during seizures in the brain either based on these histologic findings or from the results

**547**

of Reichard´s weighting. We are accustomed to move within anatomical visions and will be much more satisfied, if we can coincide these events with anatomical changes; we medics have learned to associate our medical views to pathologic anatomical perceptions. If I know that somebody suffers on an interstitial nephritis, then I have a better understanding as the result that his kidney weights 50g less, as before the disease. For a researcher who searches for explanations of clinical features based on physical methods, this may be different.

Now Reichardt claims at another spot that changes based on seizures appear momentarily and disappear completely at epilepsy, at catatonia and more rarely at paralysis. This view would indicate that during an epileptic seizure these anatomical changes occur within seconds, last for a short time and then regress within a few seconds. All what we know about pathological tissue changes, they do not appear and go like a flash, as the symptomatic picture of an epileptic seizure. It would thus be unlikely that epileptic seizures are accompanied by anatomical changes. Yet based on clinical observations, we know that these seizures do not appear and disappear as a flash. With respect to the paralysis one can often predict a seizure hours before. Wernicke has stated that each paralytic seizure results in an increase in dementia, even if there are cases where this increase is hardly noticeable, while in others there is an obvious progress in dementia.

For most of the epileptic seizures it is not the case that they appear and disappear momentarily; the seizures are often preceded over hours or even days by a discomfort or a more or less severe disgruntlement. On the other hand, one observes not rarely

**548**

after hours or even days after the seizures several residual symptoms, if one carefully examines; difficulties with language, slowness and difficulties with thinking in general, disturbances of sensibility and the Babinski reflex (36) can be detected. Also for epilepsy, at a lower grade, the statement applies that not the single seizure, but the series of seizures leads to an impairment of intelligence. I have often seen young epileptic patients from which the parents or teachers stated that they observed an obvious change in the psychic condition with the first seizures.

Within the 20 years of my psychiatric practice, I have not often observed severe catatonic seizures. Some cases of dementia praecox (11) with severe epileptiform seizures quite rapidly declined into a deep catatonic idiocy. More striking was a preliminary stage at epileptic seizures which occurred in acute forms of endarteritic syphilis (33) related dementia. A peculiar helpless timidity at a slight dampened consciousness made it frequently possible to correctly predict an epileptiform seizure even days ahead.

These coarse clinical observations show us, that seizures are getting prepared and that in no way a momentarily reconstitution and often not a reconstitution to the previous condition will happen. More precise studies, in particular with psychophysical instruments would that certainly show in more detail.

These indicate single histological findings, which require further additions since one rarely gets a clear case for an investigation. That a seizure is developed after a formation of histologic changes is indicated by the presence of multiple ameboid cells in the cortex of an epileptic case. He was drowned obviously during an epileptic seizure in the bath. We also have evidence of significant changes, even a decay of axons in the brain of an epileptic who died a few hours after a seizure. The evidence of decaying axons in the brain of deceased during status epilepticus indicates that severe seizures result in damage of intelligence.

**549**

The observation that after a seizure the tissue alterations are not normalized is indicated by the finding that in the cortex of epileptic patients who died of an intercurrent disease and who had many seizures before, but not in the last days, many tissue alterations can be found which are related to the seizures.
Based on different considerations, it seems unlikely to me that the epileptic seizure is caused by a defined morphologic and qualitative alteration of the brain. If one considers how many quite different disease conditions of the brain coincide with seizures which do not show common features (tumors, epileptic forms different from epilepsy, eclampsia, paralysis, syphilis-related dementia, arteriosclerosis, uremia, alcoholism, cocaine abuse, lead intoxication, and different intoxications in animals), it is evident that these epileptic and epileptiform seizures are initiated by previous changes in the brain and that the trigger can be due to many damages. We find in these different brains quite distinct changes and we observe seizures in patients with hysteria which cannot be differentiated from epileptic seizures by the best diagnostic medics. This still unknown event does not become clearer to us based on the assumption that it is the result of a flash-like developing and after a few minutes disappearing alteration in the brain matter.
At another point, I agree with Reichardt. I also have the opinion that one should not agree with the cause of death being 'heart and breathing paralysis' in mentally diseased patients, but one has to search for alterations in the cortex. Yet I am convinced that one has to search for it with the microscope.
In Reichardt's article on the brain matter, we find many indications on the shortcomings and sources of error of anatomical studies. I admit that this is correct, but in part

**550**

illustrated too colorfully. Most of the issues to which Reichardt points are quite obvious to every anatomist. We all know that we do not have unchanged tissue in our preparations. We work with comparable images and constantly need to compare normal and pathologic tissue components using the same methods. Nissl, the true father of the pathologic anatomy of the cortex, has emphasized this on many occasions. That we, despite this, can obtain deeper insight along that path shows the development of pathologic anatomy of the peripheral body, which faces the same difficulties.
I have never had the feeling of resignation. But the knowledge of the extreme difficulties of this research field has restrained me from hoping that the microscope will resolve all riddles of psychiatry in the near future. When I summarize what pathologic anatomy has yielded in the last decade of the last century, I can say: a good piece of work. I will not list the names and the studies here, since I am afraid to forget important ones. The physical investigation of the brain may lead to some progress in perception and we have all reasons to be happy about this new research method. Rieger's and Reichardt's merits should not be put down; yet they should persist without putting down the value of pathologic anatomy. The brain physicists may weigh; we will advance psychiatry with the microscope and will not let ourselves get disturbed in our efforts. I am sure, we should not be afraid of the scale.

**Literaturverzeichmis**

ACHÚCARRO, Zur Kenntnis der pathologischen Histologie des zentralenNervensystems bei Tollwut. Nissls histol. u. histopathol. Arbeiten,Bd. III.

ALTMANN, Die Elementarorganismen und ihre Beziehungen zu den Zellen. Leipzig 1874.

ALZHEIMER, Histologische Studien zur Differentialdiagnose der progressiven Paralyse. Nissls histol. u. histopathol. Arbeiten, Bd. I.

Ders., Über den Abbau des Nervengewebes. Zeitschr. f. Psych. 1906.

BENDA, Neurogliafärbung. Enzykl. d. mikrosk. Technik. Berlin u. Wien 1903.

BETHE, Allgemeine Anatomie und Physiologie des Nervensystems. Leipzig 1903.

BEST, Die Karminfärbung des Glykogens und des Kerns. Zeitschr. f. Mikroskopie, Bd. XXIII.

BONFIGLIO, Circa le alterazioni della cortecia cerebrale conseguenti ad intossicazione sperimentale de carbonato di piombo. Nissls histol. u. histopathol. Arbeiten, Bd. III.

CASAMAJOR, Zur Histochemie der Ganglienzellen der menschlichen Hirnrinde. Obersteiners Arbeiten, Bd. XVIII.

CERLETTI,Contributo sperimentale alla conoscenza dei processi di fagocitosi nella sostanza cerebrale. Ann.dell Istituto psich. Roma 1902.

Ders., Sulla neuronofagia e sopra alcuni rapporti normali e patologici frea elementi nervosi ed elementi non nervosi. Ann. dell Istituto psich.Roma 1903.

Ders., Sopra speciali corpuscoli perivasali nella sostanza cerebrale. Rivista sperimentale, Vol. XXXIII.

DONAGGIO, Colorazione positiva della fibre nervose etc. Rivista sperimentale, Vol. XXX.

ELZHOLZ, Zur Kenntnis der Veränderungen des zentralen Stumpfes lädierter Nerven. Jahrb. f. Psychol., Bd. XVII.

Ders., Über einen eigentümlichen histologischen Befund im zentralen Stumpf von durch Gangrän zerstörter peripherer Nerven. Monatsschrift f. Psychol. u. Neurol. 1899.

ELZHOLZ, Zur Histologie alter Nervenstümpfe in amputierten Gliedern. Zeitschr. f. Psychol. 1900.

EISATH, Über die normale und pathologische Histologie der menschlichen Neuroglia. Monatsschr. f. Psychol. u. Neurol., Bd. XX.

FARRAR, On the Phenomena of Repair in the cerebral Cortex. Nissis histol. u. histopathol. Arbeiten, Bd. II.

FORSTER, Experimentelle Beiträge zur Lehre der Phagozytose der Hirnrindenelemente. Nissls histol. u. histopathol. Arbeiten, Bd. II.

GALBOTTI, Über die Granulationen in den Zellen. Intern. Monatsschr. f. Anat. u. Physiol. 1895.

HEIDENHAIN, Plasma und Zelle. Jena 1907.

HELD, Über den Bau der Neuroglia und über den Stand der Lymphgefäße in Haut und Schleimhaut. Abhandl. d. mathem.-phys. Klasse d. sachs. Ges. d. Wiss. Leipzig 1903.

Ders., Zur weiteren Kenntnis der marginalen Neuroglia. Verhandl. d. Ges. Deutscher Naturf. u. Ärtze. Leipzig 1908.

Ders., Die Entwicklung des Nervengewebes bei den Wirbeltieren. Leipzig 1909.

LEVI, Contributo alla fisiologia della cellula nervosa. Rivista di pathologia nervosa e mentala 1896, Vol. I.

MARINESCO, Études sur l'évolution et involution de la cellule nerveuse. Revue neurol. 1899.

Ders., La cellule nerveuse. Paris 1909.

MERZBACHER, Untersuchungen üiber die Morphologie und Biologie der Abräumzellen im zentralen Nervensystem. Nissls histol. u. histo-pathol. Arbeiten, Bd. III.

DE MONTET, Über Wanderungen lipoider Substanzen im Zentralnerven-system. Inaug.-Diss. Tübingen 1906.

Nissl., Kritische Besprechung von H. Schmaus: Vorlesungen über die pathologische Anatomie des Rückenmarks. Zentralbl. f. Nervenheilk. 1903.

Ders., Uber einige Beziehungen zwischen Nervenzellenerkrankungen und gliösen Erscheinungen bei verschiedenen Psychosen. Arch. f. Psychiat., Bd. XXXII.

Ders., Zur Histopathologie der paralytischen Rindenerkrankung. Nissls histol. u. histopathol. Arbeiten, Bd. I.

OBERSTEINER, Über das hellgelbe Pigment in den Nervenzellen und das Vorkommen weiterer fettähnlicher Körper im Zentralnervensystem. Obersteiners Arbeiten, Bd. X.

Ders., Weitere Bemerkungen über die Pigmentkörnchen im Zentralnervensystem. Obersteiners Arbeiten, Bd. XI.

OLMER,Recherches sur les granulations de la cellule nerveuse. Lyon 1901.

OPPENHEIM, Zur pathologischen Anatomie der multiplen Sklerose mit besonderer Berücksichtigung der Hirnrindenherde. Neurol. Zentralblatt 1908.

PERUSINI, Über klinisch und histologisch eigenartige Erkrankungen des späteren Lebensalters. Nissls histol. u. histopathol. Arbeiten, Bd. III.

Pilcz, Beitrag zur Lehre von der Pigmententwicklung in den Nervenzellen. Obersteiners Arbeiten 1895.

REICH, Über eine neue Granulation der Nervenzellen. Arch. f. Anat.u. Physiol. 1903.

Ders., Zur feineren Anatomie der Nervenzellen. Arch. f. Psychiat. 1909.

Ders., Über die feinere Struktur der Zelle des peripheren Nerven. Zeitschr. f. Psychiat. 1905.

Ders., Über den zelligen Aufbau der Nervenfaser auf Grund mikrohistiochemischer Untersuchungen. Journ. f. Psychol. u. Neurol., Bd. VIII.

Ders., Dikussion zu dem Vortrag von Lipschutz. Zeitschr. f. Psychiat. 1907.

Ders., Über Unterschiede im Bau der zentralen und peripheren Nervenfaser auf Grund mikrohistiochemischer Untersuchungen. Zeitschr, f. Psychiat., Bd. LXVI.

REICHARDT, Über die Untersuchungen des gesunden und kranken Gehirns mittels der Wage. Jena 1906.

Ders., Über die Hirnmaterie. Monatsschr. f. Psychiat. u. Neurol.,Bd. XXIV.

Ders., Untersuchungen über das Gehirn. I. Teil. Münchener med.Wochenschr. 1909.

SAND, Le neuronophagie. Bruxelles 1906.

SCHAFFER, Weitere Beiträge zur pathologischen Histologie der familiären amaurotischen Idiotie. Journ. f. Psychol. u. Neurol. 1906.

Ders., Zur Pathogenese der Tay-Saschsschen amaurotischen Idiotie. Neurol. Zentralbl. 1905.

Ders., Beiträge zur Nosographie und Histopathologie der amaurotisch-paralytischen Idiotieformen. Arch. f. Psychiat. 1906.

Ders., Über die Anatomie und Klinik der Tay-Sachsschen amaurotisch-familiaren Idiotie mit Rücksicht auf verwandte Formen. Zeitschr. zur Erforschung u. Behandlung d. jugendl. Schwachsinns 1909.

SCHRöDER, Einführung in die Histologie und Histopathologie des Nervensystems. Jena 1908.

SPIELMEYER, Über eine besondere Form von familiärer amaurotischer Idiotie. Neurol. Zentralbl. 1906.

Ders., Klinische und anatomische Untersuchungen über eine besondere Form von familiärer amaurotischer Idiotie. Habilitationsschrift Gotha 1907.

Ders., Von der protoplasmatischen und faserigen Stützsubstanz des Zentralnervensystems. Arch. f. Psychiat., Bd. XLII.

STRANSKY, Über diskontinuierliche Zerfallsprozesse an der peripheren Nervenfaser. Journ. f. Psychol. u. Neurol. 1903, Bd. I.

STRÄUSSLER, Über eigenartige Veränderungen der Ganglienzellen und ihrer Fortsätze im Zentralnervensystem eines Falles von kongenitalen Kleinhirnatrophie. Neurol. Zentralbl. 1906.

Vogt, Über familiäre amaurotische Iddiotie und verwandte Krankheitsbilder. Monatsschr. f. Psychiat. u. Neurol., Bd XVIII.

WEIGERT, Zur pathologischen Histologie des Neurogliafasergerüstes. Zentralbl. f. allgem. Pathol. 1890.

Ders., Beiträge zur Kentnis der menschlichen Neuroglia. Frankfurt a. M. 1895.

WLASSAK, Die Herkunft des Myelins. Ein Beitrag zur Physiologie des nervösen Stützgewebes. Arch. f. Entwicklungsmechanik d. Organismen 1898, Bd. VI.

**Explanation of Tables**

Generally used abbreviations:

abs. degradation products

adv. adventitia

advl. adventitial lymphatic space

adz. adventital cell

aglz. ameboid glial cell

ax. axon

blz. red blood cell

cap. capillary

dax. degenerated axon

ekg. ectodermal tissue

ez. endothelial cell

fk. filled body

gaz. ganglion cell

glf. glial fiber

glfs. glial cell process

glz. glial cell

int. intima

l. vessel lumen

l. k. lipoid body

lz. lymphocyte

mkz. mesodermal granule cell

m. l. membrana limitans gliae

msch. myelin sheath

mph. macrophage

p. pia

p.v.r. perivascular space

r. glial border layer

**Figure Legends**

**Table XXVIII**

contains drawings from preparations which were obtained with method IV, Zeiss homogeneous immersion 1/12. Ocular 8.

Fig. 1 *a, b*. Normal glial cells with protoplasmic branches from the cortex of a 34-year-old man who died due to an accident. *c* glial cell from the cortex of a 68-year-old man diseased with arteriosclerosis. *d* glial cell from the deeper cortical layers of a 63 years old woman diseased with depression. *e, f, g* glial cells from the white matter of a 34-year-old man died due to an accident. *h, I* glial cells from the cortex of juvenile epileptic demented due to multiple seizures.

Fig. 2. Ameboid glial cells from the cortex of a case of severe delirium after infection. *a* the glial cells deform the cell body and nucleus of the ganglion cell. *b* an ameboid glial cell with two stump-like processes (maybe the rest of originally longer processes). *c* a ganglion cell surrounded by six ameboid cells.

Fig. 3. A capillary from the cortex surrounded by younger and older ameboid cells; in the older cell beginning degradation of the cytoplasm, at *a* cell with methylene blue granules.

Fig. 4. Small ameboid glial cell from the white matter of a delirium after infection.

Fig. 5. Small ameboid glial cell of a case of catatonia with sudden death.

Fig. 6. Larger ameboid glial cell with vacuoles (v), surrounding myelin sheath. Status epilepticus.

Fig. 7. *a* small glial cells potentially transition forms from round glial cells with cytoplasmic granules to ameboid cells. *b* and c small ameboid cells potentially transition forms of glial cells with cytoplasmic processes to ameboid cells. *d* large ameboid cell with two vacuoles (v) forming a wristband around a capillary and surrounding many myelinated fibers. ´fear psychosis´.

Fig. 8 *a, b, c*. large ameboid glial cells from the cortex of a very rapidly progressing paralysis. In *c* detachment of the nuclear membrane from the nuclear content. Dust-like fine methylene blue granules in the cytoplasm. Progressive paralysis.

Fig. 9. Large ameboid glial cell with methyl blue granules. ´fear psychosis´.

Fig. 10 Large ameboid glial cell with nuclear degradation and methylene blue granules. Status epilepticus.

Fig. 11. Glial cell with methyl blue granules in the arrangement of protoplasmic branching. Final delirium at phthisis (37).

Fig. 12 and 13. The same cells from the white mater of a suddenly deceased due to catatonia.

Fig. 14. Small vein of the white matter of a case of ´fear psychosis´. One can clearly observe the perivascular space and the adventitial lymphatic space. In the latter are, besides other cells, two typical grid cells (granule cells). In the perivascular space are besides a number of young glial cells a young and a large ameboid cell; the later contains large methylene blue granules and a regressive altered nucleus and is alike the piles lacking nuclei in the perivascular space. In the nervous tissue young glial cells of different types, also some with methylene blue granules.

**Table XXIX**

shows preparations which were obtained with method V. Zeiss homogeneous immersion 1/12. Fig. 1, 3-20 ocular 8. Fig. 2 ocular 4.

Fig. 1. Section of the corpus striatum close to the ventricular surface from a case of progressive chorea. The tissue is seeded with filled bodies, besides are some ameboid glial cells, some glial nuclei with little cytoplasm, single glial fibers and axons visible.

Fig. 2. Small vein from the white matter of a cases of ´fear psychosis´ with band-like development of the adventitia. One can clearly observe the perivascular space in which there are large ameboid cells besides multiple piles of degenerative products.

Fig. 3. Glial cells in the cortex of the frontal sulci from a case of progressive chorea. The cell body is dissolved in multiple, coarse methyl blue granules, the band-like processes show a uniform dark stained cytoplasm.

Fig. 4, 5, 15. Similar cells of the same case (progressive chorea), frontal, central sulcus, with rosary-like processes, partly disintegrated in single pieces. Degeneration at the nucleus.

Fig. 6. Ganglion cell with the frontal process deformed by an ameboid glial cell. The frontal process is goblet-like pressed apart. Progressive chorea. Frontal, central sulcus.

Fig. 7. In a tissue gap which shows the form of a ganglion cells are remnants of a ganglion cell, two young ameboid glial cells and three degenerated nuclei which obviously belong to decaying ameboid ganglion cells. The same case (progressive chorea).

Fig. 8. Ganglion cells surrounded by a large perivascular space in which degradation products of an ameboid glial cells are positioned. The same case (progressive chorea).

Fig. 9. Status of a severe cellular alteration according to Nissl. Delirium-acutum-like condition at paralysis. In the tissue gap, which filled the ganglion cell, lays a degenerated nucleus of the ganglion cell with dissolved membrane and a degenerated glial cell nucleus, in between piles of granules.

Fig. 10, 11, 12 and 13. Ameboid glial cells which lay in ganglion cells or their processes. Central sulcus. Progressive chorea.

Fig. 15, 16, 17 and 18. Different forms and decay stages of ameboid glial cells at a progressive chorea.

Fig. 19. Slice from the white matter of spinal cord from a half-grown goat.

Fig. 20. Slice from the white matter of the spinal cord from a case of progressive chorea.

**Table XXX**

shows preparations obtained with method VI. Zeiss homogeneous immersion 1/12. Ocular 12.

Fig. 1 *a-c*. Glial cells from the cortex of a case of epileptic deliria. *a* contains only fuchsinophil granules, *b* and *c* fuchsinophil granules and lipoid cysts.

Fig. 2 Ameboid glial cells from the ventral horn of the spinal cord of a paralysis with deliria with an acute-type course.

Fig. 3. Ameboid glial cells from the white matter of different cases of status epilepticus and peculiar psychosis of the degenerated age (24). *l, m* smallest forms of ameboid glial cells from the cortex of a case of delirium after infection. *g, h, I, k* younger forms; in *g* and *i* each with a light green granule, otherwise only fuchsinophil granules. *b, c, d, e, f* older forms with large and smaller lipoid cysts. *a* glial fiber forming cell without clear cytoplasm filled with lipoid cysts.

Fig. 4. Ganglion cells with ameboid glial cells from the cortex of a delirium acute case of progressive paralysis. Deformation of the cells. Substitution of the cell body by ameboid glial cells. Nuclear degeneration of the ameboid cells.

Fig. 5 Large cells belonging to the type of fiber forming glia with a pile of dust-like fuchsinophil granules in the center of the cytoplasm. Progressive paralysis.

Fig. 6. Similarly, a cell with complete replacement of the cytoplasm by delicate fuchsinophil granules.

Fig. 7. Capillary from the cortex of a case of epileptic delirium. In the perivascular space resides a glial nucleus, from which rows of granules emerge formed by lipoid and fuchsinophil granules. They continue to the ectodermal tissue on one side and to the vessel wall on the other.

Fig. 8. Large ameboid glial cells with fuchsinophil granules, which resides with part of their cell body in the nervous tissue and with the other it bridges a perivascular space and enwraps a capillary. Status epilepticus.

Fig. 9. Small vein of the cortex of a case of epileptic delirium. The perivascular space is pervaded by band-type bridges in which fuchsinophil granules reside. Some of them which continue into the ectodermal tissue maybe viewed as processes of glial cells, the others maybe considered as clotting products. In the perivascular space are no lipoid substances, in contrast to the adventitial lymphatic space and in the cells of the adventitia.

Fig. 10. Vein from the white matter of a case of a deadly proceeded psychosis of the degenerated age. The perivascular space is almost everywhere bridged by broad bands in which fuchsinophil granules reside. Most of them terminate at nervous tissue and show no connection to nuclei. In between are two ameboid glial cells. In the perivascular space are no lipoid substances, large accumulations of those in the large granule cells of the adventitial lymphatic space.

**Table XXXI**

shows fibrinoid granules labelled with the Weigert glia method. Zeiss homogeneous immersion1712. Ocular 12 (Fig. 14, Ocular 8). Method X.

Fig. 1-7. Cells from the cortex. Fig. 8, 9, 11, 12. Cells of the white matter. Fig. 1, 2, 3, 12 ´Fear psychosis´.

Fig. 4, 6, 8, 9. Sudden death at catatonia. Fig, 5, 7. Final delirium at phthisis (32). Fig 12. Acute dementia praecox.

Fig. 10, 13. Accumulation of fibrous coagulum in the perivascular spaces at a ´Fear psychosis´.

Fig. 14 Ganglion cell from the third cell layer of the cortex from a case of ´Fear psychosis´., embedded by a processes of a glial cell filled with fibrinoid granules.

**Table XXXII**

shows the basophil metachromatic degeneration substances. Zeiss homogeneous immersion 1/12. Ocular 8. 6 and 10 alcohol slices, the others formaldehyde frozen sections. Staining with toluidine blue.

Fig. 1. Glial cells from the cortex and white matter from different diseases with metachromatic inclusions.

Fig. 2, 3. Ganglion cells surrounded by satellite cells which contain metachromatic basophil inclusions from deeper cortical layers of a case of severe manic excitation (death by exhaustion?).

Fig. 4. Surrounding of a small vein of deeper cortical layer from a man who died after severe manic excitation. Basophil metachromatic substances in the glial cells, in the perivascular space, and greenish lipoid substances in the cells of the adventitia.

Fig. 5. Small vein from the white matter of an epileptic patient. One observes granule cells in the adventitial lymphatic space with in part are almost completely loaded with basophil metachromatic substances which contain partially greenish labelled metachromatic substances. The basophil metachromatic substances are also found in glial cells of the ectodermal tissue.

Fig. 6. Longitudinal section of a vein in the white matter from a case of delirium after infection. In the adventitial lymphatic space are lymphocytes and cells with a large, faint red labelled cytoplasmic cell body (presumably macrophages). In the granule cells originating from the macrophages are greenish, yellowish and metachromatic substances (the fatty substances are partially extracted by the alcohol).

Fig. 7. Two glial cells from the white matter of a case of senile dementia which contain basophil, metachromatic and greenish lipoid substances.

Fig. 8. Four Schwann cells from the root of a case of syphilis related dementia with menigomyelitis, which shows Reich´s p-granules.

Fig. 9. Basophil metachromatic inclusions in a slightly growing glial cell in the white matter of the spinal cord of a meningitis due to tuberculosis in the form of Reich´s p-granules. Features of degeneration at the nucleus of the cell *a*; in cell *d* onionskin arranged p-granules.

Fig. 10. Two glial cells from the medulla oblongata from a case of an arteriosclerotic loaded with basophil metachromatic substances. The cell on the right shows at is lower part a honeycomb-like structure of the cytoplasm.

Fig. 11. Three glial cells from the white matter of the midbrain of a case of chorea progressive, loaded with basophil metachromatic products.

**Table XXXIII**

alcohol slices Fig. 1, 5, 7, 11-13, 15 Toluidin blue staining, 6, 8-10 Giemsa staining, 14 methyl green pyronin staining. Zeiss homogeneous immersion 1/12. Ocular 6.

Fig. 1. Different forms of degenerative altered glial nuclei from the white matter of the spinal cord of a case of an acute meningomyelitis due to tuberculosis.

Fig. 2. Different forms of degenerative altered glial cells from the cortex and the white matter from different cases of delirium after sepsis. In the cytoplasm of the cells are in many cases basophil metachromatic granules and substances.

Fig. 3. Different forms of degenerative altered glial cells from the cortex and the white matter of different cases of delirium after sepsis. In the cytoplasm of the cells are many basophil metachromatic granules and substances.

Fig. 4. Glial grid cells (granule cells) from the white matter of the posterior and lateral strand of a case of progressive paralysis. The fat which resided in the sphere-shaped cavity is completely extracted (compare Table XXXIV, fig. 2 and 6).

Fig. 5. Glial grid cell close to a focus in the white matter of a case of syphilis (?) related encephalitis with completely empty cavities; control preparations show that they were filled with fat drops.

Fig. 6. Top process of a ganglion cell into which a roundish ameboid glial cell is pressed in. Acute catatonia. At *a* a glial cell with many degenerative granules (lipoid nature).

Fig. 7. Beetz pyramidal cell of a case of delirium after acute paralysis. Nuclear alterations of a severe cellular illness of Nissl. At the top process and at an adjacent protoplasmic process are basophil granules. At the base of the cell and at the axonal process are drop-like accumulations of simple basophil products. Also, at the cell body of the ameboid glial cells which are around the ganglion cell.

Fig. 8. Ganglion cell surrounded by five ameboid cells. The nucleus is deformed due to the encroachment of an ameboid cells. In this glial cell beginning degeneration of the nucleus. Of a strange severe disease in the climacterium.

Fig. 9. A ganglion cell with an ameboid cell intruded into the cell body. The nucleus of the ganglion cell is deformed and relocated. Caryohexis of the glial nucleus. At the nuclei of the other ameboid cells also regressive alterations. From the same case as 8.

Fig. 10. Deformation of a ganglion cell and its nucleus by approaching ameboid cells.

Fig. 11. Ganglion cell with satellite cells from a case of acute catatonia. The ameboid glial cell is within a large niche of the cell body of the ganglion cell which it does not quite fill; at the edges of the niche simple basophil products, The glial nucleus shows regressive alterations (mulberry-shaped).

Fig. 12. *a* simple basophil granules originating from the decay of a protoplasmic process of the ganglion cell arranged in coccus-like rows. *b* pile of basophil granules from the perivascular space. *c* piece of a cell body of a ganglion cell in which basophil granules dissolve. *d* rest of a ganglion cell disassembling into basophil granules. From a case of uremic psychosis.

Fig. 13. Section from the nucleus of the regio subthalmacica of a case of progressive chorea. Two ganglion cells in which basophil products dissolve, in between permanent forms of ameboid glial cells and basophil degeneration products which show a peculiar ship-like form.

Fig. 14. Capillary with perivascular space from the cortex of a case of fear psychosis. In the perivascular space reside ameboid glial cells and peculiar, red-labelled masses which represent pathologic coagulation products.

Fig. 15. Three coffin embedded, dead ganglion cells with severe nuclear alterations and almost unstained cytoplasm enwrapped by glial cells which are not of the type of ameboid cells (coffin formation). From the white matter of a case of endarteritic syphilis (33) related dementia with many foci.

**Table XXXIV.**

Herxheimer preparations. Zeiss homogeneous immersion 1/12. Ocular 6.

Fig. 1. Glial cells with lipoid inclusions from the cortex of a case of endarteritic syphilis (33) related dementia.

Fig. 2. Glial cell, corresponding to the image of Table XXXIII, fig. 5a stained with alcohole toluidine blue. Spinal cord. Progressive paralysis.

Fig. 3. Glial cells with massive lipoid inclusions at the white matter of the temporal lobe from a case of atrophic sclerosis.

Fig. 4. From a slice through the cortex of a case of ´fear psychosis´. In the ganglion cells fairly few lipoid granules (some of the fatty granules lays at the ganglion cells and should belong to the glial cells). More lipoid substances are within the glial cells and particularly many in th cells of the adventitia.

Fig. 5. From a slice of the cortex from a case of Warren-Tay-Sachs amaurotic idiocy. In the ganglion cells little dust-like distributed fat, in the glial cells, large fatty piles, in particular large accumulation of fat in the adventitia.

Fig. 6. Different developmental stages of glial granule cells in the spinal cord. Meningitis with tuberculosis. *f, g* small, fat-loaded glial cells within the still existing white matter tract. *a, c, d, e* beginning accumulation of cells with fatty granules. *b* developed fatty granule cell.

Fig. 7. Storage of lipoid substances in the adventitial cells of a small vein in the white matter from a case of epilepsy.

**Table XXXV.**

Fuchsin light green staining. Zeiss homogeneous immersion 1/12. Fig. 1-5 ocular 6, Fig. 6 ocular 4.

Fig. 1. Ganglion cell from the ventral horn of a normal, older dog. In the cells are green Nissl slabs and fuchsinophil granules associated to the cytoplasm. At two locations are lipoid granules. At the rim of the cell and at processes are red labelled piles of neurosomes (8) visible. At the capillary are lipoid substances. In the tissue, one observes multiple myelin sheaths and axons. The rest is largely filled with protoplasmic processes of neighbouring ganglion cells, which are embedded by piles of neurosomes.

Fig. 2. Degenerated section from the ventral horn of a case of progressive chorea. In the middle a ganglion cell with the nucleus not sliced with multiple lipoid inclusions. Piles of neurosomes are only occasionally observed. The axon and the protoplasmic processes of the ganglion cells are hardly visible. The entire tissue is dispersed with greenish, roundish bodies (filled bodies).

Fig. 3. From a slice through the spinal cord of a hare which had died due to a bacterial infection with ascending paralysis. Border between dorsal horn and dorsal strand. In the neighbourhood of a capillary, partially in the perivascular space, partially in the nervous tissue are multiple larger filled bodies and among them larger ameboid glial cells with fuchsinophil and lipoid granules. Filled bodies are dispersed throughout the entire tissue.

Fig. 4. Cut through the superficial border layer of the spinal cord from a case of acute progressive paralysis which died due to sepsis. Instead of a surface layer formed by glial cells, we observe masses of filled bodies, which are also present as small piles between the myelinated fibers.

Fig. 5. Cut through the border layer of the spinal cord of an old case of paralysis. The strongly thickened surface layer is formed almost exclusively by perpendicular cut glial fibers.

Fig. 6. Cut through a degenerated corpus striatum of a case of progressive chorea. A delicate glial reticulum formed by a type of ameboid cells penetrates through the entire tissue.

Comments:

1. Syphilis caused dementia

(2) The dye of the Scharlach stain is carmine, a substance extracted from the female Dactylopius coccus, a cochineal insect native to tropical and subtropical America.

(3) hematoxylin solution introduced by Paul Ehrlich contains the dye hematin and aluminum potassium sulfate as a mordant; acetic acid controls the pH of the solution. It is used as nuclear stain. haematoxylin will also stain rough endoplasmic reticulum, ribosomes, collagen and myelin

(4) A mixture of phosphatides

(5) Balsam is the resinous exudate (or sap) which forms on certain kinds of trees and shrubs.

(6) The May-Grünwald stain is methylene blue acidified with eosin

(7) Flemming fixative:

Chromium trioxide 0.25g

Osmium tetroxide, 1% aqueous 10ml

Acetic acid, glacial 0.1 ml

Distilled water 90 ml

(8) Neurosome: cell body of a neuron

(9) The trade-name of a nitrocellulose, similar to pyroxylin, but prepared from wood-pulp instead of cotton. It is used as a substitute for collodion.

(10) refers to Chorea Huntington

(11) Dementia praecox (meaning a "premature dementia" or "precocious madness") is a psychiatric diagnosis that originally designated a chronic, deteriorating psychotic disorder characterized by rapid cognitive disintegration, usually beginning in the late teens or early adulthood. Over the years, the term dementia praecox was gradually replaced by the term schizophrenia, which initially had a meaning that included what is today considered the autism spectrum.

(12) Biography: https://www.achucarro.org/about-nicolas-achucarro/

(13) Pyknosis, or karyopyknosis, is the irreversible condensation of chromatin in the nucleus of a cell undergoing necrosis or apoptosis

(14) He obviously refers to Fig. 3 in Table one

(15) German: Rückbildungsalters

(16) German: Säurefuchsin-licht grün

(17) German: blitzartig entstehend, schnell und heftig verlaufend

(18) German word: endarteritisc

(19) Pellagra is a systemic disease caused by a severe deficiency of niacin (vitamin B3). It affects the whole body and can eventually lead to death. Primary pellagra is caused by a lack of niacin in the diet. It usually occurs in poor and food-limited populations. Secondary pellagra can occur when your body is unable to absorb the niacin from food.

(20) German: Trabantzellen

(21) German: Prädilektionsstelle

(22) The Bielschowsky stain is a silver impregnation technique used in histochemistry for the visualization of nerve fibers including multipolar interneurons. It also labels neurofibrillary tangles and senile plaques. It was developed by the German neurologist and neurohistologist Max Bielschowsky (1869–1940) by improving an older method developed by Ramon y Cajal.

(23) Probably synapses on spines

(24) German: Rückbildungsjahre

(25) (See the entry for “protagon”, in Webster’s Revised Unabridged Dictionary, Springfield, Mass.: G. & C. Merriam, 1913

So called because it was the first definitely ascertained principle of the brain. A nitrogenous phosphorized principle found in brain tissue. By decomposition it yields neurine, fatty acids, and other bodies.

(26) blindness, esp when occurring without observable damage to the eye

27) German: Über den zelligen Aufbau der Nervenfasern auf Grund mikro-histochemischer Untersuchungen

(28) Metachromasia is a staining of tissue elements in two colors from a solution of a single dye.

(29) Betz cells (also known as pyramidal cells of Betz) are giant pyramidal neurons located within the fifth layer of the grey matter in the primary motor cortex. These cells are the largest neurons in the central nervous system of up to 100 μm in diameter. Betz cells were first described by the Ukrainian Volodymyr or Wladimir Betz (1834-1894) in 1874.

(30) During a bacterial infection, the fonsils release a purulent matter termed stippchen in German.

(31) Corpora amylacea (from the Latin meaning "starch-like bodies") is a general term for small hyaline masses found in the nervous system and other organs of the body.

(32) pulmonary tuberculosis or a similar progressive systemic disease.

(33) Most of the pathology of syphilis is the consequence of widespread microscopic vascular compromise caused by obliterative endarteritis. Obliterative endarteritis is characterized by concentric endothelial and fibroblastic proliferative thickening, leading to obstruction of small arterial vessels.

(34) Tabes dorsalis is a slow degeneration of the nerve cells and nerve fibers that carry sensory information to the brain. Tabes dorsalis is the result of an untreated syphilis infection. Tabes dorsalis is much more common in men than women, and usually starts in mid-life.

(35) Stupor is unresponsiveness from which a person can be aroused only by vigorous, physical stimulation in contrast to coma.

(36) The Babinski or plantar reflex occurs after the sole of the foot has been firmly stroked. Babinski reflex is one of the normal reflexes in infants and in children up to 2 years of age. It disappears as the child gets older. It was first described by Joseph Babinski (1857-1932), a neurologist in Paris in 1896.
