# Supplementary material for: The functional role of glial cells in the pathologic brain as reviewed by Alois Alzheimer in 1910
Source: Mol Neurodegener. 2026 Feb 13;21:19. doi: 10.1186/s13024-026-00933-5 (PMC13005332; doi:10.1186/s13024-026-00933-5)
Supplement: Supplementary file 2 — Supplementary Material 2 [file 13024_2026_933_MOESM2_ESM.pdf]

Nissl-
Alzheimer,
Histologische
Arbeiten,
III, 3

NPA 13

ALLE RECHTE VORBEHALTEN.

Beiträge zur Kenntnis der pathologischen Neuroglia und ihrer Beziehungen zu den Abbauvorgängen im Nervengewebe.

Von Alois Alzheimer.

(Mit Tafel XXVIII—XXXV und 11 Textfiguren.)

1. Ziele der Untersuchung.

Wenn wir mit der Nisslschen Ganglienzellen-, der Weigertschen Glia- und Markscheiden-Färbung, den Methoden, welche wir gewöhnlich bei der Untersuchung des Zentralnervensystems anwenden, und welche uns ohne besondere Schwierigkeit gestatten, eine Paralyse, eine senile Demenz, eine Arteriosklerose und einige Formen der Hirnlues von einander zu unterscheiden, Gehirnschnitte von Individuen untersuchen, die an sog. einfachen Psychosen erkrankt waren, erweist es sich bis jetzt unmöglich, für die verschiedenen klinisch abtrennbaren Geistesstörungen kennzeichnende Veränderungen nachzuweisen. Es fehlt zwar auch hier gewiß nicht an pathologischen Befunden mannigfacher Art, aber trotz allen Bemühens — pathognomonische Merkmale lassen sich darunter nicht herausfinden.

So können wir mit der Nisslschen Methode recht verschiedenartige Veränderungen an den Ganglienzellen feststellen. Sie sind aber, wie das schon von Nissl selbst und vielen anderen betont worden ist, oft bei zweifellos verschiedenen Krankheiten übereinstimmend und bei verschiedenen Fällen, die sicher derselben Krankheit angehören, zuweilen verschieden. Dabei finden sich so vielfache, mannigfaltig kombinierte, durch Übergangsbilder verbundene Erkrankungsformen der Ganglienzellen, daß man wohl unter den pathologischen Zellbildern, wie sie die Nisslsche Methode darstellt, einzelne besonders typische Formen herausgreifen, aber eine erschöpfende

Darstellung derselben, namentlich in ihren Beziehungen zu den klinischen Krankheiten, nicht geben kann. Auch wissen wir von den wenigsten Ganglienzellenveränderungen, was sie bedeuten, ob sie schwere, nicht mehr ausgleichbare, oder leichte, vorübergehende Erkrankungszustände darstellen — kurz, welcher pathologische Wert ihnen zukommt. Diese Einsicht hat heute schon dahin geführt, daß man den früher in ihrer Bedeutung überschätzten Veränderungen der Ganglienzellen, wie sie im Nissl-Bild hervortreten, heute nicht einmal mehr diejenige Beachtung schenkt, die sie wirklich verdienen.

Wenn aber dann selbst unter den Ganglienzellen, welche die Rindenbreite ausfüllen, ein recht erheblicher Teil zugrunde gegangen ist, so können wir den Ausfall bei ihrer außerordentlichen Menge und verwickelten Anordnung kaum bemerken. Nicht besser kommen wir vorwärts, wenn wir unser Augenmerk den Nervenfasern zuwenden. Über pathologische Veränderungen an den Fasern, die sehr leicht Kunstprodukte bilden, wissen wir bis jetzt noch außerordentlich wenig, und ein Untergang von Fasern wird, wenn er nicht ganz erheblich ist, bei dem unendlichen Gewirr derselben, ebenso wie bei den Zellen, unserer Wahrnehmung entgehen, zumal die Erfahrung lehrt, daß die übriggebliebenen allmählich näher aneinander rücken.

Auch was uns alle bisher gebräuchlichen Methoden über die Glia erkennen lassen, reicht nicht hin, um genügende differentialdiagnostische Merkmale für die einzelnen Krankheiten zu liefern. Weigert hatte die Meinung vertreten, daß ein jeder Ausfall von nervösem Gewebe durch eine Wucherung der Glia und Neubildung von Gliafasern ersetzt würde. Wenn das richtig wäre, hätte man auch in einem Gliapräparate, wie in einem photographischen Negativ, Abweichungen in der Anordnung der nervösen Ausfälle bei den verschiedenen Krankheiten erkennen können. Aber diese Erwartungen haben sich nicht erfüllt. Vielmehr hat sich als unzweifelhaft herausgestellt, daß ein umfangreicher Untergang von nervösem Gewebe stattfinden kann, ohne daß Gliafasern neu gebildet werden. An Präparaten, die keinen Zweifel daran lassen, daß sie die faserige Glia in vollständiger Darstellung zeigen, finden sich zuweilen kaum mehr, ja weniger Gliafasern als normal, auch wenn offenbar recht zahlreiche nervöse Elemente geschädigt worden und zugrunde gegangen sind. Wir werden sogar sehen, daß auch bei manchen diffusen Krankheitsvorgängen selbst normale und schon pathologisch gebildete Gliafasern gleichzeitig mit nervösen Strukturen der Auflösung verfallen können. Eine pathologische Gliafaservermehrung gestattet also jedenfalls nur in einem beschränkten Grade einen Rückschluß auf nervöse Ausfälle. Wo sie vorhanden ist, dürfte ein Untergang von nervöser Substanz vorausgegangen sein, ihr Fehlen beweist aber nicht, daß kein Nervengewebe zerstört worden ist.

Nissl hat dann wohl zuerst darauf hingewiesen, daß bestimmte Nervenzellenerkrankungen mit ganz bestimmten Veränderungen an der Glia einhergehen. Seine Untersuchungen beschränkten sich dabei nicht auf die Gliafaserbildung, sondern zogen progressive und regressive Veränderungen an Plasma und Kern der Gliazellen mit in Betracht. Leider sind seine Ergebnisse niemals ausführlicher mitgeteilt worden; es ist wohl anzunehmen, daß er schon mancherlei von dem gesehen hat, was im nachfolgenden dargelegt werden soll. Aber die Bilder, welche die von ihm angewandte Methode liefert — er bediente sich hauptsächlich der seinen Namen tragenden Färbung — sind so subtile, daß es nur Nissls feiner Beobachtung möglich war, die morphologischen Zusammengehörigkeiten zu erkennen. Über die Bedeutung der einzelnen Erscheinungen aber konnten seine Präparate nur wenig aufklären.

Schließlich sind auch die krankhaften Veränderungen an den mesodermalen Gewerbsbestandteilen der Hirnrinde nicht so verschieden, daß sie die Abtrennung einzelner solcher Psychosen ermöglichten. Bei der Paralyse, der Hirnlues, der senilen Demenz und der Arterioklerose haben gerade sie wichtige Kennzeichen für die Differentialdiagnose gegeben. Darüber hinaus aber finden wir bei allen Prozessen, bei denen wir einen Untergang von Nervengewebe annehmen müssen, zuweilen neben leichten Wucherungserscheinungen an den Gefäßwandzellen im wesentlichen immer gleiche Veränderungen, vor allem eine Ansammlung von fettigen Produkten in den Zellen der Adventitia und des adventitiellen Lymphraumes.

So ist also geringe Aussicht vorhanden, mit den gleichen Hilfsmitteln, mit denen es der pathologischen Histologie gelungen ist, die paralyseähnlichen Erkrankungen von einander zu unterscheiden, bei den übrigen Psychosen wesentlich weiter zu kommen.

Aber nicht nur in Rücksicht auf die Aufgabe der pathologischen Histologie, die einzelnen Krankheiten abzutrennen und zur Verfeinerung der klinischen Differentialdiagnose mitzuhelfen, scheinen wir mit unseren Methoden — von einzelnen Gebieten abgesehen, wo sie

wohl immer noch weitere Erkenntnisse bringen dürften, z. B. bei noch nicht genauer bekannten paralyseähnlichen Erkrankungen, den verschiedenen Idiotieformen — an einer Grenze der Leistungsfähigkeit angelangt. Wir sehen immer wieder Kranke unter den schwersten kortikalen Krankheitserscheinungen sterben oder in unheilbaren Blödsinn verfallen, ohne daß uns die anatomische Untersuchung Befunde lieferte, welche einen solchen Ausgang erklärlich machten. Das beweist uns jedenfalls, daß wir noch ganz im Anfang der Entwicklung einer pathologischen Anatomie der Psychosen stehen. Pathologische Veränderungen sehen wir wohl stets in solchen Gehirnen; sie finden sich aber auch im Gehirn jedes Geisteskranken, der nur an leichten psychischen Störungen gelitten hat. Was uns also zweitens sehr empfindlich mangelt, ist die Möglichkeit, die Bedeutung, den pathologischen Wert der einzelnen Veränderungen richtig zu erkennen.

Ist es nun vielleicht auf anderem Wege möglich, diese Lücken auszufüllen? Man muß voraussetzen, daß die Verschiedenheit in der Erscheinungsform der einzelnen Geisteskrankheiten bedingt wird durch eine verschiedenartige Schädigung der nervösen Elemente der Hirnrinde, mag jetzt das Wesen des pathologischen Prozesses abweichend sein, oder nur seine Anordnung oder Ausbreitung. Das Endziel unserer Bestrebungen wird bleiben müssen, die Art und Lokalisation der nervösen Veränderungen festzustellen. Unsere heutige Technik erweist sich aber dafür noch unzureichend. Wir kennen bis jetzt keine Methode, die in einer so genügenden und sicheren Weise die feinsten nervösen Strukturen darzustellen vermöchte, daß sie für pathologische Untersuchungen brauchbar wäre. So muß man auf Umwegen weiterzukommen suchen.

Wenn es nun richtig ist — worauf neuere Untersuchungen immer mehr hindeuten —, daß die Glia außer den Gliafasern noch andere, feinere Strukturen bildet, welche die nervösen Elemente in zarteste Geflechte einhüllen, würde zu erwarten sein, daß unter krankhaften Verhältnissen gleichzeitig mit Veränderungen im Nervengewebe und namentlich mit Ausfällen von nervösen Elementen, diese an das nervöse Gewebe sich aufs engste anpassenden gliösen Bildungen Veränderungen erfahren. Nissls oben erwähnte Untersuchungen dürften schon für die Richtigkeit dieser Annahme sprechen.

Es ist also das Augenmerk darauf zu richten, die feineren Gliastrukturen noch besser darzustellen.

Weiter ist es auffallend, daß bei allen Psychosen, welche zur Verblödung führen, eine beträchtliche Vermehrung der fettigen Stoffe in der Hirnrinde zu finden ist. In manchen Fällen läßt sich eine ungeheure Menge von lipoiden Substanzen in den Zellen der Adventitia und in der Pia nachweisen. Zahlreiche Zellen sind dort mit fettigen Stoffen beladen oder sogar in Fettkörnchenkugeln umgewandelt. Daß das Fett in diese Zellen nur durch eine Fettentartung der eigenen Zellsubstanz gelangt, scheint dabei durchaus unwahrscheinlich. Seine Menge ist dafür oft viel zu beträchtlich. Daher liegt die Annahme nahe, daß es aus dem Nervengewebe herstammt und dort nur aufgespeichert wird. So stellt sich die weitere Aufgabe, die Wege aufzusuchen, auf welchen die lipoiden Substanzen in die bezeichneten Zellen gelangen. Dabei wird man besonders auch nach intermediären Abbaustoffen suchen müssen, welche etwa zwischen den normalen Gewebsbestandteilen der Hirnrinde und diesen Endprodukten in den Zellen der Lymphscheide und der Pia durchlaufen werden.

So scheinen sich Wege anzudeuten, feinere Degenerationsvorgänge in der Hirnrinde festzustellen, auf ähnliche Art, wie man schon lange mit der Marchi-Methode, die nur für diese Zwecke viel zu grob erscheint, aus Zerfallsprodukten der Markscheide, den Untergang ganzer Faserbündel durch das zentrale Nervengewebe hindurch verfolgen konnte.

Bei der Suche nach pathologischen Stoffen fanden sich auch solche in reichlicher Menge und sehr verschiedener Art. Dabei ließen sich noch mancherlei andere bemerkenswerte Erscheinungen feststellen. Es ergab sich vor allem, daß die beiden eben bezeichneten Wege vielfach mit einander führen, da sich ein großer Teil wenigstens der pathologischen Produkte in Gliazellen, z. T. besonderer Form, eingeschlossen findet.

Man hat schon viel davon gesprochen, daß der Glia außer ihrer Rolle als Stützsubstanz auch nutritive Aufgaben im Nervengewebe zukämen. Soviel auch dafür spricht, die Beweise dafür sind noch recht dürftig und die Art dieser Aufgabe noch ganz dunkel. Immerhin muß uns die Möglichkeit einer solchen biologischen Bedeutung der Glia die Frage nahe legen, ob alle die besonderen Stoffe, die wir in ihr antreffen, Abbauprodukte des nervösen Gewebes sind, oder ob sie nicht wenigstens teilweise durch pathologische Vorgänge zurückgehaltene oder veränderte Nährstoffe des Nerven-

gewebes, kurz Produkte eines gestörten Stoffwechsels der nervösen Substanz darstellen.

Bei dem Versuche, die mannigfachen Befunde zu erklären, ergeben sich mancherlei Fragen, die noch ungelöst bleiben müssen.

Die vorliegenden Studien beanspruchen auch nichts weiter zu sein als ein Versuch, etwas tiefer einzudringen in die pathologischen Geschehnisse im Zentralnervensystem, besonders in der Hirnrinde.

2. Methodik der Untersuchungen.

Sehr bald wurde es klar, daß die bei der Untersuchung des zentralen Nervensystems gewöhnlich geübten Methoden nicht ausreichten, dem eben vorgezeichneten Ziele näher zu kommen. Alle die Versuche anzuführen, die in mehreren Jahren angestellt wurden, hinreichend und besser darzustellen, auf was es ankam, würde wenig Zweck haben. Viele Darstellungsmethoden konnten auch wieder verlassen werden, weil sich einfachere und bessere Wege finden ließen. So wird mancher der Herren, die im Laboratorium der hiesigen Klinik gearbeitet haben, jetzt andere Färbungen angegeben finden, als ich ihm früher gezeigt habe. Er wird, wenn er diese versucht, wohl zufriedener damit sein. Oft erwies es sich auch als wünschenswert, die gleichen Dinge mit möglichst verschiedenen, auf ganz abweichenden Prinzipien beruhenden Färbungen oder mit anderen Farbmischungen darzustellen, teils um aus den färberischen Reaktionen über die Natur der dargestellten Stoffe Anhaltspunkte zu bekommen, teils um festzustellen, ob Stoffe, die sich bei Anwendung einer Färbemethode gleich färben, bei Benutzung anderer Farbgemische nicht verschiedene Farben annehmen, also verschiedener Art sind. Aber auch den zuletzt angewandten Methoden haften noch recht empfindliche Mängel an. Durch weitere Versuche hätten sie sich vielleicht einschränken oder beseitigen lassen. Es war mir aber genug, wenn sie nur weitere Erkenntnisse ermöglichten.

Wie man sehen wird, sind die Darstellungsweisen keine völlig neuen, sondern in ihrer Grundlage alte, und nur den besonderen Zwecken angepaßt. Ich will sie hier zusammenstellen und mit Zahlen bezeichnen, auf die ich der Kürze wegen später wieder verweisen kann. Da es mit in erster Linie galt, die Stoffe darzustellen, die beim Abbau des Nervengewebes, der Ganglienzellen, Achsenzylinder und Markscheiden gebildet werden, mußte man besonders auf lipoide Körper achten. Die bei der Untersuchung des Zentralnervensystems meist angewandte Fixierung mit Alkohol oder auch die Nachbehandlung anders fixierten Materials mit Alkohol, Äther, Chloroform, Xylol zum Zwecke der Einbettung, mußte diese Stoffe zum Teil ausziehen und damit ihre Darstellung unmöglich machen. Deswegen war es angezeigt, zunächst von der Anwendung fettextrahierender Mittel ganz

abzusehen. So wurde es zuerst mit frischem Material versucht. Die Schwierigkeiten aber, die sich dabei ergeben, sind noch weit größer als bei der Untersuchung anderer frischer Gewebe. Schon bei der Quetschung entstehen so viele Kunstprodukte, daß die Deutung der Befunde ungemein erschwert wird. Der Zusatz irgend welcher Reagentien und Farben vermehrt dann die schon vorhandenen Quellungen oder erzeugt noch dazu unnatürliche Schrumpfungen. Das gequetschte Material verändert sich weiter rasch unter unseren Augen, so daß die Zeit zum Studium der Präparate zu kurz bleibt. Ihre Vergänglichkeit macht schließlich auch Vergleichungen unmöglich, die bei so verwickelten Verhältnissen unerläßlich sind. So konnte ich mich nur davon überzeugen, daß es pathologische Zelleinschlüsse gibt, die wir auch mit den später angewandten Fixierungen und Färbungen noch nicht darstellen können. Nur an der Pia kann man ganz lehrreiche Präparate gewinnen; besonders erweist sich hier eine Färbung mit Neutralrot empfehlenswert. Im übrigen war es nötig, zu fixiertem Material überzugehen, und zunächst wurde an Formolgefrierschnitten gearbeitet. Wir benutzen dieselben noch heute in erster Linie für die Scharlachfärbung nach Herxheimer, die wir in folgender Weise anwenden:

I. Mit einer Mischung von

absolutem Alkohol	70
Natronlauge 10 proz.	20
Aqu. destill.	10

füllt man ein Reagenzglas dreiviertel und gießt Scharlach R im Überschusse zu, verkorkt und läßt die Mischung einige Stunden stehen, während welcher man öfter umschüttelt. Dann ist die Farbe fertig und behält etwa 36 Stunden ihre beste Färbekraft. Zur Färbung filtriert man von der Farblösung in ein Uhrschälchen, das zur Verhinderung von Verdunstung sofort mit einem zweiten bedeckt wird. Nun bringt man die Schnitte in die Farbe, deckt wieder zu und erwärmt zwei oder dreimal vorsichtig, bis sich das obere Glas beschlägt. Dann läßt man $^1/_4$ Stunde stehen, bringt die Schnitte in destilliertes Wasser, von da 10 Minuten in stark verdünntes Ehrlichsches Hämatoxylin, in destilliertes Wasser, in Brunnenwasser 1—2 Stunden, fängt sie mit dem Objektträger auf und bettet in Glyzerin ein. Bei einiger Übung kann man leicht von Scharlachniederschlägen völlig freie Präparate erhalten. Nach einigen Wochen beginnen viele zu verderben, indem sich das Scharlach in Körnchen oder Kristallformen wieder ausscheidet. Einige Präparate erhalten sich Monate, ja selbst Jahre lang ziemlich unverändert.

II. lassen sich an den Formol-Gefrierschnitten gewisse Stoffe darstellen, die ich basophil metachromatische nennen will, und die mit den von Reich als Protagon benannten Stoffen verwandt, zum Teil auch identisch sein dürften. Es ist erforderlich, nicht zu altes Formolmaterial zu verwenden, da diese Stoffe bei langem Aufenthalt

in Formol Veränderungen einzugehen scheinen. Man färbt die Schnitte in einer 1 proz. Lösung von Toluidinblau oder Kresylviolett entweder 1 Stunde in der Kälte oder durch leichtes Erwärmen und Wiederabkühlen der Präparate, bringt die Schnitte in destilliertes Wasser, von da in Alkohol, Xylol und bettet in Balsam ein. Es läßt sich bei einiger Übung ein Zeitpunkt finden, in welchem die Markscheiden hinreichend entfärbt und die fraglichen Stoffe auch in ihren feineren Teilchen noch deutlich gefärbt sind. Die Präparate gehen rasch zugrunde. Einiges von denselben Stoffen kann man auch in Alkoholschnitten sehen, die mit der gewöhnlichen Toluidinblaufärbung behandelt sind. Jedoch ist bei der letzteren Methode immer zu berücksichtigen, daß Kunstprodukte, durch den Alkohol ausgezogene und wieder ins Gewebe ausgefällte Markscheidenbestandteile sich mitfärben.

III. Gewisse degenerative Produkte, die einigen besonderen Krankheiten zugehören, lassen sich an Formolgefrierschnitten mit Lösung von May-Grünwaldschem Farbstoff in Methylalkohol ziemlich elektiv darstellen. Man untersucht die Präparate in Glyzerin, wo sie aber rasch zugrunde gehen. Durch schnelles Überführen in wasserfreies Aceton und Xylol, sowie durch kurze Vorbehandlung der Schnitte mit Osmiumsäure, zwei Tropfen der 2 proz. Lösung in ein Uhrschälchen mit Wasser, kann man die Färbung haltbarer machen. Die Färbung läßt sich auch an Flemming-Schnitten anwenden. Auch diese Stoffe verändern sich nach einiger Zeit beim Liegen der Gehirnstücke in Formol und verlieren ihre färberische Eigenart.

Für weitere Untersuchungen ergab sich, daß die Färbbarkeit des Gliazellenplasma durch langes Liegen in Formol störend beeinträchtigt wurde. Deshalb wurde in Orthscher Flüssigkeit behandeltes Material verwendet. Die Methode II ließ sich auch hier anwenden und wird am besten nach der von Reich angegebenen Art ausgeführt. Aber trotzdem blieb noch manches unbefriedigend. Nach vielen Versuchen mit anderen Fixierungsflüssigkeiten zeigten sich die in Weigertscher Gliabeize fixierten Stücke für die Darstellung mancher Plasmastrukturen sehr brauchbar. Sie waren auch verhältnismäßig frei von störenden Schrumpfungen. Wir verwenden stets die Weigertsche Formel mit Fluorchrom. Sofort in die Gliabeize eingelegtes Material gibt viel bessere Bilder als erst in Formol fixiertes. Auch hier wurden meist Gefrierschnitte verwendet. Damit das Messer nicht von der Säure beschädigt wird, ist es nötig, kleine Stückchen mindestens 2 Stunden in fließendem Wasser auszuwaschen. Längeres Auswaschen, bis 12 Stunden, beeinträchtigt die Färbung nicht. Hauptsächlich gelangen zwei Methoden zur Anwendung.

IV. Man bringt die Gliabeize-Gefrierschnitte von 10 μ Dicke 1. kurz in destilliertes Wasser, 2. 2 Minuten in Wasser, dem einige Tropfen Eisessig zugesetzt sind (etwa 1 Tropfen auf 10 ccm), 3. direkt in eine stark verdünnte Lösung von Malloryschem Hämatoxylin

10 ccm 10 proz. Phosphor-Molybdänsäure,
1,75 g Hämatoxylin,
5 g Karbolsäure,
200 g Wasser.

Die Farbe ist erst nach 6—8 Wochen genügend gereift, im Brutofen in der Hälfte Zeit. Mit dem Alter nimmt ihre Färbekraft sehr zu. Man braucht dann nur 4—5 Tropfen auf ein großes Uhrschälchen destillierten Wassers. Die Lösung soll so viel Farbe enthalten, daß sie im Uhrschälchen undurchsichtig wird. Zu alt gewordene Farblösung gibt einen unschönen blauen Farbton. Im Hämatoxylin bleiben die Schnitte etwa 2 Minuten, 4. Überführen in destilliertes Wasser (einige Minuten), steigenden Alkohol, Xylol.

Die Farbe der Schnitte muß rötlichblau sein. Blaue Schnitte sind weniger elektiv. Die rotblaue Färbung erhält man sicherer durch die Vorbehandlung mit angesäuertem Wasser. Sie geht verloren durch zu langes Liegen der Schnitte in destilliertem Wasser und Alkohol nach der Färbung. — Diese Methode gibt eine brillante Darstellung des Plasmaleibes der amöboiden Zellen, weiter gewisser Granula dieser Zellen. Leidlich gut treten auch Gliafasern hervor. In normalen Rindenpräparaten sieht man die zarten protoplasmatischen Verästelungen der Gliazellen. Weiter wird gut dargestellt der Inhalt der perivaskulären Räume. Daneben färben sich auch Ganglienzellen, Achsenzylinder und Gefäße. Die Präparate sind besonders wertvoll als Übersichtspräparate. Man kann sich sehr rasch einen Überblick über den Zustand der Glia verschaffen. Die Methode ist aus der Eisathschen hervorgegangen. Eisathsche Präparate sind manchmal noch schöner und elektiver, häufiger aber weniger gut. Außerdem ist die Eisathsche Methode viel umständlicher. Jedenfalls ist das Plasma der amöboiden Zellen bei dieser Modifikation viel satter gefärbt. Die Granula und Inhalte der perivaskulären Räume werden weit besser als mit der ursprünglichen Methode dargestellt, die starken Schrumpfungen, welche manchmal die Eisathschen Präparate unbrauchbar machen, fehlen gänzlich. Tafel XXVIII stellt mit dieser Färbung gewonnene Präparate dar. Sie sind gut haltbar.

V. Man bringt die gleichen Schnitte

1. auf 2—12 Stunden in eine gesättigte wässerige Lösung von Phosphor-Molybdänsäure;
2. wäscht kurz zweimal in destilliertem Wasser;
3. bringt die Schnitte in Mannsche Lösung
   35 ccm 1 proz. wässeriger Methylblaulösung,
   35 ccm 1 proz. wässeriger Eosinlösung,
   100 ccm destillierten Wassers;
4. spült kurz in destilliertem Wasser ab, bis die Schnitte keine Farbwolken mehr abgeben;
5. bringt die Schnitte in 96 proz. Alkohol (1—2 Minuten), bis ein hellblauer Farbton eintritt;
6. überführt in absoluten Alkohol und Xylol.

Man findet dann das Plasma der amöboiden Zellen heller oder dunkler blau gefärbt, gewisse Granula desselben dunkelblau, Vakuolen rötlich, die Achsenzylinder sind blau oder rötlichblau, degenerierende leuchtend rot oder schwarzblau, Gliafasern hellblau, Ganglienzellen dunkelblau, Bindegewebsfasern tiefblau, Blutkörperchen leuchtend rot. Das Mark färbt sich rot, wird aber bei längerer Differenzierung fast farblos.

Die Methode gibt etwas zartere und feinere Bilder wie die Methode IV. Eine Überfärbung, die dort leicht eintritt, ist hier nicht zu befürchten. Die feinen protoplasmatischen Verästelungen der normalen Gliazellen der Hirnrinde sind nicht sichtbar. Tafel XXIX stellt mit dieser Färbung gewonnene Präparate dar.

Die Präparate sind monatelang haltbar, blassen aber dann allmählich, besonders in den roten Tönen, etwas ab.

Weiter wurde es wünschenswert, auch mit dünneren Schnitten, als sie das Gefriermikrotom anzufertigen gestattet, zu arbeiten. Namentlich erwiesen sie sich nötig, um die Beziehungen der Zellen zu den Gefäßen anschaulich zu machen, welche an Gefrierschnitten vielfach ausfallen und an dicken Schnitten in ihren Einzelheiten nicht genügend aufgelöst werden können.

VI. Am besten erwies sich hier folgende Methode:

1. Fixierung des Materials 24 Stunden in 10 Proz. Formollösung;
2. von da direkt 8 Tage in Flemmingsche Flüssigkeit;
3. 12—24 Stunden Auswaschen in fließendem Wasser;
4. Einbetten in Paraffin von 58° Schmelzpunkt;
5. Schnitte von 2—3 μ Dicke;
6. Aufkleben der Schnitte mit Wasser auf dem Objektträger;
7. Entparaffinieren und bis 96° Alkohol überführen;
8. 1 Stunde im Brutofen bei 58° in gesättigter wässeriger Lösung von S.-Fuchsin;
9. Abwaschen zweimal im Wasser, bis keine Farbe mehr abgeht;
10. Eintauchen unter Bewegung des Objektträgers in eine Lösung von gesättigter alkoholischer Pikrinsäurelösung 30, Aqua destill. 50, 10—20 Sekunden;
11. sorgfältiges Abwaschen zweimal in Wasser;
12. Einlegen in eine gesättigte wässerige Lichtgrünlösung 20—50 Minuten;
13. schnelles Abspülen in Wasser, rasches Überführen durch 96° Alkohol, absoluten Alkohol in Xylol.

Die Methode des Altmannschen nachgebildet und in ähnlicher Weise schon von Galeotti und Levi benützt, gibt ungemein hübsche Präparate. Das Lichtgrün ziehe ich dem sonst benutzten Methylgrün vor, weil es schärfere Farbenkontraste gibt. Das Lichtgrün verdrängt schon allein das S.-Fuchsin aus gewissen Gewebsteilen. So habe ich

es anfangs ohne vorherige Pikrinsäuredifferenzierung angewandt. Aber die letztere hat doch dadurch große Vorteile, daß sie gleichmäßiger gefärbte Präparate gibt.

Die zweckmäßigste Dauer der Lichtgrünfärbung muß am besten für jedes Material ausgeprüft werden. Ich färbe meistens 30 Minuten. Öfters aber gibt auch eine etwas längere Färbung noch bessere Präparate. Gute Präparate zeigen weder eine rote noch grüne Färbung, sondern einen violetten Ton.

Die Zelleiber der amöboiden Gliazellen sind grün gefärbt, gewisse Granula ihres Zelleibes leuchtend rot, die lipoiden Cystchen zart braun. Sonst treten im Gewebe, das in seinen plasmatischen Teilen grün gefärbt ist, noch zahlreiche andere rote Körnchen zutage; die Gliafasern und die roten Blutkörperchen sind rot, die Markscheiden ungefärbt. Sehr hübsch färben sich auch die Elemente der Gefäßwand, besonders die Bindegewebsfasern der Adventitia und gewisse Stoffe im perivaskulären Raum.

Stücke des Zentralnervensystems, die schon länger in Formol gelegen haben, geben deswegen weniger klare Bilder, weil jetzt durch das Osmium vielerlei Schollen und Körner sich schwärzen. Will man solches Material verarbeiten, so benutzt man zweckmäßig statt der Flemmingschen Lösung ein einfaches Chromessigsäuregemisch. Man erhält auch so ganz brauchbare Präparate, wenn man noch vorsichtiger mit Pikrinsäure differenziert und etwa die halbe Zeit mit Lichtgrün färbt.

Schließlich kann man statt der S.-Fuchsin-Lichtgrünfärbung mancherlei andere Farbenzusammenstellungen mit gutem Erfolg anwenden. Die angegebene Methode schien mir die kontrastreichsten Bilder zu liefern. Tafel XXX gibt mit dieser Methode gefärbte Präparate wieder.

VII. Zur Darstellung der fuchsinophilen Granula und der Neurosomen kann man mit Vorteil auch nachfolgende Methode anwenden.

Man behandelt wie in Methode VI bis zu Punkt 7, führt aber die Schnitte bis in destilliertes Wasser.

8. 1 Stunde im Brutofen bei 35° in gesättigte wässerige Lösung von essigsaurem Kupfer;
9. zweimaliges Abspülen in destilliertem Wasser;
10. $^1/_2$ Stunde in 10prozentige alkoholische Hämatoxylinlösung 10 ccm, destilliertes Wasser 87 ccm, gesättigte Lithionkarbonikumlösung 3 ccm;
11. kurzes Abspülen im Wasser;
12. Überführen durch Alkohol in Xylol.

Von hellem Grunde heben sich die verschiedenen Granula schwarzblau gefärbt ungemein scharf ab. Das Plasma ist leicht gelbgrau oder blau gefärbt, so daß die Zellgrenzen, Achsenzylinder genügsam hervortreten.

Zur Darstellung mancher Abbauprodukte ist schließlich eine Weigertsche Markscheidenfärbung nützlich.

VIII. Man legt dazu nicht zu altes Formolmaterial 6 Tage in WEIGERTsche Markscheidenbeize im Brutofen, wäscht kurz aus, bettet in Photoxylin ein, kupfert die Schnitte, färbt mit Lithionhämatoxylin und differenziert in Boraxferrizyankaliumlösung.

Gewisse lipoide Stoffe lassen sich mit der folgenden Methode gut zur Anschauung bringen, die im hiesigen Laboratorium von Frau Dr. MOOERS und Herrn Dr. MINKOWSKI ausgearbeitet worden ist, und nach MINKOWSKI am zweckmäßigsten so ausgeführt wird:

IX. Alkoholschnitte, auch in Photoxylin eingebettete, werden in Wasser überführt,

2. in Karbolfuchsinlösung erhitzt, bis Bläschen aufsteigen;
3. aus der noch warmen Lösung in destilliertes Wasser gebracht, bis keine Farbwolken mehr abgehen;
4. in NISSLs Seifenmethylenblau 1 Minute bei Zimmertemperatur gefärbt;
5. in Anilinölalkohol usw. differenziert, wie bei den NISSL-Präparaten.

Die Schnitte zeigen das NISSL-Bild in befriedigender Weise, daneben gewisse lipoide Stoffe in leuchtend roter Färbung.

X. Für die Darstellung der fibrinoiden Granula wird die WEIGERTsche Gliafärbung in einer geringen Modifizierung angewandt, die meines Erachtens auch die Fasern am befriedigendsten darstellt.

Das Material aus Gliabeize, das nicht vorher in Formol eingelegt war, wird rasch in 3—4 Tagen in Photoxylin eingebettet, gleich geschnitten, die Schnitte sofort weiter behandelt. Aus dem Kalium hypermanganicum kommen die Schnitte auf 10 Minuten in die Chromogen-Ameisensäure-Natrium-Sulfitlösung, doch setze ich nicht 10 ccm Natriumsulfit, sondern nur 2 zu, dann werden die Schnitte in Wasser abgespült, auf dem Objektträger aufgefangen und nach Vorschrift gefärbt. Man bekommt regelmäßiger gute Präparate, als wenn man genau die WEIGERTsche Vorschrift befolgt.

Zur Darstellung des Glykogens ist in absoluten Alkohol eingelegtes Material zu verwenden. Die Färbung geschieht am besten nach den von BEST angegebenen Methoden.

3. Über den normalen Bau der Neuroglia.

Den Arbeiten WEIGERTS, die so sehr unsere Kenntnisse von der Stützsubstanz des zentralen Nervensystems geklärt und gefördert haben, folgten bald neue Untersuchungen über die Glia, die uns neben den Gliafasern noch andere gliöse Bildungen schilderten. Das meiste ist hier wohl von HELD gearbeitet worden. Diese besonderen Gliastrukturen ebenso sicher und elektiv darzustellen, wie es WEIGERT mit den Fasern gelungen war, ist aber bisher nicht

möglich gewesen. So bleibt uns der Bau dieser feineren Glia auch heute noch nach mancher Richtung dunkel.

Wenn wir aber das Verhalten der Glia unter gewissen pathologischen Verhältnissen besprechen wollen, müssen wir klar darüber sein, welche Vorstellung wir uns von ihrem normalen Bau zu machen haben. Da WEIGERT in seiner Arbeit „Beiträge zur Kenntnis der menschlichen Neuroglia" die Entwicklung unseres Wissens eingehend dargestellt und der Lehre von der Stützsubstanz des zentralen Nervengewebes eine neue Fassung gegeben hat, werden wir von WEIGERTS Darlegungen ausgehen können.

Seine Schilderung von dem Bau des nervösen Stützgewebes fand zunächst Widerspruch in bezug auf seine Annahme, daß die Gliafasern eine echte Interzellularsubstanz seien. Tatsächlich kann man sich ohne viel Schwierigkeit überzeugen, daß manche Gliafasern den Protoplasmaleib der Gliazellen durchlaufen, daß an vielen dem Anscheine nach freiliegenden Fasern doch ein Saum oder eine Hülle einer protoplasmaähnlichen Substanz liegt, und daß die allermeisten übrigen nur Randversteifungen eines komplizierten protoplasmatischen Zelleibes darstellen. Es reicht zu diesem Nachweis jede Färbemethode hin, die neben den Fasern in einem genügend abweichenden Ton das Protoplasma zu färben vermag. WEIGERTS Methode stellt die Gliafasern zu elektiv dar, als daß sie die Beziehungen derselben zu den Protoplasmastrukturen genügend kenntlich machen könnte. So ist wohl die Auffassung WEIGERTS, daß die Gliafasern eine interzelluläre Substanz seien, nicht mehr als richtig anzuerkennen.

HELD, der dem Studium des Gliazellenprotoplasmas und seinem Verhältnis zu den Gliafasern besondere Aufmerksamkeit zugewendet hat, konnte dann zeigen, daß die Glia neben den Fasern noch andere Bildungen hervorbringt, die ihr den Charakter eines synzytialen Gewebes verleihen. WEIGERTS Darstellung der Glia erfährt damit eine wesentliche Ergänzung. Die zarten protoplasmatischen Fortsätze der Gliazellen verästeln sich, und die Verästelungen verschiedener Zellen treten zu netzartigen Bildungen zusammen. Diese gliösen Netze durchziehen das ganze zentrale Nervensystem. Nicht nur die Füllnetze BETHES, für welche dieser selbst die Möglichkeit offen gelassen hat, daß sie Gerinnungsprodukte darstellen, sondern auch die GOLGI-Netze, welche BETHE mit nervösen Strukturen in enge Beziehung brachte, erklärt HELD für Teile des Gliaretikulums. Dabei wird mehrfach betont, daß dieses Netzwerk wohl nicht aus einem

einfachen Zellprotoplasma gebildet sei, sondern aus einer irgendwie anders beschaffenen, besonderen Substanz.

Als eigenartige synzytiale Gliabildungen beschreibt HELD dann noch die Gliagrenzhäute, die Membrana neurogliae superficialis und perivascularis, von denen die eine die Oberfläche des Zentralorganes gegen die Pia abschließt, die andere die Gefäße einscheidet, welche durch dasselbe hindurchziehen. Sie sollen durch mosaikartige Zusammenfügungen von Teilen des Gliaretikulums und Gliafasern gebildet werden, die sich mit ihren Enden in Form aufgefaserter Gliafüße in die Grenzhaut einsenken.

Nicht überall im normalen Nervensystem läßt sich, wie schon HELD betont, das Gliaretikulum in gleicher Deutlichkeit darstellen. Die tiefere Hirnrinde scheint eine der ungeeignetsten Stellen für seinen Nachweis. Am schönsten tritt es in der oberflächlichsten Schicht der Hirnrinde hervor. Man kann hier leicht mit vielerlei Methoden eine netzartig angeordnete Substanz darstellen, durch deren Maschen die Markfasern hindurchziehen, und in deren Balken die Gliafasern eingelagert sind. Fortsätze der Plasmaanhäufungen, welche um Gliakerne herum liegen, stehen mit diesen Netzen in Beziehungen und verlieren sich in ihnen, ohne daß eine Zellgrenze festzustellen wäre. Gegen die erste Ganglienzellenschicht zu wird das Maschenwerk rasch enger und feiner. In der tieferen Rinde habe ich es bis jetzt mit keiner Methode isoliert darstellen können. Man kann sich davon überzeugen, daß hier die feinsten nervösen Strukturen mit ihm zusammen gefärbt sind. Im Mark ist es wieder besser zu sehen. An verschiedenen Stellen der Hirnrinde scheint seine Struktur etwas verschieden. So läßt es sich in der vorderen Zentralwindung etwas deutlicher erkennen als an den meisten anderen Orten, wohl weil es hier etwas grobmaschiger ist. Auch darin ist HELD wohl beizupflichten, daß das Retikulum der Glia kein gewöhnliches Zellprotoplasma ist. Denn mit den Methoden, mit denen sich das eigentliche Plasma aufs intensivste färbt, wird es nur in einem ganz zarten, blassen Ton dargestellt. Je nach den angewandten Fixierungsmethoden erscheint es mehr körnig oder mehr in zarter, hautartiger Ausbreitung. Die Meinung HELDS, daß BETHES GOLGI- und Füllnetze diesem Gliaretikulum entsprechen, erhält durch mancherlei Beobachtungen gute Begründung. Jedenfalls sieht man die eigentlichen GOLGI-Netze überall in die weiteren Maschen des Füllnetzes übergehen und diese selbst Markfasern einhüllen und Schnürringe bilden, wie das HELD eingehend geschildert hat.

So wäre also die BETHEsche Methode eine der leistungsfähigsten, das Gliaretikulum mit genügender Elektivität zur Anschauung zu bringen. Ich muß aber bemerken, daß sie mir am pathologischen Material fast völlig versagt hat. Denn entweder ließ sich ein Retikulum färben, das vom normalen nur wenig abwich oder es war überhaupt nicht darzustellen. Bei der Launenhaftigkeit und Unbeständigkeit der Methode kann man aus diesem Umstand keine sehr weitgehenden Schlüsse ziehen. Jedenfalls aber haben sich einstweilen die Versuche als wenig aussichtsvoll erwiesen, mit Hilfe der BETHEschen Methode über das Verhalten der synzytialen Glia unter pathologischen Umständen wichtige Aufschlüsse zu erhalten.

Dagegen gelingt es viel leichter unter pathologischen Verhältnissen, dieses Gliaretikulum mit mancherlei anderen Methoden einigermaßen sichtbar zu machen, so in stark atrophischen Rinden, in Stellen, die durch benachbarte Herde verödet sind. Auf Tafel XXXV, Fig. 6 sehen wir ein solches Gliaretikulum aus dem Streifenhügel eines Falles von progressiver Chorea. OPPENHEIM hat kürzlich mit einer sehr einfachen, aber auch keineswegs elektiven Methode dieses Retikulum gefärbt und seine Veränderungen in den Herden der multiplen Sklerose beschrieben. Ich will aber zunächst nicht weiter in die Schilderung dieser netzigen Gliastrukturen eingehen, weil sie für die akuten Veränderungen, die hier hauptsächlich beschrieben werden sollen, offenbar eine geringere Bedeutung haben. Sie scheinen erst nach Ablauf des akuten Prozesses wieder repariert zu werden, und öfters in einer gröberen, von der normalen abweichenden Form. So kann ihre Darstellung für die Erkenntnis feinerer Ausfälle im Nervengewebe von wesentlichem Nutzen werden. Daß diese retikulären Gliabildungen an den Abbauvorgängen keinen wesentlichen aktiven Anteil nehmen, dürfte vielleicht daran liegen, daß sie eben ein modifiziertes Plasma darstellen, dem nur die Aufgabe einer Hüll- und Stützsubstanz zufällt.

Wenn aber gegen den retikulären Bau der Glia auch heute kaum mehr Zweifel erhoben werden können, so muß doch der Meinung entgegengetreten werden, daß alle oder die Mehrzahl der Gliakerne, die wir im zentralen Nervensystem, insbesondere auch in der grauen Substanz antreffen, in Protoplasmaanhäufungen eines solchen

überall verbreiteten gliösen Retikulums liegen und alle Gliaelemente zugunsten dieses Netzwerkes ihre Individualität völlig verloren hätten.

Schon die GOLGI-Methode hat uns Gliazellen mit ungemein zahlreichen langen Fortsätzen dargestellt. In Schnitten aus der Markleiste, in welchen die Glia gut inkrustiert ist, sehen wir dicht beieinander liegende Gliazellen, die nach allen Seiten hin eine wahre Unzahl von außerordentlich dünnen langen Fortsätzen ausstrahlen. In ihrer Gesamtheit bilden sie einen dichten Filz sich überkreuzender Fasern. An so zahlreichen Zellen sehen wir Fortsätze, die zu Gefäßen hinziehen, daß wahrscheinlich eine jede Zelle mit dem Blutgefäßapparat, oder genauer gesagt den perivaskulären Grenzmembranen, in eine oder mehrfache Verbindung tritt und daß nur die Dünne des Schnittes, die Größe des von einer Zelle beherrschten Gebietes und die Unvollständigkeit der Inkrustation diese Beziehungen nicht an allen Elementen erkennen läßt. Wir wissen nun zwar von den nervösen Elementen her, daß die GOLGI-Methode nicht jede Zelle bis in ihre letzten Enden imprägniert. So kann der Einwand erhoben werden, daß möglicherweise ein von den dargestellten Fortsätzen der Gliazellen gebildetes Retikulum nicht zur Anschauung gebracht wird, vielleicht weil es von einer andersartigen Substanz gebildet ist, die nicht gleich günstige Bedingungen für die Imprägnation bietet. Aber die ganzen Bilder sehen doch recht wenig danach aus. Nie sehen wir, wie Fasern, die übereinander wegziehen, miteinander in Verbindung treten; warum die unendlich vielen, sich überkreuzenden Fasern, wenn das Retikulum das Wesentlichste wäre?

Mögen aber selbst diese Bedenken sich als grundlos erweisen, darüber können uns die GOLGI-Bilder jedenfalls nicht trügen, daß in dem Gliaretikulum sehr komplizierte Gliazellen eingeschlossen sind.

Dasselbe beweisen uns andere Färbungen, z. B. die von EISATH angegebene Methode und ihre von mir beschriebene Modifikation. Da es sich hierbei um einfache Färbungen handelt, entfallen bei der Deutung der Bilder noch manche Bedenken, die sich gegen die GOLGI-Methode erheben lassen. Auch mit diesen Färbungen gelingt es, verwickelt gebaute Gliazellen, besonders in der grauen Substanz, zur Anschauung zu bringen (Tafel XXVIII, Fig. 1*a*, *b*). Man sieht den Kern umgeben von einem schmalen Saum von Protoplasma, von welchem aus nach allen Seiten protoplasmatische Fortsätze ausstrahlen. Sie verzweigen sich, werden dabei immer dünner und immer zarter gefärbt, bis sie schließlich nicht weiter verfolgbar sind.

Man ist ja wohl nirgends sicher, das Ende der Aufzweigungen eines solchen Zellfortsatzes vor sich zu haben. Aber ein Übergang der Fortsätze einer Zelle in die einer anderen oder in ein Netz ist auch hier nirgends wahrzunehmen. An besonders gelungenen Präparaten kann man an einzelnen Zellen die Fortsätze wohl ebenso weit verfolgen wie an den GOLGI-Präparaten; nur können bei dieser Methode deshalb keine so reichen Verästelungen und im allgemeinen auch keine so langen Fortsätze zutage treten, weil man hier viel dünnere Schnitte benutzen muß. Wenn daher auch diese Bilder nicht mit Bestimmtheit ausschließen lassen, daß die Fortsätze der Gliazellen dort, wo sie uns unsichtbar werden, in ein Netzwerk einer etwas veränderten Gliasubstanz eintreten, so beweisen sie jedenfalls auch wieder, daß in dem Retikulum verwickelt gebaute Zellen liegen. Auch hier an diesen rein protoplasmatischen Gebilden sehen wir die schon oben beschriebenen vielfachen Beziehungen zu den Gefäßen.

Bei der gleichen Darstellungsweise finden wir nun noch wesentlich anders aussehende Gliaelemente (Tafel XXVIII, Fig. 1*e*, *f*, *g*). EISATH hat sie schon beschrieben und abgebildet. Man kann sie auch in Alkoholschnitten nach NISSL-Färbung erkennen. Um einen Gliakern finden wir ein Häufchen körniger Substanz, oft nur auf der einen Seite des Kernes, während in einem größeren Bogen, durch einen leeren Raum getrennt, eine runde oder ovale Linie, gewöhnlich aus Körnchen, manchmal nur einer Körnerreihe gebildet, den Kern umzieht. Nach innen zu liegen dieser öfters unregelmäßig begrenzte, kleine körnige Fetzchen an, die zuweilen durch eine Brücke mit dem um den Kern gelagerten körnigen Plasma zusammenhängen. Nach außen scheint die Zelle von einer zarten Membran begrenzt. Hin und wieder glaubt man, feine Fädchen von dieser nach außen ziehen und sich in der Umgebung verlieren zu sehen. Diese Fädchen sind aber zu subtil, als daß nicht auch darüber eine Täuschung möglich wäre.

Man kann wohl kaum annehmen, daß diese Elemente in lebensfrischem Zustande diese großen Lücken zwischen Kern und peripherem Plasma aufweisen. Die Bilder haben entschieden Ähnlichkeit mit eigenartigen Ganglienzellenbildern, welche man besonders in den oberen Schichten der Hirnrinde nach Alkoholfixierung und NISSL-Färbung erhält, und deren Zustandekommen NISSL wohl richtig damit erklärt hat, daß durch die Schrumpfung des Gewebes eine Zer-

reißung der Zellen im Protoplasmaleib herbeigeführt worden ist. An diesen Gliazellen gewahrt man nun jedenfalls nichts von einem so weit verzweigten Zelleib, wie bei den zuerst beschriebenen. Vielleicht aber sind die zarten Fortsätze, die man manchmal von dem Rande der Zellen in die Umgebung verlaufen zu sehen glaubt, Verbindungsstücke mit dem im übrigen nicht gefärbten Retikulum, und diese Kerne die eigentlichen Mutterkerne des Syncytiums. Möglicherweise bewirken gerade die vielfachen Verknüpfungen mit dem umliegenden Gewebe die beschriebenen Zerreißungen. Bei der BETHEschen Methode sehen wir ebenso die Gliakerne oft ganz frei im Retikulum liegen.

Einstweilen bleiben das aber nur Vermutungen, für die sich ein sicherer Beweis nicht führen läßt. Feststehen dürfte, daß wir in der normalen Hirnrinde zwei Formengruppen von Gliazellen auseinanderhalten können, solche mit einem weit verästelten protoplasmatischen Zelleib, die auch Fasern bilden können, und solche, die sich mit unseren Fixierungs- und Färbemethoden offenbar mangelhaft darstellen lassen, und an denen wir nur nur einen kleinen rundlichen Zellkörper von mehr körniger Beschaffenheit, oft durch eine Schrumpflücke vom Kern abgerissen, erkennen können. Die Beziehungen beider Formen zu einander und zum Gliaretikulum, sowie die biologische Bedeutung beider erscheint heute noch nicht genügend geklärt.

Mögen aber jetzt alle Gliazellen einem Retikulum angehören oder gewisse davon Zellindividuen geblieben sein, bei stärkeren Zerfallserscheinungen im Nervengewebe zeigen sich viele Gliaelemente durchaus selbstständig, nehmen eigenartige Formen an, assimilieren Zerfallstoffe und zerfallen selbst wieder, ohne Gliafasern gebildet zu haben. Wenn sie normalerweise einem syncytialen Verband angehört hatten, müssen sie sich also aus diesem gelöst haben.

Von großer Wichtigkeit für das Verständnis der Abbauvorgänge im Nervengewebe und für die Aufgabe, welche der Glia dabei zufällt, ist dann die Frage der gliösen Grenzhäute, die aufs engste zusammenhängt mit der der Lymphräume im zentralen Nervengewebe. Hat HELD doch die Grenzmembranen als Filterflächen für die Saftbewegung zu und von dem Nervenparenchym bezeichnet, und haben wir doch schon zu Eingang unserer Betrachtungen uns die Aufgabe gestellt, nachzuweisen, auf welchem Wege im pathologischen Nervengewebe die massenhaften Anhäufungen lipoider Substanzen, die wir in den Zellen der Adventitia finden, und von denen wir annehmen müssen, daß sie aus dem Nervengewebe stammen, an ihre Ablagerungsstätte gelangen, wie sie also diese Filterflächen passieren.

Nun ist die Frage der gliösen Grenzhäute noch keineswegs nach allen Richtungen hin völlig geklärt. Hinsichtlich der Membrana superficialis möchte ich mich auf Grund eigener Präparate ganz den Ausführungen HELDS anschließen. Auch an den gröberen Gefäßen des Zentralnervensystems selbst zeigt sich eine Grenzmembram manchmal so scharf und deutlich, daß man an ihrer Existenz nicht zweifeln kann. Wenn sie nun aber an irgend einer Stelle des Gefäßrohres unverkennbar hervortritt, muß es schon wahrscheinlich werden, daß sie überall vorhanden ist.

Viel schwieriger ist die Frage zu beantworten, ob normalerweise zwischen der Membrana gliae und der Adventitia ein wirklicher Lymphraum sich findet. NISSL hat ihn geleugnet und den perivaskulären Raum für einen Schrumpfraum erklärt. Er hält die Adventitia für die Grenzscheide zwischen mesodermalem und ektodermalem Gewebe, HELD die Gliamembran, er hat mit HIS früher einen perivaskulären Lymphraum zwischen Grenzmembran und Adventitia angenommen. Auch SCHRÖDER beschreibt einen äußeren und inneren Lymphraum und gibt an, daß beide an den kleineren Gefäßen durch weite Spalten in Verbindung stehen. Neuerdings scheint HELD, wenn ich ihn recht verstehe, eine festere Verbindung zwischen Gliagrenzhaut und Adventitia anzunehmen.

Wir sehen tatsächlich in vielen Präparaten so enge Beziehungen zwischen Glia und Adventitia, daß es schwer ist, diese mit künstlichen Verklebungen zu erklären. Mit der Säurefuchsinlichtgrünmethode z. B. gelingt es, die Gliafasern rot und das Bindegewebe grün darzustellen. An Präparaten von paralytischen Rinden kann man nun gelegentlich sehen, wie die Gliafasern so zwischen die Bindegewebsfasern hineingewachsen sind, daß unmöglich ein Lymphraum zwischen beiden gelegen sein kann. Weiter sieht man sehr häufig bei ganz verschiedenartigen pathologischen Vorgängen, wie junge plasmareiche Gliazellen sich so eng um die Kapillaren herumlegen, daß sie ganz mit der Adventitia verlötet scheinen. Dabei ist etwas wie eine Grenzmembran nicht wahrzunehmen.

Man kann nun gegen solche Beobachtungen einwenden, daß sie besondere pathologische Fälle darstellen. Jedenfalls aber machen

sie eine noch weitere Prüfung der Frage nach einem normalerweise vorhandenen perivaskulären Lymphraum nötig. Denn selbst wenn man eine Membrana limitans perivascularis als erwiesen ansieht, ist die Möglichkeit gegeben, daß sie normalerweise an die Adventitia angelegt ist, und daß zwischen beiden kein wirklicher Raum bleibt. Dann würden also Adventitia und Membrana perivaskularis zusammen die Grenzmembran darstellen.

Jedenfalls pflichte ich der Meinung Nissls bei, daß die mesodermalen Infiltrationszellen die Adventitia nur unter ganz besonderen Umständen zu überschreiten vermögen. Denn bei der Paralyse, wo der adventitielle Lymphraum oft völlig von Blutelementen angefüllt ist, sehen wir sie nur selten außerhalb desselben. Das scheint aber auch dagegen zu sprechen, daß ein perivaskulärer Lymphraum durch breite Spalten mit dem Virchow-Robinschen Raum in Verbindung steht, wie Schröder annimmt.

Allgemein ist dann wohl die Meinung verbreitet, daß andere perivaskuläre Gewebslücken, die gegen das Gefäß zu von Resten der Membrana gliae, nach außen zu vom Nervengewebe begrenzt sind, Schrumpfräume darstellen. Man sieht häufig auf das Gefäß zuziehende Gliafasern durch diesen leeren Raum hindurchlaufen und sich an die Adventitia oder dort haften gebliebene Reste der Membrana perivascularis anlegen, oder auch manchmal in füßchenartigen Verbreiterungen in ein auf kürzere oder längere Strecke sich hinziehendes Häutchen verlieren, das frei in der Gewebslücke zu liegen scheint. Das Häutchen ist auch wieder als eine Membrana limitans zu deuten, die um das Gefäß wie eine Manschette herumgelegt war und durch die Gewebsschrumpfung von dem Nervengewebe abgerissen ist, nachdem dies und das Gefäß sich in der Härtungsflüssigkeit in entgegengesetzter Richtung zusammengezogen haben.

Wenn aber nun auch bezüglich der normalen Verhältnisse der Lymphräume und der perivaskulären gliösen Grenzmembran noch mancher Punkt weiterer Klärung bedarf, wenn es auch heute noch nicht sicher bestritten werden kann und nur zweifelhaft scheinen muß, daß es normalerweise perivaskuläre Lymphräume gibt, wir werden den Nachweis bringen können, daß unter pathologischen Bedingungen perivaskuläre Räume zu finden sind, die sich nicht als Schrumpfräume deuten lassen, daß die Membrana perivascularis eine Auflösung erfahren kann, und daß in diesen perivaskulären Räumen ein wesentlicher Teil der Abbauvorgänge im Nervengewebe sich abspielt. Wie die Gefäßgrenzmembran, so kann auch die Oberflächenmembran unter krankhaften Umständen einer Auflösung verfallen. Diese perivaskulären Räume aber gehören zum Nervengewebe, denn wir finden schließlich Gliazellen in ihnen und keine mesodermalen Elemente. Das dürfte auch dagegen sprechen, daß sie präexistierende Lymphräume darstellen.

4. Die pathologischen Veränderungen der Neuroglia bei schweren akuten Erkrankungszuständen des Zentralnervensystems.

A. Die amöboide Gliazelle und ihre verschiedenen Formen.

Schon in meiner Arbeit „Histologische Studien zur Differentialdiagnose der progressiven Paralyse" habe ich Gliazellen beschrieben und abgebildet (Tafel VIII, Fig. 8, 14, 16, 17, 18), welche stark vergrößerte protoplasmatische Zelleiber zeigen und zerfallen, ohne Gliafasern hervorgebracht zu haben. Ich habe diesen Zellen später die besondere Bezeichnung „amöboide Gliazellen" gegeben, weil sie von den übrigen bekannten Gliaformen morphologisch in wesentlichen Richtungen abweichen und in ihrer ausgezeichnetsten Gestaltung große Ähnlichkeit mit einer Amöbe aufweisen. Eisath hat dann dieselben Zellen in großen Mengen in akuten Zuständen der Dementia praecox gefunden. Untersuchungen an vielerlei Material haben schließlich ergeben, daß sie als eine sehr verbreitete Begleiterscheinung akuter Erkrankungszustände des Nervengewebes auftreten. Gewiß sind die amöboiden Gliazellen auch schon von anderen gesehen worden. Ich brauche nur auf die verschiedenen Darstellungen der neurophagischen Vorgänge zu verweisen. Aber die besonderen Umstände ihres Vorkommens, ihr abweichendes morphologisches Verhalten, ihre offenbar eigenartige biologische Bedeutung, die sie ganz wesentlich von anderen Gliaformen, besonders den faserbildenden abrückt, und die große Rolle, welche sie bei den akuten Erkrankungen des Nervengewebes, besonders auch bei den akuten Seelenstörungen spielen, deren anatomische Grundlage sie uns besser verständlich machen, ist jedenfalls bis jetzt nicht hinreichend dargestellt. Zum guten Teil mag das daran liegen, daß uns die für das Studium

der Glia bisher gebräuchlichsten Methoden sehr wenig von ihnen erkennen lassen; die Weigertsche Gliamethode deshalb, weil sie eben nur die Fasern darstellt, die Nisslsche Methode, mit der wir die gröberen protoplasmatischen Gliastrukturen im übrigen gut studieren können, weil sie verwunderlich wenig von den Zelleibern der eigentlichen amöboiden Gliazellen deutlich färbt.

Neben den ungemein typischen Formen, die wir besonders im Mark antreffen, gibt es dort, hauptsächlich aber in der grauen Substanz, außerordentlich mannigfache, zum Teil sehr abweichend gestaltete. Mit der Form einer Amöbe zeigen sie oft keine Ähnlichkeit mehr. Eine reiche Zahl von Zwischenformen aber, die sich auffinden lassen, sowie das Auftreten bestimmter Körnchen, die auch diesen abweichenden Formen mit den typischen amöboiden Zellen gemeinsam sind, spricht für ihre Zusammengehörigkeit und zeigt uns, daß diese weniger durch die morphologischen, als durch biologische Besonderheiten bestimmt wird.

Alle Beobachtungen deuten darauf hin, daß die amöboiden Gliazellen kurzlebige Gebilde sind. Nur unter selteneren Umständen scheinen sich etwas abweichende Dauerformen aus ihnen zu entwickeln. Von diesen abgesehen können wir wegen des rasch ablaufenden Lebenszyklus dieser Gliazellen meist in dem gleichen Schnitt alle ihre Entwicklungsstadien auffinden.

Nun scheint zunächst mit dem Auftreten der amöboiden Gliazellen ein mehr oder minder umfangreicher Untergang alter Giaelemente Hand in Hand zu gehen. Bevor wir auf die Betrachtung der eigentlichen amöboiden Zellen eingehen, erfordert diese Tatsache einige Aufmerksamkeit.

Tafel XXXIII, Fig. 1, 2 und 3 stellt solche regressiv veränderte Gliazellen dar, die aus Schnitten stammen, in welchen gleichzeitig eine üppige Produktion von meist noch sehr jungen amöboiden Zellen zu beobachten ist. Die Zellen Fig. 1 sind aus der weißen Substanz des Rückenmarks eines Falles von tuberkulöser Meningitis, Fig. 2 und 3 aus Hirnrinde und Markleiste von verschiedenen Fällen septischer Delirien gezeichnet.

In Fig. 1 sehen wir mannigfache Veränderungen des Kerns dargestellt, die als regressive gedeutet werden müssen, Homogenisierung des Kerns und Plasmas (*a, b, c*), klumpige Zusammenballungen des Kernchromatins (*e, g, i, k*), Zerfall des Kerns in einzelne Kugeln und Klumpen (*f, h, n, o, r*), auffallend blasse Färbung des Kerns (*p, g*). Man sieht schließlich in solchen Präparaten Kerne und Kernprodukte, die kaum mehr eine Färbung angenommen haben.

In der Fig. 2 sehen wir etwas abweichende Kernveränderungen dargestellt. Teile des Kernchromatins treten aus dem Kern heraus und zerstreuen sich schließlich nach völligem Verlust der Kernmembran, wobei sie mehr und mehr ihre Färbbarkeit einbüßen. In wieder anderen Formen sehen wir den Kerninhalt sich zusammenballen und von der sehr deutlich hervortretenden Kernmembran sich abheben, wie wir das ähnlich an den Kernen der schweren Ganglienzellenerkrankung Nissls beobachten können (*m*). Schließlich färben sich einzelne, weiterhin alle aus dem Chromatin hervorgegangene Kugeln metachromatisch, um schließlich ganz ihre Färbbarkeit zu verlieren (*m, n, p, q*). Diese Veränderungen des Kerns erinnern außerordentlich an die Erkrankung der Ganglienzellkerne im Ammonshorn des wutkranken Kaninchens, die Achúcarro im 1. Hefte des 3. Bandes dieser Arbeiten beschrieben hat.

Die Fig. 3 stellt (in *a—g*) eigenartige schollige und körnige, zum Teil metachromatisch gefärbte Stoffe dar, denen wir oft im Plasma solcher untergehenden Zellen begegnen. *n* und *o* dieser Figur zeigen die Maulbeerform untergehender Gliakerne, auf die wir bei Besprechung der Kernveränderungen der amöboiden Zellen zurückkommen müssen. Sie findet sich auch an diesen zerfallenden Gliazellen, die niemals amöboide Zellen waren. Die um den Kern herum liegenden nichtmetachromatischen Körnchen dürften als abgesprengte Teile des Kernchromatins aufzufassen sein.

Neben diesen Erscheinungen der Pyknose, Karyorhexis und Karyolyse, welche bald sehr verbreitet, bald nur vereinzelt bemerkbar werden, finden wir außer jungen amöboiden Zellen oft mehr oder minder reichliche Karyokinesen in den Gliazellen, die auf der Höhe der Ausbildung der amöboiden Glia dann meist nur sehr spärlich oder gar nicht mehr nachzuweisen sind. In Gehirnen von im Status epilepticus Verstorbenen z. B. sieht man einmal zerfallende Kerne und viele Karyokinesen und nur wenige, selbst gar keine amöboiden Gliazellen, das andere Mal zahlreiche Gliakernteilungen und reichliche junge amöboide Zellen, wieder ein anderes Mal sehr viele und große amöboide Gliazellen und keine einzige Kernteilung.

Die amöboiden Gliazellen des Marks und der Rinde unterscheiden sich, wie schon erwähnt wurde, in nicht unwesentlichen Punkten. Wir wollen mit der Beschreibung der amöboiden Zellen

des Marks beginnen, weil diese viel übereinstimmendere, massigere Formen haben und bei den übersichtlicheren Verhältnissen des Marks leichter zu studieren sind. Man sieht zunächst um den Kern einen wohl abgegrenzten, anfangs nur kleinen Zelleib sich bilden. Er färbt sich kaum bei Anwendung basischer Anilinfarben, bei Benutzung der Methode IV, V, VI aber besonders intensiv. Der Kern ist klein, reich an Chromatin. Mit zunehmender Vergrößerung des Zelleibes nimmt er selbst nicht wesentlich an Größe mehr zu. So bleibt die Kleinheit des Kernes ein wesentliches Merkmal der amöboiden Gliazelle. Mit der weiteren Vergrößerung des Zelleibes treten dann lappige Fortsätze aus ihm hervor. An jüngeren kleineren Zellen zeigen diese noch vielfach konvexe Begrenzungslinien (Tafel XXX, Fig. *g—k*), an älteren, größeren weisen sie dagegen mehr oder minder zahlreiche konkave Ausschnitte auf (Tafel XXX, Fig. *b, c*). In diesen finden sich Markscheiden eingepaßt (Tafel XXVIII, Fig. 6, 7). Die Zelle umfließt also allmählich die benachbarten Markscheiden, schließlich so, daß sie auf dem Querschnitt völlig umschlossen, auf dem Längsschnitt wie von einem Mantel eingehüllt sind. Mit der zunehmenden Vergrößerung werden sie immer komplizierter gestaltete Gebilde. Sie treten nicht nur zu einer, sondern zu zahlreichen Markscheiden in nachbarliche Beziehungen. In dünnen Serienschnitten sieht man einzelne Zellen in zahlreichen aufeinanderfolgenden Schnitten und in immer anderen Umgrenzungslinien und mit immer anders gestalteten Fortsätzen. In dickeren Schnitten zeigt jede kleine Bewegung der Mikrometerschraube ganz andere Formen, weil sie eben nach allen Richtungen des Raumes lappenartige Fortsätze ausstrecken, die sich in sehr verschiedener Weise durch die nervösen Strukturen hindurchwinden. Auf Zeichnungen oder durch Photographien die ganze Kompliziertheit des Baues großer amöboider Gliazellen wiederzugeben, ist daher ganz unmöglich. In den allermeisten Fällen findet man sie um die Gefäße herum besonders zahlreich. Sie liegen oft in mehreren Schichten nebeneinander, Jugendformen zwischen älteren, und bilden förmliche Mäntel um die Gefäße. Öfters sehen wir auch, wie eine Zelle einen breiten Fortsatz zu einer Kapillare schickt und diese wie mit einer Manschette umgreift (Tafel XXX, Fig. 7). Ganz gewöhnlich aber verbreitern sich die an die Gefäße herantretenden Fortsätze der amöboiden Zellen an der Gefäßwand sehr beträchtlich.

An den jungen Elementen erscheint der Zelleib zunächst ganz homogen, ohne jede Körnung, ganz gleichmäßig und satt gefärbt.

Im weiteren Lebenslauf machen sich dann allerlei Veränderungen in ihm bemerkbar. Um sie zu verfolgen, muß man sich verschiedener Methoden bedienen. In Präparaten, die mit Malloryschem Hämatoxylin gefärbt sind, werden zunächst in den großen Zellen rundliche Vakuolen sichtbar (Tafel XXVIII, Fig. 6 u. 7). Sie sind erst klein, können aber schließlich mehrfache Kerngröße erreichen. Bei genauerem Zusehen bemerkt man, daß ihr Inhalt leicht gelblich erscheint. In der Mannschen Lösung nehmen die Vakuolen gegenüber dem blaugefärbten Plasma vielfach einen zart rötlichen Farbton an. Mit zunehmender Größe der Zelle vermehrt sich die Zahl der Vakuolen, bis schließlich der ganze Zelleib davon erfüllt ist, eine neben der anderen liegt und nur schmale Plasmabrücken dazwischen geblieben sind. Besser noch lassen sich diese Vakuolen verfolgen an Präparaten, die nach Fixierung in Flemmingscher Lösung mit Säurefuchsinlichtgrün gefärbt sind. Man sieht in dem gleichmäßig hellgrün gefärbten Zelleib leuchtend rot gefärbte, daneben zuweilen auch dunkelgrüne Granula auftreten (fuchsinophile und Lichtgrüngranula [Tafel XXX, Fig. 3*g, h, i, k*]). Die Lichtgrüngranula scheinen vereinzelter vorzukommen und in vielen Zellen ganz zu fehlen. Nur selten begegnet man ihnen in größerer Menge. Auch die fuchsinophilen Granula sind anfangs einzeln, werden aber schließlich immer zahlreicher und liegen dann im ganzen Zelleib zerstreut, meistens an einzelnen Stellen angehäuft. Sie sind stets rund, meist annähernd gleich groß. Ihre beträchtliche Größe unterscheidet sie von den sonst im Gewebe auftretenden fuchsinophilen Granula. Die Körnchen der Neurosomenhaufen sind kleiner und nahe zusammengedrängt, die normalerweise in den Ganglienzellen vorkommenden, gleichfalls von geringerer Größe und teilweise nicht rund. Nur in pathologischen Ganglienzellen treffen wir ebenso große fuchsinophile Granula.

Offenbar erst in einem späteren Lebensstadium treten dann auch mit Osmium gebräunte Stoffe auf. Diese fettigen Substanzen sind nur ganz selten in der Form runder Granula zu finden, sie zeigen sich als größere Klümpchen von anscheinend solider Beschaffenheit und meist etwas unregelmäßigerer Form oder als außerordentlich zierliche Bildungen, die das doppelte und dreifache der Kerngröße erreichen können (Tafel XXX, Fig. 3*a—e*). Es ist wohl nicht zu bezweifeln, daß diese letzten Bildungen den Vakuolen entsprechen, welche wir in den mit Malloryschem Hämatoxylin oder der Mannschen Färbung dargestellten Zellen beobachtet haben.

Es handelt sich also hier nicht um Vakuolen, sondern um lipoide Cystchen. Sie erscheinen nach dieser Behandlung meist kreisrund oder oval und aus vielen kleineren Cystchen zusammengesetzt, die gleichfalls eine rundliche Form haben. Bei Anwendung starker Systeme bemerkt man, daß ihre Wandungen von außerordentlich feinen gebräunten Körnchen gebildet werden. Manchmal sieht man eine große zentrale Cyste und um diese herumgestellt zahlreiche kleine, die dann zusammen eine große Kugel abgeben. Der Inhalt erscheint ganz leicht gelbbraun gefärbt. Diese Cystchen finden sich auch in Gliazellen, die Fasern gebildet haben und scheinen schließlich fast das einzige zu sein, was von dem Zelleib der amöboiden Zelle übriggeblieben ist (Tafel XXX, Fig. 3*a*).

Man wird wohl kaum ein Recht für die Annahme haben, daß diese lipoiden Cystchen unserer S.-Fuchsin-Lichtgrünpräparate schon im Leben in der Form vorhanden sind, in denen wir ihnen hier begegnen. Dagegen dürfte schon die Beobachtung sprechen, daß wir im Formolgefrierschnitt nach HERXHEIMER-Färbung dem Anschein nach solide rotgefärbte Kugeln um die Gliakerne finden, welche amöboiden Zellen zugehören dürften. Die Erkennung der amöboiden Gliazellen ist in diesen Präparaten allerdings nicht ganz leicht. Wenn wir daher auch annehmen müssen, daß die Form dieser Cystchen durch unsere Fixierungs- und Einbettungsmittel bestimmt worden ist, so muß doch hervorgehoben werden, daß wir die ja oft so reichlich in den Ganglienzellen, den Zellen der Gefäßwand und den gliogenen und mesodermalen Körnchenzellen angehäuften lipoiden Stoffe auch bei gleicher Vorbehandlung gewöhnlich nicht in dieser Form sehen und daß auch die fettigen Produkte, die sich in den großen faserbildenden Gliazellen eingelagert finden, uns meistens in der Gestalt solider Körnchen und Tropfen begegnen. Nur bei der noch später zu besprechenden grobkörnigen, fettigen Degeneration der Ganglienzellen treffen wir die lipoiden Stoffe in Form ganz ähnlicher kleinster Cystchen. Das dürfte wohl darauf hinweisen, daß wir vielleicht doch hier einen besondersartigen lipoiden Stoff vor uns haben. Dafür spricht auch, daß sich in der MANNschen Farbmischung der Inhalt dieser Fettröpfchen oft deutlich rötlich färbt, während die lipoiden Körnchen der Ganglienzellen und Gefäßwandzellen eine schmutziggrünliche Farbe annehmen.

Während sich nun mehr und mehr solcher Cystchen in dem Zellleib der großen amöboiden Zellen des Markes ansammeln, wird das Plasma immer spärlicher und dabei mangelhafter gefärbt. Schließlich gewinnt man den Eindruck, daß die Cystchen zerfließen und die ganze Zelle zerfällt. während auch der Zellkern regressive Veränderungen eingeht.

Neben dieser Form regressiver Veränderung der amöboiden Gliazellen sehen wir dann recht häufig noch eine zweite abweichende Art, die noch rascher zu einem Zerfall derselben zu führen scheint. Der Zelleib aller jungen amöboiden Zellen ist, wie schon erwähnt, homogen und satt gefärbt. Bei den mit MALLORYschem Hämatoxylin oder MANNscher Farblösung gefärbten Schnitten tritt aber häufig in den amöboiden Gliazellen eine Körnelung auf. Das im allgemeinen matter gefärbte Plasma zeigt sich wie bestäubt mit dunkelviolett bzw. dunkelblau gefärbten Körnchen (Tafel XXVIII, Fig. 8*c*, 9 und 10, Tafel XXIX, Fig. 17). Ich will sie der Kürze wegen weiterhin Methylblaugranula nennen, obwohl sie bei Anwendung des MALLORYschen Hämatoxylins noch schärfer hervortreten. Diese Methylblaugranula sind manchmal außerordentlich fein, in anderen Zellen wieder erheblich gröber, in den einzelnen Zellen aber gewöhnlich von sehr übereinstimmender Größe. Mit der Bildung dieser Körnchen geht offenbar eine Lockerung im Gefüge des Zellplasmas vor sich. Bei der Säurefuchsinlichtgrünfärbung, bei welcher die Methylblaugranula gar nicht dargestellt werden, färbt sich der Zelleib kaum mehr mit Lichtgrün und hat ein wabiges oder körniges Aussehen angenommen. Gleichzeitig lassen schwere Veränderungen am Zellkern erkennen, daß die Zellen zugrunde gehen. Die Kerne färben sich ganz homogen oder auffallend blaß, und man sieht, wie sich der Inhalt des Kerns zusammenballt und von der Kernmembran zurückzieht (Tafel XXVIII, Fig. 8*c* und 10). Meist gleichzeitig verändern sich dann in der Markleiste auch Gliazellen, welche nicht eigentlich den Charakter amöboider Gliazellen angenommen haben, sondern längere, schmale Fortsätze und kaum einen deutlichen Zelleib besitzen. Es hat den Anschein, als ob sich die Fortsätze in Körner auflösten, die sich ebenso wie die Methylblaugranula echter amöboider Gliazellen sehr intensiv mit Methylblau und noch intensiver mit MALLORYschem Hämatoxylin färben (Tafel XXVIII, Fig. 11, 12, 13).

Auch hier beobachten wir wieder, daß gerade um die Gefäße herum die größte Zahl von Zellen gelegen ist, welche solche Methylblaugranula führen. Aber im ganzen Mark können schließlich solche zerfallende Zellen zerstreut liegen. Ein Teil davon scheint seine

Färbbarkeit schon im Gewebe einzubüßen. Jedenfalls geht eine große Zahl amöboider Gliazellen unter Bildung dieser Methylblaugranula zugrunde.

Ungleich vielgestaltiger, verwickelter und deswegen auch viel schwerer einem klaren Verständnisse zugänglich sind die Gliaveränderungen in der grauen Substanz, besonders in der Hirnrinde, welche mit dem Auftreten amöboider Gliazellen im Marke einhergehen. Nur in ganz seltenen Fällen begegnen wir auch in der Rinde amöboiden Zellen, die denen im Mark in jeder Weise entsprechen. Von diffusen Erkrankungen der Hirnrinde habe ich das nur bei außerordentlich stürmisch verlaufenen und nach gehäuften Anfällen verstorbenen Paralysen und ähnlich, wenn auch etwas abweichend, in einem Falle von progressiver Chorea gesehen. Tafel XXVIII, Fig. 8*a*, *b* stellt amöboide Gliazellen aus der Hirnrinde einer foudroyant verlaufenen Paralyse dar. Diese Zellen haben dann häufig, während sie sonst in ihrem Verhalten von denen des Marks nicht abweichen, viel längere, schmälere und zartere Fortsätze als jene. Während die protoplasmatischen Fortsätze gewöhnlicher Gliazellen mit zunehmender Entfernung vom eigentlichen Zelleibe sich mehr und mehr zu verjüngen pflegen, enden hier die Fortsätze häufig mit keulenförmigen Verdickungen. Infolge der weiten Ausbreitung und des großen Reichtums an längeren Fortsätzen sieht man nicht selten in der Nachbarschaft der Zelle durch die Schnittführung abgetrennte Fortsatzstücke liegen, die nach ihrer Form und Verlaufsrichtung offenbar zur gleichen Zelle gehören.

Eine noch stärkere Abweichung nach der gleichen Richtung stellen dann offenbar die Zellen dar, welche auf Tafel XXIX, Fig. 3, 15 und 16 abgebildet sind und von einem Falle von progressiver Chorea stammen. Die Fortsätze, welche im übrigen ganz den Charakter der Fortsätze amöboider Zellen zeigen, sind hier noch länger und noch dünner, während ein eigentlicher Zelleib kaum gebildet ist. Sie ziehen oft lang an den Fortsätzen der Ganglienzellen hin, wie z. B. in Tafel XXIX, Fig. 16, wo die Gliazellen nach oben und nach unten einen Protoplasmafortsatz abgibt, der sich an den Hauptdendriten einer Ganglienzelle anschmiegt. An diesen Zellen beobachten wir sehr merkwürdige regressive Veränderungen, auf die wir später noch zurückkommen müssen. Sie sind auf Tafel XXIX, in Fig. 4, 5, 8 und 15 veranschaulicht. Der lange Fortsatz nimmt häufig eine perlschnurartige Form an und zerfällt später in die

einzelnen Glieder, während sich gleichzeitig der Zelleib in einzelne Schollen auflöst und der Kern die gleichen regressiven Veränderungen zeigt, wie sie schon oben von den amöboiden Zellen des Markes beschrieben worden sind. So begegnen wir schließlich Ganglienzellen, die selbst degenerative Erscheinungen aufweisen, umgeben von heller und dunkler gefärbten Schollen, welche aus Fortsätzen solcher amöboider Gliazellen hervorgegangen sind (Tafel XXIX, Fig. 8), und finden auch sonst im Gewebe, besonders aber um die Gefäße herum, Anhäufungen der gleichen Zerfallsprodukte.

Einen viel verbreiteteren Typus amöboider Gliazellen als der eben beschriebene stellt eine Form dar, die in ihrer Gestalt noch wesentlicher von den Zellformen der weißen Substanz abweicht, ihr aber doch in ihrem ganzen Entwicklungsgange sehr ähnelt. Wir sehen solche Formen zunächst auf Tafel XXVIII, Fig. 2 und 3 abgebildet, wo sie Ganglienzellen umlagern oder einer Kapillare anliegen. Der Zelleib ist hier völlig rund oder auch oval geworden, und an den kleineren jüngeren Elementen gleichmäßig und intensiv gefärbt, wie der Zelleib junger amöboider Gliazellen des Markes. Der Kern liegt bei den jugendlicheren Formen oft in der Mitte des Zelleibes, rückt aber bei den älteren häufig aus seiner zentralen Lage.

Da diese Zellform in der Rinde verhältnismäßig am häufigsten vorkommt, müssen wir sie etwas eingehender betrachten. Man kann sie schon leidlich gut darstellen an Alkoholschnitten, die mit Toluidinblau gefärbt sind, wenn die Differenzierung nicht zu weit getrieben worden ist, ebenso mit der Methylgrünpyroninfärbung, und noch besser mit einer Giemsa-Lösung, wenn man 12 Stunden färbt und mit Anilinölalkohol nur mäßig entfärbt. Besonders bei der letzten Färbung nimmt das Plasma dieser Zellen einen ganz eigenartigen blaßblauen Farbton an, so daß sie sich sehr schön aus dem Gewebe hervorheben. Schon mit diesen Färbungen lassen sich sehr bald regressive Veränderungen am Plasma und Kern dieser Zellen nachweisen. Im Plasma treten unregelmäßige Körnchen und Krümel von etwa dunklerer Färbung, oft mit einem gelblichen Mischton hervor (Tafel XXXIII, Fig. 6, 8, 9). Im Kern werden helle Flecken bemerkbar (Fig. 6 unten), er nimmt die schon erwähnte Brombeerform an, indem die Kernmembran verschwindet und grobe Chromatinschollen etwas über den Rand des Kerns heraustreten (eine Zelle in Fig. 11, die unterste Zelle in Fig. 9). Weiterhin rücken dann einzelne Chromatinteile ganz aus dem Kern heraus (Fig. 8 und 9),

und schließlich löst sich der ganze Kern in einzelne Schollen auf, die frei im Zellplasma zerstreut liegen (Fig. 9 die mittlere Zelle).

Besonders bemerkenswert sind aber nun die Beziehungen dieser amöboiden Gliazellen zu den Ganglienzellen und zu den Gefäßen. Man sieht, daß sie sich in großer Zahl um die Ganglienzellen herum anhäufen (Tafel XXVIII, Fig. 2*a*, *c*), wobei sie sich vielfach in die Ganglienzellen hineindrücken, so daß sie in Ausbuchtungen des Ganglienzelleibes zu liegen kommen, ja den Zellkern zusammenpressen, so daß er eine Biskuitform oder eine Nierenform annimmt (Tafel XXVIII, Fig. 2*a*, Tafel XXXIII, Fig. 8, 9, 10).

Nicht nur der eigentliche Zelleib, auch die Protoplasmafortsätze werden dadurch verändert. Welch merkwürdige Umgestaltungen der Zellen so herbeigeführt werden können, zeigen am besten die Figuren 8, 9 und 10 der Tafel XXXIII, und die Figuren 6, 10, 11, 12 und 13 der Tafel XXIX. An solchen Bildern wird es oft schwer, die Grenze des Zelleibes der Glia- und der Ganglienzelle zu bestimmen. Gar nicht so selten (Tafel XXXIII, Fig. 11), sieht man auch wie eine verhältnismäßig kleine amöboide Gliazelle in einer viel größeren Nische oder Aushöhlung einer Ganglienzelle gelagert ist.

Am schönsten aber dürfte die S.-Fuchsinlichtgrünfärbung das Verhältnis dieser Gliazellen zu den Ganglienzellen darstellen. Tafel XXX, Fig. 4 gibt eine Reihe solcher Bilder wieder, die aus der Hirnrinde stammen, während Tafel XXX, Fig. 2 zwei einzelne Gliazellen aus der grauen Substanz des Rückenmarkes veranschaulicht. Wir sehen auch hier wieder in mehr oder minder reichlichen Mengen fuchsinophile Granula auftreten, daneben zuweilen auch mit Lichtgrün gefärbte in größerer Zahl, und in den älteren Zellen regelmäßig Häufchen mit Osmium gebräunter Substanzen. Wie allmählich die ganze Ganglienzelle durch solche amöboide Gliaelemente ersetzt werden kann, zeigt sehr schön die Abbildung *e* und *f* in Fig. 4. Die Kerne sind auch hier in der schon früher beschriebenen Weise verändert. Häufig begegnen wir in Schnitten, in welchen sehr viele amöboide Zellen in vorgeschrittenen Stadien zu sehen sind, Bildern, wie sie Tafel XXIX, Fig. 7 und 9 wiedergibt. In Lücken des Gewebes, die, wie ihre ganze Form zeigt, durch Zerfall von Ganglienzellen entstanden sind, trifft man Kernreste, die eben noch ihre Abstammung von Ganglienzellkernen erkennen lassen, darum gelagert allerlei Körnchen, welche wohl zum Teil in regressiv veränderten amöboiden Gliazellen liegen, und hin und wieder dazwischen

junge amöboide Gliazellen, die offenbar eben erst ihren Entwicklungsgang anfangen. Wo sich diese Gliazellenformen um die Ganglienzellen herum und sonst im Gewebe finden, liegen sie in der Regel auch um die Gefäße angehäuft (Tafel XXVIII, Fig. 3; Tafel XXXIII, Fig. 14). Wir werden uns noch später mit diesen zu beschäftigen haben.

Im allgemeinen zeigen die eben beschriebenen Zellformen, wenn sie auch von den amöboiden Zellen des Markes in morphologischer Beziehung stark abweichen, doch noch mit ihnen viele Ähnlichkeit. Diese wird aber immer geringer, wenn wir andere pathologische Gliaformen betrachten, die sich auch in der grauen Substanz finden, wo echte amöboide Gliazellen im Mark auftreten. So sehen wir bei manchen infektiösen Delirien, bei experimentellen Intoxikationen Gliaelemente in der Hirnrinde, die um ihren Kern nur einen ganz schmalen, runden Zelleib bilden, noch nicht vom halben Durchmesser des Kerns, oft auch nur nach einer Seite hin, aber scharf abgegrenzt. In diesem kleinen Zelleib treten nun bei Fuchsinlichtgrünfärbung reichlich rote, grüne und bräunliche Granula auf. Man kann hier von Miniaturformen amöboider Zellen reden (Tafel XXX, Fig. 3 *l*, *m*). Aber auch der letzte Rest der Amöbenform ist verloren gegangen bei pathologischen Gliaformen, wie sie uns Tafel XXX, Fig. 1 *a—c* darstellt, die von Fällen von Epilepsie stammen, welche im Status epilepticus gestorben sind. Unter den vielen fuchsinophilen Körnchen, welche sich in der Rinde finden und anderen Strukturen zugehören, den Nervenzellen, den Neurosomenhäufchen, den Achsenzylindern, sieht man um weniges größere, die zu Gliazellen gehören müssen, da sie in oft langen Reihen wie Strahlen gegen einen Gliakern zu gerichtet liegen. Die normalen Gliazellen haben nur ganz feine oder gar keine fuchsinophile Granula. Jedenfalls findet man in einem normalen Gehirn nie diese auffällige Anordnung langer Reihen größerer Granula zu den Gliakernen. Schon aus dieser Reihenbildung läßt sich schließen, daß sie Gliazellen mit verzweigten Fortsätzen, wie sie Tafel XXVIII, Fig. 1 wiedergibt, zugehören. Gliazellen aus der gleichen Rinde mit MALLORYschem Hämatoxylin gefärbt, geben eine Bestätigung dieser Vermutung (Tafel XXVIII, Fig. 1 *h*, *i*). Sie erinnern in ihrer Form an die normalen verzweigten Gliaelemente, doch sind ihre Verzweigungen plumper, etwas massiger geworden. Damit nähern sie sich schon etwas in ihrer Form den Zellen, welche wir von der progressiven Chorea beschrieben haben. Der zarte

Protoplasmaleib dieser Gebilde hat nur bei der S.-Fuchsinlichtgrünfärbung kaum etwas von dem Lichtgrün zurückbehalten. Sehr häufig sehen wir nichts von mit Osmium gebräunten Körnchen in diesen Zellen. Manchmal aber treten auch in ihnen wieder diese Cystchen auf, die wir in den amöboiden Zellen des Markes kennen gelernt haben. Sie bleiben nur sehr erheblich kleiner. Manchmal ist es auch so, daß in den Fortsätzen die fuchsinophilen Granula, um den Kern herum in einem schmalen Protoplasmaleib die gebräunten Cystchen gelegen sind. Ganz die gleichen Elemente bemerken wir dann auch in der Nachbarschaft der Gefäße, vielfach so, daß Reihen roter Granula bis an die Wand der Gefäße heranziehen.

Zwischen diesen Zellformen, die wir besonders schön öfters bei der Epilepsie beobachten können, und den früher beschriebenen amöboiden Gliazellen mit reichlichem Plasma um den Kern herum, ohne feinere protoplasmatische Fortsätze, finden sich noch alle Übergänge, wenn man die verschiedensten Rindenerkrankungen untersucht. Wir sehen Gliazellen mit kurzen Fortsätzen oder auch nur mit einem langen Fortsatz, oder Miniaturformen mit rundlichem Zelleib und nur einem einzigen oder auch einigen längeren oder kürzeren pseudopodienartigen Verzweigungen des Plasmaleibes mit fuchsinophilen Granula und lipoiden Einschlüssen. Gerade bei den akuteren Fällen der Dementia praecox trifft man besonders oft solche Zwischenformen, die auch die innere Zusammengehörigkeit aller dieser verschiedenen Elemente zeigen.

Bei anderen akuten und subakuten Erkrankungen, z. B. dem Alkoholdelirium wieder können wir im S.-Fuchsinlichtgrünpräparat wohl eine reichlichere Anhäufung fuchsinophiler Granula um Gliakerne herum wahrnehmen, auch wohl eine Reihe solcher Körnchen zu einer Ganglienzelle hinziehen oder auf ein Gefäß zustreben sehen, während es bei dem Fehlen eines deutlich gefärbten Zelleibes und den reichlich überall im Gewebe zerstreuten fuchsinophilen Granula unmöglich wird, alle zu der Gliazelle gehörigen Granula zu bestimmen. Damit wären wir bei einer Grenze der Leistungsfähigkeit unserer Methode angelangt, welche bis jetzt trotz aller Mühe nicht beseitigt werden konnte.

Schließlich lassen sich in manchen Gehirnen, in welchen amöboide Gliazellen im Mark gefunden werden, dort und in der Rinde eigenartige Zellen mit der WEIGERTschen Gliamethode darstellen. Tafel XXXI soll ein Bild derselben geben. Sie sind gekennzeichnet durch andersartige Granula, die sich mit dieser Methode blau färben, und die ich fibrinoide nennen will. Der Name soll aber nichts über die chemische Natur dieser Körnchen sagen; die WEIGERTsche Methode färbt ja neben dem Fibrin auch noch mannigfache andersartige Stoffe. Es soll nur ein vorläufiger Name zur Verständigung darüber sein, wovon die Rede ist.

Manche von diesen Zellen zeigen eine weitgehende Ähnlichkeit mit den Zellen, die auf Tafel XXVIII, Fig. 11—13 dargestellt sind, so daß die Vermutung erweckt werden muß, daß vielleicht die Methylblaugranula mit diesen fibrinoiden Granula identisch sind. Vergleicht man mit beiden Methoden von demselben Gehirn gewonnene Präparate, so hat es wirklich den Anschein, als ob manche Zellen Granula enthielten, die sich sowohl mit der WEIGERTschen Gliamethode, als mit MALLORYschem Hämatoxylin oder MANNscher Farbe färben. Aber doch sind die auf verschiedenen Wegen erhaltenen Bilder nicht übereinstimmend. Besonders in der grauen Substanz habe ich niemals mit den anderen Färbungen so massenhafte Granula erhalten wie bei der Gliamethode. So dürfte es sich hier vielleicht ähnlich verhalten wie bei den mit Scharlach und Osmium färbbaren Stoffen, von denen manche gerötet und geschwärzt, andere nur gerötet und andere nur geschwärzt werden. Die meisten auf Tafel XXXI abgebildeten Zellen stammen von der Dementia praecox, der „Angstpsychose“ oder von schweren Infektionsdelirien. Man sieht, wie wir das vorhin bei den fuchsinophilen Granula anderer Gliazellen gesehen haben, von weither Reihen blauer Körnchen auf einen Gliakern zustreben, um den herum meist eine dichtere Anhäufung derselben gelegen ist. Nichts kann uns besser die weite Ausbreitung der Fortsätze einer Gliazelle vor Augen führen als Bilder, wie sie uns die Fig. 1, 2, 3, 6 und 14 darstellen, die alle aus der Hirnrinde stammen. Wenn wir uns zwischen den einzelnen Körnchen Protoplasmabrücken denken, dann würden wir auch hier wieder Bilder bekommen, ähnlich wie sie uns Tafel XXVIII, Fig. 1 darstellt. Da die Zellen, welche durch solche Granula gekennzeichnet sind, meist zerstreut im Gewebe liegen, begegnen wir oft auch Körnchenreihen oder einzelnen Häufchen, zu denen sich kein Kern finden läßt, offenbar nur deshalb, weil er auf einem anderen Schnitt liegt. Denn man gewinnt nicht den Eindruck, daß solche Körnchen frei, ohne Beziehungen zu Zellen vorkommen, wenn man auch meist kein Gliazellplasma nachweisen kann, in dem sie liegen. Bei der angewandten Methode wird eben das Plasma nicht dargestellt. Bemerkenswert

ist, daß auch hier wieder die Körnchen der einzelnen Zelle meist sehr in der Größe übereinstimmen, aber in verschiedenen Zellen in der Größe sehr wechseln, von den feinsten, staubartigen (Fig. 5) bis zu recht groben (Fig. 6). Sehr häufig sieht man besonders an den Zellen der grauen Substanz, daß sich eine Körnerreihe bis an ein Gefäß erstreckt (Fig. 4). Hin und wieder liegen solche Zellen eng an eine Ganglienzelle angeschmiegt, und die Körnerreihen laufen dann über sie hinweg und um sie herum (Fig. 14).

Die entsprechenden Zellen des Markes zeigen meist etwas andere Form (Fig. 8, 9, 11 und 12). Die Körnchen sind hier mehr um den Zellkern angehäuft, die Fortsätze kürzer, und nicht selten läßt sich hier ein schwach gefärbtes Plasma erkennen, in dem sie eingebettet liegen. Das macht es jedenfalls wahrscheinlich, daß auch die fibrinoiden Granula der zarteren Rindenzellen noch in einem Plasma liegen und nicht etwa, woran man denken könnte, einer Auflösung des Plasmas ihre Entstehung verdanken. Gar nicht selten kann man beobachten, daß die um den Kern herum gelegenen Granula sich blasser färben oder gar keine Farbe mehr angenommen haben und nur durch einen leicht gelblichen Ton erkennbar sind. Manchmal erscheinen auch die Körnchen etwas von dem Kern abgerückt, so daß dieser von einem von Körnchen freien Hof umgeben ist (Fig. 7). Jedenfalls haben diese fibrinoiden Granula nichts gemein mit den fuchsinophilen.

Fassen wir nun diese Beobachtungen zusammen, so ergibt sich als das Wesentlichste:

Wir finden bei gewissen akuten und subakuten Erkrankungszuständen des Zentralnervensystems, besonders auch der Hirnrinde, pathologische Gliaelemente, die meist rasch entstehen und vergehen, ohne Gliafasern zu erzeugen. In ihren ausgezeichnetsten Formen, die wir hauptsächlich in der weißen Substanz beobachten, erinnern sie an die Gestalt von Amöben, weshalb man sie als amöboide Zellen bezeichnen kann. Den mehr gleichmäßigeren Bildern dieser Zellen im Mark entsprechen viel verschiedenartigere Formen in der Rinde. Wir begegnen dort Elementen, die denen des Markes sehr ähnlich sind und anderen, die in ihrer Form so von ihnen abweichen, daß der Name der amöboiden Zelle nicht mehr ihrer Gestaltung entspricht. Alle diese Zellen aber haben die gemeinsame Eigenschaft, daß in ihnen bei ihrer Rückbildung verschiedenartige Granula entstehen, als welche wir kennen gelernt haben: die fuchsinophilen, die Lichtgrüngranula, die lipoiden Körnchen und Cystchen, die Methylblaugranula und die fibrinoiden Granula.

B. Über die Veränderungen an den Gefäßen, welche das Auftreten amöboider Gliazellen begleiten.

Mit dem Auftreten amöboider Gliazellen im Nervengewebe sehen wir so regelmäßig auch an den Gefäßen bestimmte Veränderungen sich abspielen, daß man auf Beziehungen zwischen beiden schließen muß. Zunächst finden sich in den mit MALLORYschem Hämatoxylin oder der MANNschen Farbe behandelten Gefrierschnitten aus der Gliabeize, besonders wenn gleichzeitig amöboide Gliazellen im Stadium des Zerfalls reichlich im Nervengewebe zu bemerken sind, mancherlei Produkte in einem perivaskulären Raum abgelagert. Es sind verschieden gestaltete, oft rundliche Ballen oder größere und kleinere, ganz unregelmäßige Häufchen von Körnern und Krümeln, die alle Töne, von der blassesten bis zur intensivsten Färbung, angenommen haben. An den Ballen kann man häufig eine Grundsubstanz, die gleichmäßig und heller gefärbt ist, und in ihr Stäubchen, Körnchen oder Körner von dunklerer Färbung unterscheiden, in der Weise, daß die Körnchen desselben Ballens meist die gleiche Größe aufweisen, in den verschiedenen Ballen aber sehr verschieden groß sind. Manchmal sind alle derartigen Gebilde desselben perivaskulären Raumes von weitgehender Übereinstimmung in Farbe und Größe der Ballen und ihrer Körnchen, manchmal ist jeder Ballen von dem anderen darin abweichend.

Die scharfe Färbung der Adventitia, welche diese Methoden geben, läßt keinen Zweifel darüber, daß diese Ablagerungen perivaskulär liegen. An Präparaten, welche mit der WEIGERTschen Gliafärbung hergestellt sind und die fibrinoiden Granula in schöner Darstellung zeigen, finden wir in perivaskulären Räumen Einlagerungen, die mit den eben beschriebenen viele Ähnlichkeit zeigen. Auch hier sind es wieder Ballen und Haufen von rundlicher oder unregelmäßiger Form, meist mit einer zarteren Grundfärbung und feineren oder gröberen Körnelung, wobei auch wieder die Körnchen desselben Haufens gewöhnlich eine sehr übereinstimmende Größe zeigen.

Gegenüber den eben beschriebenen, nach Fixierung in der Gliabeize und Färbung mit der MANNschen Farblösung, dem MALLORY-

schen Hämatoxylin oder der WEIGERTschen Gliafärbung gewonnenen Präparaten zeigen Schnitte, die von FLEMMING-Material stammen und mit Säurefuchsinlichtgrün gefärbt worden sind, ein wesentlich abweichendes Aussehen. Tafel XXX, Fig. 7, 8, 9 und 10 gibt solche Bilder wieder. Auch hier ist durch die scharfe Färbung der Adventitia die Begrenzung des VIRCHOW-ROBINschen Lymphraumes und auch des perivaskulären Raumes, wenigstens an den größeren Gefäßen, sehr deutlich wahrzunehmen. In letzterem liegen nun nicht die körnigen Haufen, die wir in den vorher besprochenen Präparaten gefunden haben, dagegen zeigt er sich durchzogen von zahlreichen bandartigen Brücken und schmalen Fäden, die sich vielfach an die Adventitia anheften und ebenso auch an den Rand des Nervengewebes mit einer Grenzlinie anlegen. Andere, mehr schollenartige Gebilde liegen völlig frei im perivaskulären Raum. Manchmal zeigen sich diese Brücken von ganz homogener Beschaffenheit, dann auch wieder mehr gerinnselartig gelockert. Fuchsinophilen Granula begegnet man gewöhnlich in ihnen.

Auch an Alkoholschnitten, die mit Toluidinblau oder GIESMSA-Lösung oder Methylgrünpyronin gefärbt worden sind, sieht man in solchen Fällen eigentümliche Bildungen in den perivaskulären Räumen (Tafel XXXIII, Fig. 14). Sie sind schon von CERLETTI früher beschrieben worden. Kahn- und halbmondförmige oder rundliche, aber von der einen Seite her stärker aufgehellte, ganz zart oder dunkel gefärbte Gebilde spannen sich von der Gefäßwand durch den perivaskulären Raum bis ans Nervengewebe. In ihrer Färbung ähneln sie oft so sehr der Färbung des Zelleibes der amöboiden Gliazellen, die dazwischen liegen, daß man auf den Gedanken kommen könnte, es seien Überreste solcher zerfallender Zellen. Aber manche von ihnen zeigen eine so leuchtende Farbe, wie sie das zerfallende Plasma nicht anzunehmen pflegt, und gelegentlich — ich habe das besonders bei schweren septischen Delirien öfters gesehen — ist der ganze perivaskuläre Raum, ohne daß ein Kern dazwischen zu finden ist, gleichmäßig offenbar von derselben Masse ausgefüllt, aus welcher diese Gebilde bestehen.

Was sind das aber nun für Stoffe, und wie gelangen sie in den perivaskulären Raum?

Schon der Umstand, daß sie bei verschiedenen Fixierungsmitteln ein recht verschiedenes Aussehen annehmen, muß den Gedanken nahe legen, daß sie wenigstens z. T. erst bei der Ausfällung durch die angewandten Reagentien in die in den Präparaten sichtbare Form gebracht worden sind. Besonders dort, wo wir den ganzen perivaskulären Raum von einer gleichmäßig homogenen Masse ausgefüllt finden, kann es wohl keinem Zweifel unterliegen, daß wir Gerinnungsprodukte vor uns haben.

Nun sehen wir im normalen Gehirn aber nichts von solchen Bildungen. Hin und wieder zeigen uns dieselben Methoden hier überaus spärliche Körnchen oder Fädchen, die man wohl auch als Gerinnungsprodukte der Lymphflüssigkeit auffassen muß, nie aber begegnen uns dort solche intensiv gefärbte Klumpen und Massen. Es kann sich also nur um pathologische Produkte handeln. Wenn sie aber erst durch die Härtungsflüssigkeiten in feste Form gebracht worden sind, so dürfte die Möglichkeit zugegeben werden müssen, daß sie erst bei der künstlichen Schrumpfung des Gewebes durch die Fixierungsmittel aus dem benachbarten Gewebe in artefizielle perivaskuläre Schrumpfräume gepreßt und dort erst ausgefällt worden sind.

Die gleiche Erklärung läßt sich aber kaum anwenden auf einen anderen Teil des Inhalts dieser perivaskulären Räume. Betrachten wir Tafel XXX, Fig. 8, so könnte man wohl noch annehmen, daß bei der Schrumpfung des Gewebes das sich zusammenziehende Gefäß die mit ihr verwachsene Gliazelle aus dem Nervengewebe herausgezogen hätte. Kaum denkbar aber erscheint es, daß auf dem gleichen Wege große und kleine amöboide Gliazellen, wie wir sie Tafel XXVIII, Fig. 14, Tafel XXIX, Fig. 2, Tafel XXX, Fig. 10 sehen, in diese Räume gelangt sind, in welchen sie völlig frei liegen. In manchen der oben beschriebenen großen Ballen, die sich in den perivaskulären Räumen finden, lassen sich noch Kernreste nachweisen, so in einigen der Ballen Tafel XXVIII, Fig. 14, so daß die Abstammung derselben aus degenerierten Gliazellen wahrscheinlich wird. Daß aber alle diese Produkte des perivaskulären Raumes aus degenerierten Gliazellen stammen, ist schon deshalb nicht glaubhaft, weil die Ballen oft viel größer als die größten amöboiden Zellen, viel zu massenhaft und auch viel zu bestimmbar in ihrer Form durch die Härtungsmittel erscheinen. Jedenfalls aber dürfte das gar nicht seltene Vorkommen freiliegender und dabei manchmal sehr kompliziert gebauter amöboider Gliazellen in den perivaskulären Räumen beweisen, daß unter pathologischen Umständen perivaskuläre Räume vorhanden sind. Das sie auch normalerweise existieren, läßt sich damit nicht begründen.

Weiterhin dürften diese Beobachtungen zu der Annahme zwingen, daß in diesen Fällen und an diesen Stellen eine Membrana perivascularis gliae nicht vorhanden ist. Daß es normalerweise auch keine solche gibt, können sie jedenfalls auch wieder nicht dartun. Gerade die Methoden, von denen wir Gebrauch gemacht haben, stellen die gliösen Grenzmembranen nur mangelhaft dar. So können wir durch die direkte Beobachtung sehr wenig erkennen. Doch glaube ich, einige Male gesehen zu haben, wie zahlreiche amöboide Gliazellen, zwischen welchen viele der noch später zu besprechenden Füllkörperchen gelegen waren, eine zarte Membran gegen den perivaskulären Raum vordrängten, oder wie amöboide Zellen in einen perivaskulären Raum, an welchem die Grenzmembran im übrigen Umkreise erkennbar war, an einer Stelle wie durch eine Bruchpforte hineinragten, und daß gerade an dieser Stelle eine Limitans nicht zu sehen war. Immerhin sind solche Beobachtungen nur so vereinzelt zu machen und vielleicht durch Zerrungen und Zerreißungen der Gefrierschnitte erklärbar, daß sie nicht als ganz beweiskräftig betrachtet werden können. Recht häufig aber sieht man, daß Gliazellen halb im Nervengewebe, halb frei im perivaskulären Raum liegen, und namentlich auch, daß die vorhin beschriebenen Ballen und Körnchen aus dem Nervengewebe heraus in die Räume hineinragen. Das dürfte jedenfalls dafür sprechen, daß eine Membrana limitans, wenn sie auch normalerweise vorhanden war, jetzt geschwunden ist.

Bilder, welche von Rinden stammen, in welchen zahlreiche amöboide Zellen in vollster Entwicklung, aber erst einzelne im Zerfall sind, dürften erkennen lassen, daß wenigstens ein Teil der perivaskulären Räume oder die Vergrößerung derselben durch den Zerfall der um das Gefäß herum angehäuften amöboiden Gliazellen zustande kommt. Man sieht zunächst noch das ektodermale Gewebe an der Adventitia anliegen, aber die das Gefäß unmittelbar umlagernden Gliazellen zum großen Teil in Methylblaugranula aufgelöst. Dadurch entstehen schon Lücken im Gewebe, aus welchen schließlich durch Auflösung der Limitans ein perivaskulärer Raum hervorzugehen scheint. Durch solche Lücken können dann ohne Widerstand amöboide Gliazellen in den perivaskulären Raum hineingelangen, ebenso wohl auch Zerfallsprodukte nervöser und gliöser Strukturen, teils in festem, teils in halb verflüssigtem oder verflüssigtem Zustande; aus der Gewebsflüssigkeit, welche den Raum ausfüllt, werden sie dann durch die Härtungsflüssigkeiten ausgefällt.

Etwas verwickelter und weniger übersichtlich als an den großen Gefäßen im Mark erweisen sich die Verhältnisse an den kleinen, besonders an denen der Hirnrinde. Nach der Färbung mit MALLORYschem Hämatoxylin finden wir auch hier bei recht schweren Prozessen ganz ähnliche Produkte wie im Mark. Bei der Säurefuchsinlichtgrünfärbung sieht man auch hier, nur meist viel feinere grün gefärbte Fäden oder Brücken durch den perivaskulären Raum hindurchziehen. Dort, wo die amöboiden Gliazellen der Rinde runde Formen angenommen haben, pflegen auch solche runde Zellen, z. T. noch jung und frisch, meist aber regressiv verändert, frei im perivaskulären Raum zu liegen (Tafel XXVIII, Fig. 3). Wo man Zellen mit langen Reihen fuchsinophiler Granula begegnet, ziehen die Körnerreihen manchmal durch einen perivaskulären Raum hindurch und legen sich an das Gefäß an. Hin und wieder liegt auch der Kern einer solchen Gliazelle frei im perivaskulären Raum, und seine Granula, z. T. gebräunt, z. T. rot gefärbt, wenden sich auf einer Seite dem Gefäß zu und verlieren sich auf der anderen im Nervengewebe. Gar nicht selten finden sich auch Gefäße, um die ein perivaskulärer Raum nicht sichtbar ist, von Anhäufungen fuchsinophiler Granula umlagert, von denen ein Teil in Gliazellfortsätzen zu liegen scheint, während bei einem anderen dies nicht festzustellen ist. Auch sieht man andere in einem weiten, fast völlig leeren Raum gelegenen Gefäße von zahlreichen fuchsinophilen Körnchen bedeckt. Die Deutung solcher Befunde wird natürlich dadurch erschwert, daß uns recht oft eine Entscheidung darüber unmöglich ist, ob wir einen artifiziellen Schrumpfraum oder einen schon im Leben vorhandenen perivaskulären Raum vor uns haben oder ob nicht auch möglicherweise ein wirklicher perivaskulärer Raum durch den Schrumpfungsprozeß noch erheblich erweitert und dadurch das natürliche Verhältnis verzerrt worden ist.

Wo wir nun diese perivaskulären Produkte antreffen, da spielen sich auch regelmäßig an der Adventitia und im adventitiellen Lymphraum Veränderungen ab. Wir finden in den Adventialzellen mehr oder minder reichliche Einschlüsse. Zum Teil sind sie eben so leuchtend rot gefärbt wie die fuchsinophilen Granula, nur meist erheblich größer, zum Teil sind sie mit Osmium gebräunt oder geschwärzt, auch solche die eine geschwärzte Schale und einen roten Kern haben, finden sich häufig darunter. Wenn die Masse der perivaskulären Produkte reichlicher ist, vergrößern sich die Zellen der Adventitia. Normalerweise sieht man in ihm nur Kerne mit wenig Plasma und

Bindegewebsfasern. Jetzt tritt an einzelnen Kernen ein deutlicher Plasmaleib zutage, der bald nur an einem Pol des Kernes, bald an beiden zu längeren, dunkel gefärbten Fortsätzen ausgezogen ist. Auch Zellen, deren Plasmaleib einer runden Form zustrebt, kann man bemerken. Nach Anwendung der Plasmafärbungen läßt sich dann bald ein Netzwerk im Plasma darstellen, während andere Methoden die in den Maschen gelegenen Abbaustoffe zur Darstellung bringen, seltener gerötete, meist gebräunte und geschwärzte Körnchen.

Wenn die Masse der perivaskulären Produkte eine sehr erhebliche ist, stellen sich auch im adventitiellen Lymphraum vereinzelte lymphozytäre Elemente ein, besonders an den größeren Gefäßen des Marks, wo die perivaskulären Produkte gewöhnlich am reichlichsten sind. Dort trifft man auch oft große Rundzellen mit etwas größerem Kern und in ihren Jugendformen gleichmäßig gefärbtem Zelleib, von denen ich glaube, daß sie die Mutterzellen vieler Körnchen bzw. Gitterzellen sind, die wir in späteren Stadien hier antreffen. Tafel XXVIII, Fig. 14 zeigt zwei typische solche Zellen im adventitiellen Lymphraum.

Während wir bei der Anwendung von Osmium und Scharlach nur ganz vereinzelt im perivaskulären Raum geschwärzte oder gerötete Körnchen finden können, ist die Menge der mit Osmium schwärzbaren und mit Scharlach sich färbenden Stoffe in den Zellen der Adventitia und des adventitiellen Lymphraumes häufig eine ganz enorme. Man betrachte nur die Bilder in Tafel XXXIV, Fig. 4, 5 und 7 — Bilder, wie sie sich von allen zur Degeneration des Nervengewebes führenden Rindenerkrankungen gewinnen lassen. Sehr schön tritt das gleiche Verhältnis zutage an Bildern, die von Flemming-Material stammen (Tafel XXX, Fig. 9 und 10), wo wir in den perivaskulären Räumen keine Spur von gebräunten Stoffen sehen, während sie in Fig. 9 in den Zellen der Adventitia sich reichlich finden und in Fig. 10 in großen Körnchenzellen in beträchtlicher Menge aufgestapelt sind. Die Anwendung verschiedener Farbreaktionen zeigt uns, daß es sich bei den Einlagerungen in diese Zellen nicht um einheitliche Stoffe handelt; wir werden später noch darauf zurückkommen müssen.

Weiterhin erleiden diese fettaufspeichernden Zellen zunehmende regressive Veränderungen. Die Kerne werden schließlich ganz an die Peripherie gedrückt, abgeplattet, färben sich diffus und dunkel und nehmen unregelmäßige, eckige Formen an. Allmählich scheinen auch die lipoiden Stoffe wieder aus ihnen ausgelaugt zu werden; denn schließlich findet man oft Zellen, von denen nur Reste eines retikulären Plasmas, aber keine Einlagerungen in demselben mehr nachzuweisen sind.

Wir sehen also, daß dort, wo amöboide Gliazellen in großer Menge im Gewebe auftreten, sich mannigfaltige, eigenartige Stoffe um die Gefäße in perivaskulären Räumen ansammeln. Ihre erheblichen morphologischen Abweichungen bei Anwendung verschiedener Fixierungsmittel machen es wahrscheinlich, daß sie, zum Teil wenigstens, Gerinnungsprodukte aus einer pathologischen Gewebsflüssigkeit sind. Neben diesen findet man amöboide Gliazellen, frischere und solche, die Zeichen des Zerfalles darbieten in denselben Räumen. Das spricht dafür, daß die gliöse Grenzmembran eine Auflösung erfahren hat und daß unter pathologischen Umständen sich perivaskuläre Räume finden, die nicht als Schrumpfräume artifizieller Art gedeutet werden können. Wo sich diese beschriebenen Veränderungen um die Gefäße herum abspielen, wandeln sich dann viele Zellen der Adventitia und gewisse Zellen des adventitiellen Lymphraumes in Fettkörnchenzellen um.

C. Die Pia und die Abbauvorgänge im Nervengewebe.

Wenn sich in den Zellen der Adventitia und des adventitiellen Lymphraumes die eben beschriebenen Vorgänge abspielen, pflegen auch im großen pialen Lymphraum Veränderungen vor sich zu gehen. Man ist früher wohl allgemein der Ansicht gewesen, daß Umbildungen an der Pia, abgesehen von den meningitischen und paralytischen, nur insofern von denen des zentralen Nervensystems abhängig seien, als eine Rindenverschmälerung eine Verdickung der Pia veranlasse. Sie sollte den Zweck haben, den leer gewordenen Raum auszufüllen. Daß aber die Ausfüllung eines leeren Raumes nicht die alleinige Bedeutung der pialen Veränderungen sein kann, ergibt sich schon aus der Beobachtung, daß mikroskopisch nachweisbare Umbildungen an den weichen Hirnhäuten schon dann eintreten, wenn das Nervengewebe noch gar nicht atrophiert ist, ja wenn eine Schwellung der Ganglienzellen und eine erhebliche Vermehrung des gliösen Plasmas, wie sie durch das massenhafte Auftreten amöboider Zellen verursacht wird, eher eine Volumszunahme des Gehirns bedingen muß.

Auch in der Pia sehen wir bei akuten Erkrankungsprozessen zunächst extrazelluläre Produkte auftreten, die im wesentlichen mit denen in den perivaskulären Räumen übereinstimmen, im einzelnen aber auch von ihnen abweichen. Bei besonders schweren Erkrankungsvorgängen in der Rinde sind sie zuweilen in einer ganz außerordentlichen Menge zu beobachten. Im allgemeinen wird ihr Studium dadurch etwas erschwert, daß sich der pialen Flüssigkeit häufig durch die Zerreißung oder Durchschneidung pialer Gefäße, wie sie bei der Sektion leicht eintritt, Blutserum beimengt. Auch werden andererseits pathologische Stoffe der Pia offenbar besonders leicht bei der Härtung von den sie unmittelbar umgebenden Flüssigkeiten aufgenommen. Dagegen ist es hier wieder eher möglich als am Zentralnervensystem, frische Präparate anzufertigen. Man braucht nur die Pia abzuziehen und direkt mit verschiedenen Farblösungen in Berührung zu bringen. Sehr brauchbar erweist sich dazu das Neutralrot (de Montet).

Schon im Nissl-Präparat sehen wir oft eine eigenartige Trübung der Pia, verursacht durch die Einlagerungen von einzelnen Körnchen, körnigen Haufen, Krümeln und Schollen im allgemeinen ungefärbter Substanzen, die bei Abblendung deutlicher hervortreten. Einzelne davon haben auch die basischen Anilinfarben in sehr verschiedener Stärke aufgenommen, andere erscheinen grünlich, den pigmentierten lipoiden Stoffen des Nervengewebes ähnlich. Bei der Weigertschen Gliafärbung sehen wir oft Haufen und Klumpen verschieden großer blau gefärbter Körner, bei Anwendung der Mannschen Farbe ähnliche blau gefärbte Haufen. Sehr rasch stellen sich dann auch Veränderungen an den zelligen Elementen der Pia ein. Die Fibroblasten zeigen genau wie die Zellen der Adventitia der Gefäße eine Vergrößerung ihres Plasmaleibes, bald strebt er einer runden Form zu, bald entwickelt er sich in einen oder zwei große lappige Fortsätze. Färbungen, welche das Plasma darstellen, zeigen dann an dem vergrößerten Zelleib eine netzige Struktur, Färbungen, welche den Inhalt der Maschen veranschaulichen, eine Einlagerung lipoider Stoffe. Sehr bald kann die Menge der abgelagerten fettigen Substanzen eine sehr erhebliche werden. Mit der Karbolfuchsinmethylenblaufärbung lassen sie sich besonders hübsch darstellen. Neben den Fibroblasten finden wir dann immer auch einzelne lymphozytäre Elemente und eigenartige Blutelemente mit einem größeren, runden, gut gefärbten Zelleib, denen wir schon in der adventitiellen Lymphscheide begegnet sind, und die sich später in Gitterzellen umbilden. Dazwischen liegen dann immer auch einige Fibroblasten, welche einen die normale Größe erheblich übersteigenden Umfang angenommen und ganze Reihen von Kernen gebildet haben. Auch sie scheinen sich teilweise mit fettigen Produkten zu beladen.

Im allgemeinen wird man finden, daß die beschriebenen pialen Veränderungen auf der Höhe der Windungen weniger ausgeprägt sind als in den Furchen und besonders über den Windungstälern. Vielleicht liegt das daran, daß sich hier gewöhnlich mehr Lymphe ansammelt, vielleicht auch daran, daß hier Abbauprodukte aus zwei gegenüberliegenden Rindenabschnitten zusammenkommen.

Mit den Veränderungen im Nervengewebe, welche durch das Auftreten amöboider Gliazellen gekennzeichnet sind, gehen also auch Veränderungen in der Pia Hand in Hand. Sie machen sich bemerkbar durch eine Ablagerung verschiedenartiger extrazellulärer Stoffe, durch Vermehrung der zelligen Elemente und Umwandlung derselben in mit lipoiden Produkten beladene Körnchenzellen.

D. Die Füllkörperchen und ihre Beziehungen zur Glia.

In Schnitten des Zentralnervensystems, in welchen sich zahlreiche amöboide Zellen finden, begegnen wir so häufig eigenartigen extrazellulären Bildungen, daß man annehmen muß, sie stehen in engen Beziehungen zu dem Krankheitsvorgang. Besonders eine Form scheint in ihrem Vorkommen eng an das Vorhandensein amöboider Gliazellen gebunden und jedenfalls schon wegen der großen Menge, in welcher sie sich findet, nicht ohne wesentliche pathologische Bedeutung. Sie wird deswegen auch am besten im Zusammenhang mit den amöboiden Zellen besprochen. Andere extrazelluläre pathologische Produkte, die auch ohne Begleitung amöboider Gliazellen vorkommen, sollen später betrachtet werden.

Ich will diese besonderen extrazellulären Gebilde Füllkörperchen nennen. Es ist nicht leicht, zu einem klaren Verständnis derselben zu kommen. Am ehesten kann man sich wohl am Rückenmark, wo die Verhältnisse am einfachsten und übersichtlichsten sind, ein Urteil über ihre Herkunft bilden. In die Zeichnungen 19 und 20 der Tafel XXIX, die beide Ausschnitte aus der weißen Substanz des Rückenmarks darstellen — Fig. 19 von einer normalen, halbwüchsigen Ziege, die im übrigen in allem wesentlichen der weißen Substanz

des normalen menschlichen Rückenmarks gleicht, Fig. 20 von einem an progressiver Chorea verstorbenen Menschen — sind mit großer Sorgfalt alle Einzelheiten eingezeichnet, mit Ausnahme der rot gefärbten Markscheiden.

In Fig. 19 sieht man nun zwischen den Markfasern einzelne Gliakerne mit deutlichem Protoplasmaleib und zahlreiche Gliafasern, von welchen ein Teil im Längsverlauf, ein anderer in kurzen Schrägschnitten, die meisten in punktförmigen Querschnitten getroffen sind. Eine ganz zarte Färbung der zwischen den Fasern gelegenen Substanz zeigt an, daß diese in dem eigenartig modifizierten Plasma des Gliaretikulums eingelagert sind.

Fig. 20 bietet nun ganz andere Verhältnisse. Von Gliafasern ist auch nicht eine Andeutung zu sehen. Eine Anzahl großer, blasser und einzelne satt gefärbte amöboide Gliazellen beherrschen das Bild. Neben diesen sehen wir dem Protoplasma der Gliazellen ähnlich gefärbte, mosaikartig zusammengefügte Körperchen den Raum zwischen den wohl etwas verkleinerten Markfasern ausfüllen. Das sind die Füllkörperchen. Stellt man sich vor, daß jede der in Fig. 19 gezeichneten feinen Fasern, von denen wir in Fig. 20 gar nichts mehr sehen, enorm aufgequollen wäre, so würde man sich das Zustandekommen eines solchen Bildes einigermaßen erklären können. Zunächst habe ich es auch so gedeutet. Doch so einfach liegt die Sache offenbar nicht, denn es muß auffallen, daß man höchst selten eine zu einem Bande verbreiterte, längs getroffene Faser entdecken kann. Auch wenn man, um das Verhältnis besser klar zu stellen, statt der Querschnitte Längsschnitte durch das gleiche Rückenmark macht, trifft man immer nur auf die gleiche mosaikartige Felderung und nur selten auf längere bandartige Streifen. Besonders um die Gefäße herum bilden die Füllkörperchen vielschichtige, dicke Mäntel. Das muß darauf hindeuten, daß wir hier nicht Querschnitte langer protoplasmatischer Zellfortsätze, sondern freiliegende Körperchen vor uns haben, die nicht mit Zellen in Verbindung stehen und wie Pflastersteine neben- und übereinander gefügt sind.

Wo kommen nun diese Körperchen her? Wenn man einige Stellen der Abbildung betrachtet, so besonders die Nachbarschaft der amöboiden Zelle rechts oben, könnte man auf den Gedanken kommen, daß sie von amöboiden Zellen abgestoßen werden. Wir begegnen solchen Körperchen auch in der grauen Substanz recht häufig in der Nachbarschaft amöboider Zellen, so in Tafel XXXV, Fig. 3, wo sie rechts unten in großer Zahl neben einer Gruppe amöboider Zellen liegen. Aber ein derartiger Vorgang würde doch so wenige Analogien haben, daß man noch weitere Beweise dafür verlangen muß. Gelegentlich findet man dann auch große Füllkörperchen, die ganz dem Zelleib einer amöboiden Gliazelle gleichen, nur keine Kerne enthalten. Es mögen von Gliazellen abgeschnittene Stücke sein. In anderen sieht man auch Reste degenerierter Kerne, sie sind also wohl aus degenerierten Zellen hervorgegangen. Für alle aber kann das kaum zutreffen, man muß sich nur die große Anzahl, in der diese Gebilde gelegentlich auftreten (Tafel XXIX, Fig. 1, Tafel XXXV, Fig. 2, 3, 4) vor Augen führen.

Jedenfalls bleibt es eine beachtenswerte Beobachtung, daß Gliafasern dort fehlen oder jedenfalls stark vermindert sind, wo Füllkörperchen in erheblicher Zahl auftreten. So muß man daran denken, ob sie nicht etwa einer postmortalen Veränderung der Gliafasern ihre Entstehung verdanken. Seit Weigerts Arbeiten wissen wir ja, daß diese schon bald nach dem Tode zerfallen können.

Ein glücklicher Zufall hat es ermöglicht, diese Frage, wie ich glaube, einwandfrei zu lösen. Eine Anzahl Makaken, die ich zu experimentellen Untersuchungen bezogen hatte, zeigte sich an einer Seuche erkrankt, die sich in hartnäckigen Diarrhöen äußerte. Sie magerten stark ab, und die Sektion eines gestorbenen ergab, daß er Geschwüre im Darm und Eiterherde im Gehirn hatte. Da auch die anderen verloren schienen, tötete ich sie und legte das Zentralnervensystem lebenswarm in die verschiedenen Fixierungsflüssigkeiten.

In der grauen und weißen Substanz des Rückenmarkes fanden sich nun amöboide Gliazellen neben Ganglienzellenveränderungen, welche der schweren Erkrankung Nissls entsprachen. Dabei war der Grad der Veränderungen bei verschiedenen Tieren und an verschiedenen Stellen so wechselnd, daß sich Bilder wie in Tafel XXIX, Fig. 19 und 20 von ihnen gewinnen ließen. Es waren also alle Stadien desselben Prozesses miteinander zu vergleichen. In den annähernd normalen Partien fand sich die gliöse Randschicht nach Säurefuchsinlichtgrünfärbung aus außerordentlich feinen, zarten, rot gefärbten Gliafasern gebildet. Nur ein ganz zarter grünlicher Ton, der zwischen den Fasern lag, deutete an, daß auch sie in einer protoplasmatischen Substanz eingebettet waren. An anderen Stellen mit deutlichen Veränderungen sah man keine roten Fasern, sondern

grünlich gefärbte, etwas breitere Bänder, ungefähr in derselben Anordnung, wie sie sonst die Gliafasern zeigen, aber viel breiter wie diese, und man konnte wahrnehmen, wie einzelne davon zu Kernen in Beziehung standen, die unter der Randschicht oder in dieser selbst lagen. Dazwischen traten schon einzelne Zellen vom Charakter junger amöboider Gliazellen auf und zahlreiche rundliche und eckige Gebilde, unsere Füllkörperchen. In Stellen noch vorgeschrittener Erkrankung beherrschten amöboide Zellen und Füllkörperchen das Bild. Von Fasern, überhaupt von einer Anordnung, die der normalen Oberflächenschicht ähnelte, war gar nichts mehr zu beobachten. Unter den Füllkörperchen lagen auch einige mehr gerinnselartige, körnige Gebilde von unregelmäßiger Form, vielleicht Gerinnungsprodukte veränderter Lymphe, die sich zwischen den Gewebselementen befunden hatte, vielleicht in Auflösung begriffene Füllkörperchen.

Aus diesen Bebachtungen dürfte sich mit Sicherheit ergeben, daß sich unter pathologischen Bedingungen Gliafasern wieder auflösen. Das dürfte wohl nach unseren Präparaten so vor sich gehen, daß die Faser mit dem ja meist noch an ihr vorhandenen Plasma aufquillt, und daß dann diese gequollenen Strukturen zerfallen und dabei Anlaß zur Entstehung der Füllkörperchen werden.

Die Bilder Tafel XXIX, Fig. 4, 5, 8, 16, die wir schon früher besprochen haben, stellen ähnliche Vorgänge dar. Lange protoplasmatische Fortsätze von Gliazellen nehmen eine rosenkranzähnliche Form an und zerfallen dann in die einzelnen Glieder, die den Füllkörperchen entsprechen würden.

Im menschlichen Zentralnervensystem finden wir leicht einzelne Stadien des gleichen Vorgangs, den wir eben an der Hand eines Materials, das die Lückenlosigkeit eines Experimentes zeigt, verfolgen konnten, und eine Zusammenstellung der einzelnen, aus verschiedenen Erkrankungsfällen gewonnenen Bilder spricht für dieselbe Entstehungsweise der Füllkörperchen. In einem Falle einer ganz frischen tuberkulösen Meningitis des Rückenmarkes fanden sich an einzelnen Stellen die Fasern der Randschicht ersetzt durch protoplasmatische Fasern von erheblicher Breite, von denen zahlreiche bis in die etwas angeschwollenen Zelleiber von Gliazellen zu verfolgen waren, die in oder unter der Randschicht lagen. Dazwischen fanden sich einige amöboide Gliazellen. An einzelnen Stellen war die Membrana limitans stark vorgewölbt und Haufen von großen amöboiden Zellen lagen darunter. An anderen sah man keine Membrana limitans mehr, und amöboide Zellen hatten sich hier über die ursprüngliche Grenze des Rückenmarks in das piale Gewebe vorgeschoben. Es dürfte an solchen Stellen dann leicht im Stadium der Reparation zu einer Herauswucherung der Gliafasern über die frühere Oberflächenschicht kommen, wie man das öfters bei alten Paralysen und den chronischen Formen der Meningomyelitis beobachtet.

Ein anderes, vorgeschritteneres Stadium zeigt Fig. 4 der Tafel XXXV. Es stammt von einer sehr stürmisch verlaufenen Paralyse, die an einer Sepsis zugrunde gegangen war. Möglicherweise ist die letztere nicht ohne Einfluß auf die Entstehung der Veränderungen gewesen. Oben sehen wir dunkelgrün gefärbte bindegewebige Fasern der Pia. Dieselbe ist nur teilweise gezeichnet. Ohne deutliche Grenzmembran beginnt die gliöse Oberflächenschicht des Rückenmarks, die fast $^2/_3$ der Zeichnung ausfüllt; erst im unteren Drittel stoßen wir auf gelblich und bräunlich gefärbte Querschnitte größerer und kleinerer Markfasern. In der Randschicht, welche normalerweise nahezu ausschließlich aus rot gefärbten Quer- und Längsschnitten von Gliafasern gebildet wird, sehen wir nur einzelne rote Pünktchen, die zum Teil Gliafasern entsprechen. Der weitaus größte Teil des Gewebes wird von Füllkörperchen gebildet, die sich auch noch zwischen den Markfasern in kleinen Häufchen eingestreut finden.

Der Grad der Veränderungen wird uns sehr verständlich, wenn wir dagegen die Fig. 5 derselben Tafel betrachten, welche von einer chronischen Paralyse stammt. Auch hier sehen wir wieder ganz oben etwas von den Bindegewebsfasern der Pia, dann die hier sehr deutlich ausgeprägte Membrana limitans und die fast $^4/_5$ des Bildes erfüllende, verdickte Randschicht, welche nahezu ausschließlich aus rot gefärbten Gliafaserquerschnitten besteht. Nur weniges, netziges, zart gefärbtes Protoplasma findet sich dazwischen, von Füllkörperchen ist gar nichts zu sehen.

Wie ungeheuer massenhaft diese Gebilde in der grauen Substanz unter pathologischen Umständen gelegentlich auftreten, sehen wir am besten, wenn wir die Fig. 1 u. 2 der Tafel XXXV vergleichen. Beide stellen Vorderhornzellen des Rückenmarks dar: Fig. 1 eine normale Zelle von einem Hunde, Fig. 2 eine Vorderhornzelle eines Falles von progressiver Chorea. Weitaus die überwiegende Mehrzahl der grünen Fleckchen in Fig. 2 sind Füllkörperchen, da die feineren Achsenzylinder kaum gefärbt und viel feiner sind. Auch in Fig. 3 sehen wir das ganze Gewebe von solchen Körperchen erfüllt, ebenso

zeigt uns Tafel XXIX, Fig. 1 ihre außerordentliche Menge nach Darstellung durch eine andere Färbung. Es ist nicht wahrscheinlich, daß Füllkörperchen nur dort gebildet werden, wo Gliafasern vorhanden sind, denn wir sehen sie zuweilen auch in der Hirnrinde in großer Anhäufung. So habe ich einige Fälle von Status epilepticus und einen Fall von Infektionsdelir gesehen, wo sie die ganze tiefe Rinde erfüllten. Dort ist es noch viel schwerer, ihre Entstehung zu verfolgen, am wahrscheinlichsten dürfte sein, daß sie aus einem Zerfall der retikulären Glia hervorgehen.

Mit den Füllkörperchen dürfen andere Gebilde nicht verwechselt werden, die mir vorzugsweise in der Nachbarschaft von Geschwülsten und herdförmigen Erkrankungen verschiedener Art begegnet sind und dieselben Farbreaktionen und die gleiche homogene Beschaffenheit wie die Füllkörperchen haben, aber gelegentlich eine ganz enorme Größe erreichen. Man findet sie dort besonders in der Nachbarschaft der Gefäße, einen perivaskulären Raum ausfüllend, gelegentlich aber auch in Gewebslücken, die nicht zu Gefäßen in Beziehungen zu stehen scheinen. Ich kann nach der ganzen Art ihres Vorkommens nur annehmen, daß sie durch unsere Härtungsflüssigkeiten zur Gerinnung gebrachten pathologischen Gewebsflüssigkeiten ihre Entstehung verdanken. So kann man sich wohl auch nur erklären, daß man gelegentlich amöboide Gliazellen mitten in ihnen eingeschlossen findet. Das muß uns aber an den Einwand denken lassen, ob nicht auch die Füllkörperchen die gleiche Entstehung haben können. Ich glaube, daß sich das für die meisten Füllkörperchen ausschließen läßt, besonders durch ein genaueres Studium der Präparate, wie sie Tafel XXIX, Fig. 20 darstellt, und durch eine vergleichende Betrachtung der Veränderungen der Randschicht des Rückenmarks, wie sie oben geschildert worden ist. Auch schon ihre ganze Ablagerungsart spricht wohl dagegen. Man würde dann wohl kaum solche kleine, pflastersteinartig nebeneinander gefügte, scharf umgrenzte Körperchen, sondern große, die Gewebslücken völlig ausfüllende Coagula antreffen, wie wir dies ja auch in der Nachbarschaft von Geschwülsten beobachten. Dabei sehen wir, daß sie nach Anwendung verschiedener Härtungsmittel sehr weitgehende Übereinstimmung der Form zeigen, während wir gerade bei den Gerinnungsprodukten sehr verschiedenartig geformte Gebilde erhalten, je nachdem wir die eine oder die andere Fixierung anwenden. Für einzelne zwischen den Körperchen gelegene gerinnselartige Bildungen wird man die Frage offen lassen müssen, ob sie einer Gerinnung im Leben flüssiger oder halbflüssiger Substanzen, oder einer pathologischen Lymphflüssigkeit ihre Entstehung verdanken, oder teilweise aufgelöste Füllkörperchen darstellen.

Es scheint mir, daß die Füllkörperchen nur eine kurze Lebensdauer haben, allmählich unter stärkerer Aufquellung mehr und mehr ihre Färbbarkeit einbüßen und schließlich vollständig aufgelöst werden. Jedenfalls finden wir bei Krankheitsprozessen, die zu einem Stillstand gekommen sind, bald nichts mehr von ihnen, und auch bei frischeren Erkrankungen sieht man neben den solideren und scharf abgegrenzten schon immer solche, die sich ungemein schwach färben und ihre homogene Beschaffenheit verloren haben.

Jedenfalls sind diese Füllkörperchen für das Verständnis der mit der Bildung von amöboiden Gliazellen einhergehenden Krankheitsvorgänge von großer Bedeutung. Es sind kleinere und größere, rundliche und eckige, bald zerstreut im Nervengewebe, bald zu Haufen oder mosaikartigen Felderungen zusammengefügte Gebilde, die sich wenigstens zu Anfang mit Lichtgrün, Methylblau und Malloryschem Hämatoxylin intensiv färben, später aber ihre Färbbarkeit mehr und mehr einbüßen und einer Auflösung zu verfallen scheinen. Die Beobachtung spricht dafür, daß sie jedenfalls zum größeren Teil aus dem Zerfall pathologisch veränderter Gliastrukturen hervorgehen.

E. Die Beziehungen der amöboiden Gliazellen zu den normalen Gliastrukturen, zur pathologischen faserigen Glia und zum pathologischen Gliaretikulum. Dauerformen der amöboiden Gliazellen.

Zum Verständnis der Bedeutung der amöboiden Gliazellen ist es weiter noch von Wichtigkeit festzustellen, wie sie sich zu den normalen Gliastrukturen verhalten, ob sie in der Hauptsache aus neu entstandenen Gliazellen sich bilden, und die übrigen Gliastrukturen dabei unversehrt weiter bestehen, oder ob sie aus einer Umwandlung der normalen Gliazellen hervorgehen, und dabei von welchen Formen: den mit feinen protoplasmatischen Fortsätzen oder den runden, die Körnchen im Protoplasma führen, und weiter wie sie sich zu den fasernbildenden Zellen verhalten. Dann müßte noch geprüft werden, wie nach Verlauf eines akuten Prozesses in einem Nervengewebe, in welchem amöboide Gliazellen ihre Tätigkeit entfaltet haben, die

jedenfalls mit einem Untergange vieler derselben endet, das Stützgewebe sich wieder herstellt.

Völlige Klarheit über alle diese Fragen zu erlangen, ist heute noch unmöglich, weil man das Gliaretikulum nicht genügend darstellen kann. Einzelnes wird sich auch wohl nur an der Hand lückenloser experimenteller Serien beantworten lassen, die zu bearbeiten, ich noch nicht genügend Zeit gefunden habe. Darum lassen sich bis jetzt nur einzelne Beobachtungen mitteilen, die zwar auf manche der Fragen ein Licht werfen können, die ganzen Zusammenhänge aber noch nicht in voller Klarheit erkennen lassen.

Zunächst ist wohl sicher, daß, wenigstens bei manchen besonders stürmischen Krankheitsprozessen, nicht wenige der normalen Gliazellen zugrunde gehen, ehe die Bildung amöboider anhebt. Über den Umfang dieses Untergangs bei verschiedenartigen Prozessen müssen noch eingehende Untersuchungen angestellt werden. Geeignetes Material vom Menschen zu erhalten, ist überaus schwierig. Jedenfalls aber macht ein solcher Untergang gliöser Elemente wahrscheinlich, daß auch ein Teil der retikulären Gliabildungen zerfällt, und daß andere gliöse Strukturen an seine Stelle treten. Möglicherweise entstehen auch bei diesem Zerfall Füllkörperchen, ein bestimmter Nachweis ist aber heute noch unmöglich.

Man sieht dann Bilder, die es wahrscheinlich machen, daß amöboide Gliazellen sowohl aus neugebildeten, durch indirekte Kernteilung entstandenen, als auch aus alten Zellen mit protoplasmatischen Fortsätzen und runden Zellen mit Protoplasmagranula hervorgehen können. Das erstere dürfte dadurch bewiesen werden, daß oft gleichzeitig neben den ersten Entwicklungsstadien amöboider Gliazellen zahlreiche Karyokinesen zu sehen sind, während man in späteren Stadien fast nur amöboide Zellen antrifft. Dagegen scheint auch wieder in anderen Fällen kaum eine größere Zellvermehrung der Bildung amöboider Zellen voranzugehen. Bilder, wie Tafel XXVIII, Fig. 2*b* scheinen dafür zu sprechen, daß sich Zellen mit protoplasmatischen Fortsätzen in amöboide Zellen umwandeln, Bilder wie Tafel XXVIII, Fig. 7*a*, daß kleine, runde Gliazellen mit Protoplasmagranula dasselbe tun. In wie großer Zahl die einzelnen Zellformen sich an der Bildung amöboider Zellen beteiligen, ist aber schwer festzustellen. Manchmal hat man den Eindruck, daß sogar Gliazellen im Mark, die schon Fasern gebildet haben, über diese Fasern hinaus anschwellen, und daß die Gliafasern zerfallen, während sich das Plasma satter färbt und die Form einer amöboiden Gliazelle annimmt, So würden auch Zellen, die schon Fasern gebildet haben, sich in amöboide umwandeln können. Eisath hat mich zuerst darauf aufmerksam gemacht, daß in Präparaten, die nach seiner Methode behandelt sind und allem Anscheine nach die protoplasmatischen Gliastrukturen gut darstellen, und in welchen sich amöboide Gliazellen entweder nur im Mark oder in Mark und Rinde gleichzeitig finden, fast niemals die an zarten protoplasmatischen, vielverzweigten Fortsätzen reichen Gliaelemente zu sehen sind, wie sie Tafel XXVIII, Fig. 1*a* und *b* wiedergibt, und wie sie sich in der normalen Hirnrinde mühelos färben lassen. Auch meine Beobachtungen scheinen dies für viele Fälle zu bestätigen. Das würde darauf hindeuten, daß mit dem Auftreten amöboider Gliazellen auch die übrigen normalen Gliastrukturen wesentliche Veränderungen erfahren. An Stelle der verzweigten Gliazellen sieht man dann bei der gleichen Färbemethode meist solche mit ganz wenig vermehrtem Plasma ohne Fortsätze, oder solche mit etwas vergrößertem Zelleib und etwas reichlicherer Körnchensubstanz. Diese Beobachtung verdient gewiß große Beachtung. Da es mir aber wiederholt möglich gewesen ist, in solchen Rinden, in welchen mit der Malloryschen Hämatoxylinfärbung keine Zelle mit protoplasmatischen Fortsätzen mehr darstellbar war, nach Anwendung der Golgi-Methode vielverzweigte Zellen zu bekommen, möchte ich mit der Deutung dieses Befundes vorsichtig sein und noch nicht behaupten, daß diese Fortsätze zugrunde gehen oder von den Zellen eingezogen werden. Vielleicht dürften sie nur nicht mehr dieselbe Affinität für das Mallorysche Hämatoxylin zeigen.

Besonders bemerkenswert ist, daß mit der fortschreitenden Ausbildung amöboider Gliazellen die faserige Glia in großer Ausdehnung zugrunde gehen kann. Wenn man in manchen Rinden und auch in manchem Rückenmark keine oder nur sehr dürftige Gliafasern darstellen kann, so liegt das nicht immer an einem Versagen der Weigertschen Methode, sondern auch manchmal daran, daß die alte Faserglia in größerer oder geringerer Ausdehnung der Auflösung verfallen ist. Ich habe mich lange gegen diese Annahme gesträubt, möchte auch empfehlen, nicht aus jedem Gliapräparat, das eine Verminderung der Fasern zeigt, den Schluß zu ziehen, daß die Fasern zugrunde gegangen sind. Jedenfalls aber ist es bei bestimmten Krankheitsprozessen ein gewöhnliches Vorkommen und zweifellos

festzustellen. Bei der Besprechung der Füllkörperchen habe ich schon Beweise dafür beigebracht und dargelegt, daß es sich dabei nicht etwa um postmortale Veränderungen handeln kann. So verwandelt sich also das normale Stützgewebe des Nervensystems bei manchen akuten Krankheitsprozessen so gründlich, daß wenig mehr von seiner früheren Struktur übrig bleibt.

Im übrigen sehen wir nach der Art der Krankheit ein recht verschiedenes Verhältnis zwischen der amöboiden und der neugebildeten faserigen Glia. Bei der Paralyse z. B. entwickeln sich von vornherein häufig beide Formen neben einander. Neben Gliazellen mit massigem Plasma, aus dem sich ganze Bündel dicker Gliafasern bilden, liegen riesige amöboide Gliazellen in allen Stadien der Entwicklung und des Zerfalls. Mit dem schnelleren Fortschreiten des Prozesses scheinen die amöboiden Zellen, bei langsamem Fortschreiten die faserbildenden in den Schnitten überwiegend. Ähnliches sehen wir um frische Herde herum und bei akuten luetischen Prozessen, besonders den endarteritischen, auch bei rasch fortschreitender seniler Demenz. Bei anderen akuten Krankheitsprozessen — und dazu gehört die akute Dementia praecox — sehen wir dagegen oft neben den amöboiden Zellen keinerlei Neigung zu Faserbildung. Sie wird auch in den chronischen Fällen selten erheblich, ja fehlt manchmal ganz. Auch bei der genuinen Epilepsie beschränkt sie sich meist nur auf die Oberflächenschicht oder das Marklager. Beim Alkoholismus, der Chorea progressiva bleibt sie unbedeutend. Dasselbe scheint für viele Formen der Idiotie zuzutreffen. Sicherlich dürften in diesen auffallenden Unterschieden sehr tiefgreifende Abweichungen im Wesen der einzelnen Krankheitsprozesse zum Ausdruck kommen, wenn uns auch heute noch ein völliges Verständnis ihrer Bedeutung versagt ist. Im allgemeinen wird man wohl sagen können, daß der Grad der Verdickung der Gliafaserrandschicht bei vielen Krankheitsprozessen mit dem Grade der Ausfälle in der Rinde Hand in Hand geht. Aber bei einzelnen Fällen, besonders bei manchen Formen der Idiotie, scheint auch dies nicht zuzutreffen. Die Bildung von Gliafasern in der Hirnrinde selbst ist aber vielleicht von ganz anderen Umständen abhängig als von der Menge untergehenden Nervengewebes, so vielleicht von statischen Momenten, von der Gefäßvermehrung, von der Infiltration der Lymphscheiden usw.

Wie gestalten sich aber nun die Verhältnisse der feineren Gliastrukturen bei jenen Krankheiten, die aus einem akuten Stadium, in welchem wohl eine Bildung amöboider Zellen stattgefunden hatte, in ein chronisches übergegangen oder stationär geworden sind, und bei welchen keine oder nur sehr geringe Gliafaserbildung stattgefunden hat? Manchmal scheint sich hier wieder ein dem ursprünglichen Zustande ähnlicher auszubilden. Man sieht dem Normalen ziemlich nahe kommende Gliazellbilder, häufig aber bleiben mancherlei Abweichungen zurück. Doch bedürfen diese Verhältnisse noch eines genaueren Studiums; jetzt ist es noch sehr schwierig, sich durch die verschiedenartigen Befunde hindurch zu helfen. Nur auf einzelne Beobachtungen möchte ich noch eingehen, die heute schon geklärter erscheinen und eine größere allgemeine pathologische Bedeutung haben dürften.

Wir haben schon gesehen, daß die Fettcystchen, die sich in den amöboiden Zellen bilden, auch in faserbildenden Zellen vorkommen (Tafel XXX, Fig. 3*a*). Wo noch nicht der ganze Zelleib in solche Cystchen umgewandelt ist, begegnen wir dann auch während eines Fortschreitens der Krankheit fuchsinophilen Granula in den Zellen. Solche Zellen würden also doppelte Arbeit leisten, Fasern bilden und die Aufgabe amöboider Zellen teilen. In den großen faserbildenden Gliazellen vermissen wir meist die lipoiden Cystchen, sehen aber dafür oft solide Fettkörnchen, ja derbe Fettkugeln. Die fuchsinophilen Granula, die sich in ihnen finden, sind gleichfalls von denen der amöboiden Zellen verschieden. Tafel XXX, Fig. 5 und 6, stellt solche Zellen von einer Paralyse nach S-Fuchsinlichtgrünfärbung dar. Im Mittelpunkt des Zelleibes, der dann im Nissl-Präparat meist weniger intensiv gefärbt ist und oft einen eigenartig speckigen Glanz zeigt, liegt dicht zusammengehäuft eine Wolke staubartiger, feinster, roter Granula, die gegenüber den fuchsinophilen Granula der amöboiden Gliazellen geradezu winzig erscheinen. Mit dem zunehmenden Alter der Zellen vermehren sie sich, bis schließlich der ganze Zelleib von ihnen so erfüllt sein kann, daß von dem Plasma kaum mehr etwas sichtbar ist. Die Neigung dieser Körnchen, sich in lipoide Stoffe umzuwandeln, ist offenbar gering. Nur hin und wieder bemerkt man einige geschwärzte Granula dazwischen. Auch Methylblaugranula habe ich in solchen großen faserbildenden Zellen nie gesehen. Aus diesen verschiedenen Abweichungen in den feinsten Struktureigentümlichkeiten dürften wohl auch bedeutsame Unterschiede in den Lebensvorgängen dieser verschiedenen Elemente erschlossen werden können.

Wie wir schon gesehen haben, ist die Lebensdauer der amöboiden Zellen im allgemeinen eine zeitlich eng begrenzte. Einzelne aber wandeln sich unter besonderen Umständen in eine Art von Dauerform um. Solche Dauerformen lassen sich wieder verschiedene unterscheiden. In Tafel XXXIII, Fig. 13 ist eine solche Form abgebildet, welcher man nicht selten begegnet. Sie stammt aus einem mit Toluidinblau gefärbtem Alkoholschnitt. Der Kern ist sehr dunkel und homogen gefärbt, das Plasma zeigt eine sehr geringe Färbbarkeit und einen eigenartigen Glanz, der sich in der Zeichnung nicht wiedergeben läßt. Jedenfalls deutet er an, daß es sich um ein modifiziertes Plasma handelt. Auffallend an diesen Formen ist auch der ungemein scharf hervortretende Rand des Zelleibes.

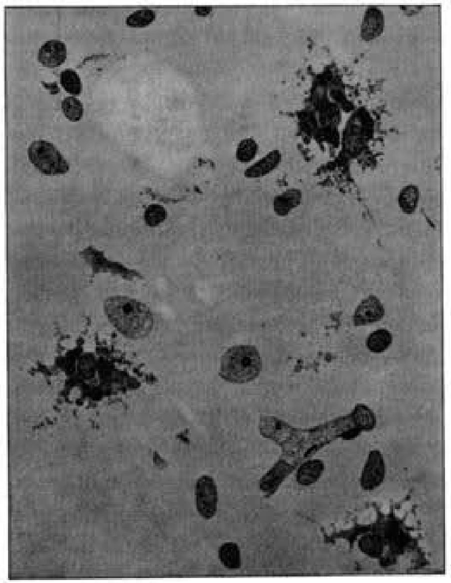

Fig. 1. Eigenartige Dauerformen amöboider Gliazellen aus der Hirnrinde eines idiotischen Epileptikers. WEIGERTsche Gliamethode.

Bei anderen Formen wieder tritt die Beziehung zu amöboiden Zellen nicht ganz so klar zutage, wird aber aus ihrer Form wahrscheinlich. Textfigur 1 gibt solche Zellen wieder. Sie stammen aus der Hirnrinde eines Falles von Idiotie mit Epilepsie und sind mit Hilfe der WEIGERTschen Gliamethode dargestellt. Der Kern scheint freizuliegen, und um ihn herum sind Platten, verzweigte Schollen, einzelne Bröckchen und Stückchen einer mit Methylviolett intensiv gefärbten Substanz angeordnet. Zuweilen finden sich einzelne Partikel weitab von den um den Kern herum angehäuften blauen Massen im Gewebe. Das Gesamtbild ähnelt unverkennbar dem einer amöboiden Zelle, nur ist die Zelleibsubstanz verändert, dem Anschein nach vom Kern abgelöst und in der Nähe

des Kerns in große, an den Fortsätzen in kleinere Bruchstücke zerfallen, die unter sich gar nicht mehr zusammenzuhängen scheinen. Da der Kern keine ausgesprochen regressiven Veränderungen zeigt, ist aber nicht wohl anzunehmen, daß er ganz vom Zellplasma abgelöst ist. Darum wird es wahrscheinlich, daß er noch durch ungefärbtes Plasma mit den mit Methylviolett gefärbten Schollen zusammenhängt. Da wir schon andere Gliazellformen kennen gelernt haben, bei welchen das Plasma die Neigung zeigte, in Stücke zu zerfallen (Füllkörperchen), so würde auch hier ein derartiger Zerfall des Zelleibes nicht als etwas unerhörtes erscheinen. Bemerkenswert aber bleibt die eigenartige Farbreaktion der Schollen und Brocken. Ihr färberisches Verhalten läßt daran denken, daß sie mit den WEIGERTschen Gliafasern stofflich verwandt sind. Dann würden wir den Fall haben, daß Gliazellen am Rande ihres Protoplasmakörpers statt Gliafasern plattenartige Gebilde aus der gleichen Substanz abscheiden. Aber natürlich kann die eine Farbreaktion die Richtigkeit einer solchen Annahme nicht erweisen. Man kann diese Schollen auch sehr gut färben, wenn man Gefrierschnitte aus der Gliabeize in wäßrigem Hämatoxylin erhitzt und mit WEIGERTscher Borax-Ferridcyankaliumlösung entfärbt. Dann sind keine Gliafasern daneben gefärbt. Aber auch das weist nicht mit Notwendigkeit darauf hin, daß sie aus einer anderen chemischen Substanz bestehen wie die Gliafasern, da im undifferenzierten Präparat beide gefärbt sind und die Nichtentfärbung der Schollen bei der Differenzierung mit ihrer viel erheblicheren Masse zusammenhängen kann. Auch der Umstand, daß sie ungefärbt stark lichtbrechend sind, spricht aus dem gleichen Grunde nicht dagegen. Aber in der Säurefuchsinlichtgrünfärbung zeigen sie eine deutliche Neigung, sich grün zu färben, ganz im Gegensatz zu den Gliafasern, die eine starke Affinität zum S-Fuchsin zeigen. Das dürfte dafür sprechen, daß hier Teile des Plasmas der amöboiden Gliazellen sich in eine eigenartige Substanz umgewandelt haben, die von der Substanz der Gliafasern abweicht.

Auch die Neubildung eines Gliaretikulums dürfte unter manchen Umständen unter der Mitwirkung von Zellen stattfinden, welche der amöboiden Glia nahestehen. So zeigt uns Tafel XXXV, Fig. 6 einen Schnitt durch den enorm atrophischen Streifenhügel eines Falles von Chorea progressiva, in welchem alle Ganglienzellen aufs schwerste degeneriert sind. Auch von einer faserigen Glia ist keine Spur zu

bemerken. Dagegen wird das Stützgewebe gebildet von eigenartigen protoplasmatischen Gliazellen, die manche Ähnlichkeit mit amöboiden Gliazellen zeigen, besonders auch darin, daß sich Anhäufungen von fuchsinophilen Granula und kleinste Cystchen in ihnen finden. Um die einzelnen Gliakerne herum liegt ein außerordentlich zart gefärbter, wohl von etwas modifiziertem Plasma gebildeter Zellkörper, der weiter entfernt vom Kern von zahlreichen Löchern durchsetzt ist und sich noch weiter ab in viele Fortsätze aufteilt, die schließlich in ein Retikulum einer gleichartigen Substanz einzutreten scheinen, das mit anderen Kernen in Beziehung steht und das ganze Gewebe durchzieht. Man wird durch ein solches Präparat nicht beweisen können, daß hier alle Zellgrenzen verwischt sind. Jedenfalls aber zeigt es, daß auch komplizierte und anscheinend dauerhaftere, eine Art Narbenbildung darstellende Gliastrukturen zustande kommen, ohne daß eine Faserbildung dabei stattfindet, unter Mitwirkung von Zellen, die der amöboiden Glia nahestehen.

Ganz ähnliche Bilder habe ich aus der Markleiste eines Falles einer noch sehr jugendlichen Dementia praecox erhalten. Auch hier fanden sich neben amöboiden Zellen, die sich an ihren Rändern unter Einschiebung zahlreicher Lücken immer mehr aufzuteilen und in ein Retikulum aufzulösen schienen, in großen Teilen des Marks netzige plasmatische Bildungen, die wohl von Zellen gleicher Art erzeugt waren.

Interessante Bilder, welche ein Licht auf die Wiederherstellung netziger Gliastrukturen werfen können, sind mir schließlich in der Nachbarschaft von blutigen Erweichungsherden, die mehrere Wochen alt waren, bei Anwendung unserer Methoden begegnet. In Partien der weißen Substanz, die nicht direkt geschädigt waren, in welchen sich aber bei der Anwendung des MANNschen Farbgemisches, offenbar durch sekundäre Degeneration bedingt, ein umfangreicher Zerfall von Achsenzylindern nachweisen ließ, lagen große Gliazellen in reicher Zahl, solche, die eine Menge Fasern bildeten, und solche, die sich in nichts von echten amöboiden Zellen unterschieden in allen Entwicklungsstadien. Andere Gliazellen aber, gleichfalls mit einem großen lappigen Protoplasmaleib, aber etwas größerem Kern, zeigten einen Zelleib, der an seinem Rande in ein scharf gefärbtes, wohl abgegrenztes Retikulum sich aufteilte, in dessen Maschen Markscheiden, normale und degenerierende Achsenzylinder lagen. Die Textfigur 2 stellt eine solche dar. An einzelnen Zellen schien sich der Zelleib

von dem Retikulum retrahiert zu haben, so daß die Zelle wie in einem selbstgebildeten Neste lag. So fanden sich also in diesem Herd alle Formen der Glia nebeneinander, fasernerzeugende, abbauende und das Retikulum bildende Elemente. Dort, wo mehrere solcher netzebildende Zellen nebeneinander lagen, schienen die einzelnen Netze völlig in einander zu verschmelzen. Solche Netze waren nur um Gliakerne herum sichtbar, in großen dazwischen liegenden Strecken war nichts von einem Retikulum wahrzunehmen. Ich glaube, ähnliche Vorgänge auch bei anderen diffusen Krankheitsprozessen gesehen zu haben. Aber in der Nachbarschaft des Erweichungsherdes erleichterte die Größe der Elemente wesentlich das Verständnis. Bei den diffusen Prozessen dagegen sind alle Strukturen so zart, so blaß gefärbt und unbestimmt, daß man keinen ganz sicheren Boden mehr unter sich fühlt. Darum ist es wohl zweckmäßiger, erst die Methodik noch weiter auszubauen, bevor wir uns in einer eingehenden Darstellung versuchen. Aber schon diese Beobachtungen, wenn sie einstweilen auch nur dürftige Bruchstücke darstellen, ergeben eine außerordentliche Kompliziertheit und Verschiedenartigkeit der gliösen Strukturen bei verschiedenen Krankheitszuständen und damit auch, wie wir immer noch deutlicher sehen werden, eine ungemeine Mannigfaltigkeit in der Art der nervösen Schädigungen, deren Negativ sie ja nur darstellen.

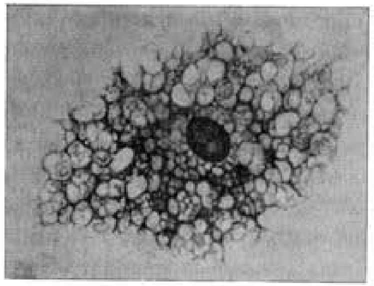

Fig. 2. Gliazelle aus der Nachbarschaft eines etwa 14 Tage alten Blutungsherdes in der weißen Substanz; durch das Retikulum ziehen Achsenzylinder hindurch. Methode V.

Schließlich möchte ich noch einiges mitteilen, über regeneratorische Vorgänge, die sich an den perivaskulären Räumen bemerken lassen. Die Verhältnisse sind ungemein schwierig zu beurteilen, so daß ich mich auf einige Beobachtungen beschränken will, ohne die Rekonstruktion des ganzen Vorgangs zu versuchen. Bei Psychosen, welche in ein chronisches Stadium übergegangen sind, z. B. bei der Dementia praecox, habe ich öfter gesehen, wie sich um das Gefäß herum eigenartige geflechtähnliche Bildungen aus einer Art von amöboiden Zellen entwickelten, die zum Teil im Nervengewebe zum Teil frei im peri-

vaskulären Raume lagen. Auch sah man hier öfter, wie plasmatische Gliafortsätze an Stelle der früheren Bänder den perivaskulären Raum überbrückten und sich an die Adventitia anlegten. So ergab sich der Eindruck, als ob hier wieder engere Beziehungen zwischen der Glia und der Adventitia angeknüpft würden.

An anderen Stellen fand sich im perivaskulären Raum, diesen mehr oder minder ausfüllend, eine stark mit S-Fuchsin gefärbte Masse. Da sich die Adventitia lebhaft grün färbt und auch hier eine grüngefärbte Adventitia deutlich vorhanden und gut abzugrenzen war, dürfte es sich wohl nicht um eine bindegewebige Masse handeln. So scheint sie eher ein gliöses Produkt, vielleicht eine besonders verdickte Membrana perivascularis.

Außerdem sieht man oft in Präparaten, welche von chronischen Fällen stammen, Lichtungen des Gewebes in der Nachbarschaft der Gefäße. Sie dürften wohl Residuen von Zirkulationsstörungen und Lymphstauungen sein, die früher hier stattgefunden hatten. Wahrscheinlich sind auf die gleiche Ursache auch die kernarmen Zonen um die Gefäße zurückzuführen, die wir so oft bei chronisch gewordenen Prozessen finden.

Wie die Membrana superficialis gliae bei einem allgemeineren oder lokalen Zerfall gliöser Strukturen aufgelöst wird und wie dies der Anlaß werden kann, zu einem Hinauswuchern der faserigen Glia über die ursprüngliche Oberflächenschichte, habe ich schon an anderer Stelle besprochen.

F. Die biologische Bedeutung der amöboiden Gliazellen, die Umstände ihres Vorkommens und ihre Beziehungen zum Abbau des Nervengewebes.

Auf die Aufgabe der amöboiden Gliazellen werden zunächst ein Licht werfen können die Umstände ihres Vorkommens. Wir finden amöboide Gliazellen nicht im erwachsenen normalen Gehirn. Dagegen treffen wir ähnliche Zellformen im fötalen Nervensystem, wenn noch ein Aufbau von Nervengewebe stattfindet. Wir begegnen ihnen auch nicht bei abgelaufenen oder stillstehenden Krankheitsprozessen, abgesehen von den in wesentlichen Punkten abweichenden Dauerformen, welche wir vorhin besprochen haben.

So vermissen wir sie in alten encephalitischen Narben, in der Umgebung von abgekapselten Blutungs- und Erweichungsherden, in den meisten Idiotenhirnen, bei alten Fällen von Dementia praecox, wenn nicht etwa in der letzten Zeit neue Krankheitsschübe erfolgt

waren; bei der Epilepsie, wenn durch das längere Ausbleiben von Anfällen oder Äquivalenten ein Stillstand oder nur langsamer Fortschritt der Krankheit angezeigt war; beim Alkoholismus, wenn dem Tode längere Abstinenz vorausging; bei stationären Paralysen. Auch bei manchen Fällen von progressiver Paralyse und seniler Demenz sind sie wenig zahlreich. Bei einer Reihe in schwerster Erregung verstorbenen manisch-depressiven Kranken habe ich sie ganz vermißt, in einigen anderen waren solche einzeln vorhanden, aber das Bild war getrübt durch das Hinzutreten schwerer körperlicher Krankheiten. Wir finden typische amöboide Gliazellen häufig nicht oder in nur mehr untergeordneter Rolle bei einem ausgedehnten Zerfall nervösen Gewebes in encephalitischen Herden, bei Erweichungen und Blutungen, bei Strang-Degenerationen im Rückenmark, unter meningitischen Infiltrationen, in den Herden raschen Unterganges von Markscheiden, wie er nach apoplektiformen Anfällen der Paralyse oftmals nachweisbar ist, also überall dort, wo es zur Bildung massenhafter Körnchenzellen ektodermaler oder mesodermaler Herkunft kommt.

Dagegen treffen wir sie bei schweren Infektionsdelirien, beim Alkoholdelir, in den akuten Stadien der Dementia praecox, in vielen Fällen von Status epilepticus und in manchen epileptischen Dämmerzuständen, bei Paralyse, seniler Demenz, Lues cerebri, bei den drei letzteren in ihrer Häufigkeit offenbar abhängig von dem rascheren oder langsameren Fortschreiten der Krankheit, bei der Chorea progressiva, bei vielen akuten und subakuten experimentellen Intoxikationen, dann weiter in der Nachbarschaft von Tumoren, zuweilen auch neben Körnchenzellen am Rande frischerer Erweichungsherde und Abszesse. Wahre Riesenformen zeigten sich in einem Falle von tuberöser Sklerose. Auch bei schwerer Urämie, beim Coma diabeticum, bei Sepsis und in mancherlei Fällen, welche infolge körperlicher Krankheit vor dem Tode tagelang dauernde Zustände völliger Bewußtlosigkeit gezeigt hatten, waren sie gelegentlich anzutreffen, auch wenn diese Kranken vorher nicht geisteskrank waren oder an psychischen Krankheiten gelitten hatten, bei welchen amöboide Zellen sonst nicht nachzuweisen sind. Schließlich handelt es sich aber doch bei allen diesen Zuständen um schwere infektiöse oder toxische finale Geistesstörungen, wenn sie auch in den Lehrbüchern der Psychiatrie meist nicht eingehender behandelt werden, weil ihnen keine besondere praktische psychiatrische Bedeutung zu-

kommt. Es ist wohl kaum möglich, erschöpfend die Krankheitsformen aufzuführen, bei welchen sie vorkommen, schon weil es darunter manche gibt, die heute noch keinen Namen haben. Aber auch diese Zusammenstellung zeigt schon, daß sie für kein Krankheitsbild charakteristisch sind, sich vielmehr als Begleiterscheinung sehr verschiedenartiger akuter Geistesstörungen und neuer Schübe chronischer Psychosen finden, die zu einer geistigen Schwäche führen oder anatomisch ausgedrückt, mit einem Zerfall von nervösem Gewebe einhergehen. Einzelne Beobachtungen, die Ausnahmen dieser Regel darzustellen scheinen, bedürfen noch genauerer Nachprüfung.

Daß amöboide Gliazellen bei manchen hierher gehörigen Krankheitsfällen von besonders raschem Verlauf gelegentlich auch einmal ganz fehlen oder nur einzeln anzutreffen sind, dürfte wohl damit zusammenhängen, daß bis zu ihrer vollen Ausbildung immerhin einige Zeit vergeht, in der erst andere Gliaelemente zugrunde zu gehen oder durch indirekte Teilung neu gebildet zu werden scheinen. So kann der Tod erfolgen, ehe sie richtig entwickelt sind. Bei manchen Fällen von Status epilepticus, schweren Vergiftungen und Infektionen dürfte dies sicher der Fall sein. Wie schnell amöboide Gliazellen werden und vergehen, läßt sich einigermaßen beim Status epilepticus der genuinen Epilepsie abschätzen. Im Gehirn des genuinen Epileptikers, der keine gehäuften Anfälle oder Dämmerzustände gehabt hat, vermissen wir amöboide Zellen oder finden sie nur ganz vereinzelt. Nach einem 12stündigen Status epilepticus sehen wir sie häufig in üppiger Entfaltung, manchmal aber auch noch wenig zahlreich neben zahlreichen Karyokinesen. In einem Status, der nach 6 Stunden zum Tode geführt hatte, fehlten sie noch ganz, aber Karyokinesen waren in großer Zahl zu sehen. Bei einem Falle, in welchem sich der Status mit einer 6stündigen Unterbrechung 27 Stunden hingezogen hatte, waren schon sehr zahlreiche zerfallene amöboide Zellen mit Methylblaugranula vorhanden, die in dem zuerst erwähnten Falle nur ganz spärlich zu sehen waren. Wenn wir nun auch nicht ausschließen können, ja sogar vereinzelte Fälle, bei welchen schon nach einem ganz kurzen Status stark regressiv veränderte amöboide Gliazellen zu finden waren, dafür sprechen, daß ihre Bildung schon anfangen kann, ehe der erste Anfall des Status epilepticus eintritt, so dürfte es doch in hohem Grade wahrscheinlich sein, daß sie sich zuweilen innerhalb weniger Stunden ausbilden. Dagegen sieht man bei experimentellen Vergiftungen, die in der Art

der Nisslschen subakuten maximalen Vergiftung ausgeführt werden, daß zuweilen eine Reihe von Wochen nötig ist, bis die ersten typischen amöboiden Zellen auftreten. Schon früher lassen sich andersartige Gliaveränderungen nachweisen; es bedarf offenbar einer schwereren Schädigung der nervösen Substanz, bis die amöboide Glia zur Entwicklung gelangt.

Wenn nun auch der Vergleich klinischer und anatomischer Erfahrung dafür spricht, daß die amöboiden Gliazellen als Begleiterscheinung des Zerfalles von nervösem Gewebe auftreten, so bleibt der pathologischen Anatomie doch noch die Aufgabe, die engeren Beziehungen zwischen beiden festzustellen. Vor allem bedarf der Zerfall des nervösen Gewebes des histologischen Nachweises. Das ist aber nun eine Aufgabe, die wir mit unseren heutigen Untersuchungsmethoden noch nicht in wünschenswerter Weise zu lösen vermögen. Trotzdem glaube ich, daß wir mit den hier angewandten Methoden auch einiges mehr über den Untergang nervöser Gewebsbestandteile erkennen können.

Zunächst ist es auffällig, daß man bei vielen Krankheitsvorgängen die meisten und größten amöboiden Zellen im Marke, besonders in der Markleiste der Windungen antrifft. Bisher hat man meist angenommen, daß sich die Krankheitsprozesse, welche Psychosen verursachen, hauptsächlich in der Rinde abspielen, und das Mark nur insofern daran Anteil nimmt, als ein Untergang nervöser Rindenelemente auch einen Ausfall von Markfasern in der Markleiste nach sich ziehen muß. Es ist nun nicht leicht zu sagen, womit das zahlreichere Auftreten amöboider Gliazellen im Mark zu erklären ist. Wenn wir noch gröbere Degenerationsprozesse ins Auge fassen als die, welche uns hier beschäftigen, Degenerationsprozesse, bei welchen die Glia typische Fettkörnchenzellen bildet, wie z. B. häufig bei der Paralyse nach apoplektiformen Anfällen, dann finden wir auch hier im Mark mehr Fettkörnchenzellen als in der Rinde. Vielleicht hängt das damit zusammen, daß das zerfallende Mark mehr fettige Produkte bei seinem Abbau liefert als die Rinde, weil es schon mehr aus lipoiden Stoffen aufgebaut ist. Dann mag aber auch zu dieser reichlicheren Aufstapelung von Abbaustoffen im Mark beitragen, daß, wie jedes Injektionspräparat zeigt, die kapillaren Maschen in der Rinde viel enger sind als im Mark, so daß der Wegtransport dort erleichtert, hier erschwert sein muß, und schließlich erscheint auch der Abbauvorgang in der Rinde etwas abweichend von dem im

Mark zu sein, insofern, als dort Verflüssigungsprozesse an den protoplasmatischen Strukturen überwiegen dürften.

Jedenfalls aber lassen sich in den meisten Fällen, in welchen amöboide Zellen in größerer Menge im Mark nachzuweisen sind, auch Abbauerscheinungen am Nervengewebe selbst, den Achsenzylindern und Markscheiden feststellen. Ein Überblick über ein reiches Untersuchungsmaterial erweckt den Eindruck, daß die Bildung amöboider Gliazellen schon beginnen kann, ehe Zerfallserscheinungen an diesen nachweisbar sind, daß letztere aber selten fehlen, wo sehr zahlreiche und zum Teil schon regressiv veränderte amöboide Zellen im Gewebe liegen. Manchmal allerdings waren auch amöboide Gliazellen in allen Entwicklungsstadien zu finden, ohne daß ein Untergang von Achsenzylindern festgestellt werden konnte. Zum Nachweis degenerierender Achsenzylinder erweist sich die Methode V verwertbar. Bei genauer Befolgung der gegebenen Vorschriften sind die normalen Achsenzylinder blau gefärbt. Es kommt vor, daß eine geringere oder größere Anzahl von Achsenzylindern in einer rötlich-blauen Mischfarbe sich darstellt. Jedenfalls aber zeigen diese rötlich-blauen Achsen, wie die blauen, keinerlei morphologische Veränderungen. Wir dürfen wohl auch sie als normal betrachten. Dagegen leuchten gewisse Achsenzylinder, meist gerade feinere, durch eine leuchtend hellrote Farbe aufs augenfälligste hervor. Diese zeigen dann in ihrer Form und Struktur wesentliche Abweichungen vom Normalen (Textfigur 3). Die normalen Achsen sind von geraden Linien begrenzt und von homogener Struktur, sowie gleichartiger Färbung. Die roten zeigen eine wechselnde Dicke, zuweilen ganz mächtige Auftreibungen, Schlängelungen in ihrem Verlauf und eine körnige Struktur, ja körnigen Zerfall. In struktureller Beziehung erinnern diese Bilder sehr an jene, welche man mit der von Donaggio angegebenen Hämatoxylinfärbung an zerfallenden Markfasern erhält. Wie Vergleichspräparate gezeigt haben, entsprechen sich auch tatsächlich diese und die Bilder Donaggios. Da es sich hier aber immer nur um einzelne und dazu noch besonders feine Fasern handelt, so erscheint die Methode von Donaggio weniger geeignet, sie elektiv darzustellen. Wenn nämlich bei dieser Methode die Entfärbung der gröberen normalen Fasern eingetreten ist, haben sich meist auch diese degenerierenden feinen Fasern völlig entfärbt. Deutlich genug, wenn auch etwas weniger brillant, treten die degenerierenden Fasern auch in Flemming-Schnitten hervor, die nach Photoxylineinbettung 5 Minuten in einer 1%igen Säurefuchsinlösung gefärbt sind. Die normalen Achsenzylinder sind dann matt hellrot, die degenerierten leuchtend dunkler rot gefärbt. Die morphologischen Abweichungen sind dieselben wie in den Mannschen Präparaten.

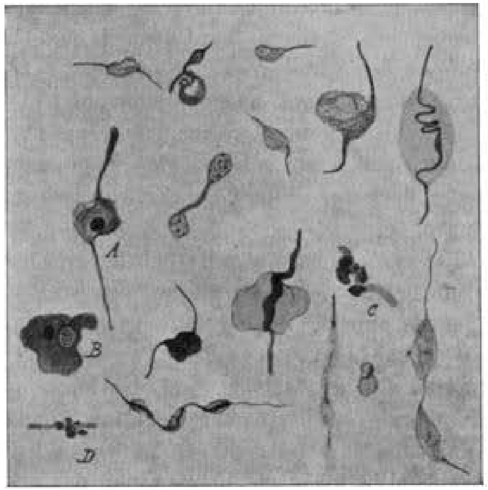

Fig. 3. Feinere degenerierende Achsenzylinder aus der Hirnrinde von Fällen von Dementia praecox und und Status epilepticus dargestellt mit Methode V. *A* eine amöboide Gliazelle liegt in dem aufgetriebenen Achsenzylinder. *B* ein Achsenzylinderrest in einer amöboiden Zelle eingeschlossen. *C* und *D* Markbrocken.

Neben den rot gefärbten Achsenzylindern findet man nach Anwendung der Methode IV gelegentlich noch andere, die pathologisch verändert erscheinen. Die Färbung ist dunkelblau oder violett, viel dunkler als die der normalen Achsen. Dabei zeigt sich der Achsenzylinder oft in Schollen und Körnern von dunkelblauer Farbe aufgelöst (Tafel XXIX, Fig. 20). Im allgemeinen sieht man solche Achsenzylinder seltener. Bei sehr schweren Prozessen können sie aber auch manchmal über die roten überwiegen oder allein vorhanden sein. Es scheint, daß diese roten und diese dunkelblauen Achsenzylinder nicht verschiedene Stadien des Zerfalls einer Faser darstellen, sondern zwei von vornherein abweichenden Degenerationsformen zugehören, da man manchmal nur die einen, manchmal nur die anderen antrifft. Hin und wieder begegnet man dann bei Anwendung der gleichen Methode noch Achsenzylindern, die enorm angeschwollen und dabei ganz blaßblau gefärbt sind. Besonders in der Nachbarschaft von Herden und unter meningitischen Infiltrationen im Rückenmark kann man sie beobachten. Behandelt man Rinden, in denen zahlreiche rote Achsenzylinder mit der Mannschen Methode darstell-

bar sind, nach der Silbermethode von Bielschowsky, so lassen sich auch damit neben glattrandigen und homogen erscheinenden Achsenzylindern unregelmäßig verlaufende, stellenweise aufgequollene, aus körnigen Massen bestehende oder in körniger Auflösung befindliche nachweisen. So finden wir bei der Anwendung sehr verschiedener Methoden, daß die Ausbildung zahlreicher amöboider Gliazellen oft mit einem Zerfall von Achsenzylindern einhergeht.

Viel schwieriger bleibt der Nachweis degenerativer Vorgänge an den Markscheiden des zentralen Nervensystems, weil bei der Neigung des Markes, rasch postmortale Veränderungen einzugehen, und bei der Schwierigkeit der Fixation und Leichtigkeit der Extraktion von Markbestandteilen die Entstehung von Kunstprodukten ungemein begünstigt wird. Man muß also mit größter Vorsicht in der Auswahl und Behandlung des Materials und unter peinlicher Kritik der erhobenen Befunde vorgehen, wenn man hier nicht durch Kunstprodukte zu falschen Urteilen veranlaßt werden soll.

Bei der Anwendung der Methode IV nehmen Teile der Markscheide des zentralen Nerven eine rote Farbe an. Dazwischen sehen wir nun zuweilen Haufen scharf abgetrennter, großer, sattroter Schollen in der Markleiste auftreten; sie sind von unregelmäßiger Form und liegen zumeist ringförmig um einen Achsenzylinder angeordnet, der selbst keine wesentlichen pathologischen Veränderungen erkennen läßt (Textfigur 3 *C, D*).

Auch mit der Marchi-Methode lassen sich, wenn man einige Vorsicht nicht außer acht läßt, pathologische Veränderungen der Markscheiden erkennen. Zunächst darf man aber für solche feinen histologischen Untersuchungen kein Material verwenden, das vorher in Formol gelegen hat; denn je länger Gehirnstücke in Formol eingelegt waren, desto leichter und zahlreicher treten gebräunte und geschwärzte Produkte im Gewebe auf, wenn man es später mit Osmium nach vorausgegangener Chromierung behandelt. Sie sind zwar nach ihrer Lage und Form von pathologischen Markscheidenprodukten abweichend, erschweren aber sehr die Erkennung der letzteren und machen sie bei einer größeren Anhäufung ganz unmöglich. Auch in nicht ganz frisch eingelegtem, durch Fingerdruck beschädigtem oder mit Wasser überspültem Material bilden sich leicht bei der Marchi-Behandlung Kunstprodukte in Form gebräunter oder geschwärzter Schollen. Dagegen findet man in frisch der Leiche entnommenen, direkt in Müllerscher Lösung

gebrachten und dann nach Marchi behandelten Rindenstückchen, dort, wo zahlreiche amöboide Gliazellen zu beobachten sind, oft vereinzelten Markzerfall, der an den Markzerfall bei der Wallerschen Degeneration erinnert, in der Form zu Reihen geordneter, schwarzbrauner Markklumpen, häufiger noch eine geringe, manchmal aber auch sehr erhebliche Anhäufung von gebräunten und geschwärzten kugeligen Gebilden, die den von Elsholz beschriebenen Körperchen des peripheren Nerven entsprechen dürften. An anderen Markscheiden wieder heben sich aus der sonst nicht geschwärzten Scheide einzelne oder Anhäufungen dunkler, gefärbter Schollen hervor, Bilder, wie man sie bei den leichteren Graden des diskontinuierlichen Zerfalls der peripheren Nervenfasern (bei toxischer und infektiöser Neuritis) häufig findet.

Eine andere Erscheinung, die Beachtung verdient, wenn sie auch noch nicht genügend geklärt ist, besteht in dem Auftreten eigentümlicher, bei der S-Fuchsinlichtgrünfärbung grün gefärbter, oft ösenförmiger oder wabiger Gebilde in der Markscheide. Da diese sehr zahlreich dort zu finden sind, wo reichlich Mark zerfällt, z. B. in der Nachbarschaft von Herden, vereinzelter überall, wo amöboide Zellen vorkommen, sehr selten oder nie im normalen Gehirn, dürfte ihnen gleichfalls eine pathologische Bedeutung zukommen.

Zu beachten ist bei diesen Untersuchungen allerdings, daß man kaum ein menschliches Gehirn antrifft, in welchem nicht, wenn auch nur in spärlicher Zahl, vereinzelte geschwärzte Körperchen und gebräunte Schollen im Marklager zu finden sind. Es ist wahrscheinlich, daß sie mit Abbauvorgängen zusammenhängen, die in die Breite des Normalen gehören. Weiter muß man sich hüten, zwischen den Markfasern gelegene, in Gliazellen eingeschlossene fettige Stoffe mit eigentlichen Markscheidenzerfallsprodukten zu verwechseln. Die schon geschilderten cystchenartigen Bildungen und die häufchenartige Anordnung der lipoiden Stoffe deuten darauf hin, daß sie in Gliazellen eingelagert sind. Um Verwechslungen mit ihnen zu vermeiden, ist es nötig, dünne Schnitte anzufertigen und mit der Marchi-Behandlung noch eine Schnittfärbung mit Saffranin, S-Fuchsinlichtgrün usw. zu verbinden. Erst dann kann man Achsenzylinder, Zellkern und Zellplasma und damit auch die Lageverhältnisse der geschwärzten und gebräunten Stoffe hinreichend erkennen. Andeutungen, die noch weiter verfolgt werden müssen, sprechen dafür, daß noch andere pathologische Veränderungen an den Markscheiden vorkommen;

jedenfalls beweisen schon die beschriebenen genügend, daß mit dem Auftreten amöboider Gliazellen im Mark sehr oft Degenerationserscheinungen am Nervengewebe einhergehen.

Wie steht aber nun das Auftreten amöboider Gliazellen zu dem Untergang dieser nervösen Gewebsbestandteile in Beziehung?

Eine raumausfüllende Aufgabe der Glia kann dabei sicher nicht ausschließlich in Frage kommen, denn die amöboiden Gliazellen treten, wie wir schon gesehen haben, auf, ehe Nervensubstanz zugrunde gegangen ist. Weiter übertreffen sie in ihrer Masse oft sehr erheblich die Masse des zerfallenden Nervengewebes. Schließlich spricht ihre kurze Lebensdauer und ihre Neigung zu raschem Untergang gegen eine derartige biologische Aufgabe. Sie müssen eine andere Bestimmung haben.

Wenn man nun sieht, wie die amöboiden Gliazellen die Markscheiden umfließen, bis sie dieselben schließlich ganz umfassen, kann die Vorstellung erweckt werden, daß sie an einer Zerstörung der nervösen Elemente mitarbeiten, also eine neurophagische Tätigkeit entwickeln. Die letzten Jahre haben uns eine recht reichliche Literatur über die Neurophagie gebracht, und wir finden darin verschiedene Meinungen vertreten über die Art und Herkunft der Zellen, welche neurophagische Tätigkeiten entwickeln, und darüber, worin diese neurophagische Tätigkeit besteht, ob die Neurophagen die nervösen Zellen nur auflösen oder Teile derselben in sich aufnehmen, ob sie lebende Zellen angreifen oder nur bereits abgestorbene zerstören.

Bei den Veränderungen, die wir jetzt besprechen, können nur Neurophagen gliöser Herkunft in Frage kommen. Auch handelt es sich zunächst nicht um eine neurophagische Tätigkeit den Ganglienzellen, sondern den Markscheiden und Achsenzylindern gegenüber. Daß Gliazellen fremdartige Stoffe in sich aufnehmen, ist ja längst bekannt. Sie beladen sich in der Nachbarschaft alter Blutungsherde mit Blutpigment, sie sammeln Tusche, Karmin, Zinnober, die man ins Nervengewebe eingeführt hat, in ihrem Zelleib an, bis sie zu kugeligen, mit Fremdstoffen voll beladenen Gebilden geworden sind.

Nun müßte die Beobachtung, daß Gliazellen Markscheiden mit ihrem protoplasmatischen Zelleibe umfließen, noch nicht notwendigerweise den ersten Akt einer neurophagischen Tätigkeit darstellen. Wenn Gliazellen, die zwischen Markfasern liegen, sich vergrößern, können sie das nur in die engen Räume zwischen die Markscheiden

hinein, und so müßte schon eine einfache Vergrößerung des Zellleibes solche Bilder zustande bringen.

Schwerere Degenerationsprozesse als die, welche wir hier vor uns haben, bei welchen Fettkörnchenzellen gebildet werden, zeigen mit einer Eindeutigkeit, die kaum anzuzweifeln ist, daß das Fett, welches sich in den gliogenen Körnchenzellen ansammelt, aus den Markscheiden und Markscheidenprodukten herstammt, daß also Gliazellen Markscheiden abbauen. Sehr deutlich läßt sich der Hergang verfolgen in frischen Degenerationsherden, z. B. im Rückenmark bei der tuberkulösen Meningomyelitis, von der die Fig. 6, Tafel XXXIV, stammt, mit Hilfe der Marchi- und der Herxheimerschen Methode. Die Marchi-Methode zeigt hier sehr viele gebräunte und geschwärzte Markschollen. Herxheimersche Präparate lassen erkennen, daß zunächst nicht wenige kleine Gliazellen in die Markscheide selbst hineingelangen und dort in ihrem Plasma feinere und gröbere Fettkörnchen aufspeichern (*f*, *g*), um, wie es scheint, rasch wieder zugrunde zu gehen, nachdem der Kern eine homogene Färbung angenommen und schließlich unfärbbar geworden ist. Man könnte solche Elemente wegen ihrer geringen Größe und ihres rundlichen Zelleibes für Blutelemente halten. Kontrollpräparate aber zeigen, daß an diesen Stellen gar keine Auswanderung von Blutelementen stattgefunden hat. Das sind aber wohl nur kleine Vorgefechte gewesen. Der eigentliche Abbauvorgang beginnt erst damit, daß sich in größeren, zwischen den Markscheiden gelegenen Gliazellen immer zahlreichere Fettropfen anhäufen (*a*, *d*, *e*, *c*), bis richtige Fettkörnchenkugeln (*b*) aus ihnen geworden sind. Nimmt man wieder Marchi-Präparate zur Hilfe, so findet man, daß fast alle diese Fettkörnchen anhäufende Gliazellen Markschollen und Markbrocken, welche die Herxheimersche Methode nicht rötet, umflossen haben, und ein Vergleich vieler solcher Bilder beseitigt jeden Zweifel, daß mit der Zunahme der Fettkörnchen im Zellleib der Gliazellen die von ihnen umschlossenen Markbrocken kleiner und heller werden, bis sie verschwinden. Jedenfalls also wandeln hier Gliaelemente die verschiedenartigen Stoffe, aus welchen die Markscheiden aufgebaut waren, und die sich mit dem Zerfall der Markscheiden in Brocken (Markschollen) zusammengeballt hatten, in Fett um. Denn die Markschollen, welche sich wohl nach Chromierung mit Osmium bräunten, aber im Formolgefrierschnitt nicht mit Scharlach röteten und durch diese Reaktion zeigten, daß sie

schon umgewandelte, aber nicht in Fett umgewandelte Stoffe darstellen, erscheinen jetzt in der Zelle selbst in einer anderen Form, in Körnchen, die sich mit Osmium schwärzen und mit Scharlach röten. Dabei finden wir keinerlei Anhaltspunkte dafür, daß die Gliazellen ihre Abbautätigkeit an normalen Markscheiden beginnen und sie schädigen. Das spricht dafür, daß diese Zellen abräumen, was durch den Krankheitsprozeß schon geschädigt und dem Untergang bereits verfallen ist. Die Gliazellen vergrößern sich sicher nicht deswegen, weil durch den Zerfall der Nervenfasern Platz geworden ist, denn die zerfallende Nervenfaser nimmt eher mehr denn weniger Platz ein als die gesunde. Sie müssen sich also wohl vergrößern auf einen Reiz hin, den das pathologische Markscheidenprodukt auf sie ausübt. Wir sehen dabei gelegentlich, daß ein einzelner Markbrocken ganz von der Gliazelle umflossen ist, also gleichsam in einer Gliazelle liegt. Wir finden aber nicht etwa, daß diese kleine Partikel davon in ihr Plasma aufnehmen. Sie scheinen im wesentlichen auflösend auf diese Produkte zu wirken, die Stoffe, welche bei der Auflösung frei werden, sich zu assimilieren und in ihrem Körper in fettige Stoffe umzuwandeln. Denn nirgends finden wir in einem solchen Gewebe freies Fett. Fett entsteht erst in der Gliazelle. Die manchmal vollständig übereinstimmende Größe der Fettkörnchen einer einzelnen Zelle beweist auch ihre intrazelluläre Entstehung. Der Abbau geht schließlich weiter, indem die fettigen Stoffe aus dem Nervengewebe heraus und in die adventitiellen Lymphbahnen überwandern. So ist also bei dieser besonderen Form der Degeneration des zentralen Nervengewebes die Tätigkeit der Gliazellen gut zu verfolgen, und das kann uns auch Licht auf die uns beschäftigenden Erkrankungszustände des Nervengewebes werfen, bei welchen die amöboiden Gliazellen eine Rolle spielen.

Auch dort, wo nicht Fettkörnchenzellen, sondern amöboide Gliazellen das histologische Bild beherrschen, finden wir hin und wieder einmal die leuchtend rot gefärbten Reste eines Achsenzylinders in einem Hohlraum einer blau gefärbten amöboiden Zelle (Textfigur 3 *B*); Bilder aber, die darauf hindeuten, daß die amöboiden Gliazellen Teile zerfallenden Nervengewebes völlig in sich aufnehmen, sind recht selten. Häufiger schon sieht man, daß sich amöboide Gliazellen an pathologisch veränderte Markfasern und Achsenzylinder anlegen (Textfigur 3 *A*). Recht oft findet man auch gerade da, wo eine Nervenfaser zerfällt, keine amöboiden Zellen, oder wo amöboide Zellen in großer Menge beisammen liegen, keine Reste zerfallender Fasern. Daraus dürfte sich jedenfalls nicht ergeben, daß die amöboiden Gliazellen lebende und gesunde Markfasern angreifen. Sie mögen aber vielleicht durch Stoffe, die sie abscheiden, mit zur Verflüssigung des untergehenden Nervenmaterials beitragen, und Produkte, die durch Verflüssigung nervösen Gewebes frei geworden und in die das Gewebe durchtränkende Lymphflüssigkeit gelangt sind, sich assimilieren, um sie in ihrem Körper weiter abzubauen. Vielleicht mögen Stoffe, die bei dem Zerfall des Nervengewebes entstehen, die Bildung amöboider Gliazellen anregen.

Dieser Erklärung dürften sich aber die selteneren Fälle nicht einordnen lassen, bei welchen sich amöboide Gliazellen ohne gleichzeitige Erscheinungen von Nervenzerfall finden. Vielleicht gehen auch hier nervöse Strukturen zugrunde, die wir nur noch nicht darstellen können, vielleicht entstehen hier pathologische Stoffwechselprodukte, die sich dem mikroskopischen Nachweis einstweilen entziehen, nicht durch den Zerfall nervöser Elemente, sondern durch Störungen in ihrer Ernährung, und sie helfen mit zu ihrer Beseitigung. Ich habe viel Zeit auf die weitere Klärung dieser Frage verwendet, ohne bis jetzt zu einem sicheren Ergebnis gelangt zu sein.

Jedenfalls sind dann die Granula, die wir im Plasma der amöboiden Gliazelle während ihres absteigenden Lebensganges auftreten sehen, nicht von den Zellen aufgenommen, sondern von ihnen gebildet. Die ersten Formen von Granula, die wir leicht nachweisen können, sind die fuchsinophilen. In besonders großen amöboiden Zellen, so z. B. bei der hypertrophischen Sklerose, haben sich neben den fuchsinophilen auch mit Lichtgrün gefärbte Granula darstellen lassen. Meine Erfahrungen über sie sind aber nicht genügend, um vieles darüber aussagen zu können. Nicht zu verwechseln mit den Lichtgrüngranula sind gewisse lipoide Stoffe, die neben einer leichten Bräunung durch das Osmium auch noch etwas Lichtgrün festhalten. Die echten Lichtgrüngranula sind von der Größe und Form der fuchsinophilen, man sieht meist in einer Zelle nur wenige. Sie scheinen keine lipoiden Stoffe darzustellen.

Die fuchsinophilen Granula gehören zur amöboiden Gliazelle und finden sich so regelmäßig in ihr, daß sie wohl mit der Lebenstätigkeit der Zelle in enge Beziehungen gebracht werden müssen. Sie sind schon in jugendlichen Zellen vorhanden, und zwar eher, als gebräunte oder geschwärzte Stoffe in ihnen auftreten. Im Mark

gibt es normalerweise nur sehr spärliche und anders geformte fuchsinophile Körperchen. Es kommen davon eigentlich nur in Betracht die mehr stäbchenförmigen fuchsinophilen Körperchen des Achsenzylinders und sehr kleine Körnchen, die im Plasma der Gliazellen, vielleicht auch im Gliareticulum liegen. Große runde Granula, wie die der amöboiden Zellen, sind dem normalen Marke fremd. Sie finden sich auch pathologischerweise nicht in dieser Art außerhalb der Zellen. Damit dürfte sichergestellt sein, daß sie in den Zellen gebildet werden.

Über ihre chemische Beschaffenheit gibt uns ihr färberisches Verhalten wenig Aufschluß. Wir sehen, daß in den Präparaten, in welchen sie dargestellt sind, vielerlei ganz verschiedene Dinge die gleiche rote Farbe zeigen, rote Blutkörperchen, Gliafasern, andere normalerweise vorhandene Granula, die zum perizellulären Apparat der Ganglienzelle und zu ihr selbst gehören. Auch bei der oben angegebenen Hämatoxylinfärbung färben sich die Granula der amöboiden Zellen wie die normalerweise vorhandenen, ebenso mit der von Held angegebenen Methode. Nun wissen wir aber seit den Arbeiten Altmanns und seiner Schüler, daß fuchsinophile Granula in sehr vielen Körperzellen vorkommen und Heidenhains neuere zusammenfassende Darstellung zeigt uns, daß ihre Bedeutung noch viel umstritten ist.

Mancherlei Beobachtungen an unseren Präparaten sprechen dafür, daß wir in der fuchsinophilen Granula der amöboiden Gliazellen Präprodukte lipoider Stoffe zu sehen haben. Wir finden nämlich meist dort, wo mit Osmium gebräunte und geschwärzte Substanzen auftreten, auch fuchsinophile Granula angehäuft und sehen darunter nicht selten einzelne, die schon braun gefärbt sind, aber doch daneben noch einen roten Ton angenommen haben. Dabei kann bald die bräunliche, bald die rote Farbe die ausgesprochenere sein. Die großen lipoiden Cystchen scheinen ganz regelmäßig aus kleinen bräunlichen Körnchen hervorzugehen, die anfangs wenig die Größe der fuchsinophilen Granula überschreiten. So dürften wir hier den Übergang von fuchsinophilen Zellgranula in lipoide Stoffe verfolgen können.

Jedenfalls ist ebenso sicher, daß auch das Fett nicht von den amöboiden Gliazellen in der Form, in der wir es darin finden, aufgenommen wird. Es ist eine sehr bemerkenswerte Tatsache, daß wir fast unter keinen Umständen im Nervengewebe, wenn es nicht

ganz vollständig zerstört ist, mit Scharlach färbbare lipoide Stoffe außerhalb der Zellen antreffen. Sie liegen nahezu alle innerhalb der Zellen und auch dort, wo sie außerhalb zu liegen scheinen, macht es eine genauere Untersuchung wahrscheinlich, daß sie in Zellfortsätzen gelegen sind, die durch die Schnittführung von der Zelle abgetrennt wurden. Ob das auch für die mit Osmium gebräunten oder geschwärzten Stoffe zutrifft, namentlich für die beim Zerfall der Markscheiden entstehenden Schollen, die sich mit Scharlach nicht färben, ist weniger sicher, da sich im einzelnen Falle oft schwer entscheiden läßt, ob eine Scholle noch dem Markscheidenverbande angehört oder frei liegt. In Präparaten vom Status epilepticus habe ich öfter amöboide Zellen mit Fettcystchen an Stellen gesehen, wo gar keine gebräunten und geschwärzten Schollen im Mark zu finden waren. So müssen wir zur Annahme kommen, daß auch die lipoiden Stoffe der amöboiden Zellen durch die Tätigkeit der Zelle selbst gebildet worden sind durch Umwandlung der fuchsinophilen Granula. Da diese Stoffe schließlich in erheblicher Menge sich in der Zelle ansammeln, ja den Plasmaleib nahezu völlig ersetzen, kann in der Gliazelle eine erhebliche Aufstapelung lipoider Stoffe stattfinden. Sie können dort länger aufgespeichert liegen bleiben oder auch allmählich wieder abgegeben werden.

Wie wir schon gesehen haben, hat es aber den Anschein, als ob die Bildung von fuchsinophilen Granula und Fettcystchen noch eine mildere Form des Abbauvorganges darstellt. Stürmischere degenerative Prozesse dürften durch die Bildung der mit Methylblau und Phosphormolybdänhämatoxylin stark färbbaren Körnchen im Zellplasma gekennzeichnet sein. Denn während das Zellprotoplasma bei der Bildung der fuchsinophilen Granula und Fettcystchen immer noch teilweise erhalten bleibt, und auch der Zellkern oft keine, jedenfalls aber erst in späteren Stadien regressive Veränderungen aufweist, sehen wir, daß das Auftreten der Methylblaugranula mit einer Auflösung von Zelleib und Kern einhergeht.

Was nun diese in außerordentlich kurzer Zeit massenhaft sich bildenden Methylblaugranula für Stoffe sind, wissen wir wieder nicht. Sie scheinen weder in Ganglienzellen noch in den mesodermalen Zellen der Hirnrinde vorzukommen und sind weder mit den fuchsinophilen Granula verwandt, noch mit den uns bekannten lipoiden Stoffen oder den färbbaren Fettsäuren. Daß auch sie nicht von den Zellen aufgenommen, sondern erst in ihnen gebildet werden, dürfte

wieder daraus hervorgehen, daß sie in der einzelnen Zelle gleich groß, in verschiedenen Zellen aber von recht verschiedener Größe zu sein pflegen. In Zellen, in welchen Methylblaugranula auftreten, sehen wir gewöhnlich auch einige kleine, gebräunte oder geschwärzte Körnchen, auch manchmal einige dürftige fuchsinophile Granula. Aber diese sind offenbar weder Vorstufen noch Umwandlungsprodukte jener. Schließlich färben sich die Methylblaugranula blasser, während sie etwas an Größe zunehmen, und endlich sind sie nicht mehr färbbar. Sie scheinen sich unter Aufquellung mit den Resten der Zelle zu verflüssigen. Ganz ähnlich wie die Methylblaugranula scheinen sich die fibrinoiden Granula zu verhalten, über deren chemische Natur wir ebensowenig wissen.

Jedenfalls zeigen sich aber überall dort, wo Methylblaugranula n Menge im Gewebe und um die Gefäße herum auftreten, gleichgefärbte Massen, Gliazellen darunter, in den perivaskulären Räumen. Wir haben schon oben Beobachtungen dafür mitgeteilt, daß diese perivaskulären Räume keine Schrumpfräume sind und wohl vielfach dadurch gebildet werden, daß die der Membrana limitans anliegenden amöboiden Zellen mit dieser zerfallen, nachdem ihr Plasma in der Bildung solcher Granula aufgebraucht worden ist.

Zu einem klaren Verständnis der Vorgänge, die sich um die Gefäße herum abspielen, wäre es wichtig zu wissen, inwieweit den Gliazellen die Fähigkeit zukommt, zu wandern. Von vielen Autoren wird es als feststehend angesehen, daß sie ihren Ort verlassen können. Bei einzelnen Formen der Gliazellen dürfte dies ihre ganz verwickelte Gestaltung unmöglich machen. Bei den rundlichen amöboiden Formen würde die Gestalt schon einen Wechsel der Lage ermöglichen. Doch ist die Entscheidung darüber recht schwierig, und selbst dann nicht, wie man erwarten sollte, eindeutig zu treffen, wenn man die Verschleppung von Stoffen verfolgt, die künstlich in das Zentralnervensystem eingeführt worden sind, weil sich nicht sicher entscheiden läßt, ob nicht etwa der Lymphstrom weit abliegenden Gliazellen Partikelchen zugeführt hat. Auch können sich die Gliazellen offenbar ungemein rasch an einem Orte vermehren, so daß große Anhäufungen derselben nicht aus einer Zuwanderung von Zellen erklärt werden müssen. Schon oben aber ist erwähnt worden, daß man Zellen, die als Gliazellen zu deuten sind, innerhalb degenerierender Markscheiden findet. Sie müssen wohl da hin-

eingewandert sein. Ebenso scheinen einzelne amöboide Gliazellen aus dem Nervengewebe in die perivaskulären Räume überzutreten.

Durch die Lücken der Membrana perivascularis können dann noch andere geformte Stoffe in die perivaskulären Räume hineingelangen, so z. B. die Füllkörperchen. Manche der Produkte, die wir darin finden, mögen auch in gelöster Form die noch unversehrte Grenzhaut passiert haben und erst jenseits derselben durch unsere Fixierungsmittel niedergeschlagen worden sein. Wir haben oben gesehen, daß die Masse aller dieser Produkte, die sich in den perivaskulären Lymphräumen ablagern, eine außerordentliche große sein kann. Wo sie besonders reichlich sich finden, sehen wir meist den perivaskulären Raum erweitert, auch bei Anwendung von Fixierungsmitteln, bei welchen das Gewebe wenig schrumpft. Ob das damit zusammenhängt, daß das Gewebe der Nachbarschaft ödematöser ist und daher trotz vorsichtiger Fixierung stärker sich zusammenzieht, oder damit, daß sich in diesen mit pathologischen Produkten angefüllten perivaskulären Räumen die Lymphe anstaut, dürfte schwer zu entscheiden sein. Histologisch läßt sich nicht mit wünschenswerter Sicherheit feststellen, inwieweit im zentralen Nervengewebe Anstauungen der Lymphflüssigkeit vorkommen, weil wir immer an artefizielle Schrumpfräume denken müssen, wo wir Lücken im Gewebe antreffen. Daß es aber Lymphstauungen gibt, ist ganz unzweifelhaft. Gelegentlich sehen wir die adventitiellen Lymphräume ganz enorm erweitert und von einem maschigen Bindegewebe mit eingelagerten Abräumzellen durchzogen, gelegentlich sehen wir auch einzelne solcher Zellen außerhalb der adventitiellen Scheide im perivaskulären Raum. Auch in dem um die Gefäße herum gelegenen Nervengewebe zeigen sich, wie man dies besonders leicht an dicken, diffus gefärbten Schnitten beobachten kann, Lichtungen und Lücken, die man bei ihrer Anordnung, welche den Verlauf des Gefäßes berücksichtigt, und bei den Besonderheiten des sie umgebenden Gewebes unmöglich als artefizielle Schrumpfräume deuten kann.

Offenbar erfahren dann alle diese Produkte des perivaskulären Raumes eine Verflüssigung, soweit sie nicht überhaupt schon in einem flüssigen oder halbflüssigen Zustande in ihn gelangt waren. Klumpen, die zur Hälfte noch im Nervengewebe stecken, zur Hälfte im perivaskulären Raum liegen, sind dort meist dunkel gefärbt und anscheinend fester gefügt als hier. Mit ihrer Auflösung muß die Lymphe eine pathologische Zusammensetzung erhalten. So erklärt

sich wohl, daß wir manchmal den ganzen Inhalt des perivaskulären Raumes mit Massen ausgefüllt finden, welche in ihren färberischen Reaktionen von den Gerinnungsprodukten normaler Lymphflüssigkeit abweichen, und daß je nach der angewandten Fixierungsflüssigkeit oder auch nach der Art der krankhaften Gewebsveränderungen anders geformte und anders sich färbende Stoffe aus ihr ausgefällt werden.

Nun finden wir fast niemals im perivaskulären Raum mit Scharlach färbbare Produkte. Um so reichlicher trifft man sie aber dort, wo die geschilderten Stoffe angehäuft im perivaskulären Raume liegen, in den Zellen der Adventitia und des adventitiellen Lymphraumes, sowie in den Zellen der Pia. Das muß uns bei der zuweilen ganz enormen Anhäufung fettiger Stoffe im mesodermalen Gewebe den Gedanken nahe legen, daß die mesodermalen Zellen rasch aus der durch pathologische Beimengungen verunreinigten Gewebsflüssigkeit Stoffe aufnehmen, um sie schließlich als fettige Produkte aufzustapeln. Erst allmählich dürften sie dann von diesen wieder abgegeben werden. So würde also der ganze Vorgang in der Weise verlaufen, daß bei einem Zerfall von Markscheiden und Achsenzylindern im Mark zunächst amöboide Zellen die Zerfallsprodukte, die wohl meist im Gewebe verflüssigt waren, sich assimilieren und in fuchsinophile Granula und lipoide Stoffe verwandeln. Bei rascherem und stürmischerem Untergang der nervösen Elemente verfallen die Gliazellen selbst wieder, unter Bildung verschiedenartiger Körnchen, besonders der Methylblaugranula. Diese Zerfallsprodukte werden schließlich im Gewebe und wohl hauptsächlich in perivaskulären Räumen verflüssigt, um von den mesodermalen Zellen aufgenommen und in fettige Stoffe umgewandelt zu werden. Es muß dabei unentschieden bleiben, ob diese Stoffe nur aus den perivaskulären Räumen entnommen oder auch in die adventitiellen Lymphräume gelangen und erst von dort aus von Zellen aufgenommen werden. Vielleicht geschieht dies auf beiden Wegen. Auch wie in die Zellen der Pia die Stoffe gelangen, ob sie ihr einfach durch die Lymphräume zugeführt werden, oder ob sich die perivaskulären Räume bis in die Pia öffnen, scheint noch unentschieden.

Die biologische Bedeutung der amöboiden Gliazelle dürfte also darin zu sehen sein, daß sie rasch die Abbauprodukte des Nervengewebes vorläufig beseitigen oder wenigstens in andere Stoffe umwandeln, die dann später nach einer neuerlichen Verflüssigung in die mesodermalen Zellen gelangen, wo sie deponiert werden. Da wir den Vorgang nicht von Anfang bis zu Ende im Mikroskop verfolgen können, weil sich offenbar Stadien dazwischen schieben, in welchen die Abbaustoffe verflüssigt und uns nur sehr teilweise darstellbar sind, fehlt dieser Annahme eine so zwingende Begründung, wie sie einer lückenlosen Serie zukommt. Wir werden aber sehen, daß sich noch einige Lücken etwas ausfüllen lassen durch Beobachtungen, die in der grauen Substanz zu machen sind, und aus der Analogie mit Abbauerscheinungen anderer Art, die wir später kennen lernen werden.

Die Abbauerscheinungen in der grauen Substanz zeigen sich in einer so mannigfaltigen Art, daß es eines eigenen Buches bedürfte, um sie erschöpfend darzustellen. Das ist aber auch nicht die Aufgabe, die hier gelöst werden soll. Hier handelt es sich zunächst nur darum, die Beziehungen gewisser Gliaformen zu den Abbauvorgängen im Nervensystem festzustellen.· Dazu dürften einige Beispiele genügen.

Eine der augenfälligsten degenerativen Veränderungen der Ganglienzellen hat Nissl zuerst als schwere Zellerkrankung genauer beschrieben. Wie bei fast allen Ganglienzellenerkrankungen kommt auch sie in verschiedenen Variationen und zuweilen mit anderen Veränderungen der Zelle vergesellschaftet vor. Gekennzeichnet ist die reine Form im Nissl-Präparat besonders durch zwei Merkmale: 1. durch eine schwere Veränderung des Zellkerns, der runder, kleiner wird und sich in einem metachromatischen Tone dunkler färbt; dabei hebt sich oft die Kernmembran ganz oder teilweise von dem zusammengeballten Kerninhalt ab: 2. durch eine eigenartige Umwandlung des Protoplasmas, das, zumeist von der Zone um den Kern herum beginnend, weniger die basische Farbe annimmt und sich dann in eigenartige kleine Körnchen oder ringartige Gebilde auflöst. Mit dem Zelleib zerfallen auch die Dendriten und der Achsenzylinder, nachdem sie sich vorher mit den basischen Anilinfarben oft in einer eigenartig schmutzigen Farbe gefärbt haben. Der Achsenzylinderursprungskegel erhält sich gewöhnlich am längsten.

Man findet diese sehr typische Zellerkrankung bei mancherlei psychischen Störungen, die unter besonders schweren Symptomen verlaufen waren. Ich habe sie bei galoppierenden Paralysen, akuter Pellagra, perniziösen Malariaformen, schweren septischen Delirien und eigenartigen, tötlich verlaufenden Psychosen der Rückbildungs-

jahre eingehender studiert. Im NISSL-Präparat sieht man meist daneben zahlreiche besonders kleine und dunkel gefärbte Gliakerne, häufig mit Veränderungen, wie wir sie oben als regressive Kernveränderungen amöboider Zellen beschrieben haben. In Tafel XXXIII, Fig. 8—11 und 14 sind solche Kernformen abgebildet. Färben wir die Alkoholschnitte in der oben angegebenen Weise mit Giemsalösung, so sehen wir, daß zu den kleinen Gliakernen verhältnismäßig große, rundliche Protoplasmaleiber gehören (Tafel XXIX, Fig. 7, 10, Tafel XXX, Fig. 4, Tafel XXXIII, Fig. 7). Neben NISSLS schwerer Ganglienzellenveränderung findet sich also eine für die Rinde recht gewöhnliche Form amöboider Gliazellen. Nach Anwendung der Methode V lassen sich in der Markleiste und auch in der Rinde selbst häufig zahlreiche zerfallende Achsenzylinder nachweisen.

Betrachten wir die Art des Zellzerfalls etwas genauer, so finden wir, daß die eigenartigen ringförmigen Körperchen, in welche das Plasma schließlich zerfällt, sich in färberischer Beziehung sehr verschieden verhalten. Einige färben sich mit basischen Anilinfarben deutlich, manche zunächst sogar sehr dunkel, die meisten aber nur blaß, andere sind ganz ungefärbt, manche nehmen bei der WEIGERTschen Gliamethode und auch bei der Fibrinfärbung einen leicht blauen Farbton an, die meisten aber nicht. Eine größere Anzahl wird nach FLEMMING-Fixierung bei der Säurefuchsin-Lichtgrünfärbung rot, dazwischen treten spärliche, mit Osmium gebräunte oder geschwärzte Körnchen hervor.

Aus diesen färberischen Reaktionen können wir nichts auf die chemische Natur dieser ringförmigen Körnchen schließen; wir müssen aber annehmen, daß sie rasche Umwandlungen erfahren und vollständig aufgelöst werden, ehe sie, wenigstens in ihrer Mehrzahl, in lipoide Stoffe umgewandelt worden sind.

Gerade diese eigenartige Zelldegeneration scheint nun in einer ganz ungemein stürmischen Weise die Bildung amöboider Gliazellen anzuregen. Hauptsächlich werden Gliazellen mit rundlichem Plasmakörper gebildet. Dabei entstehen die schönsten Bilder sog. Neurophagie. Von einer raumausfüllenden Aufgabe der Glia kann hier wieder keinesfalls gesprochen werden, denn zu einem Zeitpunkte der Erkrankung, in welchem die Nervenzellen noch eher geschwellt als verkleinert und kaum schon in erheblicher Anzahl zugrunde gegangen sind, sammeln sie sich oft in großer Anzahl um diese herum, bohren

sich in das Ganglienzellenprotoplasma hinein, schieben den Ganglienzellkern vor sich her und verunstalten ihn. Auch an den Protoplasmafortsätzen, weitab vom Ganglienzelleib, und an den degenerierenden Achsenzylindern spielen sich ähnliche Vorgänge ab. Dabei nehmen wir wieder nichts davon wahr, daß die Gliazellen Trümmer untergehender nervöser Gewebsbestandteile in sich aufnehmen. Dagegen sprechen mancherlei Beobachtungen dafür, daß sie eine verflüssigende Wirkung auf das Plasma der kranken und zerfallenden Ganglienzelle ausüben. So sieht man oft, wie in Tafel XXXIII, Fig. 11, eine amöboide Zelle in einer viel größeren aufgehellten Einbuchtung der Ganglienzelle liegen, oder daß der einer oder mehreren amöboiden Gliazellen zugekehrte Teil der Ganglienzelle blasser gefärbt und in schon vorgeschrittener Auflösung begriffen ist, als der von ihnen abgekehrte.

Wenn wir dann den weiteren Entwicklungsgang der amöboiden Gliazellen mit der S-Fuchsin-Lichtgrünmethode verfolgen, so sehen wir, daß sich in dem zunächst kleinen, tief und homogen gefärbten Zelleib fuchsinophile Granula entwickeln und daß mit zunehmender Größe schließlich mit Osmium gebräunte Stoffe in ihnen auftreten. Diese behalten meistens die Form solider Körnchen, lipoide Cystchen findet man seltener in ihnen. Mit dem Auftreten dieser Einlagerungen färbt sich das Plasma allmählich weniger intensiv, wird schließlich in seinem Gefüge gelockert, gerinnselartig und löst sich völlig auf. Ganz häufig begegnet man zerfallenden Ganglienzellen, umlagert von einer Gruppe amöboider Zellen in sehr verschiedenen Entwicklungsstadien. Manchmal sehen wir sie um den Rest einer Ganglienzelle gelagert, der nur noch aus einem zerfallenden Kern besteht. Schließlich wird die Stelle, welche ursprünglich die Ganglienzelle ausgefüllt hat, völlig von einem Haufen amöboider Gliazellen eingenommen. Gleichzeitig häufen sich pathologische Produkte, sowie manchmal recht zahlreiche amöboide Gliazellen in den perivaskulären Räumen an und die fettigen Stoffe in den mesodermalen Gefäßzellen zeigen eine beträchtliche Vermehrung.

Die Beobachtung, daß die amöboiden Gliazellen den Ganglienzelleib, seine Fortsätze und den Kern deformieren, kann den Gedanken nahe legen, daß sie in aktiver Weise die Ganglienzellen zerstören. Wahrscheinlicher aber sind es nur die Reize in den Ganglienzellen gebildeter pathologischer Produkte, die sie anziehen. Sie mögen dann ihre Verflüssigung beschleunigen. Jedenfalls liegt es

nahe anzunehmen, daß die Ganglienzelle in umgewandelter Form in das Protoplasma und die Körncheneinschlüsse der Gliazellen übergegangen ist, wenn man diese, mit verschiedenerlei Abbaustoffen beladen, schließlich in großer Zahl den Raum ausfüllen sieht, den früher die Ganglienzelle eingenommen hat. Der größte Teil der pathologischen Stoffe, die wir in den perivaskulären Räumen finden, scheint wieder vom Zerfall dieser Gliazellen herzurühren, und diese Produkte wieder dürften dann später in den mesodermalen Zellen in lipoide Stoffe umgewandelt werden. So füllt diese Beobachtung eine der Lücken aus, die sich bei der Betrachtung der Verhältnisse im Mark nicht völlig haben schließen lassen. Wir konnten dort nur selten die Gliazellen in engerer Beziehung zu den zerfallenden nervösen Elementen treten sehen. Hier beobachten wir in vielfältiger Art, wie sich die gliösen Elemente an die erkrankten Nervenzellen anlegen und offenbar mit zu ihrer Verflüssigung beitragen.

Eine andere, ungemein verbreitete Erkrankungsart der Ganglienzellen ist die fettige Degeneration. Auch hier finden wir sehr zahlreiche Verschiedenheiten, einerseits hinsichtlich der Menge, Größe und Anordnung der lipoiden Körnchen, die sich in der Zelle vorfinden, andererseits in dem Zusammentreffen mit anderen pathologischen Veränderungen der Zelle. In ihren typischsten Formen treffen wir sie bei der senilen Demenz, aber auch bei der Paralyse, der Arteriosklerose, der Lues, beim chronischen Alkoholimus und Morphinismus, bei Intoxikations- und Infektionspsychosen, ja schließlich wohl bei allen degenerativen Prozessen im Nervengewebe begegnen wir ihr, kombiniert mit anderen Veränderungen der Ganglienzelle. Am besten läßt sie sich studieren im Rückenmark bei der senilen Demenz, da sie auch bei der senilen Demenz in der Hirnrinde, meist mit anderen Veränderungen, besonders häufig mit sklerotischen, zusammen vorkommt.

Man hat die fettige Degeneration der Ganglienzellen früher auch als Pigmententartung, die Körnchen selbst als gelbes Pigment bezeichnet, weil die Körnchen in ungefärbten oder in frischen Zupfpräparaten eine lichtgelbe Färbung zeigen. Da wir aber sehen, daß vielerlei fettige Stoffe gelbliche Farbe annehmen, und festgestellt ist, daß die Körnchen die Reaktion lipoider Stoffe zeigen, werden wir besser die Einlagerungen als lipoide Körnchen und die Erkrankung als lipoide Degeneration bezeichnen. Dazu kommt noch, daß namentlich die lipoiden Körnchen, die sich in jüngeren Jahren

bilden, und die bei akuten degenerativen Prozessen frisch entstanden sind, vielfach ungefärbt erscheinen, so daß die Pigmentierung nicht als charakteristisches Merkmal gelten kann.

Über die fettige Degeneration der Ganglienzellen ist eine reiche Literatur vorhanden. In den Arbeiten Obersteiners und Marinescos finden wir sowohl diese aufgeführt, wie auch viele eigene Untersuchungen mitgeteilt. Ich kann also bezüglich der Literatur und der allgemeinen Verhältnisse der lipoiden Degeneration der Ganglienzellen auf die Darlegungen dieser Autoren verweisen. Das braune oder schwarze melanotische Pigment des Locus coeruleus, der Substantia nigra und des Vaguskerns, dem offenbar eine wesentlich andere Bedeutung zukommt, sowie das dunkle Pigment der sympathischen und spinalen Ganglienzellen will ich hier außer Betrachtung lassen, ebenso auch die verschiedene Lagerung der Fettkörnchen in den einzelnen Zellen. Hier soll uns lediglich die Bedeutung der fettigen Degeneration für die Ganglienzelle und das nervöse Gewebe im allgemeinen, die Art der fettigen Produkte und die Beziehung der fettigen Degeneration zu den amoeboiden Gliazellen beschäftigen.

Die Abschätzung der pathologischen Bedeutung der Fettdegeneration der Ganglienzelle wird erschwert durch die Beobachtung, daß sowol beim Menschen, als auch bei den größeren Tieren lipoide Körnchen zu finden sind, ohne daß irgendwelche besondere Schädigung das Nervensystem getroffen hat, und ohne daß eine Störung in der Leistung dadurch verursacht zu werden scheint. Dazu sehen wir diese fettigen Körnchen in den Ganglienzellen schon in ganz früher Jugend auftreten, ehe das Nervensystem voll entwickelt ist, so daß man von einer Abnützung, geschweige denn von einer Involution des Nervengewebes noch nicht reden kann. So konnte wohl die Meinung aufkommen (Olmer), daß das Fett in den Ganglienzellen als eine Art Reservematerial für die Ernährung der Zelle anzusehen sei. Wie aber schon Obersteiner, Marinesco u. a. betont haben, spricht die starke Anhäufung des Fettes in den Ganglienzellen im Senium, wie die Zunahme desselben bei vielerlei Schädigungen, welche die Zelle treffen, gegen eine solche Annahme. Wenn wir sehen, daß für die Funktion zweifellos wichtige Zellbestandteile, Plasma, Nisslschollen und Fibrillen geschädigt werden, ja daß die Zelle schließlich mit einer übermäßigen Anhäufung dieser Fettkörnchen der Auflösung verfällt, kann uns eine Auffassung, die in

dem Fett einen nützlichen Bestandteil der Zelle erblickt, kaum einleuchten. OBERSTEINER hat die Meinung vertreten, daß es sich auch bei diesen Fettkörnchen, die in den Ganglienzellen eines sonst nicht geschädigten Nervensystems auftreten, um Abfallsprodukte des Zellstoffwechsels handelt, deren Abführung zum Schaden der Zelle nicht möglich ist, und daß vielleicht diejenigen Zellen, in welchen sich leichter Fettkörnchen ansammeln (lipophile Zellen), stärker funktionell in Anspruch genommen sind als die, in welchen keine oder nur in geringerem Grade Fettansammlung auftritt (lipophobe Zellen OBERSTEINERS). Der erste Teil der OBERSTEINERschen Ansicht hat wohl ziemlich allgemeine Zustimmung erfahren. Wir sehen ja wohl auch in anderen Körperzellen, manchmal schon in jugendlichen Jahren, degenerative fettige Körnchen auftreten, die zunächst die Funktion der Zelle nicht zu beeinträchtigen scheinen, bis ihre Anhäufung mit vorgeschrittenerem Alter mehr und mehr exzessive Grade erreicht, das Plasma der Zelle wesentlich verändert und dann auch eine Störung in der Leistung des Organs nach sich zieht. Wir werden demnach auch diese fettigen Körnchen als degenerative Produkte ansehen. Damit hat es aber denn auch eine gewisse Berechtigung, zwischen einer physiologischen Fettdegeneration der Ganglienzellen, die in der Breite des gewöhnlichen bleibt, und einer pathologischen, die diese überschreitet, zu unterscheiden. Für das Studium der pathologischen Fettdegeneration ist aber die Kenntnis der physiologischen Voraussetzung. Da sich die einzelnen Nervenkerne und Zelltypen hinsichtlich dieser physiologischen Fettdegeneration sehr verschieden verhalten, das Alter für die Menge der Fettkörnchen von wesentlicher Bedeutung ist, und daneben noch zweifellos erhebliche individuelle Schwankungen vorkommen, wird es sehr schwierig, Regeln aufzustellen darüber, was den einen oder den anderen zugehört. Wenn NISSL sich dahin ausgesprochen hat, daß eine Zelle als degeneriert zu betrachten ist, deren ganzer Körper mit Fettkörnchen erfüllt oder deren Dendriten durch Einlagerung von Fettkörnchen an ihrer Abgangsstelle atrophiert sind, so sind damit nur die allerschwersten und offensichtlichsten pathologischen Fälle inbegriffen. Wir sehen, und werden später noch darauf zurückkommen, daß durch mancherlei pathologische Einflüsse die fettigen Körnchen in den Ganglienzellen vermehrt werden, und müssen auch dieses Fett als pathologisch betrachten. Beim Menschen läßt sich diese pathologische Fettvermehrung oft nur nachweisen, wenn wir Vor-

stufen desselben, die nur kurze Zeit zu bestehen pflegen, darstellen können; bei Tieren, bei welchen fettige Körnchen normalerweise völlig fehlen, wie beim Kaninchen, ist schon das Auftreten einzelner Fettkörnchen als pathologisch anzusehen. Auch manche ungewöhnliche Anordnungen der fettigen Körnchen in den Ganglienzellen weisen auf pathologischen Ursprung derselben hin; OBERSTEINER hat einige Beispiele dafür abgebildet.

Die von OBERSTEINER geäußerte Meinung, daß die fettigen Stoffe in den Ganglienzellen sich ansammeln, weil ihre Abführung nicht möglich sei, muß uns die Frage nahelegen, ob und in welcher Weise in den Nervenzellen gebildete lipoide Körnchen aus ihnen herausgebracht und beseitigt werden können. Die Frage ist nicht leicht zu beantworten. Wo wir reichlich Fettkörnchen in den Ganglienzellen finden, vermissen wir dieselben wohl nie in den Gliazellen, besonders in den Trabantzellen und in den Zellen der Gefäßwand. Es bleibt nun die Frage zu entscheiden, ob die fettigen Körnchen hier ebenso durch die gleichen Schädigungen gebildet werden wie in den Ganglienzellen, durch Umwandlung des Protoplasmas, oder ob sie von den Ganglienzellen herstammen. Der erste Fall dürfte sicher vorkommen. Die Gliazellen der ersten Schicht der Hirnrinde führen gewöhnlich Fett, das wahrscheinlich degenerativen Veränderungen des Ganglienzellenplasmas seine Entstehung verdankt, und in älteren gliösen Narben sehen wir zuweilen den zerfallenden Plasmaleib der Gliazellen, die reichlich Fasern gebildet haben, mit Fettkörnchen erfüllt, welche ebenso entstanden sein dürften. Gelegentlich treffen wir Bilder, welche den Eindruck erwecken könnten, als ob die Ganglienzelle wirklich in ihr enthaltene lipoide Körnchen auszustoßen imstande wäre. Man sieht besonders in den Pyramidenzellen der Hirnrinde Fettkörnchen, die zu langen wurstartigen oder hantelförmigen Gebilden ausgezogen sind und zur Hälfte über den Ganglienzelleib herausragen. Meist sind das aber sklerosierte Zellen, und das muß daran denken lassen, daß sie bei der Schrumpfung des Zelleibes gleichsam aus ihm herausgepreßt worden sind. Auch bei anderen Zellen könnten durch die künstliche Schrumpfung des Zellprotoplasmas ähnliche Bilder veranlaßt worden sein. So kann man derartige Beobachtungen nicht als einen Beweis dafür gelten lassen, daß sich die Ganglienzelle ihres Ballastes auf so einfache Weise entledigen kann.

In sklerosierten Zellen finden wir die lipoiden Körnchen dann oft schalenartig, wie ausgelaugt. Aber hier handelt es sich um nekrobiotische Zellen und daß durch fettige Degeneration in Häufchen von Fettkörnchen zerfallene Zellen rasch und, wie es scheint, unter Mitwirkung von Gliazellen, völlig aufgelöst werden, ist gelegentlich ganz unzweifelhaft wahrzunehmen.

Daß die lebende Zelle das unter pathologischen Einflüssen entstandene Fett wieder abgeben kann, dürfte aber durch das Tierexperiment wahrscheinlich gemacht werden. Die Deutung der Befunde ist hier weniger schwierig, weil man bei geeigneter Auswahl der Versuchstiere die physiologische Fettdegeneration nicht in Betracht zu ziehen braucht. Junge Kaninchen haben normalerweise kein oder höchstens Spuren von Fett im zentralen Nervengewebe. Nach zwei Monate langer Vergiftung mit Bleikarbonat war es in beträchtlicher Menge in den Ganglienzellen, vereinzelt in den Gliazellen, etwas reichlicher in den Zellen der Gefäßwand nachweisbar. Bei Kontrolltieren, bei welchen die Vergiftung an dem Tage ausgesetzt wurde, an welchem die ersten getötet worden waren, fand sich nach weiteren 6 Wochen sehr wenig Fett in den Ganglienzellen, mehr in den Gliazellen und vielleicht noch reichlicher in den Zellen der Gefäßwand. Dieser Befund spricht wieder für einen Abtransport der fettigen Stoffe von den Ganglienzellen auf dem Wege der Gliazellen in die Gefäßwandzellen. Allerdings läßt auch dieser Versuch noch einige Bedenken übrig. Wir sehen nämlich unter gewissen pathologischen Umständen Fett in den Gliazellen auftreten, das nicht aus den Ganglienzellen stammen kann, da es schon auftritt, ehe Fett in diesen nachweisbar wird. Ich habe dies schon lange bei der Alkohol- und Phosphorvergiftung beobachtet, ACHUCARRO bei der Lyssa, BONFIGLIO bei der Bleivergiftung. Wir werden später auf seinen Ursprung zurückkommen.

Jedenfalls ist bemerkenswert, daß überall dort, wo wir in physiologischer Breite Fett in den Ganglienzellen finden, auch solches in den Gliazellen und in den Zellen der Gefäßwand vorkommt, daß aber bei den Tieren, bei denen normalerweise keine lipoiden Körnchen in den Nervenzellen nachzuweisen sind, sie normalerweise auch in den Gliazellen und in den Zellen der Gefäßwand fast stets fehlen. So ist beim Menschen und einer Reihe von Tieren auch das Auftreten fettiger Stoffe in der Glia und in den Gefäßwandzellen in demselben Sinne wie das Vorkommen desselben in den Ganglienzellen eine Erscheinung in der Breite des Normalen. Man würde vergeblich nach einem normalen Gehirn eines erwachsenen Menschen suchen, wenn man auch das Auftreten von fettigen Körnchen in den Gliazellen und den Zellen der Gefäßwand als pathologich ansehen wollte. Daß aber die fettigen Produkte gleichzeitig in den Ganglienzellen, Gliazellen und Gefäßwandzellen vorkommen oder fehlen, dürfte auch wieder auf enge Beziehungen in dem Auftreten der fettigen Stoffe in den drei Zellformen schließen lassen.

Unter pathologischen Umständen scheinen aber auch Ausnahmen von dieser Regel vorzukommen. So habe ich wiederholt Fälle von Dementia senilis beobachtet, in denen die Ganglienzellen der Hirnrinde in exzessiver Weise fettig entartet waren, während die dazwischen gelegenen Gliazellen kein Fett enthielten. Bei genauerer Betrachtung aber erschienen alle diese Gliazellen erheblich degenerativ verändert, so daß nicht auszuschließen war, daß sie die Fähigkeit, fettige Stoffe aufzunehmen oder zu bilden, eingebüßt hatten.

Wahrscheinlich haben wir uns das Auftreten von Fettkörnchen in Ganglien- und Gliazellen nicht so zu erklären, daß durch eine normale oder übermäßige funktionelle Leistung fettige Produkte gebildet und dann direkt von den Gliazellen übernommen werden, sondern daß sich die Funktion der Zelle unter stofflichen Umsätzen abspielt, die wir bis jetzt noch nicht nachweisen können, bei der es aber jedenfalls nicht zur Bildung nachweisbarer fettiger Produkte kommt. Erst unter besonderen Umständen dürfte es zu einer granulären Umwandlung des Plasmas in Fett kommen. Auch besitzen wir kaum irgendwelche Anhaltspunkte dafür, daß eine Überwanderung oder Übernahme fettiger Granula von einer Zellart in die andere stattfindet. Analogien bei anderer Art Abbauvorgängen sprechen mehr dafür, daß die fettigen Stoffe wieder aufgelöst werden, ehe sie in die andere Zelle gelangen.

Das Tierexperiment macht es wahrscheinlich, daß einzelne über den ganzen Zellkörper zerstreute Fettkörnchen leichter wieder aus der Ganglienzelle verschwinden als größere Fettanhäufungen, die einen großen Teil des Zellplasmas durchsetzen und sich an Lieblingsstellen der fettigen Degeneration etabliert und hier zu weitergehenden Veränderungen der Zellstruktur geführt haben. Allerdings ist dabei noch genauerer Prüfung bedürftig, ob nicht etwa in anderen Teilen der Zelle entstandene Fettkörnchen ihre Lage in der Zelle verändern und schließlich an einer Prädilektionsstelle abgelagert werden können.

Daß es sich bei den hier in Frage kommenden Einlagerungen der Ganglienzellen um lipoide Stoffe handelt, wird heute wohl von keiner Seite mehr ernsthaft bestritten. Sie bräunen sich mit Osmium nach vorausgegangener Chromierung, sie färben sich leuchtend rot mit Scharlach und sie werden durch längeres Einlegen der Schnitte in Alkohol und Äther, besonders bei gleichzeitiger Erwärmung, zu einem großen Teile extrahiert. Doch unterscheiden sie sich in recht wesentlichen Punkten von den fettigen Stoffen, welche wir unter anderen Umständen im zentralen Nervensystem antreffen.

Fertigen wir einen Schnitt durch ein Rückenmark, in welchem die weißen Stränge mit zahlreichen gliogenen Körnchenzellen angefüllt sind, deren Einlagerungen sich ebenso nach Chromierung mit Osmium schwärzen oder im Formolgefrierschnitt mit Scharlach röten, so sehen wir im Nissl-Präparat kaum etwas von ihnen. (Tafel XXXIII, Fig. 4 u. 5.) Dieses Fett zeigt also nicht die Neigung, gelbliche Farbtöne anzunehmen. Färben wir einen solchen Schnitt des Rückenmarks, nachdem er nur kurze Zeit in 96 Proz. Alkohol bei Zimmertemperatur gelegen hat, so finden wir die meisten Einlagerungen der Körnchenzellen extrahiert und nur noch Gitterstrukturen ihres Zelleibes gut darstellbar, während gleichzeitig die kleineren lipoiden Körnchen in den Zellen der grauen Substanz noch unbeschädigt scheinen. Sie sind also schwerer in Alkohol löslich als die fettigen Stoffe der gliogenen Körnchenzellen. Daneben gibt es eine ganze Reihe von Farbreaktionen, welche den lipoiden Einschlüssen der Ganglienzellen zukommen, den fettigen Stoffen der gliogenen Markkörnchenzellen aber fehlen. Die lipoiden Stoffe der Ganglienzellen färben sich bei vielen Modifikationen der Weigertschen Markscheidenfärbung blau oder schwarz, während sich dort fast nur die Gitterstruktur färbt. Nach Chromessigsäurefixierung ohne Osmium und S-Fuchsinlichtgrünfärbung werden sie rot, nach Alkoholfixierung und Karbolfuchsinmethylenblaufärbung behalten sie die Farbe des Karbolfuchsins. Wie ich schon lange weiß, und wie Casamajor neuerdings beschrieben hat, färben sie sich mit der Weigertschen Fibrinmethode blau. Allen diesen Färbungen gegenüber verhalten sich die lipoiden Stoffe der gliogenen Körnchenzellen des Markes anders, sie färben sich nicht oder nur zu einem ganz geringen Teile.

Das sind nur einige Beispiele, die sich noch um zahlreiche andere vermehren ließen, aber jedenfalls schon hinreichend zeigen, daß wir hier einen eigenartigen lipoiden Stoff vor uns haben. Er ist nicht etwa dem zentralen Nervensystem eigentümlich, sondern findet sich auch in anderen Körperzellen. Im Nervengewebe selbst begegnen wir ihm nicht nur in den Ganglienzellen, sondern auch in den Gliazellen, in den Zellen der Gefäßwand und der Pia. Ich will davon absehen, diesem lipoiden Stoff einen Namen beizulegen, der etwas über seine speziellere chemische Konstitution aussagen würde, aus Gründen, auf die ich noch näher bei Besprechung der basophil metachromatischen Stoffe eingehen will.

Wir können dann weiter feststellen, daß dieser Stoff auch nicht immer vollständig übereinstimmende Farbreaktionen erkennen läßt. Unter manchen Umständen zeigt er im ungefärbten Präparat, wie schon erwähnt, nicht die gewöhnliche gelbliche Färbung, sondern ist farblos, in anderen wieder ist er auffällig dunkel, bräunlich, auch grünlich. Besonders bei einer sehr akuten Entstehungsweise sieht man im Alkoholpräparat viele Körnchen, die blaue basische Anlinfarben in einem grünlichen Tone annehmen. Gar nicht so selten kann man die Beobachtung machen, daß bei verschiedenen Färbungen Schale und Kern der einzelnen Körnchen sich anders färben. Es gibt Körnchen, die sich mit Osmium nach Chromierung völlig schwärzen, im übrigen wechseln gelbliche bis dunkelbraune Töne. Auf die vielen Verschiedenheiten in der Form und Größe der Körnchen, ihre Anordnung und Zusammenlagerung in der Zelle will ich hier nicht eingehen.

Da wir bei der Betrachtung der amöboiden Gliazellen zu der Meinung gekommen sind, daß die Fettcystchen, die lipoiden Einlagerungen der Gliazellen, aus fuchsinophilen Granula hervorgehen, müssen wir sehen, ob sich nicht auch vielleicht in der Ganglienzelle Präprodukte der lipoiden Stoffe auffinden lassen. Zunächst hat es den Anschein, als ob man in Präparaten, welche mit der Silbermethode Ramon y Cajals, noch besser mit der von Levaditi für den Spirochätennachweis angegebenen Silbermethode gewonnen worden sind, zuweilen mehr Körnchen darstellen kann, als sich mit Osmium bräunen oder Scharlach röten. Diesen Eindruck hat man besonders bei Zellen, in welchen eine akute Verfettung anzunehmen ist, z. B. bei schweren Infektionsdelirien. Da man aber bei Anwendung der verschiedenen Methoden nicht dieselben Zellen und nicht einmal ganz übereinstimmende Stellen der Hirnrinde untersuchen kann, ist es schwierig, zu einem sicheren Urteil zu gelangen. Dagegen sieht man öfters, daß sich bei der Säurefuchsinlichtgrünfärbung zwischen den

gebräunten Körnchen rote eingestreut finden, welche die Größe der normalen fuchsinophilen Granula der Ganglienzelle wesentlich übertreffen und an die Größe der lipoiden Körnchen heranreichen. Da aber in der Ganglienzelle pathologischerweise größere fuchsinophile Granula beobachtet werden, sind auch diese Befunde nicht eindeutig. Neuerdings aber habe ich Präparate erhalten, welche ganz unzweifelhaft dartun, daß die lipoiden Körnchen fuchsinophile Vorstadien haben. In einem Falle von schwerer Tetanie nach Kropfexstirpation fanden sich nach der Säurefuchsinlichtgrünfärbung in den Vorderhornzellen des Rückenmarks, besonders der Halsanschwellung, in sehr vielen Zellen die Häufchen lipoider Einlagerungen, umgeben von einem dichten Kranz mit Fuchsin stark gefärbter Körnchen, und in der Grenzzone zwischen beiden lagen immer solche, die braun mit roter und rot mit brauner Beimischung erschienen. Dabei zeigten sich diese Vorprodukte der lipoiden Körnchen durch ihre erhebliche Größe und ihr etwas lockeres Gefüge auffällig abweichend von den sonstigen fuchsinophilen Granula der Ganglienzellen. So müssen wir also annehmen, daß sich aus dem Zellplasma zunächst Körnchen bilden, die die Neigung haben, sich mit Säurefuchsin zu färben, und daß sich dann erst diese fuchsinophilen Körnchen in fettige Produkte umwandeln.

Die chronische Fettdegeneration der Ganglienzellen pflegt bei verschiedenen Zellformen fast stets an derselben Stelle zu beginnen und von da aus allmählich weiter zu schreiten. Bald ist es eine Zone in der Nähe des Kernes (Vorderhornzellen, Rindenpyramiden), bald eine Stelle über dem Kern am Abgang des Hauptdendriten (PURKINJEsche Zellen), bald der Hauptdendrit selbst (Zellen des Ammonshorns). Bei akuten Fettdegenerationen sehen wir häufiger eine gleichmäßigere Verteilung der Fettkörnchen über den ganzen Zelleib. Aus unseren Bildern gewinnen wir den Eindruck, daß sich das Fett aus der Plasmagrundsubstanz der Zelle bildet und die NISSL-Substanz von vornherein mit in die Umwandlung hineingezogen wird, während die Fibrillen sich verhältnismäßig widerstandsfähig zeigen. Wo Fettkörnchen massenhaft auftreten, verändert sich die Ganglienzelle wie jede andere Zelle, in der sich Fettkörnchen bilden, das Plasma nimmt an der Stelle, an der die lipoiden Granula liegen, einen wabigen Bau an, wie in den Gitterzellen, und in den so gebildeten Waben liegen die Körnchen. Die NISSL-Substanz geht dabei zugrunde. Die Fibrillen erhalten sich dagegen noch länger in den Maschen. Was man aber schließlich mit der BIELSCHOWSKYschen Methode noch färben kann, scheint mir nicht ein Netzwerk von Fibrillen, sondern die Maschen des plasmatischen Wabenwerkes. Mit der Bildung reichlicher lipoider Körnchen geht eine Anschwellung der Zelle einher. Dabei erfährt sie oft eine Deformierung. Besonders bei gleichzeitiger Sklerose wölben sich die verfetteten Stellen vor (Fettsäcke), der Kern wird verlagert, indem er den Fetteinlagerungen ausweicht, die Fibrillen teilweise verdrängt, die Fortsätze schwellen an, wenn sich in ihnen selbst Fett bildet, oder schrumpfen zusammen, wenn an ihrer Abgangsstelle eine Fettanhäufung liegt (NISSL). Schließlich kann die ganze Zelle in Fettkörnchen sich umwandeln, was dann ihren Zerfall zur Folge hat.

Von der gewöhnlichen Form der fettigen Degeneration der Ganglienzellen läßt sich eine Abart unterscheiden. Bei der senilen Demenz, der Arteriosklerose und in und um Herde, besonders auch bei der Encephalitis, finden wir gelegentlich Ganglienzellen, in welchen besonders große Fettkörner auftreten. Diese Abart dürfte wohl mit der schon von MARINESCO als vierte Form (gros corpuscules) der Fettentartung beschriebenen zusammenfallen. Man sieht dabei oft außerordentlich große Fettkörner einen Teil oder den ganzen Zellleib ausfüllen, oft so, daß jedes Fettkorn in einer Vakuole zu liegen scheint. Diese großen Fettkörner zeigen nun einige Besonderheiten. Ein Teil davon, und wie es scheint der frischer entstandene, nimmt bei Anwendung der Methode V eine auffallend dunkelblaue Farbe an, ebenso bei Färbung mit EHRLICHschem Hämatoxylin, ohne sich mit Scharlach rot zu färben, während ein anderer Teil das Scharlach annimmt. Dagegen färben sich keine der Granula mit MAY-GRÜNWALD. Mit Lithionkarmin färbt sich ein Teil der Körner auffallend leuchtendrot, im BIELSCHOWSKY-Präparat nehmen sie sehr stark das Silber auf. Bei der Säurefuchsin-Lichtgrünfärbung nach FLEMMING-Fixierung finden wir einzelne leuchtendrot gefärbt, andere stark gebräunt und in derselben Form wie die Cystchen der amöboiden Gliazellen. So sehen wir also hier einige Vorstufen der großen Fettkörner, die wir bei der feinkörnigen Fettentartung nicht nachweisen konnten.

Nun habe ich wiederholt bei Greisen, bei denen sich offenbar eine sehr rasch fortschreitende fettige Degeneration entwickelt, und bei welchen sich in kurzer Zeit eine vollständige Aufhebung der Bewegungsfähigkeit eingestellt hatte, im Rückenmark, in der grauen

und weißen Substanz, zahlreiche amöboide Zellen gefunden. Sie waren in nichts verschieden von den amöboiden Zellen, die wir bei der schweren Zellveränderung Nissls in der Hirnrinde und im Affenrückenmark gesehen hatten. Das Nissl-Präparat zeigte stark fettig entartete, meist birnenförmige oder kugelige, vielfach fortsatzlose Zellen in der grauen Substanz; einige hatten durch Fetteinlagerung stark aufgetriebene Protoplasmafortsätze. In anderen, offenbar viel chronischeren Fällen fanden sich die gleichen Veränderungen an den Ganglienzellen, ohne eine Spur amöboider Zellen. Wie ist hier die Entstehung und Bedeutung der amöboiden Gliazellen zu erklären? Ich glaube, daß das Säurefuchsin-Lichtgrünpräparat darüber Aufschluß zu geben vermag. Betrachten wir ein normales Vergleichspräparat — am zweckmäßigsten wählt man beide Male Schnitte aus der Zervikal- oder Lumbalanschwellung, wo sehr viele große Ganglienzellen nebeneinander liegen — so sieht man das ganze Gewebe außer von den Ganglienzellen selbst von reichlichen, viel verzweigten Protoplasmafortsätzen erfüllt, welche der Schnitt von den Ganglienzellen abgetrennt hat. Alle sind dicht bedeckt von zahllosen Neurosomenhäufchen, darüber ziehen dann in außerordentlicher Menge feinste Achsenzylinder hin, die vielfach parallel zu den Protoplasmafortsätzen verlaufen und allenthalben Zelle und Dendriten umspinnen. Wir sehen, wie ihre feinsten Verzweigungen bis in die Ebene der Neurosomen herabsteigen, zwischen ihnen hindurch ziehen, und hie und da glaubt man zu beobachten, wie sie mit ihnen in Beziehung treten. Mehr

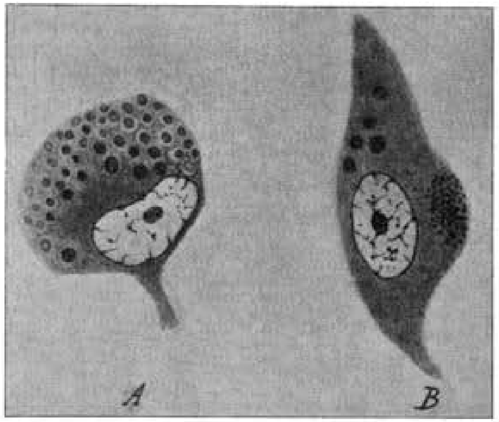

Fig. 4. Grobkörnige Fettdegeneration der Ganglienzellen. *A* aus dem Hirnstamm eines Arteriosklerotikers. *B* aus dem Ammonshorn der Dementia senilis (zeigt neben dem gelbgrünlichen gewöhnlichen Fett 5 blaugefärbte große Granula). Methode V.

zu erkennen gestatten die Präparate nicht. Daneben durchziehen noch größere Achsenzylinder und Markfasern das Gesichtsfeld. Das Übrige wird ausgefüllt durch eine außerordentlich fein strukturierte Glia und vereinzelte Gliafasern. Aber das Stützgewebe tritt stark zurück gegenüber der Fülle nervöser Elemente.

In dem senilen Rückenmark mit den stark verfetteten Ganglienzellen ist das Bild ganz anders. Die Zahl der protoplasmatischen Fortsätze ist ungemein ärmlich. Es müssen offenbar viele zugrunde gegangen sein. Damit hängt wohl auch zusammen, daß die normalerweise weit verzweigten Ganglienzellen oft Kugelform angenommen haben. Die Neurosomenhäufchen fehlen über den verfetteten Stellen der Zelle, und auch in anderen Gegenden sind sie spärlich geworden. Mit den Protoplasmafortsätzen sind sie auch im übrigen Gewebe verschwunden. Die Zahl der Achsenzylinder, welche an die Zelle herantreten, ist gering im Vergleich zu dem überraschenden Reichtum im normalen Präparate. Die Menge der Gliafasern ist größer geworden. An die Ganglienzellen angeschmiegt oder sonst im Gewebe zerstreut liegen amöboide Zellen, daneben noch überall Füllkörperchen in geringerer oder größerer Anzahl. Wer sich einmal klar gemacht hat, wie eng sich in dieser grauen Substanz normalerweise die nervösen Strukturen an einander drängen, dem muß schon die Anwesenheit solch zahlreicher gliöser Bildungen andeuten, daß hier viel Nervengewebe ausgefallen ist. Da ich nicht alle die Ver-

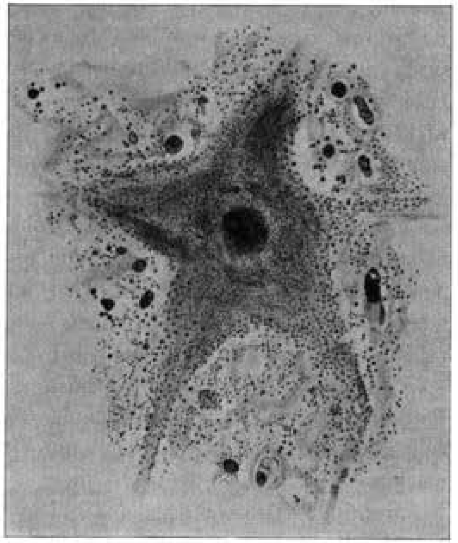

Fig. 5. Normale Ganglienzelle aus dem Vorderhorn eines Kaninchens. Zeigt die Neurosomenhäufchen aus der Ganglienzelle, die Granula im Zelleib und die zu Gliazellen gehörigen Granula. Methode VII.

hältnisse mit eigenen Zeichnungen veranschaulichen kann, möchte ich auf Tafel XXXV, Fig. 1 und 2 verweisen, die wenigstens ähnliches darstellt. So sehen wir aufs deutlichste, daß auch bei der fettigen Degeneration der Ganglienzellen neben einem Erhaltenbleiben ihrer groben Formen ein massenhafter Untergang feinerer nervöser Gewebsbestandteile stattfinden kann, dadurch, daß die weit verzweigten Dendriten der Zellen und die feinen an sie herantretenden Achsenzylinder, die perizellulären nervösen Anordnungen, zugrunde gehen. Solche Beispiele sind ungemein lehrreich, da sie zeigen, wie weitgehend nervöses Gewebe veröden kann, ohne daß wir mit unseren alten Untersuchungsmethoden viel davon feststellen können. Leider sind aber die Verhältnisse in der Hirnrinde noch viel verwickelter als im Rückenmark, alle Elemente noch viel zarter und daher viel schwieriger darzustellen, so daß hier auch mit den neuen Methoden völlig durchsichtige Bilder noch nicht zu erhalten sind. So müssen wir uns zunächst mit Beobachtungen, die an der gröbst gebauten grauen Substanz gewonnen worden sind, begnügen. Neben den amöboiden Zellen finden wir hier auch andersartige Gliaelemente, die Fasern gebildet haben, oder die, wie kleine Körnchenzellen, völlig mit Fettkörnchen beladen sind und keine Fasern zeigen. Alle Beobachtungen sprechen dafür, daß hier bei der stürmischen Fettentartung der Ganglienzellen das Auftreten der amöboiden Gliazellen mit einem raschen Zerfall der Dendriten und der die Zellen umspinnenden feinen Achsenzylinder, dem perizellulären nervösen Apparat im Zusammenhang steht. Das dürfte aber auch wahrscheinlich machen, daß dort, wo wir fettbeladene Gliatrabantzellen antreffen, ohne daß Fett in den Ganglienzellen nachweisbar ist, dies auf den Zerfall der periganglionären Strukturen zurückgeführt werden kann.

Ich glaube, diese Beispiele dürften genügen, die biologischen Aufgaben der amöboiden Gliazellen zu beleuchten. Ihnen obliegt nichts von der Aufgabe, welche der Glia als Stützsubstanz zukommt. Sie bilden keine Fasern und Netze, sie entstehen, wo ein Zerfall nervöser Gewebsbestandteile stattfindet, wohl weil der Reiz der Zerfallsprodukte sie bildet. Da wir nichts davon sehen, daß sie zerfallende Nervenstrukturen in körperlicher Form in sich aufnehmen, müssen wir annehmen, daß sie dieselben verflüssigen helfen. Bilder, die wir an Ganglienzellen beobachten, lassen kaum für eine andere Deutung Platz. Schließlich ersetzen sie, an Größe zunehmend, die Ganglienzellen, welche bis auf den letzten Rest verschwinden, oder liegen in mehr oder minder großer Zahl überall im Gewebe. Das läßt darauf schließen, daß sich Stoffe assimilieren, die bei der Auflösung der Ganglienzellen und anderer nervöser Strukturen entstehen. Auf der Höhe ihrer Entwicklung angelangt, erfahren sie rasch regressive Veränderungen, unter Bildung verschiedenartiger Körnchen, und verfallen schnell. Ihre Zerfallsprodukte geraten in die Lymphe und in die perivaskulären Räume, zum Teil wohl wieder in verflüssigtem Zustande, und werden von dort aus von den mesodermalen Zellen aufgenommen und in lipoide Stoffe umgewandelt. Sie säubern also das Nervengewebe von Abfallstoffen und führen diese in eine andere, dem ektodermalen Gewebe weniger schädliche Form über, und dem mesodermalen Gewebe zu, das sie einstweilen aufspeichert.

Wenn wir nun auch hier nicht alle die verschiedenen Abbauvorgänge im Nervengewebe darstellen können, so verdient doch jedenfalls noch die Beobachtung eine kurze Erwägung, daß neben den verhältnismäßig eintönigen Formen amöboider Gliazellen im Marke außerordentlich verschiedenartige Varietäten in der Rinde vorkommen. Man wird dies wohl damit erklären müssen, daß die Krankheitsvorgänge in der Rinde viel unterschiedlichere sind, bedingt durch ganz verschiedenartige Erkrankungszustände der Ganglienzellen, durch wechselnde Beteiligung der feineren nervösen Strukturen, der zarten Protoplasmafortsätze und der feinsten Achsenzylinderverzweigungen, während es sich bei den Erkrankungen des Markes im Vergleich hierzu nur um mehr quantitative Unterschiede handeln dürfte. Bei manchen der degenerativen Prozesse läßt sich dies schon direkt nachweisen. So hat es den Anschein, daß, je mehr sich die Zellform dem amöboiden Grundtypus nähert, desto stärker die Schädigung des nervösen Gewebes ist. Damit ergibt uns der Grad der Entwicklung der amöboiden Glia in gewisser Beziehung auch einen Gradmesser für die Schwere der nervösen Veränderungen.

Von einer großen pathologischen Bedeutung und bei einer besonderen Gruppe in enger Beziehung stehend zu dem Auftreten amöboider Gliazellen scheint das zahlreiche Auftreten großer fuch-

sinophiler Granula in den Ganglienzellen. Wir beobachten es unter verschiedenerlei Umständen: erstens bei chronischen Veränderungen der Ganglienzellen, namentlich bei manchen Formen, die der Sklerose nahe stehen, wo wir im Nissl-Bild eine retikuläre Struktur des Ganglienzellenplasmas finden und annehmen müssen, daß in den Maschen des Retikulums die großen fuchsinophilen Granula eingelagert sind, neben einer faserigen oder wenigstens inaktiven Glia (Textfigur 6); dann bei schwerer akuter Zellerkrankung, z. B. bei der Paralyse, Dementia praecox, neben reichlichen amöboiden Zellen. Schließlich finden sich dann bei mancherlei anderen Erkrankungszuständen Ganglienzellen, die vollgepfropft von fuchsinophilen Granula sind, ohne daß diese in ihrer Form und Größe von den normalen wesentlich abweichen, in den Fortsätzen strichelförmig, sonst oft länglich oder in Reihen gestellt (neben Gliazellen mit fuchsinophilen Körnchenreihen in langen Protoplasmafortsätzen oder nicht deutlich veränderten Gliazellen). Im ersten Falle scheint es sich um einen chronisch degenerativen Zustand, um eine Art nekrobiotischer Zellveränderung, im zweiten Falle um einen Abbauvorgang, im dritten zum Teil wohl nur um funktionelle Veränderungen zu handeln. Präprodukte lipoider Stoffe scheinen die fuchsinophilen Granula in keinem dieser Fälle. Versuche, diese verschiedenen fuchsinophilen Granula noch färberisch zu differenzieren, sind bis jetzt nicht geglückt.

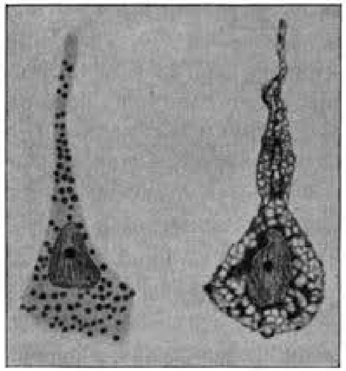

A *B*

Fig. 6. *A* Degenerative fuchsinophile Granula der Nervenzellen. 3. Zellschicht der Paralyse. Stirnhirn. *B* Entsprechendes Bild aus einem Nissl-Präparat, gleiche Stelle, zeigt statt der Granula Lücken.

Ganz eigenartige und einstweilen nicht gut verständliche Präparate habe ich schließlich noch von einem Falle einer Psychose der Rückbildungsjahre erhalten, die auch nach ihrer klinischen Erscheinung und ihrem Verlauf von besonderer Art war. Überall fanden sich massenhaft amöboide Zellen. An zahlreichen Ganglienzellen der tieferen Rinde, von welchen viele im Nissl-Bild die für die schwere Zellveränderung Nissls charakteristischen Veränderungen zeigten, lagen nach Färbung mit der S-Fuchsinlichtgrünmethode auf der Zelle keulenförmige und strichelartige, rot gefärbte Gebilde, die dem Anscheine nach nicht der Zelle angehörten, und die mir am meisten mit pathologisch veränderten, auf der Zelle gelegenen Achsenzylinderendigungen Ähnlichkeit zu haben schienen (Textfigur 7). Merkwürdigerweise färbten sie sich auch im Weigertschen Gliapräparate leicht blau. Ich habe sie sonst nirgends wieder angetroffen.

So zeigten uns diese Methoden außer einem Verständnis schon einigermaßen zugänglichen Veränderungen, noch mancherlei andere, die uns zunächst in ihrer Bedeutung unklar bleiben, aber bei genauerem Studium weitere Einblicke in die feineren pathologischen Vorgänge an den Nervenzellen in Aussicht stellen.

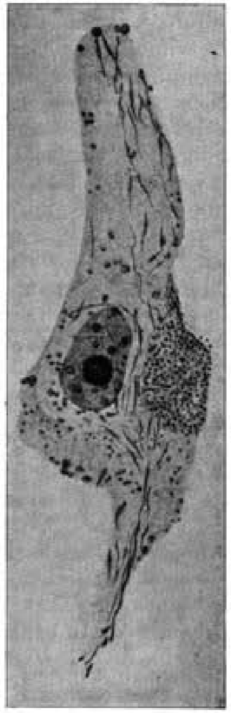

Fig. 7. Ganglienzelle mit schwerer Zellveränderung Nissls, enthält rechts lipoide Körnchen und zerstreut große fuchsinophile Granula. Die Zelle ist bedeckt mit fädenartigen, z. T. keulenförmig verdickten Gebilden, die wohl als Achsenzylinderendigungen zu deuten sind. S-Fuchsinlichtgrün.

5. Über besondere basophil metachromatische Abbauprodukte.

(π-Granula Reichs).

In mehreren Arbeiten hat Reich eine eigenartige Form von Granulationen in den Schwannschen Zellen der peripheren Nerven beschrieben und ihre morphologische Eigentümlichkeit sowie ihr färberisches Verhalten sehr eingehend geschildert. Wegen ihrer Löslichkeit in auf 45° erwärmtem Alkohol und erwärmtem Äther, sowie wegen mehrerer übereinstimmender Farbreaktionen hält er sie für identisch oder wenigstens verwandt mit dem von Liebreich 1865 auf chemischem Wege zuerst aus der Hirnsubstanz dargestellten Protagon.

Reich hat an verschiedenen Stellen der Meinung Ausdruck gegeben, daß meine Untersuchungen über den Abbau des Nervengewebes von seinen Forschungen den Ausgang genommen hätten. Zu dieser Annahme ist er wohl nur durch einen Irrtum von meiner Seite gekommen, den ich hier berichtigen muß. Ich hatte mir, wie ich am Eingang dieser Arbeit dargelegt habe, die Aufgabe gestellt, nachzuweisen, wie die lipoiden Stoffe, die wir bei degenerativen Rindenprozessen in den Adventitialzellen und der Pia angehäuft finden, dahin gelangen. Dabei suchte ich nach Vorstufen solcher Stoffe. Es war nun nichts natürlicher, als zu prüfen, ob nicht bei einem Abbau des Nervengewebes gleiche Produkte auftreten wie bei seinem Aufbau. So habe ich mich an Wlassaks Arbeit über die Herkunft des Myelins angelehnt. Wlassak hat nun angegeben, daß das Protagon sich mit der Weigertschen Markscheidenmethode färbt. Dies und einige andere Beobachtungen, die jetzt anzuführen überflüssig sein dürfte, veranlaßten mich, die Einlagerungen in den Ganglienzellen bei der amaurotischen Idiotie, auf welche wir noch später zu sprechen kommen werden, als dem Protagon verwandt anzusehen und mit anderen Körnchen, die ähnliche Reaktionen zeigten, protagonoide Granula zu benennen. Schon mit der Benennung „protagonähnliche Stoffe" sollten einige Bedenken gegen die völlige Übereinstimmung mit dem Protagon zum Ausdruck kommen, die sich im Verlauf der Untersuchung ergeben hatten. Aber erst Reichs Arbeit aus dem Jahre 1907 „Über den zelligen Aufbau der Nervenfaser auf Grund mikro-histiochemischer Untersuchungen", die erschien, als schon ein guter Teil dieser Arbeit fertig war, belehrte mich, daß wahrscheinlich Wlassak und ich im Unrecht waren, und daß die von mir und auch einigen meiner Schüler als protagonoide Granula bezeichneten Stoffe keine Protagongranula und Reichsche π-Granula waren. Seitdem habe ich mich auch viel mit den Reichschen π-Granula beschäftigt. Ich hatte sie schon früher in peripheren Nerven gesehen und war durch Reichs Dresdener Vortrag noch besonders auf sie aufmerksam gemacht worden. Aber damals glaubte ich noch, daß sie nur für den peripheren Nerven eine größere Bedeutung hätten. Wenn ich nun die zu den Reichschen π-Granula in enger Beziehung stehenden Stoffe hier als basophil metachromatische und nicht als Protagon- oder protagonoide Granula bezeichne, so hat das verschiedene Gründe. Es ist gewiß empfehlenswert, jedem neuen Stoffe, der beschrieben wird, einen Namen zu geben, damit man darüber reden kann, ohne immer wieder lange auseinandersetzen zu müssen, wovon man redet. Reich hat auch wahrscheinlich gemacht, daß seine π-Granula dem Protagon nahe stehen. Aber mein eigener früherer Mißgriff, unsere noch so mangelhaften Kenntnisse in der Chemie des Gehirns, die reichliche Zahl verschiedenartiger Granula und Stoffe, die uns im kranken Nervensystem begegnen, und die Zahl der Stoffe erheblich übersteigt, welche die Chemie bis jetzt aus dem Zentralnervensystem dargestellt hat, sowie der Umstand, daß wir ja vielfach pathologische Produkte vor uns haben, während sich die Chemie mit den unter krankhaften Bedingungen gebildeten Stoffen des Zentralnervensystems noch keineswegs erfolgreich beschäftigt hat, macht mir Bedenken, die im mikroskopischen Präparate gefärbten Stoffe mit den von der Chemie dargestellten zu identifizieren. Wir kennen heute schon verschiedene mit unseren Färbemethoden unterscheidbare Stoffe, welche zu den Fetten, verschiedene, die zu der myelinähnlichen und zwei, die zu der Protagongruppe gehören dürften. Welche sollen wir nun als Fett, Myelin und Protagon bezeichnen? Da wir sie z. T. erst allein durch färberische Eigentümlichkeiten zu trennen vermögen, können wir sie heute auch nur durch die Angaben über ihr Verhalten den Farben gegenüber charakterisieren. Dazu machen es noch mancherlei Beobachtungen wahrscheinlich, daß diese Stoffe im Zentralnervensystem nicht immer rein, sondern oft in mannigfachen Mischungsverhältnissen vorkommen und sich sozusagen in einem labilen Zustande befinden, d. h. im Gewebe rasche Veränderungen erfahren. Ich würde aber trotz allem keine Bedenken haben, die hier als basophil metachromatisch bezeichneten Stoffe mit dem von Reich vorgeschlagenen Namen „π-Granula" zu benennen, wenn ich nicht noch einige Zweifel hätte, ob alle die Stoffe, die ich im Nachfolgenden als metachromatisch basophile beschreibe, völlig mit den Reichschen π-Granula übereinstimmen.

Reich gebührt auf alle Fälle das Verdienst, daß er die morphologischen und färberischen Eigenschaften seiner π-Granula sehr eingehend festgestellt hat. Tafel XXXII, Fig. 8 zeigt uns vier Schwannsche Zellen aus einer Wurzel einer luetischen Meningo-Myelitis, welche ihre charakteristische Form und Färbung deutlich erkennen läßt. Sie sind hier etwas reichlicher als normal in dem offenbar vergrößerten Zelleib angehäuft. Man kann ganz deutlich wahrnehmen, wie die nur durch den Kern und die Anhäufung der Granula erkennbare Zelle die ganze Markscheide umfaßt. Die Granula selbst sind erstens gut gekennzeichnet durch ihre Form: es sind stäbchen-, strichel-, kommaförmige, ziemlich große Gebilde, die an einzelnen Stellen, hier an der untersten Zelle, wie Zwiebelschalen aneinander gelegt und zu größeren rundlichen Gebilden zusammengeordnet sind. Zweitens sind sie auch in ihrer Färbung sehr ausgezeichnet, indem sie bei Behandlung mit Thionin, Toluidinblau oder Kresylviolett eine besonders helle, brillante Metachromasie zeigen, heller leuchtend als die Metachromasie der Plasmazellenleiber oder der Mastzellengranula. Sie tritt, wie das Reich auch schon betont hat, bei künstlicher Beleuchtung mit elektrischem Licht

besonders schön hervor. Diese morphologischen und färberischen Eigentümlichkeiten müssen wir uns eingeprägt halten, wenn wir das Vorkommen dieser Stoffe im Zentralnervensystem selbst weiter verfolgen wollen. Dort begegnet schon ihre Darstellung etwas größeren Schwierigkeiten. Während es nämlich im peripheren Nerven leicht gelingt, eine elektive Färbung der π-Granula zu erhalten, ja während man ganz gut Alkoholschnitte zur Darstellung der Granula benutzen kann, geht dies an Alkoholschnitten des Zentralnervensystems kaum mehr, an Schnitten, die mit Formol, MÜLLERscher oder ORTHscher Flüssigkeit fixiert sind, sehr viel schwerer. In den Alkoholschnitten färbt sich nämlich das UNNAsche Neuromucin, über dessen Deutung ich mit REICH durchaus übereinstimme. In Präparaten, welche in wenig oder ungewechseltem Alkohol aufbewahrt waren, sieht man dazu noch die auch von REICH erwähnten kugel- und rosettenförmigen Konkremente, Markscheidenextraktionsprodukte, gelegentlich an Stellen, z. B. in den Vorderhornzellen des Rückenmarks, wohin sie wohl sicher erst gelangt sind durch spätere Ausfällung aus dem Alkohol, nachdem er durch Verdunstung oder Wasseraufnahme aus dem Gewebe weniger konzentriert geworden war. Mit der zunehmenden Entfärbung dieser Markscheidenstoffe entfärben sich auch die, wie Kontrollpräparate beweisen, oft tatsächlich vorhandenen, basophil metachromatischen Zelleinschlüsse so weit, daß es schwer ist, die Menge dieser Stoffe festzustellen oder sich bei geringerer Entfärbung vor einer Verwechselung mit Markstoffextrakten, die in den Zellen niedergeschlagen sind, zu schützen. In etwas geringerem, aber doch immer noch störendem Grade macht sich auch an Formol- und ORTHschen Schnitten der Übelstand bemerkbar, daß sich ein Teil der basophil metachromatischen Stoffe, namentlich die feineren Einlagerungen entfärben, ehe das Mark genügend entfärbt ist, um sie deutlich hervortreten zu lassen. Daß sie in peripheren Nerven um vieles leichter und isolierter zu färben sind, sich hier auch viel leichter dauerhafte Präparate herstellen lassen, dürfte seinen Grund darin haben, daß sie hier in größeren Portionen und in einer reineren und festeren Form abgelagert sind.

So vorsichtig man also jedenfalls bei der Beurteilung von Alkoholpräparaten sein muß, so scheint es doch nützlich, kurz auf sie einzugehen, weil die NISSL-Färbung wohl immer eine der gebräuchlichsten Methoden für die Untersuchung des Zentralnervensystems bleiben wird, um eine allgemeine Übersicht über die pathologischen Veränderungen zu erlangen, und weil wir, wie die Kontrollpräparate beweisen, auch hier schon einiges von diesen basophil-metachromatischen Zelleinschlüssen erkennen können, wenn wir nur mit genügender Vorsicht vorgehen.

Tafel XXXII, Fig. 6 zeigt uns einen Längsschnitt durch ein Gefäß mit seinem adventitiellen Lymphraum aus der Markleiste eines an einem infektiösen Delirium (unbekannte Infektionsart) rasch verstorbenen Mannes. Der Schnitt ist nach nur 18 stündiger Härtung in 96 Proz. Alkohol ohne Einbettung gefertigt und mit Toluidinblau gefärbt. Wir sehen im adventitiellen Lymphraum eine Anzahl von kleinen Kernen mit wenigem Plasma, die wir wohl unbedenklich als Lymphozyten bezeichnen können. Daneben liegen einige Zellen mit großen, hellen Kernen und größerem, runden, homogenen, blaß gefärbten Zelleib, die, wie Beobachtungen an anderen Stellen beweisen, als die Mutterzellen jener gitterigen und zum Teil mit rötlichen, gelblichen und grünlichen Massen angefüllten Körnchenzellen, welche weiter den Lymphraum ausfüllen, anzusehen sind. Von den verschiedenfarbigen Einlagerungen interessieren uns hier am meisten die metachromatisch rötlich gefärbten. Die Farbe entspricht der Farbe der REICHschen π-Granula. Dagegen sehen wir hier kaum etwas von der Form, welche den Granula der peripheren Nerven eigentümlich ist. Der rote Stoff ist vielmehr abgelagert in kleinsten Ballen, die rundlich, aber meist, doch etwas unregelmäßig abgegrenzt sind und so dicht neben und übereinander lagern, daß sie als eine wolkige Masse den ganzen Zelleib ausfüllen und den Kern an die Peripherie drängen. In den meisten Zellen aber sehen wir neben den rötlichen Stoffen gelbe und grüne Ballen und Klumpen. Im allgemeinen wird man finden, daß in den jünger aussehenden Zellen fast nur rote Stoffe, in den älteren überwiegend oder ausschließlich gelbe und grüne abgelagert sind, so daß der Schluß gerechtfertigt sein dürfte, daß sich in diesen Zellen die roten Stoffe in die gelben und grünen umwandeln.

Tafel XXXII, Fig. 11 stellt drei Gliazellen aus dem Mark des Mittelhirns eines Falles von Chorea progressiva dar, gleichfalls nach kurzer Alkoholfixierung und Toluidinblaufärbung, die zeigen sollen, wie reichlich diese Stoffe sich in Gliazellen eingelagert finden. Man kann wohl in diesen beiden Fällen ausschließen, daß diese metachromatisch gefärbten Produkte aus dem Härtungsalkohol, in welchen sie durch die Extraktion der alkohollöslichen Markbestand-

teile gelangt sein könnten, in diesen Zellen nachträglich niedergeschlagen worden sind; denn die ganze Form der Zellen, die Verlagerung des Kerns und seine degenerativen Veränderungen zeigen, daß wir hier Zellen vor uns haben, die durch Anhäufung pathologischer Stoffe zu Körnchenzellen umgebildet worden sind. Wollte man trotzdem noch Bedenken erheben, so müssen Bilder darüber beruhigen, wie sie uns Fig. 5 und 10 darstellen, die von Formolgefrierschnitten gewonnen sind. Fig. 5 gibt ein Bild einer kleinen Vene aus der Markleiste eines Epileptikers, der in der Zeit vor seinem Tode viele Anfälle gehabt hatte und in einem Anfall erstickt war. Fig. 10 zeigt zwei Gliazellen aus der Medulla oblongata eines Arteriosklerotikers. Die Bilder beweisen zunächst, daß es Körnchenzellen gibt, welche ganz mit diesen metachromatisch basophilen Stoffen gefüllt sind. Wir sehen in der adventitialen Lymphscheide in Fig. 5 zahlreiche große, kugelige Zellen mit Kernen, die an die Oberfläche gedrückt sind und dieselben regressiven Veränderungen zeigen, wie wir sie an den Kernen der mit Fettkörnchen angefüllten Abräumzellen gewöhnlich beobachten. Auch hier finden sich die roten Stoffe wieder in größeren und kleineren Ballen eingelagert, die oft aus kleineren, im allgemeinen rundlichen, meist aber unbestimmt umgrenzten Häufchen zusammengesetzt sind, nicht aber aus jenen scharfen Strichel- und Schalenformen, wie sie die π-Granula der peripheren Nerven zeigen. Auch hier beobachten wir wieder, daß die Zellen gleichzeitig grünliche Einschlüsse enthalten. Man findet Ballen, die noch rötlich, aber schon mit gelblichem oder grünlichem Anflug, oder solche, die halb rot, halb grünlich gefärbt sind. Es scheint auch hier nicht zweifelhaft, daß sich die rötlich gefärbte Substanz in der Zelle selbst in die gelbe und grünliche umwandelt. Das gleiche Bild gibt uns auch eine gute Übersicht über die Art des Vorkommens dieser basophilen metachromatischen Substanzen. Weitaus die größte Menge finden wir in großen Zellen, aller Wahrscheinlichkeit nach mesodermaler Abstammung, innerhalb der adventitiellen Lymphscheiden. Ganz links sehen wir eine Zelle, die halb im Nervengewebe, halb in einem perivaskulären Raum liegt. Wegen ihrer Lage dürfte sie für eine Gliazelle angesprochen werden, obwohl sie sich in ihrem Aussehen von den übrigen, innerhalb der Adventitia liegenden Zellen kaum unterscheidet. Allerdings wird man bei einem Gefrierschnitt nicht leicht mit Sicherheit ausschließen können, daß eine solche Zelle durch das Messer aus ihrer ursprünglichen

Lage verschoben ist. Daß aber auch ganz ähnliche Gliazellen vorkommen, beweist die Fig. 10, die mitten im nervösen Gewebe gelegene Zellen darstellt. Fast überall, wo wir besonders viele solche Stoffe in den Lymphscheiden antreffen, sehen wir dann auch im benachbarten Nervengewebe zweifellose Gliazellen, die den gleichen Stoff enthalten. Dabei liegen die am stärksten beladenen meistens dem Gefäß am nächsten, weiter abseits vom Gefäße finden sich immer weniger solche Stoffe in den Zellen. Unter den Zellen bemerken wir solche, die nur wenige Körnchen rings um den Zellkern herum zeigen, solche, bei denen sie an beiden Polen angeordnet sind, und solche, bei welchen sie in dichten Klumpen an einer Seite des Kerns oder in langen, peitschenförmig ausgezogenen Fortsätzen liegen. Die letzte Form sehen wir besonders häufig. Gewöhnlich sind in diesen Zellen keine gelben oder grünen Einschlüsse. Wie aber die Fig. 7 zeigt, finden sich auch manchmal in Gliazellen beide Formen nebeneinander. Manche von den metachromatisch basophil gefärbten Einlagerungen entsprechen durch ihre bestimmtere stäbchenförmige Gestalt mehr der Form der π-Granula, und man kann wohl sagen, daß solche am ersten dort zu sehen sind, wo sich noch sehr wenige solcher Einlagerungen in der Gliazelle befinden.

Ein ähnliches Bild wie Fig. 5 zeigt die Fig. 4, welche aus der tieferen Hirnrinde in der Gegend der Radii eines Falles einer schweren manischen Erregung stammt, der an Erschöpfung (?) zugrunde gegangen ist. Wir sehen hier wieder eine kleine Vene, die Zellen der Intima und Adventitia treten sehr deutlich hervor, außerdem der perivaskuläre Raum und das leicht gefärbte Nervengewebe. Im perivaskulären Raum liegt links ein Haufen von Gliazellen, an denen nur die Kerne hervortreten, dazwischen dann noch andere Gliazellen, die mit roten Stoffen beladen sind. Die größte Menge dieser Stoffe aber liegt in eigentümlicher, radiär zum Gefäße gestellter Anordnung, wahrscheinlich in Gliafortsätzen; in die großen rötlichen Klumpen sind auch wieder grünliche Kugeln eingelagert. Auch im Nervengewebe selbst lassen sich einige Gliazellen mit basophil metachromatischen Einschlüssen wahrnehmen.

Nicht selten begegnet man dann diesen Einschlüssen auch ganz in der Form der π-Granula der SCHWANNschen Zellen. Die Fig. 9 enthält Abbildungen von etwas gewucherten Gliazellen aus der weißen Substanz des Rückenmarks eines Falles von tuberkulöser Meningitis. Bald ist der ganze Zelleib von scharf geformten Stricheln ausgefüllt,

bald sehen wir sie nur in den Fortsätzen, oft weitab vom Zelleib. In der Fig. 9*d* sind sie ganz wie Zwiebelschalen angeordnet, wie wir das so häufig in peripheren Nerven antreffen. Auch in manchen Zellen der Fig. 1 und 2 sehen wir deutliche Strichel.

Soweit ich sehen kann, finden sich diese Stoffe nicht frei im Gewebe, sondern stets unter Verhältnissen, welche die Annahme rechtfertigen, daß sie in Zellen oder deren Fortsätzen, die nur manchmal durch den Schnitt von der Zelle abgetrennt wurden, eingelagert sind.

Sind nun diese Stoffe alle den Reichschen Granula gleich? Ihre morphologischen Eigenschaften sind nicht ganz mit ihnen übereinstimmend. Auch ihre Löslichkeitsverhältnisse scheinen etwas anders. Durch Einlegen der Formolschnitte in Alkohol nehmen sie an Menge deutlich ab. Am meisten bedenklich hat mich aber die Beobachtung gemacht, daß ein Kontrollschnitt zu dem Präparat, welchem die Zeichnung 5 entstammt, mit Scharlach gefärbt, die in den adventitiellen Lymphscheiden gelegenen Zellen in einem so leuchtenden Rot darstellte, daß sie alle nur aus rotgefärbten Massen zu bestehen schienen, während im Nervengewebe selbst an den Gliazellen kaum eine Fettfärbung nachzuweisen war. Versuche an anderem Material haben das gleiche Ergebnis geliefert, nur fand sich auch manchmal der Inhalt der Gliazellen mit Scharlach gefärbt. Nun wissen wir, daß die π-Granula keine Scharlachfärbung annehmen. Es wäre nun allerdings denkbar, daß die rote Farbe so überwiegt, daß dazwischen gelegene basophil metachromatische Stoffe völlig verdeckt würden. Aber auch bei allerlei Doppelfärbungen ergab sich immer eine vorwiegende Fettfärbung.

Man kann diese Beobachtungen vielleicht am zwanglosesten so erklären, daß unter pathologischen Umständen mit den π-Granula der peripheren Nerven übereinstimmende Stoffe in den Gliazellen auftreten. Öfters aber sind sie wohl färberisch mit diesen übereinstimmend, morphologisch aber etwas abweichend. Sie erscheinen gegenüber den π-Granula in ihrer Substanz gelockert, nicht in Stricheln und scharf umgrenzten Schollen, sondern in kleineren und größeren Ballen abgelagert. Sie finden sich interzellulär, am häufigsten und reichlichsten in den Gliazellen, in der Nachbarschaft der Gefäße und in mesodermalen Zellen des adventitiellen Lymphraumes und wandeln sich besonders in den letzteren rasch in mit basischen Farben unfärbbare oder sich gelblich oder grünlich färbende Massen um, die sich

auch mit Scharlach rot färben. Daß das Scharlach einen großen Teil des Zellinhalts rot färbt, wo auch das Toluidinblau erhebliche Mengen basophil metachromatischer Stoffe darstellt, mag darauf beruhen, daß in vielen Zellen schon eine Mischung beider Stoffe vorhanden ist. Die Eigentümlichkeit dieser Fettstoffe, von vornherein stark gelb zu erscheinen oder bei Anwendung blauer basischer Farben grünlich sich zu färben, dürfte dafür sprechen, daß es sich auch hier wieder um einen besondersartigen fettigen Stoff handelt. Diese Neigung ist so charakteristisch, daß man fast regelmäßig dort, wo man im Alkoholpräparat solche stark gelben oder grün gefärbten Massen in Zellen der adventitiellen Lymphräume findet, an Formolschnitten metachromatisch basophile Stoffe im Gewebe nachweisen kann.

Es erübrigt uns nun noch, das Verhältnis der Glia zu diesen Stoffen einer kurzen Betrachtung zu unterziehen. Das Auftreten derselben ist an andere Gliaformen gebunden, wie das Auftreten der früher beschriebenen Granula. In amöboiden Gliazellen sieht man sie nicht oder höchstens einmal andeutungsweise. Sonst sammeln sie sich in den Gliazellen und ihren Fortsätzen, die dann oft aufgetrieben erscheinen, an, ganz ebenso wie wir das von den mit Scharlach färbbaren fettigen Stoffen kennen. Mit der zunehmenden Anhäufung dieses Stoffes verliert die Zelle ihre Fortsätze und wird damit kugelig, der Kern rückt ganz wie bei den Fettkörnchenzellen an die Peripherie und zeigt dieselben regressiven Veränderungen wie in diesen. Öfters sehen wir auch in den Kernen der mit diesen Stoffen beladenen Zellen randständige große Chromatinschollen, während die Kernmembran selbst nur undeutlich oder gar nicht gefärbt erscheint. (Tafel XXXII, Fig. 9*a* und 11.)

Die Beobachtung, daß wir diesen Stoff am reichlichsten in den Zellen der Gefäßwand und dann in abnehmender Menge in den Gliazellen um die Gefäße und in den weiter ab von den Gefäßen gelegenen Zellen des Stützgewebes finden, spricht wieder dafür, daß diese Stoffe oder ihre uns nicht nachweisbare Vorstufen gefäßwärts transportiert werden. In den mesodermalen Zellen werden sie dann, soweit dies nicht schon in Gliazellen geschehen ist, vollständig in Fett umgewandelt.

Welche pathologische Bedeutung kommt nun diesen Stoffen zu? Wir müssen eine Antwort darauf suchen aus der Art ihres Vorkommens im zentralen Nervensystem und bei den verschiedenen Erkrankungsprozessen. Man findet nun diese basophil metachroma-

tischen Stoffe ganz sicher weniger in der grauen Substanz als in der weißen. Ich kann mich nicht erinnern, daß ich in irgend einem Falle nennenswerte Massen in der Hirnrinde über den Radii hinauf gefunden hätte. Dagegen begegnet man ihnen in größerer Menge in den Markleisten. Auch im Hirnstamm, wo man sie am allerreichlichsten antrifft, scheint ihr Vorkommen mehr auf die Markgegend beschränkt und in den grauen Massen vereinzelter. Ebenso begegnen wir ihnen im Rückenmark mehr in der weißen Substanz als in den Vorder- und Hinterhörnern, obwohl man auch hier gelegentlich in der grauen Substanz sie in ziemlicher Menge antrifft. Aber auch diese enthält ja reichlich Markfasern. Diese Anordnung ihres Vorkommens spricht jedenfalls dafür, daß sie Beziehungen zum Mark haben, was ja schon aus ihrem Vorkommen in den SCHWANNschen Zellen wahrscheinlich wird und auch von REICH angenommen worden ist.

Was nun ihr Vorkommen bei verschiedenen Krankheiten betrifft, so scheinen sie auch im normalen Gehirn nicht zu fehlen, jedenfalls aber sind sie dort viel seltener als in den normalen peripheren Nerven. Sie finden sich dann schon reichlicher in mancherlei Gehirnen, die nicht als pathologisch im engeren Sinne anzusehen sind, so bei älteren Leuten, im Gehirn von Phthisikern, auch im Gehirn eines schwer Anämischen sind sie in recht reichlicher Menge vorhanden gewesen. Noch größeren Anhäufungen dieser Stoffe begegnet man dann bei ausgesprochenen Geistesstörungen. Am allerreichlichsten habe ich sie in dem schon erwähnten Falle von schwerer manischer Erregung, dann auch bei Depressionszuständen des manisch depressiven Irreseins gesehen, weiter bei Infektionsdelirien, bei der Epilepsie, der progressiven Chorea und in ganz besonderer Menge auch im Mittelhirn bei der Dementia senilis und der Arteriosklerose. Auch in Fällen von Hirnlues, tuberkulöser Meningitis, in der Nachbarschaft von Geschwülsten und frischen Erweichungen und Blutungen waren sie hin und wieder anzutreffen. Manchmal fanden sich daneben amöboide Gliazellen und Erscheinungen eines Zerfalls von Nervengewebe. In einigen Fällen schwerster Rindenerkrankung mit zahlreichem Achsenzylinderzerfall sah man dagegen wieder keine Spur von ihnen. Ihr Vorkommen scheint also nicht an bestimmte Krankheitsformen gebunden, aber auch nicht notwendig an einen Zerfall von Markfasern. Dort, wo Markfasern in großer Menge abgebaut wurden, z. B. bei Seitenstrangdegeneration nach Kapselblutung, und wo sich außerordentlich zahlreiche Fettkörnchenzellen fanden, war manchmal nichts oder recht wenig von ihnen nachzuweisen, ebenso auch in einem Falle von Paralyse, wo Seiten- und Hinterstränge mit Körnchenzellen dicht angefüllt waren. Leider werden ausgedehntere vergleichende Untersuchungen dadurch etwas erschwert, daß wenigstens in den Formolpräparaten ein ziemlich schneller Zerfall dieser Stoffe stattzufinden scheint. Es ist mir wiederholt nicht mehr geglückt, einige Monate später an demselben Material die basophil metachromatischen Einlagerungen zu färben, wo sie sich vorher in der schönsten Weise hatten darstellen lassen.

Ich habe auch versucht, mit Hilfe von Tierexperimenten der Frage der Bedeutung dieser Stoffe auf den Grund zu kommen. Es war mir nun überraschend, wie wenig sich bei manchen Tieren davon finden ließ. Weder im gesunden, noch in dem durch die mannigfaltigsten experimentellen Eingriffe geschädigten Kaninchengehirn z. B. habe ich jemals eine Andeutung solcher Stoffe finden können, auch nicht in den SCHWANNschen Scheiden des peripheren Nerven. Auch bei vielerlei anderen Tieren habe ich sie nur in sehr geringen Mengen oder in Formen und mit Farbreaktionen gesehen, die ihre Übereinstimmung mit den π-Granulis des peripheren Nerven des Menschen nicht ganz sicher erscheinen ließen. Meine Untersuchungen sind nicht so erschöpfend, daß ich behaupten könnte, sie kämen bei irgend einem Wirbeltier überhaupt nicht vor. Einem Zweifel scheint es mir aber nicht zu unterliegen, daß sie dort vielfach eine geringere Rolle spielen. Jedenfalls aber ist es nötig, noch weitere Forschungen in dieser Richtung anzustellen, denn man wird über die Bedeutung dieser Stoffe am ersten aus dem Tierexperiment einen Aufschluß erhalten können.

Bis aber solche umfangreiche Untersuchungen ausgeführt sind, müssen wir uns aus unseren Befunden wenigstens ein vorläufiges Urteil zu bilden suchen. Da es feststehen dürfte, daß die metachromatisch basophilen Stoffe fehlen können oder in nur geringer Menge auftreten, wo Markscheiden zerfallen, dürften sie kein notwendiges Abbauprodukt der Markscheide sein, wie etwa die mit Scharlach färbbaren lipoiden Stoffe. Da sie aber auch dort nachweisbar sind, wo wir keinerlei Anhaltspunkte dafür haben, daß Markscheiden zerfallen, mit denen sie doch offenbar in Beziehung stehen, scheint es wahrscheinlich, daß sie auch ohne Zerfall der Markscheiden vorkommen. Da sie weiter in pathologischen Fällen zweifellos vermehrt sind, ist anzunehmen, daß sie wenigstens zum Teil unter

pathologischen Ernährungsbedingungen sich bilden. Denn daß sie im weiteren Sinne ein Abbaustoff sind, geht daraus hervor, daß sie in Fett umgewandelt und den Gefäßen zugeführt werden, ganz wie die anderen Abbaustoffe auch. Damit würden aber auch diese Stoffe den bisher beschriebenen gegenüber eine besondere Stellung einnehmen, ja sie würden ein ganz besonderes Interesse beanspruchen, da sie ein Beispiel darstellen würden für noch feinere Störungen im Nervengewebe, für die uns bis jetzt jedes anatomische Verständnis fehlt. So könnten also vielleicht die von Reich begonnenen Untersuchungen bedeutsame Fortschritte unserer Erkenntnis in Aussicht stellen.

Den Schlüssen dagegen, zu welchen Reich selbst durch seine Untersuchungen gekommen ist, kann ich nicht beipflichten. Was die Gliazellen im zentralen Nervensystem zu leisten haben, leisten in einer durch die Verhältnisse gegebenen Modifizierung die Schwannschen Zellen des peripheren Nerven. Sie bilden seine Hülle und Stützsubstanz, sie sind wohl auch am Aufbau der Markscheide beteiligt, wohl auch bei ihrer Ernährung, jedenfalls bei der Aufräumung jener, die zugrunde gehen. Sie stehen also in enger biologischer Beziehung, in einem sozusagen symbiotischen Verhältnis zur peripheren Nervenfaser. Daß in der Schwannschen Zelle verschiedenartige Granula nachzuweisen sind, bringt sie der Ganglienzelle nicht näher. Die Art der Granula läßt vielmehr ihre enge biologische Verwandtschaft zu den Gliazellen erkennen. So sprechen, glaube ich, die Befunde Reichs, wie unsere eigenen Beobachtungen nur dafür, daß die Schwannsche Zelle in biologischer Beziehung die Gliazelle des peripheren Nerven darstellt — wie sie nach den Ergebnissen der entwicklungsgeschichtlichen Untersuchung es auch nach ihrer Abstammung ist.

6. Über einfach basophile Abbaustoffe.

Neben den basophilen metachromatischen Stoffen finden wir bei verschiedenen akuten Erkrankungen des Nervengewebes noch andere basophile Stoffe, die sich nach ihren färberischen und morphologischen Eigenschaften von diesen und unter sich unterscheiden. So treffen wir nach Alkoholfixierung und -Färbung mit basischen Anilinfarben Produkte, welche die betreffende Farbe angenommen haben, ohne eine Neigung zur Metachromasie zu zeigen und deswegen als ein-

fach basophile bezeichnet werden mögen. In ihrer Neigung, sich mit basischen Farben, wie die Nisslsubstanz, zu färben, liegt eine der Schwierigkeiten ihres genaueren Studiums. Denn wenn sie in den Ganglienzellen selbst auftreten, ist es sehr schwierig, sie von der Nisslsubstanz zu unterscheiden. Alle Versuche, sie elektiv zu färben, sind bis jetzt mißglückt. Man findet die einfach basophilen Produkte recht häufig bei vielen akuten Krankheiten und experimentellen Vergiftungen. Offenbar treten sie schon bei leichteren Schädigungen der Ganglienzellen auf, begleiten aber oft auch die allerschwersten Degenerationsvorgänge der Nervenzellen, z. B. Nissls schwere Zellerkrankung. Ihr Vorkommen bei leichteren Erkrankungszuständen läßt sich am besten studieren unter experimentellen Verhältnissen. Nach subakuter maximaler Alkoholvergiftung beim Kaninchen z. B. findet man im zentralen Nervengewebe die Bahnen der im übrigen nicht oder kaum gefärbten Protoplasmafortsätze gekennzeichnet durch Reihen dunkelblauer Körnchen, die sich viel weiter durch das Nervengewebe hinziehen, als man normalerweise im Nisslpräparat die Protoplasmafortsätze verfolgen kann. Ein anderes sehr günstiges Objekt sind Hunde, die an Tetanie gestorben sind, nachdem man ihnen Schilddrüse und Epithelkörperchen entfernt hat. Die erste Vermutung, die man über die Natur dieser Körnchen haben kann, ist die, daß sie eine veränderte, in Körnchen umgewandelte Nisslsubstanz darstellen. Dagegen spricht aber, daß wir diese Körnchen auch dort sehen, wo keine Nisslschollen nachzuweisen sind, in den Protoplasmafortsätzen weitab von dem eigentlichen Zelleibe und im Achsenzylinderfortsatz, und daß manche dieser Körnchen gar nicht in den Zellfortsätzen liegen, sondern auf den Zellen, um sie herum oder weiter von den Ganglienzellen ab im Gewebe. Zuweilen sieht man ganz zarte Protoplasmafortsätze, wie mir scheint, auch Achsenzylinder, die sich in solche Körnerreihen aufzulösen scheinen, und schon öfters sind mir von Herren des Laboratoriums solche als Kokken demonstriert worden. Wo diese Stoffe nun auftreten, begegnen wir ihnen auch außerhalb der Ganglienzellen; wir finden sie in den Gliazellen und fast immer auch, dann gar nicht selten in größere Klumpen zusammengeballt, in perivaskulären Räumen. Offenbar übereinstimmenden Stoffen begegnen wir dann, manchmal in ungleich größeren Mengen, bei verschiedenen schweren Psychosen. Tafel XXXIII, Fig. 7 stellt eine Beetzsche Pyramide von einer galoppierenden Paralyse dar, die im übrigen nach der Kern-

veränderung und dem schon vorgeschritteneren Zerfall benachbarter Zellen zur schweren Zellveränderung Nissls zu rechnen ist. Ganz oben am Spitzenfortsatz und an einem in der Nähe gelegenen Protoplasmafortsatz, der wahrscheinlich nicht zur Zelle gehört, liegen in ziemlich gleichen Abständen nahezu runde, basophile Körnchen. Es läßt sich schwer sagen, ob sie innerhalb des Protoplasmas oder auf demselben liegen. Mehr nach abwärts sehen wir solche Körnchen, die sicher außerhalb der Zelle und auf ihr gelagert sind. An der Basis der Zelle, um den Achsenzylinderfortsatz und auf dessen Ursprungskegel, bis ein gutes Stück über den Zelleib hinweg, liegen große schollen- und tropfenartige Gebilde der gleichen Substanz. Über dem eigentlichen Zelleib werden sie wieder kleiner. Es wird hier unmöglich, zu entscheiden, ob es sich um in der Zelle gelegene, in der Form veränderte Nisslschollen, oder um außerhalb der Zelle abgelagerte pathologische Produkte handelt. Man wird erst noch eine Färbemethode finden müssen, die beide Substanzen in verschiedener Färbung darstellt. Die gleichen Stoffe sehen wir dann auch an den amöboiden Trabantzellen, welche die Ganglienzellen umlagern. In anderen Zellen liegen sie sicher auch innerhalb des Plasmas der Gliazellen.

Fig. 12 der gleichen Tafel zeigt uns dieselben Stoffe von einem Falle urämischen Delirs: bei a) sehen wir kokkenähnliche Reihen, bei c) einen Teil des Plasmaleibes einer Ganglienzelle, die sich offenbar in solche Stoffe auflöst, und bei d) einen anderen Teil einer Zelle, bei b) einen rundlichen Ballen aus einem perivaskulären Raum, in welchem die gleichen basophilen Substanzen in eine fast ungefärbte homogene Grundmasse eingebettet sind.

Im Gegensatz zu den metachromatisch basophilen Stoffen, die wir vorzugsweise im Mark antreffen, begegnen uns die einfach basophilen hauptsächlich in der grauen Substanz, vorzugsweise um die Ganglienzellen herum und um ihre Protoplasmafortsätze. Man wird sich nun die Frage vorlegen müssen, ob dies Produkte sind, die etwa aus den Gewebssäften an diesen Stellen deponiert werden oder ob sie dort, wo wir sie finden, aus dem Zerfall vorhandener Gewebsstrukturen hervorgehen. In sehr vielen Fällen kann man aufs deutlichste wahrnehmen, wie an den Stellen ihres Vorkommens die Protoplasmafortsätze besonders schwere Schädigungen zeigen und auch die Ganglienzellen Andeutungen von Verflüssigung erkennen lassen. Bilder wie Fig. 12*c* beweisen aufs deutlichste, daß sie mit dem Zerfall des Zellplasmas entstehen. Die Möglichkeit scheint nicht ausgeschlossen, daß sie an Stellen, wo wir sie bei verhältnismäßig noch intakten Protoplasmafortsätzen und Zellen auf diesen liegend finden, aus dem Zerfall der perizellulären nervösen Bildungen hervorgehen. Da es mir nicht gelungen ist, sie an Material darzustellen, das in Formol fixiert war, auch nicht mit der Säurefuchsinlichtgrünmethode an Flemmingschnitten, scheint es mir fraglich, ob sie in der Form, in der wir sie in den Schnitten sehen, im Leben vorkommen und ob sie nicht vielleicht erst durch die Fixierung in Alkohol in die Formen gebracht werden, die uns die Alkoholschnitte zeigen. Auch der Umstand, daß man sie häufig in großen tropfenartigen Bildungen vorfindet, wie sie die Fig. 7 auf Tafel XXXIII zeigt, dürfte dafür sprechen, daß sie sich während des Lebens in einem flüssigen oder halbflüssigen Zustande befinden.

Nicht ganz leicht ist das Verhältnis dieser einfach basophilen Produkte zu den Gliazellen darzustellen. Man findet sie sehr häufig nur um die Gliazellen herum liegen, doch oft auch so, daß es schwer zu entscheiden ist, ob sie nur auf oder auch in dem Zellenplasma gelegen sind. Hin und wieder aber findet man Gliazellen, die diese Stoffe auch in ihrem Plasma enthalten. Jedenfalls scheinen sie auch wieder den Weg in die perivaskulären Räume zu finden, wo sie oft in außerordentlichen Massen zusammenliegen. Auch hier finden wir sie häufig in größere Ballen eingebettet, die selbst nur wenig gefärbt sind. In die mesodermalen Zellen scheinen sie in der Form, in der man sie im Nervengewebe beobachtet, nicht zu gelangen. Da man aber dort, wo sie sehr zahlreich sind, die Zellen der Adventitia sehr reich mit lipoiden Stoffen beladen sieht, ist es möglich, daß sie in diesen Zellen in fettige Stoffe umgewandelt werden.

Derartige einfach basophile Stoffe findet man nun nicht nur bei ganz akuten Erkrankungsprozessen, sondern manchmal auch in ganz verödeten Rinden und um Ganglienzellen herum, die schon völlig abgestorben sind. Sie zeigen dann häufiger eckige, harte Formen, und es muß noch weiteren Untersuchungen vorbehalten bleiben zu entscheiden, ob sie mit den bei akuten Erkrankungsprozessen auftretenden völlig übereinstimmen und vielleicht nur durch die längere Ablagerung mehr geschrumpft und fester im Gefüge geworden, oder ob sie überhaupt anderer Art sind. Zum Teil dürften sich die beiden Formen basophiler Produkte, besonders die letztere, mit dem, was von Nissl früher als das „Sichtbarwerden der perizellulären Hosen“ beschrieben worden ist, decken.

Neben den eben beschriebenen finden wir in den Gliazellen des kranken Nervengewebes noch mehrerlei andere basophile Einschlüsse: solche, die keine Neigung zu metachromatischer Färbung zeigen und solche, die etwas metachromatisch gefärbt sind, wenn auch nicht in so ausgezeichneter Weise wie die eigentlich metachromatisch basophilen. Bald sind es einzelne runde Körnchen von verschiedener Größe, die in rundlichen Zellen liegen, von denen sonst oft nur der Kern und ein leicht gefärbter Zellrand hervortritt, während um den Kern herum eine große Lücke zu beobachten ist (Tafel XXXIII, Fig. 3 *h—m*); bald sind es schollenartige Bildungen, bald mehr schaumartige Strukturen (Tafel XXXIII, Fig. 3 *a—g*). Manchmal finden sie sich in recht erheblicher Menge, besonders in den Zellen des Markes, und da man dann zuweilen fast in allen Zellen den gleichen Einlagerungen begegnet, dürfte ihnen auch eine besondere pathologische Bedeutung zukommen. Einstweilen läßt sich darüber aber nichts bestimmtes sagen.

Schließlich gehören hierher wohl auch noch die eigenartigen, bei der NISSLmethode sich sehr schön abhebenden Stippchen, die man in gewucherten Gliazellen und Gliarasen antrifft und auf die NISSL aufmerksam gemacht hat. Auch über die Bedeutung dieser Plasmaeinlagerungen weiß ich nichts als daß sie von den bisher erwähnten offenbar abweichen.

7. Über einige extrazelluläre pathologische Stoffwechselprodukte.

Schon früher haben wir im akut erkrankten Nervensystem eine Form extrazellulärer Gebilde kennen gelernt, deren Auftreten eng an das Vorkommen amöboider Zellen gebunden scheint, und die wir als Zerfallsprodukte gliöser Strukturen aufgefaßt haben — die Füllkörperchen. Neben diesen gibt es nun noch mancherlei andersartige pathologische extrazelluläre Gebilde. Manche davon zeigen ein viel verbreitetes Vorkommen und sind offenbar nicht an akute Krankheitsprozesse und an das gleichzeitige Vorhandensein amöboider Zellen gebunden.

Man wird sich bei dem Studium dieser extrazellulären Bildungen fast immer die Frage vorlegen müssen, ob sie in der Form, in der wir sie in den Präparaten sehen, auch im Leben schon vorhanden sind, oder ob sie erst die Härtungsflüssigkeiten aus einem

flüssigen Zustande in feste Formen gebracht haben. Für die Entscheidung dieser Frage scheint von großer Bedeutung das Verhalten der Glia ihnen gegenüber. Wenn wir sehen, daß die Glia sie wie Fremdkörper behandelt und eigene Strukturen um sie herum bildet, dürfte es sicher sein, daß sie schon im Leben in der Form vorhanden waren, in der wir sie bei unserer Untersuchung vorfinden. Läßt die Glia jede Reaktion ihnen gegenüber vermissen, so muß die Frage offen bleiben. Denn es mag Stoffe geben, die so wenig anreizend auf das Stützgewebe wirken, daß es ihnen gegenüber keinerlei Reaktion zeigt, wenn sie wohl auch die selteneren sein dürften.

Die bekanntesten dieser pathologischen Stoffwechselprodukte des zentralen Nervengewebes sind die Corpora amylacea, denen wir auch hier eine kurze Betrachtung widmen müssen. Über sie existiert bereits eine reiche Literatur, und die Bemühungen nehmen kein Ende, ihre Entstehung von irgendwelchen Elementen des zentralen Nervengewebes abzuleiten. Die Corpora amylacea finden sich bekanntermaßen auch im normalen Nervensystem, besonders unter den Oberflächenschichten der Glia, jedenfalls aber stark vermehrt im chronisch degenerativ veränderten Nervengewebe: in den Hintersträngen der Tabes, in den Randpartien des Rückenmarks der senilen Demenz, in dem verdickten Gliafaserfilz der Rindenoberfläche, in der Nachbarschaft alter arteriosklerotischer Herde, um die degenerierten Gefäße der Stammganglien bei der Arteriosklerose. Hier finden sie sich oft in geradezu ungeheurer Anhäufung. Aber sie können sich auch oft außerordentlich rasch bilden, und man begegnet ihnen z. B. bei einer tuberkulösen Meningitis des Rückenmarks von nur wenigen Tagen Dauer in ganz außerordentlicher Menge in den Gegenden, in welchen der meningomyelitische Prozeß die schwersten Veränderungen gesetzt hat. In einem Falle eines schweren Intoxikationsdelirs bei einer jugendlichen Person, das nur wenige Tage gedauert hatte, lagen sie an vielen Stellen der Hirnrinde in mehreren Schichten übereinander unter der Gliarandschicht. Zwischen je zwei Gliafüßchen, die sich in die Grenzmembran einsenkten, lag eines dieser Körperchen. Sie gehören also auch mit zu dem Bilde mancher akuten Erkrankungsprozesse des Zentralnervensystems. In solchen frischen Fällen findet man Corpora amylacea zwischen den Resten zerfallender Achsenzylinder, sogar mitten in den körnigen Massen, in welche sie sich auflösen. Man begegnet ihnen auch gar nicht selten im Protoplasmaleib wuchernder amöboider Gliazellen. Entstehen sie

nun aus den Achsenzylindern und Gliazellen? Ihre besondere Anhäufung unter der Membrana superficialis der Glia, ihr massenhaftes Vorkommen unter der Membrana perivascularis größerer Gefäße (Textfigur 8) läßt wohl ausschließen, daß sie aus nervösen Elementen hervorgehen, wie das schon OBERSTEINER betont hat. Gerade dort,

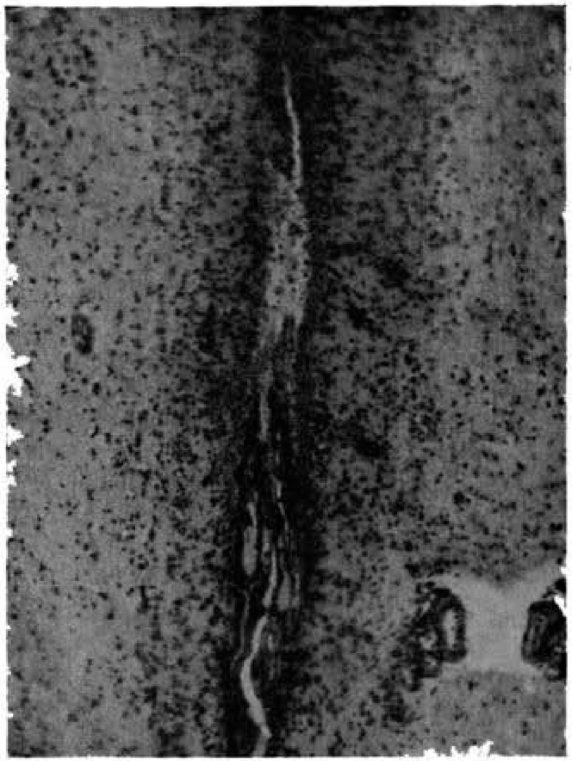

Fig. 8. Anhäufung von Corpora amylacea um ein Gefäß des Thalamus opticus eines Falles von Arteriosklerose. WEIGERTsches Eisenhämatoxylin, VAN GIESON-färbung.

wo sie offenbar ganz akut in reichlichster Zahl und in der Gliarandschicht der Hirnrinde entstanden sind, lag keine einzige innerhalb einer Gliazelle. Das macht auch ihre Entstehung aus der Glia unwahrscheinlich. Der Umstand aber, daß sie gerade vor den als Filterflächen zu betrachtenden Grenzmembranen in besonderer Menge

sich ablagern, spricht sehr dafür, daß sie aus den Gewebssäften hervorgehen, gleichsam aus ihnen niedergeschlagen werden. Die Glia behandelt sie dann, wie sie andere Fremdkörper zu behandeln pflegt. Wenn sie sich in Gliazellen selbst finden, dann sind sie von ihnen umflossen worden, und die deutlichsten gliösen Grenzhäutchen, die sich demonstrieren lassen, sind die zarten Hüllen, in welche die Glia die Amyloidkörperchen schließlich einbettet. Im Säurefuchsinlichtgrünpräparat kann man dies aufs schönste darstellen, da sich hier die Corpora amylacea grün und diese Grenzhäutchen rot färben (Textfigur 9 *A*). Manchmal beteiligen sich auch die Gliafasern an der Abkapselung.

Offenbar gehen in den amyloiden Körperchen, sobald sie im Gewebe deponiert sind, noch weitere stoffliche Umwandlungen vor sich. Das zeigt schon ihr außerordentlich verschiedenartiges Verhalten gegenüber verschiedenen Färbungsmethoden. Mit basischen Anilinfarben sind sie teils nicht, teils blaß, teils außerordentlich dunkel gefärbt. Mit Hämatoxylin färben sie sich teils kaum, teils tief dunkelblau. Sehr häufig zeigt sich, daß sie in ihren verschiedenen Schichtungen eine verschiedene Farbe annehmen. Demnach müssen wir wohl in den Corpora amylacea Produkte sehen, die aus der Gewebsflüssigkeit und zwar schon im Leben ausgefällt werden. Worin eigentlich ihre Bedeutung zu sehen ist, bleibt unklar.

Wenn wir uns nun weiter umsehen, so stoßen wir im pathologischen Nervengewebe noch auf mancherlei abweichende, aber ähnliche Dinge. So sehen wir oft im NISSL-Präparat das ganze Gewebe durchsät von ganz kleinen, meist rundlichen, hin und wieder auch ovalen, seltener unregelmäßiger begrenzten Körperchen, kaum von der Größe eines Ganglienzellkernkörperchens, die sich manchmal intensiv mit basischen Farben färben, manchmal kaum etwas Farbe annehmen. Sie geben nicht die Reaktionen der Corpora amylacea. Einige Male habe ich sie im Hinterhorn des Rückenmarks von Alkoholisten, dann auch wieder in der Hirnrinde von frischen und älteren Paralysen in ganz außerordentlicher Menge gesehen. Soweit sich beobachten läßt, scheint sich die Glia nicht um sie zu bekümmern. Vielleicht werden sie auch erst durch die Härtungsflüssigkeiten aus dem Gewebe ausgefällt.

Wahrscheinlich verhält es sich auch ähnlich mit dem Glykogen, das ich einige Male in und unter der Pia, einige Male auch in der Hirnrinde, z. T. wohl auch in Gliazellen eingeschlossen, oft nur den-

selben angelagert, meist aber in den adventitiellen Lymphräumen in zahlreichen Körnchen und tropfenartigen Gebilden, besonders bei Paralysen und infektiösen Delirien gefunden habe. Neuerdings hat es CASAMAJOR im OBERSTEINERschen Laboratorium auch in Ganglienzellen der Hirnrinde selbst nachgewiesen. Die pathologische Bedeutung des Glykogens und sein Vorkommen bei den verschiedenen Krankheitszuständen muß jedenfalls noch durch weitere Beobachtungen klargestellt werden.

Zu den extrazellulären Gebilden pathologischer Art gehören dann auch noch die von FISCHER eingehender beschriebenen Drusen, die er als charakteristisch für die senile Demenz betrachtet. Wie die Arbeit von PERUSINI und eine im hiesigen Laboratorium ausgeführte Untersuchung von SIMCHOWICZ über die Dementia senilis überzeugend dartun, ist das Wesentlichste in den Drusen die Ablagerung eines pathologischen Stoffwechselprodukts in das wahrscheinlich vorher veränderte Gliaretikulum. Die Veränderungen an den Nervenzellen und Achsenzylindern sind wohl als sekundäre zu betrachten. Die Gliazellen der Nachbarschaft bemühen sich, die abgelagerten Fremdkörperstoffe nach Möglichkeit abzukapseln, und so entstehen dann schließlich die etwas kompliziert gebauten Drusen, die einem tieferen Verständnis mancherlei Schwierigkeiten entgegensetzen. Da in jenen Arbeiten dargelegt ist, daß diese Stoffe andere Farbreaktionen zeigen als alle anderen pathologischen

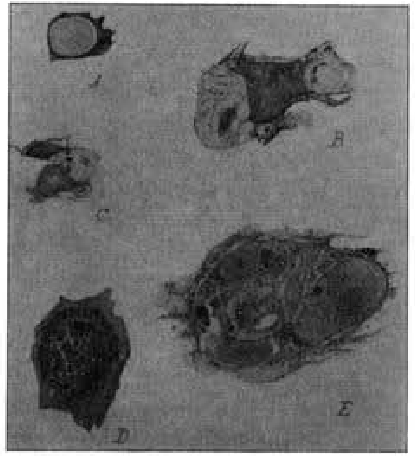

Fig. 9. Verschiedenartige extrazelluläre pathologische Bildungen. *A* Amyloidkörperchen von einer Gliahülle umgeben. *B*, *C* Zwischen den Markscheiden gelegene häufige Bildungen. *D* Mit fuchsinophilen Granula vollgestopfte Gebilde. *E* In der Wand einer Kapillare und perivaskulär abgelagerte Schollen und Brocken eigenartiger Substanzen. S-Fuchsinlichtgrün. Die dunklen Stellen sind im Präparat rot, die hellen grün gefärbt.

Stoffe, die bisher die pathologische Anatomie des Gehirns kennen gelehrt hat, müssen wir sie auch wieder als Produkte besonderer Art betrachten.

Bei einigen anderen Stoffen, die unkonstanter und seltener vorzukommen scheinen, müssen erst weitere Untersuchungen feststellen, ob ihnen eine größere Bedeutung zukommt. So bemerkt man öfters in Säurefuchsin-Lichtgrünpräparaten, besonders in der grauen Substanz, rundliche Haufen von der Größe einer mittleren Ganglienzelle, die ganz aus dicht beieinander liegenden, sehr kleinen, rotgefärbten Körnchen von etwas unregelmäßiger Form und Größe bestehen. Andere solche Ballen zeigen einen grünlichen Grundton, sind aber auch übersät von feinsten roten Körnchen. Ihre Gestalt ist unregelmäßiger, zuweilen länglich, bisquitförmig, öfters liegen in der Nachbarschaft der größeren kleinere Haufen, die dann meist durch eine große Ansammlung kleiner Körnchen eine dunkelrote Farbe haben. Offenbar wieder andere Gebilde erinnern in ihrer Größe und Form an große amöboide Zellen, sind aber etwas eckiger in ihrer Begrenzung. Die Peripherie ist grün gefärbt, das Zentrum von einer dichten Anhäufung roter Granula gebildet, zwischen welchem nichts von einer grünen Färbung mehr sichtbar ist (Textfigur 9 *D*). Einen Kern habe ich niemals in ihnen finden können; es scheint mir auch deshalb nicht, daß sie etwa zerfallende amöboide Gliazellen darstellen, weil sie sehr häufig wesentlich größer sind als alle Gliazellen, die sich im gleichen Präparate finden. Verhältnismäßig häufig sind dann noch andere, mir ebenso unverständliche Gebilde, die man besonders zwischen Markscheiden antrifft, und die die Größe des Querschnitts der größten derselben erreichen können, aber auch wieder in ganz minutiösen Formen auftreten (Textfigur 9 *B*, *C*). Ihre Gestalt ist sehr unregelmäßig, selten rund, meist mehr quadratisch, aber sehr häufig mit eingedrückten Seitenflächen. Sie färben sich bei der Säurefuchsin-Lichtgrünfärbung schmutziggrün und enthalten meist am Rande, oft auch in ihrer Mitte rote Stäubchen. Von einem Kern ist nie etwas in ihnen wahrzunehmen. Wahrscheinlich wieder etwas anderes stellen aus grünen und roten Körnchenmassen gebildete Haufen dar, die sich gewöhnlich um Gefäße herum abgelagert finden, manchmal auch noch die verdickte Gefäßwand durchsetzen (Textfigur 9 *E*). Man müßte, wenn man die Entstehung und Bedeutung dieser zuletzt beschriebenen Gebilde erforschen wollte, zunächst noch andere Färbungsmethoden anwenden, um festzustellen, ob sie auch anderen Farbgemischen

gegenüber sich gleich verhalten, oder ob sie — was mir wahrscheinlicher scheint — unter sich verschiedener Art sind. Ihr selteneres Vorkommen aber spricht dafür, daß sie für den pathologischen Stoffwechsel der Hirnrinde doch nur ein nebensächlicheres Interesse haben. So mag es genügen hervorzuheben, daß sich noch mancherlei andere Produkte bei der Degeneration des Nervengewebes bilden, über die wir heute nur sehr wenig sagen können.

8. Besonderen Krankheitsprozessen eigentümliche Abbauprodukte.

Während die pathologischen Stoffe, die wir bisher besprochen haben, sehr verbreitet bei sehr verschiedenartigen Erkrankungen des Zentralnervensystems vorkommen, so daß sie als regelmäßige Erscheinungen des Zerfalls von Nervengewebe oder pathologischer Stoffwechselstörungen im Zentralnervensystem angesehen werden müssen, scheint das Auftreten anderer beschränkt auf eine einzelne Krankheitsform oder eine Gruppe zusammengehöriger Krankheiten. Wir müssen daher wohl annehmen, daß sich hier der Abbauvorgang in einer von der gewöhnlichen abweichenden Form abspielt. Bis jetzt kennen wir als hierher gehörig nur die amaurotischen Idiotien und einen einzelnen paralyseähnlichen Erkrankungsfall, der von Herrn Dr. Baroncini im hiesigen Laboratorium eingehender untersucht worden ist. Möglicherweise dürfte auch hierher zu stellen sein ein von Sträussler beschriebener Fall von kongenitaler Kleinhirnatrophie. Außerdem habe ich aber noch von einigen anderen, auch klinisch von diesen abweichenden Fällen einzelne Präparate gesehen, die wieder andersartige Stoffe oder in einem andersartigen Vorkommen zu enthalten schienen. Nur fehlte es mir an Material, eingehendere Prüfungen anzustellen oder es stand mir zu einer Zeit zur Verfügung, in welcher mir derartige Untersuchungen noch fern lagen und ist jetzt für eine weitere Verarbeitung unbrauchbar geworden. So ist es wohl wahrscheinlich, daß die weitere Erforschung der Geistesstörungen, insbesondere die der angeborenen idiotischen Zustände, noch mehrere hierher gehörige, durch das Vorkommen besonderer Stoffe gekennzeichnete Krankheiten kennen lehren wird, und es ist wohl auch nicht unwahrscheinlich, daß wir Stoffe, die wir heute nur von einer einzigen Krankheit kennen, auch noch bei

verwandten Krankheiten, vielleicht in abweichender Anordnung finden werden.

Von der amaurotischen Idiotie kennen wir zwei verschiedene Formen, von denen die eine von Warren-Tay-Sachs zuerst beschrieben und in ihren histologischen Besonderheiten von Schaffer am genauesten studiert, die andere von Spielmeier zuerst klinisch und anatomisch untersucht und dann noch von Vogt geschildert worden ist. Kennzeichnend ist zunächst für die Warren-Tay-Sachssche Form das massenhafte Auftreten von Körnchen eines eigenartigen Stoffes in den Ganglien- und Gliazellen, der hier offenbar die Stelle jenes lipoiden Stoffes vertritt, der bei der verbreiteten fettigen Degeneration der Ganglienzellen zu finden ist. Das Plasma der Ganglienzellen wandelt sich unter mächtiger Anschwellung der Teile, in welchen dieser Stoff auftritt, um. Dabei sehen wir Formveränderungen der Ganglienzellen sich einstellen, wie wir sie auch bei der gewöhnlichen fettigen Degeneration, allerdings in weit geringerem Grade, beobachten: birnförmige Auftreibung des Zellkörpers, sackartige Vorwölbungen des Plasmaleibes, spindelförmige Anschwellungen der Plasmafortsätze und des Achsenzylinders, Verschiebung des Kernes, Hervortreten eines plasmatischen Gerüstes in der Zelle im Nissl- und Bielschowsky-Bild an der Ablagerungsstelle der Körnchen, Randstellung der Fibrillen, Bildung korbartiger Fibrillengerüste auf der Oberfläche der Zellen und den Auftreibungen der plasmatischen Fortsätze und der Achsenzylinder, sowie schließlich Umwandlung der ganzen Ganglienzelle in mit diesen Stoffen vollgepfropfte Säcke und Zerfall der Zellen. Neben einer Produktion riesiger faserbildender Gliazellen und feinfaseriger Gliaanordnungen durch die ganze Hirnrinde sehen wir auch in den Gliazellen Anhäufungen der gleichen Stoffe wie in den Ganglienzellen, wobei sich schließlich viele derselben in Körnchenzellen umwandeln, während andere nur in der Peripherie des stark vergrößerten Zelleibes solche Einlagerungen enthalten. Daneben finden sich reichliche Körnchenzellen in den oft sehr erweiterten und durch zahlreiche bindegewebige Septa sehr kompliziert gewordenen adventitiellen Lymphscheiden.

Die Spielmeier-Vogtsche Form scheint sich zunächst durch das Auftreten geringerer Massen dieser Stoffe von der Warren-Tay-Sachsschen zu unterscheiden. Daneben dürften mancherlei andere Abweichungen in den histologischen Veränderungen, welche uns hier weniger interessieren, es rechtfertigen, sie als eine besondere

Form der WARREN-TAY-SACHSschen gegenüber zu stellen. Viele klinische und anatomische Übereinstimmungen aber lassen es angebracht erscheinen, beide als amaurotische Idiotien in eine Gruppe zusammen zu fassen.

Bei der Betrachtung der Ganglienzellenveränderungen mußte man zunächst daran denken, daß es sich hier um eine fettige Degeneration handelt, die ihrem Wesen nach der senilen Fettdegeneration entspricht und sie nur dem Grade nach noch übertrifft. Bei einer genaueren Betrachtung nun aber zeigt sich, daß dieser Stoff von den lipoiden Stoffen, welche wir sonst in den Ganglienzellen finden, nach mancherlei Richtung hin abweicht. Schon an dem ersten Material, welches ich vor 6 Jahren durch die Freundlichkeit des Herrn Prof. SCHAFFER erhalten hatte, war mir aufgefallen, daß sich in den Ganglienzellen mit der HERXHEIMERschen Methode nicht eine den pathologischen Einlagerungen entsprechende Menge mit Scharlach geröteter Stoffe nachweisen ließ. Außer zerstreuten feineren Fettkörnchen, welche die Menge der körnigen Einlagerungen nicht erschöpften, zeigte sich höchstens eine ganz unbestimmt mattrote Färbung des übrigen pathologischen Zellinhalts. Dagegen waren mit Scharlach färbbare Stoffe sehr reichlich eingelagert in den Gliazellen und in den Zellen der Adventitia. Auf Tafel XXXIV, Fig. 5 ist ein solches Bild wiedergegeben. Die Zellen, die mit feinen Fettkörnchen nur wie bestäubt erscheinen, entsprechen Ganglienzellen, die Zellen mit großen Fettropfen stellen Gliazellen dar und schließlich finden sich große Fettanhäufungen in den Zellen des adventitiellen Lymphraumes. Ganz ähnlich verhielten sich nach voraus-

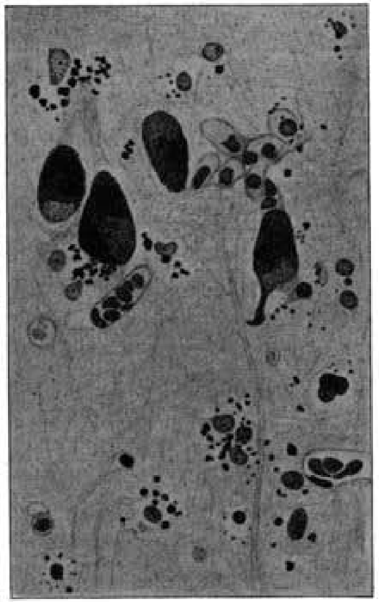

Fig. 10. Elektive Darstellung der Granula in einem Falle der SPIELMEYER-VOGTschen amaurotischen Idiotie mit Methode III.

gegangener Chromierung mit Osmium behandelte Präparate. In den Ganglienzellen waren nur spärliche feine Fettröpfchen oder überhaupt keine nachzuweisen, etwas mehr in den Gliazellen, reichlich in den Zellen der Adventitia. Die Einlagerungen der Ganglienzellen, welche sich mit Scharlach nicht färbten, konnten schließlich durch verschiedenerlei andere Methoden zur Darstellung gebracht werden. Besonders brillant waren Bilder, welche nach Methode III hergestellt worden sind. Die Textfigur 10 gibt ein solches Bild wieder, das von der SPIELMEIER-VOGTschen Form der amaurotischen Idiotie gewonnen worden ist, von der ich Material durch die Freundlichkeit des Herrn Prof. VOGT erhalten habe. Die Einlagerungen treten jetzt in den Ganglienzellen in weit größerer Menge, oft den größten Teil des Zelleibes, manchmal auch die Fortsätze erfüllend, leuchtend blau gefärbt hervor; in den Gliazellen sehen wir dieselben Stoffe in Form von Körnchenhaufen um den Kern herum gelagert. Bei der SACHSschen Form sind sie noch außerordentlich viel reichlicher vorhanden. Sie erfüllen die ganze Ganglienzelle und manche Gliazellen, welche Körnchenzellenform angenommen haben, und sind in anderen großen fasernbildenden Gliazellen in der Peripherie des protoplasmatischen Zelleibes gelegen. Ja sie scheinen sich an verschiedenen Stellen sogar als kleine Körnchen in einem Netz eingelagert zu finden, welches wohl ein Gliareticulum darstellen dürfte. Weiter gelang es mit mancherlei Modifikationen der WEIGERTschen Markscheidenfärbung, diese Einlagerungen nahezu elektiv darzustellen. Die Textfigur 11 gibt die Photographie eines solchen Bildes wieder. Alle die dunklen Klumpen sind mit diesen Stoffen beladene Zellen. Die enormen Mengen, in welchen sich diese Stoffe bei der SACHSschen Form im Nervengewebe finden, wird dadurch sehr gut veranschaulicht. An Material der SACHSschen Form, das ich der Liebenswürdigkeit des Herrn Prof. ADOLF MEYER in Baltimore verdanke, und das offenbar von einem noch vorgeschritteneren Krankheitsfalle herrührt, fanden sich etwas reichlicher mit Scharlach färbbare Stoffe in den Ganglienzellen. Aber auch sie erschöpften die Menge der stärker lichtbrechenden Körnchen nicht, die in den Zellen zu finden waren. Hier zeigten sich die Stoffe auch häufig nicht in Form von Körnchen, sondern in strichelartigen Bildungen, oft zwiebelschalenartig wie die π-Granula REICHS zu großen rundlichen Konglomeraten zusammengeordnet. Da das Material schon länger in Formol gelegen hatte, ließ sich nicht ausschließen, daß es sich hierbei um Veränderungen

handelte, die erst später in der Konservierungsflüssigkeit eingetreten waren. Man wird also erst noch an frischem Material nachsehen müssen, ob diese morphologischen Besonderheiten nicht Kunstprodukte sind; überhaupt ist es eine Eigentümlichkeit dieser Stoffe, sich in Formol allmählich zu verändern und auch manche ihrer ursprüng-

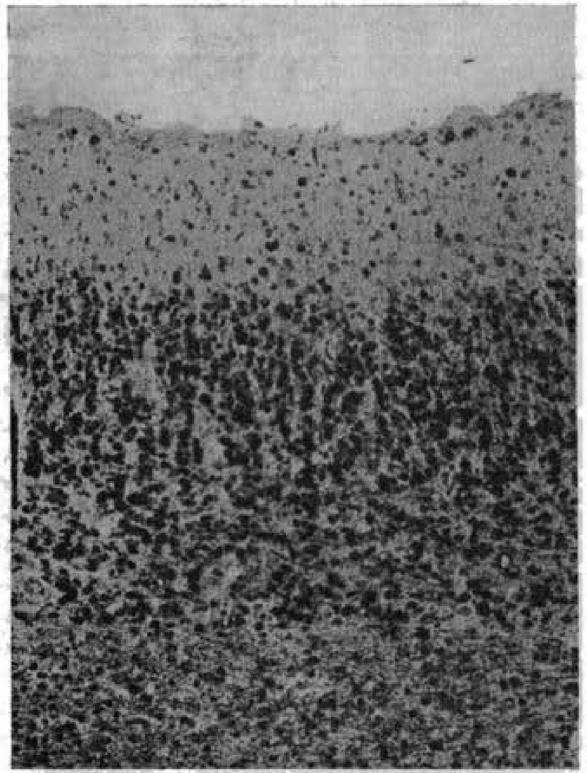

Fig. 11. Elektive Darstellung der Einlagerungen der WARREN-TAY-SACHSschen amaurotischen Idiotie. Hirnrinde. WEIGERTsche Markscheidenfärbung. Methode VIII.

lichen färberischen Reaktionen einzubüßen. Das erschwert natürlich erheblich bei der Seltenheit des Materials die Feststellung der besonderen Qualitäten dieser Stoffe. Weiter gelang auch ihre Darstellung gut mit der von BENDA zur Gliafaserfärbung angegebenen Methode ε. Dabei zeigten sich manche Körnchen rot, manche blau

gefärbt, während andere eine rote Schale und eine blaue zentrale Masse erkennen ließen.

SPIELMEIER hat sich dann eingehender mit den Einlagerungen seiner Form der amaurotischen Idiotie beschäftigt. Auch er hebt manche Ähnlichkeiten mit den senilen fettigen Einlagerungen hervor, namentlich auch die gelbliche Färbung, betont aber, daß das Fehlen der Osmiumreaktion gegen die Übereinstimmung beider Formen von Einlagerungen spreche.

Schließlich hat dann SCHAFFER eine Färbung angegeben, welche ungemein einfach ist und in einer kurzen Färbung der Formolschnitte mit EHRLICHschem Hämatoxylin besteht. Alle Zellen, welche diese Stoffe enthalten, treten dabei dunkelblau gefärbt hervor. An meinem alten Material sah man bei kurzer Färbung an vielen Stellen keine deutliche Körnchen, sondern nur wolkige blaue Massen, färbte man aber mehrere Stunden, so wurden die einzelnen Körnchen besser sichtbar. Behandelt man nun solche Formolschnitte erst mit EHRLICHschem Hämatoxylin und dann eine halbe Stunde mit alkoholischer Sudanlösung, so sieht man die Ganglienzellen fast völlig blau gefärbt und nur vereinzelte rote Körnchen in der blauen Masse; die Gliazellen dagegen zeigen neben den blauen Körnchen viele größere rote, ja manche sind ganz rot gefärbt. Recht oft liegen eine oder mehrere ganz rot gefärbte Gliazellen in Einbuchtungen des Zelleibes einer vollständig blau gefärbten Ganglienzelle.

Wenden wir nun die gleichen Methoden auf andere Rinden an, z. B. der senilen Demenz, so finden wir dort andere Farbreaktionen der lipoiden Körnchen. Sie färben sich wohl auch mit manchen Modifikationen der WEIGERTschen Markscheidenfärbung, jedenfalls aber nicht mit dem MAY-GRÜNWALDschen Farbstoff und mit EHRLICHschem Hämatoxylin. Dagegen färben sich die Körnchen der amaurotischen Idiotie nicht mit Scharlach und Osmium nach Chromierung, während die der senilen Demenz sich sehr intensiv färben. Bei Anwendung der Karbolfuchsin-Methylenblaufärbung, bei welcher sich die lipoiden Körnchen der senilen Ganglienzellenveränderung lebhaft rot färben, färbten sich die Einlagerungen der Ganglienzellen der amaurotischen Idiotie nur sehr matt rot, teilweise nahmen sie auch gar keine rote Farbe an, die Gliazellenkörnchen dagegen waren lebhafter rot gefärbt, die in Körnchenzellen umgewandelten Gliazellen leuchtend rot. Mit der Fibrinmethode konnte ich die Körnchen der amaurotischen Idiotie nicht darstellen. Wenn nun auch

diese Reaktionen nicht alle an frischem Material ausgeführt werden konnten, so daß sie nochmals an solchem nachgeprüft werden müssen, so ergeben sich doch jedenfalls so wesentliche Unterschiede, daß ihre verschiedene chemische Beschaffenheit festgestellt sein dürfte. Daß die Einlagerungen bei den beiden Formen der amaurotischen Idiotie gleicher Art sind, scheint mir nach den vielen ihnen gemeinsamen Farbenreaktionen sehr wahrscheinlich. Dem Umstand, daß bei der SACHSschen Form die Körnchen meist keine Gelbfärbung zeigen und sich leichter mit WEIGERTschem Hämatoxylin färben, möchte ich kein allzu großes Gewicht beilegen, denn ich sah in meinem Material von der amaurotischen Idiotie der SPIELMEIERschen Form auch sehr viele unpigmentierte, aber mit Einlagerungen gefüllte Zellen und konnte auch diese mit der oben angegebenen Art der WEIGERTschen Markscheidenfärbung blau färben.

Was sind das aber nur für Stoffe? Legen wir einen Formolschnitt eine Stunde in auf 50° erwärmten absoluten Alkohol, so finden wir statt der Haufen blau gefärbter Körnchen zum größten Teil leere Säcke, und auch nach längerem Liegen in Äther ist die Menge der pathologischen Einlagerungen deutlich vermindert. Das dürfte beweisen, daß wir es auch hier mit einer Substanz zu tun haben, die zu den lipoiden Stoffen gehört oder ihr nahe steht.

Von besonderem Interesse ist nun, daß die Einlagerungen, die wir in den Gliazellen finden, schon zu einem sehr viel größeren Teil sich mit Scharlach und Osmium schwärzen, und daß dies in noch höherem Grade der Fall ist bei den Einlagerungen der Adventitialzellen. So scheinen hier ganz ähnliche Verhältnisse vorzuliegen, wie wir sie bei den basophilen metachromatischen Stoffen beobachtet haben, die auch allmählich in den Gliazellen und noch mehr in den Adventitialzellen in reine Fettstoffe umgewandelt werden. So dürften wir auch hier wieder einen Beleg für den Transport der Abbaustoffe gegen die Gefäße zu und für ihren weiteren Abbau während dieses Weges sehen.

Dabei muß sich der Gedanke aufdrängen, ob denn nicht vielleicht die für die amaurotischen Idiotien charakteristischen Stoffe nur Vorstufen der lipoiden Körnchen der gewöhnlichen fettigen Degeneration der Ganglienzellen sind, die durch irgend welche pathologische Bedingungen nicht bis zu den gewöhnlichen fettigen Produkten umgewandelt werden. Leider ergeben meine Präparate keine sichere Entscheidung. In manchen Zellen allerdings erscheinen auch

sie im Säurefuchsin-Lichtgrünpräparat fuchsinophil. Aber mein Material war nicht so frisch, daß ich das Ergebnis als einwandfrei betrachten könnte. Auch möchte ich darauf hinweisen, daß wir bei der grobkörnigen fettigen Degeneration der Ganglienzellen auch mit EHRLICHschem Hämatoxylin färbbare und nebenbei fuchsinophile Körnchen als Präprodukte lipoider Stoffe auftreten sehen. Vielleicht wird sich Gelegenheit geben, die Frage, die gewiß nicht ohne Interesse ist, an einem frischeren Material sicher zu entscheiden. Einstweilen können wir nur behaupten, daß die Stoffe der amaurotischen Idiotie von denen der senilen Demenz und der gewöhnlichen fettigen Degeneration der Ganglienzellen verschieden, aber doch wohl mit ihnen verwandt sind, und müssen noch unentschieden lassen, ob es sich um ein Haltmachen auf einer früheren Abbaustufe oder um einen abweichenden Abbauvorgang handelt.

Wie schon SPIELMEIER hervorgehoben hat unterscheiden sich die Einlagerungen in dem STRÄUSSLERschen Falle durch ihre Neigung, sich nach Chromierung mit Osmium zu schwärzen, von den Einlagerungen bei den amaurotischen Idiotien. STRÄUSSLER selbst hält die eingelagerten Körnchen für identisch mit den Einlagerungen der gewöhnlichen Fettdegeneration der Ganglienzellen. Die Bilder der Zellen und die mächtigen Auftreibungen ihrer Fortsätze erinnern sehr an die Zellen der amaurotischen Idiotie, wie schon STRÄUSSLER betont. Wenn es sich also hier wirklich um einfache fettige Einlagerungen handeln würde, so würden sie so hochgradige Deformierungen der Zellen verursachen, wie sie sonst bei dieser Degeneration nicht beobachtet werden. Jedenfalls wäre es nötig, bei einem neuen Falle den färberischen Eigentümlichkeiten dieses Stoffes noch eine besondere Aufmerksamkeit zuzuwenden, um festzustellen, ob die Stoffe mit denen, welche bei der einfachen Fettdegeneration auftreten, übereinstimmen oder doch Beziehungen zu denen der amaurotischen Idiotie zeigen, oder gar von beiden abweichen.

Ganz andere ungewöhnliche Stoffe haben sich bei einem anderen Krankheitsfalle eines Erwachsenen mit paralyseähnlichem Verlauf gefunden, den Herr Dr. BARONCINI im hiesigen Laboratorium eingehend untersucht hat. Er scheint mir der einzige bisher beobachtete Fall einer eigenartigen Erkrankung zu sein. Bei weiterer Nachforschung wird man gewiß noch andere Fälle antreffen. Eine eingehende Beschreibung wird wohl Herr Dr. BARONCINI geben. Ich will hier nur hervorheben, daß es sich im wesentlichen um eine Er-

krankung des Markes handelt. Hier fanden sich neben einer starken und mehr oder weniger diffusen Atrophie des Markes, besonders der Markleisten, und frischem Marchizerfall, sehr zahlreiche große Gliazellen, teils rund, teils in der Form großer plasmatischer, faserbildender Zellen, die vollgepfropft waren mit Stoffen, welche mit Toluidinblau eine prächtige Metachromasie gaben. So hatten sie einesteils durch ihre Beschränkung auf das Mark und die tiefere, noch viele Markfasern enthaltende Rinde, andernteils durch ihre augenfälligste Farbreaktion Beziehungen zu den oben beschriebenen basophil metachromatischen Substanzen, die uns häufiger im Mark begegnen. Aber sie waren wieder in mancherlei Richtungen abweichend. Zunächst zeigten sie sich in einer Anhäufung, und in Form riesiger wolkiger Massen, wie man jene nicht zu sehen pflegt. In älteren Zellen zeigten sie vielfach nach Thioninfärbung eine eigenartig rostfarbene Metachromasie. Außerdem färbten sie sich mit EHRLICHschem Triacid leuchtend grün. Auch hier war wieder der Übergang dieser Stoffe in echte fettige innerhalb der Gliazellen und besonders in den Adventitialzellen und ein Transport gegen die Gefäße zu aufs deutlichste zu verfolgen.

So scheint es mir, daß uns gerade auch diese Krankheiten mit besonderen Abbauprodukten für das Verständnis des ganzen Abbauvorganges und für die Rolle, welche der Glia dabei zukommt, recht wichtige Tatsachen liefern. Gerade hier würde es am nächsten liegen, die in außerordentlicher Massenhaftigkeit auftretenden pathologischen Produkte einer chemischen Untersuchung zu unterwerfen. Das könnte uns wichtigere Aufschlüsse über die Art der Abweichung des Abbauvorganges versprechen als färberische Besonderheiten, und vielleicht einen tieferen Einblick gestatten in das Wesen dieser Erkrankungen. Jedenfalls dürfte es sich auch verlohnen, noch nach weiteren hierher gehörigen Krankheitsformen zu suchen. Mit den Methoden, die man bisher meist zur Untersuchung benutzt hat, treten solche pathologische Produkte nicht genügend deutlich hervor. Man wird also gut tun, auch die hier benutzten in Anwendung zu ziehen und nötigenfalls noch andere auszuprobieren.

9. Die verschiedenen Formen des Abbaues im Nervengewebe.

Nachdem wir uns klar gemacht haben, daß die biologische Bedeutung der amöboiden Gliazellen mit dem Abbau des nervösen

Gewebes in engem Zusammenhang stehen dürfte, wird es zu einem noch besseren Verständnis der Umgrenzung ihrer Aufgabe beitragen, wenn wir uns ihr Verhältnis zu anderen Abbauvorgängen im Nervengewebe klar machen.

Wenn das Nervengewebe mitsamt dem Stützgewebe vollständig zerstört wird, sei es durch irgend ein Trauma, durch eine Blutung oder Erweichung, so sehen wir, daß die Rolle, die tote Nervensubstanz wegzuräumen, in erster Linie den mesodermalen Elementen zufällt. Vom Rande des Herdes her dringen neugebildete Gefäße in das abgestorbene Gewebe; Körnchenzellen, die sich aus den Gefäßwandzellen bilden, wandern in die Umgebung, nehmen Teile des toten Materials in sich auf und wandeln sie in Fett um. Inzwischen wuchert die Glia, welche den Herd umgibt, beteiligt sich aber fast nur dort an der Aufräumungsarbeit, wo, wie es immer der Fall sein wird, auch innerhalb des erhaltenen Nervengewebes Zerfallsprodukte entstehen. Dagegen bildet sie eifrig neues faseriges Gliagewebe, welches den Herd abkapselt. Schließlich werden die fettigen Produkte in den Körnchenzellen allmählich wieder aufgelöst, und damit gehen auch die Körnchenzellen wieder zugrunde. Von dem Herde bleiben nur neugebildete Gefäße, faseriges Bindegewebe und eine oft mächtige neugebildete Gliaoberflächenschicht übrig: mesodermaler Typus (SCHRÖDER).

Anders, wenn eine Schädigung das Gewebe trifft, die allein das Nervengewebe, aber dies in größerer Ausdehnung zum Absterben bringt, das Stützgewebe dagegen unbeschädigt läßt. Jetzt übernimmt die Glia den ersten Teil der Abräumarbeit. Aus den Gliazellen werden Fettkörnchenzellen, indem sie sich um die Markbrocken herumlegen, die aus den zerfallenden Markscheiden entstehen, sie auflösen und in ihrem Zellkörper in Fett umwandeln. Erst später greifen auch hier mesodermale Zellen mit in den Abräumprozeß ein. Denn das Fett, daß wir zunächst in Gliazellen fanden, sehen wir bald auch in Zellen, die innerhalb der Adventitia und in der Pia liegen, später allein in solchen. Es wandert also aus dem ektodermalen Gewebe in das mesodermale über. Daß das so ist kann nicht zweifelhaft sein, wenn wir Bilder aus verschiedenen Stadien gleicher Prozesse vergleichen, in welchen gliogene Körnchenzellen auftreten. Denn mit dem Seltenerwerden der Fettkörnchenzellen in der Nervensubstanz vermehren sich die Fettkörnchen in der Adventitia und im adventitiellen Lymphraum. Wie das aber im

einzelnen vor sich geht, ist noch nicht ganz sichergestellt. Wenn man sieht, wie manchmal Gliakörnchenzellen halb in den perivaskulären Raum hineinragen, während um das Gefäß herum oft mehrere Reihen von Körnchenzellen durch schmale bindegewebige Septa getrennt liegen, möchte man daran denken, daß die Gliakörnchenzellen in den perivaskulären Raum wandern und dort allmählich von der Adventitia umfaßt werden. Aber da man einer Körnchenzelle ihre mesodermale oder ektodermale Abstammung nicht ansehen kann, läßt sich ein solcher Vorgang nicht sicher feststellen, und andere Beobachtungen sprechen mehr dafür, daß das Fett aus den ektodermalen Zellen wieder in veränderter Form in die Lymphe übergeführt und aus dieser von den mesodermalen Zellen aufgenommen wird. Man kann nämlich feststellen, daß sich überall dort, wo gliogene Körnchenzellen im Nervengewebe liegen, zellige Elemente der Adventitia vergrößern, daß der vergrößerte Zelleib allmählich ein wabiges Aussehen erhält und die Zelle sich aus ihrem Verbande löst und zur Gitterzelle bzw. Körnchenzelle umwandelt, und daß auch gelegentlich makrophagische Elemente des Lymphraums den gleichen Entwicklungsgang durchmachen. Jedenfalls sehen wir schon hier ein Zusammenarbeiten ektodermaler und mesodermaler Zellen beim Abbauprozeß des Nervengewebes. Erst wenn die Abräumung im vollen Gange ist, läßt sich bemerken, wie andere Gliaelemente Gliafasern bilden, und wie diese Neubildung von faseriger Glia dann immer weitere Fortschritte macht, bis schließlich das ganze Nervengewebe durch eine faserige Glianarbe ersetzt wird. Diesen ektodermalen Typus des Abbaus (Schröder) sehen wir bei sehr verschiedenerlei Prozessen, am typischsten in den Strangdegenerationen des Rückenmarks, bei der sog. lobären Sklerose und bei der Paralyse nach apoplektiformen Anfällen meist in herdartiger Beschränkung im Mark. Diese zweite Form des Abbaus ist nicht grundsätzlich von der ersten verschieden. So finden wir z. B. bei manchen Formen der Encephalitis Körnchenzellen beider Abstammung nebeneinander.

Gegenüber diesen, durch viele ältere und namentlich neuere Arbeiten (Farrar, Schröder, Merzbacher) genauer bekannt gewordenen Abbauprozessen stellen die im Vorhergehenden eingehend geschilderten, an die amöboiden Zellen gebundenen Degenerationsvorgänge eine dritte abweichende Gruppe dar. Auch hier zerfällt nervöses Gewebe, wenn auch in viel geringerem Grade und in zerstreuterer Weise als bei der vorigen Modifikation. Auch hier verarbeitet die Glia die Zerfallsprodukte nervösen Gewebes, zum Teil auch bis zu lipoiden Stoffen in den Cystchen der amöboiden Zellen. Auch diese fettigen Stoffe scheinen nach ihrer neuerlichen Auflösung schließlich in die Zellen des Gefäßapparates zu gelangen. In anderen amöboiden Zellen aber bilden sich andere Stoffe, die Methylblaugranula, aus dem Plasma der Zellen, die dabei rasch dem Untergang verfallen. Sie scheinen verflüssigt zu werden und mit anderen pathologischen Beimischungen der Gewebsflüssigkeit in perivaskuläre Räume zu gelangen, von wo sie von den mesodermalen Zellen aufgenommen und in fettige Stoffe umgewandelt werden. Wir haben schon oben gesehen, daß es sich dabei, wenigstens zum größten Teile, um andersartige fettige Stoffe handelt als die, welche in den gliogenen Körnchenzellen bei stärkerem Markzerfall auftreten.

Es ist nun eine wichtige Tatsache, daß die Bildung solcher amöboider Gliazellen und ihr Zerfall erhebliche Grade erreichen kann, ohne daß eine Faserbildung der Glia nebenhergeht oder nachfolgt. So sehen wir, wie bei der Dementia praecox, bei der Epilepsie und bei mancherlei anderen Krankheitsprozessen, bei denen wir während des stürmischen Fortschritts der Erkrankung massenhaft amöboide Zellen antreffen, eine Gliafaservermehrung in der Rinde gewöhnlich ausbleibt. Bei anderen Prozessen dagegen scheint von vornherein mit dem Auftreten amöboider Zellen auch eine Bildung pathologischer Gliafasern einzusetzen, so z. B. bei der Paralyse. Da die amöboiden Gliazellen rasch wieder verschwinden, die Gliafaserbildung aber bleibt, so pflegen solche Krankheitszustände ganz verschiedene Bilder zu geben, wenn man sie in einem akuten Zustande oder in einem chronischen zur Untersuchung bekommt. Auch diese dritte Modifikation des Abbauvorganges (Typus der amöboiden Zellen) ist nicht prinzipiell von den vorausgegangenen verschieden und wir finden z. B. bei der progressiven Paralyse, bei den verschiedenen Formen der Meningomyelitis, in der Nachbarschaft von Erweichungsherden und Tumoren amöboide Gliazellen neben gliogenen Körnchenzellen.

Aber nicht überall dort, wo nervöse Substanz zugrunde geht, lassen sich amöboide Zellen nachweisen. Sie scheinen nur bei stürmischerem Zerfall aufzutreten und vielleicht auch da nicht unter allen Umständen. So muß es wohl noch andere Formen eines noch langsameren Abbaues geben. Wir finden nun ganz gewöhnlich bei chronisch degenerativen Prozessen und auch recht häufig neben den

amöboiden Zellen verschiedenartige Formen von Gliazellen, die mehr oder minder reichliche Mengen lipoider Produkte aufgestapelt haben. Diese liegen sehr häufig um den Kern herum, in anderen Fällen wieder in einem Sacke der Gliazellen, der durch Umwandlung einer ihrer Protoplasmafortsätze gebildet worden ist (Tafel XXVIII, Fig. 1 *c*). Man findet auch Zellen, die zwei oder drei solcher Säcke aufweisen, während die übrigen Fortsätze zarte, verzweigte, protoplasmatische Strukturen darstellen oder auch Gliafasern gebildet haben. Zuweilen kommt es auch vor, daß sich solche Gliazellen völlig in kleine Fettkörnchenzellen umwandeln. Hier wird also manchmal sogar nur ein Teil der Gliazelle zur Fettaufspeicherung verwendet, während der übrige Teil anderen Funktionen erhalten bleibt. Regelmäßig findet man dann auch hier wieder die Zellen der Adventitia besonders reichlich mit Fett beladen, und gar nicht so selten kann man die Beobachtung machen, daß auch die Gliazellen in der Nachbarschaft der Gefäße viel reichlichere Fetteinlagerung zeigen, als etwas mehr abseits von denselben.

Mit der geringeren Intensität des Krankheitsvorganges wachsen natürlich auch die Schwierigkeiten, die Zusammenhänge zu verfolgen, also hier z. B. festzustellen, wie die lipoiden Substanzen in die Glia und in die Adventitialzellen gelangen. Es spricht aber vieles dafür, daß bei diesem vierten Abbautypus die vorhandenen Gliaelemente ausreichen, die durch den Zerfall des Nervenmaterials gebildeten Stoffe vorläufig aufzunehmen, um sie dann allmählich in die mesodermalen Elemente abzugeben und daß keine besonderen Formen von Gliazellen gebildet werden.

In allerdings sehr geringem Grade sehen wir diesen vierten Abbautypus wohl in jedem Gehirn des erwachsenen Menschen angedeutet, so daß es wahrscheinlich ist, daß Abbauvorgänge leichteren Grades in die physiologische Breite gehören. Wenn wir das Vorhandensein lipoider Stoffe in den Ganglienzellen, Gliazellen und Adventitialzellen als pathologisch ansehen wollten, dann würde es kein normales menschliches Gehirn geben.

Neben den verschiedenen Typen des Abbauvorganges haben wir dann noch andere Erscheinungen kennen gelernt, die mehr oder minder von diesen unabhängig sind, so die Prozesse, bei welchen einfach basophile Produkte gebildet werden. Wir haben diesen Prozeß wohl anzusehen als eine eigenartige Form der Verflüssigung protoplasmatischer Zellsubstanzen, der sich von anderen ungemein

verbreiteten, aber einem Studium noch schwerer zugänglichen Verflüssigungsprozessen dadurch unterscheidet, daß er ein einfach basophiles Produkt liefert, das wir zum Teil bis in die perivaskulären Räume verfolgen können, wo es offenbar aufgelöst wird, um wahrscheinlich auch wieder in den Zellen der Gefäßwand, in fettige Stoffe umgewandelt, zu erscheinen.

Dann haben wir einen besondersartigen Stoff noch in den metachromatisch basophilen Produkten kennen gelernt. Es ist wohl sicher, daß er mit dem Mark in Zusammenhang steht, wenn er sich auch erst als Einschluß von Gliazellen nachweisen läßt. Wir sahen, wie diese Stoffe sich besonders in den Gliazellen um die Gefäße herum ansammeln, dann in die adventitiellen Lymphscheiden gelangen und dort in fettige Stoffe umgewandelt werden, und es mußte uns nur zweifelhaft bleiben, ob diese Stoffe aus dem Zerfall von Markfasern hervorgehen oder vielleicht auch Störungen in der Ernährung derselben ihre Entstehung verdanken.

Schließlich haben wir dann gesehen, daß bei besonderen Krankheitszuständen in großer Menge Stoffe auftreten, die die lipoiden Stoffe der Ganglienzellen bei dem gewöhnlichen Abbauvorgang vertreten (amaurotische Idiotien), und Stoffe, welche eher in Beziehung zu den basophil metachromatischen zu stehen scheinen. Auch bei ihnen sehen wir, daß sie sich teils schon in Gliazellen, teils in Zellen der Gefäßwand in einfachere Fettstoffe umwandeln. Über die Natur einiger anderen Abbaustoffe haben wir wegen unzureichenden Untersuchungsmaterials noch keine genügenden Aufschlüsse erhalten können.

Nach all diesen Beobachtungen kann es wohl kaum mehr einem Zweifel unterliegen, daß allerlei degenerative Produkte, die sich im Nervengewebe bilden, mit Hilfe sehr verschiedenartiger Formen von Gliazellen dem mesodermalen Gewebe zugeführt werden und schließlich dort als fettige Stoffe erscheinen. Während die Ausgangsstoffe, die in den Ganglienzellen, Markscheiden, Gliazellen auftreten, mancherlei färberische und damit wohl auch chemische Verschiedenheiten erkennen lassen, werden sie einheitlicher, je näher sie den Gefäßen kommen. Damit werden uns jetzt auch mancherlei regressive Veränderungen an den Gliazellen viel besser verständlich. Die Zellen lassen degenerative Merkmale erkennen, wenn sie sich übermäßig mit solchen pathologischen Stoffen beladen haben und ihr kurzer Entwicklungsgang, nachdem ihre Aufgabe erfüllt ist, dem Ende zugeht. Es wird uns nun auch klar, warum so weitgehende Beziehungen

zwischen den Ganglienzellenveränderungen, den Veränderungen an der Glia und an den Gefäßen nachweisbar sind, weil sie in so engen symbiotischen Beziehungen stehen, daß eine Schädigung der einen Umwandlungen der andern zur Folge haben muß. Wir können heute schon, wenn wir mit einer Untersuchungsmethode gewisse Veränderungen an einem der vorbezeichneten Elemente festgestellt haben, mit Sicherheit schließen, daß andere Methoden uns an den anderen Elementen ganz bestimmte Veränderungen zeigen werden. Damit ist uns ein besseres Verständnis mancher feineren pathologischen Vorgänge im Zentralnervensystem gegeben. Denn wir wissen jetzt nicht nur, daß bestimmte Nervenzellenveränderungen mit bestimmten Veränderungen an der Glia einhergehen, sondern auch warum sie das tun. In dieser Arbeit ist nur ein kleiner Teil solcher Beziehungen behandelt worden. Es wird nicht schwierig sein, auch andere Veränderungen hier einzureihen. Das tritt, um nur noch ein Beispiel anzuführen, sehr deutlich hervor bei den Erscheinungen, die man gewöhnlich unter dem Namen der Neurophagie zusammengefaßt hat und die in den letzten Jahren wiederholt eingehender behandelt und zuletzt von Marinesco besonders genau beschrieben worden sind.

Wir sehen zunächst einmal eine auffällige Anhäufung gliöser Elemente um Ganglienzellen herum unter verschiedenen Umständen, bei älteren Leuten, im Gehirn von Phthisikern, bei mancherlei leichteren psychotischen Zuständen, ohne daß sich die Ganglienzellen wesentlich in ihrem Kern und ihrem Plasma verändert zeigen. Mit unserer Darstellung der gliösen Elemente läßt sich auch keine deutliche Vergrößerung des Gliazelleibes und nichts von besonderen Granula in demselben nachweisen. Das läßt wohl den Schluß zu, daß es sich hier jedenfalls nicht um eine Neurophagie handelt, wenn wir uns auch über die Bedeutung dieser periganglionären Gliazellenanhäufung zunächst noch keine bestimmte Vorstellung machen können.

In anderen Fällen wieder sehen wir, wie um die Ganglienzelle herumgelagerte Gliazellen, die aufs schwerste entartete und dem Anscheine nach in ihrer Substanz völlig umgewandelte Ganglienzelle mit zarten hautartigen Ausbreitungen einhüllen. Man kann das besonders auch an verkalkten Ganglienzellen wahrnehmen. Auch hier finden sich wieder keine oder nur sehr wenige Granula im Zellleib der Gliazelle. Darum dürfte es sich auch hier wieder nicht um neurophagische Vorgänge handeln, sondern um eine Art von Totenladenbildung, um eine Abkapselung abgestorbener, für das Nervengewebe zum Fremdkörper gewordener Ganglienzelleiber (Tafel XXXIII, Fig. 15).

Schließlich finden wir dann Gliazellen von amöboidem Charakter mit fuchsinophilen Granula und lipoiden Körnchen um die Ganglienzellen herumliegen, die selbst wesentliche Veränderungen ihrer Form und ihres Plasmas zeigen. Bald haben sich die Gliazellen in die Ganglienzelle eingebohrt, bald legt sich nur ein fingerförmiger pseudopodienartiger Fortsatz des Zelleibes an die Ganglienzelle an. Sie selbst scheint sich aufzulösen. Hier allein dürfte es sich um eine echte Neurophagie handeln, indem die Gliazelle mit zur Verflüssigung der Ganglienzelle beiträgt und sich Stoffe assimiliert, die bei dieser Verflüssigung frei geworden sind. Diese echte Neurophagie selbst kann durch die Abweichungen in der Form der beteiligten Gliazellen und die Verschiedenheiten in den Veränderungen der Ganglienzellen in sehr vielerlei Bildern auftreten. So finden wir also, daß bei genauerem Zusehen, das, was als Neurophagie bezeichnet wird, sich von ganz verschiedener Bedeutung erweist.

Wenn ich nun das im vorausgehenden über die Abbauprozesse Gesagte, noch in eine schematische Übersicht zusammenstelle (p. 530), so kann es sich keineswegs um eine fertige Darstellung der Abbauvorgänge im Nervengewebe handeln, sondern nur um eine vorläufige Skizze, die im einzelnen vielleicht noch Richtigstellungen erfahren wird und in vielem noch der Ausarbeitung bedarf. Weitere Forschungen werden noch mancherlei kaum berührtes oder gar nicht erwähntes hier einfügen können. Sie dürfte aber trotz aller Unfertigkeit und Unvollständigkeit doch von Vorteil sein, um sich leichter zurecht zu finden, Lücken, die der Ausfüllung bedürfen, besser zu ersehen und die Nachprüfung zu erleichtern.

10. Ergebnisse dieser Untersuchungen und Grenzen ihrer Leistungsfähigkeit.

In der vorausgegangenen Darlegung sind mancherlei Veränderungen, welche sich in akuten Erkrankungszuständen des zentralen Nervensystems finden ließen, beschrieben und ihre Erklärung versucht worden. Die Beobachtungen sind an so vielerlei Material gesammelt und bei so zahlreichen Nachuntersuchungen mit verschiedenen Methoden bestätigt worden, daß sie als feststehend betrachtet werden

Skizze der Abbauerscheinungen am Nervengewebe.

Formen des Abbaues	Vorkommen	Präprodukte	Färberische Eigentümlichkeiten des besonderen pathologischen Stoffes in den nervösen Elementen	Färberische Eigentümlichkeiten des besonderen pathologischen Stoffes: in den Gliazellen	in den perivaskulären Räumen	in den mesodermalen Zellen der Gefäßwand, Blutelemente, Pia	Bemerkungen
**I. Abbauerscheinungen an den Ganglienzellen.** 1. Granuläre Degeneration des Zellplasmas.							
a) Fettige Entartung der Ganglienzellen	Sehr verbreitet. Typus: senile Demenz	Fuchsinophile Granula	+ gelbliche Pigmentierung. Bräunung bis Schwärzung nach Chromosmium. Scharlach. Karbolfuchsinmethylenblau. WEIGERTsche Markscheidenfärbungen. WEIGERTsche Fibrinfärbung. — MAY-GRÜNWALD, EHRLICHsches Hämatoxylin	Ebenso. Daneben in frischeren Erkrankungszuständen auch fuchsinophile Granula. Bei rasch fortschreitender Erkrankung auch wie 1d	—	Wie in den Gliazellen, bei frischeren Krankheitszuständen daneben fuchsinophile Körperchen	—
a) Grobkörnig-fettige Entartung	Senile Demenz, Arteriosklerose, Herderkrankungen, Encephalitis	Mit MANNscher Färbung dunkelblau, mit BIELSCHOWSKY braunschwarz. + Lithionkarmin, EHRLICHS Hämatoxylin. — Scharlach, Osmium nach Chromierung	Wie 1a. Fettkörner sehr groß, zeigen im Chromosmiumpräparat die Form lipoider Cystchen	Wie 1a	—	Wie 1a	—
b) Granula der amaurotischen Idiotien	Amaurotische Idiotien	Fuchsinophile Granula?	+ MAY-GRÜNWALD, WEIGERTsche Markscheidenfärbung, EHRLICHsches Hämatoxylin. — Osmium nach Chromierung, Scharlach. Bei der SPIELMEYERschen Form leichte Pigmentierung, bei der TAY-SACHSschen fehlt diese fast ganz	Zum kleineren Teile wie in den Ganglienzellen, zum größeren Teile + Scharlach, Osmium nach Chromierung, Karbolfuchsinmethylenblau	—	+ Scharlach, Osmium nach Chromierung, Karbolfuchsinmethylenblau	—

Formen des Abbaues	Vorkommen	Präprodukte	Färberische Eigentümlichkeiten des besonderen pathologischen Stoffes in den nervösen Elementen	Färberische Eigentümlichkeiten des besonderen pathologischen Stoffes			Bemerkungen
				in den Gliazellen	in den perivaskulären Räumen	in den mesodermalen Zellen der Gefäßwand, Blutelemente, Pia	
c) Fuchsinophile Granula (degenerativer Art)	Verbreitet bei akuten Prozessen und hier z. T. mit 1d zusammenfallend und bei chronisch degenerativen Prozessen (vielleicht handelt es dabei um verschiedene Dinge)	—	+ S-Fuchsinlichtgrün, Hämatoxylinfärbung Methode VII, — Scharlach, Osmium nach Chromierung, May-Grünwald	Bei den chronisch degenerativen Prozessen oft ohne Granula<br>Bei den akuten wie 1d	Bei den akuten wie 1d	Bei den akuten wie 1d	—
d) Granula der schweren Zellerkrankung Nissls	Verbreitet bei akuten Prozessen	—	Offenbar raschem Wechsel in dem Verhalten zu verschiedenen Farbstoffen unterworfen<br>+ basische Anilinfarben, S-Fuchsinlichtgrün, Fibrinfärbung (aber nur vorübergehend).<br>— May-Grünwald, Scharlach, Osmium nach Chromierung	Amöboide Gliazellen mit fuchsinophilen Granula und Fettcystchen. Methylblaugranula, fibrinoide Granula, Füllkörperchen	Mit S-Fuchsinlichtgrün, mit Malloryschem Hämatoxylin, Mannscher Lösung (Methylblau), der Fibrinmethode färbbare Produkte. Degenerierte amöboide Gliazellen	Mit Scharlach, Osmium nach Chromierung färbbare Stoffe	Stellt wohl schon einen eigenartigen Verflüssigungsprozeß des Zellplasmas dar
2. Verflüssigungsprozesse des Zellplasmas.							
a) Einfache basophile Stoffe	Verbreitet bei akuten Prozessen	—	+ Basische Anilinfarben ohne ausgesprochene Metachromasie	Wie 1d und auch andere Gliazellformen mit einfach basophilen Einschlüssen	Wie 1d und einfach basophile Produkte	Wie 1d	—
b) Zahlreiche Verflüssigungsprozesse, bei welchen besondere, bis jetzt darstellbare Produkte nicht gebildet werden	Sehr verbreitet bei akuten und chronischen Prozessen	—	—	Wie bei 1a und 1d	Wie bei 1a u. 1d	Wie bei 1a und 1d	Man vergleiche als Beispiel das über den Schwund der Protoplasmafortsätze bei der fettigen Degeneration Gesagte

Formen des Abbaues	Vorkommen	Präprodukte	Färberische Eigentümlichkeiten des besonderen pathologischen Stoffes in den nervösen Elementen	Färberische Eigentümlichkeiten des besonderen pathologischen Stoffes			Bemerkungen
				in den Gliazellen	in den perivaskulären Räumen	in den mesodermalen Zellen, der Gefäßwand, Blutelemente, Pia	
**II. Abbauerscheinungen an den Markfasern u. Achsenzylindern.**							
a) Schollenzerfall der Markscheiden, ELZHOLZsche Körperchen, Myelinschollen und -kugeln	Sehr verbreitet. Typus: Strangdegeneration des Rückenmarks	—	+ Bräunung bis Schwärzung mit Osmium nach Chromierung. — Scharlach, Karbolfuchsinmethylenblau, MAY-GRÜNWALD, basische Anilinfarben	+ Scharlach. Osmium nach Chromierung — Gelbliche Pigmentierung. Karbolfuchsinmethylenblau	—	Wie in den Gliazellen	—
b) Basophil metachromatische Produkte (π-Granula REICHS)	Sehr verbreitet. Dem Anschein nach ohne Zerfall von Markscheiden vorkommend	—	Erst in den Gliazellen nachweisbar	Zum größeren Teil mit Toluidinblau, Thionin, Kresylviolett metachromatisch rötlich färbbar, zum kleineren Teil rasch in mit denselben Farben metachromatisch grünlich und mit Scharlach färbbare Fettstoffe übergehend	—	Zum größten Teil mit den erwähnten Farben grünliche Farbtöne annehmend, mit Scharlach und Osmium nach Chromierung färbbar	—
b) Besondere basophil metachromatische Produkte	Bei einem besonderen Krankheitsprozeß	—	Erst in den Gliazellen nachweisbar	Mit Toluidinblau und Thionin rötlich metachromatisch färbbar, in außerordentlicher Menge auftretend, in älteren Zellen rostfarbene Metachromasie. Mit EHRLICHschem Triazid grüne Färbung	—	Nur noch zum geringsten Teil rötlich metachromatisch basophil, zum größten Teil grünlich, mit Scharlach und Osmium nach Chromierung färbbar	—
c) Granulärer Zerfall der Achsenzylinder	Sehr verbreitet, besonders bei akuten Prozessen	—	Färben sich mit der MANNschen Färbung rot, mit S-Fuchsin leuchtend rot; mit der DONAGGIOschen Hämatoxylinfärbung darstellbar	Amöboide Gliazellen wie 1 d	Wie 1 d	Wie 1 d	—

dürfen. Nicht mit der gleichen Bestimmtheit läßt sich behaupten, daß auch die Schlüsse, die aus ihnen gezogen worden sind, überall und in allen Einzelheiten das Richtige treffen. Denn so ausgedehnt auch das Untersuchungsmaterial ist, aus dem sie abgeleitet worden sind, es sind immer noch empfindliche Lücken gewesen, die erst durch weitere Untersuchungen hätten ausgefüllt werden können, und über die einstweilen die Erklärung eine Brücke schlagen mußte. Auch sind einzelne Beobachtungen übrig geblieben, die sich nicht zwanglos in die den Befunden gegebene Erklärung einfügen ließen. So wird vielleicht eine noch genauere Verfolgung der Vorgänge nach einzelnen Richtungen eine Änderung ihrer Beurteilung nötig machen. Aber auch wenn eine bessere Erkenntnis mancherlei Einzelheiten an der Darstellung zu berücksichtigen haben wird — daß uns diese Untersuchungen wesentliche Fortschritte in dem Verständnis pathologischer Vorgänge innerhalb des Zentralnervensystems bringen, ist ganz gewiß.

Zunächst zeigen sie uns ungemein deutlich tiefgreifende Gewebsveränderungen in einer großen Anzahl von Krankheiten, bei welcher wir bisher keine oder nur sehr unbestimmte und schwer zu beurteilende pathologische Befunde erheben konnten. Am besten läßt sich dies an einem Beispiel zeigen. Wenn wir das Bild 20 auf Tafel XXIX betrachten, so fällt die Schwere der Gewebsveränderungen gegenüber dem normalen Rückenmark sofort in die Augen. Ehe ich solche Bilder dargestellt habe, habe ich Schnitte des gleichen Rückenmarks vor mir gehabt, die mit der Weigertschen Markscheiden- und Gliamethode und nach Nissl gefärbt waren. Sah man die Markscheidenpräparate etwas genauer an, so bemerkte man, daß die einzelnen Markfasern in der weißen Substanz etwas weiter als im normalen Rückenmark voneinander abgerückt waren. Was das für einen Grund hatte, war nicht zu verstehen. Im Gliafaserpräparat war die geringe Menge der Fasern in der Randschicht und in der ganzen weißen und grauen Substanz auffällig. Man mußte daran denken, daß es sich um eine mangelhafte Färbung handelte, jedenfalls konnte man auch mit diesem Befund nicht viel anfangen. Im Nissl-Präparat zeigten sich die Ganglienzellen stark verfettet, z. T. klein, birnenförmig. Die Gliakerne waren auffallend klein, dunkel, oft mit kleinen Protoplasmaleibern umgeben und von Häufchen grünlichen Pigments umlagert. Aber was hatte das für eine pathologische Bedeutung? Auf Grund dieser Präparate ließen sich die Veränderungen im Rückenmark nur unterbringen unter jene pathologischen Befunde, die sich im kranken Nervengewebe so oft erheben lassen, unsere Erkenntnis aber so wenig fördern, weil sie zu unbestimmt und uncharakteristisch sind und deshalb auch schwer gegenüber anderen Veränderungen abgegrenzt werden können. Jetzt sehen wir über den ganzen Rückenmarksquerschnitt ausgebreitete, scharf gekennzeichnete Abweichungen. Wir können erkennen, warum die Markfasern der weißen Substanz voneinander abgerückt erscheinen: weil sich große amöboide Gliazellen dazwischen geschoben haben. Wir erkennen auch, warum die Gliafasern so spärlich sind: weil sich amöboide Gliazellen und Füllkörperchen gebildet haben, mit deren Entstehung die Gliafasern in mehr oder minder großer Anzahl der Auflösung verfallen. Wir finden die Ganglienzellen nicht nur fettig entartet, sondern auch vielfach ihrer Protoplasmafortsätze beraubt und an ihren Endapparaten geschädigt. An Stelle der feinen nervösen Strukturen der grauen Substanz ist für die nervöse Leistung wertloses Gewebe getreten: amöboide Zellen und Füllkörperchen. Das ganze Rückenmark ist in seiner nervösen Masse enorm atrophiert, an nutzlosem Ersatz- und Füllmaterial enorm hypertrophiert. Mit dieser Erkenntnis fällt aber auch ein besseres Licht auf den ganzen Krankheitsvorgang.

Ungemein schwere Symptome und Krankheitsbilder, zu deren anatomischer Begründung bisher nur dürftige Befunde beizubringen waren, erscheinen uns nunmehr in einem weiter gehenden Grade anatomisch verständlich: der Status epilepticus, schwere paralytische Anfälle, schwere Infektions- und Intoxikationsdelirien, katatonische Erregungszustände. Wer noch nicht über Zweifel hinwegkommen konnte, ob es sich denn wirklich bei der Dementia praecox, der Epilepsie um Krankheiten handelt, die mit schweren organischen Veränderungen in der Hirnrinde einhergehen, wird jetzt leicht seine Zweifel beseitigen können. Bei anderen akuten Erkrankungen, bei welchen wir bisher schon pathologische Veränderungen kannten, zeigen sie uns dieselben noch deutlicher. Dies tritt uns auch besonders vor Augen, wenn wir experimentelle Reihen subakuter maximaler Vergiftungen, die mit neuen Untersuchungsmethoden bearbeitet sind, den schon früher mit der Nisslmethode gewonnenen Ergebnissen gegenüberstellen.

Mit der Handhabe, die pathologischen Veränderungen schärfer und deutlicher darzustellen, ergibt sich dann natürlich auch die Mög-

lichkeit, ihre Verbreitung im Zentralnervensystem besser zu studieren. Dabei sind manche Ergebnisse sehr überraschend.

Allerdings stoßen wir auch bald auf Grenzen der Leistungsfähigkeit. Die amöboiden Zellen sind kurzlebige Gebilde, und sie selbst und die meisten der pathologischen Stoffe, welche sich im Nervengewebe bilden, verschwinden in verhältnismäßig kurzer Zeit wieder aus demselben. Wenn z. B. ein Sturm katatonischer Veränderungen die Hirnrinde durchfegt hat, ist bald nichts mehr von eigenartigen Gliazellen und pathologischen Granula zu sehen, abgesehen von Anhäufungen lipoider Stoffe in Ganglien- und Gliazellen und in den Zellen der Gefäßwand, die viel langsamer wieder beseitigt werden. Die Spuren, die er sonst an Nervenzellen und Glia zurückgelassen hat, sind auch nach Anwendung der neuen Untersuchungsmethoden wieder viel unbestimmter und schwerer darzustellen und zu deuten. So erhalten wir von derselben Krankkeit recht verschiedene Bilder, wenn wir akute Zustände und chronische untersuchen, und bekommen dagegen Befunde von weitgehender Übereinstimmung bei ganz verschiedenen Krankheiten, wenn es sich nur um gleich schwere Krankheitsvorgänge handelt. In einem Schnitte eines schweren Intoxikationsdelirs von Delirium acutumartigem Charakter sehen wir sehr ähnliche Veränderungen wie in einer an einer schweren Erregung gestorbenen Katatonie. Daraus ergibt sich, daß sich einer differentialdiagnostischen Verwertung der Befunde mancherlei Schwierigkeiten entgegenstellen. Wollte man daraus schließen, daß der Krankheitsprozeß in den beiden erwähnten Fällen der gleiche sei, so wäre das sicher unrichtig. Wir stellen eben nur die Abbauerscheinungen dar. Auch am Rückenmark ergeben sich, wenn wir nur die Körnchenzellen färben, ganz verschiedenartige Befunde, wenn es sich um eine rasch fortschreitende oder chronische oder stationär gewordene Tabes handelt, und an den Körnchenzellen im Hinterstrang allein können wir noch nicht unterscheiden, ob es sich um eine Tabes oder um eine aufsteigende Hinterstrangserkrankung nach Querschnittsläsion des Rückenmarks handelt. Der Unterschied zwischen diesen beiden Erkrankungsprozessen beruht in der verschiedenen Ausbreitung, in der Beteiligung verschiedener Systeme des Rückenmarks, in dem, was schließlich zerstört wird und was unversehrt bleibt. Die Ausbreitung akuter Veränderungen in der Hirnrinde können wir jetzt bei geeigneten Fällen besser studieren als vordem, weil sie uns deutlicher dargestellt werden. Die endlichen

Ausfälle aber sind in der Hirnrinde heute noch viel schwerer festzustellen als im Rückenmark, z. T. wohl überhaupt noch nicht nachweisbar. So kommt es auch, daß wir bei manchen Krankheitsprozessen ganz auffällige Veränderungen während eines akuten Zustandes sehen, daß uns aber die im chronisch gewordenen Zustande übrig gebliebenen Veränderungen viel unbedeutender erscheinen, und daß wir ihnen oft ganz ratlos gegenüberstehen. So ist die Hilfe, welche diese Untersuchungen unseren differentialdiagnostischen Bestrebungen leisten können, jedenfalls noch eine beschränkte.

Zunächst dürfte uns aber schon das Vorkommen zahlreicher amöboider Zellen in einzelnen Fällen einen wichtigen Anhaltspunkt für die Diagnose geben. Wir finden z. B., daß bei einzelnen, in schwerster Erregung verstorbenen Kranken die Bildung jeder Form von amöboiden Zellen ausgeblieben ist, während sie in anderen in reichlichster Menge nachzuweisen sind. Man muß wohl annehmen, daß es sich dabei um verschiedenartige Krankheitsprozesse handelt. Schon heute scheint es mir wahrscheinlich, daß solche Fälle, in welchen sich reichlich amöboide Zellen finden, ohne daß gleichzeitig eine sehr schwere körperliche Erkrankung bestanden hat, die erfahrungsgemäß auch die Bildung amöboider Gliazellen im Nervensystem anregen kann, nicht zur Manie gehören, und daß das Fehlen aller amöboider Gliazellen in einer mit schwerer Erregung einhergegangenen Psychose gegen eine Dementia praecox sprechen dürfte. In den Stadtasylen beobachtet man ja recht häufig solche akute schwere Erregungszustände, bei welchen eine klinische Differentialdiagnose bis zum Tode kaum möglich war. Daß das Vorhandensein reichlicher amöboider Gliazellen in solchen Fällen eine Dementia praecox, ihr Fehlen ein manisch-depressives Irresein beweise, will ich schon um deswillen nicht behaupten, weil wohl manche Fälle, bei welchen wir uns heute um die Differentialdiagnose zwischen den beiden Krankheiten bemühen, mit dem weiteren Fortschreiten psychiatrischer Erkenntnisse weder bei der einen noch bei der anderen ihren Platz finden dürften.

Wenn es dann weiter auch den Anschein hat, daß die Form der amöboiden Gliazellen mehr von der Schwere des Krankheitszustandes, als von der Art der Krankheit abhängt, so werden sich doch vielleicht daraus bei weiterer Nachforschung und besserer Sichtung der Befunde auch differentialdiagnostische Anhaltspunkte gewinnen lassen. So habe ich beim Status epilepticus niemals dieselben runden amöboiden Zellen in der Rinde angetroffen, wie sie z. B. beim Status

paralyticus oder bei schweren Infektionsdelirien gewöhnlich sind. Bei diesen Formen selbst bedürfen wir ja kaum einer histologischen Untersuchung zur Sicherung der Diagnose, wohl aber deutet diese Beobachtung auf eine Möglichkeit differentialdiagnostischer Verwertung bei anderen Fällen.

Manche Krankheitsformen scheinen dann auch durch Abbauprodukte besonderer Art gekennzeichnet. Ihre Zahl ist zwar bisher noch recht klein, doch sind wir auch noch ganz in den Anfängen der Arbeit.

Interessant ist jedenfalls auch noch ein anderes Ergebnis. Wir sehen aus dem Verhalten der Glia, daß viele degenerative Krankheitsprozesse einen starken Wechsel in der Stärke des Fortschreitens unterworfen sind, daß sie bald nur langsam fortschreiten, bald stark anschwellen, bald stationär werden. Bei der Untersuchung eines größeren anatomischen Materials habe ich mir wenigstens hierüber andere Ansichten bilden müssen, als ich sie vorher, durch die klinische Beobachtung allein gewonnen hatte.

Von größerer Bedeutung dürfte dann wieder der Fortschritt in unseren allgemeinen pathologischen Erkenntnissen sein. Aufs deutlichste wird jetzt klar, daß die Neuroglia noch andere Zwecke zu erfüllen hat als die einer Stützsubstanz des zentralen Nervengewebes. Ein ganzer Formenkreis pathologischer Gliaelemente ist dieser Aufgabe entzogen. Er räumt auf, beseitigt die Abfallstoffe und hilft sie in die Lymphbahnen abführen. Unter gewissen Umständen erlangt die amöboide Glia, welche diese Aufgabe zu erfüllen hat, gleichsam das Übergewicht über die faserbildende Stützsubstanz, die selbst zu mehr oder minder großem Teile zerfällt. Eine ganz ungemein reiche Mannigfaltigkeit gliöser Zellformen lernen wir kennen und ihre Bedeutung einigermaßen verstehen. So können wir tiefer in das pathologische Geschehen in der Hirnrinde und das Wesen der Krankheitsvorgänge hineinsehen.

Wir haben dann auch einzelne, freilich noch einer Nachprüfung bedürftige Beobachtungen machen können, welche anzudeuten scheinen, daß die Glia nicht nur beim Zerfall untergehenden Nervengewebes, sondern auch bei Störungen in den Ernährungsvorgängen beteiligt ist. Damit würden sich noch weitere Aussichten eröffnen, durch das Studium ihrer Veränderungen in ein besseres Verständnis mancher pathologischen Prozesse einzudringen.

Mit dem Nachweis sehr verschiedenartiger Stoffe im pathologischen Nervengewebe muß aber allmählich auch der Versuch einer Zusammenarbeit mit einer Chemie des pathologischen Gehirns, wie sie von Wlassak und Reich schon mit der des normalen versucht worden ist, Aussicht auf Erfolg versprechen. Denn da solche pathologische Stoffe bei einigen Krankheitszuständen in ganz außerordentlicher Menge im Gehirn anzutreffen sind, dürfte es keine unübersteiglichen Schwierigkeiten geben, sie zu extrahieren, zu isolieren und ihre Zusammensetzung genauer zu erforschen. Dadurch würden wir dann dem Verständnis der Krankheiten noch um ein weiteres Stück näher kommen.

11. Reichardts Hirnwägungen und kritische Bemerkungen über die pathologische Histologie des Zentralnervensystems.

In mehreren Arbeiten der letzten Jahre, die im übrigen das Hirngewicht behandeln, hat sich Reichardt auch mit der Unzulänglichkeit der gegenwärtigen histopathologischen Untersuchungsmethoden des Gehirns und damit im Zusammenhange mit meinen Arbeiten beschäftigt. Es liegt mir nun daran, gerade hier auf seine Ausstellungen einzugehen, weil seine Untersuchungen über das Hirngewicht und die Hirnmaterie mancherlei Berührungspunkte haben mit den akuten Veränderungen des Nervengewebes, die im vorstehenden eingehend behandelt worden sind und weil sich dabei Gelegenheit gibt, die Brauchbarkeit der pathologischen Histologie auf einigen spezielleren Gebieten noch etwas eingehender zu erörtern.

Als Beweis für die gedrückte Stimmung, die nach der Begeisterung im letzten Jahrzehnt des vorigen Jahrhunderts sich gegenwärtig der Pathologen des Zentralnervensystems bemächtigt hätte, führt Reichardt einen einleitenden Satz meines Autoreferates „Über den Abbau des Nervengewebes" an, in dessen weiterer Ausführung über Untersuchungen berichtet wird, die eben jene Mängel teilweise beheben und weitere Fortschritte ermöglichen sollen. Es scheint mir nun, daß gerade auf den dort vorgezeichneten Wegen die pathologische Anatomie dasselbe festzustellen vermag, was Reichardt in seiner Abhandlung über die Hirnmaterie als einen Erkenntnisfortschritt darstellt, den allein die physikalische Hirnuntersuchung bringen konnte, und zwar nach mancher Richtung hin noch viel mehr ins einzelne gehend und damit auch viel exakter, als das je mit der Wage ohne Chemie möglich sein dürfte.

Schon in dem oben erwähnten Vortrage habe ich kurz darauf hingewiesen, daß wir bei gewissen akuten Krankheitszuständen des Zentralnervensystems eine solche Menge eigenartig gewucherter Gliazellen und besonderer pathologischer Produkte im Gewebe antreffen, daß bei dem noch jedenfalls geringen Ausfall und der oft gleichzeitig nachweisbaren Quellung vieler nervöser Strukturen eine Anschwellung des Gehirns zustande kommen dürfte, wie das von den Franzosen schon für die akuten Stadien der Paralyse früher behauptet worden ist. Was ich dort nur flüchtig andeuten konnte, im einzelnen damals auch noch weiterer Nachprüfung bedürftig schien, ist nun in der vorliegenden Arbeit weiter ausgeführt.

Die Ergebnisse der Reichardtschen exakten Hirnwägungen werden also durch die anatomische Untersuchung bestätigt und geben ihrerseits wieder eine erfreuliche Ergänzung und Bestätigung anatomischer Untersuchungen durch eine ganz andere Forschungsmethode. Während aber Reichardt eine Zahl erhält, die uns wohl einen Ausdruck einer bedeutsamen Veränderung des Gehirns, in ganz frischen Fällen, wohl auch des Grades derselben gibt, zu deren Erklärung jedoch die physikalische Hirnuntersuchung bis jetzt kaum etwas anderes heranziehen kann als Hypothesen, vor welchen Reichardt in der pathologischen Histologie so sehr warnt, sehen wir im Mikroskop, daß die Gewichtsvermehrung sich im einzelnen Falle aus sehr verschiedenen Bestandteilen zusammensetzt, veränderten alten und hinzugekommenen neuen Elementen, aus geformten und ungeformten Bestandteilen, aus Stoffen von ganz abweichendem morphologischen und färberischen Verhalten und deshalb auch von verschiedener chemischer Beschaffenheit. Wir können auch manche Beziehungen dieser pathologischen Bildungen zu den normalen Strukturen erkennen. Ja, wir vermögen in einzelnen Fällen festzustellen, daß neben einer solchen Produktion reichlicherer und größerer pathologischer Elemente und einer Ablagerung von Stoffen, welche das Hirngewicht vermehren müssen, nervöse Strukturen in geringerer und größerer Zahl zugrunde gegangen sind, daß sich also das schließliche Hirngewicht aus einer Verminderung von normalen und einer Vermehrung von pathologischen Gewebsbestandteilen zusammensetzen muß. Damit eröffnet sich aber eine tiefere Einsicht in die krankhaften Veränderungen des Nervengewebes. Wenn ich also auch zugebe, daß es von großem Interesse sein mag, auch einen zahlenmäßigen Ausdruck für die Veränderungen des Gehirns zu haben,

so meine ich doch, daß der Reichardtschen Methode auf diesem Gebiete keine größere Leistungsfähigkeit zukommt als der pathologischen Histologie des Gehirns.

Was hier im allgemeinen dargelegt ist, soll noch durch einige besondere Beispiele näher erläutert werden.

Bei den Überlegungen, welche den Anlaß zu diesen Untersuchungen gegeben haben, spielte einer jener seltenen und immer überraschenden Fälle eine Rolle, von denen auch Reichardt spricht, der eines stuporösen Katatonikers, welcher ohne irgend welche Anzeichen einer Erkrankung körperlicher Organe plötzlich tot zu Boden gefallen war. Nachdem die Leichenuntersuchung keinerlei Ursache für das rasche Ende ergeben hatte, lag es nahe. Veränderungen des Gehirns dafür verantwortlich zu machen. Bei der äußerlichen Betrachtung war an ihm nichts auffallendes zu bemerken gewesen. Jedenfalls war es ohne Hyperämie, ohne das geringste Ödem, im Gegenteil war die Hirnsubstanz abnorm trocken, ohne Hydrocephalus, ohne jede Exsudation. Aber auch die Untersuchung mit den damals bekannten Methoden ergab keine Befunde, die den plötzlichen Tod erklärten. Jedenfalls war nichts von entzündlichen Vorgängen, nichts von Gliose und nichts von jenen Veränderungen nachzuweisen, die dem pathologischen Anatomen so geläufig sind, wenn es sich um die Vergrößerung von Organen handelt. Manche scheinbare kleine Abweichungen vom Normalen blieben in ihrer Bedeutung unverständlich.

Der Mangel entsprechender Befunde ließ sich aber auch auf einem anderen Wege erklären als dadurch, daß sich etwa das Zellplasma dieses Hirns in ein ganz andersartiges, sagen wir mit Reichardt etwa pflanzenähnliches, jedenfalls mikroskopisch nicht unterscheidbares umgewandelt hätte. Es war viel näherliegend, anzunehmen, daß die angewandten Untersuchungsmethoden nicht ausreichten, tatsächlich vorhandene pathologische Veränderungen zu erkennen. Wer sich einmal klar gemacht hat, wie viele Strukturen des so ungeheuer verwickelten Baues des Gehirns wir heute nur mühsam, unsicher, gelegentlich einmal unter ganz besonderen Bedingungen darzustellen vermögen, wird ohne weiteres zugeben müssen, daß es auch einen Hirntod geben kann, dessen anatomische Ursachen sich jetzt noch nicht feststellen lassen und wird sich bemühen müssen, seine Untersuchungsmethoden zu verbessern. So habe ich das interessante Gehirn immer von neuem vorgenommen und an ihm die Methoden

versucht, die zunächst bei gröberen Hirnveränderungen ausprobiert worden waren. Jetzt zeigt sich, daß in der Hirnrinde sehr tiefgreifende Störungen nachzuweisen sind, morphologischer und qualitativer Art. Verschiedene Abbildungen dieser Arbeit stammen von diesem Falle. Die pathologische Histologie hat ihm also etwas von dem Rätselhaften genommen.

Nun wendet sich Reichardt gegen die Deutung dieser Befunde als Abbauerscheinungen. Die Ergebnisse seiner Gehirnwägungen sprechen gegen eine solche Erklärung. Er will aber zufrieden sein, wenn sie im Sinne einer anderen Mischung des Protoplasmas aufgefaßt werden. Tatsächlich sind in diesem Gehirn keine degenerierenden Achsenzylinder festgestellt worden. Auch ein Ausfall von Zellen hat sich nicht nachweisen lassen. Trotzdem bin ich der Meinung, daß die hier beobachteten Veränderungen mit unter die Abbauerscheinungen im allgemeinen gerechnet werden müssen. Der Zerfall des Nervengewebes, besonders der akute, geht, wie die vorliegenden Untersuchungen gezeigt haben, nicht etwa in der Weise vor sich, daß Teil um Teil des nervösen Gewebes spurlos verschwindet. Ganz zweifellos spielen, namentlich bei den akuten Prozessen, Verflüssigungsvorgänge eine erhebliche Rolle. Diesen geht sehr häufig eine mehr oder minder starke Anschwellung der protoplasmatischen Strukturen oft mit stofflichen Veränderungen des Plasmas voraus. Die Glia zeigt stürmische reaktive Veränderungen, die zu einer starken Anschwellung der gliösen Elemente und zu einem Zerfall vieler derselben führt. Durch die Auflösung nervöser Gewebsbestandteile und degenerierter Gliazellen in der Flüssigkeit, welche das Nervengewebe durchtränkt, muß dieselbe verändert werden. Im mikroskopischen Bilde kann auch dies dargestellt werden, wenn durch die Fixierungsmittel pathologische Bestandteile der Gewebsflüssigkeit ausgefällt, in unlösbare Produkte übergeführt und durch Färbungen anschaulich gemacht werden. Solche Produkte haben wir in größerer Anzahl kennen gelernt. Der Abbau des Nervengewebes geht also tatsächlich einher mit mannigfachen Veränderungen in der Mischung der verschiedenen protoplasmatischen Bildungen und der Gewebsflüssigkeit und der größte Teil der vorausgegangenen Darlegungen beschäftigt sich, um schließlich wieder einen Reichardtschen Ausdruck zu gebrauchen, mit Art- und Mischungsveränderungen des Protoplasmas oder, besser gesagt, der Gewebsstrukturen.

Ich glaube nun, daß es auch solche Gewebsveränderungen gibt, bei denen es sich um in der Reichardtschen Anschauungsweise zu sprechen, im wesentlichen nur um qualitative Veränderungen des Plasmas ohne Abbauerscheinungen handelt. Ich sehe solche besonders in den Gewebsbildern, die man bei manchen leichteren Psychosen nach Infektionskrankheiten (nicht bei den schweren infektiösen Delirien) beobachtet, wo uns eine Schwellung der nervösen Elemente in der Form der akuten Zellveränderung Nissls oder der trüben Schwellung anderer Autoren, aber kein Anzeichen eines Zerfalls nervöser Strukturen begegnet. Man braucht nur Zellen aus einer bestimmten Stelle der Hirnrinde von solchen Infektionspsychosen zu photographieren und mit gleich vergrößerten entsprechenden Stellen aus der normalen Hirnrinde zu vergleichen, um sich zu überzeugen, in welch bedeutendem Grade die akute Zellveränderung das Volum der Ganglienzellen vergrößern kann. Mit dieser Anschwellung der Ganglienzellen sind auch stoffliche Veränderungen in derselben vor sich gegangen. Die auffälligste derselben macht sich in einem Schwund der Nissl-Schollen bemerkbar. Hier finden wir nun aber daneben keine amöboiden Gliazellen und keine von den Stoffen, die wir oben kennen gelernt und mit dem Zerfall des Nervengewebes in Zusammenhang gebracht haben. In dem erwähnten Fall von Dementia praecox dagegen waren amöboide Gliazellen reichlich vorhanden, besonders Zellen mit fibrinoiden Granula fanden sich in außerordentlicher Menge, und ich glaube, daß aus Gründen, die oben ausführlich dargelegt worden sind, diese Bildungen mit dem Zerfall von Nervengewebe im Zusammenhang gebracht werden müssen und als Abbauerscheinungen aufzufassen sind, wenn wir auch im vorliegenden Falle nur die ersten Anfänge davon vor Augen haben, weil der Tod ihre weitere Entwicklung abgeschnitten hatte.

Gerade aber darin zeigt sich meines Erachtens auch wieder eine Überlegenheit der histologischen Untersuchungen über die Hirnwägungen, daß wir mit ihrer Hilfe verschiedenartige Prozesse auseinanderhalten können, die die Wage niemals zu trennen vermögen wird.

Was nun für die Dementia praecox gilt, das gilt auch von den anfallsartigen Störungen der Paralyse und der Epilepsie. Es ist zunächst nur unter Einschränkungen richtig, wenn Reichardt behauptet, daß man mittels des Mikroskops nicht feststellen könne, ob ein Kranker, der paralytische Veränderungen in seinem Hirn zeigt,

nun auch wirklich geisteskrank war, ob er im Blödsinn starb oder in einer sehr vollständigen psychischen Remission, ob er einem Anfall bzw. seiner Hirnkrankheit oder einer zufälligen hinzugetretenen anderen Erkrankung erlegen ist.

Wir können sehr wohl aus den Veränderungen der Hirnrinde bei der progressiven Paralyse einiges über den klinischen Zustand zur Zeit des Todes erkennen. Ein Paralytiker, in dessen Hirn sich etwa massenhaft amöboide Zellen, degenerierende Achsenzylinder, Ganglienzellenveränderungen in der Form der schweren Zellveränderung Nissls nachweisen lassen, ist wohl an seiner Hirnkrankheit verstorben, vielleicht auch in schwerer geistiger Störung an einer Pneumonie, Sepsis oder anderen interkurrenten, körperlichen Krankheit und ein Kranker, bei dem große Ganglienzellreihen und zahlreiche Markfasern in der Hirnrinde zugrunde gegangen sind, hat ebenso sicher keine vollständige psychische Remission geboten, wie er keine Patellarreflexe gehabt hat, wenn seine Hinterstränge vollständig degeneriert waren. Ebenso ist die Behauptung Reichardts nicht ganz zutreffend, die pathologische Anatomie könne keinen Aufschluß darüber geben, was in den paralytischen Anfällen im Gehirn vor sich gehe. Starlinger hat schon 1895 den Nachweis erbracht, daß gewisse apoplektiforme Anfälle bei der Paralyse mit einem ausgedehnten Markzerfall in bestimmten Rindengebieten einhergehen, und durch die Untersuchungen von Lissauer und mir über die atypische Paralyse, die ich seitdem noch immer fortgesetzt habe, ist wohl der histologische Nachweis geliefert, daß wir in den apoplektiformen Anfällen der Paralytiker den Ausdruck eines besonders stürmischen Krankheitsfortschrittes an besonderen Stellen der Hirnrinde zu sehen haben. Aber auch bei gehäuften epileptiformen paralytischen Anfällen läßt sich meistens aufs deutlichste feststellen, daß der paralytische Krankheitsvorgang eine erhebliche akute Steigerung erfahren hat, die sich in eben den Gewebsveränderungen äußert, die in dieser Abhandlung geschildert sind, mit besonderen Modifikationen (Gefäßinfiltrationen usw.), die der Paralyse zugehören. Auch im Status epilepticus der genuinen Epilepsie, behaupte ich, regelmäßig bestimmte Veränderungen zu finden. Es ist in der vorliegenden Arbeit so oft auf sie hingewiesen worden, daß ich ihre Wiederholung hier dem Leser ersparen will.

Es erscheint mir nun eine müßige Frage, ob man sich an der Hand dieser histologischen Befunde oder aus den Ergebnissen Reichardtscher Wägungen eine bessere Vorstellung davon machen kann, was nun eigentlich während der Anfälle im Gehirn vor sich geht. Wir, die wir gewohnt sind, uns viel in anatomischen Vorstellungen zu bewegen, werden jedenfalls viel mehr befriedigt sein, wenn wir die Erscheinungen einigermaßen mit anatomischen Veränderungen in Einklang bringen können; wir Mediziner haben ja auch sonst gelernt, unsere allgemein medizinischen Anschauungen an pathologisch anatomische Vorstellungen anzuknüpfen. Wenn ich weiß, daß jemand an einer interstitiellen Nephritis leidet, kann ich mir mehr dabei vorstellen, als wenn ich feststellen könnte, daß seine Niere 50 g weniger wiegt, als vor der Erkrankung. Für einen Forscher, der mit physikalischen Methoden die Erklärungen klinischer Erscheinungen sucht, mag das vielleicht anders liegen.

Nun behauptet Reichardt an anderer Stelle, daß bei der Epilepsie, der Katatonie und gelegentlich, wenn auch seltener, bei der Paralyse, die den Anfällen zugrunde liegenden Veränderungen momentan entstehen und momentan spurlos, restlos verschwinden. Diese Auffassung würde zur Folge haben müssen, daß bei einem epileptischen Anfalle z. B. in wenigen Sekunden die anatomischen Veränderungen sich einstellen, kurze Zeit andauern und in wenigen Sekunden sich zurückbilden müßten. Von dem aber nun, was wir über pathologische Gewebsveränderungen kennen, wissen wir, daß sie keineswegs so blitzartig kommen und gehen, wie das Symptomenbild eines epileptischen Anfalls. Das würde also schon unwahrscheinlich machen, daß epileptische Anfälle mit anatomischen Veränderungen einhergehen. Nun wissen wir aber durch die klinische Beobachtung, daß solche Anfälle keineswegs immer blitzartig kommen und spurlos vorübergehen. Was die Paralyse betrifft, so kann man gar nicht so selten den Anfall Stunden vorher kommen sehen. Wernicke hat dann schon behauptet, daß jeder paralytische Anfall eine Zunahme der Demenz bedeute, und wenn man auch Fällen begegnet, in welchen diese Zunahme kaum merklich scheint, so sieht man doch nach anderen einen ungemein auffälligen Fortschritt der Verblödung.

Es ist auch durchaus nicht für die meisten Anfälle von Epilepsie zutreffend, daß sie momentan entstehen und momentan restlos verschwinden; recht häufig geht den Anfällen schon ein auf Stunden, selbst Tage sich erstreckendes Unbehagen, eine mehr oder minder schwere Verstimmung voraus. Andererseits sieht man nicht selten

noch Stunden, sogar Tage nach den Anfällen allerlei Residuen, wenn man nur sorgfältig untersucht; Erschwerungen auf sprachlichem Gebiet, Verlangsamung und Erschwerung des Gedankenganges überhaupt, Störungen in der Sensibilität, BABINSKIsches Phänomen und auch für die Epilepsie gilt, wenn schon im geringeren Grade, der Satz, daß vielleicht nicht der einzelne Anfall, aber eine Häufung derselben eine Schädigung der Intelligenz zur Folge hat. Schon öfters sind mir jugendliche Epileptiker begegnet, von denen Eltern und Lehrer versicherten, daß mit den ersten Anfällen eine auffallende Veränderung des psychischen Zustandes des Kranken eingetreten sei.

Schwerere katatonische Anfälle habe ich in den 20 Jahren meiner psychiatrischen Tätigkeit noch nicht sehr zahlreich gesehen. Einige Fälle von Dementia praecox mit schweren epileptiformen Anfällen verfielen ganz besonders schnell in tiefen katatonischen Blödsinn. Noch auffälliger trat ein eigenartiges Vorstadium bei epileptiformen Anfällen, welche bei den akuten Formen der endarteritischen Lues vorkommen, zutage. Eine eigenartige ratlose Ängstlichkeit bei einem leicht dämmerigen Bewußtseinszustand machte es wiederholt möglich, den Eintritt eines epileptiformen Anfalls schon am Tage vorher zutreffend vorauszusagen.

So zeigen unsere ganz groben klinischen Beobachtungen schon, daß die Anfälle sich vorbereiten und daß eine keineswegs momentane Wiederherstellung und meist auch keine Wiederherstellung zum früheren Zustande stattfindet. Exaktere Untersuchungen, besonders auch mit psychophysischen Meßapparaten würden das sicher noch deutlicher dartun.

Dafür sprechen nun auch einzelne histologische Befunde, die freilich noch weiterer Ergänzung bedürfen, da man ja nur selten ganz einwandfreie Fälle zur Untersuchung bekommen wird. Dafür, daß der Anfall erst nach einer gewissen Ausbildung histologischer Veränderungen zur Entwicklung kommt, spricht das Vorhandensein zahlreicher amöboider Gliazellen im Hirnmantel eines Epileptikers, der im Bade, offenbar in einem epileptischen Anfall, ertrunken war, sowie der Nachweis sehr weitgehender Veränderungen, ja selbst schon eines Zerfalles von Achsenzylindern im Gehirn eines Epileptikers, der nach einem Status von nur wenigen Stunden Dauer gestorben war. Der Nachweis zerfallender Achsenzylinder im Gehirn von im Status epilepticus Verstorbenen macht uns verständlich, daß schwere Anfälle eine Schädigung der Intelligenz zur Folge haben. Dafür, daß mit der Beendigung des Anfalls nicht alle Gewebsveränderungen beseitigt sind, spricht die Beobachtung, daß sich in den Hirnrinden von solchen Epileptikern, die an interkurrenten Erkrankungen gestorben sind und in der letzten Zeit vor dem Tode, wenn auch nicht an dem letzten Tage, viele Anfälle gehabt hatten, Gewebsveränderungen feststellen lassen, die mit den Anfällen im Zusammenhang stehen dürften.

Nun scheint es mir aber auf Grund verschiedener Erwägungen gar nicht wahrscheinlich, daß der epileptische Anfall selbst durch ganz bestimmte morphologische und qualitative Veränderungen des Gehirns verursacht wird. Wenn man sich überlegt, wie ganz außerordentlich verschiedene Erkrankungszustände des Gehirns mit Anfällen einhergehen, die in ihrer Erscheinung keinerlei durchgreifende Unterschiede zeigen (Tumoren, von der genuinen Epilepsie abweichende Epilepsieformen, Eklampsie, Paralyse, Hirnlues, Arteriosklerose, Urämie, Alkoholismus, Kokainismus, Bleivergiftung, bei den Tieren dann noch eine ganze Menge von experimentellen Vergiftungen), und daß wir dann bei der anatomischen Untersuchung solcher Gehirne ganz abweichende Veränderungen finden, ja daß wir selbst bei der Hysterie Anfälle sehen, bei denen es auch den besten Diagnostikern nicht möglich ist, sie von epileptischen Anfällen zu unterscheiden, muß es wohl sehr wahrscheinlich werden, daß die epileptischen und epileptiformen Anfälle durch einen schon im Gehirn vorbereiteten Mechanismus zur Auslösung gebracht werden und daß den Anstoß zu diesen Auslösungen sehr verschiedenerlei Schädigungen geben können. Jedenfalls wird uns dieser in seinem Wesen nach wie vor dunkle Vorgang um nicht vieles verständlicher durch die Vorstellung, daß er durch eine blitzartig sich entwickelnde und nach wenigen Minuten bereits restlos verschwundene Änderung in der Hirnmaterie verursacht wird.

In einem anderen Punkte stimme ich REICHARDT völlig bei. Auch ich bin der Meinung, daß man sich mit der Todesursache „Herz- oder Atmungslähmung" bei Geisteskranken nicht so leicht zufrieden geben soll, sondern daß man nach Veränderungen im Hirnmantel suchen muß. Ich glaube nur, man wird sie auch jetzt schon sehr häufig mit dem Mikroskope nachweisen können.

In REICHARDTS Aufsatz über die Hirnmaterie finden wir dann noch öftere Hinweise auf die Mängel und Fehlerquellen anatomischer Untersuchungen. Ich gebe zu, daß sie durchaus richtig, z. T. jedoch

mit etwas starken Farben dargestellt sind. Das meiste aber, auf das REICHARDT versichert, wieder einmal hinweisen zu müssen, ist wohl jedem Anatomen durchaus geläufig. Wir alle wissen, daß wir in unseren Präparaten kein unverändertes Gewebe vor uns haben, sondern daß wir mit Äquivalentbildern arbeiten müssen unter ständigem Vergleichen, wie dieselben Methoden normale und pathologische Gewebsbestandteile uns darstellen. NISSL, der eigentliche Vater der pathologischen Anatomie der Hirnrinde, hat das an vielen Stellen immer wieder betont Daß wir aber trotzdem auf diesem Wege zu tieferen Erkenntnissen gelangen können, das beweist der Entwicklungsgang der pathologischen Anatomie des übrigen Körpers, die ja mit den gleichen Schwierigkeiten arbeitet.

Ein Gefühl der Resignation hat mich nie beschlichen. Die Kenntnis der außerordentlichen Schwierigkeiten des Arbeitsgebietes haben mich aber auch abgehalten, je zu hoffen, daß das Mikroskop in naher Zeit der Psychiatrie alle Rätsel lösen wird. Wenn ich zusammenstelle, was die pathologische Anatomie des Zentralnervensystems seit dem letzten Jahrzehnt des vorigen Jahrhunderts geleistet hat, kann ich wohl sagen: ein gutes Stück Arbeit. Ich will die Namen und Arbeiten hier nicht anführen, weil ich wichtiges zu vergessen fürchte. Die physikalische Untersuchung des Gehirns ist gewiß auch berufen, mancherlei Erkenntnisfortschritte herbeizuführen und so haben wir allen Grund uns über diese neue Forschungsmethode zu freuen. RIEGERS und REICHARDTS Verdienste sollen nicht verkleinert werden; aber sie dürften auch bestehen können ohne Herabsetzung des Wertes der pathologischen Anatomie. Die Hirnphysiker mögen wägen; wir, die wir der Psychiatrie mit dem Mikroskope weiterhelfen wollen, werden uns damit in unserer Arbeit nicht stören lassen. Ich bin sicher, wir werden die Wage nicht zu fürchten brauchen.

Literaturverzeichnis.

ACHÚCARRO, Zur Kenntnis der pathologischen Histologie des zentralen Nervensystems bei Tollwut. Nissls histol. u. histopathol. Arbeiten, Bd. III.

ALTMANN, Die Elementarorganismen und ihre Beziehungen zu den Zellen. Leipzig 1874.

ALZHEIMER, Histologische Studien zur Differentialdiagnose der progressiven Paralyse. Nissls histol. u. histopathol. Arbeiten, Bd. I.

Ders., Über den Abbau des Nervengewebes. Zeitschr. f. Psych. 1906.

BENDA, Neurogliafärbung. Enzykl. d. mikrosk. Technik. Berlin u. Wien 1903.

BETHE, Allgemeine Anatomie und Physiologie des Nervensystems. Leipzig 1903.

BEST, Die Karminfärbung des Glykogens und des Kerns. Zeitschr. f. Mikroskopie, Bd. XXIII.

BONFIGLIO, Circa le alterazioni della cortecia cerebrale conseguenti ad intossicazione sperimentale de carbonato di piombo. Nissls histol. u. histopathol. Arbeiten, Bd. III.

CASAMAJOR, Zur Histochemie der Ganglienzellen der menschlichen Hirnrinde. Obersteiners Arbeiten, Bd. XVIII.

CERLETTI, Contributo sperimentale alla conoscenza dei processi di fagocitosi nella sostanza cerebrale. Ann. dell Istituto psich. Roma 1902.

Ders., Sulla neuronofagia e sopra alcuni rapporti normali e patologici frea elementi nervosi ed elementi non nervosi. Ann. dell Istituto psich. Roma 1903.

Ders., Sopra speciali corpuscoli perivasali nella sostanza cerebrale. Rivista sperimentale, Vol. XXXIII.

DONAGGIO, Colorazione positiva della fibre nervose etc. Rivista sperimentale, Vol. XXX.

ELZHOLZ, Zur Kenntnis der Veränderungen des zentralen Stumpfes lädierter Nerven. Jahrb. f. Psychol., Bd. XVII.

Ders., Über einen eigentümlichen histologischen Befund im zentralen Stumpf von durch Gangrän zerstörter peripherer Nerven. Monatsschrift f. Psychol. u. Neurol. 1899.

ELZHOLZ, Zur Histologie alter Nervenstümpfe in amputierten Gliedern. Zeitschr. f. Psychol. 1900.

EISATH, Über die normale und pathologische Histologie der menschlichen Neuroglia. Monatsschr. f. Psychol. u. Neurol., Bd. XX.

FARRAR, On the Phenomena of Repair in the cerebral Cortex. Nissls histol. u. histopathol. Arbeiten, Bd. II.

FORSTER, Experimentelle Beiträge zur Lehre der Phagozytose der Hirnrindenelemente. Nissls histol. u. histopathol. Arbeiten, Bd. II.

GALEOTTI, Über die Granulationen in den Zellen. Intern. Monatsschr. f. Anat. u. Physiol. 1895.

HEIDENHAIN, Plasma und Zelle. Jena 1907.

HELD, Über den Bau der Neuroglia und über den Stand der Lymphgefäße in Haut und Schleimhaut. Abhandl. d. mathem.-phys. Klasse d. sächs. Ges. d. Wiss. Leipzig 1903.

Ders., Zur weiteren Kenntnis der marginalen Neuroglia. Verhandl. d. Ges. Deutscher Naturf. u. Ärzte. Leipzig 1908.

Ders., Die Entwicklung des Nervengewebes bei den Wirbeltieren. Leipzig 1909.

LEVI, Contributo alla fisiologia della cellula nervosa. Rivista di pathologia nervosa e mentala 1896, Vol. I.

MARINESCO, Études sur l'évolution et involution de la cellule nerveuse. Revue neurol. 1899.

Ders., La cellule nerveuse. Paris 1909.

MERZBACHER, Untersuchungen über die Morphologie und Biologie der Abräumzellen im zentralen Nervensystem. Nissls histol. u. histopathol. Arbeiten, Bd. III.

DE MONTET, Über Wanderungen lipoider Substanzen im Zentralnervensystem. Inaug.-Diss. Tübingen 1906.

NISSL, Kritische Besprechung von H. Schmaus: Vorlesungen über die pathologische Anatomie des Rückenmarks. Zentralbl. f. Nervenheilk. 1903.

Ders., Über einige Beziehungen zwischen Nervenzellenerkrankungen und gliösen Erscheinungen bei verschiedenen Psychosen. Arch. f. Psychiat., Bd. XXXII.

Ders., Zur Histopathologie der paralytischen Rindenerkrankung. Nissls histol. u. histopathol. Arbeiten, Bd. I.

OBERSTEINER, Über das hellgelbe Pigment in den Nervenzellen und das Vorkommen weiterer fettähnlicher Körper im Zentralnervensystem. Obersteiners Arbeiten, Bd. X.

Ders., Weitere Bemerkungen über die Pigmentkörnchen im Zentralnervensystem. Obersteiners Arbeiten, Bd. XI.

OLMER, Recherches sur les granulations de la cellule nerveuse. Lyon 1901.

OPPENHEIM, Zur pathologischen Anatomie der multiplen Sklerose mit besonderer Berücksichtigung der Hirnrindenherde. Neurol. Zentralblatt 1908.

PERUSINI, Über klinisch und histologisch eigenartige Erkrankungen des späteren Lebensalters. Nissls histol. u. histopathol. Arbeiten, Bd. III.

PILCZ, Beitrag zur Lehre von der Pigmententwicklung in den Nervenzellen. Obersteiners Arbeiten 1895.

REICH, Über eine neue Granulation der Nervenzellen. Arch. f. Anat. u. Physiol. 1903.

Ders., Zur feineren Anatomie der Nervenzellen. Arch. f. Psychiat. 1909.

Ders., Über die feinere Struktur der Zelle des peripheren Nerven. Zeitschr. f. Psychiat. 1905.

Ders., Über den zelligen Aufbau der Nervenfaser auf Grund mikrohistiochemischer Untersuchungen. Journ. f. Psychol. u. Neurol., Bd. VIII.

Ders., Diskussion zu dem Vortrag von Lipschütz. Zeitschr. f. Psychiat. 1907.

Ders., Über Unterschiede im Bau der zentralen und peripheren Nervenfaser auf Grund mikrohistiochemischer Untersuchungen. Zeitschr. f. Psychiat., Bd. LXVI.

REICHARDT, Über die Untersuchungen des gesunden und kranken Gehirns mittels der Wage. Jena 1906.

Ders., Über die Hirnmaterie. Monatsschr. f. Psychiat. u. Neurol., Bd. XXIV.

Ders., Untersuchungen über das Gehirn. I. Teil. Münchener med. Wochenschr. 1909.

SAND, Le neuronophagie. Bruxelles 1906.

SCHAFFER, Weitere Beiträge zur pathologischen Histologie der familiären amaurotischen Idiotie. Journ. f. Psychol. u. Neurol. 1906.

Ders., Zur Pathogenese der TAY-SACHSschen amaurotischen Idiotie. Neurol. Zentralbl. 1905.

Ders., Beiträge zur Nosographie und Histopathologie der amaurotisch-paralytischen Idiotieformen. Arch. f. Psychiat. 1906.

Ders., Über die Anatomie und Klinik der TAY-SACHSschen amaurotisch-familiären Idiotie mit Rücksicht auf verwandte Formen. Zeitschr. zur Erforschung u. Behandlung d. jugendl. Schwachsinns 1909.

SCHRÖDER, Einführung in die Histologie und Histopathologie des Nervensystems. Jena 1908.

SPIELMEYER, Über eine besondere Form von familiärer amaurotischer Idiotie. Neurol. Zentralbl. 1906.

Ders., Klinische und anatomische Untersuchungen über eine besondere Form von familiärer amaurotischer Idiotie. Habilitationsschrift Gotha 1907.

Ders., Von der protoplasmatischen und faserigen Stützsubstanz des Zentralnervensystems. Arch. f. Psychiat., Bd. XLII.

STRANSKY, Über diskontinuierliche Zerfallsprozesse an der peripheren Nervenfaser. Journ. f. Psychol. u. Neurol. 1903, Bd. I.

STRÄUSSLER, Über eigenartige Veränderungen der Ganglienzellen und ihrer Fortsätze im Zentralnervensystem eines Falles von kongenitalen Kleinhirnatrophie. Neurol. Zentralbl. 1906.
VOGT, Über familiäre amaurotische Idiotie und verwandte Krankheitsbilder. Monatsschr. f. Psychiat. u. Neurol., Bd. XVIII.
WEIGERT, Zur pathologischen Histologie des Neurogliafasergerüstes. Zentralbl. f. allgem. Pathol. 1890.
Ders., Beiträge zur Kentnis der menschlichen Neuroglia. Frankfurt a. M. 1895.
WLASSAK, Die Herkunft des Myelins. Ein Beitrag zur Physiologie des nervösen Stützgewebes. Arch. f. Entwicklungsmechanik d. Organismen 1898, Bd. VI.

Tafelerklärung.

Allgemein angewendete Abkürzungen:

abp. = Abbauprodukte.
adv. = Adventitia.
advl. = adventitieller Lymphraum.
adz. = Adventialzelle.
aglz. = amöboide Gliazelle.
ax. = Achsenzylinder.
blz. = rotes Blutkörperchen.
cap. = Capillare.
dax. = degenerierter Achsenzylinder.
ekg. = ektodermales Gewebe.
ez. = Endothelzelle.
fk. = Füllkörperchen.
gaz. = Ganglienzelle.
glf. = Gliafaser.
glfs. = Gliazellfortsatz.
glz. = Gliazelle.
int. = Intima.
l. = Gefäßlumen.
l. k. = lipoide Körperchen.
lz. = Lymphozyth.
mkz. = mesodermale Körnchenzelle.
m. l. = membrana limitans gliae.
msch. = Markscheide.
mph. = Makrophag.
p. = Pia.
p. v. r. = perivaskulärer Raum.
r. = gliöse Randschicht.

Tafel XXVIII

enthält Zeichnungen aus Präparaten, welche mit der Methode IV gewonnen sind. Zeiss homogen. Immersion $^1/_{12}$. Okular 8.

Fig. 1 *a*, *b*. Normale Gliazellen mit protoplasmatischen Verzweigungen aus der Hirnrinde eines 34 jährigen, an einem Unglücksfall verstorbenen Mannes. *c* Gliazelle aus der Hirnrinde eines 68-jährigen, an Arteriosklerose erkrankten Mannes. *d* Gliazelle aus der tieferen Hirnrinde einer an einem Depressionszustand erkrankten 63-jährigen Frau. *e*, *f*, *g* Gliazellen aus der Markleiste eines 34 jährigen, durch einen Unglücksfall verstorbenen Mannes. *h*, *i* Gliazellen aus der Hirnrinde einer jugendlichen, verblödeten Epilepsie mit sehr gehäuften Anfällen.

Fig. 2. Amöboide Gliazellen aus der Hirnrinde eines Falles von schwerem Infektionsdelir. *a* Die Gliazellen deformieren Ganglien-Zelleib und Kern. *b* Eine amöboide Gliazelle mit zwei stummeligen Fortsätzen (vielleicht Resten ursprünglich längerer Fortsätze). *c* Eine Ganglienzelle von sechs amöboiden Zellen umlagert.

Fig. 3. Eine Kapillare aus der Hirnrinde, von jüngeren und älteren amöboiden Zellen umgeben; in den älteren Zellen beginnende Zerfallserscheinungen des Plasmas, bei *a* Zelle mit Methylblaugranula.

Fig. 4. Kleine amöboide Gliazelle aus dem Marke eines Infektionsdelirs.

Fig. 5. Kleine amöboide Gliazelle von einem plötzlich im Anfall verstorbenen Katatoniker.

Fig. 6. Größere amöboide Gliazelle mit Vakuolen (*v*), Markscheiden umfließend. Status epilepticus.

Fig. 7. *a* Kleine Gliazellen, vielleicht Übergangsformen von runden Gliazellen mit Plasmakörnchen zu amöboiden Zellen. *b* und *c* Kleine amöboide Zellen, vielleicht Übergangsform einer Gliazelle mit Plasmaausläufern zu amöboiden Zellen. *d* Große amöboide Zelle mit zwei Vakuolen (*v*), eine Manschette um eine Kapillare bildend und viele Markfasern umfassend. „Angstpsychose."

Fig. 8 *a*, *b*, *c*. Große amöboide Gliazellen aus der Hirnrinde einer sehr stürmisch verlaufenen Paralyse. In *c* Ablösuug der Kernmembran vom Kerninhalt. Staubförmig feine Methylblaugranula im Plasma. Progressive Paralyse. ·

Fig. 9. Große amöboide Gliazelle mit großem Methylblaugranula. „Angstpsychose."

Fig. 10. Große amöboide Gliazelle mit Kerndegeneration und Methylblaugranula. Status epilepticus.

Fig. 11. Gliazelle mit Methylblaugranula in der Anordnung protoplasmatischer Verzweigungen. Finales Delirium bei Phthise.

Fig. 12 und 13. Die gleichen Zellen aus der Markleiste eines plötzlich verstorbenen Katatonikers.

Fig. 14. Kleine Vene der Markleiste eines Falles von „Angstpsychose". Man sieht sehr deutlich den perivaskulären Raum und den adventitiellen Lymphraum. In letzterem neben einigen anderen Zellen zwei typische Gitterzellen (Körnchenzellen). Im perivaskulären Raum neben einer Anzahl junger Gliazellen eine junge und eine große amöboide Zelle; die letztere enthält große Methylblaugranula und einen regressiv veränderten Kern und ähnelt im übrigen sehr anderen im perivaskulären Raum liegenden kernlosen Haufen. Im Nervengewebe junge Gliazellen verschiedener Art, auch solche mit Methylblaugranula.

Tafel XXIX

stellt Präparate dar, welche mit der Methode V gewonnen sind. Zeiss homogen. Immersion $^1/_{12}$. Fig 1, 3—20 Okular 8. Fig. 2 Okular 4.

Fig. 1. Schnitt aus dem Corpus striatum nahe der Ventrikeloberfläche eines Falles von progressiver Chorea. Das Gewebe ist von Füllkörperchen durchsät, daneben sind einige amöboide Gliazellen, einige Gliakerne mit wenig Plasma, einzelne Gliafasern und Achsenzylinder zu sehen.

Fig. 2. Kleine Vene aus der Markleiste eines Falles von „Angstpsychose" mit bandartiger Entwicklung der Adventitia. Man sieht deutlich den perivaskulären Raum, in welchem neben zahlreichen Haufen von Abbauprodukten eine große amöboide Zelle liegt.

Fig. 3. Gliazelle aus der Rinde der vorderen Zentralwindung eines Falles von progressiver Chorea. Der Zelleib ist in zahlreiche grobe Methylblaugranula aufgelöst, die bandartigen Fortsätze zeigen ein noch gleichmäßig und dunkel gefärbtes Plasma.

Fig. 4, 5, 15. Ähnliche Zellen von demselben Fall, vordere Zentralwindung, mit rosenkranzähnlichen Fortsätzen, teilweise schon in einzelne Stücke zerfallen. Degenerationserscheinungen am Kern.

Fig. 6. Ganglienzelle, deren Spitzenfortsatz eine amöboide Gliazelle deformiert hat. Der Spitzenfortsatz ist kelchförmig auseinandergedrückt. Progressive Chorea. Vordere Zentralwindung.

Fig. 7. In einer Gewebslücke, welche noch die Form einer Ganglienzelle zeigt, liegen Reste einer Ganglienzelle, zwei junge amöboide Gliazellen und drei degenerierte Kerne, welche offenbar zerfallenden amöboiden Gliazellen zugehören. Derselbe Fall.

Fig. 8. Ganglienzelle, von einem großen, perizellulären Raum umgeben, in welchem Zerfallsprodukte einer amöboiden Gliazelle liegen. Derselbe Fall.

Fig. 9. Ausgangszustand der schweren Zellveränderung Nissls. Delirium acutum-artiger Zustand bei Paralyse. In der Gewebslücke, welche die Ganglienzelle ausfüllte, liegt ein degenerierter Ganglienzellkern mit abgelöster Membran und ein degenerierter Gliazellkern, dazwischen Häufchen von Granula.

Fig. 10, 11, 12 und 13. Amöboide Gliazellen, die in Ganglienzellen oder deren Fortsätzen liegen. Zentralwindung. Progressive Chorea.

Fig. 15, 16, 17 und 18. Verschiedene Formen und Zerfallsstadien amöboider Gliazellen bei progressiver Chorea.

Fig. 19. Schnitt aus der weißen Substanz des Rückenmarks einer halbwüchsigen Ziege.

Fig. 20. Schnitt aus der weißen Substanz des Rückenmarks eines Falles von progressiver Chorea.

Tafel XXX

stellt Präparate dar, welche mit der Methode VI gewonnen sind. Zeiss homogene Immersion $^1/_{12}$. Okular 12.

Fig. 1 *a*—*c*. Gliazellen aus der Hirnrinde eines Falles von epileptischem Delirium. *a* enthält nur fuchsinophile Granula, *b* und *c* fuchsinophile Granula und lipoide Cystchen.

Fig. 2. Amöboide Gliazellen aus dem Vorderhorn des Rückenmarkes einer Paralyse mit Delirium acutum-artigem Verlauf.

Fig. 3. Amöboide Gliazellen aus der Markleiste verschiedener Fälle von Status epilepticus und eigenartiger Psychosen des Rückbildungsalters. *l*, *m* kleinste Formen amöboider Gliazellen aus der Hirnrinde eines Falles von Infektionsdelir. *g*, *h*, *i*, *k* jüngere Formen; in *g* und *i* je ein Lichtgrüngranulum, sonst nur fuchsinophile Granula. *b*, *c*, *d*, *e*, *f* ältere Formen mit großen und kleineren lipoiden Cystchen. *a* Gliafaserbildende Zelle ohne deutlichen Protoplasmaleib voller lipoider Cystchen.

Fig. 4. Ganglienzellen mit amöboiden Gliazellen aus der Hirnrinde eines Delirium acutum-artig verlaufenen Falles von progressiver Paralyse. Deformation der Zellen. Ersatz des Zelleibes durch amöboide Gliazellen. Kerndegeneration an den amöboiden Zellen.

Fig. 5. Große, der Faserglia zugehörige Zelle mit einem Haufen staubförmiger fuchsinophiler Körnchen in der Mitte des Protoplasmaleibes. Progressive Paralyse.

Fig. 6. Ebensolche Zelle mit vollständigem Ersatz des Plasmas durch feinste fuchsinophile Körnchen.

Fig. 7. Kapillare aus der Hirnrinde eines Falles von epileptischem Delirium. Im perivaskulären Raum liegt ein Gliakern, von welchem aus lipoiden und fuchsinophilen Körnchen gebildete Körnchenreihen abgehen, die sich in das ektodermale Gewebe einerseits und bis an die Gefäßwand andererseits fortsetzen.

Fig. 8. Große amöboide Gliazelle mit fuchsinophilen Granula, die mit einem Teil ihres Zelleibes im Nervengewebe liegt, mit einem anderen Teil einen perivaskulären Raum überbrückt und eine Kapillare umfaßt. Status epilepticus.

Fig. 9. Kleine Vene der Hirnrinde eines Falles von epileptischem Delirium. Der perivaskuläre Raum ist durchzogen von bandartigen Brücken, in welchen fuchsinophile Granula liegen. Einige davon, die sich ins ektodermale Gewebe fortsetzen, dürften wohl als Gliazellfortsätze anzusehen sein, die anderen werden als Gerinnungsprodukte gedeutet. Im perivaskulären Raum finden sich keine lipoiden Stoffe, dagegen im adventitiellen Lymphraum und in den Zellen der Adventitia.

Fig. 10. Vene aus der Markleiste eines Falles einer tödlich verlaufenen Psychose im Rückbildungsalter. Der perivaskuläre Raum ist fast überall überbrückt von breiten Bändern, in welchen fuchsinophile Granula liegen. Die meisten enden am Nervengewebe und zeigen keine Verbindung mit Kernen. Dazwischen liegen zwei amöboide Gliazellen. Im perivaskulären Raum keine lipoiden Stoffe, starke Anhäufung derselben in großen Körnchenzellen des adventitiellen Lymphraumes.

Tafel XXXI

zeigt die mit der WEIGERTschen Gliamethode dargestellten fibrinoiden Granula. Zeiss homogene Immersion $^1/_{12}$. Okular 12 (Fig. 14, Okular 8). Methode X.

Fig. 1—7. Zellen aus der Hirnrinde. Fig. 8, 9, 11, 12. Zellen aus der Markleiste. Fig. 1, 2, 3, 12. „Angstpsychose." Fig. 4, 6, 8, 9. Plötzlicher Tod bei Katatonie. Fig. 5, 7. Finales Delirium bei Phthise. Fig. 12. Akute Dementia praecox.

Fig. 10, 13. Anhäufung fibrinoider Gerinnsel in den perivaskulären Räumen bei der „Angstpsychose".

Fig. 14. Ganglienzelle aus der dritten Zellschicht der Hirnrinde eines Falles von „Angstpsychose", eingehüllt von den mit fibrinoiden Granula erfüllten Fortsätzen einer Gliazelle.

Tafel XXXII

stellt die basophil metachromatischen Abbaustoffe dar. Zeiss homogene Immersion $^1/_{12}$. Okular 8. 6 u. 10 Alkoholschnitte, die übrigen Formolgefrierschnitte. Färbung mit Toluidinblau.

Fig. 1. Gliazellen aus der Hirnrinde und Markleiste von verschiedenen Erkrankungen mit metachromatisch basophilen Einlagerungen.

Fig. 2, 3. Ganglienzellen von Trabantzellen umlagert, die metachromatisch basophile Einlagerungen enthalten aus der tieferen Rinde eines Falles von schwerer manischer Erregung (Tod in Erschöpfung?).

Fig. 4. Umgebung einer kleinen Vene der tieferen Hirnrinde eines in schwerer manischer Erregung verstorbenen Mannes. Basophil metachromatische Stoffe in den Gliazellen, im perivaskulären Raum, und grünliche lipoide Stoffe in den Zellen der Adventitia.

Fig. 5. Kleine Vene aus der Markleiste eines Epileptikers. Man sieht im adventitiellen Lymphraum Körnchenzellen, welche zum Teil fast ganz mit basophil metachromatischen Stoffen beladen sind, zum Teil grünlich gefärbte lipoide Stoffe enthalten. Die basophil metachromatischen Stoffe finden sich auch in den Gliazellen im ektodermalen Gewebe.

Fig. 6. Längsschnitt einer Vene der Markleiste eines Falles von infektiösem Delirium. Im adventitiellen Lymphraum Lymphozyten und Zellen mit großem, mattrot gefärbten Protoplasmaleib (wohl zur Gruppe der Makrophagen gehörig). In den aus Makrophagen hervorgegangenen Körnchenzellen grünliche, gelbliche und metachromatisch basophile Stoffe (z. T. sind die fettigen Stoffe durch den Alkohol extrahiert).

Fig. 7. Zwei Gliazellen aus dem Mark einer Dementia senilis, welche basophil metachromatische und grünliche lipoide Stoffe enthalten.

Fig. 8. Vier SCHWANNsche Zellen aus einer Wurzel einer luetischen Meningomyelitis, welche die REICHschen π-Granula zeigen.

Fig. 9. Basophil metachromatische Einschlüsse in etwas gewucherten Gliazellen der weißen Substanz des Rückenmarks einer tuberkulösen Meningitis in der Form der REICHschen π-Granula. Degenerationserscheinungen am Kern der Zelle *a*; in Zelle *d* Zwiebelschalenanordnung der π-Granula.

Fig. 10. Zwei Gliazellen aus der Medulla oblongata eines Arteriosklerotikers mit basophil metachromatischen Stoffen beladen. Die Zelle rechts zeigt in ihrem unteren Teile eine wabige Struktur des Zellplasmas

Fig. 11. Drei Gliazellen aus dem Mark des Mittelhirns eines Falles von Chorea progressiva, vollbeladen mit basophil metachromatischen Produkten.

Tafel XXXIII

Alkoholschnitte Fig. 1, 5, 7, 11—13, 15. Toluidinblau-, 6. 8—10, Giemsa-, 14 Methylgrünpyroninfärbung. Zeiss homogene Immersion $^1/_{12}$. Okular 6.

Fig. 1. Verschiedene Formen degenerativ veränderter Gliakerne aus der weißen Substanz des Rückenmarks eines Falles von frischer tuberkulöser Meningomyelitis.

Fig. 2. Verschiedene Formen degenerativ veränderter Gliazellen aus der Markleiste eines Falles von septischem Delirium.

Fig. 3. Verschiedene Formen degenerativ veränderter Gliazellen aus der Hirnrinde und dem Mark verschiedener Fälle von septischem Delirium. Im Plasma der Zellen vielfach basophil metachromatische Körner und Stoffe.

Fig. 4. Gliagitterzellen (Körnchenzellen) aus der weißen Substanz des Hinter- und Seitenstranges eines Falles von progressiver Paralyse. Das Fett, welches in den kugeligen Höhlungen lag, ist vollständig extrahiert (vgl. Tafel XXXIV, Fig. 2 und 6).

Fig. 5. Gliagitterzellen in der Nähe eines Herdes in der Markleiste in einem Falle von syphilitischer (?) Enzephalitis mit vollständig leeren Kammern; Kontrollpräparate zeigen, daß sie mit großen Fetttropfen gefüllt waren.

Fig. 6. Spitzenfortsatz einer Ganglienzelle, in welche sich rundliche amöboide Zellen eingedrückt haben. Akute Katatonie. Bei *a* eine Gliazelle mit vielen Abbaukörnern (lipoider Natur).

Fig. 7. Beetzsche Pyramide von einem Falle von Delirium acutum-artig verlaufener Paralyse. Kernveränderung der schweren Zellerkrankung Nissls. Im Spitzenfortsatz und an einem daneben liegenden Protoplasmafortsatz basophile Körner. An der Basis der Zelle und um den Achsenzylinderfortsatz tropfenförmige Anhäufungen einfach basophiler Produkte. Ebenso auf dem Zelleib der amöboiden Gliazellen, welche um die Ganglienzelle herumliegen.

Fig. 8. Ganglienzelle von fünf amöboiden Zellen umlagert, der Kern durch den Andrang einer amöboiden Zelle deformiert. In dieser Gliazelle beginnende Kerndegeneration. Von einer eigenartigen schweren Erkrankung im Klimakterium.

Fig. 9. Ganglienzelle, in deren Zelleib eine amöboide Zelle eingedrungen ist. Der Ganglienzellkern ist deformiert und verlagert. Karyorhexis des Gliakerns. An den Kernen der anderen amöboiden Zellen gleichfalls regressive Veränderungen. Vom gleichen Fall.

Fig. 10. Deformierung der Ganglienzelle und ihres Kernes durch andrängende amöboide Zellen.

Fig. 11. Ganglienzelle mit Trabantzellen von einem Falle von akuter Katatonie. Die eine amöboide Gliazelle liegt in einer großen Nische des Ganglienzelleibes, welchen sie nicht ausfüllt; an den Rändern der Nische einfach basophile Produkte. Der eine Gliakern zeigt regressive Veränderungen (Maulbeerform).

Fig. 12. *a* Einfach basophile Körnchen, aus dem Zerfall von Plasmafortsätzen der Ganglienzellen hervorgegangen, zu kokkenartigen Reihen angeordnet. *b* Haufen basophiler Körnchen aus einem perivaskulären Raum. *c* Stück eines Zelleibes einer Ganglienzelle, in einfach basophile Körnchen sich auflösend. *d* Rest einer Ganglienzelle, in basophile Krümel zerfallend. Von einem Falle von urämischer Psychose.

Fig. 13. Stück aus einem Kern der Regio subthalmacica eines Falles von progressiver Chorea. Zwei Ganglienzellen in basophile Produkte sich auflösend, dazwischen Dauerformen amöboider Gliazellen und basophile Abbauprodukte, welche eigentümliche Schiffchenformen zeigen.

Fig. 14. Kapillare mit perivaskulärem Raum aus der Hirnrinde eines Falles von Angstpsychose. Im perivaskulären Raum liegen amöboide Gliazellen und eigentümliche, rotgefärbte Massen, welche pathologische Gerinnungsprodukte darstellen.

Fig. 15. Drei eingesargte, abgestorbene Ganglienzellen mit schweren Kernveränderungen und nahezu ungefärbtem Plasma, von Gliazellen eingewickelt, die nicht den Typus amöboider Zellen zeigen. (Totenladenbildung.) Aus der Markleiste eines Falles von endarteritischer Lues mit vielen Erweichungsherden.

Tafel XXXIV.

Herxheimersche Präparate. Zeiss homogen. Immersion $^1/_{12}$. Okular 6.

Fig. 1. Gliazellen mit lipoiden Einlagerungen aus der Hirnrinde eines Falles von endarteritischer Hirnlues.

Fig. 2. Gliazelle, dem Alkohol-Toluidinblaubild Tafel XXXIII, Fig. 5*a* entsprechend. Rückenmark. Progressive Paralyse.

Fig. 3. Gliazellen mit massenhaften lipoiden Einlagerungen aus dem Mark des Schläfelappens eines Falles von atrophischer Sklerose.

Fig. 4. Aus einem Schnitt durch die Hirnrinde eines Falles von „Angstpsychose“. In den Ganglienzellen verhältnismäßig wenige lipoide Körnchen (ein Teil der Fettkörnchen liegt zudem noch auf den Ganglienzellen und dürfte zu Gliazellen gehören). Mehr lipoide Stoffe liegen in Gliazellen und besonders viel in den Zellen der Adventitia.

Fig. 5. Aus einem Schnitt durch die Hirnrinde eines Falles der Warren Tay-Sachsschen amaurotischen Idiotie. In den Ganglienzellen wenig staubförmig fein verteiltes Fett, in den Gliazellen große Fetthaufen, besonders reichliche Fettanhäufung in der Adventitia.

Fig. 6. Verschiedene Entwicklungsstadien der Gliakörnchenzellen im Rückenmark. Tuberkulöse Meningitis. *f*, *g* Kleine, fettbeladene Gliazellen, innerhalb der noch erhaltenen Markscheide ge-

legen. *a, c, d, e* Allmähliche Anreicherung der Zellen mit Fettkörnchen. *b* Fertige Fettkörnchenzelle.

Fig. 7. Aufspeicherung lipoider Stoffe in den adventitiellen Zellen einer kleinen Vene der Markleiste von einem Falle von Epilepsie.

Tafel XXXV.

Fuchsinlichtgrünfärbung. Zeiss homogen. Immersion $^1/_{12}$. Fig. 1—5 Okular 6, Fig. 6 Okular 4.

Fig. 1. Vorderhornganglienzelle eines normalen, älteren Hundes. In der Zelle sind die grünen Nisslschollen und dem Zellplasma angehörige fuchsinophile Granula sichtbar. An zwei Stellen liegen lipoide Körnchen. Am Rand der Zelle und den Fortsätzen sind die rot gefärbten Neurosomenhäufchen sichtbar. An der Kapillare lipoide Stoffe. Sonst sieht man im Gewebe zahlreiche Markscheiden und Achsenzylinder. Das Übrige wird zum größten Teil ausgefüllt von Protoplasmafortsätzen benachbarter Ganglienzellen, die alle von Neurosomenhaufen umlagert sind.

Fig. 2. Verödete Partie aus dem Vorderhorn eines Falles von progressiver Chorea. In der Mitte eine Ganglienzelle, deren Kern nicht geschnitten ist, mit reichlichen lipoiden Einlagerungen. Neurosomenhäufchen sind nur noch ganz vereinzelt festzustellen. Achsenzylinder und Protoplasmafortsätze von Ganglienzellen sind kaum noch sichtbar, dagegen ist das ganze Gewebe durchsät von grünlichen, rundlichen Körperchen (Füllkörperchen).

Fig. 3. Aus einem Schnitt durch das Rückenmark eines an einer Bakterieninfektion mit aufsteigender Lähmung gestorbenen Feldhasen. Grenze zwischen Hinterhorn und Hinterstrang. In der Nachbarschaft einer Kapillare liegen zum Teil im perivaskulären Raum, zum Teil im Nervengewebe sehr zahlreiche größere Füllkörperchen und mitten unter ihnen größere amöboide Gliazellen mit fuchsinophilen Granula und lipoiden Körnchen. Füllkörperchen sind durchs ganze Gewebe zerstreut.

Fig. 4. Schnitt durch die oberflächliche Randschicht des Rückenmarkes eines Falles von akuter progressiver Paralyse, der an Sepsis zugrunde ging. Statt einer aus Gliafasern gebildeten Oberflächenschicht sehen wir eine Unmenge von Füllkörperchen, die auch noch in kleinen Häufchen zwischen den Markfasern liegen.

Fig. 5. Schnitt durch die Randschicht des Rückenmarkes eines alten Falles von Paralyse. Die stark verdickte Oberflächenschicht wird fast ausschließlich aus hier quer getroffenen Gliafasern gebildet.

Fig. 6. Schnitt durch den stark verödeten Streifenhügel eines Falles von progressiver Chorea. Ein zartes Gliaretikulum, von einer Art amöboider Zellen gebildet, durchzieht das ganze Gewebe.

Buchdruckerei Ant. Kämpfe, Jena.

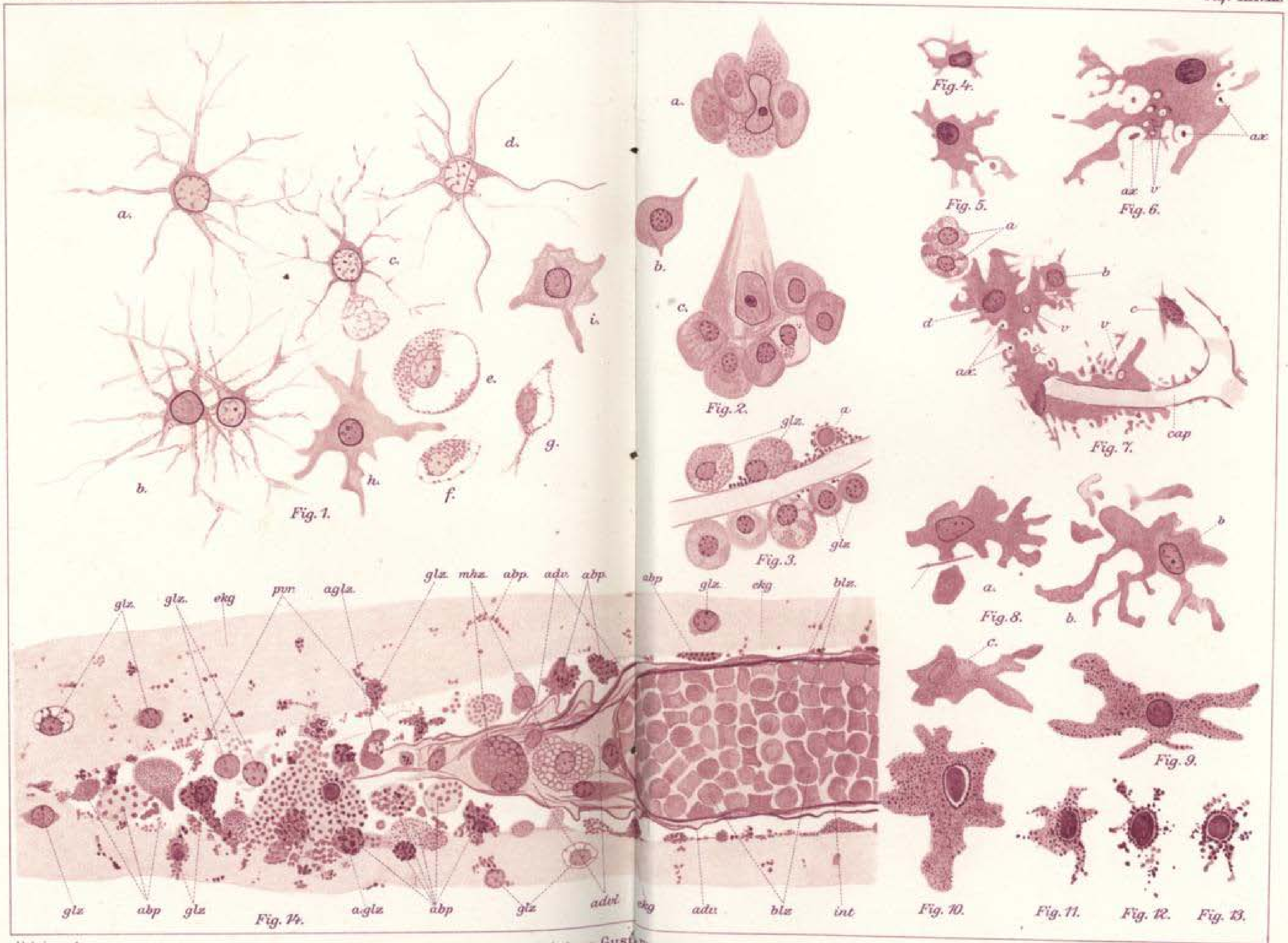

Alzheimer gez.

Verlag von Gust[illegible] Fischer in Jena.

Lith.Anst.v.K.Wesser,Jena.

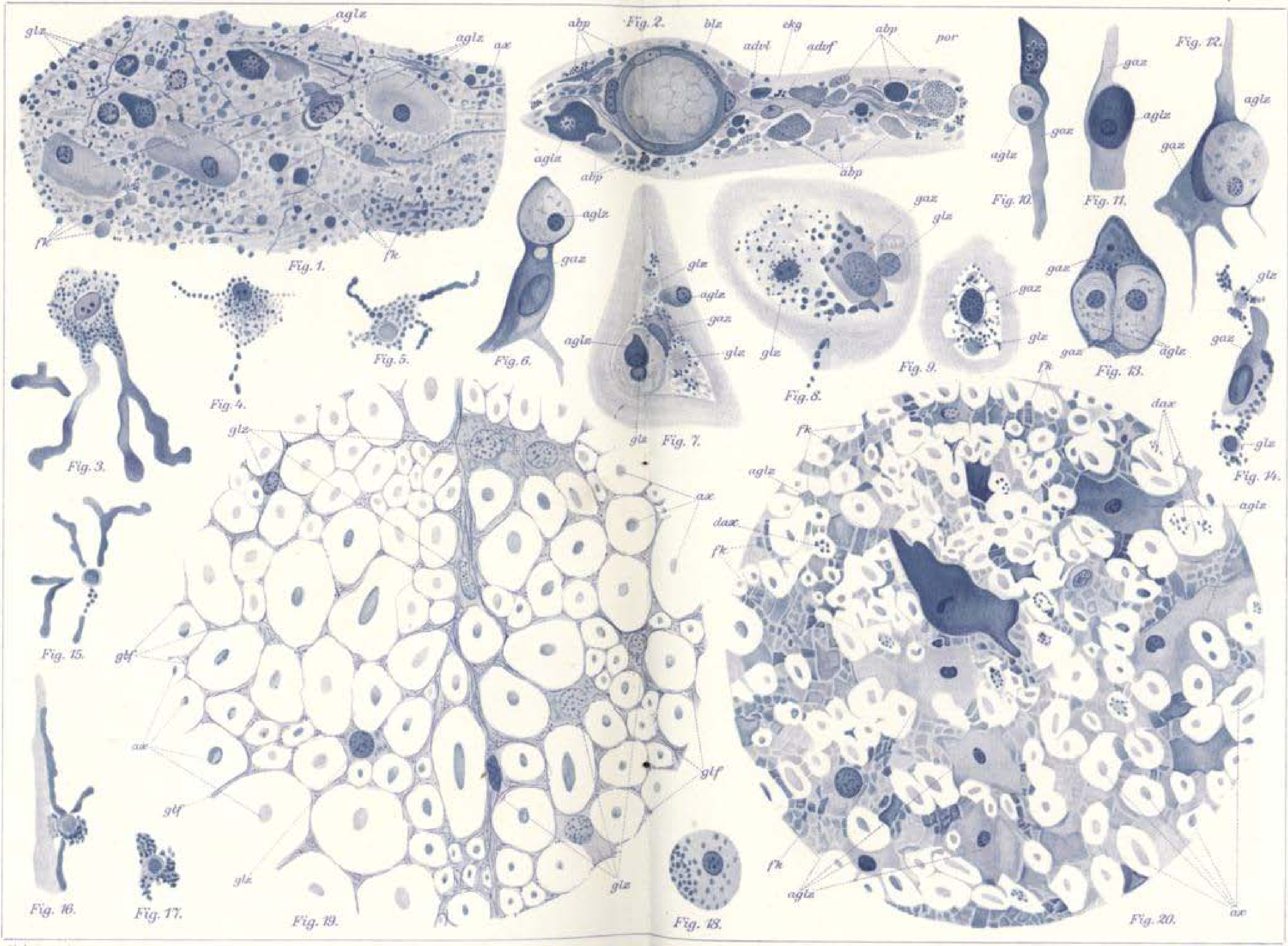

Alzheimer gez.

Verlag von Gustav Fischer in Jena.

Lith.Anst.v.K.Wesser, Jena.

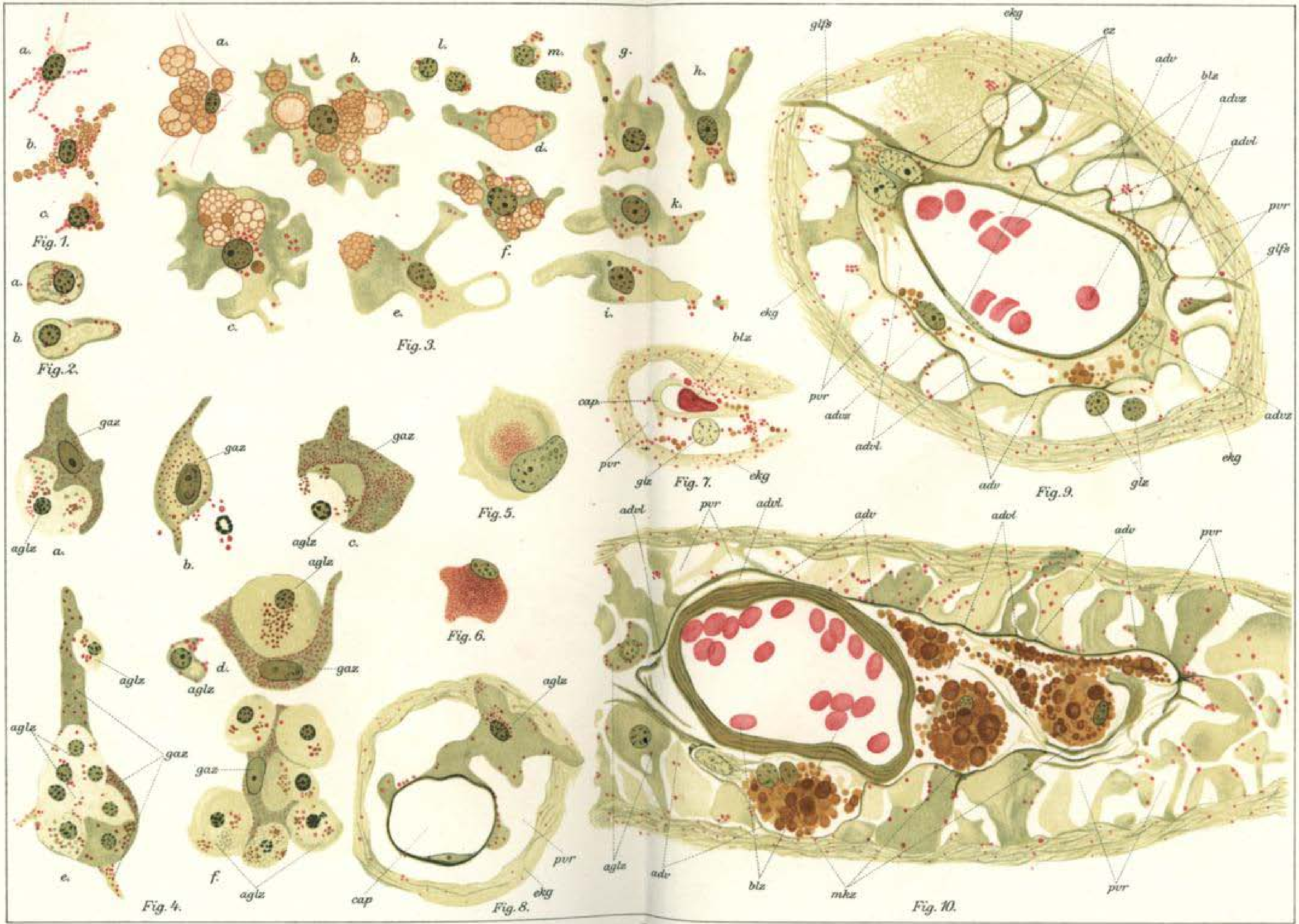

Alzheimer gez.

Verlag von Gustav Fischer in Jena.

Lith. Anst. v. K. Wesser, Jena.

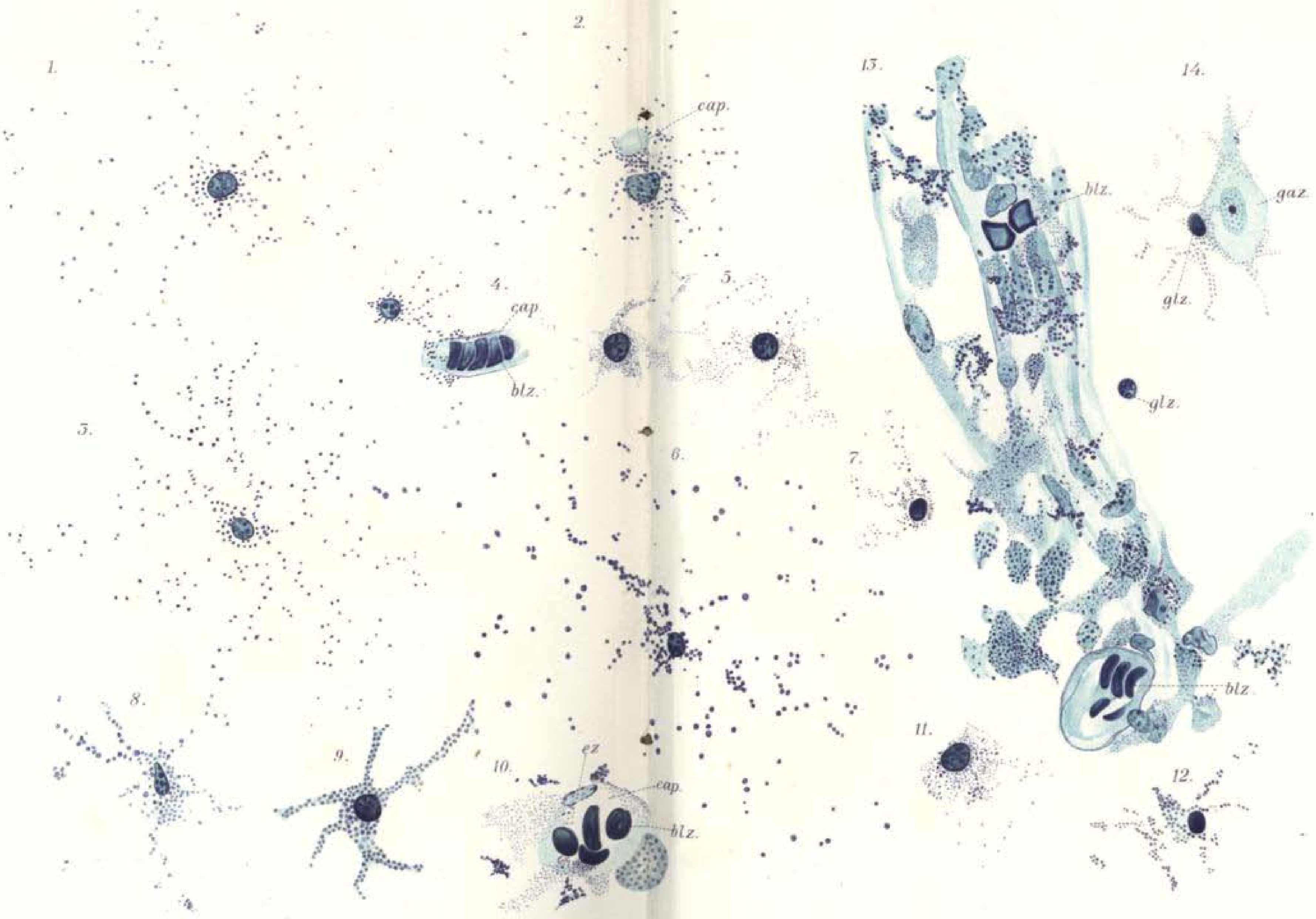

gez. Kiessling. Verlag von Gustav Fischer in Jena. Lith. Anst. v. A. Giltsch, Jena.

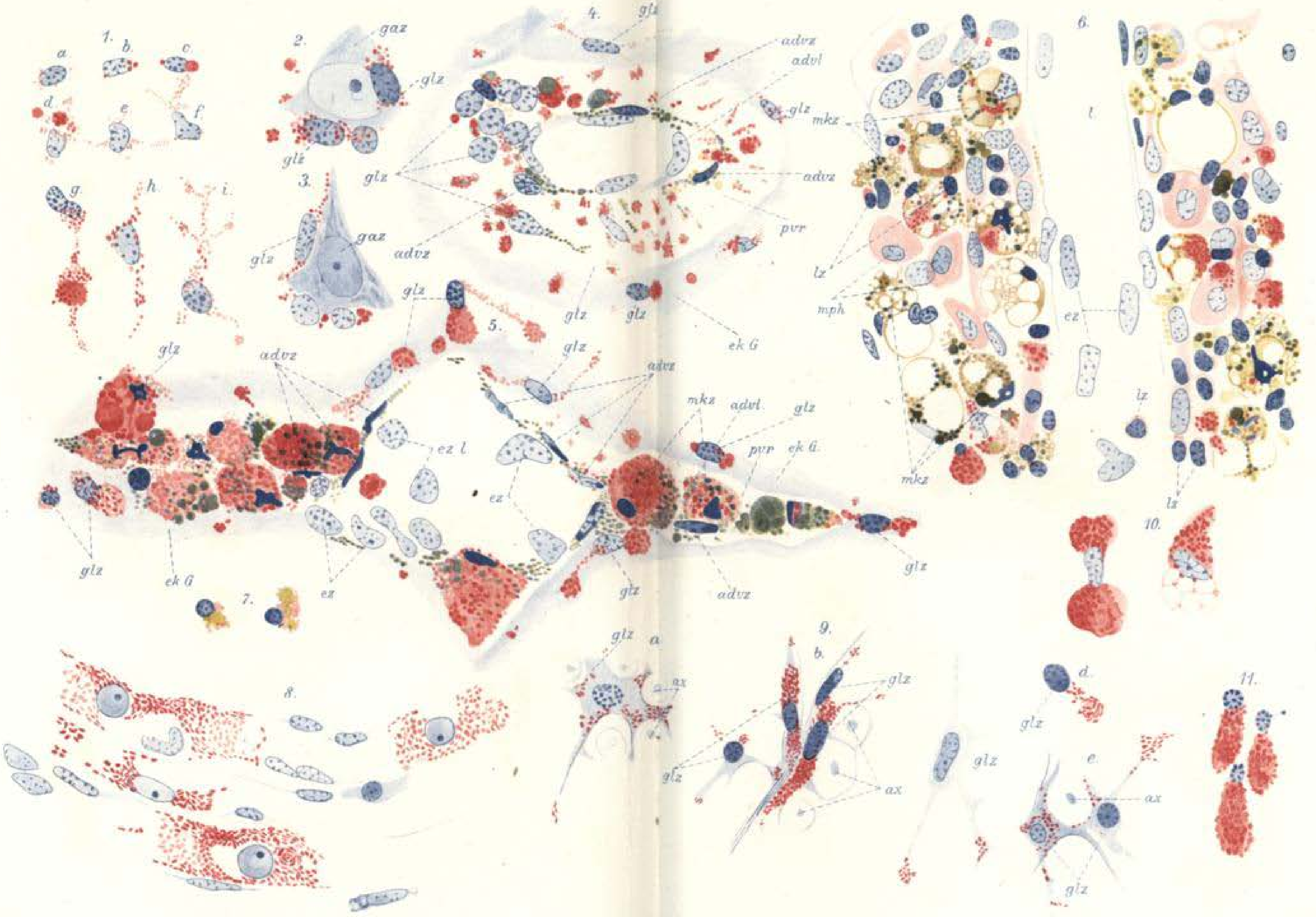

gez. Alzheimer.

Verlag von Gustav Fischer in Jena.

Lith. Anst. v. A. Giltsch, Jena.

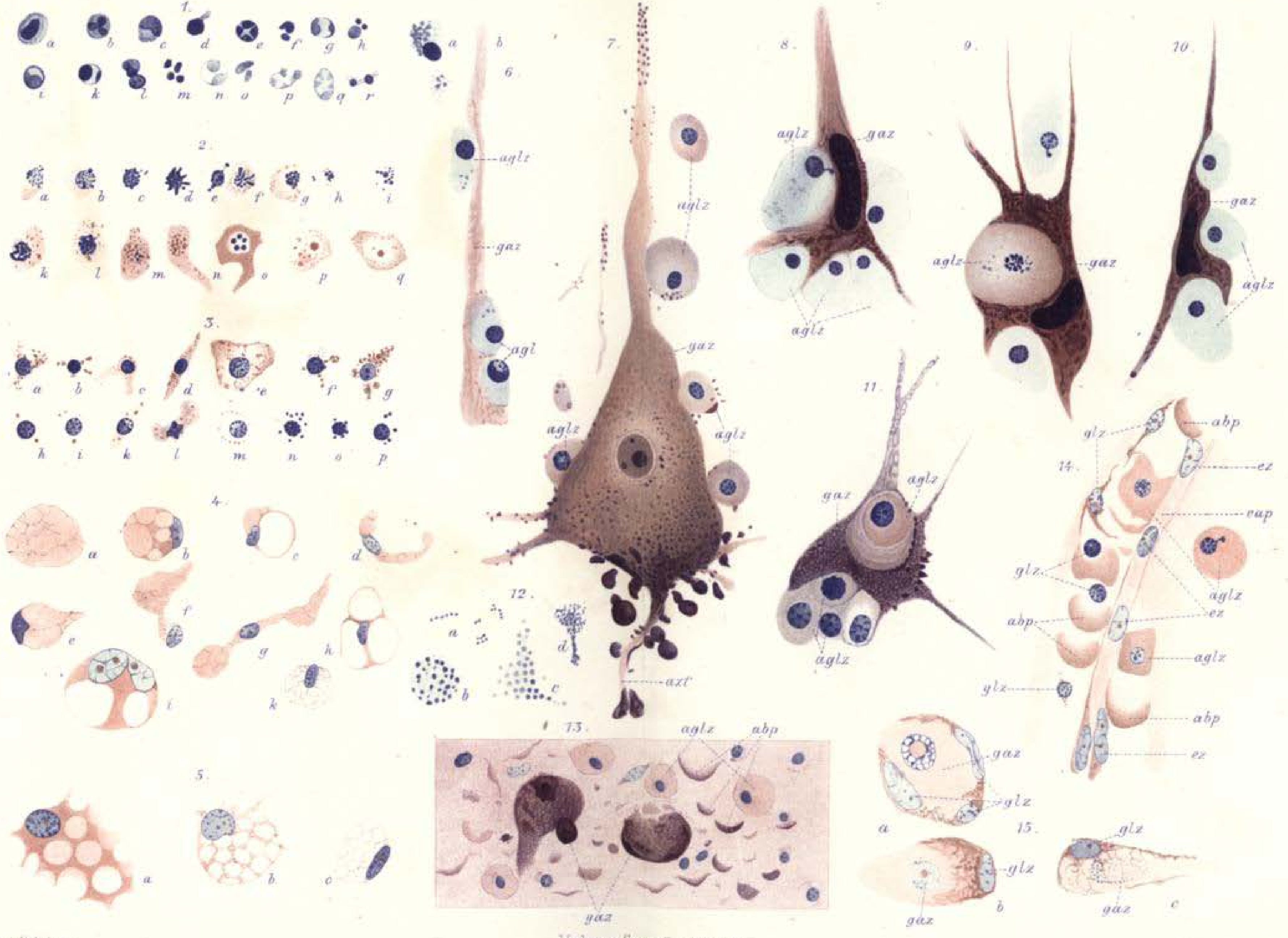

gez. Alzheimer

Verlag von Gustav Fischer in Jena.

Lith. Anst. v. A. Giltsch, Jena.

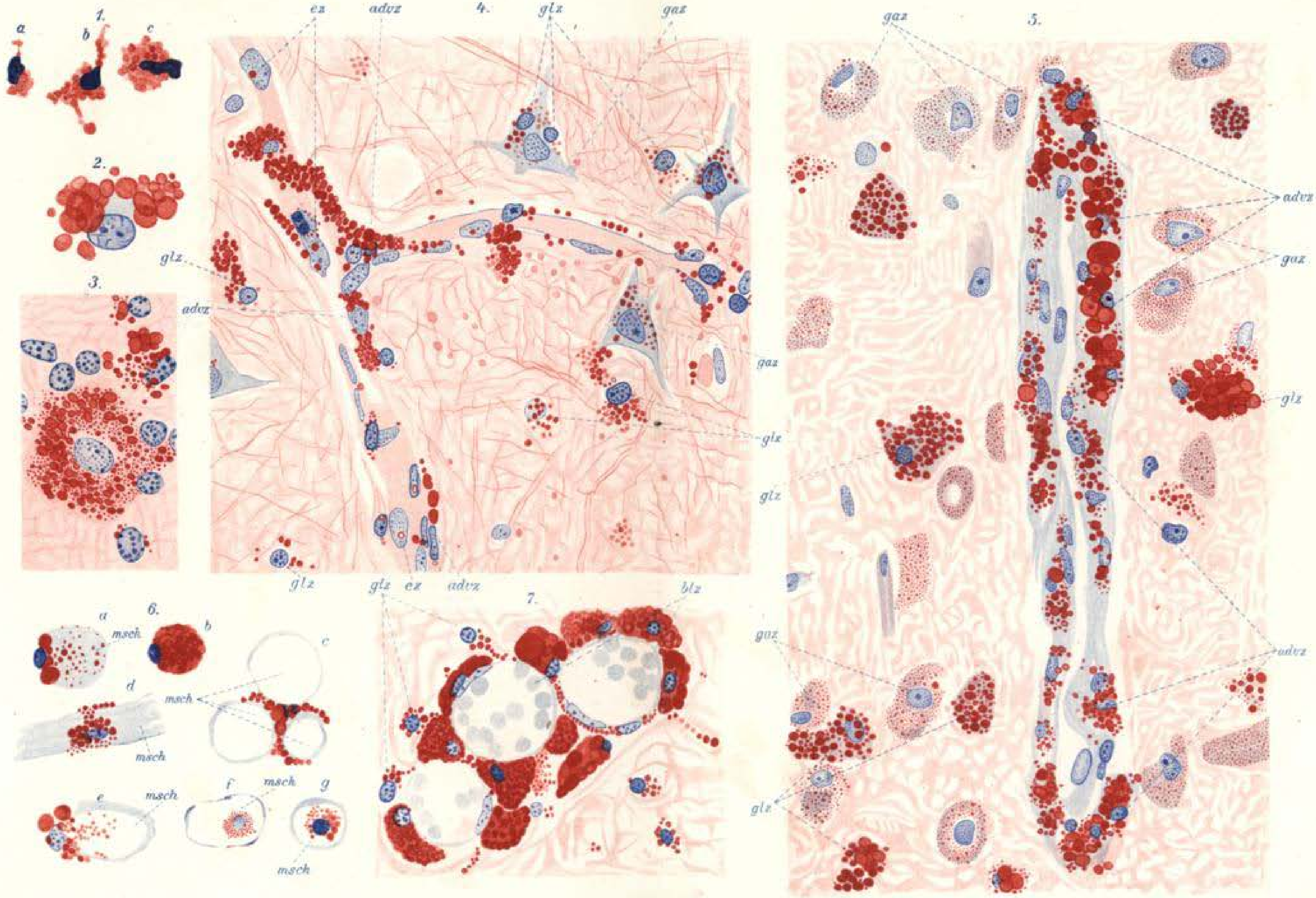

gez. Kiessling.

Verlag von Gustav Fischer in Jena.

Lith. Anst. v. A. [illegible], Jena.

1.

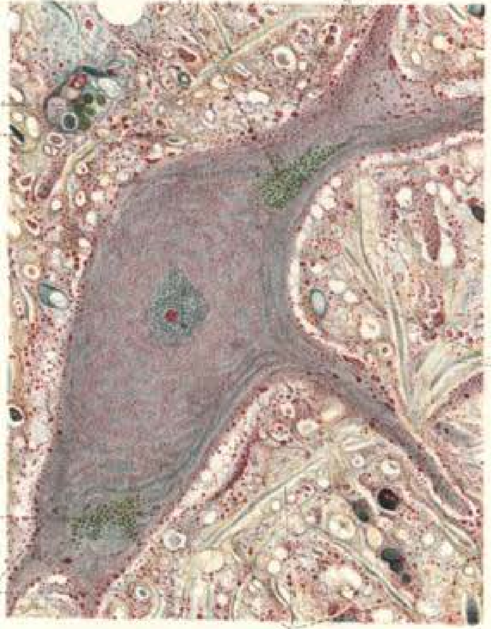

2.

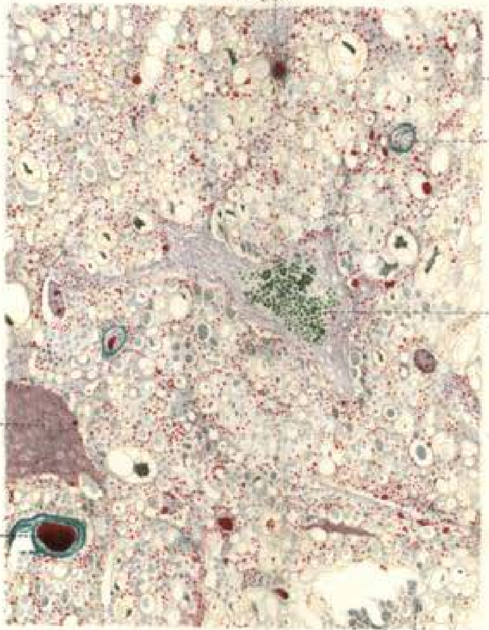

3.

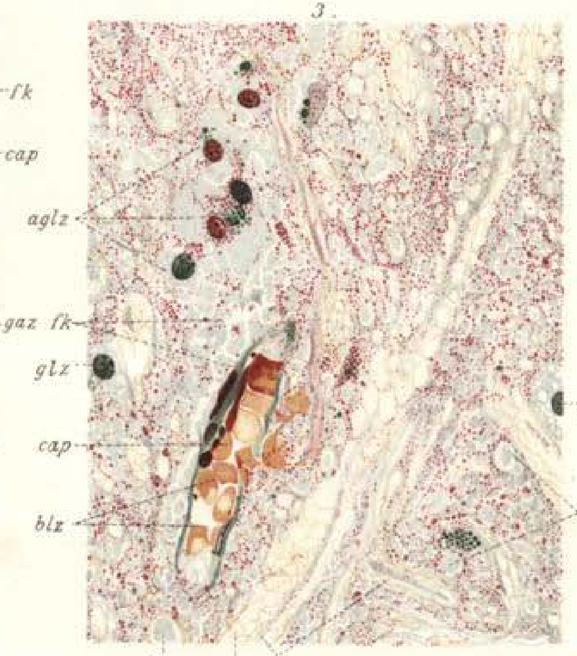

4.

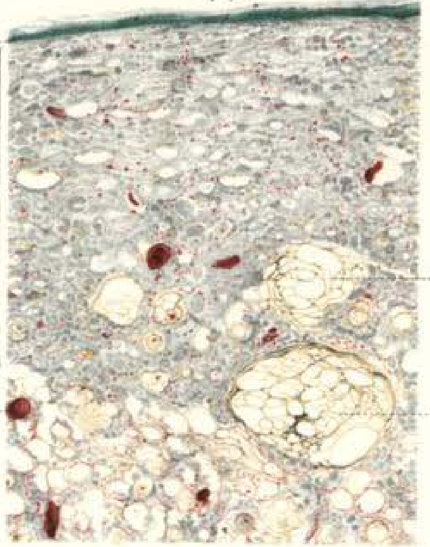

5.

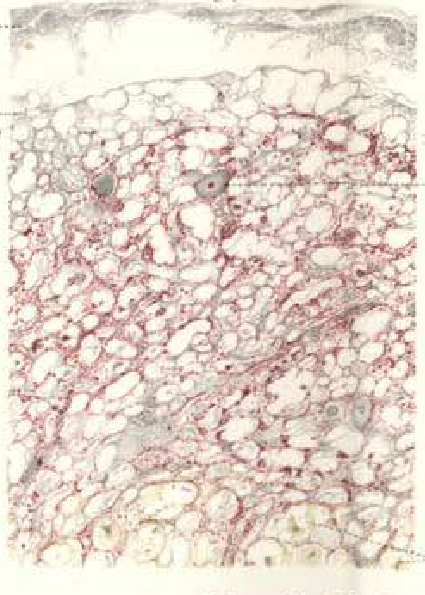

6.

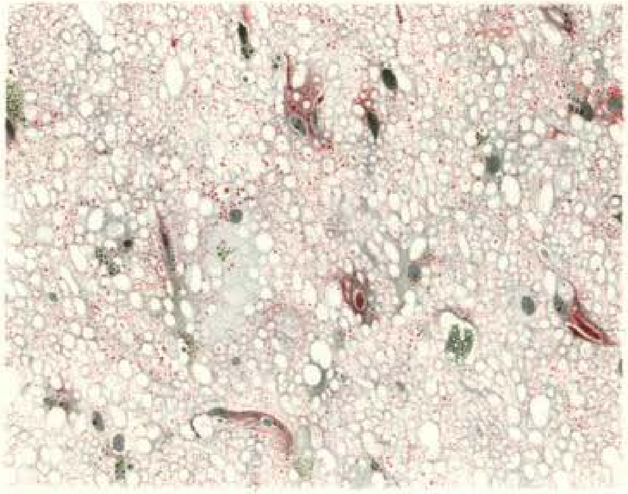

gez. Dirr.

Verlag von Gustav Fischer in Jena

Lith. Anst. v. A. Giltsch, Jena.
